# Supplementary material for: Comparison of Experimental Methodologies Based on Bulk-Metagenome and Virus-like Particle Enrichment: Pros and Cons for Representativeness and Reproducibility in the Study of the Fecal Human Virome
Source: Microorganisms. 2024 Jan 13;12(1):162. doi: 10.3390/microorganisms12010162 (PMC10820677; doi:10.3390/microorganisms12010162)
Supplement: Supplementary file 1 [file microorganisms-12-00162-s001.zip › Figure S1.pdf]

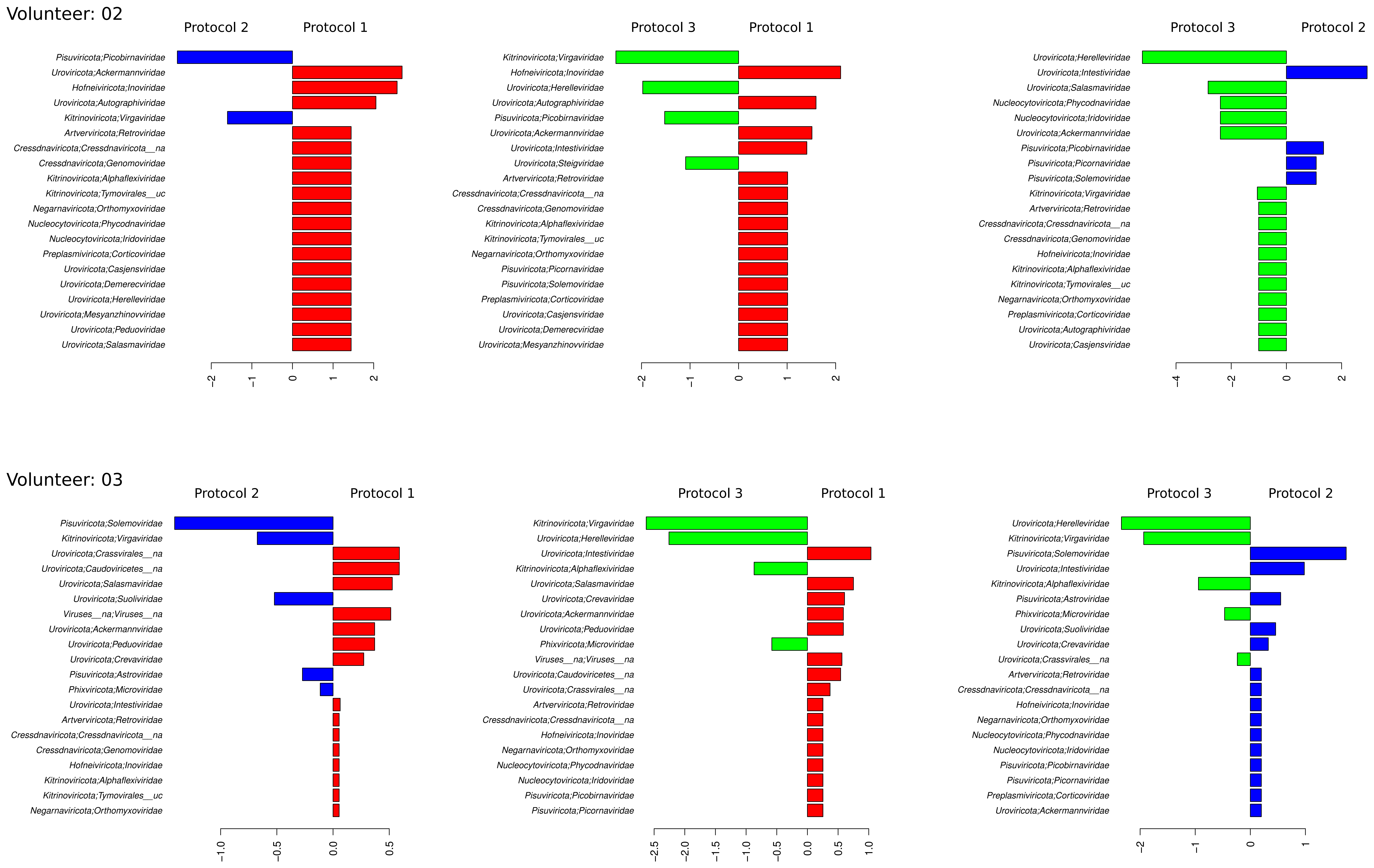

Volunteer: 02
Protocol 2
Protocol 1
Pisuviricota;Picobirnaviridae
Uroviricota;Ackermannviridae
Hofneiviricota;Inoviridae
Uroviricota;Autographiviridae
Kitrinoviricota;Virgaviridae
Artverviricota;Retroviridae
Cressdnaviricota;Cressdnaviricota__na
Cressdnaviricota;Genomoviridae
Kitrinoviricota;Alphaflexiviridae
Kitrinoviricota;Tymovirales__uc
Negarnaviricota;Orthomyxoviridae
Nucleocytoviricota;Phycodnaviridae
Nucleocytoviricota;Iridoviridae
Preplasmiviricota;Corticoviridae
Uroviricota;Casjensviridae
Uroviricota;Demerecviridae
Uroviricota;Herelleviridae
Uroviricota;Mesyanzhinovviridae
Uroviricota;Peduoviridae
Uroviricota;Salasmaviridae
−2 −1 0 1 2
Protocol 3
Protocol 1
Kitrinoviricota;Virgaviridae
Hofneiviricota;Inoviridae
Uroviricota;Herelleviridae
Uroviricota;Autographiviridae
Pisuviricota;Picobirnaviridae
Uroviricota;Ackermannviridae
Uroviricota;Intestiviridae
Uroviricota;Steigviridae
Artverviricota;Retroviridae
Cressdnaviricota;Cressdnaviricota__na
Cressdnaviricota;Genomoviridae
Kitrinoviricota;Alphaflexiviridae
Kitrinoviricota;Tymovirales__uc
Negarnaviricota;Orthomyxoviridae
Pisuviricota;Picornaviridae
Pisuviricota;Solemoviridae
Preplasmiviricota;Corticoviridae
Uroviricota;Casjensviridae
Uroviricota;Demerecviridae
Uroviricota;Mesyanzhinovviridae
−2 −1 0 1 2
Protocol 3
Protocol 2
Uroviricota;Herelleviridae
Uroviricota;Intestiviridae
Uroviricota;Salasmaviridae
Nucleocytoviricota;Phycodnaviridae
Nucleocytoviricota;Iridoviridae
Uroviricota;Ackermannviridae
Pisuviricota;Picobirnaviridae
Pisuviricota;Picornaviridae
Pisuviricota;Solemoviridae
Kitrinoviricota;Virgaviridae
Artverviricota;Retroviridae
Cressdnaviricota;Cressdnaviricota__na
Cressdnaviricota;Genomoviridae
Hofneiviricota;Inoviridae
Kitrinoviricota;Alphaflexiviridae
Kitrinoviricota;Tymovirales__uc
Negarnaviricota;Orthomyxoviridae
Preplasmiviricota;Corticoviridae
Uroviricota;Autographiviridae
Uroviricota;Casjensviridae
−4 −2 0 2
Volunteer: 03
Protocol 2
Protocol 1
Pisuviricota;Solemoviridae
Kitrinoviricota;Virgaviridae
Uroviricota;Crassvirales__na
Uroviricota;Caudoviricetes__na
Uroviricota;Salasmaviridae
Uroviricota;Suoliviridae
Viruses__na;Viruses__na
Uroviricota;Ackermannviridae
Uroviricota;Peduoviridae
Uroviricota;Crevaviridae
Pisuviricota;Astroviridae
Phixviricota;Microviridae
Uroviricota;Intestiviridae
Artverviricota;Retroviridae
Cressdnaviricota;Cressdnaviricota__na
Cressdnaviricota;Genomoviridae
Hofneiviricota;Inoviridae
Kitrinoviricota;Alphaflexiviridae
Kitrinoviricota;Tymovirales__uc
Negarnaviricota;Orthomyxoviridae
−1.0 −0.5 0.0 0.5
Protocol 3
Protocol 1
Kitrinoviricota;Virgaviridae
Uroviricota;Herelleviridae
Uroviricota;Intestiviridae
Kitrinoviricota;Alphaflexiviridae
Uroviricota;Salasmaviridae
Uroviricota;Crevaviridae
Uroviricota;Ackermannviridae
Uroviricota;Peduoviridae
Phixviricota;Microviridae
Viruses__na;Viruses__na
Uroviricota;Caudoviricetes__na
Uroviricota;Crassvirales__na
Artverviricota;Retroviridae
Cressdnaviricota;Cressdnaviricota__na
Hofneiviricota;Inoviridae
Negarnaviricota;Orthomyxoviridae
Nucleocytoviricota;Phycodnaviridae
Nucleocytoviricota;Iridoviridae
Pisuviricota;Picobirnaviridae
Pisuviricota;Picornaviridae
−2.5 −2.0 −1.5 −1.0 −0.5 0.0 0.5 1.0
Protocol 3
Protocol 2
Uroviricota;Herelleviridae
Kitrinoviricota;Virgaviridae
Pisuviricota;Solemoviridae
Uroviricota;Intestiviridae
Kitrinoviricota;Alphaflexiviridae
Pisuviricota;Astroviridae
Phixviricota;Microviridae
Uroviricota;Suoliviridae
Uroviricota;Crevaviridae
Uroviricota;Crassvirales__na
Artverviricota;Retroviridae
Cressdnaviricota;Cressdnaviricota__na
Hofneiviricota;Inoviridae
Negarnaviricota;Orthomyxoviridae
Nucleocytoviricota;Phycodnaviridae
Nucleocytoviricota;Iridoviridae
Pisuviricota;Picobirnaviridae
Pisuviricota;Picornaviridae
Preplasmiviricota;Corticoviridae
Uroviricota;Ackermannviridae
−2 −1 0 1

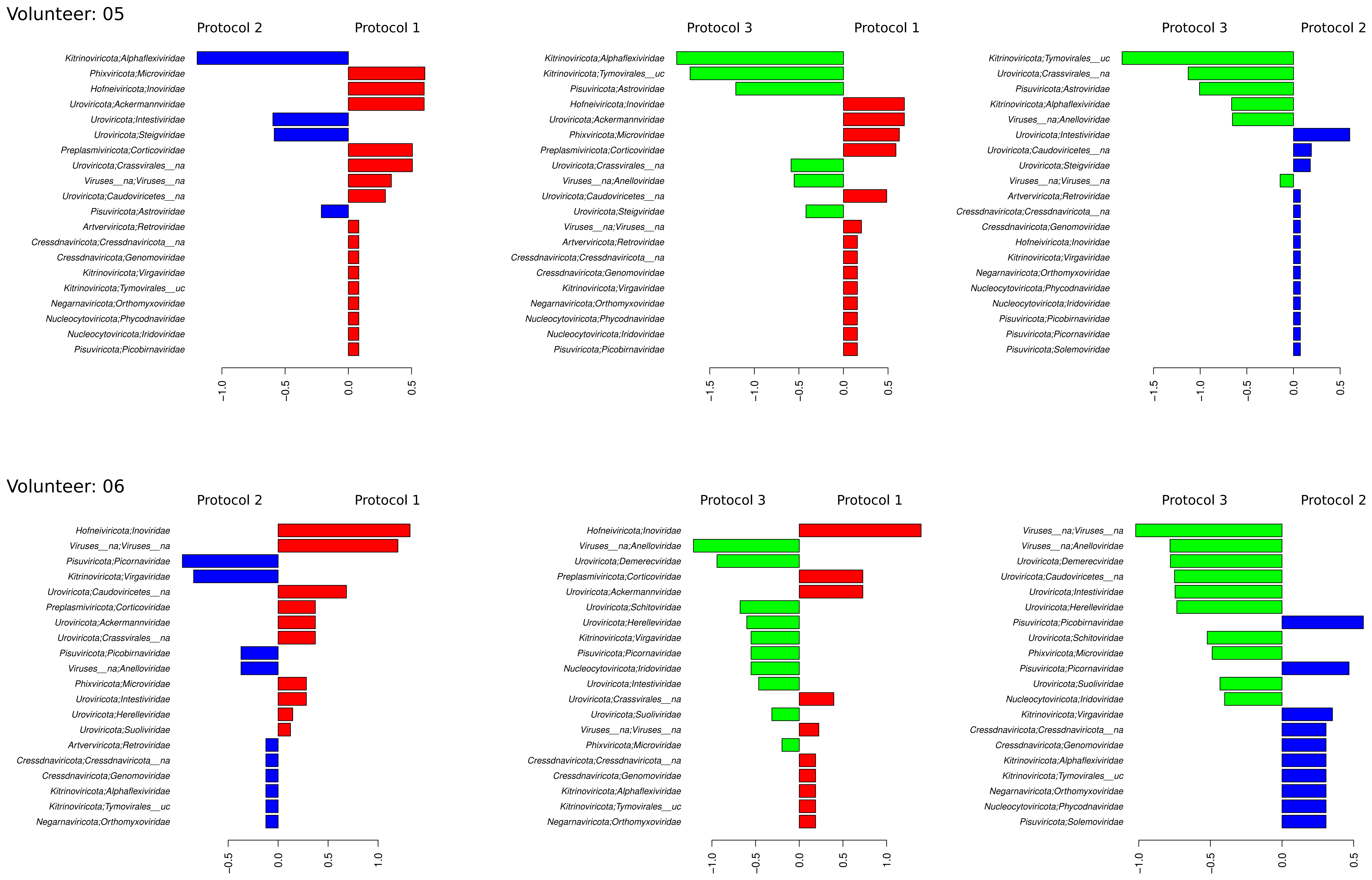

Volunteer: 05
Protocol 2
Protocol 1
Kitrinoviricota;Alphaflexiviridae
Phixviricota;Microviridae
Hofneiviricota;Inoviridae
Uroviricota;Ackermannviridae
Uroviricota;Intestiviridae
Uroviricota;Steigviridae
Preplasmiviricota;Corticoviridae
Uroviricota;Crassvirales__na
Viruses__na;Viruses__na
Uroviricota;Caudoviricetes__na
Pisuviricota;Astroviridae
Artverviricota;Retroviridae
Cressdnaviricota;Cressdnaviricota__na
Cressdnaviricota;Genomoviridae
Kitrinoviricota;Virgaviridae
Kitrinoviricota;Tymovirales__uc
Negarnaviricota;Orthomyxoviridae
Nucleocytoviricota;Phycodnaviridae
Nucleocytoviricota;Iridoviridae
Pisuviricota;Picobirnaviridae
-1.0
-0.5
0.0
0.5
Protocol 3
Protocol 1
Kitrinoviricota;Alphaflexiviridae
Kitrinoviricota;Tymovirales__uc
Pisuviricota;Astroviridae
Hofneiviricota;Inoviridae
Uroviricota;Ackermannviridae
Phixviricota;Microviridae
Preplasmiviricota;Corticoviridae
Uroviricota;Crassvirales__na
Viruses__na;Anelloviridae
Uroviricota;Caudoviricetes__na
Uroviricota;Steigviridae
Viruses__na;Viruses__na
Artverviricota;Retroviridae
Cressdnaviricota;Cressdnaviricota__na
Cressdnaviricota;Genomoviridae
Kitrinoviricota;Virgaviridae
Negarnaviricota;Orthomyxoviridae
Nucleocytoviricota;Phycodnaviridae
Nucleocytoviricota;Iridoviridae
Pisuviricota;Picobirnaviridae
-1.5
-1.0
-0.5
0.0
0.5
Protocol 3
Protocol 2
Kitrinoviricota;Tymovirales__uc
Uroviricota;Crassvirales__na
Pisuviricota;Astroviridae
Kitrinoviricota;Alphaflexiviridae
Viruses__na;Anelloviridae
Uroviricota;Intestiviridae
Uroviricota;Caudoviricetes__na
Uroviricota;Steigviridae
Viruses__na;Viruses__na
Artverviricota;Retroviridae
Cressdnaviricota;Cressdnaviricota__na
Cressdnaviricota;Genomoviridae
Hofneiviricota;Inoviridae
Kitrinoviricota;Virgaviridae
Negarnaviricota;Orthomyxoviridae
Nucleocytoviricota;Phycodnaviridae
Nucleocytoviricota;Iridoviridae
Pisuviricota;Picobirnaviridae
Pisuviricota;Picornaviridae
Pisuviricota;Solemoviridae
-1.5
-1.0
-0.5
0.0
0.5
Volunteer: 06
Protocol 2
Protocol 1
Hofneiviricota;Inoviridae
Viruses__na;Viruses__na
Pisuviricota;Picornaviridae
Kitrinoviricota;Virgaviridae
Uroviricota;Caudoviricetes__na
Preplasmiviricota;Corticoviridae
Uroviricota;Ackermannviridae
Uroviricota;Crassvirales__na
Pisuviricota;Picobirnaviridae
Viruses__na;Anelloviridae
Phixviricota;Microviridae
Uroviricota;Intestiviridae
Uroviricota;Herelleviridae
Uroviricota;Suoliviridae
Artverviricota;Retroviridae
Cressdnaviricota;Cressdnaviricota__na
Cressdnaviricota;Genomoviridae
Kitrinoviricota;Alphaflexiviridae
Kitrinoviricota;Tymovirales__uc
Negarnaviricota;Orthomyxoviridae
-0.5
0.0
0.5
1.0
Protocol 3
Protocol 1
Hofneiviricota;Inoviridae
Viruses__na;Anelloviridae
Uroviricota;Demerecviridae
Preplasmiviricota;Corticoviridae
Uroviricota;Ackermannviridae
Uroviricota;Schitoviridae
Uroviricota;Herelleviridae
Kitrinoviricota;Virgaviridae
Pisuviricota;Picornaviridae
Nucleocytoviricota;Iridoviridae
Uroviricota;Intestiviridae
Uroviricota;Crassvirales__na
Uroviricota;Suoliviridae
Viruses__na;Viruses__na
Phixviricota;Microviridae
Cressdnaviricota;Cressdnaviricota__na
Cressdnaviricota;Genomoviridae
Kitrinoviricota;Alphaflexiviridae
Kitrinoviricota;Tymovirales__uc
Negarnaviricota;Orthomyxoviridae
-1.0
-0.5
0.0
0.5
1.0
Protocol 3
Protocol 2
Viruses__na;Viruses__na
Viruses__na;Anelloviridae
Uroviricota;Demerecviridae
Uroviricota;Caudoviricetes__na
Uroviricota;Intestiviridae
Uroviricota;Herelleviridae
Pisuviricota;Picobirnaviridae
Uroviricota;Schitoviridae
Phixviricota;Microviridae
Pisuviricota;Picornaviridae
Uroviricota;Suoliviridae
Nucleocytoviricota;Iridoviridae
Kitrinoviricota;Virgaviridae
Cressdnaviricota;Cressdnaviricota__na
Cressdnaviricota;Genomoviridae
Kitrinoviricota;Alphaflexiviridae
Kitrinoviricota;Tymovirales__uc
Negarnaviricota;Orthomyxoviridae
Nucleocytoviricota;Phycodnaviridae
Pisuviricota;Solemoviridae
-1.0
-0.5
0.0
0.5

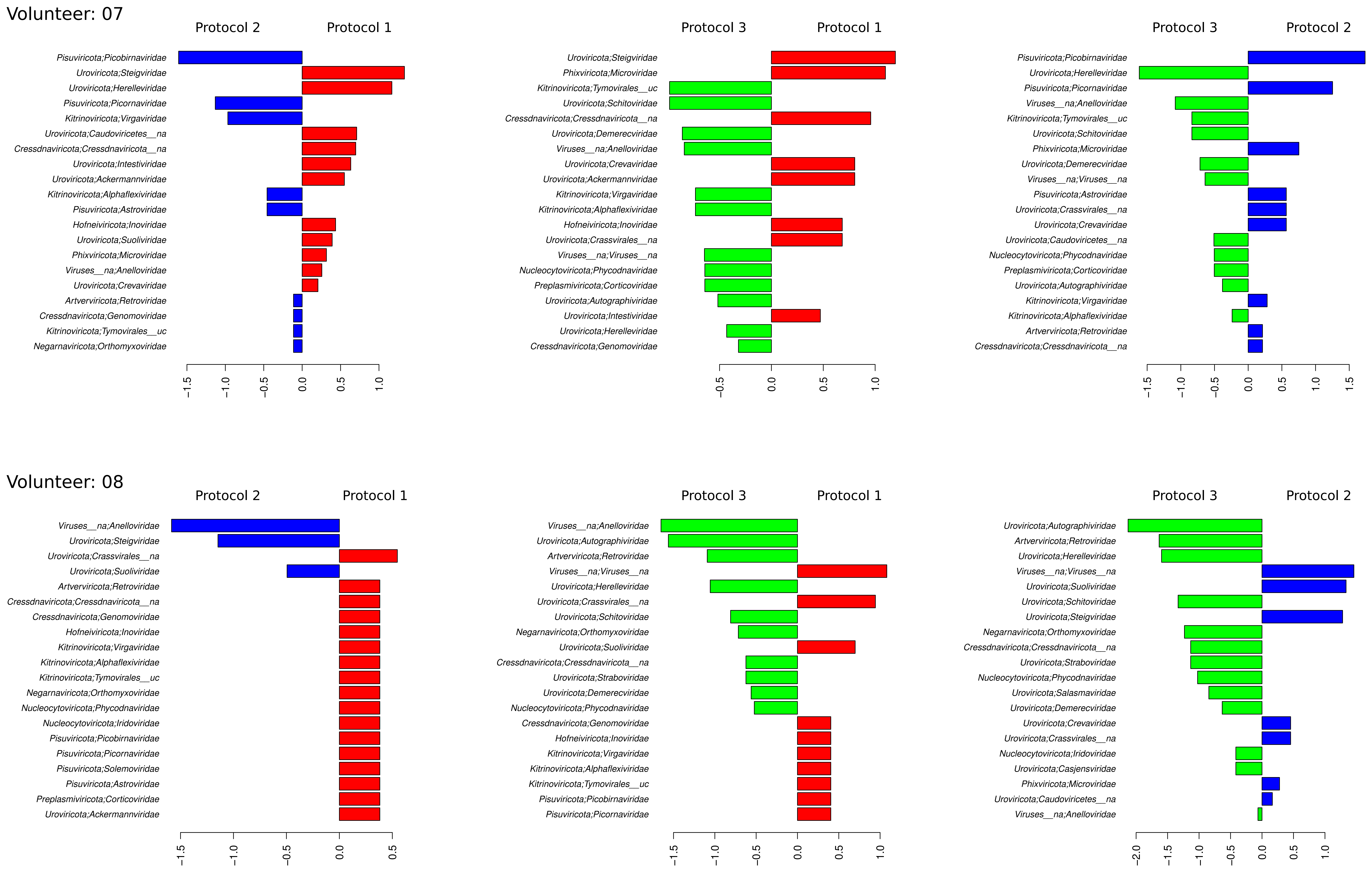

Volunteer: 07
Protocol 2
Protocol 1
Pisuviricota;Picobirnaviridae
Uroviricota;Steigviridae
Uroviricota;Herelleviridae
Pisuviricota;Picornaviridae
Kitrinoviricota;Virgaviridae
Uroviricota;Caudoviricetes__na
Cressdnaviricota;Cressdnaviricota__na
Uroviricota;Intestiviridae
Uroviricota;Ackermannviridae
Kitrinoviricota;Alphaflexiviridae
Pisuviricota;Astroviridae
Hofneiviricota;Inoviridae
Uroviricota;Suoliviridae
Phixviricota;Microviridae
Viruses__na;Anelloviridae
Uroviricota;Crevaviridae
Artverviricota;Retroviridae
Cressdnaviricota;Genomoviridae
Kitrinoviricota;Tymovirales__uc
Negarnaviricota;Orthomyxoviridae
-1.5
-1.0
-0.5
0.0
0.5
1.0
Protocol 3
Protocol 1
Uroviricota;Steigviridae
Phixviricota;Microviridae
Kitrinoviricota;Tymovirales__uc
Uroviricota;Schitoviridae
Cressdnaviricota;Cressdnaviricota__na
Uroviricota;Demerecviridae
Viruses__na;Anelloviridae
Uroviricota;Crevaviridae
Uroviricota;Ackermannviridae
Kitrinoviricota;Virgaviridae
Kitrinoviricota;Alphaflexiviridae
Hofneiviricota;Inoviridae
Uroviricota;Crassvirales__na
Viruses__na;Viruses__na
Nucleocytoviricota;Phycodnaviridae
Preplasmiviricota;Corticoviridae
Uroviricota;Autographiviridae
Uroviricota;Intestiviridae
Uroviricota;Herelleviridae
Cressdnaviricota;Genomoviridae
-0.5
0.0
0.5
1.0
Protocol 3
Protocol 2
Pisuviricota;Picobirnaviridae
Uroviricota;Herelleviridae
Pisuviricota;Picornaviridae
Viruses__na;Anelloviridae
Kitrinoviricota;Tymovirales__uc
Uroviricota;Schitoviridae
Phixviricota;Microviridae
Uroviricota;Demerecviridae
Viruses__na;Viruses__na
Pisuviricota;Astroviridae
Uroviricota;Crassvirales__na
Uroviricota;Crevaviridae
Uroviricota;Caudoviricetes__na
Nucleocytoviricota;Phycodnaviridae
Preplasmiviricota;Corticoviridae
Uroviricota;Autographiviridae
Kitrinoviricota;Virgaviridae
Kitrinoviricota;Alphaflexiviridae
Artverviricota;Retroviridae
Cressdnaviricota;Cressdnaviricota__na
-1.5
-1.0
-0.5
0.0
0.5
1.0
1.5
Volunteer: 08
Protocol 2
Protocol 1
Viruses__na;Anelloviridae
Uroviricota;Steigviridae
Uroviricota;Crassvirales__na
Uroviricota;Suoliviridae
Artverviricota;Retroviridae
Cressdnaviricota;Cressdnaviricota__na
Cressdnaviricota;Genomoviridae
Hofneiviricota;Inoviridae
Kitrinoviricota;Virgaviridae
Kitrinoviricota;Alphaflexiviridae
Kitrinoviricota;Tymovirales__uc
Negarnaviricota;Orthomyxoviridae
Nucleocytoviricota;Phycodnaviridae
Nucleocytoviricota;Iridoviridae
Pisuviricota;Picobirnaviridae
Pisuviricota;Picornaviridae
Pisuviricota;Solemoviridae
Pisuviricota;Astroviridae
Preplasmiviricota;Corticoviridae
Uroviricota;Ackermannviridae
-1.5
-1.0
-0.5
0.0
0.5
Protocol 3
Protocol 1
Viruses__na;Anelloviridae
Uroviricota;Autographiviridae
Artverviricota;Retroviridae
Viruses__na;Viruses__na
Uroviricota;Herelleviridae
Uroviricota;Crassvirales__na
Uroviricota;Schitoviridae
Negarnaviricota;Orthomyxoviridae
Uroviricota;Suoliviridae
Cressdnaviricota;Cressdnaviricota__na
Uroviricota;Straboviridae
Uroviricota;Demerecviridae
Nucleocytoviricota;Phycodnaviridae
Cressdnaviricota;Genomoviridae
Hofneiviricota;Inoviridae
Kitrinoviricota;Virgaviridae
Kitrinoviricota;Alphaflexiviridae
Kitrinoviricota;Tymovirales__uc
Pisuviricota;Picobirnaviridae
Pisuviricota;Picornaviridae
-1.5
-1.0
-0.5
0.0
0.5
1.0
Protocol 3
Protocol 2
Uroviricota;Autographiviridae
Artverviricota;Retroviridae
Uroviricota;Herelleviridae
Viruses__na;Viruses__na
Uroviricota;Suoliviridae
Uroviricota;Schitoviridae
Uroviricota;Steigviridae
Negarnaviricota;Orthomyxoviridae
Cressdnaviricota;Cressdnaviricota__na
Uroviricota;Straboviridae
Nucleocytoviricota;Phycodnaviridae
Uroviricota;Salasmaviridae
Uroviricota;Demerecviridae
Uroviricota;Crevaviridae
Uroviricota;Crassvirales__na
Nucleocytoviricota;Iridoviridae
Uroviricota;Casjensviridae
Phixviricota;Microviridae
Uroviricota;Caudoviricetes__na
Viruses__na;Anelloviridae
-2.0
-1.5
-1.0
-0.5
0.0
0.5
1.0

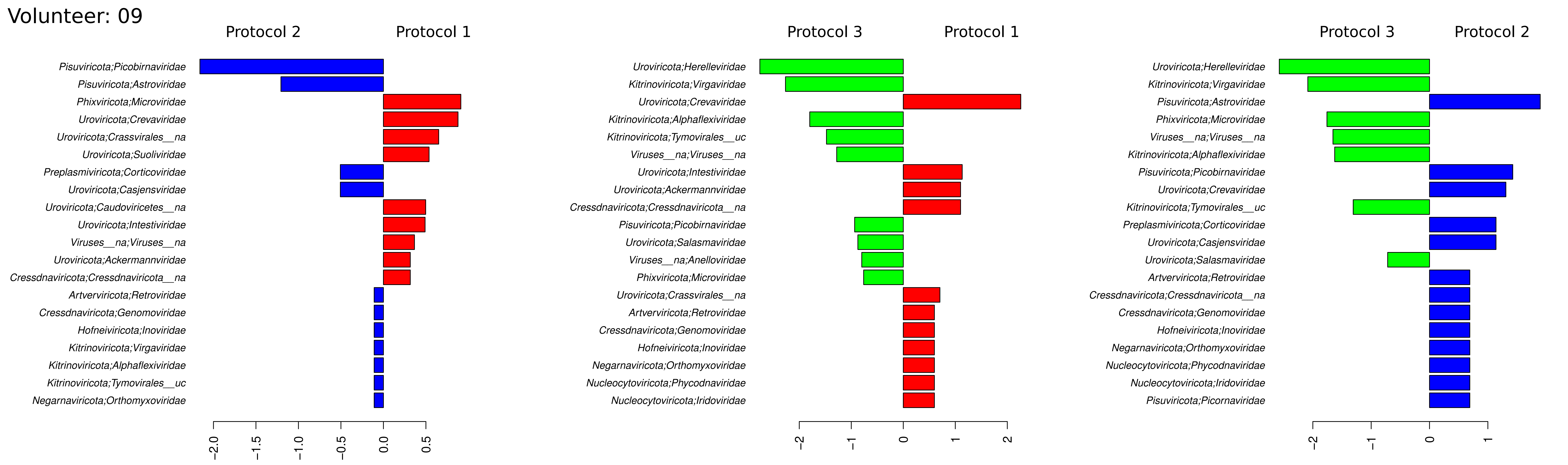
Volunteer: 09
Protocol 2
Protocol 1
Pisuviricota;Picobirnaviridae
Pisuviricota;Astroviridae
Phixviricota;Microviridae
Uroviricota;Crevaviridae
Uroviricota;Crassvirales__na
Uroviricota;Suoliviridae
Preplasmiviricota;Corticoviridae
Uroviricota;Casjensviridae
Uroviricota;Caudoviricetes__na
Uroviricota;Intestiviridae
Viruses__na;Viruses__na
Uroviricota;Ackermannviridae
Cressdnaviricota;Cressdnaviricota__na
Artverviricota;Retroviridae
Cressdnaviricota;Genomoviridae
Hofneiviricota;Inoviridae
Kitrinoviricota;Virgaviridae
Kitrinoviricota;Alphaflexiviridae
Kitrinoviricota;Tymovirales__uc
Negarnaviricota;Orthomyxoviridae
-2.0
-1.5
-1.0
-0.5
0.0
0.5
Protocol 3
Protocol 1
Uroviricota;Herelleviridae
Kitrinoviricota;Virgaviridae
Uroviricota;Crevaviridae
Kitrinoviricota;Alphaflexiviridae
Kitrinoviricota;Tymovirales__uc
Viruses__na;Viruses__na
Uroviricota;Intestiviridae
Uroviricota;Ackermannviridae
Cressdnaviricota;Cressdnaviricota__na
Pisuviricota;Picobirnaviridae
Uroviricota;Salasmaviridae
Viruses__na;Anelloviridae
Phixviricota;Microviridae
Uroviricota;Crassvirales__na
Artverviricota;Retroviridae
Cressdnaviricota;Genomoviridae
Hofneiviricota;Inoviridae
Negarnaviricota;Orthomyxoviridae
Nucleocytoviricota;Phycodnaviridae
Nucleocytoviricota;Iridoviridae
-2
-1
0
1
2
Protocol 3
Protocol 2
Uroviricota;Herelleviridae
Kitrinoviricota;Virgaviridae
Pisuviricota;Astroviridae
Phixviricota;Microviridae
Viruses__na;Viruses__na
Kitrinoviricota;Alphaflexiviridae
Pisuviricota;Picobirnaviridae
Uroviricota;Crevaviridae
Kitrinoviricota;Tymovirales__uc
Preplasmiviricota;Corticoviridae
Uroviricota;Casjensviridae
Uroviricota;Salasmaviridae
Artverviricota;Retroviridae
Cressdnaviricota;Cressdnaviricota__na
Cressdnaviricota;Genomoviridae
Hofneiviricota;Inoviridae
Negarnaviricota;Orthomyxoviridae
Nucleocytoviricota;Phycodnaviridae
Nucleocytoviricota;Iridoviridae
Pisuviricota;Picornaviridae
-2
-1
0
1

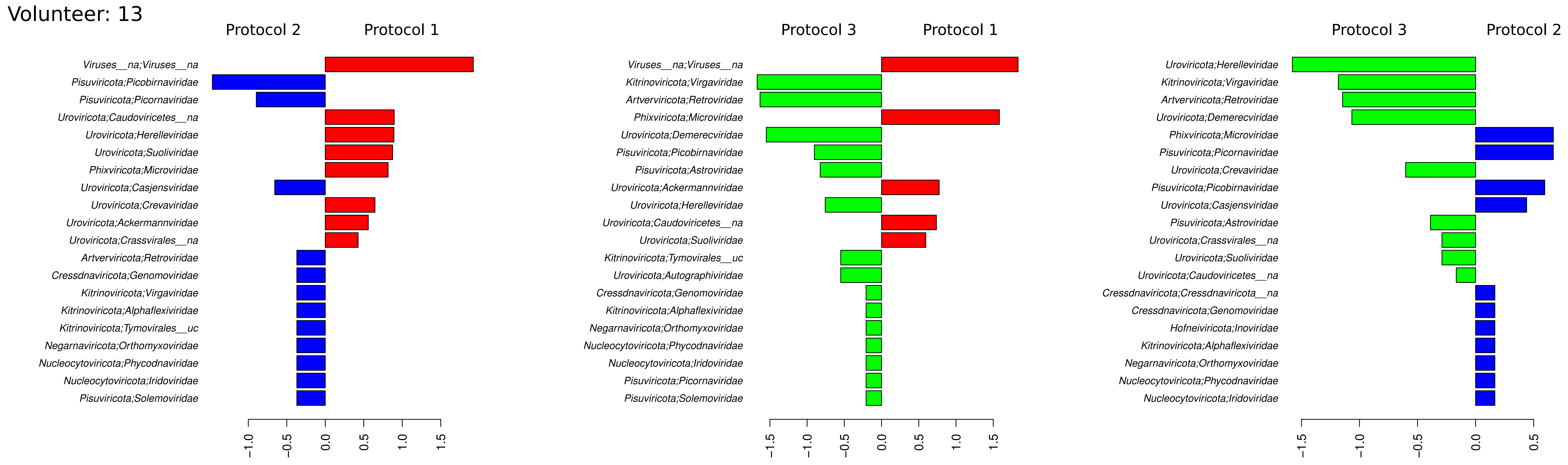
Volunteer: 13
Protocol 2
Protocol 1
Viruses__na;Viruses__na
Pisuviricota;Picobirnaviridae
Pisuviricota;Picornaviridae
Uroviricota;Caudoviricetes__na
Uroviricota;Herelleviridae
Uroviricota;Suoliviridae
Phixviricota;Microviridae
Uroviricota;Casjensviridae
Uroviricota;Crevaviridae
Uroviricota;Ackermannviridae
Uroviricota;Crassvirales__na
Artverviricota;Retroviridae
Cressdnaviricota;Genomoviridae
Kitrinoviricota;Virgaviridae
Kitrinoviricota;Alphaflexiviridae
Kitrinoviricota;Tymovirales__uc
Negarnaviricota;Orthomyxoviridae
Nucleocytoviricota;Phycodnaviridae
Nucleocytoviricota;Iridoviridae
Pisuviricota;Solemoviridae
-1.0
-0.5
0.0
0.5
1.0
1.5
Protocol 3
Protocol 1
Viruses__na;Viruses__na
Kitrinoviricota;Virgaviridae
Artverviricota;Retroviridae
Phixviricota;Microviridae
Uroviricota;Demerecviridae
Pisuviricota;Picobirnaviridae
Pisuviricota;Astroviridae
Uroviricota;Ackermannviridae
Uroviricota;Herelleviridae
Uroviricota;Caudoviricetes__na
Uroviricota;Suoliviridae
Kitrinoviricota;Tymovirales__uc
Uroviricota;Autographiviridae
Cressdnaviricota;Genomoviridae
Kitrinoviricota;Alphaflexiviridae
Negarnaviricota;Orthomyxoviridae
Nucleocytoviricota;Phycodnaviridae
Nucleocytoviricota;Iridoviridae
Pisuviricota;Picornaviridae
Pisuviricota;Solemoviridae
-1.5
-1.0
-0.5
0.0
0.5
1.0
1.5
Protocol 3
Protocol 2
Uroviricota;Herelleviridae
Kitrinoviricota;Virgaviridae
Artverviricota;Retroviridae
Uroviricota;Demerecviridae
Phixviricota;Microviridae
Pisuviricota;Picornaviridae
Uroviricota;Crevaviridae
Pisuviricota;Picobirnaviridae
Uroviricota;Casjensviridae
Pisuviricota;Astroviridae
Uroviricota;Crassvirales__na
Uroviricota;Suoliviridae
Uroviricota;Caudoviricetes__na
Cressdnaviricota;Cressdnaviricota__na
Cressdnaviricota;Genomoviridae
Hofneiviricota;Inoviridae
Kitrinoviricota;Alphaflexiviridae
Negarnaviricota;Orthomyxoviridae
Nucleocytoviricota;Phycodnaviridae
Nucleocytoviricota;Iridoviridae
-1.5
-1.0
-0.5
0.0
0.5

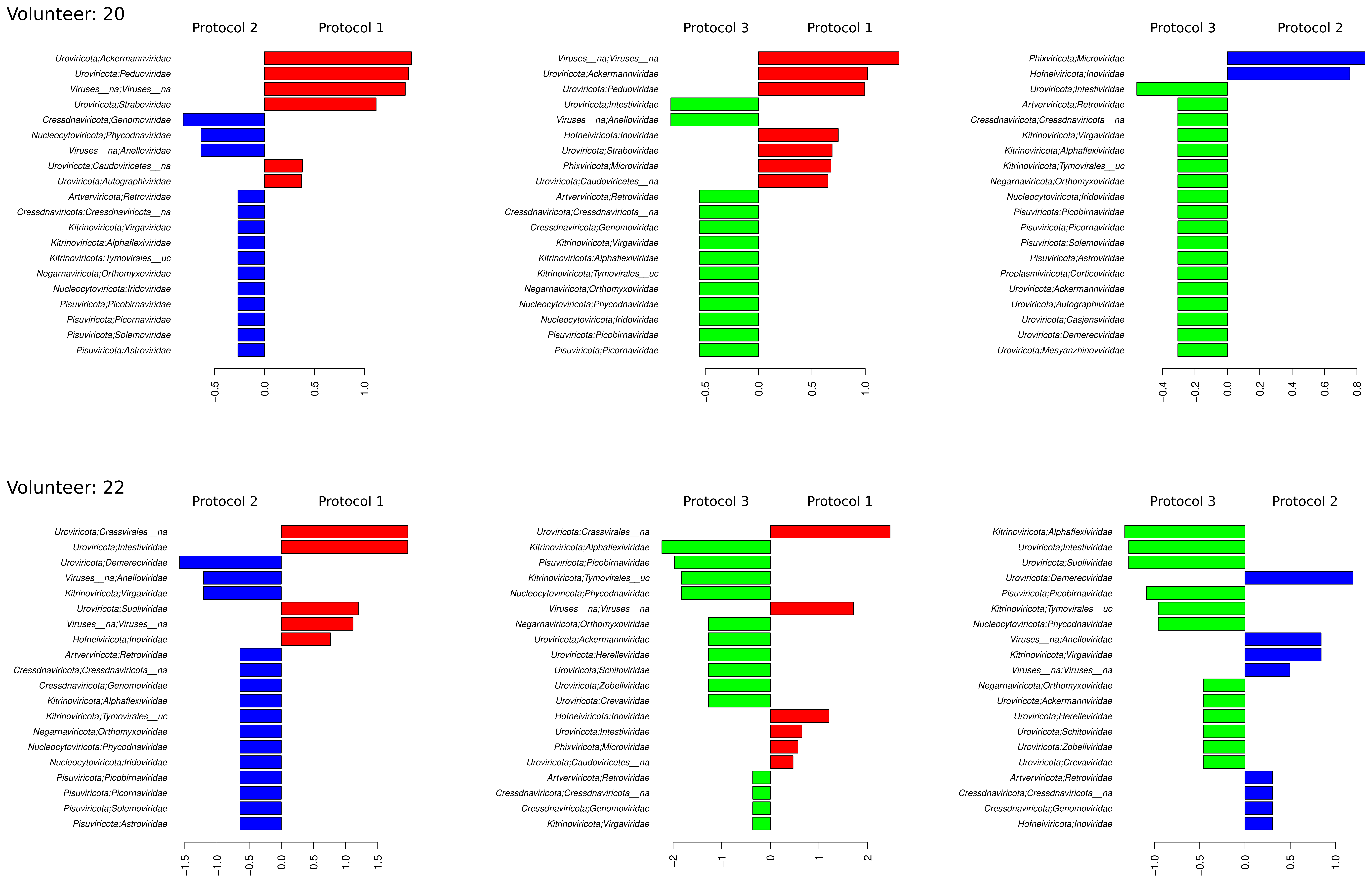

Figure S1. Bar graph showing the log2FC values for differences in the viral composition of each volunteer attributed to protocol variation (Protocol 1: red, Protocol 2: blue, Protocol 3: green).
