# Supplementary material for: Comparison of Experimental Methodologies Based on Bulk-Metagenome and Virus-like Particle Enrichment: Pros and Cons for Representativeness and Reproducibility in the Study of the Fecal Human Virome
Source: Microorganisms. 2024 Jan 13;12(1):162. doi: 10.3390/microorganisms12010162 (PMC10820677; doi:10.3390/microorganisms12010162)
Supplement: Supplementary file 1 [file microorganisms-12-00162-s001.zip › Table S2.pdf]

**Table S2.** Relative abundance of the viral families identified for each sample in the study, used for Figure 3.

Taxon 02.1A 02.1B 02.2A 02.2B 02.3A 02.3B 03.1A 03.1B 03.2A 03.2B 03.3A 03.3B 05.1A
05.1B 05.2A 05.2B 05.3A 05.3B 06.1A 06.1B 06.2A 06.2B 06.3A 06.3B 07.1A 07.1B
07.2A 07.2B 07.3A 07.3B 08.1A 08.1B 08.2A 08.2B 08.3A 08.3B 09.1A 09.1B 09.2A
09.2B 09.3A 09.3B 13.1A 13.1B 13.2A 13.2B 13.3A 13.3B 20.1A 20.1B 20.2A 20.2B
20.3A 20.3B 22.1A 22.1B 22.2A 22.2B 22.3A 22.3B
"Artverviricota;Caulimoviridae" 0 0 0 0 0 0 0 0 0 0
0 0 0 0 0 0 0 0 0 0 0 0 0
8.461538462 0 0 0 0 0 0.008289812 0 0 0 0
0 0 0 0 0 0 0 0 0 0 0 0 0
0 0 0 0 0 0 0 0 0 0 0 0 0
"Artverviricota*;Retroviridae" 0 0 0 0 0 0 0 0 0 0
0 0 0 0 0 0 0 0 0 0 0 0 0
0.384615385 0 0 0 0 0 0 0 0 0 0
2.906208719 0 0 0 0 0 0 0 0 0 0 0
0 19.31540342 0 0 0 0 0 0 0 0 0 0
0 0
"Cossaviricota;Papillomaviridae" 0 0 0 0 0 0 0 0 0 0
0 0 0 0 0 0 0 0 0 0 0 0 0
1.538461538 0 0 0 0 0 0.953328359 0 0 0 0
0 0 0 0 0 0 0 0 0 0 0 0 0
0 0 0 0 0 0 0 0 0 0 0 0 0
"Cossaviricota*;Parvoviridae" 0 0 0 0 0 0 0 0 0 0
0 0 0 0 0 0 0 0 0 0 0 0 0
0 0 0 0 0 0 0 0 0 0 0 0
4.022988506 0 0 0 0 0 0 0 0 0 0 0
0 0 0 0 0 0 0 0 0 0 0 0 0
"Cressdnaviricota;Circoviridae" 0 0 0 0 0 0 0 0 0 0
0 0 0 0 0 0 0 0 0 0 0 0 0
0 0 0 0 0 0 0 0 0 0 0 0
0.574712644 0 0 0 0 0 0 0 0 0 0 0
0 0 0 0 0 0 0 0 0 0 0 0 0
"Cressdnaviricota;Cressdnaviricota__na" 0 0 0 0 0 0 0 0 0
0 0 0 0 0 0 0 0 0 0 0 0 0
0 0 0 0.534188034 0 0 0 0 0 0 0 0
0 5.747126437 0.084317032 0 0 0 0 0 0 0.069348128
0 0 0 0 0 0 0 0 0 0 0 0 0
0 0 0
"Cressdnaviricota;Genomoviridae" 0 0 0 0 0 0 0 0 0 0
0.086956522 0 0 0 0 0 0 0 0 0 0 0
0 0 0 0 0 0 0 0.008289812 0 0 0 0
0 0 0 0 0 0 0 0 0 0 0 0 0
0 0 0 0 0.930232558 0 0 0 0 0 0 0
0
"Duplornaviricota;Chrysoviridae" 0 0 0 0 0 0 0 0 0 0
0 0 0 0 0 0 0 0 0 0 0 0 0
0 0 0 0 0 0 0 0 0 0 0 0 0
0 0 0 0 0 0 0 0 0 0 0 0 0
0 0 0 0 0 0 0 0 0.847457627 0 0
"Hofneiviricota;Inoviridae" 0.130378096 0.457456542 0 0 0 0 0 0
0 0 0 0 0.174216028 0.165837479 0 0 0 0
2.559726962 3.184713376 0 0 0.019928258 0 0 0.213675214 0
0 0 0 0 0 0 0 0 0 0 0 0 0
0 0 0 0.069348128 0 0 0 0 0.199004975 0.99009901
3.092783505 2.325581395 0 0 0 0.762195122 0 0 0 0
"Kitrinoviricota;Virgaviridae" 0 0 0.445599703 0.8057676 34.75853584 2.964872704
0 0 0 5.747126437 38.52173913 96.02577873 0 0 0 0

0 0 0 0 16.66666667 0 0.039856517 0.384615385 0 0
2.272727273 1.19047619 0 0.033159247 0 0 0 0 0 0
0 0 0 0 14.83979764 0.262123198 0 0 0 0 0
23.47188264 0 0 0 0 0 0 0 0 0 1.694915254
0 0
"Kitrinoviricota;Alphaflexiviridae" 0 0 0 0 0 0 0 0 0 0
0.086956522 0.161117078 0 0 7.326732673 1.333333333 13.67521368 51
0 0 0 0 0 0 0 0 0 1.19047619 0
0.033159247 0 0 0 0 0 0 0 0 0 0
18.04384486 0 0 0 0 0 0 0 0 0 0 0
0 0 0 0 0 0 4.516129032 0
"Kitrinoviricota;Betaflexiviridae" 0 0 0 0 0 0 0 0 0 0
0 0 0 0 0 0 0 0 0 0 0 0 0
0 0 0 0 0 0 0 0 0 0 0 0 0
0 0 0 0 0 0 0 0 0 0 0 0 0
0 2.06185567 0.465116279 0 0 0 0 0 0 0 0
"Kitrinoviricota;Tymovirales__na" 0 0 0 0 0 0 0 0 0 0
0 0 0 0 0.396039604 0 0 0 0 0 0 0
0 0 0 0 0 0 0 0 0 0 0 0 0
0 0 0 0 0 0 0 0 0 0 0 0 0
0 0 0 0 0 0 0 0 0 0 0
"Kitrinoviricota;Tymovirales__uc" 0 0 0 0 0 0 0 0 0 0
0 0.026852846 0 0 0 0 7.081807082 31 0 0 0
0 0 0 0 0 0 0 0 0.074608306 0 0 0
0 0 0 0 0 0 0 5.564924115 0 0 0 0
0 2.173913043 0 0 0 0 0 0 0 0 0 0
0 1.935483871 0
"Kitrinoviricota;Flaviviridae" 0 0 0 0 0 0 0 0 0 0
0 0 0 0 0 0 0 0 0 0 0 0 0
0.384615385 0 0 0 0 0 0 0 0 0 0
0.616468516 0 0 0 0 0 0 0 0 0 0 0
0 0 0 0 0 0 0 0 0 0 0 0 0
0
"Lenarviricota;Mitoviridae" 0 0 0 0 0 0 0 0 0 0
0 0 0 0 0 0 0 0 0 0 0 0 0
0 0 0 0.757575758 0 0 0 0 0 0 0 0
0 0 0 0.223214286 0 0 0 0 0 0 0 0
0 0 0 0 0 0 0 0 0 0 0 0 0
"Negarnaviricota;Orthomyxoviridae" 0 0 0 0 0 0 0 0 0
0 0 0 0 0 0 0 0 0 0 0 0 0
0 0 0 0 0 0 0 0.008289812 0 0 0 0
0.616468516 0 0 0 0 0 0 0 0 0 0 0
0 0 0 0 0 0 0 0 0 0 0
0.64516129 0
"Negarnaviricota;Pneumoviridae" 0 0 0 0 0 0 0 0 0 0
0 0 0 0 0 0 0 0 0 0 0 0 0
0 0 0 0 0 0 0 0 0 0 0 0 0
0 0 0 0 0 0 0 0 0 0 0
0.298507463 0 0 0 0 0 0 0 0 0 0 0
"Nucleocytoviricota;Phycodnaviridae" 0 0 0 0 0 0.032226877 0 0
0 0 0 0 0 0 0 0 0 0 0 0 0
0 0 0 0 0 0 0 0 0.024869435 0 0 0
0 0 4.022988506 0 0 0 0 0 0 0 0 0
0 0 0 0 0 1.030927835 0 0 0 0 0 0
0 1.935483871 0
"Nucleocytoviricota;Mimiviridae" 0 0 0 0 0 0 0 0 0 0
0 0 0 0 0 0 0 0 0 0 0 0 0
0 0 0 0 0 0 0 0 0 0 0 0 0

0 0 0 0 0 0 0.098425197 0 0 0 0 0
0 0 0 0 0 0 0 0 0 0 0 0
"Nucleocytoviricota;Iridoviridae" 0 0 0 0 0.030759766 0 0 0 0
0 0 0 0 0 0 0 0 0 0 0 0 0
0 1.923076923 0 0 0 0 0 0 0 0 0 0
0 0.574712644 0 0 0 0 0 0 0 0 0 0
0 0 0 0 0 0 0 0 0 0 0 0 0
0
"Nucleocytoviricota;Marseilleviridae" 0 0 0 0 0 0 0 0 0
0 0 0 0 0 0 0 0 0 0 0 0 0
0 0 0 0 0 0 0 0 0 0 0 0 0
3.448275862 0 0 0 0 0 0.02621232 0 0 0 0
0 0 0 0 0 0 0 0 0 0 0 0 0
0
"Nucleocytoviricota;Poxviridae" 0 0 0 0 0 0 0 0 0 0
0 0 0 0 0 0 0 0 0 0 0 0 0
0 0 0 0 0 0 0.008289812 0 0 0 0 0
0 0 0 0 0 0 0 0 0 0 0 0 0
0 0 0 0 0 0 0 0 0 0 0 0
"Phixviricota;Microviridae" 1.303780965 1.0978957 5.681396212 6.658184902 23.71577976
0.515630035 1.048218029 0.273972603 5.785123967 3.448275862 2.434782609
0.37593985 2.090592334 1.824212272 1.98019802 0 0.366300366 2 0
0.318471338 0 0 0.139497808 0 0.668576886 0.427350427 3.03030303
1.19047619 0 0.008289812 0.140449438 0.579374276 0.236183278 0.657894737
0 5.172413793 0.084317032 0.455927052 0 0 1.517706577 0.471821756
1.57480315 0.970873786 8.823529412 0 0 0 0.646766169 0.792079208
6.18556701 7.906976744 8.333333333 0 1.910219675 1.219512195 20.83333333
1.694915254 2.580645161 0
"Pisuviricota*;Partitiviridae" 0 0 0 0 0 0 0 0 0 0
0 0 0 0 0 0 0 0 0 0 0 0 0
0 0 0 0 0 0 0 0 0 0 0 0 0
0 0 0 0 0 0 0 0 0 0 0 0 0
0 0 0 0 0 0 0 0 0.847457627 0 0
"Pisuviricota*;Picobirnaviridae" 0 0 47.34496844 44.99575912 2.799138727 0.032226877
0 0 0 0 0 0 0 0 0 0 0 0 0
0 1.666666667 0 0 0 0 0 8.333333333 9.523809524 0
0 0 0 0 0 0 0 0 0 17.41071429 20.75471698
1.180438449 0 0 0 14.70588235 29.41176471 8.695652174 0 0
0 0 0 0 0 0 0 0 0 2.580645161 0
"Pisuviricota;Coronaviridae" 0 0 0 0 0 0 0 0 0 0
0 0 0 0 0 0 0 0 0 0 0 0 0
0 0 0 0 0 0 0 0 0 0.094473311 0
0.528401585 0 0 0 0 0 0 0 0 0 0 0
0 0 0 0 0 0 0 0 0 0 0 0 0
0
"Pisuviricota;Picornaviridae" 0 0 0 0.042408821 0 0 0 0 0
0 0 0 0 0 0 0 0 0 0 0 8.333333333
14.28571429 0.039856517 0.384615385 0 0 0.757575758 7.142857143 0
0 0 0 0 0 0 0 0 0 0 0 0 0
0 0 2.941176471 5.882352941 0 0 0 0 0 0 0
0 0 0 0 0 0 0
"Pisuviricota;Solemoviridae" 0 0 0 0.042408821 0 0 0 0
8.26446281 11.49425287 0 0 0 0 0 0 0 0 0
0 0 0 0 0 0 0 0 0 0 0 0 0
0 0 0 0 0 0 0 0 0 0 0 0 0
0 0 0 0 0 0 0 0 0 0 0 0 0
0 0
"Pisuviricota;Astroviridae" 0 0 0.074266617 0.042408821 0.030759766 0.032226877
0 0 0.826446281 0 0 0 0 0 0 1.333333333

1.953601954	4	0	0	0	0	0	0	0	0	0.757575758
0	0	0	0	0	0	0	0	0	0	0	0.223214286
9.433962264	0	0	0	0	0	0	6.52173913	0	0	0
0	0	0	0	0	0	0	0	0	0
"Preplasmiviricota;Adintoviridae"	0	0	0	0	0	0	0	0	0	0
0.086956522	0	0	0	0	0	0	0	0	0	0	0
0	0	0	0	0	0	0	0	0	0	0	0
0.574712644	0	0	0	0	0	0	0	0	0	0	0
0	0	0	0	0	0	0	0	0	0	0	0
"Preplasmiviricota;Corticoviridae"	0	0	0	0	0	0	0	0	0	0
0	0	0.348432056	0	0	0	0	0	0.170648464	0.159235669
0	0	0	0	0	0	0	0	0	0.024869435	0	0
0	0	0	0	0	0	0.223214286	0	0	0	0	0
0	0	0	0	0	0	0	0	0	0	0	0	0
0	0	0
"Uroviricota;Ackermannviridae"	0.651890482	0.182982617	0	0	0	0.032226877	0
0.136986301	0	0	0	0	0.174216028	0.165837479	0	0	0
0	0.170648464	0.159235669	0	0	0	0	0.286532951	0	0
0	0	0	0	0	0	0	0	0	0	0.151975684	0
0	0	0	0.196850394	0.138696255	0	0	0	0	0.945273632
0.792079208	0	0	0	0	0	0	0	0	0.64516129	0
"Uroviricota;Autographiviridae"	0	0.182982617	0	0	0	0	0	0	0
0	0	0	0	0	0	0	0	0	0	0	0	0
0.019928258	0	0	0	0	0	0	0.016579624	0	0	0
0	5.151915456	3.448275862	0	0	0	0	0	0.02621232	0
0	0	0	0	0.244498778	0.099502488	0	0	0	0	0
0.095510984	0	0	0	0	0
"Uroviricota;Casjensviridae"	0	0	0	0	0	0	0	0	0	0
0	0	0	0	0	0	0	0	0	0	0	0	0
0	0	0	0	0	0	0	0	0	0	0	0
0.574712644	0	0	0.223214286	0	0	0	0	0	0
5.882352941	0	0	0	0	0	0	0	0	0	0	0
0	0	0
"Uroviricota;Caudoviricetes__na"	81.3559322	88.38060384	13.99925733	15.30958439	15.01076592
43.50628424	86.16352201	80.54794521	38.84297521	37.93103448	6.086956522
1.235230934	95.12195122	91.37645108	63.56435644	80	62.75946276	8
94.88054608	93.31210191	70	85.71428571	98.14667198	69.23076923	93.21872015
86.11111111	29.54545455	38.0952381	45.45454545	14.00978198	84.97191011
90.03476246	37.12801134	30.92105263	15.58784676	42.52873563	43.50758853
73.70820669	21.875	58.49056604	10.96121417	76.22542595	87.5	88.07212205
73.52941176	58.82352941	19.56521739	29.82885086	93.9800995	94.05940594
87.62886598	86.51162791	75	98.88888889	73.63896848	74.69512195	62.5
83.89830508	60.64516129	0
"Uroviricota;Chaseviridae"	0	0	0	0	0	0	0	0	0	0
0.260869565	0	0	0	0	0	0	0	0	0	0	0
0	0	0	0	0	0	0	0	0	0	0	0	0
0	0	0	0	0	0	0	0	0	0	0	0	0
0	0	0	0	0	0	0	0	0	0	0	0
"Uroviricota;Demerecviridae"	0	0	0	0	0	0	0	0	0	0
0	0.026852846	0	0	0	0	0	0	0	0	0	0
0.019928258	4.230769231	0	0	0	0	0	0.049738871	0	0
0	0.219298246	0.352267723	0	0	0	0	0	0	0	0
0	0	0	0	12.95843521	0	0	0	0	0	0	0
0	4.166666667	2.542372881	0	0
"Uroviricota;Drexlerviridae"	0	0	0	0	0	0	0	0	0	0
0	0	0	0	0	0	0	0	0	0	0	0	0
0	0	0	0	0	0	0	0	0	0	0	0
1.149425287	0	0	0	0	0	0	0	0	0	0	0
0	0	0	0	0	0	0	0	0	0	0	0	0

"Uroviricota;Herelleviridae"	0	0	0	0	0.615195325	2.835965195	0	0
0	0	47.82608696	1.799140709	0	0	0	0	0	0	0
0.159235669	0	0	0.079713033	1.153846154	0.095510984	1.495726496	0
0	1.398601399	0.671474758	0	0	0	0	2.465874064	0	0
0	0	0	10.96121417	10.03931848	0.492125984	0.27739251	0	0
36.95652174	13.93643032	0.099502488	0.396039604	0	0.930232558	8.333333333
0	0	0	0	0	0.64516129	0
"Uroviricota;Kyanoviridae"	0	0	0	0	0	0	0	0	0	0
0	0	0	0	0	0	0	0	0	0	0	0	0
0	0	0	0	0	0	0	0	0	0.047236656	0	0
0	0	0	0	0	0	0	0	0	0	0	0	0
0	0	0	0	0	0	0	0	0	0	0	0
"Uroviricota;Mesyanzhinovviridae"	0	0	0	0	0	0	0	0	0
0	0.086956522	0	0	0	0	0	0	0	0	0	0
0	0.019928258	0	0	0	0	0	0	0.008289812	0	0
0	0	0	0	0	0	0	0	0	0	0	0	0
0	0	0	0	0	0	0	0	0	0	0	0	0
0	0
"Uroviricota;Pachyviridae"	0	0	0	0	0	0	0	0	0	0
0	0	0	0	0	0	0	0	0	0	0	0	0
0	0	0	0	0	0	0	0	0	0	0	0	0
0	0	0	0	0.168634064	0	0	0	0	0	0	0
0	0	0	0	0	0	0	0	0	0	0	0
"Uroviricota;Peduoviridae"	0	0	0	0	0	0	0.209643606	0	0
0	0	0	0	0	0	0	0	0	0	0	0	0
0	0	0	0	0	0	0	0	0	0	0	0	0
0	0	0	0	0	0	0	0	0	0	0	0	0
1.044776119	0.594059406	0	0	0	0	0	0	0	0	0
0
"Uroviricota;Rountreeviridae"	0	0	0	0	0	0	0	0	0	0
0	0	0	0	0	0	0	0	0	0	0	0	0
1.153846154	0	0	0	0	0	0	0	0	0	0	0
7.177454866	0	0	0	0	0	0	0	0	0	0	0
0	0	0	0	0	0	0	0	0	0	0	0	0
0
"Uroviricota;Salasmaviridae"	0	0	0	0	0	0.064453754	0	0.273972603
0	0	0	0	0	0	0	0	0	0	0	0	0
0	0	0	0	0	0	0	0	0	0	0	0	0
0	2.298850575	0	0	0	0	0	0.157273919	0.098425197	0
0	0	0	0	0	0	0	0	0	0	0.095510984	0
0	0	0	0
"Uroviricota;Schitoviridae"	0	0	0	0	0	0	0	0	0	0
0	0	0	0	0	0	0	0	0	0	0	0	0
3.076923077	0	0	0	0	0	0.074608306	0	0	0	0
0	11.49425287	0	0	0	0	0	0	0	0	0	0
0	0	0	0	0	0	0	0	0	0	0	0
0.64516129	0
"Uroviricota;Straboviridae"	0	0	0	0	0	0	0	0	0	0
0	0	0	0	0	0	0	0	0	0	0	0	0
0.384615385	0	0	0	0	0	0	0	0	0	0	0
5.747126437	0	0	0	0	0	0	0	0	0	0	0
0	0.348258706	0.396039604	0	0	0	0	0	0	0	0
0	0
"Uroviricota;Zierdtviridae"	0	0	0	0	0	0	0	0	0	0
0	0	0	0	0	0	0	0	0	0	0	0
0.298923874	0	0	0	0	0	0	0	0	0	0	0
0	0	0	0	0	0	0	0	0	0	0	0	0
0	0	0	0	0	0	0	0	0	0	0	0	0

"Uroviricota;Zobellviridae" 0 0 0 0 0 0 0 0 0 0
0 0.026852846 0 0 0 0 0 0 0 0 0 0
0.019928258 0 0 0 0 0 0 0 0 0 0 0
0 0 0 0 0 0 0 0 0 0 0 0 0
0 0 0 0 0 0 0 0 0 0 0 0.64516129
0
"Uroviricota;Crassvirales__na" 0.130378096 0.274473925 0.556999629 0.254452926 0.184558597
0.708991299 2.51572327 4.657534247 3.305785124 4.597701149 2 0.080558539
0 0.331674959 0 0 4.151404151 0 0.170648464 0.159235669 0
0 0.019928258 0 0 0.213675214 0.757575758 0 0 0 0
0.347624565 0.047236656 0 0 0 2.023608769 0.911854103 2.232142857
0 0.168634064 0.262123198 0.098425197 0.138696255 0 0 4.347826087
0 0 0 0 0 0 0 3.820439351 3.048780488 0 0
0 0
"Uroviricota;Crevaviridae" 0.260756193 0 0.482733012 0.212044105 0.030759766
0.290041895 0.419287212 0.136986301 1.652892562 0 0 0.026852846 0
0 0 0 0 0 0 0 0 0 0 0.095510984
0.106837607 0.757575758 0 0 0 0 0 0 0.219298246 0
0 13.32209106 6.534954407 9.151785714 0 0.337268128 0 0.098425197
0.346740638 0 0 15.2173913 0 0 0 0 0 0 0
0 0 0 0 0.64516129 0
"Uroviricota;Intestiviridae" 0.130378096 0 0.222799851 0.084817642 0 0
2.725366876 10.82191781 29.75206612 24.13793103 0.956521739 0 0.174216028
2.819237148 21.18811881 16 6.105006105 0 0.341296928 0 0 0
0.398565165 0 0 0.427350427 0 0 0.699300699 0 0 0
0 0 0 0 32.8836425 13.82978723 37.05357143 7.547169811
0.674536256 0.419397117 0.196850394 0 0 0 2.173913043 0 0
0 0 0 0 1.111111111 13.18051576 13.64329268 0 0.847457627
10.32258065 0
"Uroviricota;Steigviridae" 0 0 0.111399926 0.084817642 0.061519532 0.354495649 0
0 0 0 0 0 0 0.99009901 0 0.366300366 0
0 0 0 0 0 0 0.859598854 0.534188034 0 0
0.699300699 0 0 0 0.75578649 0.877192982 0 0.574712644 0
0 0 0 0.168634064 0 0 0 0 0 0 0 0
0 0 0 0 0 0 0 0 0 0 0
"Uroviricota;Suoliviridae" 4.302477184 3.568161025 22.53991831 23.07039864 18.60965857
43.08733484 0.209643606 0.273972603 7.438016529 4.597701149 0.956521739 0
0 0 0 0 0 0 0.170648464 0.159235669 1.666666667 0
0.318852132 0 1.146131805 3.739316239 12.12121212 2.380952381 2.797202797
0.315012849 0 0.115874855 0.850259802 0 0 0 7.504215852
3.951367781 10.71428571 1.886792453 3.541315346 0.262123198 0.295275591
0.416088766 0 0 4.347826087 0 0 0.198019802 0 0 0
0 1.432664756 0.914634146 4.166666667 0 10.32258065 0
"Viruses__na;Baculoviridae" 0 0 0 0 0 0 0 0 0 0
0 0 0 0 0 0 0 0 0 0 0 0 0
0 0 0 0 0 0 0 0 0 0 0 0 0
0 0 0 0 0 0 0 0 0 0 0 0 0
0 0 0 0 0 0 0 0 0 0.64516129 0
"Viruses__na;Nimaviridae" 0 0 0 0 0 0 0 0 0 0
0 0 0 0 0 0 0 0 0 0 0 0 0
0.384615385 0 0 0 0 0 0 0 0 0 0 0
0 0 0 0 0 0 0 0 0 0 0 0 0
0 0 0 0 0 0 0 0 0 0 0 0
"Viruses__na;Anelloviridae" 0 0 0 0 0 0 0 0 0 0
0.086956522 0 0 0 0 0 0.244200244 1 0 0
1.666666667 0 0.079713033 5.769230769 0 0.106837607 0 0 0
0.18237586 0 0 1.133679735 8.114035088 64.55306033 0 0 0
0 0 0.168634064 0.05242464 0 0 0 0 0 0 0

0 0 0.465116279 8.333333333 0 0 0 8.333333333 0 0
0
"Viruses__na;Cruciviridae" 0 0 0 0 0 0 0 0 0 0
0 0.026852846 0 0 0 0 0 0 0 0 0 0
0 0 0 0 0 0 0 0 0 0 0 0 0
0 0 0 0 0 0 0 0 0 0 0 0 0
0 0 0 0 0 0 0 0 0 0 0 0
"Viruses__na;Viruses__na" 11.73402868 5.855443733 8.540660973 8.396946565 4.15256844
5.543022881 6.708595388 2.876712329 4.132231405 8.045977011 0.52173913
0.187969925 1.916376307 3.316749585 4.554455446 1.333333333 3.296703297 3
1.535836177 2.388535032 0 0 0.338780391 1.153846154 3.629417383
6.08974359 40.90909091 39.28571429 48.95104895 83.48669485 14.88764045
8.922363847 59.70713274 58.99122807 0.044033465 8.045977011 0.590219224
0.455927052 0.669642857 1.886792453 31.70320405 11.79554391 9.350393701
9.500693481 0 0 0 0.244498778 2.338308458 1.782178218 0
0.465116279 0 0 5.82617001 5.716463415 0 7.627118644 0.64516129
0
