# Supplementary material for: Comparison of Experimental Methodologies Based on Bulk-Metagenome and Virus-like Particle Enrichment: Pros and Cons for Representativeness and Reproducibility in the Study of the Fecal Human Virome
Source: Microorganisms. 2024 Jan 13;12(1):162. doi: 10.3390/microorganisms12010162 (PMC10820677; doi:10.3390/microorganisms12010162)
Supplement: Supplementary file 1 [file Table S1.pdf › Table S1.pdf]

Table S1. Abundance matrix displaying viral counts across samples.

TAXONOMY	CN_1A	CN_1B	CN_2A	CN_2B	CN_3A	CN_3B	CN_PCR3A	CN_PCR3B	CN_RT2A	CN_RT2B	CN_RT3A	CN_RT3B	02.1A	02.1B	02.2A	02.2B	02.3A	02.3B	03.1A	03.1B	03.2A	03.2B	03.3A	03.3B	05.1A	05.1B	05.2A	05.2B	05.3A	05.3B	06.1A	06.1B	06.2A	06.2B	06.3A	06.3B	07.1A	07.1B	07.2A	07.2B	07.3A	07.3B	08.1A	08.1B	08.2A	08.2B	08.3A	08.3B	09.1A	09.1B	09.2A	09.2B	09.3A	09.3B	13.1A	13.1B	13.2A	13.2B	13.3A	13.3B	20.1A	20.1B	20.2A	20.2B	20.3A	20.3B	22.1A	22.1B	22.2A	22.2B	22.3A	22.3B
"Viruses;Artverviricota;Revtraviricetes;Ortervirales;Caulimoviridae;Badnavirus;Aglaonema_bacilliform_virus"	0	0	0	0	0	0	0	0	0	0	0	0	0	0	0	0	0	0	0	0	0	0	0	0	0	0	0	0	0	0	0	0	0	0	0	5	0	0	0	0	0	0	0	0	0	0	0	0	0	0	0	0	0	0	0	0	0	0	0	0	0	0	0	0	0	0	0	0	0	0	0	0
"Viruses;Artverviricota;Revtraviricetes;Ortervirales;Caulimoviridae;Badnavirus;Ambrosia_asymptomatic_virus_2_UKM-2007"	0	0	0	0	0	0	0	90	0	0	0	0	0	0	0	0	0	0	0	0	0	0	0	0	0	0	0	0	0	0	0	0	0	0	0	0	0	0	0	0	0	0	0	0	0	0	0	0	0	0	0	0	0	0	0	0	0	0	0	0	0	0	0	0	0	0	0	0	0	0	0	0
"Viruses;Artverviricota;Revtraviricetes;Ortervirales;Caulimoviridae;Badnavirus;Badnavirus__uc"	0	0	0	0	0	0	0	0	0	0	0	0	0	0	0	0	0	0	0	0	0	0	0	0	0	0	0	0	0	0	0	0	0	0	0	9	0	0	0	0	0	0	0	0	0	0	0	0	0	0	0	0	0	0	0	0	0	0	0	0	0	0	0	0	0	0	0	0	0	0	0	0
"Viruses;Artverviricota;Revtraviricetes;Ortervirales;Caulimoviridae;Badnavirus;Banana_streak_virus"	0	0	0	0	0	0	0	0	0	0	0	0	0	0	0	0	0	0	0	0	0	0	0	0	0	0	0	0	0	0	0	0	0	0	0	1	0	0	0	0	0	0	0	0	0	0	0	0	0	0	0	0	0	0	0	0	0	0	0	0	0	0	0	0	0	0	0	0	0	0	0	0
"Viruses;Artverviricota;Revtraviricetes;Ortervirales;Caulimoviridae;Badnavirus;Cacao_swollen_shoot_CE_virus"	0	0	0	0	0	0	0	0	0	0	0	0	0	0	0	0	0	0	0	0	0	0	0	0	0	0	0	0	0	0	0	0	0	0	0	3	0	0	0	0	0	0	0	0	0	0	0	0	0	0	0	0	0	0	0	0	0	0	0	0	0	0	0	0	0	0	0	0	0	0	0	0
"Viruses;Artverviricota;Revtraviricetes;Ortervirales;Caulimoviridae;Badnavirus;Grapevine_badna_FI_virus"	0	0	0	0	0	0	0	0	0	0	0	0	0	0	0	0	0	0	0	0	0	0	0	0	0	0	0	0	0	0	0	0	0	0	0	1	0	0	0	0	0	0	0	0	0	0	0	0	0	0	0	0																				

0 0 0 0 0 0 0 0 0 0 0 0 0
0 0 0 0 0 0 0
"Viruses;Artverviricota;Revtraviricetes;Ortervirales;Caulimoviridae;Badnavirus;Taro_bacilliform_CH_virus"
0 0 0 0 0 0 0 0 0 0 0 0 0
0 0 0 0 0 0 0 0 0 0 0 0 0
0 0 0 0 0 0 0 0 0 1 0 0 0
0 0 0 0 0 0 0 0 0 0 0 0 0
0 0 0 0 0 0 0 0 0 0 0 0 0
0 0 0 0 0 0 0
"Viruses;Artverviricota;Revtraviricetes;Ortervirales;Caulimoviridae;Caulimoviridae__na;Aristotelia_chilensis_vi
rus_1" 0 0 2 0 0 0 0 0 0 1 0 0 0
0 0 0 0 0 0 0 0 0 0 0 0 0
0 0 0 0 0 0 0 0 0 0 0 0 0
0 0 0 0 0 0 0 0 0 0 0 0 0
0 0 0 0 0 0 0 0 0 0 0 0 0
0 0 0 0 0 0 0
"Viruses;Artverviricota;Revtraviricetes;Ortervirales;Caulimoviridae;Petuvirus;Petunia_vein_clearing_virus"
0 0 0 0 0 0 0 0 0 1 0 0 0
0 0 0 0 0 0 0 0 0 0 0 0 0
0 0 0 0 0 0 0 0 0 0 0 0 0
0 0 0 0 0 0 0 0 0 0 0 0 0
0 0 0 0 0 0 0 0 0 0 0 0 0
0 0 0 0 0 0 0
"Viruses;Artverviricota;Revtraviricetes;Ortervirales;Caulimoviridae;Solendovirus;Tobacco_vein_clearing_virus"
0 0 0 0 0 0 0 0 0 0 7 0 0
0 0 0 0 0 0 0 0 0 0 0 0 0
0 0 0 0 0 0 0 0 0 0 0 0 0
0 0 1 0 0 0 0 0 0 0 0 0 0
0 0 0 0 0 0 0 0 0 0 0 0 0
0 0 0 0 0 0 0
"Viruses;Artverviricota;Revtraviricetes;Ortervirales;Caulimoviridae;Tungrovirus;Rice_tungro_bacilliform_virus"
0 0 0 0 0 0 0 0 0 0 0 0 0
0 0 0 0 0 0 0 0 0 0 0 0 0
0 0 0 0 0 0 0 0 0 2 0 0 0
0 0 0 0 0 0 0 0 0 0 0 0 0
0 0 0 0 0 0 0 0 0 0 0 0 0
0 0 0 0 0 0 0
"Viruses;Artverviricota;Revtraviricetes;Ortervirales;Retroviridae;Gammaretrovirus;Gammaretrovirus__uc"
0 0 0 1 0 0 0 0 0 0 0 0 0
0 0 0 0 0 0 0 0 0 0 0 0 0
0 0 0 0 0 0 0 0 0 0 0 0 0
0 0 0 0 0 0 0 0 0 0 0 0 0
0 0 0 0 0 0 0 0 0 0 0 0 0
0 0 0 0 0 0 0
"Viruses;Artverviricota;Revtraviricetes;Ortervirales;Retroviridae;Gammaretrovirus;Murine_leukemia-
related_retroviruses" 0 0 0 0 0 0 0 1 0 0 0
0 0 0 0 0 0 0 0 0 0 0 0 0
0 0 0 0 0 0 0 0 0 0 0 0 0
0 0 0 0 0 0 0 0 0 0 0 0 0

0 0 0 0 0 0 0 0 0 0 0 0 0
0 0 0 0 0 0 0 0 0
"Viruses;Artverviricota;Revtraviricetes;Ortervirales;Retroviridae;Gammaretrovirus;Porcine_type-C_oncovirus"
0 0 0 0 0 0 0 3 0 0 0 0 0
0 0 0 0 0 0 0 0 0 0 0 0 0
0 0 0 0 0 0 0 0 0 1 0 0 0
0 0 0 0 0 0 0 0 0 0 0 0 0
0 0 0 0 0 0 0 0 0 0 0 0 0
0 0 0 0 0 0 0
"Viruses;Artverviricota;Revtraviricetes;Ortervirales;Retroviridae;Retroviridae__na;Avian_endogenous_retroviru
s_EAV-HP" 0 0 0 0 0 0 0 0 0 0 0 0
0 0 0 0 0 0 0 0 0 0 0 0 0
0 0 0 0 0 0 0 0 0 0 0 0 0
0 0 0 0 0 0 0 0 5 0 0 0 0
0 0 0 0 0 0 0 0 0 0 0 0 0
0 0 0 0 0 0 0 0
"Viruses;Artverviricota;Revtraviricetes;Ortervirales;Retroviridae;Retroviridae__na;Citrus_blight-
associated_pararetrovirus" 0 0 2 0 0 0 0 0 0 0
0 0 0 0 0 0 0 0 0 0 0 0 0
0 0 0 0 0 0 0 0 0 0 0 0 0
0 0 0 0 0 0 0 0 0 0 15 0 0
0 0 0 0 0 0 0 0 0 0 0 0 0
0 0 0 0 0 0 0 0 0 0
"Viruses;Artverviricota;Revtraviricetes;Ortervirales;Retroviridae;Retroviridae__na;Citrus_endogenous_pararetr
ovirus" 0 0 0 0 0 0 0 0 0 0 0 0 0
0 0 0 0 0 0 0 0 0 0 0 0 0
0 0 0 0 0 0 0 0 0 0 0 0 0
0 0 0 0 0 0 0 28 0 0 0 0 0
0 0 0 0 0 0 0 0 0 0 0 0 0
0 0 0 0 0 0 0
"Viruses;Artverviricota;Revtraviricetes;Ortervirales;Retroviridae;Retroviridae__uc;Retroviridae__uc" 0
0 0 0 0 0 0 0 0 1 0 0 0 0
0 0 0 0 0 0 0 0 0 0 0 0 0
0 0 0 0 0 0 0 0 0 0 0 0 0
0 0 0 0 0 0 18 0 0 0 0 0 0
0 0 0 0 0 0 79 0 0 0 0 0 0
0 0 0 0 0 0
"Viruses;Cossaviricota;Papovaviricetes;Zurhausenvirales;Papillomaviridae;Betapapillomavirus;Betapapillomavi
rus_2" 0 0 0 0 0 0 0 0 0 1 0 0 0
0 0 0 0 0 0 0 0 0 0 0 0 0
0 0 0 0 0 0 0 0 0 0 0 0 0
0 0 112 0 0 0 0 0 0 0 0 0 0
0 0 0 0 0 0 0 0 0 0 0 0 0
0 0 0 0 0 0 0
"Viruses;Cossaviricota;Papovaviricetes;Zurhausenvirales;Papillomaviridae;Gammapapillomavirus;Gammapapil
lomavirus_sp." 0 0 0 0 0 0 0 0 0 0 0 0
0 0 0 0 0 0 0 0 0 0 0 0 0
0 0 0 0 0 0 0 0 0 0 4 0 0
0 0 0 0 0 0 0 0 0 0 0 0 0

0 0 0 0 0 0 0 0 0 0 0 0 0
0 0 0 0 0 0 0 0
"Viruses;Cossaviricota;Papovaviricetes;Zurhausenvirales;Papillomaviridae;Papillomaviridae__uc;Papillomaviri
dae__uc" 0 0 0 0 0 0 0 0 0 0 0 0
0 0 0 0 0 0 0 0 0 0 0 0 0
0 0 0 0 0 0 0 0 0 0 0 0 0
0 0 0 3 0 0 0 0 0 0 0 0 0
0 0 0 0 0 0 0 0 0 0 0 0 0
0 0 0 0 0 0 0 0
"Viruses;Cossaviricota;Quintoviricetes;Piccovirales;Parvoviridae;Ambidensovirus;Aegithalos_caudatus_ambide
nsovirus" 0 0 0 1 0 0 0 0 0 0 0 0
0 0 0 0 0 0 0 0 0 0 0 0 0
0 0 0 0 0 0 0 0 0 0 0 0 0
0 0 0 0 0 0 0 0 0 0 0 0 0
0 0 0 0 0 0 0 0 0 0 0 0 0
0 0 0 0 0 0 0 0
"Viruses;Cossaviricota;Quintoviricetes;Piccovirales;Parvoviridae;Ambidensovirus;Ambidensovirus__uc" 0
0 0 1 0 0 0 0 0 0 0 0 0 0
0 0 0 0 0 0 0 0 0 0 0 0 0
0 0 0 0 0 0 0 0 0 0 0 0 0
0 0 0 0 0 0 0 2 0 0 0 0 0
0 0 0 0 0 0 0 0 0 0 0 0 0
0 0 0 0 0 0
"Viruses;Cossaviricota;Quintoviricetes;Piccovirales;Parvoviridae;Ambidensovirus;Periparus_ater_ambidensovir
us" 0 0 1 2 0 0 0 0 0 0 0 0 0
0 0 0 0 0 0 0 0 0 0 0 0 0
0 0 0 0 0 0 0 0 0 0 0 0 0
0 0 0 0 0 0 0 0 0 0 0 0 0
0 0 0 0 0 0 0 0 0 0 0 0 0
0 0 0 0 0 0 0
"Viruses;Cossaviricota;Quintoviricetes;Piccovirales;Parvoviridae;Ambidensovirus;Phylloscopus_inornatus_ambi
densovirus" 0 0 0 0 1 1 0 0 0 0 0 0
0 0 0 0 0 0 0 0 0 0 0 0 0
0 0 0 0 0 0 0 0 0 0 0 0 0
0 0 0 0 0 0 0 0 0 1 0 0 0
0 0 0 0 0 0 0 0 0 0 0 0 0
0 0 0 0 0 0 0 0
"Viruses;Cossaviricota;Quintoviricetes;Piccovirales;Parvoviridae;Ambidensovirus;Phylloscopus_proregulus_am
bidensovirus" 0 0 0 0 0 0 0 0 0 0 0 0
0 0 0 0 0 0 0 0 0 0 0 0 0
0 0 0 0 0 0 0 0 0 0 0 0 0
0 0 0 0 0 0 0 0 0 1 0 0 0
0 0 0 0 0 0 0 0 0 0 0 0 0
0 0 0 0 0 0 0 0
"Viruses;Cossaviricota;Quintoviricetes;Piccovirales;Parvoviridae;Aveparvovirus;Psittacidae_aveparvovirus"
0 0 0 0 48 0 0 0 0 0 0 0 0
0 0 0 0 0 0 0 0 0 0 0 0 0
0 0 0 0 0 0 0 0 0 0 0 0 0
0 0 0 0 0 0 0 0 0 0 0 0 0

0 0 0 0 0 0 0 0 0 0 0 0 0
0 0 0 0 0 0 0
"Viruses;Cossaviricota;Quintoviricetes;Piccovirales;Parvoviridae;Aveparvovirus;Psittaciform_aveparvovirus"
0 0 25 74 138 478 0 0 0 0 0 0 0
0 0 0 3 0 0 0 0 0 1 4 0 0
0 0 0 0 0 0 0 0 111 4 0 0 0
2 2 7 0 0 1 0 0 23 0 0 0 0
1 0 0 0 0 0 0 0 0 0 0 0 0
0 0 0 0 0 9 0
"Viruses;Cossaviricota;Quintoviricetes;Piccovirales;Parvoviridae;Dependoparvovirus;Anseriform_dependoparv
ovirus_1" 0 0 2 4 84 12 0 0 0 0 0 0
0 0 0 0 3 3 0 0 0 0 0 1 0
0 0 0 0 0 0 0 0 0 0 0 0 0
0 0 0 123 0 0 0 0 0 6 0 0 0
0 0 0 0 0 0 0 0 0 0 0 0 0
0 0 0 0 0 0 4 0
"Viruses;Cossaviricota;Quintoviricetes;Piccovirales;Parvoviridae;Parvoviridae__na;Accipiter_gentilis_parvo-
like_hybrid_virus" 0 0 0 1 0 0 0 0 0 0 0
0 0 0 0 0 0 0 0 0 0 0 0 0
0 0 0 0 0 0 0 0 0 0 0 0 0
0 0 0 0 0 0 0 0 0 0 0 0 0
0 0 0 0 0 0 0 0 0 0 0 0 0
0 0 0 0 0 0 0 0 0
"Viruses;Cossaviricota;Quintoviricetes;Piccovirales;Parvoviridae;Parvoviridae__na;Ciconia_boyciana_parvo-
like_hybrid_virus" 0 0 0 0 0 1 0 0 0 0 0
0 0 0 0 0 0 0 0 0 0 0 0 0
0 0 0 0 0 0 0 0 0 0 0 0 0
0 0 0 0 0 0 0 0 0 0 0 0 0
0 0 0 0 0 0 0 0 0 0 0 0 0
0 0 0 0 0 0 0 0 0
"Viruses;Cossaviricota;Quintoviricetes;Piccovirales;Parvoviridae;Parvoviridae__na;Cygnus_columbianus_parvo
-like_hybrid_virus" 0 0 0 0 1 0 0 0 0 0 0
0 0 0 0 0 0 0 0 0 0 0 0 0
0 0 0 0 0 0 0 0 0 0 0 0 0
0 0 0 0 0 0 0 0 0 0 0 0 0
0 0 0 0 0 0 0 0 0 0 0 0 0
0 0 0 0 0 0 0 0 0
"Viruses;Cossaviricota;Quintoviricetes;Piccovirales;Parvoviridae;Parvoviridae__na;Densovirus_SC116" 0
0 0 1 0 0 0 0 0 0 0 0 0 0
0 0 0 0 0 0 0 0 0 0 0 0 0
0 0 0 0 0 0 0 0 0 0 0 0 0
0 0 0 0 0 0 0 0 0 0 0 0 0
0 0 0 0 0 0 0 0 0 0 0 0 0
0 0 0 0 0 0
"Viruses;Cossaviricota;Quintoviricetes;Piccovirales;Parvoviridae;Parvoviridae__na;Densovirus_SC444" 0
0 2 0 0 0 0 0 0 0 0 0 0 0
0 0 0 0 0 0 0 0 0 0 0 0 0
0 0 0 0 0 0 0 0 0 0 0 0 0
0 0 0 0 0 0 0 0 0 0 0 0 0

0 0 0 0 0 0 0 0 0 0 0 0 0
0 0 0 0 0 0
"Viruses;Cossaviricota;Quintoviricetes;Piccovirales;Parvoviridae;Parvoviridae__na;Emberiza_pusilla_parvo-like_hybrid_virus" 0 0 1 3 81 32 0 0 0 0 0
0 0 0 0 0 0 0 0 0 0 0 1 2
0 0 0 0 0 0 0 0 0 0 0 0 0
0 0 0 0 0 0 0 0 0 0 9 0 0
0 0 0 0 0 0 0 0 0 0 0 0 0
0 0 0 0 0 0 0 0 0
"Viruses;Cossaviricota;Quintoviricetes;Piccovirales;Parvoviridae;Parvoviridae__na;Fish-associated_parvo-like_hybrid_virus" 0 0 0 2 0 1 0 0 0 0 0
0 0 0 0 0 0 0 0 0 0 0 0 0
0 0 0 0 0 0 0 0 0 0 0 0 0
0 0 0 0 0 0 0 0 0 0 0 0 0
0 0 0 0 0 0 0 0 0 0 0 0 0
0 0 0 0 0 0 0 0 0
"Viruses;Cossaviricota;Quintoviricetes;Piccovirales;Parvoviridae;Parvoviridae__na;Flumine_parvovirus_13"
0 0 0 0 0 1 0 0 0 0 0 0 0
0 0 0 0 0 0 0 0 0 0 0 0 0
0 0 0 0 0 0 0 0 0 0 0 0 0
0 0 0 0 0 0 0 0 0 0 0 0 0
0 0 0 0 0 0 0 0 0 0 0 0 0
0 0 0 0 0 0 0
"Viruses;Cossaviricota;Quintoviricetes;Piccovirales;Parvoviridae;Parvoviridae__na;Flumine_parvovirus_15"
0 0 0 0 19 4 0 0 0 0 0 0 0
0 0 0 1 0 0 0 0 0 0 2 0 0
0 0 0 0 0 0 0 0 0 0 0 0 0
0 0 0 0 0 0 0 0 0 0 0 0 0
0 0 0 0 0 0 0 0 0 0 0 0 0
0 0 0 0 0 3 0
"Viruses;Cossaviricota;Quintoviricetes;Piccovirales;Parvoviridae;Parvoviridae__na;Flumine_parvovirus_6"
0 0 0 4 69 9 0 0 0 0 0 0 0
0 0 0 0 0 0 0 0 0 0 0 0 0
0 0 0 0 0 0 0 0 0 2 0 0 0
0 0 0 0 0 0 0 0 2 0 0 0 0
0 0 0 0 0 0 0 0 0 0 0 0 0
0 0 0 0 0 0 0
"Viruses;Cossaviricota;Quintoviricetes;Piccovirales;Parvoviridae;Parvoviridae__na;Grus_japonensis_parvo-like_hybrid_virus" 0 0 2 7 22 7 0 0 0 0 0
0 0 0 0 0 0 0 0 0 0 0 0 0
0 0 0 0 0 0 0 0 0 0 0 0 0
0 0 0 0 3 0 0 0 0 0 4 0 0
0 0 0 0 0 0 0 0 0 0 0 0 0
0 0 0 0 0 0 0 1 0
"Viruses;Cossaviricota;Quintoviricetes;Piccovirales;Parvoviridae;Parvoviridae__na;Grus_japonensis_parvoviridae_sp." 0 0 0 0 0 2 0 0 0 0 0 0 0
0 0 0 0 0 0 0 0 0 0 0 0 0
0 0 0 0 0 0 0 0 0 0 0 0 0
0 0 0 0 0 0 0 0 0 0 0 0 0

0 0 0 0 0 0 0 0 0 0 0 0 0
0 0 0 0 0 0 0
"Viruses;Cossaviricota;Quintoviricetes;Piccovirales;Parvoviridae;Parvoviridae__na;Lactuca_sativa_parvo-
like_virus" 0 0 0 0 1 0 0 0 0 0 0 0
0 0 0 0 0 0 0 0 0 0 0 0 0
0 0 0 0 0 0 0 0 0 0 0 0 0
0 0 0 1 0 0 0 0 0 0 0 0 0
0 0 0 0 0 0 0 0 0 0 0 0 0
0 0 0 0 0 0 0 0
"Viruses;Cossaviricota;Quintoviricetes;Piccovirales;Parvoviridae;Parvoviridae__na;Luscinia_sibilans_parvo-
like_hybrid_virus" 0 0 0 1 7 10 0 0 0 0 0
0 0 0 0 0 0 0 0 0 0 0 0 0
0 0 0 0 0 0 0 0 0 0 0 0 0
0 0 0 0 0 0 0 0 0 0 3 0 0
0 0 0 0 0 0 0 0 0 0 0 0 0
0 0 0 0 0 0 0 2 0
"Viruses;Cossaviricota;Quintoviricetes;Piccovirales;Parvoviridae;Parvoviridae__na;Motacilla_cinerea_parvo-
like_hybrid_virus" 0 0 0 1 1 0 0 0 0 0 0
0 0 0 0 0 0 0 0 0 0 0 0 0
0 0 0 0 0 0 0 0 0 0 0 0 0
0 0 0 0 0 0 0 0 0 0 1 0 0
0 0 0 0 0 0 0 0 0 0 0 0 0
0 0 0 0 0 0 0 0 0
"Viruses;Cossaviricota;Quintoviricetes;Piccovirales;Parvoviridae;Parvoviridae__na;Otus_scops_parvoviridae_sp
." 0 0 0 0 10 0 0 0 0 0 0 0 0
0 0 0 0 0 0 0 0 0 0 0 0 0
0 0 0 0 0 0 0 0 0 0 0 0 0
0 0 0 0 0 0 0 0 0 0 0 0 0
0 0 0 0 0 0 0 0 0 0 0 0 0
0 0 0 0 0 0 0
"Viruses;Cossaviricota;Quintoviricetes;Piccovirales;Parvoviridae;Parvoviridae__na;Parvoviridae_sp." 0
0 2 14 117 40 0 0 0 0 0 0 0 0
0 0 1 2 0 0 0 0 1 3 0 0 0
0 0 0 0 0 0 0 2 5 0 0 0 0
0 0 0 0 1 0 0 32 0 0 0 0 1
73 0 0 0 0 0 0 0 0 0 0 0 0
0 0 0 0 3 0
"Viruses;Cossaviricota;Quintoviricetes;Piccovirales;Parvoviridae;Parvoviridae__na;Parvovirinae_sp." 0
0 0 0 14 7 0 0 0 0 0 0 0 0
0 0 0 0 0 0 0 0 0 1 0 0 0
0 0 0 0 0 0 0 0 0 0 0 0 0
0 0 0 0 0 0 0 1 0 0 0 0 0
0 0 0 0 0 0 0 0 0 0 0 0 0
0 0 0 0 0 0
"Viruses;Cossaviricota;Quintoviricetes;Piccovirales;Parvoviridae;Parvoviridae__na;Parvovirus_NIH-CQV"
0 0 2 6 104 53 0 0 0 0 0 0 0
0 0 0 1 7 0 0 0 0 1 4 0 0
0 0 0 0 0 0 0 0 0 0 0 0 0
0 0 8 0 0 0 0 0 14 0 0 0 0

0 0 0 0 0 0 0 0 0 0 0 0 0
0 0 0 0 0 1 0
"Viruses;Cossaviricota;Quintoviricetes;Piccovirales;Parvoviridae;Parvoviridae__na;Pavo_cristatus_parvo-like_hybrid_virus" 0 0 0 0 5 0 0 0 0 0 0
0 0 0 0 0 0 0 0 0 0 0 0 0
0 0 0 0 0 0 0 0 0 0 0 0 0
0 0 0 0 0 0 0 0 0 0 0 0 0
0 0 0 0 0 0 0 0 0 0 0 0 0
0 0 0 0 0 0 0 0 0
"Viruses;Cossaviricota;Quintoviricetes;Piccovirales;Parvoviridae;Parvoviridae__na;Phoenicopteridae_parvo-like_hybrid_virus" 0 0 21 57 180 129 0 0 0 0 0
0 0 0 0 0 4 2 0 0 2 0 3 1
0 0 0 0 0 0 0 0 0 0 1 0 0
0 0 0 0 6 0 0 0 0 0 7 0 0
0 2 0 1 0 0 0 0 0 0 0 0 0
0 0 0 0 0 0 0 17 0
"Viruses;Cossaviricota;Quintoviricetes;Piccovirales;Parvoviridae;Parvoviridae__na;Phoenicopterus_roseus_parvo-like_hybrid_virus" 0 0 0 3 96 16 0 0 0 0 0
0 0 0 0 0 1 0 0 0 0 0 0 1
0 0 0 0 0 0 0 0 0 0 0 0 0
0 0 0 0 0 0 0 0 0 0 11 0 0
0 0 0 0 0 0 0 0 0 0 0 0 0
0 0 0 0 0 0 0 0 0
"Viruses;Cossaviricota;Quintoviricetes;Piccovirales;Parvoviridae;Parvoviridae__na;Phylloscopus_proregulus_parvoviridae_sp." 0 0 0 0 2 0 0 0 0 0 0 0
0 0 0 0 0 0 0 0 0 0 0 0 0
0 0 0 0 0 0 0 0 0 0 0 0 0
0 0 0 0 0 0 0 0 0 0 0 0 0
0 0 0 0 0 0 0 0 0 0 0 0 0
0 0 0 0 0 0 0 0
"Viruses;Cossaviricota;Quintoviricetes;Piccovirales;Parvoviridae;Parvoviridae__na;Psittacidae_parvo-like_hybrid_virus" 0 0 1 1 77 4 0 0 0 0 0
0 0 0 0 0 1 0 0 0 0 0 0 2
0 0 0 0 0 0 0 0 0 0 0 0 0
0 0 0 0 0 0 0 0 0 0 3 0 0
0 0 0 0 0 0 0 0 0 0 0 0 0
0 0 0 0 0 0 0 0 0
"Viruses;Cossaviricota;Quintoviricetes;Piccovirales;Parvoviridae;Parvoviridae__na;Psittacidae_parvoviridae_sp." 0 0 0 0 3 0 0 0 0 0 0 0 0
0 0 0 0 0 0 0 0 0 0 0 0 0
0 0 0 0 0 0 0 0 0 0 0 0 0
0 0 0 0 0 0 0 0 1 0 0 0 0
0 0 0 0 0 0 0 0 0 0 0 0 0
0 0 0 0 0 0 0
"Viruses;Cossaviricota;Quintoviricetes;Piccovirales;Parvoviridae;Parvoviridae__na;Psittaciform_parvoviridae_sp." 3 0 38 115 288 538 0 0 0 0 0 0 0
0 0 0 13 8 0 0 0 0 2 6 0 0
0 0 0 0 0 0 0 0 3 2 0 0 0
1 0 22 0 0 3 0 0 141 0 0 0 0

3	0	0	0	0	0	0	0	0	0	1	0	0
0	0	0	0	0	21	0
"Viruses;Cossaviricota;Quintoviricetes;Piccovirales;Parvoviridae;Parvoviridae__na;Trichosanthes_kirilowii_parvo-like_virus"	0	0	0	0	2	12	0	0	0	0	0	0
0	0	0	0	0	0	0	0	0	0	0	0	0
0	0	0	0	0	0	0	0	0	0	0	0	0
0	0	0	0	0	0	0	0	0	1	0	0	0
0	0	0	0	0	0	0	0	0	0	0	0	0
0	0	0	0	0	0	0	0
"Viruses;Cossaviricota;Quintoviricetes;Piccovirales;Parvoviridae;Parvoviridae__uc;Parvoviridae__uc"	0
0	25	55	259	271	0	0	0	0	0	0	0	0
0	0	3	0	0	0	0	0	1	1	0	0	0
0	0	0	0	0	0	0	2	0	0	0	0	0
0	19	0	0	0	0	0	24	0	0	0	0	1
1	0	0	0	0	0	0	0	0	0	0	0	0
0	0	0	0	1	0
"Viruses;Cossaviricota;Quintoviricetes;Piccovirales;Parvoviridae;Protoambidensovirus;Dipteran_protoambidensovirus_1"	0	0	4	0	0	0	0	0	0	0	0	0
0	0	0	0	0	0	0	0	0	0	0	0	0
0	0	0	0	0	0	0	0	0	0	0	0	0
0	0	0	0	0	0	0	0	0	4	0	0	0
0	0	0	0	0	0	0	0	0	0	0	0	0
0	0	0	0	0	0	0	0
"Viruses;Cossaviricota;Quintoviricetes;Piccovirales;Parvoviridae;Protoparvovirus;Carnivore_protoparvovirus_1"	0	0	0	1	7	3	0	0	0	0	0	0	0
0	0	0	0	0	0	0	0	0	0	0	0	0
0	0	0	0	0	0	0	0	0	0	0	0	0
0	0	0	0	0	0	0	0	0	0	0	0	0
0	0	0	0	0	0	0	0	0	0	0	0	0
0	0	0	0	0	0	0
"Viruses;Cressdnaviricota;Arfiviricetes;Cirlivirales;Circoviridae;Circoviridae__na;Avon-Heathcote_Estuary_associated_circular_virus_3"0	0	0	0	2	0	0	0
0	0	0	0	0	0	0	0	0	0	0	0	0
0	0	0	0	0	0	0	0	0	0	0	0	0
0	0	0	0	0	0	0	0	0	0	0	0	0
0	0	0	0	0	0	0	0	0	0	0	0	0
0	0	0	0	0	0	0	0	0	0	0	0
"Viruses;Cressdnaviricota;Arfiviricetes;Cirlivirales;Circoviridae;Circoviridae__na;Circoviridae_LDMD-2013b"
0	0	0	0	2	2	0	0	0	0	0	0	0
0	0	0	0	0	0	0	0	0	0	0	0	0
0	0	0	0	0	0	0	0	0	0	0	0	0
0	0	0	0	0	0	0	0	0	0	0	0	0
0	0	0	0	0	0	0	0	0	0	0	0	0
0	0	0	0	0	0	0
"Viruses;Cressdnaviricota;Arfiviricetes;Cirlivirales;Circoviridae;Circoviridae__na;Circoviridae_sp."	0
0	1	3	2	28	0	0	0	0	0	0	0	0
0	1	0	3	0	0	0	0	0	0	0	0	0
0	0	0	0	0	0	0	0	0	0	0	0	0
0	0	0	0	0	0	0	1	0	0	0	0	0

0 0 0 0 0 0 0 0 0 0 0 0 0
0 0 0 0 0 0
"Viruses;Cressdnaviricota;Arfiviricetes;Cirlivirales;Circoviridae;Circoviridae__na;Circovirus-like_genome_DCCV-13"0 0 3 3 69 29 0 0 0 0 0
0 0 0 0 0 0 0 0 0 0 0 1 1
0 0 0 0 0 1 0 0 0 0 0 0 0
0 0 0 0 0 0 0 0 0 0 16 0 0
0 0 0 0 0 0 0 0 0 0 0 0 0
0 0 0 0 0 0 0 0 0
"Viruses;Cressdnaviricota;Arfiviricetes;Cirlivirales;Circoviridae;Circoviridae__na;Circovirus-like_genome_DCCV-4" 0 0 0 7 1 0 0 0 0 0 0
0 0 0 0 0 0 0 0 0 0 0 0 0
0 0 0 0 0 0 0 0 0 0 0 0 0
0 0 0 0 0 0 0 0 0 0 3 0 0
0 0 0 0 0 0 0 0 0 0 0 0 0
0 0 0 0 0 0 0 0 0
"Viruses;Cressdnaviricota;Arfiviricetes;Cirlivirales;Circoviridae;Circoviridae__na;Circovirus-like_genome_DCCV-7" 0 0 0 0 11 0 0 0 0 0 0
0 0 0 0 0 0 0 0 0 0 0 0 0
0 0 0 0 0 0 0 0 0 0 0 0 0
0 0 0 0 0 0 0 0 0 0 0 0 0
0 0 0 0 0 0 0 0 0 0 0 0 0
0 0 0 0 0 0 0 0 0
"Viruses;Cressdnaviricota;Arfiviricetes;Cirlivirales;Circoviridae;Circoviridae__na;Circovirus-like_genome_DHCV-2" 0 0 0 0 0 0 0 0 0 0 0
0 0 0 0 0 0 0 0 0 0 0 0 0
0 0 0 0 0 0 0 0 0 0 0 0 0
0 0 0 0 0 0 0 0 0 0 1 0 0
0 0 0 0 0 0 0 0 0 0 0 0 0
0 0 0 0 0 0 0 0 0
"Viruses;Cressdnaviricota;Arfiviricetes;Cirlivirales;Circoviridae;Circoviridae__na;Tick-associated_circular_virus-2" 0 0 0 3 0 1 0 0 0 0
0 0 0 0 0 0 0 0 0 0 0 0 0
0 0 0 0 0 0 0 0 0 0 0 0 0
0 0 0 0 0 0 0 0 0 0 0 0 0
0 0 0 0 0 0 0 0 0 0 0 0 0
0 0 0 0 0 0 0 0 0 0
"Viruses;Cressdnaviricota;Arfiviricetes;Cirlivirales;Circoviridae;Circoviridae__na;Wastewater_CRESS_DNA_virus_3" 0 0 0 0 0 8 0 0 0 0 0 0 0
0 0 0 0 0 0 0 0 0 0 0 0 0
0 0 0 0 0 0 0 0 0 2 0 0 0
0 0 0 0 0 0 0 0 2 0 0 0 0
0 0 0 0 0 0 0 0 0 0 0 0 0
0 0 0 0 0 4 0
"Viruses;Cressdnaviricota;Arfiviricetes;Cirlivirales;Circoviridae;Circoviridae__uc;Circoviridae__uc" 0
0 4 17 155 102 0 0 0 0 0 0 0 0
0 0 1 0 0 0 0 0 3 2 0 0 0
0 0 0 0 0 0 0 0 0 0 0 0 0
0 8 0 0 0 1 0 51 0 0 0 1 0

0 0 0 0 0 0 21 0 0 0 0 0 0
0 0 0 0 5 0
"Viruses;Cressdnaviricota;Arfiviricetes;Cirlivirales;Circoviridae;Circovirus;Bat_circovirus" 0 0
0 0 5 0 0 0 0 0 0 0 0 0 0
0 0 0 0 0 0 0 0 0 0 0 0 0
0 0 0 0 0 0 0 0 0 0 0 0 0
0 0 0 0 0 0 0 0 0 0 0 0 0
0 0 0 0 0 0 0 0 0 0 0 0 0
0 0 0 0 0
"Viruses;Cressdnaviricota;Arfiviricetes;Cirlivirales;Circoviridae;Circovirus;Beak_and_feather_disease_virus"
0 0 0 4 0 5 0 0 0 0 0 0 0
0 0 0 0 0 0 0 0 0 0 0 0 0
0 0 0 0 0 0 0 0 0 0 0 0 0
0 0 0 0 0 0 0 0 0 0 0 0 0
0 0 0 0 0 0 0 0 0 0 0 0 0
0 0 0 0 0 0 0
"Viruses;Cressdnaviricota;Arfiviricetes;Cirlivirales;Circoviridae;Circovirus;Circovirus_sp." 0 0
234 844 2030 1486 0 0 0 0 0 0 0 0 1
2 33 10 0 0 5 0 19 40 0 0 5 0
0 3 0 0 0 0 44 10 0 0 3 0 2
888 0 0 19 5 0 301 0 0 1 9 8 1
0 0 0 0 0 0 0 0 0 0 0 3 0
0 0 0 38 0
"Viruses;Cressdnaviricota;Arfiviricetes;Cirlivirales;Circoviridae;Circovirus;Giant_panda_circovirus_1" 0
0 0 1 6 6 0 0 0 0 0 0 0 0
0 0 1 0 0 0 0 0 0 0 0 0 0
0 0 0 0 0 0 0 0 0 0 0 0 0
0 0 0 0 0 0 0 0 0 0 0 0 0
0 0 0 0 0 0 0 0 0 0 0 0 0
0 0 0 0 0 0
"Viruses;Cressdnaviricota;Arfiviricetes;Cirlivirales;Circoviridae;Circovirus;Sichuan_tick-
associated_circovirus_4" 0 0 0 0 0 1 0 0 0 0
0 0 0 0 0 0 0 0 0 0 0 0 0
0 0 0 0 0 0 0 0 0 0 0 0 0
0 0 0 0 0 0 0 0 0 0 0 0 0
0 0 0 0 0 0 0 0 0 0 0 0 0
0 0 0 0 0 0 0 0 0 0
"Viruses;Cressdnaviricota;Arfiviricetes;Cirlivirales;Circoviridae;Circovirus;Sichuan_tick-
associated_circovirus_5" 0 0 0 1 0 0 0 0 0 0
0 0 0 0 0 0 0 0 0 0 0 0 0
0 0 0 0 0 0 0 0 0 0 0 0 0
0 0 0 0 0 0 0 0 0 0 0 0 0
0 0 0 0 0 0 0 0 0 0 0 0 0
0 0 0 0 0 0 0 0 0 0
"Viruses;Cressdnaviricota;Arfiviricetes;Cirlivirales;Vilyaviridae;Glamdringvirus;Rodent_stool-
associated_circular_genome_virus" 0 0 3 4 1 29 0 0 0
0 0 0 0 0 0 0 0 0 0 0 0 0
0 0 0 0 0 0 0 0 0 0 0 0 0
0 0 0 0 0 0 0 0 0 0 0 0 1

0 0 0 0 0 0 0 0 0 0 0 0 0
0 0 0 0 0 0 0 0 0 0 0
"Viruses;Cressdnaviricota;Cressdnaviricota__na;Cressdnaviricota__na;Cressdnaviricota__na;Cressdnaviricota__
na;Arizlama_virus" 0 0 9 26 15 126 0 0 0 0 0
0 0 0 0 0 1 0 0 0 0 0 0 8
0 0 0 0 0 0 0 0 0 0 0 0 0
0 0 0 0 0 0 0 2 0 0 71 0 0
0 0 0 0 0 0 0 0 0 0 0 0 0
0 0 0 0 0 0 0 2 0
"Viruses;Cressdnaviricota;Cressdnaviricota__na;Cressdnaviricota__na;Cressdnaviricota__na;Cressdnaviricota__
na;Chicken_proventriculitis-associated_circular_virus_4" 0 0 0 0 18 0
0 0 0 0 0 0 0 0 0 0 0 0 0
0 0 0 0 0 0 0 0 0 0 0 0 0
0 0 0 0 0 0 0 0 0 0 0 0 0
0 0 0 0 0 0 0 0 0 0 0 0 0
0 0 0 0 0 0 0 0 0 0 0 0 0
0
"Viruses;Cressdnaviricota;Cressdnaviricota__na;Cressdnaviricota__na;Cressdnaviricota__na;Cressdnaviricota__
na;CRESS_virus_SC_1_H1_2017" 0 0 0 0 0 0 0 0 0
0 0 0 0 0 0 0 0 0 0 0 0 0
0 0 0 0 0 0 0 0 0 0 0 0 0
0 0 0 0 0 0 0 0 0 0 0 0 0
1 0 0 0 0 0 0 0 0 0 0 0 0
0 0 0 0 0 0 0 0 0 0 0
"Viruses;Cressdnaviricota;Cressdnaviricota__na;Cressdnaviricota__na;Cressdnaviricota__na;Cressdnaviricota__
na;CRESS_virus_sp." 0 0 20 44 193 79 0 0 0 0 0
0 0 0 1 0 4 0 0 0 0 0 4 0
0 0 0 0 0 0 0 0 0 0 4 0 0
0 0 0 1 7 0 0 1 0 0 38 0 0
0 0 0 14 0 1 0 0 0 0 0 0 0
0 0 1 0 0 0 0 8 0
"Viruses;Cressdnaviricota;Cressdnaviricota__na;Cressdnaviricota__na;Cressdnaviricota__na;Cressdnaviricota__
na;CRESS_virus_sp._cta0f7" 0 0 0 0 3 0 0 0 0 0
0 0 0 0 0 0 0 0 0 0 0 0 0
0 0 0 0 0 0 0 0 0 0 0 0 0
0 0 0 0 0 0 0 0 0 0 0 0 0
0 0 0 0 0 0 0 0 0 0 0 0 0
0 0 0 0 0 0 0 0 0 0
"Viruses;Cressdnaviricota;Cressdnaviricota__na;Cressdnaviricota__na;Cressdnaviricota__na;Cressdnaviricota__
na;CRESS_virus_sp._ctBnw2" 0 0 0 0 0 0 0 0 0 0
0 0 0 0 0 0 0 0 0 0 0 0 0
0 0 0 0 0 0 0 0 0 0 0 0 0
0 5 0 0 0 0 0 0 0 0 0 0 0
0 0 0 0 0 0 0 0 0 0 0 0 0
0 0 0 0 0 0 0 0 0 0
"Viruses;Cressdnaviricota;Cressdnaviricota__na;Cressdnaviricota__na;Cressdnaviricota__na;Cressdnaviricota__
na;Cressdnaviricota_sp." 0 0 0 4 80 10 0 0 0 0
0 0 0 0 0 0 0 0 0 0 0 0 0
2 0 0 0 0 0 0 0 0 0 0 0 0
0 0 0 0 0 0 0 0 0 0 0 8 0

0 0 0 0 0 0 0 0 0 0 0 0 0
0 0 0 0 0 0 0 0 0 0
"Viruses;Cressdnaviricota;Cressdnaviricota__na;Cressdnaviricota__na;Cressdnaviricota__na;Cressdnaviricota__
na;Cygnus_columbianus_CRESS-DNA-virus_sp." 0 0 0 1 0 0 0
0 0 0 0 0 0 0 0 0 0 0 0 0
0 0 0 0 0 0 0 0 0 0 0 0 0
0 0 0 0 0 0 0 0 0 0 0 0 0
0 0 0 0 0 0 0 0 0 0 0 0 0
0 0 0 0 0 0 0 0 0 0 0 0 0
"Viruses;Cressdnaviricota;Cressdnaviricota__na;Cressdnaviricota__na;Cressdnaviricota__na;Cressdnaviricota__
na;Grus_japonensis_CRESS-DNA-virus_sp." 0 0 0 1 0 0 0 0
0 0 0 0 0 0 0 0 0 0 0 0 1
0 0 0 0 0 0 0 0 0 0 0 0 0
0 0 0 0 0 0 0 0 0 0 0 0 0
10 0 0 0 0 0 0 0 0 0 0 0 0
0 0 0 0 0 0 0 0 0 0 0 0
"Viruses;Cressdnaviricota;Cressdnaviricota__na;Cressdnaviricota__na;Cressdnaviricota__na;Cressdnaviricota__
na;Grus_monacha_CRESS-DNA-virus_sp." 0 0 0 0 0 1 0 0
0 0 0 0 0 0 0 0 0 0 0 0 0
0 0 0 0 0 0 0 0 0 0 0 0 0
0 0 0 0 0 0 0 0 0 0 0 0 0
0 0 0 0 0 0 0 0 0 0 0 0 0
0 0 0 0 0 0 0 0 0 0 0 0
"Viruses;Cressdnaviricota;Cressdnaviricota__na;Cressdnaviricota__na;Cressdnaviricota__na;Cressdnaviricota__
na;Luscinia_sibilans_CRESS-DNA-virus_sp." 0 0 0 1 1 0 0 0
0 0 0 0 0 0 0 0 0 0 0 0 0
0 0 0 0 0 0 0 0 0 0 0 0 0
0 0 0 0 0 0 0 0 0 0 0 0 0
0 0 0 0 0 0 0 0 0 0 0 0 0
0 0 0 0 0 0 0 0 0 0 0 0
"Viruses;Cressdnaviricota;Cressdnaviricota__na;Cressdnaviricota__na;Cressdnaviricota__na;Cressdnaviricota__
na;Molossus_molossus_associated_CRESS_DNA_virus" 0 0 0 1 0 1 0
0 0 0 0 0 0 0 0 0 0 0 0 0
0 0 0 0 0 0 0 0 0 0 0 0 0
0 0 0 0 0 0 0 0 0 0 0 0 0
0 0 0 0 0 0 0 0 0 0 0 0 0
0 0 0 0 0 0 0 0 0 0 0 0 0
"Viruses;Cressdnaviricota;Cressdnaviricota__na;Cressdnaviricota__na;Cressdnaviricota__na;Cressdnaviricota__
na;Motacilla_cinerea_CRESS-DNA-virus_sp." 0 0 0 0 0 3 0 0
0 0 0 0 0 0 0 0 0 0 0 0 0
0 0 0 0 0 0 0 0 0 0 0 0 0
0 0 0 0 0 0 0 0 0 0 0 0 0
5 0 0 0 0 0 0 0 0 0 0 0 0
0 0 0 0 0 0 0 0 0 0 0 0
"Viruses;Cressdnaviricota;Cressdnaviricota__na;Cressdnaviricota__na;Cressdnaviricota__na;Cressdnaviricota__
na;Mute_swan_feces_associated_circular_virus_2" 0 0 0 0 0 1 0
0 0 0 0 0 0 0 0 0 0 0 0 0
0 0 0 0 0 0 0 0 0 0 0 0 0
0 0 0 0 0 0 0 0 0 0 0 0 0

0 0 0 0 0 0 0 0 0 0 0 0 0
0 0 0 0 0 0 0 0 0 0 0 0 0
"Viruses;Cressdnaviricota;Cressdnaviricota__na;Cressdnaviricota__na;Cressdnaviricota__na;Cressdnaviricota__na;Tragopan_caboti_CRESS-DNA-virus_sp." 0 0 1 0 0 0 0 0
0 0 0 0 0 0 0 0 0 0 0 0 0
0 0 0 0 0 0 0 0 0 0 0 0 0
0 0 0 0 0 0 0 0 0 0 0 0 0
0 0 0 0 0 0 0 0 0 0 0 0 0
0 0 0 0 0 0 0 0 0 0 0 0
"Viruses;Cressdnaviricota;Cressdnaviricota__uc;Cressdnaviricota__uc;Cressdnaviricota__uc;Cressdnaviricota__uc;Cressdnaviricota__uc" 0 0 2 4 3 1 0 0 0 0
0 0 0 0 0 0 0 0 0 0 0 0 0
0 0 0 0 0 0 0 0 0 0 0 0 0
0 0 0 0 0 0 0 0 0 0 0 6 0
0 0 0 0 0 0 0 0 0 0 0 0 0
0 0 0 0 0 0 0 0 0 0
"Viruses;Cressdnaviricota;Repensiviricetes;Geplafuvirales;Geminiviridae;Begomovirus;Okra_enation_leaf_curl_virus" 0 0 0 0 0 0 0 0 0 2 0 0 0
0 0 0 0 0 0 0 0 0 0 0 0 0
0 0 0 0 0 0 0 0 0 0 0 0 0
0 0 0 0 0 0 0 0 0 0 0 0 0
0 0 0 0 0 0 0 0 0 0 0 0 0
0 0 0 0 0 0 0
"Viruses;Cressdnaviricota;Repensiviricetes;Geplafuvirales;Genomoviridae;Gemycircularvirus;Gemycircularvirus_derva1" 0 0 0 0 3 0 0 0 0 0 0 0
0 0 0 0 0 0 0 0 0 0 0 0 0
0 0 0 0 0 0 0 0 0 0 0 0 0
0 0 0 0 0 0 0 0 0 1 0 0 0
0 0 0 0 0 0 0 0 0 0 0 0 0
0 0 0 0 0 0 0 0
"Viruses;Cressdnaviricota;Repensiviricetes;Geplafuvirales;Genomoviridae;Gemycircularvirus;Gemycircularvirus_ptero2" 0 0 0 0 0 0 0 0 0 0 0 0
0 0 0 0 0 0 0 0 0 0 1 0 0
0 0 0 0 0 0 0 0 0 0 0 0 0
0 0 0 0 0 0 0 0 0 0 0 0 0
0 0 0 0 0 0 0 0 0 0 0 0 0
0 0 0 0 0 0 0 0
"Viruses;Cressdnaviricota;Repensiviricetes;Geplafuvirales;Genomoviridae;Gemycircularvirus;Giant_panda_feces-associated_gemycircularvirus"0 0 0 0 0 0 0 0 0 0
0 0 0 0 0 0 0 0 0 0 0 0 0
0 0 0 0 0 0 0 0 0 0 0 0 0
0 0 0 0 0 1 0 0 0 0 0 0 0
0 0 0 0 0 0 0 0 0 0 0 0 0
0 0 0 0 0 0 0 0 0 0
"Viruses;Cressdnaviricota;Repensiviricetes;Geplafuvirales;Genomoviridae;Gemycircularvirus;Murine_feces-associated_gemycircularvirus_1" 0 0 0 0 0 0 0 0 0
0 0 0 0 0 0 0 0 0 0 0 0 0
0 0 0 0 0 0 0 0 0 0 0 0 0
0 0 0 0 0 0 0 0 0 0 0 0 0

0 0 0 0 0 0 0 0 0 0 0 0 0
0 0 1 0 0 0 0 0 0 0 0
"Viruses;Cressdnaviricota;Repensiviricetes;Geplafuvirales;Genomoviridae;Genomoviridae__uc;Genomoviridae_
_uc" 0 0 0 0 0 0 0 0 0 0 0 0 0
0 0 0 0 0 0 0 0 0 0 0 0 0
0 0 0 0 0 0 0 0 0 0 0 0 0
0 0 0 0 0 0 0 0 0 0 0 0 0
0 0 0 0 0 0 0 0 0 0 0 1 0
0 0 0 0 0 0 0
"Viruses;Duplornaviricota;Chrymotiviricetes;Ghabrivirales;Chrysoviridae;Alphachrysovirus;Alphachrysovirus_
perseae" 0 0 0 0 0 0 0 0 0 0 0 0
0 0 0 0 0 0 0 0 0 0 0 0 0
0 0 0 0 0 0 0 0 0 0 0 0 0
0 0 0 0 0 0 0 0 0 0 0 0 0
0 0 0 0 0 0 0 0 0 0 0 0 0
0 0 0 0 0 1 0 0
"Viruses;Duplornaviricota;Chrymotiviricetes;Ghabrivirales;Chrysoviridae;Chrysovirus;Diplodia_seriata_chryso
virus_1" 0 0 1 0 0 0 0 0 0 0 0 0
0 0 0 0 0 0 0 0 0 0 0 0 0
0 0 0 0 0 0 0 0 0 0 0 0 0
0 0 0 0 0 0 0 0 0 0 0 0 0
0 0 0 0 0 0 0 0 0 0 0 0 0
0 0 0 0 0 0 0 0
"Viruses;Duplornaviricota;Chrymotiviricetes;Ghabrivirales;Totiviridae;Totiviridae__na;Malassezia_restricta_vir
us_MrV40L" 0 0 1 0 0 0 0 0 0 0 0 0
0 0 0 0 0 0 0 0 0 0 0 0 0
0 0 0 0 0 0 0 0 0 0 0 0 0
0 0 0 0 0 0 0 0 0 0 0 0 0
0 0 0 0 0 0 0 0 0 0 0 0 0
0 0 0 0 0 0 0 0
"Viruses;Duplornaviricota;Chrymotiviricetes;Ghabrivirales;Totiviridae;Totiviridae__na;Malassezia_restricta_vir
us_MrV40S" 0 0 0 2 0 0 0 0 0 0 0 0
0 0 0 0 0 0 0 0 0 0 0 0 0
0 0 0 0 0 0 0 0 0 0 0 0 0
0 0 0 0 0 0 0 0 0 0 0 0 0
0 0 0 0 0 0 0 0 0 0 0 0 0
0 0 0 0 0 0 0 0
"Viruses;Duplornaviricota;Chrymotiviricetes;Ghabrivirales;Totiviridae;Totiviridae__uc;Totiviridae__uc" 0
0 0 2 0 0 0 0 0 0 0 0 0 0
0 0 0 0 0 0 0 0 0 0 0 0 0
0 0 0 0 0 0 0 0 0 0 0 0 0
0 0 0 0 0 0 0 0 0 0 0 0 0
0 0 0 0 0 0 0 0 0 0 0 0 0
0 0 0 0 0 0
"Viruses;Duplornaviricota;Chrymotiviricetes;Ghabrivirales;Totiviridae;Totivirus;Totivirus_sp." 0 0
2 1 2 0 0 0 0 0 0 0 0 0 0
0 0 0 0 0 0 0 0 0 0 0 0 0
0 0 0 0 0 0 0 0 0 0 0 0 0
0 0 0 0 0 0 0 0 0 0 0 0 0

0 0 0 0 0 0 0 0 0 0 0 0 0
0 0 0 0 0
"Viruses;Hofneiviricota;Faserviricetes;Tubulavirales;Inoviridae;Inoviridae__na;Inoviridae_sp." 0 4
1 1 82 0 0 0 1 2 0 0 69 102 6
4 7 19 73 153 5 2 8 0 24 45 5 0
2 0 147 168 2 3 17 7 262 239 13 10 3
65 33 36 6 0 11 0 108 110 3 1 14 9
248 307 4 1 0 0 115 136 14 33 3 3 102
252 1 12 7 0
"Viruses;Hofneiviricota;Faserviricetes;Tubulavirales;Inoviridae;Inoviridae__na;Inoviridae_sp._ct1ro12" 0
0 0 0 0 0 0 0 0 0 0 0 0 2
0 0 0 0 0 0 0 0 0 0 1 1 0
0 0 0 1 0 0 0 0 0 0 0 0 0
0 0 0 0 0 0 0 0 0 0 0 0 0
0 0 0 0 0 0 0 0 1 0 0 0 0
0 0 0 0 0 0
"Viruses;Hofneiviricota;Faserviricetes;Tubulavirales;Inoviridae;Inoviridae__na;Inoviridae_sp._ctaKY11" 0
0 0 0 0 0 0 0 0 0 0 0 0 0
0 0 0 0 0 0 0 0 0 0 0 0 0
0 0 0 4 2 0 0 0 0 0 0 0 0
0 0 0 0 0 0 0 0 0 0 0 0 0
0 0 0 0 0 0 0 0 0 0 0 0 0
0 0 0 0 0 0
"Viruses;Hofneiviricota;Faserviricetes;Tubulavirales;Inoviridae;Inoviridae__na;Inoviridae_sp._ctDEu7" 0
0 0 0 0 0 0 0 0 0 0 0 0 0
0 0 0 0 0 0 0 0 0 0 0 0 0
0 0 0 0 2 0 0 0 0 0 0 0 0
0 0 0 0 0 0 0 0 0 0 0 0 0
0 0 0 0 0 0 0 0 0 0 0 0 0
0 10 0 0 0 0
"Viruses;Hofneiviricota;Faserviricetes;Tubulavirales;Inoviridae;Inoviridae__uc;Inoviridae__uc" 0 0
0 0 0 0 0 0 0 0 0 0 1 3 0
0 0 0 0 0 0 0 0 0 0 0 0 0
0 0 10 16 0 0 1 0 0 2 0 0 0
0 0 0 0 0 0 0 0 0 0 0 0 0
0 1 0 0 0 0 2 1 2 4 0 1 0
0 0 0 0 0
"Viruses;Hofneiviricota;Faserviricetes;Tubulavirales;Inoviridae;Inovirus;Inovirus_sp." 0 0 0
0 0 0 0 0 0 0 0 0 0 0 0 0
0 0 0 0 0 0 0 0 0 0 0 0 0
0 0 0 0 0 0 0 0 0 0 0 0 0
0 0 0 0 0 0 0 0 0 0 0 0 0
0 0 0 0 0 2 3 1 1 0 0 0 0
0 0 0 0
"Viruses;Kitrinoviricota;Alsuviricetes;Martellivirales;Endornaviridae;Alphaendornavirus;Bell_pepper_alphaend
ornavirus" 0 0 0 1 0 0 0 0 0 0 0 0
0 0 0 0 0 0 0 0 0 0 0 0 0
0 0 0 0 0 0 0 0 0 0 0 0 0
0 0 0 0 0 0 0 0 0 0 0 0 0

0 0 0 0 0 0 0 0 0 0 0 0 0
0 0 0 0 0 0 0 0
"Viruses;Kitrinoviricota;Alsuviricetes;Martellivirales;Virgaviridae;Tobamovirus;Chili_pepper_mild_mottle_viru
s" 0 0 0 0 0 0 0 0 0 0 0 0 0
0 0 1 0 0 0 0 0 0 0 0 0 0
0 0 0 0 0 0 0 0 0 0 0 0 0
0 0 0 0 0 0 0 0 0 0 0 0 0
0 0 0 0 0 0 0 0 0 0 0 0 0
0 0 0 0 0 0 0
"Viruses;Kitrinoviricota;Alsuviricetes;Martellivirales;Virgaviridae;Tobamovirus;Cucumber_green_mottle_mosai
c_virus"0 0 0 0 0 0 0 0 0 0 0 0 0
0 0 0 0 0 0 0 0 0 0 0 0 0
0 0 0 0 0 0 10 0 0 0 0 0 0
0 0 0 0 0 0 0 0 0 0 0 0 0
1 0 0 0 0 0 0 0 0 0 0 0 0
0 0 0 0 0 0 0
"Viruses;Kitrinoviricota;Alsuviricetes;Martellivirales;Virgaviridae;Tobamovirus;Obuda_pepper_virus" 0
0 0 0 0 0 0 0 0 0 0 0 0 0
0 0 0 0 0 0 0 0 0 1 0 0 0
0 0 0 0 0 0 0 0 0 0 0 0 0
0 0 0 0 0 0 0 0 0 0 0 0 0
0 0 0 0 0 0 0 0 0 0 0 0 0
0 0 0 0 0 0
"Viruses;Kitrinoviricota;Alsuviricetes;Martellivirales;Virgaviridae;Tobamovirus;Paprika_mild_mottle_virus"
0 0 2 0 0 0 0 0 0 0 0 0 0
0 7 4 4 6 0 0 1 1 0 4 0 0
0 0 0 0 0 0 0 0 0 0 0 0 1
0 0 0 0 0 0 0 0 0 0 0 0 0
0 0 0 0 0 0 0 0 0 0 0 0 0
0 0 0 0 0 0 0
"Viruses;Kitrinoviricota;Alsuviricetes;Martellivirales;Virgaviridae;Tobamovirus;Pepper_mild_mottle_virus"
0 0 12 0 0 0 0 0 0 0 0 0 0
0 100 81 156 8 0 0 112 104 49 539 0 0
0 0 0 0 0 0 1 0 0 0 0 0 10
3 0 0 0 0 0 0 0 0 0 0 0 0
10 0 0 0 0 0 0 0 0 0 0 3 0
0 0 0 1 5 0 0
"Viruses;Kitrinoviricota;Alsuviricetes;Martellivirales;Virgaviridae;Tobamovirus;Streptocarpus_flower_break_vir
us" 0 0 0 0 0 0 0 0 0 0 0 0 0
0 0 0 0 0 0 0 0 0 0 1 0 0
0 0 0 0 0 0 0 0 0 0 0 0 0
0 0 0 0 0 0 0 0 0 0 0 0 0
0 0 0 0 0 0 0 0 0 0 0 0 0
0 0 0 0 0 0 0
"Viruses;Kitrinoviricota;Alsuviricetes;Martellivirales;Virgaviridae;Tobamovirus;Tobacco_mild_green_mosaic_vi
rus" 0 0 0 0 0 0 0 0 0 0 0 0 0
0 11 17 11 0 0 0 0 4 1 1 0 0
0 0 0 0 0 0 0 0 0 0 0 0 3
0 0 0 0 0 0 0 0 0 0 0 0 0

0 0 0 0 0 0 0 0 0 0 0 0 0
0 0 0 0 1 0 0
"Viruses;Kitrinoviricota;Alsuviricetes;Martellivirales;Virgaviridae;Tobamovirus;Tobamovirus__uc" 0
0 0 0 0 0 0 0 0 1 0 0 0 0
0 1 1 0 0 0 0 1 0 0 0 0 0
0 0 0 0 0 0 0 0 1 0 0 0 1
0 0 0 0 0 0 0 0 0 0 0 0 0
0 0 0 0 0 0 0 0 0 0 0 0 0
0 0 0 1 0 0
"Viruses;Kitrinoviricota;Alsuviricetes;Martellivirales;Virgaviridae;Tobamovirus;Tomato_mosaic_virus" 0
0 0 0 0 0 0 0 0 0 0 0 0 0
0 0 0 0 0 0 0 0 0 1 0 0 0
0 0 0 0 0 0 0 0 0 0 0 0 0
0 0 0 0 0 0 0 0 0 0 0 0 0
0 0 0 0 0 0 0 0 0 0 0 0 0
0 0 0 0 0 0
"Viruses;Kitrinoviricota;Alsuviricetes;Martellivirales;Virgaviridae;Tobamovirus;Tropical_soda_apple_mosaic_virus" 0 0 0 0 0 0 0 0 0 0 0 0 0
0 1 0 0 0 0 0 0 0 0 3 0 0
0 0 0 0 0 0 0 0 0 0 0 0 0
0 0 0 0 0 0 0 0 0 0 0 0 0
0 0 0 0 0 0 0 0 0 0 0 0 0
0 0 0 0 0 0 0
"Viruses;Kitrinoviricota;Alsuviricetes;Martellivirales;Virgaviridae;Virgaviridae__uc;Virgaviridae__uc" 0
0 77 0 0 0 0 0 5 0 0 0 0 0
653 562 958 78 0 0 606 717 393 3026 0 0 19
1 0 0 0 0 24 4 2 0 0 0 60 29
0 4 0 0 0 0 0 0 0 0 0 0 77
10 0 0 5 2 0 96 0 0 5 8 0 4
0 0 0 11 0 0
"Viruses;Kitrinoviricota;Alsuviricetes;Tymovirales;Alphaflexiviridae;Potexvirus;Lettuce_virus_X"0 0
0 0 0 0 0 0 0 0 0 0 0 0 0
0 0 0 0 0 0 0 0 0 0 0 37 1
1 0 0 0 0 0 0 0 0 0 0 1 0
0 0 0 0 0 0 0 0 0 0 0 0 0
0 0 0 0 0 0 0 0 0 0 0 0 0
0 0 0 0 0
"Viruses;Kitrinoviricota;Alsuviricetes;Tymovirales;Alphaflexiviridae;Potexvirus;Pepino_mosaic_virus" 0
0 4 0 0 0 0 0 8 0 0 0 0 0
12 12 0 0 0 0 22 28 1 6 0 0 7450
206 111 51 0 0 0 0 0 0 0 0 152 170
0 4 0 0 15 4 0 0 0 0 21 5 107
0 0 0 0 0 0 0 0 0 2 4 0 1
0 0 2 48 7 0
"Viruses;Kitrinoviricota;Alsuviricetes;Tymovirales;Betaflexiviridae;Betaflexiviridae__uc;Betaflexiviridae__uc"
0 0 0 0 0 0 0 0 0 0 0 0 0
0 0 0 0 0 0 0 0 0 0 0 0 0
0 0 0 0 0 0 0 0 0 0 0 0 0
0 0 0 0 0 0 0 0 0 0 0 0 0

0 0 0 0 0 0 0 0 0 0 0 1 0
0 0 0 0 0 0 0
"Viruses;Kitrinoviricota;Alsuviricetes;Tymovirales;Betaflexiviridae;Carlavirus;Carlavirus__uc" 0 0
0 0 0 0 0 0 0 0 0 0 0 0 0
0 0 0 0 0 0 0 0 0 0 0 0 0
0 0 0 0 0 0 0 0 0 0 0 0 0
0 0 0 0 0 0 0 0 0 0 0 0 0
0 0 0 0 0 0 0 0 2 0 0 0 0
0 0 0 0 0
"Viruses;Kitrinoviricota;Alsuviricetes;Tymovirales;Tymovirales__na;Tymovirales__na;Erysiphe_necator_associa
ted_flexivirus_3" 0 0 0 0 0 0 0 0 0 0 186
0 0 0 0 0 0 0 0 0 0 0 0 0
0 0 0 0 0 0 0 0 0 0 0 0 0
0 0 0 0 0 0 0 0 0 0 0 0 0
0 0 0 0 0 0 0 0 0 0 0 0 0
0 0 0 0 0 0 0 0 0
"Viruses;Kitrinoviricota;Alsuviricetes;Tymovirales;Tymovirales__na;Tymovirales__na;Tymovirales_sp." 0
0 0 0 0 0 0 0 0 0 0 0 0 0
0 0 0 0 0 0 0 0 0 0 0 0 2
0 0 0 0 0 0 0 0 0 0 0 0 0
0 0 0 0 0 0 0 0 0 0 0 0 0
0 0 0 0 0 0 0 0 0 0 0 0 0
0 0 0 0 0 0
"Viruses;Kitrinoviricota;Alsuviricetes;Tymovirales;Tymovirales__uc;Tymovirales__uc;Tymovirales__uc" 0
0 2 0 0 0 0 0 3 0 0 0 0 0
3 0 0 0 0 0 6 6 0 1 0 0 2447
78 58 31 0 0 0 0 0 0 0 0 53 52
0 9 0 0 1 2 0 0 0 0 11 1 33
0 0 0 0 0 1 0 0 0 2 0 0 0
0 0 0 14 3 0
"Viruses;Kitrinoviricota;Flasuviricetes;Amarillovirales;Flaviviridae;Hepacivirus;Hepacivirus_C" 0 0
0 0 0 0 0 0 0 0 0 0 0 0 0
0 0 0 0 0 0 0 0 0 0 0 0 0
0 0 0 0 0 0 0 1 0 0 0 0 0
0 0 0 0 0 0 0 0 0 0 0 0 0
0 0 0 0 0 0 0 0 0 0 0 0 0
0 0 0 0 0
"Viruses;Kitrinoviricota;Flasuviricetes;Amarillovirales;Flaviviridae;Pegivirus;Pegivirus__uc" 0 0
0 0 0 0 0 0 0 0 0 0 0 0 0
0 0 0 0 0 0 0 0 0 0 0 0 0
0 0 0 0 0 0 0 0 0 0 0 0 0
0 0 0 0 0 14 0 0 0 0 0 0 0
0 0 0 0 0 0 0 0 0 0 0 0 0
0 0 0 0 0
"Viruses;Lenarviricota;Amabiliviricetes;Wolframvirales;Narnaviridae;Narnaviridae__na;Narnaviridae_sp."
0 0 0 2 0 0 0 0 0 0 0 0 0
0 0 0 0 0 0 0 0 0 0 0 0 0
0 0 0 0 0 0 0 0 0 0 0 0 0
0 0 0 0 0 0 0 0 0 0 0 0 0

0 0 0 0 0 0 0 0 0 0 0 0 0
0 0 0 0 0 0 0
"Viruses;Lenarviricota;Howeltoviricetes;Cryppavirales;Mitoviridae;Mitoviridae__na;Mitoviridae_sp." 0
0 0 0 0 0 0 0 2 0 0 0 0 0
0 0 0 0 0 0 0 0 0 0 0 0 0
0 0 0 0 0 0 0 0 0 0 0 1 0
0 0 0 0 0 0 0 0 0 0 1 0 0
0 0 0 0 0 0 0 0 0 0 0 0 0
0 0 0 0 0 0
"Viruses;Negarnaviricota;Insthoviricetes;Articulavirales;Orthomyxoviridae;Alphainfluenzavirus;Influenza_A_vi
rus" 0 0 2 0 0 0 0 0 0 3 0 0 0
0 0 0 0 0 0 0 0 0 0 0 0 0
0 0 0 0 0 0 0 0 0 0 0 0 0
0 0 1 0 0 0 0 14 0 0 0 0 0
0 0 0 0 0 0 0 0 0 0 0 0 0
0 0 0 0 1 1 0
"Viruses;Negarnaviricota;Monjiviricetes;Mononegavirales;Pneumoviridae;Orthopneumovirus;Bovine_orthopne
umovirus" 0 0 0 0 0 0 0 0 0 0 0 0
0 0 0 0 0 0 0 0 0 0 0 0 0
0 0 0 0 0 0 0 0 0 0 0 0 0
0 0 0 0 0 0 0 0 0 0 0 0 0
0 0 0 0 0 0 0 0 0 6 0 0 0
0 0 0 0 0 0 0 0
"Viruses;Negarnaviricota;Monjiviricetes;Mononegavirales;Rhabdoviridae;Rhabdoviridae__na;Wheat_rosette_st
unt_virus" 0 0 0 0 0 0 0 0 2 0 0 0
0 0 0 0 0 0 0 0 0 0 0 0 0
0 0 0 0 0 0 0 0 0 0 0 0 0
0 0 0 0 0 0 0 0 0 0 0 0 0
0 0 0 0 0 0 0 0 0 0 0 0 0
0 0 0 0 0 0 0 0
"Viruses;Nucleocytoviricota;Megaviricetes;Algavirales;Phycodnaviridae;Chlorovirus;Acanthocystis_turfacea_C
hlorella_virus_MN0810.1" 0 0 0 0 0 0 0 0 0 0
0 0 0 0 0 0 0 0 0 0 0 0 0
0 0 0 0 0 0 0 0 0 0 0 0 0
0 0 0 0 0 0 0 0 0 0 0 0 0
0 0 0 0 0 0 0 0 0 0 0 0 0
0 0 0 0 0 0 0 0 3 0
"Viruses;Nucleocytoviricota;Megaviricetes;Algavirales;Phycodnaviridae;Chlorovirus;Acanthocystis_turfacea_C
hlorella_virus_OR0704.3" 0 0 0 0 0 0 0 0 0 0
0 0 0 0 0 0 0 0 0 0 0 0 0
0 0 0 0 0 0 0 0 0 0 0 0 0
0 0 0 0 0 0 0 0 0 0 0 2 0
0 0 0 0 0 0 0 0 0 0 0 0 0
0 0 0 0 0 0 0 0 0 0
"Viruses;Nucleocytoviricota;Megaviricetes;Algavirales;Phycodnaviridae;Chlorovirus;Chlorovirus__uc" 0
1 0 3 1 2 0 0 0 0 0 0 0 0
0 0 0 0 0 0 0 0 0 0 0 0 0
0 0 0 0 0 0 0 0 0 0 0 0 0
0 0 0 0 0 0 0 1 0 0 0 0 0

0 0 0 0 0 0 0 0 0 0 0 0 0
0 0 0 0 0 0
"Viruses;Nucleocytoviricota;Megaviricetes;Algavirales;Phycodnaviridae;Chlorovirus;Paramecium_bursaria_Chlorella_virus_NY2A" 0 0 0 0 0 0 0 0 0 0 0
0 0 0 0 0 0 0 0 0 0 0 0 0
0 0 0 0 0 0 0 0 0 0 0 0 0
0 0 0 0 0 0 0 0 0 0 2 0 0
0 0 0 0 0 0 0 0 0 0 0 0 0
0 0 0 0 0 0 0 0 0
"Viruses;Nucleocytoviricota;Megaviricetes;Algavirales;Phycodnaviridae;Phycodnaviridae__na;Aureococcus_anophagefferens_virus" 0 0 0 0 0 0 0 0 0 0 0
0 0 0 0 0 0 0 0 0 0 0 0 0
0 0 0 0 0 0 0 0 0 0 0 0 0
0 0 0 0 0 0 0 0 0 0 0 0 0
0 0 0 0 0 0 0 0 0 0 0 0 1
0 0 0 0 0 0 0 0 0
"Viruses;Nucleocytoviricota;Megaviricetes;Algavirales;Phycodnaviridae;Phycodnaviridae__na;Chrysochromulina_ericina_virus" 0 0 0 0 8 0 0 0 0 0 0
0 0 0 0 0 0 0 0 0 0 0 0 0
0 0 0 0 0 0 0 0 0 0 0 0 0
0 0 0 0 0 0 0 0 0 0 0 0 0
0 0 0 0 0 0 0 0 0 0 0 0 0
0 0 0 0 0 0 0 0 0
"Viruses;Nucleocytoviricota;Megaviricetes;Algavirales;Phycodnaviridae;Phycodnaviridae__na;Organic_Lake_phycodnavirus_1" 0 0 0 1 0 0 0 0 0 0 0
0 0 0 0 0 0 0 0 0 0 0 0 0
0 0 0 0 0 0 0 0 0 0 0 0 0
0 0 0 0 0 0 0 0 0 0 0 0 0
0 0 0 0 0 0 0 0 0 0 0 0 0
0 0 0 0 0 0 0 0 0
"Viruses;Nucleocytoviricota;Megaviricetes;Algavirales;Phycodnaviridae;Prasinovirus;Prasinovirus__uc" 0
0 0 0 0 0 0 0 0 0 0 0 0 0
0 0 0 0 0 0 0 0 0 0 0 0 0
0 0 0 0 0 0 0 0 0 0 0 0 0
0 3 0 0 0 0 0 0 0 0 0 0 0
0 0 0 0 0 0 0 0 0 0 0 0 0
0 0 0 0 0 0
"Viruses;Nucleocytoviricota;Megaviricetes;Algavirales;Phycodnaviridae;Prasinovirus;Yellowstone_lake_phycodnavirus_2" 0 0 0 0 0 0 0 0 0 0 0 0
0 0 0 0 0 1 0 0 0 0 0 0 0
0 0 0 0 0 0 0 0 0 0 0 0 0
0 0 0 0 0 0 0 0 0 0 0 0 0
0 0 0 0 0 0 0 0 0 0 0 0 0
0 0 0 0 0 0 0 0
"Viruses;Nucleocytoviricota;Megaviricetes;Algavirales;Phycodnaviridae;Prasinovirus;Yellowstone_lake_phycodnavirus_3" 0 0 0 1 0 0 0 0 0 0 0 0
0 0 0 0 0 0 0 0 0 0 0 0 0
0 0 0 0 0 0 0 0 0 0 0 0 0
0 0 0 0 0 0 0 0 0 0 0 0 0

0 0 0 0 0 0 0 0 0 0 0 0 0
0 0 0 0 0 0 0 0
"Viruses;Nucleocytoviricota;Megaviricetes;Algavirales;Phycodnaviridae;Prymnesiovirus;Prymnesiovirus__uc"
0 0 0 0 0 0 0 0 0 0 0 0 0
0 0 0 0 0 0 0 0 0 0 0 0 0
0 0 0 0 0 0 0 0 0 0 0 0 0
0 0 0 0 0 0 0 0 3 0 0 0 0
0 0 0 0 0 0 0 0 0 0 0 0 0
0 0 0 0 0 0 0
"Viruses;Nucleocytoviricota;Megaviricetes;Imitervirales;Mimiviridae;Indivirus;Indivirus_ILV1" 0 0
0 0 0 1 0 0 0 0 0 0 0 0 0
0 0 0 0 0 0 0 0 0 0 0 0 0
0 0 0 0 0 0 0 0 0 0 0 0 0
0 0 0 0 0 0 0 0 0 0 0 0 0
0 0 0 0 0 0 0 0 0 0 0 0 0
0 0 0 0 0
"Viruses;Nucleocytoviricota;Megaviricetes;Imitervirales;Mimiviridae;Klosneuvirus;Bodo_saltans_virus" 1
0 0 0 0 0 0 0 0 0 0 0 0 0
0 0 0 0 0 0 0 0 0 0 0 0 0
0 0 0 0 0 0 0 0 0 0 0 0 0
0 0 0 0 0 0 0 0 0 0 0 0 0
0 0 0 0 0 0 0 0 0 0 0 0 0
0 0 0 0 0 0
"Viruses;Nucleocytoviricota;Megaviricetes;Imitervirales;Mimiviridae;Mimiviridae__na;Barrevirus_sp." 0
0 0 1 0 1 0 0 0 0 0 0 0 0
0 0 0 0 0 0 0 0 0 0 0 0 0
0 0 0 0 0 0 0 0 0 0 0 0 0
0 0 0 0 0 0 0 0 0 0 0 0 0
0 0 0 0 0 0 0 0 0 0 0 0 0
0 0 0 0 0 0
"Viruses;Nucleocytoviricota;Megaviricetes;Imitervirales;Mimiviridae;Mimiviridae__na;Chlorella_virus_XW01"
0 0 0 0 5 9 0 0 0 0 0 0 0
0 0 0 1 0 0 0 0 0 0 0 0 0
0 0 0 0 0 0 0 0 0 0 0 0 0
0 0 0 0 0 0 0 0 0 0 0 0 0
0 0 0 0 0 0 0 0 0 0 0 0 0
0 0 0 0 0 0 0
"Viruses;Nucleocytoviricota;Megaviricetes;Imitervirales;Mimiviridae;Mimiviridae__na;Dasosvirus_sp." 0
0 0 0 0 9 0 0 0 0 0 0 0 0
0 0 0 0 0 0 0 0 0 0 0 0 0
0 0 0 0 0 0 0 0 0 0 0 0 0
0 0 0 0 0 0 0 0 0 0 0 0 0
0 0 0 0 0 0 0 0 0 0 0 0 0
0 0 0 0 0 0
"Viruses;Nucleocytoviricota;Megaviricetes;Imitervirales;Mimiviridae;Mimiviridae__na;Edafosvirus_sp." 0
0 0 0 3 0 0 0 0 0 0 0 0 0
0 0 0 0 0 0 0 0 0 0 0 0 0
0 0 0 0 0 0 0 0 0 0 0 0 0
0 0 0 0 0 0 0 0 0 0 0 0 0

0 0 0 0 0 0 0 0 0 0 0 0 0
0 0 0 0 0 0
"Viruses;Nucleocytoviricota;Megaviricetes;Imitervirales;Mimiviridae;Mimiviridae__na;Megaviridae_environmental_sample" 0 0 0 0 0 3 0 0 0 0 0 0
0 0 0 0 0 0 0 0 0 0 0 0 0
0 0 0 0 0 0 0 0 0 0 0 0 0
0 0 0 0 0 0 0 0 0 0 0 0 0
0 0 0 0 0 0 0 0 0 0 0 0 0
0 0 0 0 0 0 0 0
"Viruses;Nucleocytoviricota;Megaviricetes;Imitervirales;Mimiviridae;Mimiviridae__na;Mimiviridae_sp._ChoanoV1" 0 0 0 0 0 1 0 0 0 0 0 0 0
0 0 0 0 0 0 0 0 0 0 0 0 0
0 0 0 0 0 0 0 0 0 0 0 0 0
0 0 0 0 0 0 0 0 0 0 0 0 0
0 0 1 0 0 0 0 0 0 0 0 0 0
0 0 0 0 0 0 0
"Viruses;Nucleocytoviricota;Megaviricetes;Imitervirales;Mimiviridae;Mimiviridae__na;Yellowstone_lake_mimivirus" 0 0 0 0 1 0 0 0 0 0 0 0 0
0 0 0 0 0 0 0 0 0 0 0 0 0
0 0 0 0 0 0 0 0 0 0 0 0 0
0 0 0 0 0 0 0 0 0 0 0 0 0
0 0 0 0 0 0 0 0 0 0 0 0 0
0 0 0 0 0 0 0
"Viruses;Nucleocytoviricota;Megaviricetes;Imitervirales;Mimiviridae;Mimivirus;Mimivirus_LCMiAC01" 0
0 1 0 0 0 0 0 0 0 0 0 0 0
0 0 0 0 0 0 0 0 0 0 0 0 0
0 0 0 0 0 0 0 0 0 0 0 0 0
0 0 0 0 0 0 0 0 0 0 0 0 0
0 0 0 0 0 0 0 0 0 0 0 0 0
0 0 0 0 0 0
"Viruses;Nucleocytoviricota;Megaviricetes;Megaviricetes__uc;Megaviricetes__uc;Megaviricetes__uc;Megaviricetes__uc" 0 0 0 0 0 3 0 0 0 0 0 0 0
0 0 0 0 0 0 0 0 0 0 0 0 0
0 0 0 0 0 0 0 0 0 0 0 0 0
0 0 0 0 0 0 0 0 0 0 0 0 0
0 0 0 0 0 0 0 0 0 0 0 0 0
0 0 0 0 0 0 0
"Viruses;Nucleocytoviricota;Megaviricetes;Pimascovirales;Iridoviridae;Iridoviridae__na;carnivorous_sponge_associated_iridovirus" 0 0 0 0 0 0 0 0 0 0 0
0 0 0 0 0 1 0 0 0 0 0 0 0
0 0 0 0 0 0 0 0 0 0 0 0 0
0 0 0 0 0 0 0 0 0 0 0 0 0
0 0 0 0 0 0 0 0 0 0 0 0 0
0 0 0 0 0 0 0 0 0
"Viruses;Nucleocytoviricota;Megaviricetes;Pimascovirales;Iridoviridae;Iridoviridae__na;Erythrocytic_necrosis_virus" 0 0 0 0 0 0 0 0 0 0 0 0 0
0 0 0 0 0 0 0 0 0 0 0 0 0
0 0 0 0 0 0 0 0 0 5 0 0 0
0 0 0 0 0 0 0 0 0 0 0 0 0

0 0 0 0 0 0 0 0 0 0 0 0 0
0 0 0 0 0 0 0
"Viruses;Nucleocytoviricota;Megaviricetes;Pimascovirales;Iridoviridae;Iridoviridae__na;Iridovirus_LCIVAC01"
0 0 0 0 0 0 0 0 0 0 0 0 0
0 0 0 0 0 0 0 0 0 0 0 0 0
0 0 0 0 0 0 0 0 0 0 0 0 0
0 0 0 0 0 0 0 0 1 0 0 0 0
0 0 0 0 0 0 0 0 0 0 0 0 0
0 0 0 0 0 0 0
"Viruses;Nucleocytoviricota;Megaviricetes;Pimascovirales;Iridoviridae;Lymphocystivirus;Lymphocystis_disease
_virus_1" 0 0 0 0 0 1 0 0 0 0 0 0
0 0 0 0 0 0 0 0 0 0 0 0 0
0 0 0 0 0 0 0 0 0 0 0 0 0
0 0 0 0 0 0 0 0 0 0 0 0 0
0 0 0 0 0 0 0 0 0 0 0 0 0
0 0 0 0 0 0 0 0
"Viruses;Nucleocytoviricota;Megaviricetes;Pimascovirales;Marseilleviridae;Marseilleviridae__na;Marseillevirus_
LCMAC101" 0 0 1 0 0 0 0 0 0 0 0 0
0 0 0 0 0 0 0 0 0 0 0 0 0
0 0 0 0 0 0 0 0 0 0 0 0 0
0 0 0 0 0 0 0 0 0 0 0 0 0
0 0 0 0 0 0 0 0 0 0 0 0 0
0 0 0 0 0 0 0 0
"Viruses;Nucleocytoviricota;Megaviricetes;Pimascovirales;Marseilleviridae;Marseilleviridae__na;Marseillevirus_
LCMAC201" 0 0 0 0 2 0 0 0 0 0 0 0
0 0 0 0 0 0 0 0 0 0 0 0 0
0 0 0 0 0 0 0 0 0 0 0 0 0
0 0 0 0 0 0 0 0 0 0 0 0 0
0 0 0 0 0 0 0 0 0 0 0 0 0
0 0 0 0 0 0 0 0
"Viruses;Nucleocytoviricota;Megaviricetes;Pimascovirales;Marseilleviridae;Marseilleviridae__na;Marseillevirus_
LCMAC202" 0 0 0 0 0 0 0 0 0 0 0 0
0 0 0 0 0 0 0 0 0 0 0 0 0
0 0 0 0 0 0 0 0 0 0 0 0 0
0 0 0 0 0 0 0 0 0 6 0 0 0
0 0 1 0 0 0 0 0 0 0 0 0 0
0 0 0 0 0 0 0 0
"Viruses;Nucleocytoviricota;Nucleocytoviricota__na;Nucleocytoviricota__na;Nucleocytoviricota__na;Nucleocyt
oviricota__na;Nucleocytoviricota_sp." 0 0 0 0 3 0 0 0 0
0 0 0 0 0 0 0 0 0 0 0 0 0
0 0 0 0 0 0 0 0 0 0 0 0 0
0 0 0 0 0 0 0 0 0 0 0 0 0
0 0 0 0 0 0 0 0 0 0 0 0 0
0 0 0 0 0 0 0 0 0 0 0
"Viruses;Nucleocytoviricota;Pokkesviricetes;Chitovirales;Poxviridae;Leporipoxvirus;Myxoma_virus" 0
0 0 0 0 0 0 0 1 0 0 0 0 0
0 0 0 0 0 0 0 0 0 0 0 0 0
0 0 0 0 0 0 0 0 0 0 0 0 0
0 0 0 0 0 0 0 0 0 0 0 0 0

0 0 0 0 0 0 0 0 0 0 0 0 0
0 0 0 0 0 0
"Viruses;Nucleocytoviricota;Pokkesviricetes;Chitovirales;Poxviridae;Molluscipoxvirus;Molluscum_contagiosum_virus" 0 0 0 0 0 0 0 0 0 1 0 0 0
0 0 0 0 0 0 0 0 0 0 0 0 0
0 0 0 0 0 0 0 0 0 0 0 0 0
0 0 0 0 0 0 0 0 0 0 0 0 0
0 0 0 0 0 0 0 0 0 0 0 0 0
0 0 0 0 0 0 0
"Viruses;Nucleocytoviricota;Pokkesviricetes;Chitovirales;Poxviridae;Parapoxvirus;Orf_virus" 0 0
0 0 0 0 0 0 0 0 0 0 0 0 0
0 0 0 0 0 0 0 0 0 0 0 0 0
0 0 0 0 0 0 0 0 0 0 0 0 0
1 0 0 0 0 0 0 0 0 0 0 0 0
0 0 0 0 0 0 0 0 0 0 0 0 0
0 0 0 0 0
"Viruses;Nucleocytoviricota;Pokkesviricetes;Chitovirales;Poxviridae;Suipoxvirus;Swinepox_virus" 0
0 0 0 1 0 0 0 0 0 0 0 0 0
0 0 0 0 0 0 0 0 0 0 0 0 0
0 0 0 0 0 0 0 0 0 0 0 0 0
0 0 0 0 0 0 0 0 0 0 0 0 0
0 0 0 0 0 0 0 0 0 0 0 0 0
0 0 0 0 0 0
"Viruses;Nucleocytoviricota;Pokkesviricetes;Chitovirales;Poxviridae;Vespertilionpoxvirus;Eptesipox_virus"
0 0 0 0 0 2 0 0 0 0 0 0 0
0 0 0 0 0 0 0 0 0 0 0 0 0
0 0 0 0 0 0 0 0 0 0 0 0 0
0 0 0 0 0 0 0 0 0 0 0 0 0
0 0 0 0 0 0 0 0 0 0 0 0 0
0 0 0 0 0 0 0
"Viruses;Phixviricota;Malgrandaviricetes;Petitvirales;Microviridae;Microviridae__na;Apis_mellifera_associated_microvirus_1" 0 0 0 0 6 5 0 0 0 0 0 0
0 0 0 0 0 0 0 0 0 0 1 0 0
0 0 0 0 0 0 0 0 0 0 0 0 0
0 0 0 0 0 0 0 0 0 2 0 0 0
0 0 0 0 0 0 0 0 0 0 0 0 0
0 0 0 0 0 0 1 0
"Viruses;Phixviricota;Malgrandaviricetes;Petitvirales;Microviridae;Microviridae__na;Apis_mellifera_associated_microvirus_14" 0 0 0 0 0 1 0 0 0 0 0 0
0 0 0 0 0 0 0 0 0 0 0 0 0
0 0 0 0 0 0 0 0 0 0 0 0 0
0 0 0 0 0 0 0 0 0 0 0 0 0
0 0 0 0 0 0 0 0 0 0 0 0 0
0 0 0 0 0 0 0 0
"Viruses;Phixviricota;Malgrandaviricetes;Petitvirales;Microviridae;Microviridae__na;Apis_mellifera_associated_microvirus_15" 0 0 0 0 3 0 0 0 0 0 0 0
0 0 0 0 0 0 0 0 0 0 0 0 0
0 0 0 0 0 0 0 0 0 0 0 0 0
0 0 0 0 0 0 0 0 0 0 0 0 0

0 0 0 0 0 0 0 0 0 0 0 0 0
0 0 0 0 0 0 0 0
"Viruses;Phixviricota;Malgrandaviricetes;Petitvirales;Microviridae;Microviridae__na;Apis_mellifera_associated_
microvirus_18" 0 0 0 0 1 0 0 0 0 0 0 0
0 0 0 0 0 0 0 0 0 0 0 0 0
0 0 0 0 0 0 0 0 0 0 0 0 0
0 0 0 0 0 0 0 0 0 0 0 0 0
0 0 0 0 0 0 0 0 0 0 0 0 0
0 0 0 0 0 0 0 0
"Viruses;Phixviricota;Malgrandaviricetes;Petitvirales;Microviridae;Microviridae__na;Apis_mellifera_associated_
microvirus_19" 0 0 0 0 0 4 0 0 0 0 0 0
0 0 0 0 0 0 0 0 0 0 0 0 0
0 0 0 0 0 0 0 0 0 0 0 0 0
0 0 0 0 0 0 0 0 0 10 0 0 0
0 0 0 0 0 0 0 0 0 0 0 0 0
0 0 0 0 0 0 0 0
"Viruses;Phixviricota;Malgrandaviricetes;Petitvirales;Microviridae;Microviridae__na;Apis_mellifera_associated_
microvirus_2" 0 0 0 1 2 1 0 0 0 0 0 0
0 0 0 0 0 0 0 0 0 0 0 0 0
0 0 0 0 0 0 0 0 0 0 0 0 0
0 0 0 0 0 0 0 0 0 1 0 0 0
0 0 0 0 0 0 0 0 0 0 0 0 0
0 0 0 0 0 0 0 0
"Viruses;Phixviricota;Malgrandaviricetes;Petitvirales;Microviridae;Microviridae__na;Apis_mellifera_associated_
microvirus_20" 0 0 0 1 0 1 0 0 0 0 0 0
0 0 0 0 0 0 0 0 0 0 0 0 0
0 0 0 0 0 0 0 0 0 0 0 0 0
0 0 0 0 0 0 0 0 0 0 0 0 0
0 0 0 0 0 0 0 0 0 0 0 0 0
0 0 0 0 0 0 0 0
"Viruses;Phixviricota;Malgrandaviricetes;Petitvirales;Microviridae;Microviridae__na;Apis_mellifera_associated_
microvirus_21" 0 0 0 0 0 1 0 0 0 0 0 0
0 0 0 0 0 0 0 0 0 0 0 0 0
0 0 0 0 0 0 0 0 0 0 0 0 0
0 0 0 0 0 0 0 0 0 1 0 0 0
0 0 0 0 0 0 0 0 0 0 0 0 0
0 0 0 0 0 0 1 0
"Viruses;Phixviricota;Malgrandaviricetes;Petitvirales;Microviridae;Microviridae__na;Apis_mellifera_associated_
microvirus_22" 0 0 0 0 2 7 0 0 0 0 0 0
0 0 0 0 0 0 0 0 0 0 0 0 0
0 0 0 0 0 0 0 0 0 0 0 0 0
0 0 0 0 0 0 0 0 0 0 0 0 0
0 0 0 0 0 0 0 0 0 0 0 0 0
0 0 0 0 0 0 0 0
"Viruses;Phixviricota;Malgrandaviricetes;Petitvirales;Microviridae;Microviridae__na;Apis_mellifera_associated_
microvirus_23" 0 0 0 0 6 3 0 0 0 0 0 0
0 0 0 0 0 0 0 0 0 0 0 0 0
0 0 0 0 0 0 0 0 0 0 0 0 0
0 0 0 0 0 0 0 0 0 1 0 0 0

0 0 0 0 0 0 0 0 0 0 0 0 0
0 0 0 0 0 0 0 0
"Viruses;Phixviricota;Malgrandaviricetes;Petitvirales;Microviridae;Microviridae__na;Apis_mellifera_associated_
microvirus_24" 0 0 0 0 0 3 0 0 0 0 0 0
0 0 0 0 0 0 0 0 0 0 0 0 0
0 0 0 0 0 0 0 0 0 0 0 0 0
0 0 0 0 0 0 0 0 0 0 0 0 0
0 0 0 0 0 0 0 0 0 0 0 0 0
0 0 0 0 0 0 0 0
"Viruses;Phixviricota;Malgrandaviricetes;Petitvirales;Microviridae;Microviridae__na;Apis_mellifera_associated_
microvirus_25" 0 0 0 0 0 1 0 0 0 0 0 0
0 0 0 0 0 0 0 0 0 0 0 0 0
0 0 0 0 0 0 0 0 0 0 0 0 0
0 0 0 0 0 0 0 0 0 0 0 0 0
0 0 0 0 0 0 0 0 0 0 0 0 0
0 0 0 0 0 0 0 0
"Viruses;Phixviricota;Malgrandaviricetes;Petitvirales;Microviridae;Microviridae__na;Apis_mellifera_associated_
microvirus_27" 0 0 0 0 2 0 0 0 0 0 0 0
0 0 0 0 0 0 0 0 0 0 0 0 0
0 0 0 0 0 0 0 0 0 0 0 0 0
0 0 0 0 0 0 0 0 0 0 0 0 0
0 0 0 0 0 0 0 0 0 0 0 0 0
0 0 0 0 0 0 0 0
"Viruses;Phixviricota;Malgrandaviricetes;Petitvirales;Microviridae;Microviridae__na;Apis_mellifera_associated_
microvirus_28" 0 0 1 0 3 0 0 0 0 0 0 0
0 0 0 0 0 0 0 0 0 0 0 1 0
0 0 0 0 0 0 0 0 0 0 0 0 0
0 0 0 0 0 0 0 0 0 0 0 0 0
0 0 0 0 0 0 0 0 0 0 0 0 0
0 0 0 0 0 0 0 0
"Viruses;Phixviricota;Malgrandaviricetes;Petitvirales;Microviridae;Microviridae__na;Apis_mellifera_associated_
microvirus_29" 0 0 1 4 13 10 0 0 0 0 0 0
0 0 0 0 0 0 0 0 0 0 0 0 0
0 0 0 0 0 0 0 0 0 0 0 0 0
0 0 0 0 0 0 0 0 0 1 0 0 0
0 0 0 0 0 0 0 0 0 0 0 0 0
0 0 0 0 0 0 0 0
"Viruses;Phixviricota;Malgrandaviricetes;Petitvirales;Microviridae;Microviridae__na;Apis_mellifera_associated_
microvirus_3" 0 0 0 0 3 0 0 0 0 0 0 0
0 0 0 0 0 0 0 0 0 0 1 0 0
0 0 0 0 0 0 0 0 0 0 0 0 0
0 0 0 0 0 0 0 0 0 0 0 0 0
0 0 0 0 0 0 0 0 0 0 0 0 0
0 0 0 0 0 0 0 0
"Viruses;Phixviricota;Malgrandaviricetes;Petitvirales;Microviridae;Microviridae__na;Apis_mellifera_associated_
microvirus_31" 0 0 1 3 74 89 0 0 0 0 0 0
0 0 0 0 0 0 0 0 0 0 0 0 0
0 0 0 0 0 0 0 0 0 0 0 0 0
0 0 0 0 0 0 0 0 0 6 0 0 0

0 0 0 0 0 0 0 0 0 0 0 0 0
0 0 0 0 0 0 3 0
"Viruses;Phixviricota;Malgrandaviricetes;Petitvirales;Microviridae;Microviridae__na;Apis_mellifera_associated_
microvirus_32" 0 0 0 3 29 45 0 0 0 0 0 0
0 0 0 0 0 0 0 0 0 0 1 0 0
0 0 0 0 0 0 0 0 0 0 0 0 0
0 0 0 0 0 0 0 0 0 3 0 0 0
0 0 0 0 0 0 0 0 0 0 0 0 0
0 0 0 0 0 0 0 0
"Viruses;Phixviricota;Malgrandaviricetes;Petitvirales;Microviridae;Microviridae__na;Apis_mellifera_associated_
microvirus_33" 0 0 0 2 3 2 0 0 0 0 0 0
0 0 0 0 0 0 0 0 0 0 0 0 0
0 0 0 0 0 0 0 0 0 0 0 0 0
0 0 0 0 0 0 0 0 0 0 0 0 0
0 0 0 0 0 0 0 0 0 0 0 0 0
0 0 0 0 0 0 0 0
"Viruses;Phixviricota;Malgrandaviricetes;Petitvirales;Microviridae;Microviridae__na;Apis_mellifera_associated_
microvirus_38" 0 0 0 2 1 0 0 0 0 0 0 0
0 0 0 0 0 0 0 0 0 0 0 0 0
0 0 0 0 0 0 0 0 0 0 0 0 0
0 0 0 0 0 0 0 0 0 0 0 0 0
0 0 0 0 0 0 0 0 0 0 0 0 0
0 0 0 0 0 0 0 0
"Viruses;Phixviricota;Malgrandaviricetes;Petitvirales;Microviridae;Microviridae__na;Apis_mellifera_associated_
microvirus_40" 0 0 0 1 8 0 0 0 0 0 0 0
0 0 0 0 0 0 0 0 0 0 0 0 0
0 0 0 0 0 0 0 0 0 0 0 0 0
0 0 0 0 0 0 0 0 0 0 0 0 0
0 0 0 0 0 0 0 0 0 0 0 0 0
0 0 0 0 0 0 0 0
"Viruses;Phixviricota;Malgrandaviricetes;Petitvirales;Microviridae;Microviridae__na;Apis_mellifera_associated_
microvirus_41" 0 0 0 0 4 0 0 0 0 0 0 0
0 0 0 0 0 0 0 0 0 0 0 0 0
0 0 0 0 0 0 0 0 0 0 0 0 0
0 0 0 0 0 0 0 0 0 0 0 0 0
0 0 0 0 0 0 0 0 0 0 0 0 0
0 0 0 0 0 0 0 0
"Viruses;Phixviricota;Malgrandaviricetes;Petitvirales;Microviridae;Microviridae__na;Apis_mellifera_associated_
microvirus_45" 0 0 0 2 25 1 0 0 0 0 0 0
0 0 0 0 0 0 0 0 0 0 0 0 0
0 0 0 0 0 0 0 0 0 0 0 0 0
0 0 0 0 0 0 0 0 0 7 0 0 0
0 0 0 0 0 0 0 0 0 0 0 0 0
0 0 0 0 0 0 0 0
"Viruses;Phixviricota;Malgrandaviricetes;Petitvirales;Microviridae;Microviridae__na;Apis_mellifera_associated_
microvirus_46" 0 0 0 0 1 0 0 0 0 0 0 0
0 0 0 0 0 0 0 0 0 0 0 0 0
0 0 0 0 0 0 0 0 0 0 0 0 0
0 0 0 0 0 0 0 0 0 0 0 0 0

0 0 0 0 0 0 0 0 0 0 0 0 0
0 0 0 0 0 0 0 0
"Viruses;Phixviricota;Malgrandaviricetes;Petitvirales;Microviridae;Microviridae__na;Apis_mellifera_associated_
microvirus_49" 0 0 2 1 3 0 0 0 0 0 0 0
0 0 0 0 1 0 0 0 0 0 0 1 0
0 0 0 0 0 0 0 0 0 0 0 0 0
0 0 0 1 0 0 0 0 0 0 0 0 0
0 0 0 0 0 0 0 0 0 0 0 0 0
0 0 0 0 0 0 0 0
"Viruses;Phixviricota;Malgrandaviricetes;Petitvirales;Microviridae;Microviridae__na;Apis_mellifera_associated_
microvirus_5" 0 0 0 0 0 1 0 0 0 0 0 0
0 0 0 0 0 0 0 0 0 0 0 0 0
0 0 0 0 0 0 0 0 0 0 0 0 0
0 0 0 0 0 0 0 0 0 0 0 0 0
0 0 0 0 0 0 0 0 0 0 0 0 0
0 0 0 0 0 0 0 0
"Viruses;Phixviricota;Malgrandaviricetes;Petitvirales;Microviridae;Microviridae__na;Apis_mellifera_associated_
microvirus_51" 0 0 0 0 13 0 0 0 0 0 0 0
0 0 0 0 1 0 0 0 0 0 0 0 0
0 0 0 0 0 0 0 0 0 0 0 0 0
0 0 0 0 0 0 0 0 0 7 0 0 0
0 0 0 0 0 0 0 0 0 0 0 0 0
0 0 0 0 0 0 0 0
"Viruses;Phixviricota;Malgrandaviricetes;Petitvirales;Microviridae;Microviridae__na;Apis_mellifera_associated_
microvirus_53" 0 0 0 0 1 0 0 0 0 0 0 0
0 0 0 0 0 0 0 0 0 0 0 0 0
0 0 0 0 0 0 0 0 0 0 0 0 0
0 0 0 0 0 0 0 0 0 0 0 0 0
0 0 0 0 0 0 0 0 0 0 0 0 0
0 0 0 0 0 0 0 0
"Viruses;Phixviricota;Malgrandaviricetes;Petitvirales;Microviridae;Microviridae__na;Apis_mellifera_associated_
microvirus_58" 0 0 0 0 0 2 0 0 0 0 0 0
0 0 0 0 0 0 0 0 0 0 0 0 0
0 0 0 0 0 0 0 0 0 0 0 0 0
0 0 0 0 0 0 0 0 0 0 0 0 0
0 0 0 0 0 0 0 0 0 0 0 0 0
0 0 0 0 0 0 0 0
"Viruses;Phixviricota;Malgrandaviricetes;Petitvirales;Microviridae;Microviridae__na;Apis_mellifera_associated_
microvirus_6" 0 0 0 0 1 0 0 0 0 0 0 0
0 0 0 0 0 0 0 0 0 0 0 0 0
0 0 0 0 0 0 0 0 0 0 0 0 0
0 0 0 0 0 0 0 0 0 0 0 0 0
0 0 0 0 0 0 0 0 0 0 0 0 0
0 0 0 0 0 0 0 0
"Viruses;Phixviricota;Malgrandaviricetes;Petitvirales;Microviridae;Microviridae__na;Apis_mellifera_associated_
microvirus_8" 0 0 0 0 0 0 0 0 0 0 0 0
0 0 0 0 0 0 0 0 0 0 0 0 0
0 0 0 0 0 0 0 0 0 0 0 0 0
0 0 0 0 0 0 0 0 0 1 0 0 0

0 0 0 0 0 0 0 0 0 0 0 0 0
0 0 0 0 0 0 0 0
"Viruses;Phixviricota;Malgrandaviricetes;Petitvirales;Microviridae;Microviridae__na;Arizlama_microvirus"
0 0 14 47 641 292 0 0 0 0 0 0 0
0 0 0 6 0 0 0 0 1 4 5 0 0
0 2 0 0 0 0 0 0 2 1 0 0 0
0 1 12 0 0 2 0 0 107 0 0 0 0
2 3 0 0 0 0 0 0 0 0 0 0 0
0 0 0 0 0 9 0
"Viruses;Phixviricota;Malgrandaviricetes;Petitvirales;Microviridae;Microviridae__na;Bacteriophage_N127_L503
5_C77.3" 0 0 0 0 0 0 0 0 0 0 0 0
0 0 0 0 0 0 0 0 0 0 0 0 0
0 0 0 0 0 0 0 0 0 1 0 0 0
0 0 0 0 0 0 0 0 0 0 0 0 0
0 0 0 0 0 0 0 0 0 0 0 0 0
0 0 0 0 0 0 0 0
"Viruses;Phixviricota;Malgrandaviricetes;Petitvirales;Microviridae;Microviridae__na;Capybara_microvirus_Cap
1_SP_141" 0 0 0 0 0 2 0 0 0 0 0 0
0 0 0 0 0 0 0 0 0 0 0 0 0
0 0 0 0 0 0 0 0 0 0 0 0 0
0 0 0 0 0 0 0 0 0 0 0 0 0
0 0 0 0 0 0 0 0 0 0 0 0 0
0 0 0 0 0 0 0 0
"Viruses;Phixviricota;Malgrandaviricetes;Petitvirales;Microviridae;Microviridae__na;Capybara_microvirus_Cap
1_SP_158" 0 0 0 0 0 6 0 0 0 0 0 0
0 0 0 0 0 0 0 0 0 0 0 0 0
0 0 0 0 0 0 0 0 0 0 0 0 0
0 0 0 0 0 0 0 0 0 0 0 0 0
0 0 0 0 0 0 0 0 0 0 0 0 0
0 0 0 0 0 0 0 0
"Viruses;Phixviricota;Malgrandaviricetes;Petitvirales;Microviridae;Microviridae__na;Capybara_microvirus_Cap
1_SP_162" 0 0 0 0 1 0 0 0 0 0 0 0
0 0 0 0 0 0 0 0 0 0 0 0 0
0 0 0 0 0 0 0 0 0 0 3 0 0
0 0 0 1 0 0 0 0 0 2 0 0 0
0 0 0 0 0 0 0 0 0 0 0 0 0
0 0 0 0 0 0 2 0
"Viruses;Phixviricota;Malgrandaviricetes;Petitvirales;Microviridae;Microviridae__na;Capybara_microvirus_Cap
1_SP_175" 0 0 0 0 0 1 0 0 0 0 0 0
0 0 0 0 0 0 0 0 0 0 0 0 0
0 0 0 0 0 0 0 0 0 0 0 0 0
0 0 0 0 0 0 0 0 0 0 0 0 0
0 0 0 0 0 0 0 0 0 0 0 0 0
0 0 0 0 0 0 0 0
"Viruses;Phixviricota;Malgrandaviricetes;Petitvirales;Microviridae;Microviridae__na;Capybara_microvirus_Cap
1_SP_178" 0 0 0 0 0 1 0 0 0 0 0 0
0 0 0 0 0 0 0 0 0 0 0 0 0
0 0 0 0 0 0 0 0 0 0 0 0 0
0 0 0 0 0 0 0 0 0 0 0 0 0

0 0 0 0 0 0 0 0 0 0 0 0 0
0 0 0 0 0 0 0 0
"Viruses;Phixviricota;Malgrandaviricetes;Petitvirales;Microviridae;Microviridae__na;Capybara_microvirus_Cap
1_SP_209" 0 0 0 1 4 0 0 0 0 0 0 0
0 0 0 0 0 0 0 0 0 0 0 0 0
0 0 0 0 0 0 0 0 0 0 0 0 0
0 0 0 0 0 0 0 0 0 0 0 0 0
0 0 0 0 0 0 0 0 0 0 0 0 0
0 0 0 0 0 0 0 0
"Viruses;Phixviricota;Malgrandaviricetes;Petitvirales;Microviridae;Microviridae__na;Capybara_microvirus_Cap
1_SP_228" 0 0 0 0 2 1 0 0 0 0 0 0
0 0 0 0 0 0 0 0 0 0 0 0 0
0 0 0 0 0 0 0 0 0 0 0 0 0
0 0 0 0 0 0 0 0 0 0 0 0 0
0 0 0 0 0 0 0 0 0 0 0 0 0
0 0 0 0 0 0 0 0
"Viruses;Phixviricota;Malgrandaviricetes;Petitvirales;Microviridae;Microviridae__na;Capybara_microvirus_Cap
3_SP_641" 0 0 0 1 9 0 0 0 0 0 0 0
0 0 0 0 0 0 0 0 0 0 0 0 0
0 0 0 0 0 0 0 0 0 0 0 0 0
0 0 0 0 0 0 0 0 0 0 0 0 0
0 0 0 0 0 0 0 0 0 0 0 0 0
0 0 0 0 0 0 1 0
"Viruses;Phixviricota;Malgrandaviricetes;Petitvirales;Microviridae;Microviridae__na;Capybara_microvirus_Cap
3_SP_645" 0 0 0 4 8 7 0 0 0 0 0 0
0 0 0 0 0 0 0 0 0 0 0 0 0
0 0 0 0 0 0 0 0 0 0 0 0 0
0 0 0 0 0 0 0 0 0 0 0 0 0
0 0 0 0 0 0 0 0 0 0 0 0 0
0 0 0 0 0 0 0 0
"Viruses;Phixviricota;Malgrandaviricetes;Petitvirales;Microviridae;Microviridae__na;Capybara_microvirus_Cap
3_SP_649" 0 0 0 1 0 0 0 0 0 0 0 0
0 0 0 0 0 0 0 0 0 0 0 0 0
0 0 0 0 0 0 0 0 0 0 0 0 0
0 0 0 0 0 0 0 0 0 0 0 0 0
0 0 0 0 0 0 0 0 0 0 0 0 0
0 0 0 0 0 0 0 0
"Viruses;Phixviricota;Malgrandaviricetes;Petitvirales;Microviridae;Microviridae__na;Chifec_microvirus_UA13_
14" 0 0 0 0 0 0 0 0 0 0 0 0 0
0 0 0 0 0 0 0 0 0 0 0 0 0
0 0 0 0 0 0 0 0 0 0 0 0 0
0 0 0 0 0 0 0 0 1 0 0 0 0
0 0 0 0 0 0 0 0 0 0 0 0 0
0 0 0 0 0 0 0
"Viruses;Phixviricota;Malgrandaviricetes;Petitvirales;Microviridae;Microviridae__na;Chifec_microvirus_UA13_
27" 0 0 0 0 0 0 0 0 0 0 0 0 0
0 0 0 0 0 0 0 0 0 0 0 0 0
0 0 0 1 0 0 0 0 0 0 0 0 0
0 0 0 0 0 0 0 0 0 0 0 0 0

0 0 0 0 0 0 0 0 0 0 0 0 0
0 0 0 0 0 0 0
"Viruses;Phixviricota;Malgrandaviricetes;Petitvirales;Microviridae;Microviridae__na;Dipodfec_virus_RodF1_50"
0 0 0 0 0 0 0 0 0 0 0 0 0
0 0 0 0 0 0 0 0 0 0 0 0 0
0 0 0 0 0 0 0 0 0 0 0 0 0
0 0 0 0 0 0 1 0 0 0 0 0 0
0 0 0 0 0 0 0 0 0 0 0 0 0
0 0 0 0 0 0 0
"Viruses;Phixviricota;Malgrandaviricetes;Petitvirales;Microviridae;Microviridae__na;Dipodfec_virus_RodF1_85"
0 0 0 0 0 0 0 0 0 0 0 0 0
0 1 2 1 0 0 0 0 0 0 0 0 0
0 0 0 0 0 0 0 0 0 0 0 0 0
0 0 0 0 0 0 0 0 0 0 0 0 0
0 0 0 0 0 0 0 0 0 0 0 0 0
0 0 0 0 0 0 0
"Viruses;Phixviricota;Malgrandaviricetes;Petitvirales;Microviridae;Microviridae__na;Dipodfec_virus_UOA04_R
od_1082" 0 0 0 0 1 0 0 0 0 0 0 0
0 0 0 0 0 0 0 0 0 0 0 0 0
0 0 0 0 0 0 0 0 0 0 0 0 0
0 0 0 0 0 0 0 0 0 0 0 0 0
0 0 0 0 0 0 0 0 0 0 0 0 0
0 0 0 0 0 0 0 0
"Viruses;Phixviricota;Malgrandaviricetes;Petitvirales;Microviridae;Microviridae__na;Dipodfec_virus_UOA04_R
od_495"0 0 0 0 0 0 0 0 0 0 0 0 0
0 5 1 5 0 0 0 0 0 0 0 0 0
0 0 0 0 0 0 0 0 0 0 0 0 0
0 0 0 0 0 0 0 0 0 0 0 0 0
0 0 0 0 0 0 0 0 0 0 0 0 0
0 0 0 0 0 0 0
"Viruses;Phixviricota;Malgrandaviricetes;Petitvirales;Microviridae;Microviridae__na;Dipodfec_virus_UOA04_R
od_753"0 0 0 0 1 0 0 0 0 0 0 0 0
0 0 0 0 0 0 0 0 0 0 0 0 0
0 0 0 0 0 0 0 0 0 0 0 0 0
0 0 0 0 0 0 0 0 0 0 0 0 0
0 0 0 0 0 0 0 0 0 0 0 0 0
0 0 0 0 0 0 0
"Viruses;Phixviricota;Malgrandaviricetes;Petitvirales;Microviridae;Microviridae__na;Dipodfec_virus_UOA04_R
od_861"0 0 0 0 4 0 0 0 0 0 0 0 0
0 0 0 0 0 0 0 0 0 0 0 0 0
0 0 0 0 0 0 0 0 0 0 0 0 0
0 0 0 0 0 0 0 0 6 0 0 0 0
0 0 0 0 0 0 0 0 0 0 0 0 0
0 0 0 0 0 0 0
"Viruses;Phixviricota;Malgrandaviricetes;Petitvirales;Microviridae;Microviridae__na;Eel_River_basin_pequenov
irus" 0 0 0 2 0 12 0 0 0 0 0 0 0
0 0 0 0 1 0 0 0 0 0 0 0 0
0 0 0 0 0 0 0 0 0 0 0 0 0
0 0 0 0 0 0 0 0 0 0 0 0 0

0 0 0 0 0 0 0 0 0 0 0 0 0
0 0 0 0 0 0 0
"Viruses;Phixviricota;Malgrandaviricetes;Petitvirales;Microviridae;Microviridae__na;Flumine_microvirus_5"
0 0 0 0 0 0 0 0 0 0 0 0 0
0 0 0 0 0 0 0 0 0 0 0 0 0
0 0 0 0 0 0 0 0 0 0 0 0 0
0 0 0 0 0 0 0 0 1 0 0 0 0
0 0 0 0 0 0 0 0 0 0 0 0 0
0 0 0 0 0 0 0
"Viruses;Phixviricota;Malgrandaviricetes;Petitvirales;Microviridae;Microviridae__na;Flyfo_microvirus_Tbat2_11
6" 0 0 0 0 1 0 0 0 0 0 0 0 0
0 0 0 0 0 0 0 0 0 0 0 0 0
0 0 0 0 0 0 0 0 0 0 0 0 0
0 0 0 0 0 0 0 0 2 0 0 0 0
0 0 0 0 0 0 0 0 0 0 0 0 0
0 0 0 0 0 0 0
"Viruses;Phixviricota;Malgrandaviricetes;Petitvirales;Microviridae;Microviridae__na;Flyfo_microvirus_Tbat2_13
0" 0 0 0 0 6 0 0 0 0 0 0 0 0
0 0 0 0 0 0 0 0 0 0 0 0 0
0 0 0 0 0 0 0 0 0 0 0 0 0
0 0 0 0 0 0 0 0 0 0 0 0 0
0 0 0 0 0 0 0 0 0 0 0 0 0
0 0 0 0 0 0 0
"Viruses;Phixviricota;Malgrandaviricetes;Petitvirales;Microviridae;Microviridae__na;Gokushovirinae_Bog5712_
52" 0 0 0 2 7 5 0 0 0 0 0 0 0
0 0 0 0 0 0 0 0 0 0 0 0 0
0 0 0 0 0 0 0 0 0 0 0 0 0
0 0 0 0 0 0 0 0 0 0 0 0 0
0 0 0 0 0 0 0 0 0 0 0 0 0
0 0 0 0 0 0 0
"Viruses;Phixviricota;Malgrandaviricetes;Petitvirales;Microviridae;Microviridae__na;Gokushovirinae_environm
ental_samples" 0 0 1 9 71 54 0 0 0 0 0 0
0 0 0 0 1 0 0 0 0 0 1 0 0
0 0 0 0 0 0 0 0 0 0 0 0 0
0 0 1 1 0 0 0 0 0 23 0 0 0
2 0 0 0 0 0 0 0 0 0 0 0 0
0 0 0 0 0 0 2 0
"Viruses;Phixviricota;Malgrandaviricetes;Petitvirales;Microviridae;Microviridae__na;Gokushovirinae_Fen672_3
1" 0 0 0 0 2 0 0 0 0 0 0 0 0
0 0 0 0 0 0 0 0 0 0 0 0 0
0 0 0 0 0 0 0 0 0 0 0 0 0
0 0 0 0 0 0 0 0 0 0 0 0 0
0 0 0 0 0 0 0 0 0 0 0 0 0
0 0 0 0 0 0 0
"Viruses;Phixviricota;Malgrandaviricetes;Petitvirales;Microviridae;Microviridae__na;Gokushovirinae_GAIR4"
0 0 0 0 0 1 0 0 0 0 0 0 0
0 0 0 0 0 0 0 0 0 0 0 0 0
0 0 0 0 0 0 0 0 0 0 0 0 0
0 0 0 0 0 0 0 0 0 0 0 0 0

0 0 0 0 0 0 0 0 0 0 0 0 0
0 0 0 0 0 0 0
"Viruses;Phixviricota;Malgrandaviricetes;Petitvirales;Microviridae;Microviridae__na;Gokushovirinae_GNX3R"
0 0 0 1 2 14 0 0 0 0 0 0 0
0 0 0 0 0 0 0 0 0 5 0 0 0
0 0 0 0 0 0 0 0 0 0 0 0 0
0 0 0 0 0 0 0 0 0 0 0 0 0
0 0 0 0 0 0 0 0 0 0 0 0 0
0 0 0 0 0 1 0
"Viruses;Phixviricota;Malgrandaviricetes;Petitvirales;Microviridae;Microviridae__na;Gokushovirinae_sp."
0 0 0 2 0 7 0 0 0 0 0 0 0
0 18 15 97 0 0 0 0 0 1 0 0 0
1 0 0 0 0 0 0 0 0 1 0 0 0
0 0 0 0 0 3 1 0 5 0 0 0 0
1 128 1 0 0 0 0 0 0 0 0 0 0
0 0 0 4 4 0 0
"Viruses;Phixviricota;Malgrandaviricetes;Petitvirales;Microviridae;Microviridae__na;Gokushovirus_WZ-2015a"
0 0 0 3 5 16 0 0 1 0 0 0 1
0 10 11 25 1 1 0 0 0 2 0 5 1
865 181 293 350 0 0 0 0 1 2 1 0 0
0 1 14 0 0 2 0 0 1 0 0 0 0
2 938 0 2 0 0 0 0 0 0 0 0 0
0 0 0 17 9 0 0
"Viruses;Phixviricota;Malgrandaviricetes;Petitvirales;Microviridae;Microviridae__na;Human_gut_gokushovirus
" 0 0 0 0 7 9 0 0 0 0 0 0 0
0 1 0 1 0 0 0 0 0 0 0 0 0
1 0 0 3 0 0 0 0 0 0 0 0 0
0 0 0 0 0 0 0 0 0 0 0 0 0
0 190 0 0 0 0 0 0 0 0 0 0 0
0 0 0 5 2 0 0
"Viruses;Phixviricota;Malgrandaviricetes;Petitvirales;Microviridae;Microviridae__na;Human_gut_microviridae_
SH-CHD12" 0 0 0 0 0 0 0 0 0 0 0 0
0 0 12 19 42 0 0 0 0 0 0 0 0
0 0 0 0 0 0 0 0 0 0 0 0 0
0 0 0 0 0 0 0 0 0 0 0 0 0
0 0 0 0 0 0 0 0 0 0 0 0 0
0 0 0 0 0 0 0 0
"Viruses;Phixviricota;Malgrandaviricetes;Petitvirales;Microviridae;Microviridae__na;Kummerowia_striata_goku
shovirus" 0 0 1 2 32 2 0 0 0 0 0 0
0 0 0 0 0 0 0 0 0 0 0 0 0
0 0 0 0 0 0 0 0 0 0 0 0 0
0 0 1 0 0 0 0 0 0 1 0 0 0
0 0 0 0 0 0 0 0 0 0 0 0 0
0 0 0 0 0 0 0 0
"Viruses;Phixviricota;Malgrandaviricetes;Petitvirales;Microviridae;Microviridae__na;Marine_gokushovirus"
0 0 0 1 28 0 0 0 0 0 0 0 0
0 0 0 0 0 0 0 0 0 0 0 0 0
0 0 0 0 0 0 0 0 0 0 0 0 0
0 0 0 0 0 0 0 0 3 0 0 0 0

0 0 0 0 0 0 0 0 0 0 0 0 0
0 0 0 0 0 0 0
"Viruses;Phixviricota;Malgrandaviricetes;Petitvirales;Microviridae;Microviridae__na;Microviridae_Bog9017_22"
0 0 0 0 7 3 0 0 0 0 0 0 0
0 0 0 0 0 0 0 0 0 0 0 0 0
0 0 0 0 0 0 0 0 0 0 0 0 0
0 0 0 0 0 0 0 0 2 0 0 0 0
0 0 0 0 0 0 0 0 0 0 0 0 0
0 0 0 0 0 0 0
"Viruses;Phixviricota;Malgrandaviricetes;Petitvirales;Microviridae;Microviridae__na;Microviridae_sp." 1
0 156 332 4593 2684 0 1 28 3 0 0 43 52
5298 6260 11427 721 25 14 563 336 1122 1788 77 56 44257
9179 16352 17967 45 41 25 4 17355 231 70 22 291 94
14 2938 10 5 58 7 167 718 39 22 6593 637 1322
9619 46 78 0 1 3832 1885 68 18 240 462 65 81
54 53 244 143 104181 1
"Viruses;Phixviricota;Malgrandaviricetes;Petitvirales;Microviridae;Microviridae__na;Microviridae_sp._ct08i1"
0 0 0 0 0 0 0 0 0 0 0 0 0
0 0 0 0 0 0 0 0 0 0 0 0 0
0 0 0 0 0 0 0 0 0 0 0 0 0
0 0 0 0 0 0 0 0 0 0 0 0 0
1 0 0 0 0 0 0 0 0 0 0 0 0
0 0 0 0 0 0 0
"Viruses;Phixviricota;Malgrandaviricetes;Petitvirales;Microviridae;Microviridae__na;Microviridae_sp._ct0DW36
" 0 0 0 0 1 0 0 0 0 0 0 0 0
0 0 0 0 0 0 0 0 0 0 0 0 0
0 0 0 0 0 0 0 0 0 0 0 0 0
0 0 0 0 0 0 0 0 0 0 0 0 0
0 0 0 0 0 0 0 0 0 0 0 0 0
0 0 0 0 0 0 0
"Viruses;Phixviricota;Malgrandaviricetes;Petitvirales;Microviridae;Microviridae__na;Microviridae_sp._ct13s5"
0 0 0 0 0 0 0 0 0 0 0 0 0
0 0 0 0 0 1 1 0 0 0 0 1 0
0 0 0 0 0 1 0 0 1 0 0 0 0
0 0 0 0 0 0 0 0 0 0 2 0 0
0 0 0 2 0 0 0 0 1 0 0 0 0
0 0 0 0 0 0 0
"Viruses;Phixviricota;Malgrandaviricetes;Petitvirales;Microviridae;Microviridae__na;Microviridae_sp._ct2OM3"
0 0 0 0 26 0 0 0 0 0 0 0 0
0 0 0 0 0 0 0 0 0 0 0 0 0
0 0 0 0 0 0 0 0 0 0 0 0 0
0 0 0 0 0 0 0 0 1 0 0 0 0
0 0 0 0 0 0 0 0 0 0 0 0 0
0 0 0 0 0 0 0
"Viruses;Phixviricota;Malgrandaviricetes;Petitvirales;Microviridae;Microviridae__na;Microviridae_sp._ct2x44"
0 0 0 0 0 0 0 0 0 0 0 0 0
0 0 0 0 0 0 0 0 0 0 0 0 0
4 0 0 0 0 0 0 0 0 0 0 0 0
0 0 0 0 0 0 0 0 0 0 0 0 0

0 0 0 0 0 0 0 0 5 1 2 8 0
0 0 0 0 0 0 0
"Viruses;Phixviricota;Malgrandaviricetes;Petitvirales;Microviridae;Microviridae__na;Microviridae_sp._ct4S516"
0 0 0 0 0 0 0 0 0 0 0 0 0
2 1 0 2 0 0 0 0 0 0 0 0 0
0 0 0 0 0 0 0 0 0 0 0 0 0
0 0 0 0 0 0 0 0 0 0 0 0 0
0 0 0 0 0 0 0 0 0 0 0 0 0
0 0 0 0 0 0 0
"Viruses;Phixviricota;Malgrandaviricetes;Petitvirales;Microviridae;Microviridae__na;Microviridae_sp._ct9ZF1"
0 0 0 0 0 0 0 0 0 0 0 0 0
0 0 0 0 0 0 0 0 0 0 0 0 0
0 0 0 0 0 0 0 0 0 0 0 0 1
0 0 0 0 0 0 0 0 0 0 0 0 0
1 0 0 0 0 0 0 0 0 0 0 0 0
0 0 0 0 0 0 0
"Viruses;Phixviricota;Malgrandaviricetes;Petitvirales;Microviridae;Microviridae__na;Microviridae_sp._ctbuH30"
0 0 0 0 5 0 0 0 0 0 0 0 0
0 0 0 0 0 0 0 0 0 0 0 0 0
0 0 0 0 0 0 0 0 0 0 0 0 0
0 0 0 0 0 0 0 0 0 0 0 0 0
0 0 0 0 0 0 0 0 0 0 0 0 0
0 0 0 0 0 0 0
"Viruses;Phixviricota;Malgrandaviricetes;Petitvirales;Microviridae;Microviridae__na;Microviridae_sp._ctbyf5"
0 0 0 0 0 0 0 0 0 0 0 0 1
0 0 0 0 0 2 1 0 0 0 0 0 0
0 0 0 0 0 0 0 0 0 0 0 0 0
0 0 0 1 3 0 0 0 0 1 0 0 0
0 0 1 0 0 0 0 0 5 1 0 0 0
0 1 1 0 0 0 0
"Viruses;Phixviricota;Malgrandaviricetes;Petitvirales;Microviridae;Microviridae__na;Microviridae_sp._ctCVC7"
0 0 0 0 0 0 0 0 0 0 0 0 0
0 0 0 0 0 0 0 0 0 0 0 0 0
0 0 0 0 0 0 0 0 0 0 0 1 0
0 0 0 0 0 0 0 0 0 0 0 0 0
0 0 0 0 0 0 0 0 0 0 0 0 0
0 0 0 0 0 0 0
"Viruses;Phixviricota;Malgrandaviricetes;Petitvirales;Microviridae;Microviridae__na;Microviridae_sp._ctD0m35
" 0 0 0 0 1 0 0 0 0 0 0 0 0
0 0 0 0 0 0 0 0 0 0 0 0 0
0 0 0 0 0 0 0 0 0 0 0 0 0
0 0 0 0 0 0 0 0 0 0 0 0 0
0 0 0 0 0 0 0 0 0 0 0 0 0
0 0 0 0 0 0 0
"Viruses;Phixviricota;Malgrandaviricetes;Petitvirales;Microviridae;Microviridae__na;Microviridae_sp._ctdfd8"
0 0 0 0 0 0 0 0 0 0 0 0 0
0 0 0 0 0 0 0 0 0 0 0 0 0
0 0 0 0 0 1 0 0 0 0 0 0 0
0 0 0 0 0 0 0 0 0 0 0 0 0

0 0 0 0 0 0 0 0 0 0 0 0 0
0 0 0 0 0 0 0
"Viruses;Phixviricota;Malgrandaviricetes;Petitvirales;Microviridae;Microviridae__na;Microviridae_sp._ctemt10"
0 0 0 0 0 0 0 0 0 0 0 0 0
0 0 0 0 0 0 0 0 0 0 0 0 0
0 0 0 0 0 0 0 0 0 0 0 0 0
0 0 0 0 0 3 2 0 0 0 0 0 0
0 0 0 0 0 0 0 0 0 0 0 0 0
0 0 0 0 0 0 0
"Viruses;Phixviricota;Malgrandaviricetes;Petitvirales;Microviridae;Microviridae__na;Microviridae_sp._ctFGM2"
0 0 0 0 0 0 0 0 0 0 0 0 0
0 0 0 0 0 0 0 0 0 0 0 0 0
0 0 0 0 0 0 0 0 0 0 1 0 2
1 0 0 0 0 0 0 0 0 0 0 0 0
2 0 0 0 0 0 0 0 0 0 0 0 0
0 0 0 0 0 0 0
"Viruses;Phixviricota;Malgrandaviricetes;Petitvirales;Microviridae;Microviridae__na;Microviridae_sp._ctfUi1"
0 0 2 0 0 0 0 0 0 0 0 0 0
0 0 0 0 0 0 0 9 4 7 7 0 0
0 0 0 0 0 0 0 0 0 0 0 0 0
0 0 0 0 0 0 0 0 0 0 0 0 0
0 18 0 0 0 0 0 0 0 0 0 0 0
0 0 0 0 0 0 0
"Viruses;Phixviricota;Malgrandaviricetes;Petitvirales;Microviridae;Microviridae__na;Microviridae_sp._ctHN216
" 0 0 0 0 0 0 0 0 0 0 0 0 0
0 0 0 0 0 0 0 0 0 0 0 0 0
0 0 0 0 0 0 0 0 0 0 1 1 0
0 0 0 0 0 0 0 0 0 0 0 0 0
0 0 0 0 0 0 0 0 0 0 0 0 0
0 0 0 0 0 0 0
"Viruses;Phixviricota;Malgrandaviricetes;Petitvirales;Microviridae;Microviridae__na;Microviridae_sp._ctITQ3"
0 0 0 0 0 2 0 0 0 0 0 0 0
0 0 0 0 0 0 0 0 0 0 0 0 0
0 0 0 0 0 0 0 0 0 0 0 0 0
0 0 0 0 0 0 0 0 0 0 0 0 0
0 0 0 0 0 0 0 0 0 0 0 0 0
0 0 0 0 0 0 0
"Viruses;Phixviricota;Malgrandaviricetes;Petitvirales;Microviridae;Microviridae__na;Microviridae_sp._ctJby12"
0 0 0 0 0 0 0 0 0 0 0 0 0
0 1 3 4 0 0 0 1 0 0 0 0 0
0 0 0 0 0 0 0 0 0 0 0 0 0
0 0 0 0 0 0 0 0 0 0 0 0 0
0 0 0 0 0 0 0 0 0 0 0 0 0
0 0 0 0 0 0 0
"Viruses;Phixviricota;Malgrandaviricetes;Petitvirales;Microviridae;Microviridae__na;Microviridae_sp._ctJKB8"
0 0 0 0 0 0 0 0 0 0 0 0 0
0 0 1 2 3 0 0 0 0 0 0 0 0
1 0 0 0 0 0 0 0 0 0 0 0 0
0 0 0 0 0 0 0 0 0 0 0 0 0

0 0 0 0 0 0 0 0 0 0 0 0 0
0 0 0 0 0 0 0

"Viruses;Phixviricota;Malgrandaviricetes;Petitvirales;Microviridae;Microviridae__na;Microviridae_sp._ctkQD1"
0 0 0 0 7 16 0 0 0 0 0 0 0
0 0 0 0 0 0 0 0 0 0 2 0 0
0 0 0 0 0 0 0 0 0 0 0 0 0
0 0 0 0 0 1 0 0 2 0 0 0 0
0 0 0 0 0 0 0 0 0 0 0 0 0
0 0 0 0 0 2 0

"Viruses;Phixviricota;Malgrandaviricetes;Petitvirales;Microviridae;Microviridae__na;Microviridae_sp._ctKRd3"
0 0 0 0 0 0 0 0 0 0 0 0 0
0 6 7 4 0 0 0 0 0 1 2 0 0
1 0 0 0 0 0 0 0 0 0 0 0 0
0 0 0 0 0 0 0 0 0 0 0 0 0
0 0 0 0 0 0 0 0 1 0 2 5 1
0 0 0 0 0 0 0

"Viruses;Phixviricota;Malgrandaviricetes;Petitvirales;Microviridae;Microviridae__na;Microviridae_sp._ctlXB4"
0 0 0 0 0 0 0 0 0 0 0 0 0
0 0 0 0 0 0 0 0 0 0 0 0 0
0 0 0 0 0 0 0 0 0 0 0 0 0
0 0 0 0 0 0 0 0 0 0 0 0 0
1 0 0 0 0 0 0 0 0 0 0 0 0
0 0 0 0 0 0 0

"Viruses;Phixviricota;Malgrandaviricetes;Petitvirales;Microviridae;Microviridae__na;Microviridae_sp._ctMIi2"
0 0 2 0 0 0 0 0 0 0 0 0 0
0 0 0 0 0 0 0 0 0 0 0 0 0
0 0 0 0 0 0 0 0 0 0 0 0 0
0 0 0 0 0 0 0 0 0 0 0 0 0
0 0 0 0 0 0 0 0 0 0 0 0 0
0 0 0 0 0 0 0

"Viruses;Phixviricota;Malgrandaviricetes;Petitvirales;Microviridae;Microviridae__na;Microviridae_sp._ctNWS1"
0 0 0 0 0 0 0 0 0 0 0 0 3
3 1 2 1 4 0 0 0 0 0 0 0 0
0 0 0 0 0 0 0 0 0 0 0 0 0
0 0 0 0 0 0 0 0 0 0 0 0 0
0 0 0 0 0 0 0 0 0 1 0 0 0
0 3 1 0 0 0 0

"Viruses;Phixviricota;Malgrandaviricetes;Petitvirales;Microviridae;Microviridae__na;Microviridae_sp._ctOkR17
" 0 0 0 0 0 0 0 0 0 0 0 0 0
0 0 0 0 0 0 0 0 0 0 0 0 0
0 0 0 0 0 0 0 0 0 0 0 0 0
0 0 0 0 0 0 0 0 0 0 0 0 0
0 0 0 0 0 0 0 0 0 0 0 1 0
0 0 0 0 0 0 0

"Viruses;Phixviricota;Malgrandaviricetes;Petitvirales;Microviridae;Microviridae__na;Microviridae_sp._ctOsc38"
0 0 0 2 0 5 0 0 0 0 0 0 0
0 0 0 0 0 0 0 0 0 0 0 0 0
0 0 0 0 0 0 0 0 0 0 0 0 1
0 0 0 0 0 0 0 0 1 0 0 0 0

0 0 0 0 0 0 0 0 0 0 0 0 0
0 0 0 0 0 0 0
"Viruses;Phixviricota;Malgrandaviricetes;Petitvirales;Microviridae;Microviridae__na;Microviridae_sp._ctrJ69"
0 0 0 1 0 0 0 0 0 0 0 0 0
0 0 0 0 0 0 0 0 0 0 0 0 0
0 0 0 0 0 0 0 0 0 0 0 0 0
0 0 0 0 0 0 0 0 1 0 0 0 0
0 0 0 0 0 0 0 0 0 0 0 0 0
0 0 0 0 0 0 0
"Viruses;Phixviricota;Malgrandaviricetes;Petitvirales;Microviridae;Microviridae__na;Microviridae_sp._ctUND6"
0 0 0 0 0 0 0 0 0 0 0 0 0
0 25 22 3 0 0 0 4 3 1 4 0 0
0 0 0 0 0 0 0 0 0 0 0 0 0
0 0 0 0 0 0 0 0 0 0 0 0 0
0 0 0 0 0 0 0 0 0 0 1 2 0
0 0 1 0 0 0 0
"Viruses;Phixviricota;Malgrandaviricetes;Petitvirales;Microviridae;Microviridae__na;Microviridae_sp._ctuZ46"
0 0 0 0 0 0 0 0 0 0 0 0 0
0 0 0 0 0 0 0 0 0 0 1 0 0
0 0 0 0 0 0 0 0 0 0 0 0 0
0 0 0 0 0 0 0 0 0 0 0 0 0
0 0 0 0 0 0 0 0 0 0 0 0 0
0 0 0 0 0 0 0
"Viruses;Phixviricota;Malgrandaviricetes;Petitvirales;Microviridae;Microviridae__na;Microviridae_sp._ctwzP10"
0 0 0 0 0 0 0 0 0 0 0 0 0
0 5 7 18 0 0 0 0 0 1 0 0 0
0 0 0 0 0 0 0 0 0 0 0 0 0
0 0 0 0 1 0 0 0 0 0 0 0 0
0 0 0 0 0 0 0 0 0 0 0 0 0
0 0 0 0 0 0 0
"Viruses;Phixviricota;Malgrandaviricetes;Petitvirales;Microviridae;Microviridae__na;Microviridae_sp._ctX0F7"
0 0 0 0 0 0 0 0 0 0 0 0 0
0 0 0 0 8 0 0 0 0 0 0 0 0
3 0 3 0 0 0 0 0 0 0 0 0 0
0 0 0 0 0 0 0 0 0 0 0 0 0
0 0 2 3 0 0 0 0 0 0 0 0 0
0 1 1 0 0 0 0
"Viruses;Phixviricota;Malgrandaviricetes;Petitvirales;Microviridae;Microviridae__na;Microviridae_sp._ctX401"
0 0 0 0 0 0 0 0 0 0 0 0 0
0 0 0 1 1 0 0 0 0 0 0 0 0
0 0 0 0 0 0 0 0 0 0 0 0 0
0 0 0 0 0 0 0 0 0 0 0 0 0
0 0 0 0 0 0 0 0 0 0 0 0 0
0 0 0 0 0 0 0
"Viruses;Phixviricota;Malgrandaviricetes;Petitvirales;Microviridae;Microviridae__na;Microviridae_sp._ctydc4"
0 0 0 0 3 0 0 0 0 0 0 0 0
0 0 0 0 0 0 0 0 0 0 0 0 0
0 0 0 0 0 0 0 0 0 0 0 0 0
0 0 0 0 0 0 0 0 0 0 0 0 0

0 0 0 0 0 0 0 0 0 0 0 0 0
0 0 0 0 0 0 0
"Viruses;Phixviricota;Malgrandaviricetes;Petitvirales;Microviridae;Microviridae__na;Microviridae_sp._ctzsU3"
0 0 0 0 4 0 0 0 0 0 0 0 0
0 0 0 0 0 0 0 0 0 0 0 0 0
0 0 0 0 0 0 0 0 0 0 0 0 0
0 0 0 0 0 0 0 0 0 0 0 0 0
0 0 0 0 0 0 0 0 0 0 0 0 0
0 0 0 0 0 0 0
"Viruses;Phixviricota;Malgrandaviricetes;Petitvirales;Microviridae;Microviridae__na;Microvirus_AZ-2020"
0 0 0 0 15 0 0 0 0 0 0 0 0
0 0 0 0 0 0 0 0 0 0 0 0 0
0 0 0 0 0 0 0 0 9 0 0 0 0
0 0 0 0 0 0 0 0 0 0 0 0 0
0 0 0 0 0 0 0 0 0 0 0 0 0
0 0 0 0 0 0 0
"Viruses;Phixviricota;Malgrandaviricetes;Petitvirales;Microviridae;Microviridae__na;Microvirus_mar20" 0
0 0 0 1 0 0 0 0 0 0 0 0 0
0 0 0 0 0 0 0 0 0 0 0 0 0
0 0 0 0 0 0 0 0 0 0 0 0 0
0 0 0 0 0 0 0 0 0 0 0 0 0
0 0 0 0 0 0 0 0 0 0 0 0 0
0 0 0 0 0 0
"Viruses;Phixviricota;Malgrandaviricetes;Petitvirales;Microviridae;Microviridae__na;Microvirus_mar28" 0
0 0 0 1 0 0 0 0 0 0 0 0 0
0 0 0 0 0 0 0 0 0 0 0 0 0
0 0 0 0 0 0 0 0 0 0 0 0 0
0 0 0 0 0 0 0 0 0 0 0 0 0
0 0 0 0 0 0 0 0 0 0 0 0 0
0 0 0 0 0 0
"Viruses;Phixviricota;Malgrandaviricetes;Petitvirales;Microviridae;Microviridae__na;Microvirus_mar30" 0
0 0 0 1 0 0 0 0 0 0 0 0 0
0 0 0 0 0 0 0 0 0 0 0 0 0
0 0 0 0 0 0 0 0 0 0 0 0 0
1 0 0 0 0 0 0 1 0 0 0 0 0
0 0 0 0 0 0 0 0 0 0 0 0 0
0 0 0 0 0 0
"Viruses;Phixviricota;Malgrandaviricetes;Petitvirales;Microviridae;Microviridae__na;Microvirus_mar40" 0
0 0 0 0 0 0 0 0 0 0 0 0 0
23 15 155 0 0 0 0 0 5 0 0 0 0
0 0 0 0 0 0 0 0 0 0 0 0 0
0 0 0 0 0 0 0 0 0 0 0 0 0
0 0 0 0 0 0 0 0 0 0 0 0 0
0 0 0 0 0 0
"Viruses;Phixviricota;Malgrandaviricetes;Petitvirales;Microviridae;Microviridae__na;Microvirus_mar45" 0
0 0 0 0 0 0 0 0 0 0 0 0 0
16 27 208 0 0 0 0 0 2 0 0 0 0
0 0 0 0 0 0 0 0 0 0 0 0 0
0 0 0 0 0 0 0 0 0 0 0 0 0

0 0 0 0 0 0 0 0 0 0 0 0 0
0 0 0 0 0 0
"Viruses;Phixviricota;Malgrandaviricetes;Petitvirales;Microviridae;Microviridae__na;Microvirus_mar49" 0
0 0 0 0 0 0 0 0 0 0 0 0 0
24 16 200 0 0 0 0 0 6 0 0 0 0
0 0 0 0 0 0 0 0 0 0 0 0 0
0 0 0 0 0 0 0 0 0 0 0 0 1
0 0 0 0 0 0 0 0 0 0 0 0 0
0 0 0 0 0 0
"Viruses;Phixviricota;Malgrandaviricetes;Petitvirales;Microviridae;Microviridae__na;Microvirus_mar52" 0
0 0 0 0 0 0 0 0 0 0 0 0 0
1 0 0 0 0 0 0 0 0 0 0 0 0
0 0 0 0 0 0 0 0 0 0 0 0 0
0 0 0 0 0 0 0 0 0 0 0 0 0
0 0 0 0 0 0 0 0 0 0 0 0 0
0 0 0 0 0 0
"Viruses;Phixviricota;Malgrandaviricetes;Petitvirales;Microviridae;Microviridae__na;Microvirus_mar56" 0
0 0 0 0 0 0 0 0 0 0 0 0 0
0 0 0 0 0 0 2 0 3 0 0 0 0
0 0 0 0 0 0 0 0 0 0 0 0 0
0 0 0 0 0 0 0 0 0 0 0 0 0
0 0 0 0 0 0 0 0 0 0 0 0 0
0 0 0 0 0 0
"Viruses;Phixviricota;Malgrandaviricetes;Petitvirales;Microviridae;Microviridae__na;Microvirus_mar60" 0
0 0 0 0 0 0 0 0 0 0 0 0 0
0 0 1 0 0 0 0 0 0 0 0 0 0
0 0 0 0 0 0 0 0 0 0 0 0 0
0 0 0 0 0 0 0 0 0 0 0 0 0
0 0 0 0 0 0 0 0 0 0 0 0 0
0 0 0 0 0 0
"Viruses;Phixviricota;Malgrandaviricetes;Petitvirales;Microviridae;Microviridae__na;Microvirus_Sku115" 0
0 0 0 1 0 0 0 0 0 0 0 0 0
0 0 0 0 0 0 0 0 0 0 0 0 0
0 0 0 0 0 0 0 0 0 0 0 0 0
0 0 0 0 0 0 0 0 0 0 0 0 0
0 0 0 0 0 0 0 0 0 0 0 0 0
0 0 0 0 0 0
"Viruses;Phixviricota;Malgrandaviricetes;Petitvirales;Microviridae;Microviridae__na;Microvirus_sp." 0
0 79 248 2975 1983 0 0 0 0 0 0 0 0
114 137 224 18 0 0 20 17 31 54 0 3 739
167 220 162 0 0 0 0 434 8 0 0 2 0
4 28 0 0 5 2 0 495 0 0 4 3 24
2256 0 0 0 0 0 0 0 1 2 2 0 0
2 4 47 23 121 0
"Viruses;Phixviricota;Malgrandaviricetes;Petitvirales;Microviridae;Microviridae__na;Peromfec_virus_RodF5_17
" 0 0 0 0 0 0 0 0 0 0 0 0 0
0 0 0 0 0 0 0 0 0 0 0 0 0
0 0 0 0 0 0 0 0 0 0 0 0 0
0 0 0 0 0 0 0 0 0 0 0 0 0

0 0 0 0 3 0 0 0 0 0 0 0 0
0 0 0 0 0 0 0
"Viruses;Phixviricota;Malgrandaviricetes;Petitvirales;Microviridae;Microviridae__na;Peromfec_virus_RodF7_15
" 0 0 0 0 1 0 0 0 0 0 0 0 0
0 0 0 0 0 0 0 0 0 0 0 0 0
0 0 0 0 0 0 0 0 0 0 0 0 0
0 0 0 0 0 0 0 0 0 0 0 0 0
0 0 0 0 0 0 0 0 0 0 0 0 0
0 0 0 0 0 0 0
"Viruses;Phixviricota;Malgrandaviricetes;Petitvirales;Microviridae;Microviridae__na;Peromfec_virus_RodF7_16
" 0 0 0 1 0 2 0 0 0 0 0 0 0
0 0 0 0 0 0 0 0 0 0 0 0 0
0 0 0 0 0 0 0 0 0 0 0 0 0
0 0 0 0 0 0 0 0 1 0 0 0 0
0 0 0 0 0 0 0 0 0 0 0 0 0
0 0 0 0 0 0 0
"Viruses;Phixviricota;Malgrandaviricetes;Petitvirales;Microviridae;Microviridae__na;Peromfec_virus_RodF7_9"
0 0 0 0 0 0 0 0 0 0 0 0 0
0 2 3 0 0 0 0 0 0 0 0 0 0
0 0 0 0 0 0 0 0 0 0 0 0 0
0 0 0 0 0 0 0 0 0 0 0 0 0
0 0 0 0 0 0 0 0 0 0 0 0 0
0 0 0 0 0 0 0
"Viruses;Phixviricota;Malgrandaviricetes;Petitvirales;Microviridae;Microviridae__na;Peromfec_virus_RodF8_14
" 0 0 0 0 0 0 0 0 0 0 0 0 0
0 1 0 0 0 0 0 0 0 0 0 0 0
0 0 0 0 0 0 0 0 0 0 0 0 0
0 0 0 0 0 0 0 0 0 0 0 0 0
0 0 0 0 0 0 0 0 0 0 0 0 0
0 0 0 0 0 0 0
"Viruses;Phixviricota;Malgrandaviricetes;Petitvirales;Microviridae;Microviridae__na;Peromfec_virus_RodF8_19
" 0 0 0 0 0 0 0 0 0 0 0 0 0
0 0 0 4 0 0 0 0 0 0 0 0 0
0 0 0 0 0 0 0 0 0 0 0 0 0
0 0 0 0 0 0 0 0 0 0 0 0 0
0 0 0 0 0 0 0 0 0 0 0 0 0
0 0 0 0 0 1 0
"Viruses;Phixviricota;Malgrandaviricetes;Petitvirales;Microviridae;Microviridae__na;Peromfec_virus_RodF8_21
" 0 0 0 0 0 0 0 0 0 0 0 0 0
0 3 2 0 0 0 0 0 0 0 0 0 0
0 0 0 0 0 0 0 0 0 0 0 0 0
0 0 0 0 0 0 0 0 0 0 0 0 0
0 0 0 0 0 0 0 0 0 0 0 0 0
0 0 0 0 0 0 0
"Viruses;Phixviricota;Malgrandaviricetes;Petitvirales;Microviridae;Microviridae__na;Peromfec_virus_RodF8_33
" 0 0 0 0 0 0 0 0 0 0 0 0 0
0 1 0 0 0 0 0 0 0 0 0 0 0
0 0 0 0 0 0 0 0 0 0 0 0 0
0 0 0 0 0 0 0 0 0 0 0 0 0

0 0 0 0 0 0 0 0 0 0 0 0 0
0 0 0 0 0 0 0
"Viruses;Phixviricota;Malgrandaviricetes;Petitvirales;Microviridae;Microviridae__na;Peromfec_virus_RodF8_36
" 0 0 0 0 1 0 0 0 0 0 0 0 0
0 0 0 0 0 0 0 0 0 0 0 0 0
0 0 0 0 0 0 0 0 0 0 0 0 0
0 0 0 0 0 0 0 0 3 0 0 0 0
0 0 0 0 0 0 0 0 0 0 0 0 0
0 0 0 0 0 0 0
"Viruses;Phixviricota;Malgrandaviricetes;Petitvirales;Microviridae;Microviridae__na;Peromfec_virus_RodF8_38
" 0 0 0 0 0 0 0 0 0 0 0 0 0
0 0 0 0 0 0 0 0 0 0 0 0 0
0 0 0 0 0 0 0 0 0 0 0 0 0
0 0 1 0 0 0 0 0 0 0 0 0 0
1 0 0 0 0 0 0 0 0 0 0 0 0
0 0 0 0 0 0 0
"Viruses;Phixviricota;Malgrandaviricetes;Petitvirales;Microviridae;Microviridae__na;Poophage_MBI-2016a"
0 0 0 0 0 0 0 0 0 0 0 0 0
0 9 16 84 0 0 0 0 0 1 0 0 0
0 0 0 0 0 0 0 0 0 0 0 0 0
0 0 0 0 0 0 0 0 0 0 0 0 0
0 0 0 0 0 0 0 0 0 0 0 0 0
0 0 0 0 0 0 0
"Viruses;Phixviricota;Malgrandaviricetes;Petitvirales;Microviridae;Microviridae__na;Poophage_SC_4_H6H8_20
17" 0 0 0 0 0 0 0 0 0 0 0 0 0
0 3 7 17 0 0 0 0 0 0 0 0 0
0 0 0 0 0 0 0 0 0 0 0 0 0
0 0 0 0 0 0 0 0 0 0 0 0 0
1 0 0 0 0 0 0 0 0 0 0 0 0
0 0 0 0 0 0 0
"Viruses;Phixviricota;Malgrandaviricetes;Petitvirales;Microviridae;Microviridae__na;robinz_microvirus_RP_103
" 0 0 0 0 6 0 0 0 0 0 0 0 0
0 0 0 0 0 0 0 0 0 0 0 0 0
0 0 0 0 0 0 0 0 0 0 0 0 0
0 0 0 0 0 0 0 0 0 0 0 0 0
0 0 0 0 0 0 0 0 0 0 0 0 0
0 0 0 0 0 0 0
"Viruses;Phixviricota;Malgrandaviricetes;Petitvirales;Microviridae;Microviridae__na;robinz_microvirus_RP_105
" 0 0 0 0 0 0 0 0 0 0 0 0 0
0 0 0 0 0 0 0 0 0 0 0 0 0
0 0 0 0 0 0 0 0 0 0 0 0 0
0 0 0 0 0 0 0 0 1 0 0 0 0
0 0 0 0 0 0 0 0 0 0 0 0 0
0 0 0 0 0 0 0
"Viruses;Phixviricota;Malgrandaviricetes;Petitvirales;Microviridae;Microviridae__na;robinz_microvirus_RP_111
" 0 0 0 0 18 0 0 0 0 0 0 0 0
0 0 0 0 0 0 0 0 0 0 0 0 0
0 0 0 0 0 0 0 0 0 0 0 0 0
0 0 0 0 0 1 0 0 0 0 0 0 0

0 0 0 0 0 0 0 0 0 0 0 0 0
0 0 0 0 0 0 0
"Viruses;Phixviricota;Malgrandaviricetes;Petitvirales;Microviridae;Microviridae__na;robinz_microvirus_RP_132
" 0 0 0 0 1 0 0 0 0 0 0 0 0
0 0 0 0 0 0 0 0 0 0 0 0 0
0 0 0 0 0 0 0 0 0 0 0 0 0
0 0 0 0 0 0 0 0 1 0 0 0 0
0 0 0 0 0 0 0 0 0 0 0 0 0
0 0 0 0 0 0 0
"Viruses;Phixviricota;Malgrandaviricetes;Petitvirales;Microviridae;Microviridae__na;robinz_microvirus_RP_143
" 0 0 0 2 0 6 0 0 0 0 0 0 0
0 0 0 0 0 0 0 0 0 0 0 0 0
0 0 0 0 0 0 0 0 0 0 0 0 0
0 0 0 0 0 0 0 0 0 0 0 0 0
0 0 0 0 0 0 0 0 0 0 0 0 0
0 0 0 0 0 0 0
"Viruses;Phixviricota;Malgrandaviricetes;Petitvirales;Microviridae;Microviridae__na;robinz_microvirus_RP_38"
0 0 0 0 20 8 0 0 0 0 0 0 0
0 0 0 0 0 0 0 0 0 0 0 0 0
0 0 0 0 0 0 0 0 0 0 0 0 0
0 0 0 0 0 0 0 0 0 0 0 0 0
0 0 0 0 0 0 0 0 0 0 0 0 0
0 0 0 0 0 0 0
"Viruses;Phixviricota;Malgrandaviricetes;Petitvirales;Microviridae;Microviridae__na;robinz_microvirus_RP_41"
0 0 0 0 3 1 0 0 0 0 0 0 0
0 0 0 0 0 0 0 0 0 0 0 0 0
0 0 0 0 0 0 0 0 0 0 0 0 0
0 0 0 0 0 0 0 0 0 0 0 0 0
0 0 0 0 0 0 0 0 0 0 0 0 0
0 0 0 0 0 0 0
"Viruses;Phixviricota;Malgrandaviricetes;Petitvirales;Microviridae;Microviridae__na;robinz_microvirus_RP_44"
0 0 0 0 0 3 0 0 0 0 0 0 0
0 0 0 0 0 0 0 0 0 0 0 0 0
0 0 0 0 0 0 0 0 0 0 0 0 0
0 0 0 0 0 0 0 0 0 0 0 0 0
0 0 0 0 0 0 0 0 0 0 0 0 0
0 0 0 0 0 0 0
"Viruses;Phixviricota;Malgrandaviricetes;Petitvirales;Microviridae;Microviridae__na;robinz_microvirus_RP_56"
0 0 0 0 0 8 0 0 0 0 0 0 0
0 0 0 0 0 0 0 0 0 0 0 0 0
0 0 0 0 0 0 0 0 0 0 0 0 0
0 0 0 0 0 0 0 0 0 0 0 0 0
0 0 0 0 0 0 0 0 0 0 0 0 0
0 0 0 0 0 0 0
"Viruses;Phixviricota;Malgrandaviricetes;Petitvirales;Microviridae;Microviridae__na;robinz_microvirus_RP_58"
0 0 0 0 0 2 0 0 0 0 0 0 0
0 0 0 0 0 0 0 0 0 0 0 0 0
0 0 0 0 0 0 0 0 0 0 0 0 0
0 0 0 0 0 0 0 0 0 0 0 0 0

0 0 0 0 0 0 0 0 0 0 0 0 0
0 0 0 0 0 0 0
"Viruses;Phixviricota;Malgrandaviricetes;Petitvirales;Microviridae;Microviridae__na;robinz_microvirus_RP_61"
0 0 0 0 1 0 0 0 0 0 0 0 0
0 0 0 0 0 0 0 0 0 0 0 0 0
0 0 0 0 0 0 0 0 0 0 0 0 0
0 0 0 0 0 0 0 0 0 0 0 0 0
0 0 0 0 0 0 0 0 0 0 0 0 0
0 0 0 0 0 0 0
"Viruses;Phixviricota;Malgrandaviricetes;Petitvirales;Microviridae;Microviridae__na;robinz_microvirus_RP_62"
0 0 0 0 0 0 0 0 0 0 0 0 0
0 0 0 0 0 0 0 0 0 0 0 0 0
0 0 0 0 0 0 0 0 1 0 0 0 0
0 0 0 0 0 0 0 0 0 0 0 0 0
0 0 0 0 0 0 0 0 0 0 0 0 0
0 0 0 0 0 0 0
"Viruses;Phixviricota;Malgrandaviricetes;Petitvirales;Microviridae;Microviridae__na;robinz_microvirus_RP_93"
0 0 0 0 15 0 0 0 0 0 0 0 0
0 0 0 0 0 0 0 0 0 0 0 0 0
0 0 0 0 0 0 0 0 0 0 0 0 0
0 0 0 0 0 0 0 0 0 0 0 0 0
0 0 0 0 0 0 0 0 0 0 0 0 0
0 0 0 0 0 0 0
"Viruses;Phixviricota;Malgrandaviricetes;Petitvirales;Microviridae;Microviridae__na;robinz_microvirus_RP_94"
0 0 0 1 13 3 0 0 0 0 0 0 0
0 0 0 0 0 0 0 0 0 0 0 0 0
0 0 0 0 0 0 0 0 0 0 0 0 0
0 0 0 0 0 0 0 0 0 0 0 0 0
0 0 0 0 0 0 0 0 0 0 0 0 0
0 0 0 0 0 0 0
"Viruses;Phixviricota;Malgrandaviricetes;Petitvirales;Microviridae;Microviridae__na;robinz_microvirus_RP_95"
0 0 0 2 0 0 0 0 0 0 0 0 0
0 0 0 0 0 0 0 0 0 0 0 0 0
0 0 0 0 0 0 0 0 0 0 0 0 0
0 0 0 0 0 0 0 0 0 0 0 0 0
0 0 0 0 0 0 0 0 0 0 0 0 0
0 0 0 0 0 0 0
"Viruses;Phixviricota;Malgrandaviricetes;Petitvirales;Microviridae;Microviridae__na;robinz_microvirus_RP_96"
0 0 0 0 8 4 0 0 0 0 0 0 0
0 0 0 0 0 0 0 0 0 0 0 0 0
0 0 0 0 0 0 0 0 0 0 0 0 0
0 0 0 0 0 0 0 0 2 0 0 0 0
0 0 0 0 0 0 0 0 0 0 0 0 0
0 0 0 0 0 0 0
"Viruses;Phixviricota;Malgrandaviricetes;Petitvirales;Microviridae;Microviridae__na;Sigmofec_virus_UA08Rod
_4411" 0 0 0 0 0 0 0 0 0 0 0 0 0
0 2 2 2 0 0 0 0 0 0 0 0 0
0 0 0 0 0 0 0 0 0 0 0 0 0
0 0 0 0 0 0 0 0 0 0 0 0 0

0	0	0	0	0	0	0	0	0	0	0	0	0
0	0	0	0	0	0	0
"Viruses;Phixviricota;Malgrandaviricetes;Petitvirales;Microviridae;Microviridae__na;Sigmofec_virus_UA08Rod
_4510"	0	0	0	0	0	0	0	0	0	0	0	0	0
0	7	5	12	0	0	0	0	0	0	0	0	0
0	0	0	0	0	0	0	0	0	0	0	0	0
0	0	0	0	0	0	0	0	0	0	0	0	0
0	0	0	0	0	0	0	0	0	0	0	0	0
0	0	0	0	0	0	0
"Viruses;Phixviricota;Malgrandaviricetes;Petitvirales;Microviridae;Microviridae__na;Sigmofec_virus_UA08Rod
_4626"	0	0	0	0	0	0	0	0	0	0	0	0	0
0	0	0	1	0	0	0	0	0	0	0	0	0
0	0	0	0	0	0	0	0	0	0	0	0	0
0	0	0	0	0	0	0	0	0	0	0	0	0
0	0	0	0	0	0	0	0	0	0	0	0	0
0	0	0	0	0	0	0
"Viruses;Phixviricota;Malgrandaviricetes;Petitvirales;Microviridae;Microviridae__na;Sigmofec_virus_UA08Rod
_4686"	0	0	0	0	0	0	0	0	0	0	0	0	0
0	2	0	3	0	0	0	0	0	0	0	0	0
0	0	0	0	0	0	0	0	0	0	0	0	0
0	0	0	0	0	0	0	0	0	0	0	0	0
0	0	0	0	0	0	0	0	0	0	0	0	0
0	0	0	0	0	0	0
"Viruses;Phixviricota;Malgrandaviricetes;Petitvirales;Microviridae;Microviridae__na;Sigmofec_virus_UA08Rod
_4687"	0	0	0	0	0	0	0	0	0	0	0	0	0
0	1	0	1	0	0	0	0	0	0	0	0	0
0	0	0	0	0	0	0	0	0	0	0	0	0
0	0	0	0	0	0	0	0	0	0	0	0	0
0	0	0	0	0	0	0	0	0	0	0	0	0
0	0	0	0	0	0	0
"Viruses;Phixviricota;Malgrandaviricetes;Petitvirales;Microviridae;Microviridae__na;Sigmofec_virus_UA08Rod
_5307"	0	0	0	0	0	0	0	0	0	0	0	0	0
0	0	0	0	0	0	0	0	0	0	0	0	0
0	0	0	1	0	0	0	0	0	0	0	0	0
0	0	0	0	0	0	0	0	0	0	0	0	0
0	0	0	0	0	0	0	0	0	0	0	0	0
0	0	0	0	0	0	0
"Viruses;Phixviricota;Malgrandaviricetes;Petitvirales;Microviridae;Microviridae__na;Sigmofec_virus_UA08Rod
_5492"	0	0	0	0	0	0	0	0	0	0	0	0	0
0	0	0	0	0	0	0	0	0	0	0	0	0
0	0	0	0	0	0	0	0	0	0	0	0	1
0	0	0	0	0	0	0	0	0	0	0	0	0
0	0	0	0	0	0	0	0	0	0	0	0	0
0	0	0	0	0	0	0
"Viruses;Phixviricota;Malgrandaviricetes;Petitvirales;Microviridae;Microviridae__na;Sigmofec_virus_UA08Rod
_5594"	0	0	0	0	1	0	0	0	0	0	0	0	0
0	0	0	0	0	0	0	0	0	0	0	0	0
0	0	0	0	0	0	0	0	0	0	0	0	0
0	1	0	0	0	0	0	0	0	0	0	0	0

0 0 0 0 0 0 0 0 0 0 0 0 0
0 0 0 0 0 0 0
"Viruses;Phixviricota;Malgrandaviricetes;Petitvirales;Microviridae;Microviridae__na;Sigmofec_virus_UA08Rod
_5667" 0 0 0 0 0 0 0 0 0 0 0 0 0
0 0 0 0 0 0 0 0 0 0 0 0 0
0 0 0 0 0 0 0 0 1 0 0 0 0
0 0 0 0 0 0 0 0 0 0 0 0 0
0 0 0 0 0 0 0 0 0 0 0 0 0
0 0 0 0 0 0 0
"Viruses;Phixviricota;Malgrandaviricetes;Petitvirales;Microviridae;Microviridae__na;Sigmofec_virus_UA08Rod
_5935" 0 0 0 0 0 1 0 0 0 0 0 0 0
0 0 0 1 0 0 0 0 0 0 0 0 0
0 0 0 0 0 0 0 0 0 0 0 0 0
0 0 0 0 0 0 0 0 0 0 0 0 0
0 0 0 0 0 0 0 0 0 0 0 0 0
0 0 0 0 0 0 0
"Viruses;Phixviricota;Malgrandaviricetes;Petitvirales;Microviridae;Microviridae__na;Termite_gut_associated_m
icrovirus_1" 0 0 0 1 0 9 0 0 0 0 0 0
0 0 0 0 0 0 0 0 0 0 0 0 0
0 0 0 0 0 0 0 0 0 0 0 0 0
0 0 0 0 0 0 0 0 0 0 0 0 0
0 0 0 0 0 0 0 0 0 0 0 0 0
0 0 0 0 0 0 0 0
"Viruses;Phixviricota;Malgrandaviricetes;Petitvirales;Microviridae;Microviridae__na;Tortoise_microvirus_13"
0 0 0 0 0 0 0 0 0 0 0 0 0
0 0 0 0 0 0 0 0 0 0 0 0 0
0 0 0 0 0 0 0 0 0 0 0 0 0
0 0 0 0 0 0 0 0 0 0 0 0 0
0 0 0 0 0 0 0 0 0 0 0 0 0
0 0 0 0 0 3 0
"Viruses;Phixviricota;Malgrandaviricetes;Petitvirales;Microviridae;Microviridae__na;Tortoise_microvirus_50"
0 0 0 1 3 0 0 0 0 0 0 0 0
0 0 0 0 0 0 0 0 0 0 0 0 0
0 0 0 0 0 0 0 0 0 0 0 0 0
0 0 0 0 0 0 0 0 0 0 0 0 0
0 0 0 0 0 0 0 0 0 0 0 0 0
0 0 0 0 0 0 0
"Viruses;Phixviricota;Malgrandaviricetes;Petitvirales;Microviridae;Microviridae__na;Tortoise_microvirus_78"
0 0 0 0 1 0 0 0 0 0 0 0 0
0 0 0 0 0 0 0 0 0 0 0 0 0
0 0 0 0 0 0 0 0 0 0 0 0 0
0 0 0 0 0 0 0 0 0 0 0 0 0
0 0 0 0 0 0 0 0 0 0 0 0 0
0 0 0 0 0 0 0
"Viruses;Phixviricota;Malgrandaviricetes;Petitvirales;Microviridae;Microviridae__na;Tortoise_microvirus_85"
0 0 0 0 6 0 0 0 0 0 0 0 0
0 0 0 0 0 0 0 0 0 0 0 0 0
0 0 0 0 0 0 0 0 0 0 0 0 0
0 0 0 0 0 0 0 0 0 0 0 0 0

0 0 0 0 0 0 0 0 0 0 0 0 0
0 0 0 0 0 0 0
"Viruses;Phixviricota;Malgrandaviricetes;Petitvirales;Microviridae;Microviridae__na;Tortoise_microvirus_88"
0 0 0 0 0 0 0 0 0 0 0 0 0
0 0 0 0 0 0 0 0 0 0 0 0 0
0 0 0 0 0 0 0 0 0 0 0 0 0
0 0 0 0 0 0 0 0 3 0 0 0 0
0 0 0 0 0 0 0 0 0 0 0 0 0
0 0 0 0 0 0 0
"Viruses;Phixviricota;Malgrandaviricetes;Petitvirales;Microviridae;Microviridae__na;Tortoise_microvirus_90"
0 0 0 0 15 0 0 0 0 0 0 0 0
0 0 0 0 0 0 0 0 0 0 0 0 0
0 0 0 0 0 0 0 0 0 0 0 0 0
0 0 0 0 0 0 0 0 0 0 0 0 0
0 0 0 0 0 0 0 0 0 0 0 0 0
0 0 0 0 0 0 0
"Viruses;Phixviricota;Malgrandaviricetes;Petitvirales;Microviridae;Microviridae__na;Trichosanthes_kirilowii_go
kushovirus" 0 0 0 0 4 0 0 0 0 0 0 0
0 0 0 0 0 0 0 0 0 0 0 0 0
0 0 0 0 0 0 0 0 0 0 3 0 0
0 0 0 0 0 0 0 0 0 0 0 0 0
0 0 0 0 0 0 0 0 0 0 0 0 0
0 0 0 0 0 0 0 0
"Viruses;Phixviricota;Malgrandaviricetes;Petitvirales;Microviridae;Microviridae__na;uncultured_marine_Micro
viridae"0 0 1 2 8 10 0 0 0 0 0 0 0
0 0 0 1 0 0 0 0 0 2 0 0 0
0 0 0 0 0 0 0 0 0 0 0 0 0
0 0 0 0 0 0 0 0 2 0 0 0 0
0 0 0 0 0 0 0 0 0 0 0 0 0
0 0 0 0 0 0 0
"Viruses;Phixviricota;Malgrandaviricetes;Petitvirales;Microviridae;Microviridae__na;uncultured_Microviridae"
0 0 1 3 29 0 0 0 0 0 0 0 0
0 0 0 0 1 0 0 0 0 0 0 0 0
0 0 0 0 0 0 0 0 0 0 0 0 0
0 0 0 0 0 0 0 0 4 0 0 0 0
0 0 0 0 0 0 0 0 0 0 0 0 0
0 0 0 0 0 0 0
"Viruses;Phixviricota;Malgrandaviricetes;Petitvirales;Microviridae;Microviridae__na;Wigfec_virus_K19_141"
0 0 0 0 10 0 0 0 0 0 0 0 0
0 0 0 1 0 0 0 0 0 0 0 0 0
0 0 0 0 0 0 0 0 0 0 0 0 0
0 0 0 0 0 0 0 0 0 0 0 0 0
0 0 0 0 0 0 0 0 0 0 0 0 0
0 0 0 0 0 1 0
"Viruses;Phixviricota;Malgrandaviricetes;Petitvirales;Microviridae;Microviridae__na;Wigfec_virus_K19_151"
0 0 1 2 15 12 0 0 0 0 0 0 0
0 0 0 0 0 0 0 0 0 0 1 0 0
0 0 0 0 0 0 0 0 0 0 0 0 0
0 0 0 0 0 0 0 0 8 0 0 0 0

0 0 0 0 0 0 0 0 0 0 0 0 0
0 0 0 0 0 0 0
"Viruses;Phixviricota;Malgrandaviricetes;Petitvirales;Microviridae;Microviridae__na;Wigfec_virus_K19_152"
0 0 1 0 17 0 0 0 0 0 0 0 0
0 0 0 0 0 0 0 0 0 0 0 0 0
0 0 0 0 0 0 0 0 0 0 0 0 0
0 0 0 0 0 0 0 0 0 0 0 0 0
0 0 0 0 0 0 0 0 0 0 0 0 0
0 0 0 0 0 0 0
"Viruses;Phixviricota;Malgrandaviricetes;Petitvirales;Microviridae;Microviridae__na;Wigfec_virus_K19_160"
0 0 0 1 0 0 0 0 0 0 0 0 0
0 0 0 0 0 0 0 0 0 1 0 0 0
0 0 0 0 0 0 0 0 0 0 0 0 0
0 0 0 0 0 0 0 0 0 0 0 0 0
0 0 0 0 0 0 0 0 0 0 0 0 0
0 0 0 0 0 0 0
"Viruses;Phixviricota;Malgrandaviricetes;Petitvirales;Microviridae;Microviridae__na;Wigfec_virus_K19_161"
0 0 1 1 4 4 0 0 0 0 0 0 0
0 0 0 0 0 0 0 0 0 0 0 0 0
0 0 0 0 0 0 0 0 0 0 0 0 0
0 0 0 0 0 0 0 0 2 0 0 0 0
0 0 0 0 0 0 0 0 0 0 0 0 0
0 0 0 0 0 0 0
"Viruses;Phixviricota;Malgrandaviricetes;Petitvirales;Microviridae;Microviridae__na;Wigfec_virus_K19_170"
0 0 0 2 11 10 0 0 0 0 0 0 0
0 0 0 0 0 0 0 0 0 1 0 0 0
0 0 0 0 0 0 0 0 0 0 0 0 0
0 0 2 0 0 0 0 0 0 0 0 0 0
1 0 0 0 0 0 0 0 0 0 0 0 0
0 0 0 0 0 0 0
"Viruses;Phixviricota;Malgrandaviricetes;Petitvirales;Microviridae;Microviridae__na;Wigfec_virus_K19_177"
0 0 1 0 17 2 0 0 0 0 0 0 0
0 0 0 0 0 0 0 0 0 0 0 0 0
0 0 0 0 0 0 0 0 0 0 0 0 0
0 0 0 0 0 0 0 0 5 0 0 0 0
0 0 0 0 0 0 0 0 0 0 0 0 0
0 0 0 0 0 1 0
"Viruses;Phixviricota;Malgrandaviricetes;Petitvirales;Microviridae;Microviridae__na;Wigfec_virus_K19_179"
0 0 1 1 41 19 0 0 0 0 0 0 0
0 0 0 0 0 0 0 0 0 1 0 0 0
0 0 1 0 0 0 0 0 0 0 0 0 0
0 0 0 0 0 0 0 0 1 0 0 0 0
0 0 0 0 0 0 0 0 0 0 0 0 0
0 0 0 0 0 1 0
"Viruses;Phixviricota;Malgrandaviricetes;Petitvirales;Microviridae;Microviridae__na;Wigfec_virus_K19_81"
0 0 0 1 10 14 0 0 0 0 0 0 0
0 0 0 0 0 0 0 0 0 0 0 0 0
0 0 0 0 0 0 0 0 0 0 0 0 0
0 0 0 0 0 0 0 0 3 0 0 0 0

0 0 0 0 0 0 0 0 0 0 0 0 0
0 0 0 0 0 1 0
"Viruses;Phixviricota;Malgrandaviricetes;Petitvirales;Microviridae;Microviridae__na;Wigfec_virus_K19_83"
0 0 0 3 35 27 0 0 0 0 0 0 0
0 0 0 0 0 0 0 0 0 0 0 0 0
0 0 0 0 0 0 0 0 0 0 0 0 0
0 0 0 0 0 0 0 0 0 0 0 0 0
0 0 0 0 0 0 0 0 0 0 0 0 0
0 0 0 0 0 0 0
"Viruses;Phixviricota;Malgrandaviricetes;Petitvirales;Microviridae;Microviridae__na;Wigfec_virus_K19_94"
0 0 0 0 2 0 0 0 0 0 0 0 0
0 0 0 0 0 0 0 0 0 0 0 0 0
0 0 0 0 0 0 0 0 0 0 0 0 0
0 0 0 0 0 0 0 0 0 0 0 0 0
0 0 0 0 0 0 0 0 0 0 0 0 0
0 0 0 0 0 0 0
"Viruses;Phixviricota;Malgrandaviricetes;Petitvirales;Microviridae;Microviridae__uc;Microviridae__uc" 0
0 21 58 549 481 0 0 4 0 0 0 5 7
331 408 953 33 1 0 11 8 57 35 6 7 1968
577 891 2045 0 0 2 1 976 38 4 2 23 5
3 92 0 1 20 5 0 75 0 1 2 0 50
9126 12 7 0 0 0 144 1 0 26 38 1 8
13 8 252 118 20 0
"Viruses;Phixviricota;Malgrandaviricetes;Petitvirales;Microviridae;Microvirus;Antarctic_microvirus_COCH21_
V_SP_13" 0 0 0 2 0 0 0 0 0 0 0 0
0 0 0 0 0 0 0 0 0 0 0 0 0
0 0 0 0 0 0 0 0 0 0 0 0 0
0 0 0 0 0 0 0 0 0 1 0 0 0
0 0 0 0 0 0 0 0 0 0 0 0 0
0 0 0 0 0 0 0 0
"Viruses;Phixviricota;Malgrandaviricetes;Petitvirales;Microviridae;Microvirus;Antarctic_microvirus_TYR_006_V
_SP_13"0 0 0 0 0 1 0 0 0 0 0 0 0
0 0 0 0 0 0 0 0 0 0 0 0 0
0 0 0 0 0 0 0 0 0 0 0 0 0
0 0 0 0 0 0 0 0 0 0 0 0 0
0 0 0 0 0 0 0 0 0 0 0 0 0
0 0 0 0 0 0 0
"Viruses;Phixviricota;Malgrandaviricetes;Petitvirales;Microviridae;Microvirus;Army_ant_associated_microvirus
_6" 0 0 0 1 2 0 0 0 0 0 0 0 0
0 0 0 0 0 0 0 0 0 0 0 0 0
0 0 0 0 0 0 0 0 0 0 0 0 0
0 0 0 0 0 0 0 0 0 0 0 0 0
0 0 0 0 0 0 0 0 0 0 0 0 0
0 0 0 0 0 0 0
"Viruses;Phixviricota;Malgrandaviricetes;Petitvirales;Microviridae;Microvirus;Blackfly_microvirus_SF02" 0
0 3 15 147 35 0 0 0 0 0 0 0 0
0 0 2 0 0 0 0 0 0 1 0 0 0
0 0 0 0 0 0 0 0 3 0 0 0 0
0 0 0 0 1 1 0 32 0 0 0 0 1

0 0 0 0 0 0 0 0 0 0 0 0 0
0 0 0 0 2 0
"Viruses;Phixviricota;Malgrandaviricetes;Petitvirales;Microviridae;Microvirus;Chicken_microvirus_mg7_14"
0 0 0 1 0 0 0 0 0 0 0 0 0
0 0 0 0 0 0 0 0 0 0 0 0 0
0 0 0 0 0 0 0 0 0 0 0 0 0
0 0 0 0 0 0 0 0 0 0 0 0 0
0 0 0 0 0 0 0 0 0 0 0 0 0
0 0 0 0 0 0 0
"Viruses;Phixviricota;Malgrandaviricetes;Petitvirales;Microviridae;Microvirus;Chicken_microvirus_mg7_16"
0 0 0 0 0 2 0 0 0 0 0 0 0
0 0 0 0 0 0 0 0 0 0 0 0 0
0 0 0 0 0 0 0 0 0 0 0 0 0
0 0 0 0 0 0 0 0 0 0 0 0 0
0 0 0 0 0 0 0 0 0 0 0 0 0
0 0 0 0 0 0 0
"Viruses;Phixviricota;Malgrandaviricetes;Petitvirales;Microviridae;Microvirus;Chicken_microvirus_mg8_109"
0 0 0 0 3 0 0 0 0 0 0 0 0
0 0 0 0 0 0 0 0 0 0 0 0 0
0 0 0 1 0 0 0 0 0 0 0 0 0
0 0 0 0 0 0 0 0 1 0 0 0 0
0 0 0 0 0 0 0 0 0 0 0 0 0
0 0 0 0 0 0 0
"Viruses;Phixviricota;Malgrandaviricetes;Petitvirales;Microviridae;Microvirus;Rattus_norvegicus_microvirus"
0 0 0 0 0 0 0 0 0 0 0 0 0
0 0 0 0 0 0 0 0 0 0 0 0 0
0 0 0 0 0 0 0 0 3 0 0 0 0
0 0 0 0 0 0 0 0 0 0 0 0 0
1 0 0 0 0 0 0 0 0 0 1 1 0
0 0 0 0 0 0 0
"Viruses;Pisuviricota;Duplopiviricetes;Durnavirales;Partitiviridae;Partitiviridae__na;Alternaria_alternata_partiti
virus_1" 0 0 0 0 0 0 0 0 0 0 7 0
0 0 0 0 0 0 0 0 0 0 0 0 0
0 0 0 0 0 0 0 0 0 0 0 0 0
0 0 0 0 0 0 0 0 0 0 0 0 0
0 0 0 0 0 0 0 0 0 0 0 0 0
0 0 0 0 0 0 0 0
"Viruses;Pisuviricota;Duplopiviricetes;Durnavirales;Partitiviridae;Partitiviridae__na;Fusarium_equiseti_partitivi
rus_1" 0 0 0 0 0 0 0 0 0 0 3 0 0
0 0 0 0 0 0 0 0 0 0 0 0 0
0 0 0 0 0 0 0 0 0 0 0 0 0
0 0 0 0 0 0 0 0 0 0 0 0 0
0 0 0 0 0 0 0 0 0 0 0 0 0
0 0 0 0 0 0 0
"Viruses;Pisuviricota;Duplopiviricetes;Durnavirales;Partitiviridae;Partitiviridae__na;Partitiviridae_sp." 0
0 0 0 0 0 0 0 0 0 11 0 0 0
0 0 0 0 0 0 0 0 0 0 0 0 0
0 0 0 0 0 0 0 0 0 0 0 0 0
0 0 0 0 0 0 0 0 0 0 0 0 0

0 0 0 0 0 0 0 0 0 0 0 0 0
0 0 0 0 0 0
"Viruses;Pisuviricota;Duplopiviricetes;Durnavirales;Partitiviridae;Partitiviridae__na;Rose_partitivirus" 0
0 0 0 0 0 0 0 0 0 0 0 0 0
0 0 0 0 0 0 0 0 0 0 0 0 0
0 0 0 0 0 0 0 0 0 0 0 0 0
0 0 0 0 0 0 0 0 0 0 0 0 0
0 0 0 0 0 0 0 0 0 0 0 0 0
0 0 0 1 0 0
"Viruses;Pisuviricota;Duplopiviricetes;Durnavirales;Partitiviridae;Partitiviridae__na;Ustilaginoidea_virens_parti
tivirus_5" 0 0 0 0 0 0 0 0 0 0 1 0
0 0 0 0 0 0 0 0 0 0 0 0 0
0 0 0 0 0 0 0 0 0 0 0 0 0
0 0 0 0 0 0 0 0 0 0 0 0 0
0 0 0 0 0 0 0 0 0 0 0 0 0
0 0 0 0 0 0 0 0
"Viruses;Pisuviricota;Duplopiviricetes;Durnavirales;Picobirnaviridae;Orthopicobirnavirus;Orthopicobirnavirus_
hominis" 0 0 0 0 0 0 0 0 0 0 0 0
0 0 2 2 2 0 0 0 0 0 0 0 0
0 0 0 0 0 0 0 0 0 0 0 0 0
0 0 0 0 0 0 0 0 0 0 0 0 10
0 0 0 0 0 0 0 0 0 0 0 0 0
0 0 0 0 0 0 1 0
"Viruses;Pisuviricota;Duplopiviricetes;Durnavirales;Picobirnaviridae;Picobirnaviridae__na;Macaque_picobirnav
irus_32"0 0 0 0 0 0 0 0 0 0 0 0 0
0 0 1 0 0 0 0 0 0 0 0 0 0
0 0 0 0 0 0 0 0 0 0 0 0 0
0 0 0 0 0 0 0 0 0 0 0 0 0
0 0 0 0 0 0 0 0 0 0 0 0 0
0 0 0 0 0 0 0
"Viruses;Pisuviricota;Duplopiviricetes;Durnavirales;Picobirnaviridae;Picobirnaviridae__na;Picobirnaviridae_sp.
" 0 0 0 0 0 0 0 0 0 0 0 0 0
0 1209 1013 79 0 0 0 0 0 0 0 0 0
0 0 0 0 0 0 0 0 0 0 0 0 4
3 0 0 0 0 0 0 0 0 0 0 0 0
0 0 0 0 1 0 0 0 0 0 0 0 0
0 0 0 0 0 0 0
"Viruses;Pisuviricota;Duplopiviricetes;Durnavirales;Picobirnaviridae;Picobirnaviridae__na;Picobirnaviridae_sp.
_ct6rZ1" 0 0 0 0 0 0 0 0 0 0 0 0
0 0 3 4 0 0 0 0 0 0 0 0 0
0 0 0 0 0 0 0 0 0 0 0 0 0
0 0 0 0 0 0 0 0 0 0 0 0 0
0 0 0 0 0 0 0 0 0 0 0 0 0
0 0 0 0 0 0 0 0
"Viruses;Pisuviricota;Duplopiviricetes;Durnavirales;Picobirnaviridae;Picobirnaviridae__na;Picobirnavirus_PRE
DICT_PbV-104"0 0 0 0 0 0 0 0 0 0 0 0
0 0 8 3 0 0 0 0 0 0 0 0 0
0 0 0 0 0 0 0 0 0 0 0 0 0
0 0 0 0 0 0 0 0 0 0 0 0 0

0 0 0 0 0 0 0 0 0 0 0 0 0
0 0 0 0 0 0 0 0
"Viruses;Pisuviricota;Duplopiviricetes;Durnavirales;Picobirnaviridae;Picobirnaviridae__na;Picobirnavirus_PREDICT_PbV-109" 0 0 0 0 0 0 0 0 0 0 0 0
0 0 1 1 0 0 0 0 0 0 0 0 0
0 0 0 0 0 0 0 0 0 0 0 0 0
0 0 0 0 0 0 0 0 0 0 0 0 0
0 0 0 0 0 0 0 0 0 0 0 0 0
0 0 0 0 0 0 0 0
"Viruses;Pisuviricota;Duplopiviricetes;Durnavirales;Picobirnaviridae;Picobirnaviridae__na;Picobirnavirus_PREDICT_PbV-89" 0 0 0 0 0 0 0 0 0 0 0 0
0 0 1 0 0 0 0 0 0 0 0 0 0
0 0 0 0 0 0 0 0 0 0 0 0 0
0 0 0 0 0 0 0 0 0 0 0 0 0
0 0 0 0 0 0 0 0 0 0 0 0 0
0 0 0 0 0 0 0 0
"Viruses;Pisuviricota;Duplopiviricetes;Durnavirales;Picobirnaviridae;Picobirnaviridae__na;Simian_picobirnavirus" 0 0 0 0 0 0 0 0 0 0 0 0 0
0 0 0 0 0 0 0 0 0 0 0 0 0
0 0 0 0 0 0 0 0 0 0 0 0 0
0 0 0 0 0 0 0 0 0 0 0 4 0
0 0 0 0 0 0 0 0 0 0 0 0 0
0 0 0 0 0 0 0
"Viruses;Pisuviricota;Duplopiviricetes;Durnavirales;Picobirnaviridae;Picobirnaviridae__uc;Picobirnaviridae__uc" 0 0 0 0 0 0 0 0 0 0 0 0 0
0 1 2 2 0 0 0 0 0 0 0 0 0
0 0 0 0 0 0 0 0 0 0 0 0 0
0 0 0 0 0 0 0 0 0 0 0 31 6
0 0 0 0 0 0 4 0 0 0 0 0 0
0 0 0 0 0 1 0
"Viruses;Pisuviricota;Duplopiviricetes;Durnavirales;Picobirnaviridae;Picobirnavirus;Hubei_picobirnavirus_53"
0 0 0 0 0 0 0 0 0 0 0 0 0
0 3 3 1 0 0 0 0 0 0 0 0 0
0 0 0 0 0 0 0 0 0 0 0 0 0
0 0 0 0 0 0 0 0 0 0 0 0 0
0 0 0 0 0 0 0 0 0 0 0 0 0
0 0 0 0 0 0 0
"Viruses;Pisuviricota;Duplopiviricetes;Durnavirales;Picobirnaviridae;Picobirnavirus;Marmot_picobirnavirus"
0 0 0 0 0 0 0 0 0 0 0 0 0
0 3 1 0 0 0 0 0 0 0 0 0 0
0 0 0 0 0 0 0 0 0 0 0 0 0
0 0 0 0 0 0 0 0 0 0 0 0 0
0 0 0 0 0 0 0 0 0 0 0 0 0
0 0 0 0 0 0 0
"Viruses;Pisuviricota;Duplopiviricetes;Durnavirales;Picobirnaviridae;Picobirnavirus;Picobirnavirus__uc" 0
0 0 0 0 0 0 0 0 0 0 0 0 0
4 3 2 0 0 0 0 0 0 0 0 0 0
0 0 0 0 0 0 0 0 0 0 0 0 0
0 0 0 0 0 0 0 0 0 0 5 0 0

0 0 0 0 0 0 0 0 0 0 0 0 0
0 0 0 0 1 0
"Viruses;Pisuviricota;Duplopiviricetes;Durnavirales;Picobirnaviridae;Picobirnavirus;Picobirnavirus_sp." 0
0 0 0 0 0 0 0 0 0 4 1 0 0
18 12 4 0 0 0 0 0 0 0 0 0 0
0 0 0 0 0 0 0 0 0 0 0 7 3
0 0 0 0 0 0 0 0 0 0 23 5 7
0 0 0 2 1 0 0 0 0 0 0 0 0
0 0 0 0 1 0
"Viruses;Pisuviricota;Duplopiviricetes;Durnavirales;Picobirnaviridae;Picobirnavirus;Porcine_picobirnavirus"
0 0 0 0 0 0 0 0 0 0 0 0 0
0 22 15 1 1 0 0 0 0 0 0 0 0
0 0 0 0 0 0 0 0 0 0 0 0 0
2 0 0 0 0 0 0 0 0 0 0 5 0
0 0 0 0 2 4 0 0 0 0 0 0 0
0 0 0 0 0 0 0
"Viruses;Pisuviricota;Duplopiviricetes;Durnavirales;Picobirnaviridae;Picobirnavirus;Rat_picobirnavirus" 0
0 0 0 0 0 0 0 0 0 0 0 0 0
0 0 0 0 0 0 0 0 0 0 0 0 0
0 0 0 0 0 1 0 0 0 0 0 0 0
0 0 0 0 0 0 0 0 0 0 0 0 0
0 0 0 0 0 0 0 0 0 0 0 0 0
0 0 0 0 0 0
"Viruses;Pisuviricota;Duplopiviricetes;Durnavirales;Picobirnaviridae;Picobirnavirus;Tasmanian_devil-associated_picobirnavirus_5" 0 0 0 0 0 0 0 0 0 0
0 0 0 0 0 1 0 0 0 0 0 0 0
0 0 0 0 0 0 0 0 0 0 0 0 0
0 0 0 0 0 0 0 0 0 0 0 0 0
0 0 0 0 0 0 0 0 0 0 0 0 0
0 0 0 0 0 0 0 0 0 0
"Viruses;Pisuviricota;Pisoniviricetes;Nidovirales;Coronaviridae;Betacoronavirus;Severe_acute_respiratory_syndrome-related_coronavirus" 0 0 0 0 0 0 0 0 0 1
0 0 0 0 0 0 0 0 0 0 0 0 0
0 0 0 0 0 0 0 0 0 0 0 0 0
0 0 0 0 0 0 0 0 0 0 0 0 0
0 0 0 0 0 0 0 0 0 0 0 0 0
0 0 0 0 0 0 0 0 0 0
"Viruses;Pisuviricota;Pisoniviricetes;Nidovirales;Coronaviridae;Coronaviridae__uc;Coronaviridae__uc" 0
0 0 0 0 0 0 0 1 0 0 0 0 0
0 0 0 0 0 0 0 0 0 0 0 0 0
0 0 0 0 0 0 0 0 0 0 0 0 0
0 0 0 0 2 0 12 0 0 0 0 0 0
0 0 0 0 0 0 0 0 0 0 0 0 0
0 0 0 0 0 0
"Viruses;Pisuviricota;Pisoniviricetes;Picornavirales;Picornaviridae;Enterovirus;Enterovirus__uc" 0 0
0 0 0 0 0 0 0 0 0 0 0 0 0
1 0 0 0 0 0 0 0 0 0 0 0 0
0 0 0 0 0 0 0 0 0 0 0 0 0
0 0 0 0 0 0 0 0 0 0 0 0 0

0 0 0 0 0 0 0 0 0 0 0 0 0
0 0 0 0 0
"Viruses;Pisuviricota;Pisoniviricetes;Picornavirales;Picornaviridae;Picornaviridae__na;Sichuan_sapelo-like_virus_1" 0 0 0 0 0 0 0 0 0 0 0 0
0 0 0 0 0 0 0 0 0 0 0 0 0
0 0 0 0 0 0 0 0 0 2 1 0 0
0 0 0 0 0 0 0 0 0 0 0 0 0
0 0 0 0 0 0 1 0 0 0 0 0 0
0 0 0 0 0 0 0 0
"Viruses;Pisuviricota;Pisoniviricetes;Picornavirales;Picornaviridae;Picornaviridae__na;Sichuan_tescho-like_virus_1" 0 0 0 0 0 0 0 0 0 0 0 0
0 0 0 0 0 0 0 0 0 0 0 0 0
0 0 0 0 0 0 0 5 2 0 0 0 0
1 6 0 0 0 0 0 0 0 0 0 0 0
0 0 0 0 0 1 0 0 0 0 0 0 0
0 0 0 0 0 0 0 0
"Viruses;Pisuviricota;Pisoniviricetes;Sobelivirales;Solemoviridae;Polerovirus;Beet_western_yellows_virus"
0 0 0 0 0 0 0 0 0 0 0 0 0
0 0 1 0 0 0 0 0 0 0 0 0 0
0 0 0 0 0 0 0 0 0 0 0 0 0
0 0 0 0 0 0 0 0 0 0 0 0 0
0 0 0 0 0 0 0 0 0 0 0 0 0
0 0 0 0 0 0 0
"Viruses;Pisuviricota;Pisoniviricetes;Sobelivirales;Solemoviridae;Sobemovirus;Xufa_yellow_dwarf_virus" 0
0 0 0 0 0 0 0 0 0 0 0 0 0
0 0 0 0 0 0 10 10 0 0 0 0 0
0 0 0 0 0 0 0 0 0 0 0 0 0
0 0 0 0 0 0 0 0 0 0 0 0 0
0 0 0 0 0 0 0 0 0 0 0 0 0
0 0 0 0 0 0
"Viruses;Pisuviricota;Stelpaviricetes;Stellavirales;Astroviridae;Mamastrovirus;Mamastrovirus_3" 0 0
0 0 0 0 0 0 0 0 0 0 0 0 2
1 1 1 0 0 1 0 0 0 0 0 0 1
16 4 0 0 0 0 0 0 0 0 1 0 0
0 0 0 0 0 0 0 0 0 1 5 0 0
0 0 0 0 3 0 0 0 0 0 0 0 0
0 0 0 0 0
"Viruses;Preplasmiviricota;Polintoviricetes;Orthopolintovirales;Adintoviridae;Adintoviridae__na;Branchiostoma_lancelet_adintovirus" 0 0 0 0 0 0 0 0 0 0 0
0 0 0 0 0 0 0 0 0 0 0 1 0
0 0 0 0 0 0 0 0 0 0 0 0 0
0 0 0 0 0 0 0 0 0 0 0 0 0
0 0 0 0 0 0 0 0 0 0 0 0 0
0 0 0 0 0 0 0 0 0
"Viruses;Preplasmiviricota;Polintoviricetes;Orthopolintovirales;Adintoviridae;Adintoviridae__na;Larimichthys_croaker_adintovirus" 0 0 0 0 0 0 0 0 0 0 0
0 0 0 0 0 0 0 0 0 0 0 0 0
0 0 0 0 0 0 0 0 0 0 0 0 0
0 0 0 0 0 0 0 0 0 0 1 0 0

0 0 0 0 0 0 0 0 0 0 0 0 0
0 0 0 0 0 0 0 0 0
"Viruses;Preplasmiviricota;Tectiliviricetes;Vinavirales;Corticoviridae;Corticoviridae__na;Corticoviridae_sp."
0 0 0 0 0 0 0 0 0 0 0 0 0
0 0 0 0 0 0 0 0 0 0 0 2 0
0 0 0 0 1 1 0 0 0 0 0 0 0
0 0 3 0 0 0 0 0 0 0 0 1 0
0 0 0 0 0 0 0 0 0 0 0 0 0
0 0 0 0 0 0 0
"Viruses;Uroviricota;Caudoviricetes;Caudoviricetes__na;Ackermannviridae;Ackermannviridae__na;Ackermann
viridae_sp." 0 0 1 0 0 0 0 0 0 0 0 0
1 1 2 1 0 0 0 1 0 0 0 0 1
1 1 0 0 0 0 1 0 0 0 0 3 0
0 1 0 0 0 0 0 0 0 0 0 1 0
0 0 0 1 1 0 0 0 0 19 4 0 0
0 0 0 0 0 0 0 0
"Viruses;Uroviricota;Caudoviricetes;Caudoviricetes__na;Ackermannviridae;Ackermannviridae__na;Ackermann
viridae_sp._ctaCq7" 0 0 0 0 0 0 0 0 0 0 0
0 0 0 0 0 0 0 0 0 0 0 0 0
0 0 0 0 0 0 0 0 0 0 0 0 0
0 0 0 0 0 0 0 0 0 0 0 0 0
0 0 0 0 0 0 0 0 0 0 0 0 0
0 0 0 0 0 0 0 1 0
"Viruses;Uroviricota;Caudoviricetes;Caudoviricetes__na;Ackermannviridae;Ackermannviridae__na;Ackermann
viridae_sp._ctQad106" 0 0 0 0 0 0 0 0 0 0 0
0 2 0 0 0 0 0 0 0 0 0 0 0
0 0 0 0 0 0 0 0 0 0 0 0 0
0 0 0 0 0 0 0 0 0 0 0 0 0
0 0 0 0 0 0 0 0 0 0 0 0 0
0 0 0 0 0 0 0 0 0
"Viruses;Uroviricota;Caudoviricetes;Caudoviricetes__na;Ackermannviridae;Ackermannviridae__uc;Ackermann
viridae__uc" 0 0 0 0 0 0 0 0 0 0 0 0
2 1 0 0 0 1 0 0 0 0 0 0 0
0 0 0 0 0 1 0 0 0 0 0 0 0
0 0 0 0 0 0 0 0 0 0 0 0 0
0 0 0 1 1 0 0 0 0 0 0 0 0
0 0 0 0 0 0 0 0
"Viruses;Uroviricota;Caudoviricetes;Caudoviricetes__na;Ackermannviridae;Kuttervirus;Kuttervirus__uc"0
0 0 0 0 1 0 0 0 0 0 0 0 0
0 0 0 0 0 0 0 0 0 0 0 0 0
0 0 0 0 0 0 0 0 0 0 0 0 0
0 0 0 0 0 0 0 0 0 0 0 0 0
0 0 0 0 0 0 0 0 0 0 0 0 0
0 0 0 0 0 0
"Viruses;Uroviricota;Caudoviricetes;Caudoviricetes__na;Ackermannviridae;Nezavisimistyvirus;Nezavisimistyv
irus__uc" 0 0 0 0 0 3 0 0 0 0 0 0
0 0 0 0 0 0 0 0 0 0 0 0 0
0 0 0 0 0 0 0 0 0 0 0 0 0
0 0 0 0 0 0 0 0 0 0 0 0 0

0 0 0 0 0 0 0 0 0 0 0 0 0
0 0 0 0 0 0 0 0
"Viruses;Uroviricota;Caudoviricetes;Caudoviricetes__na;Ackermannviridae;Taipeivirus;Taipeivirus__uc" 0
0 1 0 0 0 0 0 0 0 0 0 0 0
0 0 0 0 0 0 0 0 0 0 0 0 0
0 0 0 0 0 0 0 0 0 0 0 0 0
0 0 0 0 0 0 0 0 0 0 0 0 0
0 0 0 0 0 0 0 0 0 0 0 0 0
0 0 0 0 0 0
"Viruses;Uroviricota;Caudoviricetes;Caudoviricetes__na;Ackermannviridae;Tedavirus;Tedavirus_A829" 0
0 0 0 0 6 0 0 0 0 0 0 0 0
0 0 0 0 0 0 0 0 0 0 0 0 0
0 0 0 0 0 0 0 0 0 0 0 0 0
0 0 0 0 0 0 0 0 0 0 0 0 0
0 0 0 0 0 0 0 0 0 0 0 0 0
0 0 0 0 1 0
"Viruses;Uroviricota;Caudoviricetes;Caudoviricetes__na;Aggregaviridae;Harrekavirus;Harrekavirus_harreka"
0 0 0 0 9 0 0 0 0 0 0 0 0
0 0 0 0 0 0 0 0 0 0 0 0 0
0 0 0 0 0 0 0 0 0 0 0 0 0
0 0 0 0 0 0 0 0 3 0 0 0 0
0 0 0 0 0 0 0 0 0 0 0 0 0
0 0 0 0 0 0 0
"Viruses;Uroviricota;Caudoviricetes;Caudoviricetes__na;Assiduviridae;Assiduviridae__na;Rhodobacteraceae_p
hage_LS06-2018-MD05" 0 0 3 10 2 5 0 0 0 0 0
0 0 0 0 0 0 0 0 0 0 0 0 0
0 0 0 0 0 0 0 0 0 0 0 0 0
0 0 0 0 0 0 0 0 0 0 0 0 0
0 0 0 0 0 0 0 0 0 0 0 0 0
0 0 0 0 0 0 0 1 0
"Viruses;Uroviricota;Caudoviricetes;Caudoviricetes__na;Assiduviridae;Assiduviridae__uc;Assiduviridae__uc"
0 0 1 0 3 0 0 0 0 0 0 0 0
0 0 0 0 0 0 0 0 0 0 0 0 0
0 0 0 0 0 0 0 0 0 0 0 0 0
0 0 0 0 0 0 0 0 0 0 0 0 0
0 0 0 0 0 0 0 0 0 0 0 0 0
0 0 0 0 0 0 0
"Viruses;Uroviricota;Caudoviricetes;Caudoviricetes__na;Assiduviridae;Cebadecemvirus;Cebadecemvirus_phi10
una" 0 0 0 1 0 2 0 0 0 0 0 0 0
0 0 0 0 0 0 0 0 0 0 0 0 0
0 0 0 0 0 0 0 0 0 0 0 0 0
0 0 0 0 0 0 0 0 9 0 0 0 0
0 0 0 0 0 0 0 0 0 0 0 0 0
0 0 0 0 0 0 0
"Viruses;Uroviricota;Caudoviricetes;Caudoviricetes__na;Assiduviridae;Cellubavirus;Cellubavirus_phi19una"
0 0 0 2 0 0 0 0 0 0 0 0 0
0 0 0 0 0 0 0 0 0 0 0 0 0
0 0 0 0 0 0 0 0 0 0 0 0 0
0 0 0 0 0 0 0 0 0 0 0 0 0

0 0 0 0 0 0 0 0 0 0 0 0 0
0 0 0 0 0 0 0
"Viruses;Uroviricota;Caudoviricetes;Caudoviricetes__na;Assiduviridae;Nekkelsvirus;Nekkelsvirus__uc" 0
0 1 0 0 0 0 0 0 0 0 0 0 0
0 0 0 0 0 0 0 0 0 0 0 0 0
0 0 0 0 0 0 0 0 0 0 0 0 0
0 0 0 0 0 0 0 0 0 0 0 0 0
0 0 0 0 0 0 0 0 0 0 0 0 0
0 0 0 0 0 0
"Viruses;Uroviricota;Caudoviricetes;Caudoviricetes__na;Autographiviridae;Autographiviridae__na;Aeromonas_phage_Aer_P220" 0 0 0 0 0 6 0 0 0 0 0
0 0 0 0 0 0 0 0 0 0 0 0 0
0 0 0 0 0 0 0 0 0 0 0 0 0
0 0 0 0 1 0 0 0 0 0 0 0 0
0 0 0 0 0 0 0 0 0 0 0 0 0
0 0 0 0 0 0 0 0 0
"Viruses;Uroviricota;Caudoviricetes;Caudoviricetes__na;Autographiviridae;Autographiviridae__na;Aeromonas_phage_JELG-KS1" 0 0 0 0 0 2 0 0 0 0 0
0 0 0 0 0 0 0 0 0 0 0 0 0
0 0 0 0 0 0 0 0 0 0 0 0 0
0 0 0 0 0 0 0 0 0 0 0 0 0
0 0 0 0 0 0 0 0 0 0 0 0 0
0 0 0 0 0 0 0 0 0
"Viruses;Uroviricota;Caudoviricetes;Caudoviricetes__na;Autographiviridae;Autographiviridae__na;Aeromonas_phage_PS" 0 2 3 9 3 27 0 0 0 0 0 0
0 0 1 0 5 0 0 0 0 0 0 0 0
0 0 0 0 0 0 0 0 0 0 0 0 0
0 0 0 0 0 0 1 0 0 0 0 0 0
0 0 0 0 0 0 0 0 0 0 0 0 0
0 0 0 0 0 0 0 0
"Viruses;Uroviricota;Caudoviricetes;Caudoviricetes__na;Autographiviridae;Autographiviridae__na;Aeromonas_phage_vB_AspA_Bolek" 0 0 0 1 0 0 0 0 0 0
0 0 0 0 0 0 0 0 0 0 0 0 0
0 0 0 0 0 0 0 0 0 0 0 0 0
0 0 0 0 0 0 0 0 0 0 0 4 0
0 0 0 0 0 0 0 0 0 0 0 0 0
0 0 0 0 0 0 0 0 0 0
"Viruses;Uroviricota;Caudoviricetes;Caudoviricetes__na;Autographiviridae;Autographiviridae__na;Aeromonas_phage_vB_AspA_Tola"0 0 0 0 4 0 0 0 0 0 0
0 0 0 0 0 0 0 0 0 0 0 0 0
0 0 0 0 0 0 0 0 0 0 0 0 0
0 0 0 0 0 0 0 0 0 0 0 0 0
0 0 0 0 0 0 0 0 0 0 0 0 0
0 0 0 0 0 0 0 0 0
"Viruses;Uroviricota;Caudoviricetes;Caudoviricetes__na;Autographiviridae;Autographiviridae__na;Autographiviridae_sp." 0 0 0 0 0 0 0 0 0 0 0 0
0 2 0 0 0 0 0 0 0 0 0 0 0
0 0 0 0 0 0 0 0 0 0 0 0 0
0 0 0 0 0 0 0 0 0 0 0 0 0

0 0 0 0 0 0 0 0 0 0 0 0 0
0 0 0 0 0 0 0 0
"Viruses;Uroviricota;Caudoviricetes;Caudoviricetes__na;Autographiviridae;Autographiviridae__na;Bacteriopha
ge_Phi_NF-1" 0 0 0 0 0 1 0 0 0 0 0 0
0 0 0 0 0 0 0 0 0 0 0 0 0
0 0 0 0 0 0 0 0 0 0 0 0 0
0 0 0 0 0 0 0 0 0 1 0 0 0
0 0 0 0 0 0 0 0 0 0 0 0 0
0 0 0 0 0 0 0 0
"Viruses;Uroviricota;Caudoviricetes;Caudoviricetes__na;Autographiviridae;Autographiviridae__na;Paracoccus_
phage_ParMal1" 0 0 0 0 0 1 0 0 0 0 0
0 0 0 0 0 0 0 0 0 0 0 0 0
0 0 0 0 0 0 0 0 0 0 0 0 0
0 0 0 0 0 0 0 0 0 0 0 0 0
0 0 0 0 0 0 0 0 0 0 0 0 0
0 0 0 0 0 0 0 0 0
"Viruses;Uroviricota;Caudoviricetes;Caudoviricetes__na;Autographiviridae;Autographiviridae__na;Ralstonia_p
hage_Reminis" 0 0 0 0 5 0 0 0 0 0 0 0
0 0 0 0 0 0 0 0 0 0 0 0 0
0 0 0 0 0 0 0 0 0 0 0 0 0
0 0 0 0 0 0 0 0 0 0 0 0 0
0 0 0 0 0 0 0 0 0 0 0 0 0
0 0 0 0 0 0 0 0
"Viruses;Uroviricota;Caudoviricetes;Caudoviricetes__na;Autographiviridae;Autographiviridae__na;Rhizobium_
phage_Paso" 0 0 0 1 0 0 0 0 0 0 0 0
0 0 0 0 0 0 0 0 0 0 0 0 0
0 0 0 0 0 0 0 0 0 0 0 0 0
0 0 0 0 0 0 0 0 0 0 0 0 0
0 0 0 0 0 0 0 0 0 0 0 0 0
0 0 0 0 0 0 0 0
"Viruses;Uroviricota;Caudoviricetes;Caudoviricetes__na;Autographiviridae;Autographiviridae__na;Sphaerotilu
s_phage_vB_SnaP-R1" 2 0 0 0 0 3 0 0 0 0 0
0 0 0 0 0 0 0 0 0 0 0 0 0
0 0 0 0 0 0 0 0 0 0 0 0 0
0 0 0 0 0 0 0 0 0 0 0 0 0
0 0 0 0 0 0 0 0 0 0 0 0 0
0 0 0 0 0 0 0 0 0
"Viruses;Uroviricota;Caudoviricetes;Caudoviricetes__na;Autographiviridae;Autographiviridae__na;Synechococ
cus_phage_S-SRP01" 0 0 0 2 0 10 0 0 0 0 0
0 0 0 0 0 0 1 0 0 0 0 0 0
0 0 0 0 0 0 0 0 0 0 0 0 0
0 0 0 0 0 0 0 0 0 0 0 0 0
0 0 0 0 0 0 0 0 0 0 0 0 0
0 0 0 0 0 0 0 2 0
"Viruses;Uroviricota;Caudoviricetes;Caudoviricetes__na;Autographiviridae;Autographiviridae__na;Vibrio_pha
ge_vB_VhaP_VH-5" 0 0 0 0 0 2 0 0 0 0 0
0 0 0 0 0 0 0 0 0 0 0 0 0
0 0 0 0 0 0 0 0 0 0 0 0 0
0 0 0 0 0 0 0 0 0 0 0 0 0

0 0 0 0 0 0 0 0 0 0 0 0 0
0 0 0 0 0 0 0 0 0
"Viruses;Uroviricota;Caudoviricetes;Caudoviricetes__na;Autographiviridae;Autographiviridae__na;Xylella_pha
ge_Cota" 0 1 0 0 1 0 0 0 0 0 0 0
0 0 0 0 0 0 0 0 0 0 0 0 0
0 0 0 0 0 0 0 0 0 0 0 0 0
0 0 0 0 0 0 0 0 0 0 0 0 0
0 0 0 0 0 0 0 0 0 0 0 0 0
0 0 0 0 0 0 0 0
"Viruses;Uroviricota;Caudoviricetes;Caudoviricetes__na;Autographiviridae;Autographiviridae__uc;Autographi
viridae__uc" 0 0 0 4 2 8 0 0 0 0 0 0
0 0 0 0 0 0 0 0 0 0 0 0 0
0 0 0 0 0 0 0 0 0 0 0 0 0
0 0 0 0 0 0 0 0 0 0 0 0 0
0 0 0 0 0 0 0 0 0 0 0 0 0
0 0 0 0 0 0 0 0
"Viruses;Uroviricota;Caudoviricetes;Caudoviricetes__na;Autographiviridae;Bifseptvirus;Bifseptvirus__uc"
0 0 0 0 0 11 0 0 0 0 0 0 0
0 0 0 0 0 0 0 0 0 0 0 0 0
0 0 0 0 0 0 0 0 0 0 0 0 0
0 0 0 0 0 0 0 0 0 0 0 0 0
0 0 0 0 0 0 0 0 0 0 0 0 0
0 0 0 0 0 0 0
"Viruses;Uroviricota;Caudoviricetes;Caudoviricetes__na;Autographiviridae;Cheungvirus;Cheungvirus_NATL1
A7" 0 0 0 0 0 0 0 0 0 0 0 0 0
0 0 0 0 0 0 0 0 0 0 0 0 0
0 0 0 0 0 0 0 0 0 0 0 0 0
0 0 0 0 0 0 0 0 0 0 0 0 0
0 0 0 0 0 0 0 1 0 0 0 0 0
0 0 0 0 0 0 0
"Viruses;Uroviricota;Caudoviricetes;Caudoviricetes__na;Autographiviridae;Cuernavacavirus;Cuernavacavirus_
_uc" 0 0 0 0 0 1 0 0 0 0 0 0 0
0 0 0 0 0 0 0 0 0 0 0 0 0
0 0 0 0 0 0 0 0 0 0 0 0 0
0 0 0 0 0 0 0 0 0 0 0 0 0
0 0 0 0 0 0 0 0 0 0 0 0 0
0 0 0 0 0 0 0
"Viruses;Uroviricota;Caudoviricetes;Caudoviricetes__na;Autographiviridae;Daemvirus;Daemvirus_acibel007"
0 0 0 0 0 0 0 0 0 0 0 0 0
0 0 0 0 0 0 0 0 0 0 0 0 0
0 0 0 0 0 0 0 0 0 0 0 0 0
0 0 0 0 0 0 0 0 0 0 0 0 0
0 0 0 0 0 0 0 0 0 0 0 0 0
0 1 0 0 0 0 0
"Viruses;Uroviricota;Caudoviricetes;Caudoviricetes__na;Autographiviridae;Ermolevavirus;Ermolevavirus__uc"
0 0 1 0 0 0 0 0 0 0 0 0 0
0 0 0 0 0 0 0 0 0 0 0 0 0
0 0 0 0 0 0 0 0 0 0 0 0 0
0 0 0 0 0 0 0 0 0 0 0 0 0

0 0 0 0 0 0 0 0 0 0 0 0 0
0 0 0 0 0 0 0
"Viruses;Uroviricota;Caudoviricetes;Caudoviricetes__na;Autographiviridae;Foturvirus;Foturvirus_H44" 0
0 0 0 0 4 0 0 0 0 0 0 0 0
0 0 0 0 0 0 0 0 0 0 0 0 0
0 0 0 0 0 0 0 0 0 0 0 0 0
0 0 0 0 0 0 0 0 0 0 0 0 0
0 0 0 0 0 0 0 0 0 0 0 0 0
0 0 0 0 0 0
"Viruses;Uroviricota;Caudoviricetes;Caudoviricetes__na;Autographiviridae;Friunavirus;Friunavirus__uc"
0 0 0 0 16 0 0 0 0 0 0 0 0
0 0 0 0 0 0 0 0 0 0 0 0 0
0 0 0 0 0 0 0 0 0 0 0 0 0
0 0 0 0 0 0 0 0 0 0 0 0 0
0 0 0 0 0 0 0 0 0 0 0 0 0
0 0 0 0 0 0 0
"Viruses;Uroviricota;Caudoviricetes;Caudoviricetes__na;Autographiviridae;Gyeongsanvirus;Gyeongsanvirus__
uc" 0 0 0 0 0 1 0 0 0 0 0 0 0
0 0 0 0 0 0 0 0 0 0 0 0 0
0 0 0 0 0 0 0 0 0 0 0 0 0
0 0 0 0 0 0 0 0 0 0 0 0 0
0 0 0 0 0 0 0 0 0 0 0 0 0
0 0 0 0 0 0 0
"Viruses;Uroviricota;Caudoviricetes;Caudoviricetes__na;Autographiviridae;Kafavirus;Kafavirus_SWcelC56"
0 0 0 0 1 0 0 0 0 0 0 0 0
0 0 0 0 0 0 0 0 0 0 0 0 0
0 0 0 0 0 0 0 0 0 0 0 0 0
0 0 0 0 0 0 0 0 0 0 0 0 0
0 0 0 0 0 0 0 0 0 0 0 0 0
0 0 0 0 0 0 0
"Viruses;Uroviricota;Caudoviricetes;Caudoviricetes__na;Autographiviridae;Kalppathivirus;Kalppathivirus_P26
059B" 1 0 5 11 41 4 0 0 0 0 0 0 0
0 0 0 0 0 0 0 0 0 0 0 0 0
0 0 0 0 0 0 0 0 0 1 0 0 0
0 0 6 0 0 0 0 0 0 0 0 0 0
0 0 0 0 0 0 0 0 0 0 0 0 0
0 0 0 0 0 0 0
"Viruses;Uroviricota;Caudoviricetes;Caudoviricetes__na;Autographiviridae;Kelmasvirus;Kelmasvirus_RSB2"
0 0 0 1 0 0 0 0 0 0 0 0 0
0 0 0 0 0 0 0 0 0 0 0 0 0
0 0 0 0 0 0 0 0 0 0 0 0 0
0 0 0 0 0 0 0 0 0 0 0 0 0
0 0 0 0 0 0 0 0 0 0 0 0 0
0 0 0 0 0 0 0
"Viruses;Uroviricota;Caudoviricetes;Caudoviricetes__na;Autographiviridae;Pifdecavirus;Pseudomonas_phage_
PSA6" 0 0 0 0 0 0 0 0 1 0 0 0 0
0 0 0 0 0 0 0 0 0 0 0 0 0
0 0 0 0 0 0 0 0 0 0 0 0 0
0 0 0 0 0 0 0 0 0 0 0 0 0

0 0 0 0 0 0 0 0 0 0 0 0 0
0 0 0 0 0 0 0
"Viruses;Uroviricota;Caudoviricetes;Caudoviricetes__na;Autographiviridae;Pijolavirus;Pijolavirus__uc" 0
0 0 0 0 4 0 0 0 0 0 0 0 0
0 0 0 0 0 0 0 0 0 0 0 0 0
0 0 0 0 0 0 0 0 0 0 0 0 0
0 0 0 0 0 0 0 0 0 0 0 0 0
0 0 0 0 0 0 0 0 0 0 0 0 0
0 0 0 0 0 0
"Viruses;Uroviricota;Caudoviricetes;Caudoviricetes__na;Autographiviridae;Pradovirus;Bacteriophage_Titan-X"
0 0 0 1 0 0 0 0 0 0 0 0 0
0 0 0 0 0 0 0 0 0 0 0 0 0
0 0 0 0 0 0 0 0 0 0 0 0 0
0 0 0 0 0 0 0 0 0 0 0 0 0
0 0 0 0 0 0 0 0 0 0 0 0 0
0 0 0 0 0 0 0
"Viruses;Uroviricota;Caudoviricetes;Caudoviricetes__na;Autographiviridae;Przondovirus;Przondovirus__uc"
0 0 0 0 0 0 0 0 0 0 0 0 0
0 0 0 0 0 0 0 0 0 0 0 0 0
0 0 0 0 0 0 0 0 0 0 0 0 0
0 0 2 0 0 0 0 0 0 0 0 0 0
0 0 0 0 0 0 0 0 0 0 0 0 0
0 0 0 0 0 0 0
"Viruses;Uroviricota;Caudoviricetes;Caudoviricetes__na;Autographiviridae;Qadamvirus;Qadamvirus_SB28"
0 0 0 0 8 0 0 0 0 0 0 0 0
0 0 0 0 0 0 0 0 0 0 0 0 0
0 0 0 0 0 0 0 0 0 0 0 0 0
0 0 3 0 0 0 0 0 0 0 0 0 0
0 0 0 0 0 0 0 0 0 0 0 0 0
0 0 0 0 0 0 0
"Viruses;Uroviricota;Caudoviricetes;Caudoviricetes__na;Autographiviridae;Stompelvirus;Stompelvirus_RPSC1"
0 0 0 1 0 0 0 0 0 0 0 0 0
0 0 0 0 0 0 0 0 0 0 0 0 0
0 0 0 0 0 0 0 0 0 0 0 0 0
0 0 0 0 0 0 0 0 0 0 0 0 0
0 0 0 0 0 0 0 0 0 0 0 0 0
0 0 0 0 0 0 0
"Viruses;Uroviricota;Caudoviricetes;Caudoviricetes__na;Autographiviridae;Stubburvirus;Stubburvirus_LKA1"
0 0 0 7 0 0 0 0 0 21 0 0 0
0 0 0 0 0 0 0 0 0 0 0 0 0
0 0 0 0 0 0 0 0 0 0 0 0 0
0 0 0 0 0 4 0 117 0 0 0 0 0
0 0 0 0 1 0 0 0 0 0 0 0 0
0 0 0 0 0 0 0
"Viruses;Uroviricota;Caudoviricetes;Caudoviricetes__na;Autographiviridae;Tangaroavirus;Tangaroavirus_tv951
510a" 0 0 0 0 2 0 0 0 0 0 0 0 0
0 0 0 0 0 0 0 0 0 0 0 0 0
0 0 0 0 0 0 0 0 0 0 0 0 0
0 0 0 0 0 0 0 0 1 0 0 0 0

0 0 0 0 0 0 0 0 0 0 0 0 0
0 0 0 0 0 0 0
"Viruses;Uroviricota;Caudoviricetes;Caudoviricetes__na;Autographiviridae;Teetrevirus;Enterobacter_phage_EN
C18" 0 0 0 1 0 0 0 0 0 0 0 0 0
0 0 0 0 0 0 0 0 0 0 0 0 0
0 0 0 0 0 0 0 0 0 0 0 0 0
0 0 0 0 0 0 0 0 1 0 0 0 0
0 0 0 0 0 0 0 0 0 0 0 0 0
0 0 0 0 0 0 0
"Viruses;Uroviricota;Caudoviricetes;Caudoviricetes__na;Autographiviridae;Teetrevirus;Teetrevirus__uc" 0
0 0 0 0 0 0 0 0 0 0 0 0 0
0 0 0 0 0 0 0 0 0 0 0 0 0
0 0 0 0 0 0 0 0 0 0 0 0 0
0 0 0 0 0 0 0 0 0 0 0 0 0
0 0 0 0 0 0 0 2 0 0 0 0 0
0 0 0 0 0 0
"Viruses;Uroviricota;Caudoviricetes;Caudoviricetes__na;Autographiviridae;Teetrevirus;Teetrevirus_ECA2"
0 0 0 0 0 0 0 0 0 0 0 0 0
0 0 0 0 0 0 0 0 0 0 0 0 0
0 0 0 0 0 0 0 0 1 0 0 0 0
0 0 0 0 0 0 0 0 0 0 0 0 0
0 0 0 0 0 0 0 0 0 0 0 0 0
0 0 0 0 0 0 0
"Viruses;Uroviricota;Caudoviricetes;Caudoviricetes__na;Autographiviridae;Tiamatvirus;Tiamatvirus_PSSP7"
0 0 0 1 0 0 0 0 0 0 0 0 0
0 0 0 0 0 0 0 0 0 0 0 0 0
0 0 0 0 0 0 0 0 0 0 0 0 0
0 0 0 0 0 0 0 0 1 0 0 0 0
0 0 0 0 0 0 0 0 0 0 0 0 0
0 0 0 0 0 0 0
"Viruses;Uroviricota;Caudoviricetes;Caudoviricetes__na;Autographiviridae;Tiilvirus;Tiilvirus_P60" 0
0 0 0 1 0 0 0 0 0 0 0 0 0
0 0 0 0 0 0 0 0 0 0 0 0 0
0 0 0 0 0 0 0 0 0 0 0 0 0
0 0 0 0 0 0 0 0 0 0 0 0 0
0 0 0 0 0 0 0 0 0 0 0 0 0
0 0 0 0 0 0
"Viruses;Uroviricota;Caudoviricetes;Caudoviricetes__na;Autographiviridae;Waewaevirus;Waewaevirus_limeze
ro" 0 0 0 0 5 0 0 0 0 0 0 0 0
0 0 0 0 0 0 0 0 0 0 0 0 0
0 0 0 0 0 0 0 0 0 0 0 0 0
0 0 0 0 0 0 0 0 0 0 0 0 0
0 0 0 0 0 0 0 0 0 0 0 0 0
0 0 0 0 0 0 0
"Viruses;Uroviricota;Caudoviricetes;Caudoviricetes__na;Autographiviridae;Warsawvirus;Warsawvirus_3MF5"
0 0 0 0 0 0 0 0 0 0 0 0 0
0 0 0 0 0 0 0 0 0 0 0 0 0
0 0 0 0 0 0 0 0 0 0 0 0 0
0 0 0 0 0 0 0 0 0 0 0 0 0

0 1 0 0 0 0 0 0 0 0 0 0 0
0 0 0 0 0 0 0
"Viruses;Uroviricota;Caudoviricetes;Caudoviricetes__na;Casjensviridae;Casjensviridae__na;Rhodobacter_phage
_RcCWillis" 0 0 0 2 0 0 0 0 0 0 0 0
0 0 0 0 0 0 0 0 0 0 0 0 0
0 0 0 0 0 0 0 0 0 0 0 0 0
0 0 0 0 0 0 3 0 0 0 0 0 0
0 0 0 0 0 0 0 0 0 0 0 0 0
0 0 0 0 0 0 0 0
"Viruses;Uroviricota;Caudoviricetes;Caudoviricetes__na;Casjensviridae;Casjensviridae__uc;Casjensviridae__uc"
0 0 0 2 0 12 0 0 0 0 0 0 0
0 0 0 0 0 0 0 0 0 0 0 0 0
0 0 0 0 0 0 0 0 0 0 0 0 0
0 0 0 0 0 0 0 0 0 0 0 0 0
0 0 0 0 0 0 0 0 0 0 0 0 0
0 0 0 0 0 0 0
"Viruses;Uroviricota;Caudoviricetes;Caudoviricetes__na;Casjensviridae;Dunedinvirus;Dunedinvirus_JS26"
0 0 0 0 0 0 0 0 0 0 0 0 0
0 0 0 0 0 0 0 0 0 0 0 0 0
0 0 0 0 0 0 0 0 0 0 0 0 0
0 0 0 0 0 0 0 0 0 0 0 1 0
0 0 0 0 0 0 0 0 0 0 0 0 0
0 0 0 0 0 0 0
"Viruses;Uroviricota;Caudoviricetes;Caudoviricetes__na;Casjensviridae;Gediminasvirus;Gediminasvirus_AchV
4" 0 0 0 0 0 0 0 0 0 0 0 0 0
0 0 0 0 0 0 0 0 0 0 0 0 0
0 0 0 0 0 0 0 0 0 0 0 0 0
0 0 0 0 0 0 0 0 0 0 0 0 0
0 0 0 0 0 1 0 0 0 0 0 0 0
0 0 0 0 0 0 0
"Viruses;Uroviricota;Caudoviricetes;Caudoviricetes__na;Casjensviridae;Nazgulvirus;Burkholderia_virus_BcepN
azgul" 0 0 0 0 0 3 0 0 0 0 0 0 0
0 0 0 0 0 0 0 0 0 0 0 0 0
0 0 0 0 0 0 0 0 0 0 0 0 0
0 0 0 0 0 0 0 0 0 0 0 0 0
0 0 0 0 0 0 0 0 0 0 0 0 0
0 0 0 0 0 0 0
"Viruses;Uroviricota;Caudoviricetes;Caudoviricetes__na;Casjensviridae;Phobosvirus;Phobosvirus_phobos"
0 0 0 0 0 0 0 0 0 0 0 0 0
0 0 0 0 0 0 0 0 0 0 0 0 0
0 0 0 0 0 0 0 0 0 0 0 0 0
0 0 0 0 0 0 0 0 1 0 0 0 0
0 0 0 0 0 0 0 0 0 0 0 0 0
0 0 0 0 0 0 0
"Viruses;Uroviricota;Caudoviricetes;Caudoviricetes__na;Casjensviridae;Salvovirus;Salvovirus_bacata" 0
0 0 0 0 0 0 0 0 1 0 0 0 0
0 0 0 0 0 0 0 0 0 0 0 0 0
0 0 0 0 0 0 0 0 0 0 0 0 0
0 0 0 0 0 0 0 0 0 0 0 0 0

0 0 0 0 0 0 0 0 0 0 0 0 0
0 0 0 0 0 0
"Viruses;Uroviricota;Caudoviricetes;Caudoviricetes__na;Casjensviridae;Sanovirus;Sanovirus__uc" 0
0 0 0 0 3 0 0 0 0 0 0 0 0
0 0 0 0 0 0 0 0 0 0 0 0 0
0 0 0 0 0 0 0 0 0 0 0 0 0
0 0 0 0 0 0 0 0 0 0 0 0 0
0 0 0 0 0 0 0 0 0 0 0 0 0
0 0 0 0 0 0
"Viruses;Uroviricota;Caudoviricetes;Caudoviricetes__na;Caudoviricetes__na;Akonivirus;Akonivirus__uc"
0 0 0 0 0 10 0 0 0 0 0 0 0
0 0 0 0 0 0 0 0 0 0 0 0 0
0 0 0 0 0 0 0 0 0 0 0 0 0
0 0 0 0 0 0 0 0 0 0 0 0 0
0 0 0 0 0 0 0 0 0 0 0 0 0
0 0 0 0 0 0 0
"Viruses;Uroviricota;Caudoviricetes;Caudoviricetes__na;Caudoviricetes__na;Alachuavirus;Alachuavirus_Xp15"
0 0 0 0 0 0 0 0 2 0 0 0 0
0 0 0 0 0 0 0 0 0 0 0 0 0
0 0 0 0 0 0 0 0 0 3 0 0 0
0 0 0 0 0 0 0 0 0 0 0 0 1
0 2 0 0 0 0 0 0 0 0 0 0 0
0 0 0 0 0 0 0
"Viruses;Uroviricota;Caudoviricetes;Caudoviricetes__na;Caudoviricetes__na;Alcyoneusvirus;Klebsiella_phage_
KP13-7"0 0 0 0 5 0 0 0 0 0 0 0 0
0 0 0 0 0 0 0 0 0 0 0 0 0
0 0 0 0 0 0 0 0 0 0 0 0 0
0 0 0 0 0 0 0 0 0 0 0 0 0
0 0 0 0 0 0 0 0 0 0 0 0 0
0 0 0 0 0 0 0
"Viruses;Uroviricota;Caudoviricetes;Caudoviricetes__na;Caudoviricetes__na;Alegriavirus;Alegriavirus_av2B8"
0 0 0 0 0 0 0 0 0 0 0 0 0
0 0 0 0 0 0 0 0 0 0 0 0 0
0 0 0 0 0 0 0 0 0 0 0 0 0
0 0 0 0 0 0 0 0 0 0 0 0 0
0 0 0 0 0 0 0 0 1 0 0 0 0
0 0 0 0 0 0 0
"Viruses;Uroviricota;Caudoviricetes;Caudoviricetes__na;Caudoviricetes__na;Amoyvirus;Amoyvirus_R7M"
0 0 0 1 0 0 0 0 0 0 0 0 0
0 0 0 0 0 0 0 0 0 0 0 0 0
0 0 0 0 0 0 0 0 0 0 0 0 0
0 0 0 0 0 0 0 0 0 0 0 0 0
0 0 0 0 0 0 0 0 0 0 0 0 0
0 0 0 0 0 0 0
"Viruses;Uroviricota;Caudoviricetes;Caudoviricetes__na;Caudoviricetes__na;Andrewvirus;Arthrobacter_phage_
Renna12" 0 0 0 0 0 0 0 0 0 0 0 0
0 0 0 0 0 0 0 0 0 0 0 0 0
0 0 0 0 0 0 0 0 0 6 0 0 0
0 0 0 0 0 0 0 0 0 0 0 0 0

0 0 0 0 0 0 0 0 0 0 0 0 0
0 0 0 0 0 0 0 0
"Viruses;Uroviricota;Caudoviricetes;Caudoviricetes__na;Caudoviricetes__na;Anjalivirus;Anjalivirus_anjali"
0 0 0 0 1 2 0 0 0 0 0 0 0
0 0 0 0 0 0 0 0 0 0 0 0 0
0 0 0 0 0 0 0 0 0 0 0 0 0
0 0 0 0 0 0 0 0 0 0 0 0 0
0 0 0 0 0 0 0 0 0 0 0 0 0
0 0 0 0 0 0 0
"Viruses;Uroviricota;Caudoviricetes;Caudoviricetes__na;Caudoviricetes__na;Armstrongvirus;Armstrongvirus_a
rmstrong" 0 0 0 1 0 0 0 0 0 0 0 0
0 0 0 0 0 0 0 0 0 0 0 0 0
0 0 0 0 0 0 0 0 0 0 0 0 0
0 0 0 0 0 0 0 0 0 0 0 0 0
0 0 0 0 0 0 0 0 0 0 0 0 0
0 0 0 0 0 0 0 0
"Viruses;Uroviricota;Caudoviricetes;Caudoviricetes__na;Caudoviricetes__na;Armstrongvirus;Microbacterium_p
hage_Franklin22" 0 0 0 0 0 0 0 0 0 0 0
0 0 0 0 0 0 0 0 0 0 0 0 0
0 0 0 0 0 0 0 0 0 0 1 0 0
0 0 0 0 0 0 0 0 0 0 0 0 0
0 0 0 0 0 0 0 0 0 0 0 0 0
0 0 0 0 0 0 0 0 0
"Viruses;Uroviricota;Caudoviricetes;Caudoviricetes__na;Caudoviricetes__na;Astrithrvirus;Pectobacterium_phag
e_DU_PP_III" 0 0 0 0 0 0 0 0 0 0 0 0
0 0 0 0 0 0 0 0 0 0 0 0 0
0 0 0 0 0 0 0 0 0 0 0 0 0
0 0 0 0 0 0 0 0 0 0 0 0 0
0 0 0 0 1 0 0 0 0 0 0 0 0
0 0 0 0 0 0 0 0
"Viruses;Uroviricota;Caudoviricetes;Caudoviricetes__na;Caudoviricetes__na;Audreyjarvisvirus;Audreyjarvisvir
us_AM4" 0 0 0 0 0 0 0 0 0 0 0 0
0 0 0 0 0 0 0 0 0 0 0 0 0
0 0 0 0 0 0 0 0 0 0 0 0 0
0 0 0 0 0 0 0 0 0 0 0 0 0
0 0 0 1 1 0 0 0 0 0 0 0 0
0 0 0 0 0 0 0 0
"Viruses;Uroviricota;Caudoviricetes;Caudoviricetes__na;Caudoviricetes__na;Baikalvirus;Baikalvirus_PaBG"
0 0 0 0 0 0 0 0 0 0 0 0 0
0 0 0 0 0 0 0 0 0 0 0 0 0
0 0 0 0 0 0 0 0 0 1 0 0 0
0 0 0 0 0 0 0 0 0 0 0 0 0
0 0 0 0 0 0 0 0 0 0 0 0 0
0 0 0 0 0 0 0
"Viruses;Uroviricota;Caudoviricetes;Caudoviricetes__na;Caudoviricetes__na;Bantamvirus;Bantamvirus_bantam
" 0 0 0 0 6 0 0 0 0 0 0 0 0
0 0 0 0 0 0 0 0 0 0 0 0 0
0 0 0 0 0 0 0 0 0 0 0 0 0
0 0 0 0 0 0 0 0 0 0 0 0 0

0 0 0 0 0 0 0 0 0 0 0 0 0
0 0 0 0 0 0 0
"Viruses;Uroviricota;Caudoviricetes;Caudoviricetes__na;Caudoviricetes__na;Barbavirus;Barbavirus__uc" 0
0 1 1 2 3 0 0 0 0 0 0 0 0
0 0 0 0 0 0 0 0 0 1 0 0 0
0 0 0 0 0 0 0 0 2 0 0 0 0
0 2 0 0 0 0 0 6 0 0 0 1 0
0 0 0 0 0 0 0 0 0 0 0 0 0
0 0 0 0 0 0
"Viruses;Uroviricota;Caudoviricetes;Caudoviricetes__na;Caudoviricetes__na;Barbavirus;Barbavirus_barba19A"
0 1 0 0 1 0 0 0 0 0 0 0 0
0 0 0 0 0 0 0 0 0 0 0 0 0
0 0 0 0 0 0 0 0 0 0 0 0 0
0 0 0 0 0 0 0 0 0 0 0 0 0
0 0 0 0 0 0 0 0 0 0 0 0 0
0 0 0 0 0 0 0
"Viruses;Uroviricota;Caudoviricetes;Caudoviricetes__na;Caudoviricetes__na;Bendigovirus;Bendigovirus_GMA6
" 0 0 0 0 0 0 0 0 0 1 0 0 0
0 0 0 0 0 0 0 0 0 0 0 0 0
0 0 0 0 0 0 0 0 0 0 0 0 0
0 0 0 0 0 0 0 0 0 0 0 0 0
0 0 0 0 0 0 0 0 0 0 0 0 0
0 0 0 0 0 0 0
"Viruses;Uroviricota;Caudoviricetes;Caudoviricetes__na;Caudoviricetes__na;Beograduvirus;Xanthomonas_pha
ge_MYK3" 0 0 0 1 0 0 0 0 0 0 0 0
0 0 0 0 0 0 0 0 0 0 0 0 0
0 0 0 0 0 0 0 0 0 0 0 0 0
0 0 0 0 0 0 0 0 0 0 0 0 0
0 0 0 0 0 0 0 0 0 0 0 0 0
0 0 0 0 0 0 0 0
"Viruses;Uroviricota;Caudoviricetes;Caudoviricetes__na;Caudoviricetes__na;Bertelyvirus;Bertelyvirus__uc"
0 0 0 0 8 0 0 0 0 0 0 0 0
0 0 0 0 0 0 0 0 0 0 0 0 0
0 0 0 0 0 0 0 0 0 0 0 0 0
0 0 0 0 0 0 0 0 0 0 0 0 0
0 0 0 0 0 0 0 0 0 0 0 0 0
0 0 0 0 0 0 0
"Viruses;Uroviricota;Caudoviricetes;Caudoviricetes__na;Caudoviricetes__na;Bievrevirus;Bievrevirus_bv4A7"
0 0 0 0 0 0 0 0 0 0 0 0 0
0 0 0 0 0 0 0 0 0 0 0 0 0
0 0 0 0 0 0 0 0 0 0 0 0 0
0 0 0 0 0 0 0 0 0 0 0 0 0
0 0 0 0 0 0 0 0 4 4 0 0 0
0 0 0 0 0 0 0
"Viruses;Uroviricota;Caudoviricetes;Caudoviricetes__na;Caudoviricetes__na;Bievrevirus;Escherichia_phage_3W
" 0 0 0 0 0 0 0 0 0 0 0 0 0
0 0 0 0 0 0 0 0 0 0 0 0 0
0 0 0 0 0 0 0 0 0 0 0 0 0
0 0 0 0 0 0 0 0 0 0 0 0 0

0 0 0 0 0 0 0 0 5 1 0 0 0
0 0 0 0 0 0 0
"Viruses;Uroviricota;Caudoviricetes;Caudoviricetes__na;Caudoviricetes__na;Bingvirus;Bingvirus__uc" 0
0 0 0 0 0 0 0 0 0 0 0 0 0
0 0 0 0 0 0 0 0 0 0 0 0 0
0 0 0 0 0 0 0 0 0 0 0 0 0
0 0 0 0 0 0 0 1 0 0 0 0 0
0 0 0 0 0 0 0 0 0 0 0 0 0
0 0 0 0 0 0
"Viruses;Uroviricota;Caudoviricetes;Caudoviricetes__na;Caudoviricetes__na;Bingvirus;Streptomyces_phage_Cl
ubPenguin" 0 0 0 0 5 0 0 0 0 0 0 0
0 0 0 1 0 1 0 0 0 0 0 0 0
0 0 0 0 0 0 0 0 0 0 0 0 0
0 0 0 0 0 0 0 0 0 9 0 0 0
0 0 0 0 0 0 0 0 0 0 0 0 0
0 0 0 0 0 0 0 0
"Viruses;Uroviricota;Caudoviricetes;Caudoviricetes__na;Caudoviricetes__na;Bingvirus;Streptomyces_phage_Ka
rdashian" 0 0 0 0 6 1 0 0 0 0 0 0
0 0 0 0 0 0 0 0 0 0 0 0 0
0 0 0 0 0 0 0 0 0 0 0 0 0
0 0 0 0 0 0 0 0 0 0 0 0 0
0 0 0 0 0 0 0 0 0 0 0 0 0
0 0 0 0 0 0 0 0
"Viruses;Uroviricota;Caudoviricetes;Caudoviricetes__na;Caudoviricetes__na;Bingvirus;Streptomyces_phage_Ssc
P1EGY"0 0 0 0 0 0 0 0 0 0 0 0 0
0 0 0 0 0 0 0 0 0 0 0 0 0
0 0 0 0 0 0 0 0 0 0 0 0 0
0 0 0 0 0 0 0 0 0 0 0 0 0
0 0 0 0 0 0 0 0 0 0 0 0 0
0 0 0 0 0 1 0
"Viruses;Uroviricota;Caudoviricetes;Caudoviricetes__na;Caudoviricetes__na;Bjornvirus;Bjornvirus_bjorn"
0 0 0 0 1 0 0 0 0 0 0 0 0
0 0 0 0 0 0 0 0 0 0 0 0 0
0 0 0 0 0 0 0 0 0 0 0 0 0
0 0 0 0 0 2 0 0 0 0 0 0 0
0 0 0 0 0 0 0 0 0 0 0 0 0
0 0 0 0 0 0 0
"Viruses;Uroviricota;Caudoviricetes;Caudoviricetes__na;Caudoviricetes__na;Borockvirus;Borockvirus_brock"
0 0 0 1 0 1 0 0 0 0 0 0 0
2 0 0 0 0 0 0 0 0 0 0 0 0
0 0 0 0 0 0 0 0 0 0 0 0 0
0 0 0 0 0 0 0 0 0 0 0 0 0
0 0 0 0 0 0 0 0 0 0 0 0 0
0 0 0 0 0 0 0
"Viruses;Uroviricota;Caudoviricetes;Caudoviricetes__na;Caudoviricetes__na;Brigitvirus;Brigitvirus_brigit"
0 0 0 0 0 0 0 0 0 0 0 0 0
0 0 0 0 0 0 0 0 0 0 0 0 0
0 0 0 0 0 1 0 0 0 0 0 0 0
0 0 0 0 0 0 0 0 0 14 15 0 0

0 0 0 0 0 0 0 0 1 1 0 0 0
0 1 0 0 0 0 0
"Viruses;Uroviricota;Caudoviricetes;Caudoviricetes__na;Caudoviricetes__na;Britbratvirus;Britbratvirus_britbrat
" 0 0 0 0 0 0 0 0 0 1 0 0 0
0 0 0 0 0 0 0 0 0 0 0 0 0
0 0 0 0 0 0 0 0 0 0 0 0 0
0 0 0 0 0 0 0 0 0 0 0 0 0
0 0 0 0 0 0 0 0 0 0 0 0 0
0 0 0 0 0 0 0
"Viruses;Uroviricota;Caudoviricetes;Caudoviricetes__na;Caudoviricetes__na;Brussowvirus;Brussowvirus_bvO1
205" 0 0 0 0 0 0 0 0 0 0 0 0 0
0 0 0 0 0 0 0 0 0 0 0 0 0
0 0 0 0 0 0 0 0 0 0 1 0 0
0 0 0 0 0 0 0 0 0 0 0 0 0
0 0 0 0 0 0 0 0 0 0 0 0 0
0 0 0 0 0 0 0
"Viruses;Uroviricota;Caudoviricetes;Caudoviricetes__na;Caudoviricetes__na;Brussowvirus;Streptococcus_phag
e_P7953" 0 0 0 0 0 0 0 0 0 0 0 0
0 0 0 0 0 0 0 0 0 0 0 0 0
0 0 0 0 0 0 0 0 0 0 0 0 0
0 0 0 0 0 0 0 0 0 0 0 0 0
0 0 0 0 1 0 0 0 0 0 0 0 0
0 0 0 0 0 0 0 0
"Viruses;Uroviricota;Caudoviricetes;Caudoviricetes__na;Caudoviricetes__na;Bruynoghevirus;Bruynoghevirus__
uc" 0 0 0 1 0 0 0 0 0 0 0 0 0
0 0 0 0 0 0 0 0 0 0 0 0 0
0 0 0 0 0 0 0 0 0 0 0 0 0
0 0 0 0 0 0 0 0 0 0 0 0 0
0 0 0 0 0 0 0 0 0 0 0 0 0
0 0 0 0 0 0 0
"Viruses;Uroviricota;Caudoviricetes;Caudoviricetes__na;Caudoviricetes__na;Burrovirus;Burrovirus__uc" 0
0 4 0 0 0 0 0 0 0 0 0 0 0
0 0 0 0 0 0 0 0 0 0 0 0 0
0 0 0 0 0 0 0 0 0 0 0 0 0
0 0 0 0 0 0 0 0 0 0 0 0 0
0 0 0 0 0 0 0 0 0 0 0 0 0
0 0 0 0 0 0
"Viruses;Uroviricota;Caudoviricetes;Caudoviricetes__na;Caudoviricetes__na;Burrovirus;Burrovirus_arete"
0 0 0 0 0 0 0 0 1 0 0 0 0
0 0 0 0 0 0 0 0 0 0 0 0 0
0 0 0 0 0 0 0 0 0 0 0 0 0
0 0 0 0 0 0 0 0 0 0 0 0 0
0 0 0 0 0 0 0 0 0 0 0 0 0
0 0 0 0 0 0 0
"Viruses;Uroviricota;Caudoviricetes;Caudoviricetes__na;Caudoviricetes__na;Burrovirus;Burrovirus_burro"
0 0 0 0 0 0 0 0 2 0 0 0 0
0 0 0 0 0 0 0 0 0 0 0 0 0
0 0 0 0 0 0 0 0 0 0 0 0 0
0 0 0 0 0 0 0 0 0 0 0 0 0

0 0 0 0 0 0 0 0 0 0 0 0 0
0 0 0 0 0 0 0
"Viruses;Uroviricota;Caudoviricetes;Caudoviricetes__na;Caudoviricetes__na;Busanvirus;Busanvirus_ACP17"
0 0 0 1 7 7 0 0 0 0 0 0 0
0 0 0 0 0 0 0 0 0 0 0 0 0
0 0 0 0 0 0 0 0 0 0 0 0 0
0 0 0 0 0 0 0 0 0 0 0 0 0
0 0 0 0 0 0 0 0 0 0 0 0 0
0 0 0 0 0 0 0
"Viruses;Uroviricota;Caudoviricetes;Caudoviricetes__na;Caudoviricetes__na;Carmenvirus;Carmenvirus__uc"
0 0 0 0 0 4 0 0 0 0 0 0 0
0 0 0 0 0 0 0 0 0 0 0 0 0
0 0 0 0 0 0 0 0 0 0 0 0 0
0 0 0 0 0 0 0 0 0 0 0 0 0
0 0 0 0 0 0 0 0 0 0 0 0 0
0 0 0 0 0 0 0
"Viruses;Uroviricota;Caudoviricetes;Caudoviricetes__na;Caudoviricetes__na;Casadabanvirus;Pseudomonas_ph
age_ZCPA1" 0 0 0 0 0 0 0 0 0 0 1 0
0 0 0 0 0 0 0 0 0 0 0 0 0
0 0 0 1 0 0 0 0 0 0 0 0 0
0 0 0 0 0 0 0 0 0 0 0 0 0
0 0 0 0 0 0 0 0 0 0 0 0 1
0 0 0 0 0 0 0 0
"Viruses;Uroviricota;Caudoviricetes;Caudoviricetes__na;Caudoviricetes__na;Caudoviricetes__na;Acaryochloris
_phage_A-HIS1" 0 0 0 0 0 0 0 0 0 0 0
0 0 0 0 0 0 0 0 0 0 0 0 0
0 0 0 0 0 0 0 0 0 0 0 0 0
1 0 0 0 0 0 0 0 0 0 0 0 0
0 0 0 0 0 0 0 0 0 0 0 0 0
0 0 0 0 0 0 0 0 0
"Viruses;Uroviricota;Caudoviricetes;Caudoviricetes__na;Caudoviricetes__na;Caudoviricetes__na;Acinetobacter_
phage_BS46" 0 0 0 0 0 0 0 0 0 0 0 0
0 0 0 0 0 0 0 0 0 0 0 0 0
0 0 0 0 0 0 0 0 0 0 0 0 0
0 0 0 0 0 0 0 0 0 1 0 0 0
0 0 0 0 0 0 0 0 0 0 0 0 0
0 0 0 0 0 0 0 0
"Viruses;Uroviricota;Caudoviricetes;Caudoviricetes__na;Caudoviricetes__na;Caudoviricetes__na;Acinetobacter_
phage_MD-2021a" 0 0 0 3 0 0 0 0 0 0 0
0 0 0 0 0 0 0 0 0 0 0 0 0
0 0 0 0 0 0 0 0 0 0 0 0 0
0 0 0 0 0 0 0 0 0 0 0 0 0
0 0 0 0 0 0 0 0 0 0 0 0 0
0 0 0 0 0 0 0 0 0
"Viruses;Uroviricota;Caudoviricetes;Caudoviricetes__na;Caudoviricetes__na;Caudoviricetes__na;Acinetobacter_
phage_phiAC-1" 0 0 0 1 1 0 0 0 0 0 0
0 0 0 0 0 0 0 0 0 0 0 1 0
0 0 0 0 0 0 0 0 0 0 0 0 0
0 0 0 0 0 0 0 0 0 0 0 0 0

0	0	0	0	0	0	0	0	0	0	0	0	0
0	0	0	0	0	0	0	0	0
"Viruses;Uroviricota;Caudoviricetes;Caudoviricetes__na;Caudoviricetes__na;Caudoviricetes__na;Acinetobacter_phage_TaPaz"	0	0	0	0	0	1	0	0	0	0	0	0
0	0	0	0	0	0	0	0	0	0	0	0	0
0	0	0	0	0	0	0	0	0	0	0	0	0
0	0	0	0	0	0	0	0	0	2	0	0	0
0	0	0	0	0	0	0	0	0	0	0	0	0
0	0	0	0	0	0	0	0
"Viruses;Uroviricota;Caudoviricetes;Caudoviricetes__na;Caudoviricetes__na;Caudoviricetes__na;Actinoplanes_phage_phiAsp2"	0	0	0	0	0	0	0	0	0	0	0
0	0	0	0	0	0	0	0	0	1	0	0	0
0	0	0	0	0	0	0	0	0	0	0	0	0
0	0	0	0	0	0	0	0	0	0	0	0	0
0	0	0	0	0	0	0	0	0	0	0	0	0
0	0	0	0	0	0	0	0	0
"Viruses;Uroviricota;Caudoviricetes;Caudoviricetes__na;Caudoviricetes__na;Caudoviricetes__na;Aeromonas_phage_4_4512"	0	0	4	9	42	13	0	0	0	0	0	0
0	0	0	0	2	0	0	0	0	0	0	0	0
0	0	0	0	0	0	0	0	0	0	0	0	0
0	0	0	0	0	0	0	0	0	0	0	0	0
0	0	0	0	0	0	0	0	0	0	0	0	0
0	0	0	0	0	0	0	0
"Viruses;Uroviricota;Caudoviricetes;Caudoviricetes__na;Caudoviricetes__na;Caudoviricetes__na;Aeromonas_phage_62AhydR11PP"	0	1	1	2	0	0	0	0	0	0	0
0	0	0	0	0	0	0	0	0	0	0	0	0
0	0	0	0	0	0	0	0	0	0	0	0	0
0	0	0	0	0	0	0	0	0	0	0	0	0
0	0	0	0	0	0	0	0	0	0	0	0	0
0	0	0	0	0	0	0	0	0
"Viruses;Uroviricota;Caudoviricetes;Caudoviricetes__na;Caudoviricetes__na;Caudoviricetes__na;Aeromonas_phage_CF8"	0	0	0	0	0	0	0	0	0	0	0	0
0	0	0	0	0	0	0	0	0	0	0	0	0
0	0	0	0	0	0	0	0	0	0	0	0	0
0	0	2	0	0	0	0	0	0	0	0	0	0
0	0	0	0	0	0	0	0	0	0	0	0	0
0	0	0	0	0	0	0	0
"Viruses;Uroviricota;Caudoviricetes;Caudoviricetes__na;Caudoviricetes__na;Caudoviricetes__na;Aeromonas_phage_HJ05"	0	0	0	1	0	0	0	0	0	0	0	0
0	0	0	0	0	0	0	0	0	0	0	0	0
0	0	0	0	0	0	0	0	0	0	0	0	0
0	0	0	0	0	0	0	0	0	0	0	0	0
0	0	0	0	0	0	0	0	0	0	0	0	0
0	0	0	0	0	0	0	0
"Viruses;Uroviricota;Caudoviricetes;Caudoviricetes__na;Caudoviricetes__na;Caudoviricetes__na;Aeromonas_phage_P5"	0	0	0	1	0	0	0	0	0	0	0	0
0	0	0	0	0	0	0	0	0	0	0	0	0
0	0	0	0	0	0	0	0	0	0	0	0	0
0	0	0	0	0	0	0	0	0	0	0	0	0

0 0 0 0 0 0 0 0 0 0 0 0 0
0 0 0 0 0 0 0 0
"Viruses;Uroviricota;Caudoviricetes;Caudoviricetes__na;Caudoviricetes__na;Caudoviricetes__na;Aeromonas_p
hage_phiA005" 0 0 0 0 1 0 0 0 0 0 0 0
0 0 0 0 0 0 0 0 0 0 0 0 0
0 0 0 0 0 0 0 0 0 0 0 0 0
0 0 0 0 0 0 0 0 0 0 0 0 0
0 0 0 0 0 0 0 0 0 0 0 0 0
0 0 0 0 0 0 0 0
"Viruses;Uroviricota;Caudoviricetes;Caudoviricetes__na;Caudoviricetes__na;Caudoviricetes__na;Aeromonas_p
hage_vB_AveS_KLEA5"0 0 1 1 0 0 0 0 0 0 0
0 0 0 0 0 0 0 0 0 0 0 0 0
0 0 0 0 0 0 0 0 0 0 0 0 0
0 0 0 1 0 0 0 0 0 0 0 0 0
0 0 0 0 0 0 0 0 0 0 0 0 0
0 0 0 0 0 0 0 5 0
"Viruses;Uroviricota;Caudoviricetes;Caudoviricetes__na;Caudoviricetes__na;Caudoviricetes__na;Alcaligenes_p
hage_vB_Af_QDWS535" 0 1 8 18 46 41 0 0 0 0
0 0 0 0 0 0 1 0 0 0 0 0 0
2 0 0 0 0 0 1 0 0 0 0 0 0
0 0 0 0 0 4 0 0 0 0 0 12 0
0 0 0 1 0 0 0 0 0 0 0 0 0
0 0 0 0 0 0 0 0 0 0
"Viruses;Uroviricota;Caudoviricetes;Caudoviricetes__na;Caudoviricetes__na;Caudoviricetes__na;Alistipes_phag
e_AS73P1" 0 0 0 0 0 0 0 0 0 0 0 0
0 0 0 0 0 0 0 0 0 0 0 0 0
0 0 0 0 0 0 0 0 0 0 0 0 0
0 0 0 0 2 0 0 0 0 0 0 0 0
0 0 0 0 0 0 0 0 0 0 0 0 0
0 0 0 0 0 0 0 0
"Viruses;Uroviricota;Caudoviricetes;Caudoviricetes__na;Caudoviricetes__na;Caudoviricetes__na;Alteromonas_
phage_P24" 0 0 0 0 0 2 0 0 0 0 0 0
0 0 0 0 0 0 0 0 0 0 0 0 0
0 0 0 0 0 0 0 0 0 0 0 0 0
0 0 0 0 0 0 0 0 0 0 0 0 0
0 0 0 0 0 0 0 0 0 0 0 0 0
0 0 0 0 0 0 0 0
"Viruses;Uroviricota;Caudoviricetes;Caudoviricetes__na;Caudoviricetes__na;Caudoviricetes__na;Alteromonas_
phage_XX1924" 0 0 0 0 1 1 0 0 0 0 0 0
0 0 0 0 0 0 0 0 0 0 0 0 0
0 0 0 0 0 0 0 0 0 0 0 0 0
0 0 0 0 0 0 0 0 0 0 0 0 0
0 0 0 0 0 0 0 0 0 0 0 0 0
0 0 0 0 0 0 0 0
"Viruses;Uroviricota;Caudoviricetes;Caudoviricetes__na;Caudoviricetes__na;Caudoviricetes__na;Arthrobacter_
phage_Atuin" 0 0 0 0 1 0 0 0 0 0 0 0
0 0 0 0 0 0 0 0 0 0 0 0 0
0 0 0 0 0 0 0 0 0 0 0 0 0
0 0 0 0 0 0 0 0 0 0 0 0 0

0 0 0 0 0 0 0 0 0 0 0 0 0
0 0 0 0 0 0 0 0
"Viruses;Uroviricota;Caudoviricetes;Caudoviricetes__na;Caudoviricetes__na;Caudoviricetes__na;Bacillus_phage
_1_ICo-2020" 0 0 0 0 0 0 0 0 0 0 0 0
0 0 0 0 0 0 0 0 0 0 0 0 0
0 0 0 0 0 0 0 0 0 0 0 0 0
0 0 0 0 0 0 0 0 0 0 0 0 0
0 0 0 0 0 0 0 0 0 0 1 0 0
0 0 0 0 0 0 0 0
"Viruses;Uroviricota;Caudoviricetes;Caudoviricetes__na;Caudoviricetes__na;Caudoviricetes__na;Bacillus_phage
_Kirov" 0 0 0 0 0 0 0 0 0 0 0 0 0
0 0 0 0 2 0 0 0 0 0 0 0 0
0 0 0 0 0 0 0 0 0 0 0 0 0
0 0 0 0 0 0 0 0 0 0 0 0 0
0 0 0 0 0 0 0 0 0 0 0 0 0
0 0 0 0 0 0 0
"Viruses;Uroviricota;Caudoviricetes;Caudoviricetes__na;Caudoviricetes__na;Caudoviricetes__na;Bacillus_phage
_vB_BanS_Chewbecca" 0 0 0 0 0 0 0 0 0 0 0
0 0 0 0 0 0 0 0 0 0 0 0 0
0 0 0 0 0 0 0 0 0 0 0 2 0
0 0 0 0 0 0 0 0 0 0 0 0 0
0 0 0 0 0 0 0 0 0 0 0 0 0
0 0 0 0 0 0 0 0 0
"Viruses;Uroviricota;Caudoviricetes;Caudoviricetes__na;Caudoviricetes__na;Caudoviricetes__na;Bacillus_phage
_vB_BanS_Sophrita" 0 0 2 0 0 0 0 0 0 0 0
0 0 0 0 0 0 0 0 0 0 0 0 0
0 0 0 0 0 0 0 0 0 0 0 0 0
0 0 0 0 0 0 0 0 0 0 0 0 0
0 0 0 0 0 0 0 0 0 0 0 0 0
0 0 0 0 0 0 0 0 0
"Viruses;Uroviricota;Caudoviricetes;Caudoviricetes__na;Caudoviricetes__na;Caudoviricetes__na;Bacillus_phage
_vB_BanS-Thrax2" 0 0 0 0 0 0 0 0 0 0 0
0 0 0 0 0 0 0 0 0 0 0 0 0
0 0 0 0 0 0 0 0 0 0 0 0 0
0 0 0 0 0 0 0 0 0 0 0 0 0
0 0 0 0 0 1 0 0 0 0 0 0 0
0 0 0 0 0 0 0 0 0
"Viruses;Uroviricota;Caudoviricetes;Caudoviricetes__na;Caudoviricetes__na;Caudoviricetes__na;Bacillus_phage
_vB_BceS_LY1" 0 0 0 0 0 0 0 0 0 0 0 0
1 0 0 0 0 0 0 0 0 0 0 0 0
0 0 0 0 0 0 0 0 0 0 0 0 0
0 0 0 0 0 0 0 0 0 0 0 0 0
0 0 0 0 0 0 0 0 0 0 0 0 0
0 0 0 0 0 0 0 0
"Viruses;Uroviricota;Caudoviricetes;Caudoviricetes__na;Caudoviricetes__na;Caudoviricetes__na;Bacillus_phage
_vB_BcoS-136" 0 0 3 0 0 3 0 0 0 0 0 0
0 0 0 1 0 0 0 0 0 0 0 0 0
0 0 0 0 0 0 0 0 0 0 0 0 0
0 0 0 0 0 0 0 0 0 0 0 0 0

0 0 0 0 0 0 0 0 0 0 0 0 0
0 0 0 0 0 0 0 0
"Viruses;Uroviricota;Caudoviricetes;Caudoviricetes__na;Caudoviricetes__na;Caudoviricetes__na;Bacillus_phage
_vB_BhaS-171" 0 0 0 0 0 0 0 0 0 0 0 0
0 0 0 0 1 0 0 0 0 0 0 0 0
0 0 0 0 0 0 0 0 0 0 0 0 0
0 0 0 0 0 0 0 0 0 0 0 0 0
0 0 0 0 0 0 0 0 0 0 0 0 0
0 0 0 0 0 0 0 0
"Viruses;Uroviricota;Caudoviricetes;Caudoviricetes__na;Caudoviricetes__na;Caudoviricetes__na;Bacillus_phage
_vB_BspM_AgentSmith" 0 0 0 0 0 1 0 0 0 0
0 0 0 0 0 0 0 0 0 0 0 0 0
0 0 0 0 0 0 0 0 0 0 0 0 0
0 0 0 0 0 0 0 0 0 0 0 0 0
0 0 0 0 0 0 0 0 0 0 0 0 0
0 0 0 0 0 0 0 0 0 0
"Viruses;Uroviricota;Caudoviricetes;Caudoviricetes__na;Caudoviricetes__na;Caudoviricetes__na;Bacteroides_p
hage_B124-14" 0 0 0 0 0 0 0 0 0 0 0 0
0 0 0 0 0 0 0 0 0 0 0 0 0
0 0 0 0 0 0 0 0 0 0 0 1 0
0 0 0 0 0 0 0 0 0 0 0 0 0
0 0 0 0 0 0 0 0 0 0 0 0 0
0 0 0 0 0 0 0 0
"Viruses;Uroviricota;Caudoviricetes;Caudoviricetes__na;Caudoviricetes__na;Caudoviricetes__na;Bacteroides_p
hage_BF10P2" 0 0 0 0 2 0 0 0 0 0 0 0
0 0 0 0 0 0 0 0 0 0 0 0 0
0 0 0 0 0 0 0 0 0 0 0 0 0
0 0 0 0 0 0 0 0 0 0 0 0 0
0 0 0 0 0 0 0 0 0 0 0 0 0
0 0 0 0 0 0 0 0
"Viruses;Uroviricota;Caudoviricetes;Caudoviricetes__na;Caudoviricetes__na;Caudoviricetes__na;Bacteroides_p
hage_BF766P1" 0 0 0 0 6 5 0 0 0 0 0 0
0 0 0 0 0 0 0 0 0 0 0 0 0
0 0 0 0 0 0 0 0 0 0 0 0 0
0 0 0 0 0 0 0 0 0 0 0 0 0
0 0 0 0 0 0 0 0 0 0 0 0 0
0 0 0 0 0 0 0 0
"Viruses;Uroviricota;Caudoviricetes;Caudoviricetes__na;Caudoviricetes__na;Caudoviricetes__na;Bacteroides_p
hage_BT638P6" 0 0 0 0 0 0 0 0 0 0 0 0
0 0 0 0 0 0 1 0 0 0 0 0 0
0 0 0 0 0 0 0 0 0 0 0 0 0
0 0 0 0 0 0 0 0 0 0 0 0 0
0 0 0 0 0 0 0 0 0 0 0 0 0
0 0 0 0 0 0 0 0
"Viruses;Uroviricota;Caudoviricetes;Caudoviricetes__na;Caudoviricetes__na;Caudoviricetes__na;Bacteroides_p
hage_DAC23" 0 0 0 0 0 0 0 0 0 0 0 0
0 0 0 0 0 0 0 0 2 2 0 0 0
0 0 0 0 0 0 0 0 0 0 0 0 0
0 0 1 0 0 0 6 0 0 0 0 0 0

0 0 0 0 0 0 0 0 0 0 0 0 0
0 0 0 0 0 0 0 0
"Viruses;Uroviricota;Caudoviricetes;Caudoviricetes__na;Caudoviricetes__na;Caudoviricetes__na;Bacteroides_phage_LoVEphage" 0 0 4 0 0 0 0 0 0 0 0
0 0 0 0 0 0 0 54 54 86 113 4 14
19 34 290 60 319 3 0 0 0 0 0 0 0
0 0 0 1 0 14 12 58 5 0 0 37 24
1 4 0 0 7 9 0 0 0 0 7 0 0
0 0 0 0 0 0 0 0 0
"Viruses;Uroviricota;Caudoviricetes;Caudoviricetes__na;Caudoviricetes__na;Caudoviricetes__na;Bacteroides_phage_SJC10" 0 0 0 0 0 0 0 0 0 0 0 0
0 0 0 0 0 9 0 0 0 0 0 0 0
0 0 0 0 0 0 0 0 0 0 0 0 0
0 0 0 0 0 0 0 0 0 0 0 0 0
0 0 0 0 0 0 0 0 0 0 0 0 0
0 0 0 0 0 0 0 0
"Viruses;Uroviricota;Caudoviricetes;Caudoviricetes__na;Caudoviricetes__na;Caudoviricetes__na;Bifidobacterium_phage_Bbif-1" 0 0 0 0 0 0 0 0 0 0 0
0 0 0 0 0 0 0 0 0 0 0 0 0
0 0 0 0 0 0 0 0 0 0 0 0 0
0 0 0 0 0 0 0 0 0 0 0 0 0
0 0 0 0 0 0 0 0 0 0 0 0 0
0 0 0 2 0 0 0 0 0
"Viruses;Uroviricota;Caudoviricetes;Caudoviricetes__na;Caudoviricetes__na;Caudoviricetes__na;Brevundimonas_phage_vB_BgoS-Fanboy" 0 0 0 0 0 0 0 0 0 1
0 0 0 0 0 0 0 0 0 0 0 0 0
0 0 0 0 0 0 0 0 0 0 0 0 0
0 0 0 0 0 0 0 0 0 0 0 0 0
0 0 0 0 0 0 0 0 0 0 0 0 0
0 0 0 0 0 0 0 0 0 0
"Viruses;Uroviricota;Caudoviricetes;Caudoviricetes__na;Caudoviricetes__na;Caudoviricetes__na;Brevundimonas_phage_vB_BpoS-Gurke" 0 0 0 0 0 1 0 0 0 0
0 0 0 0 0 0 0 0 0 0 0 0 0
0 0 0 0 0 0 0 0 0 0 0 0 0
0 0 0 0 0 0 0 0 0 0 0 0 0
0 0 0 0 0 0 0 0 0 0 0 0 0
0 0 0 0 0 0 0 0 0 0
"Viruses;Uroviricota;Caudoviricetes;Caudoviricetes__na;Caudoviricetes__na;Caudoviricetes__na;Brevundimonas_phage_vB_BpoS-Leszy" 0 0 0 0 0 0 0 0 0 1
0 0 0 0 0 0 0 0 0 0 0 0 0
0 0 0 0 0 0 0 0 0 0 0 0 0
0 0 0 0 0 0 0 0 0 0 0 0 0
0 0 0 0 0 0 0 0 0 0 0 0 0
0 0 0 0 0 0 0 0 0 0
"Viruses;Uroviricota;Caudoviricetes;Caudoviricetes__na;Caudoviricetes__na;Caudoviricetes__na;Brevundimonas_phage_vB_BpoS-Polewnik" 0 0 1 1 0 0 0 0 0 0
0 0 0 0 0 0 0 0 0 0 0 0 0
0 0 0 0 0 0 0 0 0 0 0 0 0
0 0 0 0 0 0 0 0 0 0 0 0 0

0 0 0 0 0 0 0 0 0 0 0 0 0
0 0 0 0 0 0 0 0 0 0
"Viruses;Uroviricota;Caudoviricetes;Caudoviricetes__na;Caudoviricetes__na;Caudoviricetes__na;Brevundimona
s_phage_vB_BpoS-Poludnitsa" 0 0 0 0 0 0 0 0 0 0
0 0 0 0 0 0 0 0 0 0 0 0 0
0 0 0 0 0 0 0 0 0 0 0 0 0
0 0 0 0 0 1 0 0 0 0 0 0 0
0 0 0 0 0 0 0 0 0 0 0 0 0
0 0 0 0 0 0 0 0 0 0
"Viruses;Uroviricota;Caudoviricetes;Caudoviricetes__na;Caudoviricetes__na;Caudoviricetes__na;Burkholderia_
phage_BcepB1A" 0 0 0 0 1 0 0 0 0 0 0
0 0 0 0 0 0 0 0 0 0 0 0 0
0 0 0 0 0 0 0 0 0 0 0 0 0
0 0 0 0 0 0 0 0 0 0 0 0 0
0 0 0 0 0 0 0 0 0 0 0 0 0
0 0 0 0 0 0 0 0 0
"Viruses;Uroviricota;Caudoviricetes;Caudoviricetes__na;Caudoviricetes__na;Caudoviricetes__na;Burkholderia_
phage_FLC9" 0 0 0 0 0 0 0 0 0 0 0 0
0 0 0 0 0 0 0 0 0 0 0 0 0
0 0 0 0 0 0 0 0 0 0 0 0 0
0 0 0 0 0 0 0 0 0 1 0 0 0
0 0 0 0 0 0 0 0 0 0 0 0 0
0 0 0 0 0 0 0 0
"Viruses;Uroviricota;Caudoviricetes;Caudoviricetes__na;Caudoviricetes__na;Caudoviricetes__na;Burkholderiale
s_phage_68_11" 0 0 0 2 0 0 0 0 0 0 0 0
0 0 0 0 0 0 0 0 0 0 0 0 0
0 0 0 0 0 0 0 0 0 0 0 0 0
0 0 0 0 0 0 0 0 0 0 0 0 0
0 0 0 0 0 0 0 0 0 0 0 0 0
0 0 0 0 0 0 0 0
"Viruses;Uroviricota;Caudoviricetes;Caudoviricetes__na;Caudoviricetes__na;Caudoviricetes__na;Caudovirales_
sp._ct0jG3" 0 0 0 0 0 0 0 0 0 0 0 0
3 6 0 1 0 0 0 0 0 0 0 0 0
1 0 0 0 0 0 1 0 0 1 0 0 0
0 0 0 0 11 8 7 2 0 0 0 0 0
0 0 0 0 1 0 0 0 0 0 0 0 0
0 0 0 0 0 0 0 0
"Viruses;Uroviricota;Caudoviricetes;Caudoviricetes__na;Caudoviricetes__na;Caudoviricetes__na;Caudovirales_
sp._ct0YK8" 0 0 0 0 0 0 0 0 0 0 0 0
0 0 0 0 0 0 23 11 0 1 2 0 3
7 0 0 0 0 2 2 0 0 55 0 0 0
0 0 6 0 9 8 22 0 0 0 18 18 1
0 0 644 1 8 0 0 1 0 1 1 0 0
0 0 3 4 1 6 0 0
"Viruses;Uroviricota;Caudoviricetes;Caudoviricetes__na;Caudoviricetes__na;Caudoviricetes__na;Caudovirales_
sp._ct1Jx6" 0 0 0 0 0 11 0 0 0 0 0 0
0 1 0 0 0 0 0 0 0 0 0 0 0
0 0 0 0 0 0 0 0 0 0 0 0 0
0 0 0 0 0 0 0 0 0 0 0 0 0

0 0 0 0 0 0 0 0 0 0 0 0 0
0 0 0 0 0 0 0 0
"Viruses;Uroviricota;Caudoviricetes;Caudoviricetes__na;Caudoviricetes__na;Caudoviricetes__na;Caudovirales_
sp._ct2A51" 0 0 0 0 0 0 0 0 0 0 0 0
0 0 0 0 0 0 0 0 0 0 0 0 0
0 0 0 0 0 0 0 0 0 0 0 0 0
0 0 0 0 0 0 0 0 0 0 0 0 0
0 0 0 0 0 0 0 0 0 3 10 0 0
0 0 0 0 0 0 0 0
"Viruses;Uroviricota;Caudoviricetes;Caudoviricetes__na;Caudoviricetes__na;Caudoviricetes__na;Caudovirales_
sp._ct7Ux15" 0 0 0 0 0 0 0 0 0 0 0 0
0 0 0 0 0 0 0 0 0 0 0 0 0
2 0 0 0 0 1 0 0 0 1 0 0 0
0 0 0 0 0 0 0 0 0 0 0 0 0
0 0 0 1 0 0 0 0 0 0 0 0 0
0 0 0 0 0 0 0 0
"Viruses;Uroviricota;Caudoviricetes;Caudoviricetes__na;Caudoviricetes__na;Caudoviricetes__na;Caudovirales_
sp._ctaix4" 0 0 0 0 0 0 0 0 0 0 0 0
0 2 0 0 0 0 0 2 0 0 0 0 0
0 0 0 0 0 0 0 0 0 0 0 0 0
0 0 0 0 0 0 0 0 0 0 0 0 0
0 0 0 0 0 0 0 0 0 0 0 0 0
0 0 0 0 0 0 0 0
"Viruses;Uroviricota;Caudoviricetes;Caudoviricetes__na;Caudoviricetes__na;Caudoviricetes__na;Caudovirales_
sp._ctcLF7" 0 0 0 0 0 0 0 0 0 0 0 0
0 0 0 0 0 0 0 0 0 0 0 0 0
0 0 0 0 0 0 0 0 0 0 0 0 0
0 0 0 0 0 0 0 0 0 0 0 0 0
0 0 0 0 5 0 0 0 0 0 0 0 0
0 0 3 15 0 0 0 0
"Viruses;Uroviricota;Caudoviricetes;Caudoviricetes__na;Caudoviricetes__na;Caudoviricetes__na;Caudovirales_
sp._ctCpR1" 0 0 0 0 0 0 0 0 0 0 0 0
0 0 0 0 0 0 0 0 0 0 0 0 0
0 0 0 0 0 2 0 0 0 28 0 4 3
0 1 0 0 0 0 0 0 0 0 0 0 0
0 0 0 3 1 0 0 0 0 0 0 0 0
0 0 0 0 0 0 0 0
"Viruses;Uroviricota;Caudoviricetes;Caudoviricetes__na;Caudoviricetes__na;Caudoviricetes__na;Caudovirales_
sp._ctCVG11" 0 0 0 0 0 0 0 0 0 0 0 0
1 1 0 0 0 0 0 0 0 0 0 0 0
0 0 0 0 0 0 0 0 0 0 0 1 0
0 0 0 0 1 0 0 0 0 0 0 1 0
0 0 0 4 3 0 0 0 0 0 0 0 0
0 0 3 0 0 0 0 0
"Viruses;Uroviricota;Caudoviricetes;Caudoviricetes__na;Caudoviricetes__na;Caudoviricetes__na;Caudovirales_
sp._ctFWA4" 0 0 0 0 0 0 0 0 0 0 0 0
0 0 0 0 0 0 0 0 0 0 0 0 0
0 0 0 0 0 2 0 0 0 0 0 0 1
0 0 0 0 0 0 0 0 0 0 0 0 0

0 1 0 2 11 0 0 0 0 0 0 0 0
0 0 0 0 0 0 0 0
"Viruses;Uroviricota;Caudoviricetes;Caudoviricetes__na;Caudoviricetes__na;Caudoviricetes__na;Caudovirales_
sp._ctIsq18" 0 0 0 0 0 0 0 0 0 0 0 0
0 0 0 0 0 0 4 0 1 1 1 0 0
0 0 0 0 0 0 0 0 0 0 0 0 0
0 0 0 0 0 0 0 0 0 0 0 0 0
0 0 3 0 0 0 0 0 0 1 0 0 0
0 0 8 13 0 0 0 0
"Viruses;Uroviricota;Caudoviricetes;Caudoviricetes__na;Caudoviricetes__na;Caudoviricetes__na;Caudovirales_
sp._ctIZM3" 0 0 0 0 0 0 0 0 0 0 0 0
0 1 0 0 0 0 0 0 0 0 0 0 0
0 0 0 0 0 0 0 0 0 0 0 0 0
0 0 0 0 0 0 0 0 0 0 0 0 0
0 0 0 1 1 0 0 0 0 0 0 0 0
0 0 0 0 0 0 0 0
"Viruses;Uroviricota;Caudoviricetes;Caudoviricetes__na;Caudoviricetes__na;Caudoviricetes__na;Caudovirales_
sp._ctkvU4" 0 0 0 0 0 0 0 0 0 0 0 0
0 1 0 0 0 0 0 0 0 0 0 0 0
0 0 0 0 0 0 0 0 0 0 0 0 0
0 0 0 0 0 0 0 0 0 0 0 0 0
0 0 0 0 0 0 0 0 0 0 0 0 0
0 0 0 0 0 0 0 0
"Viruses;Uroviricota;Caudoviricetes;Caudoviricetes__na;Caudoviricetes__na;Caudoviricetes__na;Caudovirales_
sp._ctlwr10" 0 0 0 0 0 0 0 0 0 0 0 0
0 0 0 1 0 0 0 0 0 0 0 0 0
0 0 0 0 0 2 3 0 0 0 0 0 1
0 0 0 5 0 0 0 0 0 0 0 0 0
0 0 0 12 19 0 0 0 0 1 0 0 0
0 0 3 4 0 0 0 0
"Viruses;Uroviricota;Caudoviricetes;Caudoviricetes__na;Caudoviricetes__na;Caudoviricetes__na;Caudovirales_
sp._ctNZz8" 0 0 0 0 0 0 0 0 0 0 0 0
0 0 0 0 0 0 0 0 0 0 0 0 0
0 0 0 0 0 0 1 0 0 0 0 0 0
0 1 0 13 0 0 0 0 0 0 0 0 0
0 0 0 0 0 0 0 0 0 0 0 0 0
0 0 1 0 0 0 0 0
"Viruses;Uroviricota;Caudoviricetes;Caudoviricetes__na;Caudoviricetes__na;Caudoviricetes__na;Caudovirales_
sp._ctOwN3" 2 0 0 2 3 0 0 0 0 0 0 0
0 0 0 0 0 0 0 0 0 0 0 0 0
0 0 0 0 0 0 0 0 0 0 0 0 0
0 0 0 0 0 0 0 0 0 0 0 0 0
0 0 0 0 0 0 0 0 0 0 0 0 0
0 0 0 0 0 0 0 0
"Viruses;Uroviricota;Caudoviricetes;Caudoviricetes__na;Caudoviricetes__na;Caudoviricetes__na;Caudovirales_
sp._ctqPn17" 0 0 0 0 0 0 0 0 0 0 0 0
0 1 0 0 0 0 0 0 0 0 0 0 3
6 0 0 0 0 0 0 0 0 0 0 2 5
0 0 0 0 0 0 0 0 0 0 0 0 0

0 0 0 0 0 0 0 0 0 0 0 0 0
0 0 0 0 0 0 0 0
"Viruses;Uroviricota;Caudoviricetes;Caudoviricetes__na;Caudoviricetes__na;Caudoviricetes__na;Caudovirales_
sp._ctt3K6" 0 0 0 0 0 0 0 0 0 0 0 0
0 0 0 0 0 0 0 0 0 0 0 0 0
0 0 0 0 0 0 0 0 0 0 0 1 1
0 2 0 0 0 0 0 0 0 0 0 0 0
0 0 0 0 0 0 0 0 0 0 1 0 0
0 0 0 0 0 0 0 0
"Viruses;Uroviricota;Caudoviricetes;Caudoviricetes__na;Caudoviricetes__na;Caudoviricetes__na;Caudovirales_
sp._ctTVN2" 0 0 0 0 0 0 0 0 0 0 0 0
0 0 0 0 0 0 0 0 0 0 0 0 17
7 0 0 0 0 0 3 0 0 0 0 1 2
0 2 0 0 0 0 0 0 0 0 2 7 0
0 0 0 1 0 0 0 0 0 1 0 0 0
0 0 0 5 0 1 0 0
"Viruses;Uroviricota;Caudoviricetes;Caudoviricetes__na;Caudoviricetes__na;Caudoviricetes__na;Caudovirales_
sp._ctu3532" 0 0 0 0 0 0 0 0 0 0 0 0
0 0 0 0 0 0 0 2 0 0 0 0 0
2 0 0 0 0 0 2 0 0 0 0 1 0
0 0 0 0 0 0 0 0 0 0 0 0 0
0 0 0 4 2 0 0 0 0 0 0 0 0
0 0 0 0 0 0 0 0
"Viruses;Uroviricota;Caudoviricetes;Caudoviricetes__na;Caudoviricetes__na;Caudoviricetes__na;Caudovirales_
sp._ctU7I6" 0 0 0 0 0 0 0 0 0 0 0 0
0 0 0 0 0 0 0 0 0 0 0 0 3
2 0 0 0 0 0 0 0 0 0 0 1 2
0 0 0 0 0 0 0 0 0 0 0 0 0
0 0 0 0 0 0 0 0 0 0 0 0 0
0 0 0 0 0 0 0 0
"Viruses;Uroviricota;Caudoviricetes;Caudoviricetes__na;Caudoviricetes__na;Caudoviricetes__na;Caudovirales_
sp._ctUJJ3" 0 0 0 0 0 0 0 0 0 0 0 0
0 0 0 0 0 0 0 0 0 0 0 0 0
0 0 0 0 0 0 0 0 0 0 0 2 0
0 0 0 0 2 9 6 0 0 0 1 1 0
0 0 0 0 1 0 0 0 0 0 0 0 0
0 0 0 0 0 0 0 0
"Viruses;Uroviricota;Caudoviricetes;Caudoviricetes__na;Caudoviricetes__na;Caudoviricetes__na;Caudovirales_
sp._ctUL28" 0 0 0 0 0 0 0 0 0 0 0 0
0 0 0 0 0 0 0 0 0 0 0 0 0
0 0 0 0 0 0 2 0 0 3 0 0 0
0 0 0 0 0 0 0 0 0 0 0 0 0
0 0 0 0 0 0 0 0 0 0 0 0 0
0 0 0 0 0 0 0 0
"Viruses;Uroviricota;Caudoviricetes;Caudoviricetes__na;Caudoviricetes__na;Caudoviricetes__na;Caudovirales_
sp._ctVfb8" 0 0 0 0 0 0 0 0 0 0 0 0
0 0 0 0 0 0 0 0 0 0 0 0 0
0 0 0 0 0 0 0 0 0 0 0 2 1
2 0 0 0 0 0 0 0 0 0 0 0 0

0 1 1 0 0 0 0 0 0 0 0 0 0
0 0 0 0 0 0 0 0
"Viruses;Uroviricota;Caudoviricetes;Caudoviricetes__na;Caudoviricetes__na;Caudoviricetes__na;Caudovirales_
sp._ctyaR3" 0 0 0 0 0 0 0 0 0 0 0 0
0 0 0 0 0 0 0 0 0 0 0 0 0
1 0 0 0 0 0 0 0 0 0 0 2 2
0 0 0 0 0 0 0 0 0 0 2 2 0
0 0 0 1 4 0 0 0 0 5 2 0 0
0 0 7 6 0 2 0 0
"Viruses;Uroviricota;Caudoviricetes;Caudoviricetes__na;Caudoviricetes__na;Caudoviricetes__na;Caudoviricetes
_sp." 104 166 685 452 4813 1458 8 61 87 79 667 1 21774
20310 18110 18633 19171 68656 13819 15834 4459 3525 3340 1679 11813 12349
6593 1455 5069 563 12374 13033 1467 944 98319 1131 33781 30394 3085
2857 12102 67989 8790 10123 68363 13722 5575 1480 31683 17744 6205 2052
7895 164530 19533 27132 1669 1089 2099 3935 24404 8319 1525 2714 4106
624 18072 24102 1358 4535 1614 0
"Viruses;Uroviricota;Caudoviricetes;Caudoviricetes__na;Caudoviricetes__na;Caudoviricetes__na;Chaetfec_virus
_UA24_144" 0 0 0 0 2 0 0 0 0 0 0 0
0 0 0 0 0 0 0 0 0 0 0 0 0
0 0 0 0 0 0 0 0 0 0 0 0 0
0 0 0 0 0 0 0 0 0 0 0 0 0
0 0 0 0 0 0 0 0 0 0 0 0 0
0 0 0 1 0 0 0 0
"Viruses;Uroviricota;Caudoviricetes;Caudoviricetes__na;Caudoviricetes__na;Caudoviricetes__na;Chaetfec_virus
_UA24_244" 0 0 0 0 0 0 0 0 0 0 0 0
0 0 0 0 0 0 0 0 0 0 0 0 0
0 0 0 0 0 0 0 0 0 0 0 0 0
0 0 0 0 0 0 0 0 0 0 0 0 0
0 0 0 1 0 0 0 0 0 0 0 0 0
0 0 0 0 0 0 0 0
"Viruses;Uroviricota;Caudoviricetes;Caudoviricetes__na;Caudoviricetes__na;Caudoviricetes__na;Citromicrobiu
m_phage_vB_CbaS-RXM" 0 0 1 0 0 0 0 0 0 0
0 0 0 0 0 0 0 0 0 0 0 0 0
0 0 0 0 0 0 0 0 0 0 0 0 0
0 0 0 0 0 0 0 0 0 0 0 0 0
0 0 0 0 0 0 0 0 0 0 0 0 0
0 0 0 0 0 0 0 0 0 0
"Viruses;Uroviricota;Caudoviricetes;Caudoviricetes__na;Caudoviricetes__na;Caudoviricetes__na;Clostridium_p
hage_phiCTP1" 0 0 0 0 0 0 0 0 0 0 0 0
0 0 0 0 0 0 0 0 0 0 0 0 0
0 0 0 0 0 0 0 0 0 0 0 0 0
0 0 0 0 0 0 0 0 0 0 0 0 0
0 0 0 0 1 0 0 0 0 0 0 0 0
0 0 0 0 0 0 0 0
"Viruses;Uroviricota;Caudoviricetes;Caudoviricetes__na;Caudoviricetes__na;Caudoviricetes__na;Corynebacteri
um_phage_Bran" 0 0 0 1 0 0 0 0 0 0 0
0 0 0 0 0 0 0 0 0 0 0 0 0
0 0 0 0 0 0 0 0 0 0 0 0 0
0 0 0 0 0 0 0 0 0 0 0 0 0

0 0 0 0 0 0 0 0 0 0 0 0 0
0 0 0 0 0 0 0 0 0
"Viruses;Uroviricota;Caudoviricetes;Caudoviricetes__na;Caudoviricetes__na;Caudoviricetes__na;Corynebacteri
um_phage_EmiRose" 0 0 0 0 0 0 0 0 0 0 0
0 0 0 0 0 0 0 0 0 0 0 0 0
0 0 0 0 0 0 0 0 0 0 0 0 0
0 0 0 0 0 0 0 3 0 0 0 0 0
0 0 0 0 0 0 0 0 0 0 0 0 0
0 0 0 0 0 0 0 0 0
"Viruses;Uroviricota;Caudoviricetes;Caudoviricetes__na;Caudoviricetes__na;Caudoviricetes__na;Corynebacteri
um_phage_IME1320_01" 0 0 0 0 0 0 0 0 0 0
0 0 0 0 0 0 0 0 0 0 0 0 0
0 0 0 0 0 0 0 0 0 0 0 0 1
0 0 0 0 0 0 0 0 0 0 0 0 0
0 0 0 0 0 0 0 0 0 0 0 0 0
0 0 0 0 0 0 0 0 0 0
"Viruses;Uroviricota;Caudoviricetes;Caudoviricetes__na;Caudoviricetes__na;Caudoviricetes__na;Croceibacter_p
hage_P2559S" 0 0 0 0 1 0 0 0 0 0 0 0
0 0 1 0 0 0 0 0 0 0 0 0 0
0 0 0 0 0 0 0 0 0 0 0 0 0
0 0 0 0 0 0 0 0 0 0 0 0 0
0 0 0 0 0 0 0 0 0 0 0 0 0
0 0 0 0 0 0 0 0
"Viruses;Uroviricota;Caudoviricetes;Caudoviricetes__na;Caudoviricetes__na;Caudoviricetes__na;Croceibacter_p
hage_P2559Y" 0 0 1 0 1 0 0 0 0 0 0 0
0 0 0 0 0 0 0 0 0 0 0 0 0
0 0 0 0 0 0 0 0 0 0 0 0 0
0 0 0 0 0 0 0 0 0 0 0 0 0
0 0 0 0 0 0 0 0 0 0 0 0 0
0 0 0 0 0 0 0 0
"Viruses;Uroviricota;Caudoviricetes;Caudoviricetes__na;Caudoviricetes__na;Caudoviricetes__na;Cronobacter_p
hage_vB_CsaP_Ss1" 0 0 0 0 0 0 0 0 0 0 0
0 0 0 0 0 0 0 0 0 0 0 0 0
0 0 0 0 0 0 0 0 0 0 0 0 0
0 0 0 0 0 0 0 0 0 0 1 0 0
0 0 0 0 0 0 0 0 0 0 0 0 0
0 0 0 0 0 0 0 0 0
"Viruses;Uroviricota;Caudoviricetes;Caudoviricetes__na;Caudoviricetes__na;Caudoviricetes__na;Curvibacter_p
hage_P26059A" 1 1 1 0 1 0 0 0 0 0 0 0
0 0 0 0 0 0 0 0 1 0 1 11 0
0 0 0 0 0 0 0 0 0 0 0 0 0
0 0 0 0 0 0 0 0 0 0 0 0 0
0 0 0 0 0 0 0 0 0 0 0 0 0
0 0 0 0 0 0 10 0
"Viruses;Uroviricota;Caudoviricetes;Caudoviricetes__na;Caudoviricetes__na;Caudoviricetes__na;Cyanophage_
KBS-S-2A" 0 0 0 0 2 0 0 0 0 0 0 0
0 0 0 0 0 0 0 0 0 0 0 0 0
0 0 0 0 0 0 0 0 0 0 0 0 0
0 0 0 0 0 0 0 0 0 0 0 0 0

0	0	0	0	0	0	0	0	0	0	0	0	0
0	0	0	0	0	0	0	0
"Viruses;Uroviricota;Caudoviricetes;Caudoviricetes__na;Caudoviricetes__na;Caudoviricetes__na;Cylindrospermopsis_phage_Cr-LKS3"	0	0	0	0	0	1	0	0	0	0
0	0	0	0	0	0	0	0	0	0	0	0	0
0	0	0	0	0	0	0	0	0	0	0	0	0
0	0	0	0	0	0	0	0	0	0	0	0	0
0	0	0	0	0	0	0	0	0	0	0	0	0
0	0	0	0	0	0	0	0	0	0
"Viruses;Uroviricota;Caudoviricetes;Caudoviricetes__na;Caudoviricetes__na;Caudoviricetes__na;Cylindrospermopsis_raciborskii_virus_RM-2018a"	0	0	0	0	0	0	0	0	0
0	0	0	0	0	0	0	0	0	0	0	0	0
0	0	0	0	0	0	0	0	0	0	0	0	0
0	0	0	0	0	0	0	0	0	0	0	0	2
0	0	0	0	0	0	0	0	0	0	0	0	0
0	0	0	0	0	0	0	0	0	0	0
"Viruses;Uroviricota;Caudoviricetes;Caudoviricetes__na;Caudoviricetes__na;Caudoviricetes__na;Desulfofustis_phage_LS06-2018-MD01"	0	0	0	0	0	1	0	0	0	0
0	0	0	0	0	0	0	0	0	0	0	0	0
0	0	0	0	0	0	0	0	0	0	0	0	0
0	0	0	0	0	0	0	0	0	0	0	1	0
0	0	0	0	0	0	0	0	0	0	0	0	0
0	0	0	0	0	0	0	0	0	0
"Viruses;Uroviricota;Caudoviricetes;Caudoviricetes__na;Caudoviricetes__na;Caudoviricetes__na;Desulfofustis_phage_LS06-2018-MD02"	0	0	0	0	0	43	0	0	0	0
0	0	0	0	0	0	0	0	0	0	0	0	0
0	0	0	0	0	0	0	0	0	0	0	0	0
0	0	0	0	0	0	0	0	0	0	0	0	0
0	0	0	0	0	0	0	0	0	0	0	0	0
0	0	0	0	0	0	0	0	0	0
"Viruses;Uroviricota;Caudoviricetes;Caudoviricetes__na;Caudoviricetes__na;Caudoviricetes__na;Dorea_phage_DO134P1"	0	0	0	0	0	0	0	0	0	0	0	0
0	0	0	0	0	0	0	0	0	0	0	0	0
1	0	0	0	0	0	0	0	0	0	0	0	0
0	0	0	0	0	0	0	0	0	0	0	0	0
0	0	0	0	0	0	0	0	0	0	0	0	0
0	0	0	0	0	0	0	0
"Viruses;Uroviricota;Caudoviricetes;Caudoviricetes__na;Caudoviricetes__na;Caudoviricetes__na;Dunaliella_viridis_virus_SI2"	1	0	0	0	0	0	0	0	0	0	0	0
0	0	0	0	0	0	0	0	0	0	0	0	0
0	0	0	0	0	0	0	0	0	0	0	0	0
0	0	0	0	0	0	0	0	0	0	0	0	0
0	0	0	0	0	0	0	0	0	0	0	0	0
0	0	0	0	0	0	0	0
"Viruses;Uroviricota;Caudoviricetes;Caudoviricetes__na;Caudoviricetes__na;Caudoviricetes__na;EBPR_podovirus_1"	0	0	0	3	17	1	0	0	0	0	0	0	0
0	0	0	1	0	0	0	0	0	0	0	0	0
1	0	0	0	0	0	0	0	0	0	0	0	0
0	0	0	0	0	0	0	0	0	0	0	0	0

0 0 0 0 0 0 0 0 0 0 0 0 0
0 0 0 0 0 0 0
"Viruses;Uroviricota;Caudoviricetes;Caudoviricetes__na;Caudoviricetes__na;Caudoviricetes__na;EBPR_podovir
us_2" 1 0 6 7 12 5 0 0 0 0 0 0 0
0 0 1 0 0 0 0 0 0 0 0 0 0
0 0 0 0 0 0 0 0 0 0 0 0 0
0 0 4 0 0 0 0 0 0 0 0 0 0
0 0 0 0 0 0 0 0 0 0 0 0 0
0 0 0 0 0 0 0
"Viruses;Uroviricota;Caudoviricetes;Caudoviricetes__na;Caudoviricetes__na;Caudoviricetes__na;EBPR_podovir
us_3" 1 0 1 2 1 11 0 0 0 0 0 0 0
0 0 0 0 0 0 0 0 0 0 0 0 0
0 0 0 0 0 0 0 0 1 0 0 0 0
0 0 0 0 0 0 0 0 0 0 0 0 0
0 0 0 0 0 0 0 0 0 0 0 0 0
0 0 0 0 0 0 0
"Viruses;Uroviricota;Caudoviricetes;Caudoviricetes__na;Caudoviricetes__na;Caudoviricetes__na;EBPR_siphovi
rus_1" 0 0 0 0 14 0 0 0 0 0 0 0 0
0 0 0 0 0 0 0 0 0 0 0 0 0
0 0 0 0 0 0 0 0 0 0 0 0 0
0 0 0 0 0 0 0 0 0 0 0 0 0
0 0 0 0 0 0 0 0 0 0 0 0 0
0 0 0 0 0 0 0
"Viruses;Uroviricota;Caudoviricetes;Caudoviricetes__na;Caudoviricetes__na;Caudoviricetes__na;EBPR_siphovi
rus_2" 0 0 1 1 0 13 0 0 0 0 0 0 0
0 0 0 0 0 0 0 0 0 0 0 0 0
0 0 0 0 0 0 0 0 0 0 0 0 0
0 0 0 0 0 0 0 0 0 0 0 0 0
0 0 0 0 0 0 0 0 0 0 0 0 0
0 0 0 0 0 0 0
"Viruses;Uroviricota;Caudoviricetes;Caudoviricetes__na;Caudoviricetes__na;Caudoviricetes__na;Edwardsiella_
phage_vB_EpM_ZHS" 0 0 0 0 0 0 0 0 0 0 0
0 0 0 0 0 0 0 0 0 0 0 0 0
0 0 0 0 0 0 0 0 0 0 0 0 0
0 0 0 0 0 0 0 0 0 10 0 0 0
0 0 0 0 0 0 0 0 0 0 0 0 0
0 0 0 0 0 0 0 0 0
"Viruses;Uroviricota;Caudoviricetes;Caudoviricetes__na;Caudoviricetes__na;Caudoviricetes__na;Edwardsiella_
phage_vB_EtaM_ET-ABTNL-9" 0 0 1 2 0 0 0 0 0 1
0 0 0 0 0 0 0 0 0 0 0 0 0
0 0 0 0 0 0 0 0 0 0 0 0 0
0 0 0 0 0 0 0 0 0 0 0 0 0
0 0 0 0 0 0 0 0 0 0 0 0 0
0 0 0 0 0 0 0 0 0 0
"Viruses;Uroviricota;Caudoviricetes;Caudoviricetes__na;Caudoviricetes__na;Caudoviricetes__na;Elizabethkingi
a_phage_TCUEAP2" 0 0 0 1 0 0 0 0 0 0 0
0 0 0 0 0 0 0 0 0 0 0 0 0
0 0 0 0 0 0 0 0 0 0 0 0 0
0 0 0 0 0 0 0 0 0 0 0 0 0

0 0 0 0 0 0 0 0 0 0 0 0 0
0 0 0 0 0 0 0 0 0
"Viruses;Uroviricota;Caudoviricetes;Caudoviricetes__na;Caudoviricetes__na;Caudoviricetes__na;Enterobacteria
_phage_mEp021" 0 0 0 0 0 3 0 0 0 0 0
0 0 0 0 0 0 0 0 0 0 0 0 0
0 0 0 0 0 0 0 0 0 0 0 0 0
0 0 0 0 0 0 0 0 0 0 0 0 0
0 0 0 0 0 0 0 0 0 0 0 0 0
0 0 0 0 0 0 0 0 0
"Viruses;Uroviricota;Caudoviricetes;Caudoviricetes__na;Caudoviricetes__na;Caudoviricetes__na;Enterocloster_
phage_PMBT10" 0 0 0 0 0 2 0 0 0 0 0
0 0 0 0 0 0 0 0 0 0 0 0 1
0 0 0 0 0 0 0 0 0 0 0 0 0
0 0 0 0 0 0 0 0 0 0 0 0 0
0 0 0 0 0 0 0 0 0 0 0 0 0
0 0 0 0 0 0 0 0 0
"Viruses;Uroviricota;Caudoviricetes;Caudoviricetes__na;Caudoviricetes__na;Caudoviricetes__na;Erythrobacter_
phage_vB_EliS-L02" 0 0 1 0 5 0 0 0 0 0 0
0 0 0 0 0 0 0 0 0 0 0 0 0
0 0 0 0 0 0 0 0 0 0 0 0 0
0 0 0 0 0 0 0 0 0 0 0 0 0
1 0 0 0 0 0 0 0 0 0 0 0 0
0 0 0 0 0 0 0 0 0
"Viruses;Uroviricota;Caudoviricetes;Caudoviricetes__na;Caudoviricetes__na;Caudoviricetes__na;Escherichia_p
hage_BUCT618"0 0 0 0 0 0 0 0 0 0 0 0
0 0 0 0 0 0 0 0 0 0 0 0 0
0 0 0 0 0 0 0 0 0 0 0 0 0
0 0 0 0 0 0 0 0 0 1 0 0 0
0 0 0 0 0 0 0 0 0 0 0 0 0
0 0 0 0 0 0 0 0
"Viruses;Uroviricota;Caudoviricetes;Caudoviricetes__na;Caudoviricetes__na;Caudoviricetes__na;Escherichia_p
hage_YDC107_2" 0 0 0 0 0 0 0 0 0 0 0
0 0 0 0 0 0 0 0 0 0 0 0 0
0 0 0 0 0 0 0 0 0 0 0 0 0
0 0 0 0 0 0 0 0 0 0 0 0 0
0 0 0 0 0 0 0 0 0 0 72 11 0
1 0 0 0 0 0 0 0 0
"Viruses;Uroviricota;Caudoviricetes;Caudoviricetes__na;Caudoviricetes__na;Caudoviricetes__na;Exiguobacteri
um_phage_vB_EauM-23" 0 0 0 0 0 0 0 0 0 0
0 0 0 0 0 0 0 0 0 0 0 0 0
0 0 0 0 0 0 0 0 0 0 0 0 0
0 0 0 0 0 0 0 0 0 0 0 1 0
0 0 0 0 0 0 0 0 0 0 0 0 0
0 0 0 0 0 0 0 0 0 0
"Viruses;Uroviricota;Caudoviricetes;Caudoviricetes__na;Caudoviricetes__na;Caudoviricetes__na;Flavobacteriu
m_phage_11b" 0 2 0 2 1 10 0 0 0 0 0 0
0 0 0 0 0 0 0 0 0 0 0 0 0
0 0 0 0 0 0 0 0 0 0 1 0 0
0 0 0 0 0 0 0 0 0 13 0 0 0

0 0 0 0 0 0 0 0 0 0 0 0 0
0 0 0 0 0 0 0 0
"Viruses;Uroviricota;Caudoviricetes;Caudoviricetes__na;Caudoviricetes__na;Caudoviricetes__na;Flumine_myo-like_phage_1" 0 0 0 1 0 0 0 0 0 0 0 0
0 0 0 0 0 0 0 0 0 0 0 0 0
0 0 0 0 0 0 0 0 0 0 0 0 0
0 0 0 0 0 0 0 0 0 0 0 0 0
0 0 0 0 0 0 0 0 0 0 0 0 0
0 0 0 0 0 0 0 0
"Viruses;Uroviricota;Caudoviricetes;Caudoviricetes__na;Caudoviricetes__na;Caudoviricetes__na;Flumine_podo-like_phage_1" 0 0 0 0 0 1 0 0 0 0 0 0
0 0 0 0 0 0 0 0 0 0 0 0 0
0 0 0 0 0 0 0 0 0 0 0 0 0
0 0 0 0 0 0 0 0 0 0 0 0 0
0 0 0 0 0 0 0 0 0 0 0 0 0
0 0 0 0 0 0 0 0
"Viruses;Uroviricota;Caudoviricetes;Caudoviricetes__na;Caudoviricetes__na;Caudoviricetes__na;Flyfo_podovirus_Tbat2_2" 0 0 1 0 0 0 0 0 0 0 0 0
0 0 0 0 0 0 0 0 0 0 0 0 0
0 0 0 0 0 0 0 0 0 0 0 0 0
0 0 0 0 0 0 0 0 0 1 0 0 0
0 0 0 0 0 0 0 0 0 0 0 0 0
0 0 0 0 0 0 0 0
"Viruses;Uroviricota;Caudoviricetes;Caudoviricetes__na;Caudoviricetes__na;Caudoviricetes__na;Freshwater_phage_uvFW-CGR-AMD-COM-C203" 0 0 0 1 0 0 0 0 0
0 0 0 0 0 0 0 0 0 0 0 0 0
0 0 0 0 0 0 0 0 0 0 0 0 0
0 0 0 0 0 0 0 0 0 0 0 0 0
0 0 0 0 0 0 0 0 0 0 0 0 0
0 0 0 0 0 0 0 0 0 0 0
"Viruses;Uroviricota;Caudoviricetes;Caudoviricetes__na;Caudoviricetes__na;Caudoviricetes__na;Freshwater_phage_uvFW-CGR-AMD-COM-C403" 0 0 0 0 0 0 0 0 0
0 0 0 0 0 0 0 0 0 0 0 0 0
0 0 0 0 0 1 0 0 0 0 0 0 0
0 0 0 0 0 0 0 0 0 0 0 0 0
0 0 0 0 0 0 0 0 0 0 0 0 0
0 0 0 0 0 0 0 0 0 0 0
"Viruses;Uroviricota;Caudoviricetes;Caudoviricetes__na;Caudoviricetes__na;Caudoviricetes__na;Freshwater_phage_uvFW-CGR-AMD-COM-C429" 0 0 0 0 1 0 0 0 0
0 0 0 0 0 0 0 0 0 0 0 0 0
0 0 0 0 0 0 0 0 0 0 0 0 0
0 0 0 0 0 0 0 0 0 0 0 0 0
0 0 0 0 0 0 0 0 0 0 0 0 0
0 0 0 0 0 0 0 0 0 0 0
"Viruses;Uroviricota;Caudoviricetes;Caudoviricetes__na;Caudoviricetes__na;Caudoviricetes__na;Freshwater_phage_uvFW-CGR-AMD-COM-C440" 0 0 0 0 12 3 0 0 0
0 0 0 0 0 0 0 0 0 0 0 0 0
0 0 0 0 0 0 0 0 0 0 0 0 0
0 0 0 0 0 0 0 0 0 0 0 0 0

0 0 0 0 0 0 0 0 0 0 0 0 0
0 0 0 0 0 0 0 0 0 0 0
"Viruses;Uroviricota;Caudoviricetes;Caudoviricetes__na;Caudoviricetes__na;Caudoviricetes__na;Freshwater_phage_uvFW-CGR-AMD-COM-C455" 0 0 0 0 1 0 0 0 0
0 0 0 0 0 0 0 0 0 0 0 0 0
0 0 0 0 0 0 0 0 0 0 0 0 0
0 0 0 0 0 0 0 0 0 0 0 0 0
0 0 0 0 0 0 0 0 0 0 0 0 0
0 0 0 0 0 0 0 0 0 0 0
"Viruses;Uroviricota;Caudoviricetes;Caudoviricetes__na;Caudoviricetes__na;Caudoviricetes__na;Geobacillus_phage_vB_GthS_PK3.6" 0 0 0 0 0 2 0 0 0 0 0
0 0 0 0 0 1 0 0 0 0 0 0 0
0 0 0 0 0 0 0 0 0 0 0 0 0
0 0 0 0 0 0 0 0 0 0 0 0 0
0 0 0 0 0 0 0 0 0 0 0 0 0
0 0 0 0 0 0 0 0 0
"Viruses;Uroviricota;Caudoviricetes;Caudoviricetes__na;Caudoviricetes__na;Caudoviricetes__na;Geobacillus_phage_vB_GthS_PT9.1" 0 0 0 0 0 1 0 0 0 0 0
0 0 0 0 0 0 0 0 0 0 0 0 0
0 0 0 0 0 0 0 0 0 0 0 0 0
0 0 0 0 0 0 0 0 0 0 0 0 0
0 0 0 0 0 0 0 0 0 0 0 0 0
0 0 0 0 0 0 0 0 0
"Viruses;Uroviricota;Caudoviricetes;Caudoviricetes__na;Caudoviricetes__na;Caudoviricetes__na;Geobacillus_virus_E3" 0 0 0 0 6 0 0 0 0 0 0 0 0
0 0 0 0 0 0 0 0 0 0 0 0 0
0 0 0 0 0 0 0 0 0 0 0 0 0
0 0 0 0 0 0 0 0 0 0 0 0 0
0 0 0 0 0 0 0 0 0 0 0 0 0
0 0 0 0 0 0 0
"Viruses;Uroviricota;Caudoviricetes;Caudoviricetes__na;Caudoviricetes__na;Caudoviricetes__na;Gordonia_phage_Camerico" 0 0 0 1 0 0 0 0 0 0 0 0
0 0 0 0 0 0 0 0 0 0 0 0 0
0 0 0 0 0 0 0 0 0 0 0 0 0
0 0 0 0 0 0 0 0 0 0 0 0 0
0 0 0 0 0 0 0 0 0 0 0 0 0
0 0 0 0 0 0 0 0
"Viruses;Uroviricota;Caudoviricetes;Caudoviricetes__na;Caudoviricetes__na;Caudoviricetes__na;Idiomarinaceae_phage_1N2-2" 0 0 1 1 1 12 0 0 0 0 0
0 0 0 0 0 0 2 0 0 0 0 0 0
0 0 0 0 0 0 0 0 0 0 0 0 0
0 0 0 0 0 0 0 0 0 0 0 0 0
0 0 0 0 0 0 0 0 0 0 0 0 0
0 0 0 0 0 0 0 0 0
"Viruses;Uroviricota;Caudoviricetes;Caudoviricetes__na;Caudoviricetes__na;Caudoviricetes__na;Idiomarinaceae_phage_Phi1M2-2" 0 0 1 0 0 0 0 0 0 0 0
0 0 0 0 0 0 0 0 0 0 0 0 0
0 0 0 0 0 0 0 0 0 0 0 0 0
0 0 0 0 0 0 0 0 0 0 1 0 0

0 0 0 0 0 0 0 0 0 0 0 0 0
0 0 0 0 0 0 0 0 0
"Viruses;Uroviricota;Caudoviricetes;Caudoviricetes__na;Caudoviricetes__na;Caudoviricetes__na;Klebsiella_phage_KP13-16" 0 0 0 0 0 1 0 0 0 0 0 0
0 0 0 0 0 0 0 0 0 0 0 0 0
0 0 0 0 0 0 0 0 0 0 0 0 0
0 0 0 0 0 0 0 0 0 0 0 0 0
0 0 0 0 0 0 0 0 0 0 0 0 0
0 0 0 0 0 0 0 0
"Viruses;Uroviricota;Caudoviricetes;Caudoviricetes__na;Caudoviricetes__na;Caudoviricetes__na;Klebsiella_phage_KP13-26" 0 0 0 1 0 0 0 0 0 0 0 0
0 0 0 0 0 0 0 0 0 0 0 0 0
0 0 0 0 0 0 0 0 0 0 0 0 0
0 0 0 0 0 0 0 0 0 0 0 0 0
0 0 0 0 0 0 0 0 0 0 0 0 0
0 0 0 0 0 0 0 0
"Viruses;Uroviricota;Caudoviricetes;Caudoviricetes__na;Caudoviricetes__na;Caudoviricetes__na;Klebsiella_phage_vB_KaeS_Diencephalon" 0 1 0 0 0 0 0 0 0 0
0 0 0 0 0 0 0 0 0 0 0 0 0
0 0 0 0 0 0 0 0 0 0 0 0 0
0 0 0 0 0 0 0 0 0 0 0 0 0
0 0 0 0 0 0 0 0 0 0 0 0 0
0 0 0 0 0 0 0 0 0 0
"Viruses;Uroviricota;Caudoviricetes;Caudoviricetes__na;Caudoviricetes__na;Caudoviricetes__na;Klebsiella_phage_vB_KpM_FBKp34" 0 0 0 0 0 1 0 0 0 0 0
0 0 0 0 0 0 0 0 0 0 0 0 0
0 0 0 0 0 0 0 0 0 0 0 0 0
0 0 0 0 0 0 0 0 0 0 0 0 0
0 0 0 0 0 0 0 0 0 0 0 0 0
0 0 0 0 0 0 0 0 0
"Viruses;Uroviricota;Caudoviricetes;Caudoviricetes__na;Caudoviricetes__na;Caudoviricetes__na;Kosakonia_phage_Kc166B" 0 0 1 0 5 0 0 0 0 0 0 0
0 0 0 0 0 0 0 0 0 0 0 0 0
0 0 0 0 0 0 0 0 0 0 0 0 0
0 0 0 0 0 0 0 0 0 0 0 0 0
0 0 0 0 0 0 0 0 0 0 0 0 0
0 0 0 0 0 0 0 0
"Viruses;Uroviricota;Caudoviricetes;Caudoviricetes__na;Caudoviricetes__na;Caudoviricetes__na;Lactobacillus_phage_ATCCB" 0 0 0 0 0 0 0 0 0 0 0 0
0 0 0 0 0 0 0 0 0 0 0 0 0
0 0 0 0 0 0 0 0 0 0 0 0 0
0 0 0 0 0 0 0 0 0 0 0 0 0
0 0 0 0 2 0 0 0 0 0 0 0 0
0 0 0 0 0 0 0 0
"Viruses;Uroviricota;Caudoviricetes;Caudoviricetes__na;Caudoviricetes__na;Caudoviricetes__na;Lactococcus_phage_38502" 0 0 0 0 0 0 0 0 0 0 0 0
0 0 0 0 0 0 0 0 0 0 0 0 0
0 0 0 0 0 1 0 0 0 0 0 0 0
0 0 0 5 0 0 0 0 0 0 0 0 0

0 0 0 0 0 0 0 0 0 0 0 0 0
0 0 0 0 0 0 0 0
"Viruses;Uroviricota;Caudoviricetes;Caudoviricetes__na;Caudoviricetes__na;Caudoviricetes__na;Lactococcus_p
hage_98204" 0 0 0 0 0 0 0 0 0 0 0 0
0 0 0 0 0 0 0 0 0 0 0 0 0
0 0 0 0 0 0 0 0 0 0 0 0 0
0 0 0 1 0 0 0 0 0 0 0 0 0
0 0 0 0 0 0 0 0 0 0 0 0 0
0 0 0 0 0 0 0 0
"Viruses;Uroviricota;Caudoviricetes;Caudoviricetes__na;Caudoviricetes__na;Caudoviricetes__na;Lactococcus_p
hage_bIL286" 0 0 0 0 0 0 0 0 0 0 0 0
0 0 0 0 0 0 0 0 0 0 0 0 0
0 0 0 0 0 0 1 0 0 0 0 0 0
0 0 0 0 0 0 0 0 0 0 0 0 0
0 0 0 0 0 0 0 0 0 0 0 0 0
0 0 0 0 0 0 0 0
"Viruses;Uroviricota;Caudoviricetes;Caudoviricetes__na;Caudoviricetes__na;Caudoviricetes__na;Lactococcus_p
hage_C41431" 0 0 0 0 0 0 0 0 0 0 0 0
0 0 0 0 0 0 0 0 0 0 0 0 0
0 0 0 0 0 0 0 0 0 0 3 0 0
0 0 0 2 0 0 0 0 0 0 0 0 0
0 0 0 0 0 0 0 0 0 0 0 0 0
0 0 0 0 0 0 0 0
"Viruses;Uroviricota;Caudoviricetes;Caudoviricetes__na;Caudoviricetes__na;Caudoviricetes__na;Lactococcus_p
hage_CHPC836" 0 0 0 0 0 0 0 0 0 0 0
0 0 0 0 0 0 0 0 0 0 0 0 0
0 0 0 0 0 0 1 0 0 0 0 0 0
0 0 0 0 0 0 0 0 0 0 0 0 0
0 0 0 0 0 0 0 0 0 0 0 0 0
0 0 0 0 0 0 0 0 0
"Viruses;Uroviricota;Caudoviricetes;Caudoviricetes__na;Caudoviricetes__na;Caudoviricetes__na;Lactococcus_p
hage_D4840" 0 0 0 0 0 0 0 0 0 0 0 0
0 0 0 0 0 0 0 0 0 0 0 0 0
0 0 0 0 0 0 1 0 0 0 0 0 0
0 0 0 0 0 0 0 0 0 0 0 0 0
0 0 0 0 0 0 0 0 0 0 0 0 0
0 0 0 0 0 0 0 0
"Viruses;Uroviricota;Caudoviricetes;Caudoviricetes__na;Caudoviricetes__na;Caudoviricetes__na;Lactococcus_p
hage_phi31" 0 0 0 0 0 0 0 0 0 0 0 0
0 0 0 0 0 0 0 0 0 0 0 0 0
0 0 0 0 0 0 0 0 0 0 1 0 0
0 0 0 5 0 0 0 0 0 0 0 0 0
0 0 0 0 0 0 0 0 0 0 0 0 0
0 0 0 0 0 0 0 0
"Viruses;Uroviricota;Caudoviricetes;Caudoviricetes__na;Caudoviricetes__na;Caudoviricetes__na;Lake_Baikal_p
hage_Baikal-20-5m-C28" 0 0 0 0 29 16 0 0 0 0
0 0 0 0 0 0 0 0 0 0 0 0 0
0 0 0 0 0 0 0 0 0 0 0 0 0
0 0 0 0 0 0 0 0 0 0 0 0 0

0	0	0	0	0	0	0	0	0	0	0	0	0
0	0	0	0	0	0	0	0	0	0
"Viruses;Uroviricota;Caudoviricetes;Caudoviricetes__na;Caudoviricetes__na;Caudoviricetes__na;Luteibacter_p
hage_vB_LflM-Pluto"	0	0	0	0	2	0	0	0	0	0	0
0	0	0	0	0	0	0	0	0	0	0	0	0
0	0	0	0	0	0	0	0	0	0	0	0	0
0	0	0	0	0	0	0	0	0	0	0	0	0
0	0	0	0	0	0	0	0	0	0	0	0	0
0	0	0	0	0	0	0	0	0
"Viruses;Uroviricota;Caudoviricetes;Caudoviricetes__na;Caudoviricetes__na;Caudoviricetes__na;Lysinibacillus_
phage_LC76P1"	0	0	0	0	0	1	0	0	0	0	0	0
0	0	0	0	0	0	0	0	0	0	0	0	0
0	0	0	0	0	0	0	0	0	0	0	0	0
0	0	0	0	0	0	0	0	0	0	0	0	0
0	0	0	0	0	0	0	0	0	0	0	0	0
0	0	0	0	0	0	0	0
"Viruses;Uroviricota;Caudoviricetes;Caudoviricetes__na;Caudoviricetes__na;Caudoviricetes__na;Methylophilac
eae_phage_P19250A"	0	0	0	0	1	8	0	0	0	0	0
0	0	0	0	0	0	0	0	0	0	0	0	1
1	0	0	0	0	0	0	0	0	0	0	0	0
0	0	0	0	0	0	0	0	0	0	0	0	0
0	0	0	0	0	0	0	0	0	0	0	0	0
0	0	0	0	0	0	0	0	0
"Viruses;Uroviricota;Caudoviricetes;Caudoviricetes__na;Caudoviricetes__na;Caudoviricetes__na;Methylophilal
es_phage_MEP301"	0	0	0	1	0	0	0	0	0	0	0
0	0	0	0	0	0	0	0	0	0	0	0	0
0	0	0	0	0	0	0	0	0	0	0	0	0
0	0	0	0	0	0	0	0	0	0	0	0	0
0	0	0	0	0	0	0	0	0	0	0	0	0
0	0	0	0	0	0	0	0	0
"Viruses;Uroviricota;Caudoviricetes;Caudoviricetes__na;Caudoviricetes__na;Caudoviricetes__na;Methylophilal
es_phage_Venkman_EXVC282S"	0	0	0	0	0	7	0	0	0
0	0	0	0	0	0	0	0	0	0	0	0	0
0	0	0	0	0	0	0	0	0	0	0	0	0
0	0	0	0	0	0	0	0	0	0	0	0	0
0	0	0	0	0	0	0	0	0	0	0	0	0
0	0	0	0	0	0	0	0	0	0	0
"Viruses;Uroviricota;Caudoviricetes;Caudoviricetes__na;Caudoviricetes__na;Caudoviricetes__na;Microbacteriu
m_phage_Count"	0	0	0	0	0	0	0	0	0	0	0
0	0	0	1	0	0	0	0	0	0	0	0	0
0	0	0	0	0	0	0	0	0	0	0	0	0
0	0	0	0	0	0	0	0	0	0	0	0	0
0	0	0	0	0	0	0	0	0	0	0	0	0
0	0	0	0	0	0	0	0	0
"Viruses;Uroviricota;Caudoviricetes;Caudoviricetes__na;Caudoviricetes__na;Caudoviricetes__na;Microbacteriu
m_phage_DizzyRudy"	0	0	0	0	0	1	0	0	0	0	0
0	0	0	0	0	0	0	0	0	0	0	0	0
0	0	0	0	0	0	0	0	0	0	0	0	0
0	0	0	0	0	0	0	0	0	0	0	0	0

0 0 0 0 0 0 0 0 0 0 0 0 0
0 0 0 0 0 0 0 0 0
"Viruses;Uroviricota;Caudoviricetes;Caudoviricetes__na;Caudoviricetes__na;Caudoviricetes__na;Microbacterium_phage_Lupine" 0 0 0 0 0 1 0 0 0 0 0
0 0 0 0 0 0 0 0 0 0 0 0 0
0 0 0 0 0 0 0 0 0 0 0 0 0
0 0 0 0 0 0 0 0 0 0 0 0 0
0 0 0 0 0 0 0 0 0 0 0 0 0
0 0 0 0 0 0 0 0 0
"Viruses;Uroviricota;Caudoviricetes;Caudoviricetes__na;Caudoviricetes__na;Caudoviricetes__na;Microbacterium_phage_Pumpernickel" 0 0 0 2 14 2 0 0 0 0
0 0 0 0 0 0 0 0 0 0 0 0 0
0 0 0 0 0 0 0 0 0 0 0 0 0
0 0 0 0 0 0 0 0 0 0 0 0 0
0 0 0 0 0 0 0 0 0 0 0 0 0
0 0 0 0 0 0 0 0 0 0
"Viruses;Uroviricota;Caudoviricetes;Caudoviricetes__na;Caudoviricetes__na;Caudoviricetes__na;Microcystis_phage_Me-ZS1" 0 0 0 1 0 0 0 0 0 0 0 0
0 0 0 0 0 0 0 0 0 0 0 0 0
0 0 0 0 0 0 0 0 0 0 0 0 0
0 0 0 0 0 0 0 0 0 0 0 0 0
0 0 0 0 0 0 0 0 0 0 0 0 0
0 0 0 0 0 0 0 0
"Viruses;Uroviricota;Caudoviricetes;Caudoviricetes__na;Caudoviricetes__na;Caudoviricetes__na;Mu-like_cryoconite_phage_AB09" 0 0 0 0 2 0 0 0 0 0
0 0 0 0 0 0 0 0 0 0 0 0 0
0 0 0 0 0 0 0 0 0 0 0 0 0
0 0 0 0 0 0 0 0 0 0 0 0 0
0 0 0 0 0 0 0 0 0 0 0 0 0
0 0 0 0 0 0 0 0 0 0
"Viruses;Uroviricota;Caudoviricetes;Caudoviricetes__na;Caudoviricetes__na;Caudoviricetes__na;Mycobacterium_phage_Boilgate" 0 0 0 1 0 0 0 0 0 0 0
0 0 0 0 0 0 0 0 0 0 0 0 0
0 0 0 0 0 0 0 0 0 0 0 0 0
0 0 0 0 0 0 0 0 0 0 0 0 0
0 0 0 0 0 0 0 0 0 0 0 0 0
0 0 0 0 0 0 0 0 0
"Viruses;Uroviricota;Caudoviricetes;Caudoviricetes__na;Caudoviricetes__na;Caudoviricetes__na;Mycobacterium_phage_NoShow" 0 0 0 0 0 0 0 0 0 0 0
0 0 0 0 0 0 0 0 0 0 0 1 0
0 0 0 0 0 0 0 0 0 0 0 0 0
0 0 0 0 0 0 0 0 0 0 0 0 0
0 0 0 0 0 0 0 0 0 0 0 0 0
0 0 0 0 0 0 0 0 0
"Viruses;Uroviricota;Caudoviricetes;Caudoviricetes__na;Caudoviricetes__na;Caudoviricetes__na;Mycobacterium_phage_Sejanus" 0 0 0 0 0 0 0 0 0 1 0
0 0 0 0 0 0 0 0 0 0 0 0 0
0 0 0 0 0 0 0 0 0 0 0 0 0
0 0 0 0 0 0 0 0 0 0 0 0 0

0 0 0 0 0 0 0 0 0 0 0 0 0
0 0 0 0 0 0 0 0 0
"Viruses;Uroviricota;Caudoviricetes;Caudoviricetes__na;Caudoviricetes__na;Caudoviricetes__na;Myoviridae_environmental_samples" 0 0 0 2 44 0 0 0 0 2 0
0 0 0 0 0 0 0 0 0 0 0 0 0
0 0 0 0 0 0 0 0 0 0 0 0 0
0 0 0 0 0 0 0 0 0 0 0 0 0
0 0 0 0 0 0 0 0 0 0 0 0 0
0 0 0 0 0 0 0 0 0
"Viruses;Uroviricota;Caudoviricetes;Caudoviricetes__na;Caudoviricetes__na;Caudoviricetes__na;Myoviridae_sp._ct04y17" 0 0 0 0 0 0 0 0 0 0 0 0
0 0 0 0 0 0 0 0 0 0 0 0 0
0 0 0 0 0 0 0 0 0 0 0 0 0
0 0 0 0 0 0 0 0 0 0 0 0 0
0 0 110 0 2 0 0 0 0 0 0 0 0
0 0 20 18 1 4 0 0
"Viruses;Uroviricota;Caudoviricetes;Caudoviricetes__na;Caudoviricetes__na;Caudoviricetes__na;Myoviridae_sp._ct0e511" 0 0 0 0 0 0 0 0 0 0 0 0
4 5 2 4 1 0 1 0 1 0 0 1 2
0 0 0 0 0 1 0 0 0 0 0 1 0
0 0 0 0 9 0 0 0 0 0 2 2 0
0 0 0 0 0 0 0 0 0 3 0 0 0
0 0 5 2 0 0 0 0
"Viruses;Uroviricota;Caudoviricetes;Caudoviricetes__na;Caudoviricetes__na;Caudoviricetes__na;Myoviridae_sp._ct0Tg8" 0 0 0 0 0 0 0 0 0 0 0 0
6 7 15 11 5 10 2 0 1 0 0 0 6
2 2 0 0 0 34 43 0 0 2 0 21 35
0 0 0 0 2 0 0 0 0 0 1 0 0
0 0 0 5 7 0 0 0 0 1 1 0 0
0 0 4 2 0 0 0 0
"Viruses;Uroviricota;Caudoviricetes;Caudoviricetes__na;Caudoviricetes__na;Caudoviricetes__na;Myoviridae_sp._ct1CM14" 0 0 0 0 0 0 0 0 0 0 0 0
0 0 0 0 0 0 0 0 0 0 0 0 0
0 0 0 0 0 0 0 0 0 0 0 0 0
0 0 0 0 0 0 0 0 0 0 0 0 0
0 0 0 4 3 0 0 0 0 0 0 0 0
0 0 0 0 0 0 0 0
"Viruses;Uroviricota;Caudoviricetes;Caudoviricetes__na;Caudoviricetes__na;Caudoviricetes__na;Myoviridae_sp._ct1TR10" 0 0 0 0 0 0 0 0 0 0 0 0
0 0 0 0 0 0 0 0 0 0 0 0 0
0 0 0 0 0 0 2 0 0 7 0 0 0
0 0 0 0 0 0 0 0 0 0 0 0 0
0 0 0 0 0 0 0 0 0 0 0 0 0
0 0 0 0 0 0 0 0
"Viruses;Uroviricota;Caudoviricetes;Caudoviricetes__na;Caudoviricetes__na;Caudoviricetes__na;Myoviridae_sp._ct25F5" 0 0 0 0 0 0 0 0 0 0 0 0
0 0 0 0 0 0 0 0 0 0 0 0 0
0 0 0 0 0 0 0 0 0 0 0 3 0
0 0 0 0 0 0 0 0 0 0 0 0 0

0 0 0 0 0 0 0 0 0 0 0 0 0
0 0 0 0 0 0 0 0
"Viruses;Uroviricota;Caudoviricetes;Caudoviricetes__na;Caudoviricetes__na;Caudoviricetes__na;Myoviridae_s
p._ct2cn10" 0 0 0 0 0 0 0 0 0 0 0 0
0 0 0 0 0 0 0 0 0 0 0 0 0
0 0 0 0 0 0 0 0 0 0 0 0 0
0 0 0 0 0 0 0 0 0 0 0 0 0
0 0 0 1 0 0 0 0 0 0 0 0 0
0 0 0 0 0 0 0 0
"Viruses;Uroviricota;Caudoviricetes;Caudoviricetes__na;Caudoviricetes__na;Caudoviricetes__na;Myoviridae_s
p._ct2DO6" 0 0 0 0 0 0 0 0 0 0 0 0
0 1 0 0 0 0 1 0 0 0 0 0 0
0 0 0 0 0 0 0 0 0 0 0 0 0
0 0 0 0 0 0 0 0 0 0 2 0 0
0 0 0 0 0 0 0 0 0 0 0 0 0
0 0 0 1 0 0 0 0
"Viruses;Uroviricota;Caudoviricetes;Caudoviricetes__na;Caudoviricetes__na;Caudoviricetes__na;Myoviridae_s
p._ct2Pw37" 0 0 0 2 0 0 0 0 0 0 0 0
0 0 0 0 0 0 0 0 0 0 0 0 0
0 0 0 0 0 0 0 0 0 0 0 0 0
0 0 0 0 0 0 0 0 6 0 0 0 0
0 0 0 141 192 1 0 0 0 0 0 0 0
0 0 0 0 0 0 0 0
"Viruses;Uroviricota;Caudoviricetes;Caudoviricetes__na;Caudoviricetes__na;Caudoviricetes__na;Myoviridae_s
p._ct2Qy24" 0 0 0 0 0 0 0 0 0 0 0 0
0 0 0 0 0 0 0 0 0 0 0 0 0
0 0 0 0 0 0 0 0 0 0 0 0 0
0 0 0 0 0 0 0 0 0 0 0 0 0
0 0 0 0 0 0 0 0 0 0 0 0 0
0 0 0 1 0 0 0 0
"Viruses;Uroviricota;Caudoviricetes;Caudoviricetes__na;Caudoviricetes__na;Caudoviricetes__na;Myoviridae_s
p._ct2th6" 0 0 0 0 0 0 0 0 0 0 0 0
0 0 0 0 0 0 0 0 0 0 0 0 1
1 0 1 0 0 6 7 2 3 0 0 1 1
0 0 0 0 0 0 0 0 0 0 0 0 0
0 0 0 0 0 0 0 0 0 0 0 0 0
0 0 0 0 0 1 0 0
"Viruses;Uroviricota;Caudoviricetes;Caudoviricetes__na;Caudoviricetes__na;Caudoviricetes__na;Myoviridae_s
p._ct3D84" 0 0 0 0 0 0 0 0 0 0 0 0
5 7 0 0 0 0 0 0 0 0 0 0 0
0 0 0 0 0 0 0 0 0 0 0 1 0
0 0 0 0 0 0 0 0 0 0 0 0 0
0 0 0 0 0 0 0 0 0 0 0 0 0
0 0 0 0 0 0 0 0
"Viruses;Uroviricota;Caudoviricetes;Caudoviricetes__na;Caudoviricetes__na;Caudoviricetes__na;Myoviridae_s
p._ct3hC12" 0 0 0 0 0 0 0 0 0 0 0 0
0 3 0 0 0 0 0 0 0 0 0 0 0
0 0 0 0 0 0 0 0 0 0 0 0 0
0 0 0 0 0 0 0 0 0 0 0 0 0

0	0	0	1	0	0	0	0	0	2	1	0	0
0	0	0	1	0	0	0	0
"Viruses;Uroviricota;Caudoviricetes;Caudoviricetes__na;Caudoviricetes__na;Caudoviricetes__na;Myoviridae_sp._ct3Oc10"	0	0	0	0	0	0	0	0	0	0	0	0
0	0	0	0	0	0	1	1	0	0	0	0	0
0	0	0	0	0	0	1	0	0	58	0	0	0
0	0	0	0	0	0	0	0	0	0	0	0	0
0	0	0	0	0	0	0	0	0	0	0	0	0
0	0	0	0	0	0	0	0
"Viruses;Uroviricota;Caudoviricetes;Caudoviricetes__na;Caudoviricetes__na;Caudoviricetes__na;Myoviridae_sp._ct3Sw5"	0	0	0	0	0	0	0	0	0	0	0	0
1	2	3	0	3	2	2	4	0	0	0	1	5
5	3	1	2	0	0	0	0	0	0	0	1	0
0	0	0	0	3	7	3	0	0	0	0	0	0
0	0	41	0	0	0	0	0	0	24	8	0	0
0	0	1	1	0	1	0	0
"Viruses;Uroviricota;Caudoviricetes;Caudoviricetes__na;Caudoviricetes__na;Caudoviricetes__na;Myoviridae_sp._ct3tv2"	0	0	0	0	0	0	0	0	0	0	0	0
11	6	16	22	21	162	0	0	0	0	0	0	0
0	0	0	0	0	0	0	0	0	0	0	32	21
5	1	10	57	9	10	72	16	0	2	0	0	0
0	8	0	12	18	3	3	0	0	0	0	0	0
0	0	0	0	0	0	0	0
"Viruses;Uroviricota;Caudoviricetes;Caudoviricetes__na;Caudoviricetes__na;Caudoviricetes__na;Myoviridae_sp._ct49F125"	0	0	0	0	0	0	0	0	0	0	0	0
31	11	21	35	44	82	0	0	0	0	1	0	1
0	0	0	0	0	0	0	0	0	0	0	11	8
0	0	0	0	0	0	0	0	0	0	1	0	0
0	0	0	0	0	0	0	0	0	0	0	0	0
0	0	1	0	0	0	0	0
"Viruses;Uroviricota;Caudoviricetes;Caudoviricetes__na;Caudoviricetes__na;Caudoviricetes__na;Myoviridae_sp._ct4yW2"	0	0	0	0	0	0	0	0	0	0	0	0
10	10	13	9	3	38	0	3	0	0	0	0	0
0	0	0	1	0	1	0	0	0	0	0	31	11
0	0	0	0	2	1	0	0	0	0	0	0	0
0	0	0	3	1	0	0	0	0	0	0	0	0
0	0	2	0	0	0	0	0
"Viruses;Uroviricota;Caudoviricetes;Caudoviricetes__na;Caudoviricetes__na;Caudoviricetes__na;Myoviridae_sp._ct55Z2"	0	0	0	0	0	0	0	0	0	0	0	0
0	0	0	0	0	0	0	0	0	0	0	0	0
0	0	0	0	0	0	0	0	0	0	0	0	0
0	0	0	0	0	1	1	0	0	0	0	0	0
0	0	0	1	0	0	0	0	0	0	0	0	0
0	0	0	0	0	0	0	0
"Viruses;Uroviricota;Caudoviricetes;Caudoviricetes__na;Caudoviricetes__na;Caudoviricetes__na;Myoviridae_sp._ct5hB2"	0	0	0	0	0	0	0	0	0	0	0	0
0	0	0	0	0	0	2	0	0	0	0	0	0
0	0	0	0	0	0	0	0	0	1	0	0	0
0	0	0	0	2	3	0	0	0	0	0	0	0

0 0 0 0 0 0 0 0 0 0 0 0 0
0 0 0 0 0 0 0 0
"Viruses;Uroviricota;Caudoviricetes;Caudoviricetes__na;Caudoviricetes__na;Caudoviricetes__na;Myoviridae_sp._ct5lt7" 0 0 0 0 0 0 0 0 0 0 0 0
0 0 0 0 0 0 0 0 0 0 0 0 0
0 2 1 0 0 0 0 0 0 0 0 0 0
0 0 0 0 0 0 3 0 0 0 0 0 20
9 0 0 0 0 0 0 1 0 0 0 0 0
0 0 0 0 0 0 40 0
"Viruses;Uroviricota;Caudoviricetes;Caudoviricetes__na;Caudoviricetes__na;Caudoviricetes__na;Myoviridae_sp._ct5MF11" 0 0 0 0 0 0 0 0 0 0 0 0
0 0 0 0 0 0 0 0 0 0 0 0 0
0 0 0 0 0 0 0 0 0 0 0 0 0
0 0 0 0 0 0 0 0 4 0 0 0 0
0 0 0 0 0 1 1 0 0 0 0 0 0
0 0 0 0 0 0 0 0
"Viruses;Uroviricota;Caudoviricetes;Caudoviricetes__na;Caudoviricetes__na;Caudoviricetes__na;Myoviridae_sp._ct5nJ10" 0 0 0 0 0 0 0 0 0 0 0 0
0 0 0 0 0 0 0 0 0 0 0 0 0
0 0 0 0 0 0 0 0 0 0 0 0 0
0 0 0 0 0 0 0 0 0 0 1 2 0
0 0 0 0 0 0 0 0 0 0 0 0 0
0 0 0 0 0 0 0 0
"Viruses;Uroviricota;Caudoviricetes;Caudoviricetes__na;Caudoviricetes__na;Caudoviricetes__na;Myoviridae_sp._ct5ra14" 0 0 0 0 0 0 0 0 0 0 0 0
1 1 0 0 0 0 0 0 0 0 0 0 3
3 0 0 1 0 0 0 0 0 0 0 0 0
0 0 0 0 0 0 0 0 0 0 5 3 0
0 0 0 1 0 0 0 0 0 0 0 0 0
0 0 0 0 0 0 0 0
"Viruses;Uroviricota;Caudoviricetes;Caudoviricetes__na;Caudoviricetes__na;Caudoviricetes__na;Myoviridae_sp._ct5Tq8" 0 0 0 0 0 0 0 0 0 0 0 0
0 0 0 0 0 0 0 0 0 0 0 0 0
0 0 0 0 0 0 0 0 0 0 0 0 0
0 0 0 0 0 0 0 0 0 0 0 0 0
0 0 0 0 0 0 0 0 0 0 0 0 0
0 0 1 3 0 0 0 0
"Viruses;Uroviricota;Caudoviricetes;Caudoviricetes__na;Caudoviricetes__na;Caudoviricetes__na;Myoviridae_sp._ct5xZ3" 0 0 0 0 0 0 0 0 0 0 0 0
0 0 0 0 0 0 0 0 0 0 0 0 0
0 0 0 0 0 0 0 0 0 0 0 1 0
0 1 0 0 0 0 0 0 0 0 0 0 0
0 0 1 0 0 0 0 0 0 0 0 0 0
0 0 0 0 0 0 0 0
"Viruses;Uroviricota;Caudoviricetes;Caudoviricetes__na;Caudoviricetes__na;Caudoviricetes__na;Myoviridae_sp._ct6aW5" 0 0 0 0 0 0 0 0 0 0 0 0
3 3 0 1 1 0 0 0 0 0 0 0 0
0 0 0 0 0 0 0 0 0 0 0 0 0
0 0 0 0 0 0 0 0 0 0 0 0 0

0 0 0 0 0 0 0 0 0 0 0 0 0
0 0 0 0 0 0 0 0
"Viruses;Uroviricota;Caudoviricetes;Caudoviricetes__na;Caudoviricetes__na;Caudoviricetes__na;Myoviridae_sp._ct6eX13" 0 0 0 0 0 0 0 0 0 0 0 0
0 0 0 0 0 0 0 0 0 0 0 0 0
0 0 0 0 0 0 0 0 0 0 0 0 0
0 0 0 0 0 0 0 0 0 0 0 0 0
0 0 0 1 0 1 0 0 0 0 0 0 0
0 0 0 0 0 0 0 0
"Viruses;Uroviricota;Caudoviricetes;Caudoviricetes__na;Caudoviricetes__na;Caudoviricetes__na;Myoviridae_sp._ct6nn14" 0 0 0 0 0 0 0 0 0 0 0 0
9 22 4 1 0 2 0 0 0 0 0 0 0
0 0 0 0 0 0 0 0 0 0 0 0 0
0 0 0 0 0 0 0 0 0 0 0 0 0
0 0 0 9 8 0 0 0 0 0 0 0 0
0 0 0 0 0 0 0 0
"Viruses;Uroviricota;Caudoviricetes;Caudoviricetes__na;Caudoviricetes__na;Caudoviricetes__na;Myoviridae_sp._ct7113" 0 0 0 0 0 0 0 0 0 0 0 0
0 0 0 0 0 0 0 0 0 0 0 0 0
0 0 0 0 0 17 18 4 1 104 24 1 1
0 0 0 0 0 0 0 0 0 0 0 0 0
0 0 0 0 1 0 0 0 0 0 0 0 0
0 0 0 0 0 0 0 0
"Viruses;Uroviricota;Caudoviricetes;Caudoviricetes__na;Caudoviricetes__na;Caudoviricetes__na;Myoviridae_sp._ct78050" 0 0 0 0 0 0 0 0 0 0 0 0
0 0 0 0 0 0 0 7 0 0 1 0 0
0 0 0 0 0 0 0 0 0 0 0 0 0
0 0 0 0 0 0 0 0 0 0 0 0 0
0 0 0 0 1 0 0 0 0 0 0 0 0
0 0 0 0 0 0 0 0
"Viruses;Uroviricota;Caudoviricetes;Caudoviricetes__na;Caudoviricetes__na;Caudoviricetes__na;Myoviridae_sp._ct7CH26" 0 0 0 0 0 0 0 0 0 0 0 0
20 10 18 24 22 35 2 4 1 0 0 0 0
0 1 0 0 0 2 1 0 0 0 0 7 4
0 0 0 0 4 4 0 0 0 0 3 2 0
0 0 1 0 0 0 0 0 0 12 3 0 0
0 0 23 12 0 3 0 0
"Viruses;Uroviricota;Caudoviricetes;Caudoviricetes__na;Caudoviricetes__na;Caudoviricetes__na;Myoviridae_sp._ct7Q419" 0 0 0 0 0 0 0 0 0 0 0 0
0 1 0 0 0 0 0 0 0 0 0 0 0
0 0 0 0 0 0 0 0 0 0 0 5 2
0 0 0 1 0 0 0 0 0 0 0 0 0
0 0 0 3 14 0 0 0 0 0 0 0 0
0 0 27 32 0 2 0 0
"Viruses;Uroviricota;Caudoviricetes;Caudoviricetes__na;Caudoviricetes__na;Caudoviricetes__na;Myoviridae_sp._ct89I2" 0 0 0 0 0 0 0 0 0 0 0 0
0 0 0 0 0 0 0 0 0 0 0 0 0
0 0 0 0 0 0 0 0 0 0 0 0 0
0 0 0 0 0 0 0 0 0 0 0 0 0

0 0 0 0 2 0 0 0 0 0 0 0 0
0 0 0 0 0 0 0 0
"Viruses;Uroviricota;Caudoviricetes;Caudoviricetes__na;Caudoviricetes__na;Caudoviricetes__na;Myoviridae_s
p._ct8Eu10" 0 0 0 0 0 0 0 0 0 0 0 0
0 0 0 0 0 0 3 2 0 0 1 0 0
0 0 0 0 0 0 0 0 0 0 0 0 0
0 0 0 0 0 0 0 0 0 0 1 3 0
0 0 170 0 0 0 0 0 0 0 0 0 0
0 0 0 0 0 0 0 0
"Viruses;Uroviricota;Caudoviricetes;Caudoviricetes__na;Caudoviricetes__na;Caudoviricetes__na;Myoviridae_s
p._ct8iP21" 0 0 0 0 0 0 0 0 0 0 0 0
0 2 0 0 0 0 0 0 0 0 0 0 0
0 0 0 0 0 0 0 0 0 0 0 0 0
0 0 0 0 0 0 0 0 0 0 0 0 0
0 0 0 0 0 0 0 0 0 0 0 0 0
0 0 0 0 0 0 0 0
"Viruses;Uroviricota;Caudoviricetes;Caudoviricetes__na;Caudoviricetes__na;Caudoviricetes__na;Myoviridae_s
p._ct8mY9" 0 0 0 0 0 0 0 0 0 0 0 0
1 0 0 0 0 0 0 0 0 0 0 0 0
0 1 0 0 0 0 0 0 0 0 0 0 1
0 0 0 0 0 0 0 0 0 0 0 0 0
0 0 0 0 1 0 0 0 0 0 0 0 0
0 0 0 1 0 0 0 0
"Viruses;Uroviricota;Caudoviricetes;Caudoviricetes__na;Caudoviricetes__na;Caudoviricetes__na;Myoviridae_s
p._ct9dX1" 0 0 0 0 0 0 0 0 0 0 0 0
0 0 0 0 0 0 0 0 0 0 0 0 0
0 0 0 0 0 0 0 0 0 0 0 0 0
0 0 0 0 1 1 0 0 0 0 0 0 0
0 0 0 0 0 0 0 0 0 5 3 0 0
0 0 32 34 0 0 0 0
"Viruses;Uroviricota;Caudoviricetes;Caudoviricetes__na;Caudoviricetes__na;Caudoviricetes__na;Myoviridae_s
p._ct9Fw19" 0 0 0 0 0 0 0 0 0 0 0 0
0 0 0 0 0 0 0 0 0 0 0 0 0
0 0 0 0 0 0 0 0 0 0 0 0 0
0 0 0 0 0 0 0 0 0 0 0 0 0
0 0 0 0 2 0 0 0 0 0 0 0 0
0 0 0 0 0 0 0 0
"Viruses;Uroviricota;Caudoviricetes;Caudoviricetes__na;Caudoviricetes__na;Caudoviricetes__na;Myoviridae_s
p._ct9MV2" 0 0 0 0 0 0 0 0 0 0 0 0
3 6 0 0 0 0 0 3 0 0 0 0 4
6 1 0 0 0 1 0 0 0 0 0 0 0
0 0 0 0 0 1 0 0 0 0 3 1 0
1 0 0 2 0 0 0 0 0 0 0 0 0
0 0 1 0 0 0 0 0
"Viruses;Uroviricota;Caudoviricetes;Caudoviricetes__na;Caudoviricetes__na;Caudoviricetes__na;Myoviridae_s
p._ct9Ns12" 0 0 0 0 0 0 0 0 0 0 0 0
0 0 0 0 0 0 7 13 1 4 1 1 0
0 0 0 0 0 0 0 0 0 0 0 0 0
0 0 0 0 0 0 0 0 0 0 0 0 0

0 0 0 0 0 0 0 0 0 0 0 0 0
0 0 0 0 0 0 0 0
"Viruses;Uroviricota;Caudoviricetes;Caudoviricetes__na;Caudoviricetes__na;Caudoviricetes__na;Myoviridae_s
p._ct9Uc11" 0 0 0 0 0 0 0 0 0 0 0 0
0 0 0 0 0 0 0 0 0 0 0 0 0
0 0 0 0 0 1 0 0 0 0 0 0 0
0 0 0 0 0 0 0 0 0 0 0 0 0
0 0 0 0 0 0 0 0 0 0 0 0 0
0 0 0 0 0 0 0 0
"Viruses;Uroviricota;Caudoviricetes;Caudoviricetes__na;Caudoviricetes__na;Caudoviricetes__na;Myoviridae_s
p._ctA1z6" 0 0 0 0 0 0 0 0 0 0 0 0
1 0 0 0 0 0 0 0 0 0 0 0 0
0 0 0 0 0 0 1 0 0 0 0 0 0
0 0 0 0 0 0 0 0 0 0 0 1 0
0 0 0 1 0 0 0 0 0 0 0 0 0
0 0 0 0 0 0 0 0
"Viruses;Uroviricota;Caudoviricetes;Caudoviricetes__na;Caudoviricetes__na;Caudoviricetes__na;Myoviridae_s
p._cta6i12" 0 0 0 0 0 0 0 0 0 0 0 0
1 11 0 1 0 0 0 0 0 0 0 0 0
0 0 0 0 0 0 0 0 0 0 0 0 0
0 0 0 0 0 0 0 0 0 0 0 0 0
0 0 0 0 0 0 0 0 0 0 0 0 0
0 0 0 0 0 0 0 0
"Viruses;Uroviricota;Caudoviricetes;Caudoviricetes__na;Caudoviricetes__na;Caudoviricetes__na;Myoviridae_s
p._ctAbS6" 0 0 1 0 0 0 0 0 0 0 0 0
0 0 0 0 0 0 67 39 8 2 6 1 0
0 0 0 0 0 0 0 0 0 0 0 0 0
0 0 4 0 46 30 19 0 0 1 0 0 0
0 0 0 0 0 0 0 0 0 0 0 0 0
0 0 0 0 0 0 0 0
"Viruses;Uroviricota;Caudoviricetes;Caudoviricetes__na;Caudoviricetes__na;Caudoviricetes__na;Myoviridae_s
p._ctai52" 0 0 0 0 0 0 0 0 1 0 0 0
0 0 0 0 0 0 1 36 1 0 7 7 0
0 1 0 0 0 0 0 0 0 0 0 0 0
0 0 0 0 0 1 0 0 0 0 0 0 0
0 0 0 0 0 0 0 0 0 0 0 0 0
0 0 0 0 0 0 0 0
"Viruses;Uroviricota;Caudoviricetes;Caudoviricetes__na;Caudoviricetes__na;Caudoviricetes__na;Myoviridae_s
p._ctaMv1" 0 0 0 0 0 0 0 0 0 0 0 0
0 0 0 0 0 0 0 0 0 0 0 0 0
0 0 0 0 0 0 0 0 0 0 0 0 0
0 0 0 0 0 0 0 0 0 0 0 0 0
0 0 0 3 6 0 0 0 0 0 0 0 0
0 0 0 0 0 0 0 0
"Viruses;Uroviricota;Caudoviricetes;Caudoviricetes__na;Caudoviricetes__na;Caudoviricetes__na;Myoviridae_s
p._ctBbR2" 0 0 0 0 0 0 0 0 0 0 0 0
0 0 0 0 0 0 0 0 0 0 0 0 0
0 0 0 0 0 1 0 0 0 0 0 0 0
0 0 0 0 0 0 0 0 0 0 0 0 0

0	0	0	0	2	0	0	0	0	0	0	0	0
0	0	0	0	0	0	0	0					
"Viruses;Uroviricota;Caudoviricetes;Caudoviricetes__na;Caudoviricetes__na;Caudoviricetes__na;Myoviridae_s												
p._ctBCv9"	0	0	0	0	0	0	0	0	0	0	0	0
0	1	0	0	0	0	0	0	0	0	0	0	0
0	0	0	0	0	2	1	0	0	0	0	1	0
0	0	0	0	0	0	0	0	0	0	0	0	0
0	0	0	0	1	0	0	0	0	0	0	0	0
0	0	0	0	0	0	0	0					
"Viruses;Uroviricota;Caudoviricetes;Caudoviricetes__na;Caudoviricetes__na;Caudoviricetes__na;Myoviridae_s												
p._ctbEa13"	0	0	0	0	0	0	0	0	0	0	0	0
0	0	0	0	0	0	0	0	0	0	0	0	0
0	0	0	0	0	0	0	0	0	0	0	0	0
0	0	0	0	0	0	0	0	0	0	0	0	0
0	0	0	0	1	0	0	0	0	0	0	0	0
0	0	0	0	0	0	0	0					
"Viruses;Uroviricota;Caudoviricetes;Caudoviricetes__na;Caudoviricetes__na;Caudoviricetes__na;Myoviridae_s												
p._ctBtV12"	0	0	0	0	0	0	0	0	0	0	0	0
0	0	0	0	0	0	0	0	0	0	0	0	0
0	0	0	0	0	1	0	0	0	0	0	2	0
0	0	0	0	0	0	0	0	0	0	0	0	0
0	0	0	1	0	0	0	0	0	0	0	0	0
0	0	0	0	0	0	0	0					
"Viruses;Uroviricota;Caudoviricetes;Caudoviricetes__na;Caudoviricetes__na;Caudoviricetes__na;Myoviridae_s												
p._ctBvM24"	0	0	0	0	0	0	0	0	0	0	0	0
0	0	0	0	0	1	0	0	0	0	0	0	0
0	0	0	0	0	0	0	0	0	0	0	0	0
0	0	0	0	0	0	0	0	0	0	0	0	0
0	0	0	0	0	0	0	0	0	0	0	0	0
0	0	0	0	0	0	0	0					
"Viruses;Uroviricota;Caudoviricetes;Caudoviricetes__na;Caudoviricetes__na;Caudoviricetes__na;Myoviridae_s												
p._ctbwh6"	0	0	0	0	0	0	0	0	0	0	0	0
1	0	0	0	0	0	0	0	0	0	0	0	0
0	0	0	0	0	0	0	0	0	0	0	0	0
0	0	0	0	0	0	0	0	0	0	23	18	0
0	0	0	0	0	0	0	0	0	2	5	0	0
0	0	0	0	0	0	0	0					
"Viruses;Uroviricota;Caudoviricetes;Caudoviricetes__na;Caudoviricetes__na;Caudoviricetes__na;Myoviridae_s												
p._ctbWL16"	0	0	0	0	0	0	0	0	0	0	0	0
1	0	0	1	0	0	0	0	0	0	0	0	0
0	0	0	0	0	0	0	0	0	0	0	0	0
0	0	0	0	0	0	0	0	0	0	0	0	0
0	0	0	0	0	0	0	0	0	0	0	0	0
0	0	0	0	0	0	0	0					
"Viruses;Uroviricota;Caudoviricetes;Caudoviricetes__na;Caudoviricetes__na;Caudoviricetes__na;Myoviridae_s												
p._ctBZY1"	0	0	0	0	0	0	0	0	0	0	0	0
1	0	0	0	0	0	0	0	0	0	0	0	0
0	0	0	0	0	0	0	0	0	0	0	0	0
0	0	0	0	0	0	0	0	0	0	0	0	0

0 0 0 0 0 0 0 0 0 0 0 0 0
0 0 0 0 0 0 0 0
"Viruses;Uroviricota;Caudoviricetes;Caudoviricetes__na;Caudoviricetes__na;Caudoviricetes__na;Myoviridae_s
p._ctcaJ26" 0 0 0 0 0 0 0 0 0 0 0 0
0 1 0 0 0 0 3 5 0 0 1 0 0
2 0 0 0 0 1 1 0 0 0 0 1 0
0 0 0 0 0 0 0 0 0 0 12 24 0
0 0 0 2 0 0 0 0 0 15 18 0 0
0 0 1 5 0 0 0 0
"Viruses;Uroviricota;Caudoviricetes;Caudoviricetes__na;Caudoviricetes__na;Caudoviricetes__na;Myoviridae_s
p._ctcFb5" 0 0 0 0 0 0 0 0 0 0 0 0
0 0 0 0 0 0 0 0 0 0 0 0 0
0 0 0 0 1 0 0 0 0 0 0 0 0
0 0 0 0 0 0 0 0 0 0 0 0 0
0 0 0 0 0 0 0 0 0 0 2 0 0
0 0 0 0 0 0 0 0
"Viruses;Uroviricota;Caudoviricetes;Caudoviricetes__na;Caudoviricetes__na;Caudoviricetes__na;Myoviridae_s
p._ctCjb12" 0 0 0 0 0 0 0 0 0 0 0 0
0 0 0 0 0 0 4 3 0 0 0 0 0
0 0 0 0 0 0 0 0 0 0 0 2 7
0 0 0 0 0 0 0 0 0 0 0 0 0
0 0 0 0 0 0 0 0 0 0 0 0 0
0 0 0 5 0 0 0 0
"Viruses;Uroviricota;Caudoviricetes;Caudoviricetes__na;Caudoviricetes__na;Caudoviricetes__na;Myoviridae_s
p._ctCL221" 0 0 0 0 0 0 0 0 0 0 0 0
0 0 0 0 0 0 0 0 0 0 0 0 0
0 0 0 0 0 1 4 0 0 0 0 0 0
0 0 0 0 0 0 0 0 0 0 0 0 0
0 0 0 0 1 0 0 0 0 0 0 0 0
0 0 0 0 0 0 0 0
"Viruses;Uroviricota;Caudoviricetes;Caudoviricetes__na;Caudoviricetes__na;Caudoviricetes__na;Myoviridae_s
p._ctcPl3" 0 0 0 0 0 0 0 0 0 0 0 0
0 0 0 0 0 0 0 0 0 1 0 0 0
0 0 0 0 0 0 0 0 0 0 0 0 1
0 0 0 0 0 0 0 0 0 0 0 0 0
0 2 0 0 0 0 0 0 0 1 0 0 0
0 0 0 0 0 0 0 0
"Viruses;Uroviricota;Caudoviricetes;Caudoviricetes__na;Caudoviricetes__na;Caudoviricetes__na;Myoviridae_s
p._ctcwu24" 0 0 0 0 0 0 0 0 0 0 0 0
1 6 0 0 1 0 1 0 0 0 0 0 2
2 0 0 0 0 0 0 0 0 0 0 0 0
0 1 0 0 0 0 0 0 0 0 0 0 0
0 0 0 0 0 0 0 0 0 0 0 0 0
0 0 0 0 0 0 0 0
"Viruses;Uroviricota;Caudoviricetes;Caudoviricetes__na;Caudoviricetes__na;Caudoviricetes__na;Myoviridae_s
p._ctCXW4" 0 0 0 0 0 0 0 0 0 0 0 0
0 0 0 0 0 0 0 0 0 0 0 0 0
0 0 0 0 0 0 0 0 0 0 0 0 0
0 0 0 0 0 0 0 0 0 0 0 0 0

0 0 0 0 1 0 0 0 0 0 0 0 0
0 0 0 0 0 0 0 0
"Viruses;Uroviricota;Caudoviricetes;Caudoviricetes__na;Caudoviricetes__na;Caudoviricetes__na;Myoviridae_s
p._ctdWz11" 0 0 0 0 0 0 0 0 0 0 0 0
0 0 0 0 0 0 0 0 0 0 0 0 0
0 0 0 0 0 0 0 0 0 0 0 0 0
0 0 0 0 0 0 0 0 0 0 0 0 0
0 0 0 0 0 0 0 0 0 0 0 0 0
0 0 1 2 0 0 0 0
"Viruses;Uroviricota;Caudoviricetes;Caudoviricetes__na;Caudoviricetes__na;Caudoviricetes__na;Myoviridae_s
p._ctDzM5" 0 0 0 0 0 0 0 0 0 0 0 0
0 0 0 0 0 0 0 0 0 0 0 0 0
0 0 0 0 0 0 0 0 0 0 0 0 0
0 0 0 0 0 0 0 0 0 0 0 0 0
0 0 0 1 2 0 0 0 0 0 0 0 0
0 0 0 0 0 0 0 0
"Viruses;Uroviricota;Caudoviricetes;Caudoviricetes__na;Caudoviricetes__na;Caudoviricetes__na;Myoviridae_s
p._ctE3x18" 0 0 0 0 0 0 0 0 0 0 0 0
0 0 0 0 0 0 0 0 0 0 0 0 0
0 0 0 0 0 10 4 0 0 0 0 0 0
0 0 0 0 0 0 0 0 0 0 0 0 0
0 0 0 0 0 0 0 0 0 0 0 0 0
0 0 0 0 0 0 0 0
"Viruses;Uroviricota;Caudoviricetes;Caudoviricetes__na;Caudoviricetes__na;Caudoviricetes__na;Myoviridae_s
p._cte5Z19" 0 0 0 0 0 0 0 0 0 0 0 0
0 0 0 0 0 0 0 0 0 0 0 0 0
1 0 0 0 0 0 0 0 0 0 0 0 0
0 0 0 0 0 1 0 0 0 0 0 0 0
0 0 0 0 1 0 0 0 0 0 0 0 0
0 0 0 0 0 0 0 0
"Viruses;Uroviricota;Caudoviricetes;Caudoviricetes__na;Caudoviricetes__na;Caudoviricetes__na;Myoviridae_s
p._cteBs22" 0 0 0 0 0 0 0 0 0 0 0 0
0 0 0 0 0 0 0 0 0 0 0 0 1
0 0 0 0 0 3 3 0 0 2 0 0 0
0 0 0 0 0 0 0 0 0 0 0 0 0
0 0 0 0 0 0 0 0 0 18 1 0 1
0 0 0 0 0 0 0 0
"Viruses;Uroviricota;Caudoviricetes;Caudoviricetes__na;Caudoviricetes__na;Caudoviricetes__na;Myoviridae_s
p._cted82" 0 0 0 0 0 0 0 0 0 0 0 0
0 0 0 0 0 0 0 0 0 0 0 0 0
0 0 0 0 0 0 0 0 0 0 0 0 0
0 0 0 0 0 0 0 0 0 0 0 0 0
0 0 0 0 0 0 0 0 0 1 0 0 0
0 0 0 0 0 0 0 0
"Viruses;Uroviricota;Caudoviricetes;Caudoviricetes__na;Caudoviricetes__na;Caudoviricetes__na;Myoviridae_s
p._ctEg02" 0 0 0 0 0 0 0 0 0 0 0 0
0 0 0 0 0 0 0 0 0 0 0 0 0
0 0 0 0 0 0 0 0 0 0 0 0 0
0 0 0 0 0 0 0 0 0 0 0 0 0

0 0 0 1 2 0 0 0 0 0 0 0 0
0 0 0 1 0 0 0 0
"Viruses;Uroviricota;Caudoviricetes;Caudoviricetes__na;Caudoviricetes__na;Caudoviricetes__na;Myoviridae_sp._ctEtC12" 0 0 0 0 0 0 0 0 0 0 0 0
6 12 4 3 0 0 0 0 0 0 0 0 0
0 0 0 0 0 0 0 0 0 0 0 0 0
0 0 0 0 0 0 0 0 0 0 0 0 0
0 0 0 1 0 0 0 0 0 0 0 0 0
0 0 0 0 0 0 0 0
"Viruses;Uroviricota;Caudoviricetes;Caudoviricetes__na;Caudoviricetes__na;Caudoviricetes__na;Myoviridae_sp._ctEXz2" 0 0 0 0 0 0 0 0 0 0 0 0
2 5 0 0 0 0 0 0 0 0 0 0 0
0 0 0 0 0 0 0 0 0 0 0 0 0
0 0 0 0 0 0 0 0 0 0 0 0 0
0 0 0 0 0 0 0 0 0 0 0 0 0
0 0 0 0 0 0 0 0
"Viruses;Uroviricota;Caudoviricetes;Caudoviricetes__na;Caudoviricetes__na;Caudoviricetes__na;Myoviridae_sp._ctfA14" 0 0 0 0 0 0 0 0 0 0 0 0
0 0 0 0 0 0 0 0 0 0 0 0 5
20 22 9 18 0 0 0 0 0 0 0 0 0
0 0 1 0 0 0 2 0 0 0 0 0 0
0 0 0 0 0 0 0 0 0 486 107 6 6
0 0 0 0 0 0 0 0
"Viruses;Uroviricota;Caudoviricetes;Caudoviricetes__na;Caudoviricetes__na;Caudoviricetes__na;Myoviridae_sp._ctfJc17" 0 0 0 0 0 0 0 0 0 0 0 0
0 0 0 0 0 0 0 0 0 0 0 0 0
0 0 0 0 0 0 0 0 0 0 0 19 5
3 0 0 10 0 0 0 0 0 0 0 0 0
0 2 0 0 0 0 0 0 0 0 0 0 0
0 0 0 0 0 0 0 0
"Viruses;Uroviricota;Caudoviricetes;Caudoviricetes__na;Caudoviricetes__na;Caudoviricetes__na;Myoviridae_sp._ctfuG5" 0 0 0 0 0 0 0 0 0 0 0 0
0 0 0 0 0 0 0 0 0 0 0 0 0
0 0 0 0 0 0 0 0 0 0 0 0 0
0 0 0 8 0 0 0 0 0 0 0 0 0
0 0 0 0 0 0 0 0 0 0 0 0 0
0 0 0 0 0 0 0 0
"Viruses;Uroviricota;Caudoviricetes;Caudoviricetes__na;Caudoviricetes__na;Caudoviricetes__na;Myoviridae_sp._ctFYw8" 0 0 0 0 0 0 0 0 0 0 0 0
0 0 0 0 0 0 0 0 0 0 0 0 0
0 0 0 0 0 2 0 0 0 4 0 0 0
0 0 0 0 0 0 0 0 0 0 0 0 0
0 0 0 0 0 0 0 0 0 0 0 0 0
0 0 0 0 0 0 0 0
"Viruses;Uroviricota;Caudoviricetes;Caudoviricetes__na;Caudoviricetes__na;Caudoviricetes__na;Myoviridae_sp._ctg8M33" 0 0 0 0 0 0 0 0 0 0 0 0
0 0 0 0 0 0 1 0 0 0 0 0 3
6 0 0 0 0 0 0 0 0 0 0 0 0
0 0 0 0 0 0 0 0 0 0 0 0 0

0 0 0 0 0 0 0 0 0 0 0 0 0
0 0 1 0 0 0 0 0
"Viruses;Uroviricota;Caudoviricetes;Caudoviricetes__na;Caudoviricetes__na;Caudoviricetes__na;Myoviridae_s
p._ctGjZ5" 0 0 0 0 0 0 0 0 0 0 0 0
0 0 0 0 0 0 0 0 0 0 0 0 0
0 0 0 0 0 0 0 0 0 0 0 0 0
0 0 0 0 0 0 0 0 0 0 0 0 0
0 0 0 0 0 0 0 0 0 0 0 0 0
0 0 0 1 0 0 0 0
"Viruses;Uroviricota;Caudoviricetes;Caudoviricetes__na;Caudoviricetes__na;Caudoviricetes__na;Myoviridae_s
p._ctGk74" 0 0 0 0 0 0 0 0 0 0 0 0
2 9 0 0 0 0 0 0 0 0 0 0 0
0 0 0 0 0 0 0 0 0 0 0 0 0
0 0 0 0 0 0 0 0 0 0 0 0 0
0 0 0 0 0 0 0 0 0 0 0 0 0
0 0 0 0 0 0 0 0
"Viruses;Uroviricota;Caudoviricetes;Caudoviricetes__na;Caudoviricetes__na;Caudoviricetes__na;Myoviridae_s
p._ctgpD8" 0 0 0 0 0 0 0 0 0 0 0 0
0 0 0 0 0 0 0 0 0 0 0 0 0
0 0 0 0 0 0 0 0 0 0 0 3 6
0 0 0 0 0 0 0 0 0 0 0 1 0
0 0 0 0 0 0 0 0 0 0 0 0 0
0 0 0 0 0 0 0 0
"Viruses;Uroviricota;Caudoviricetes;Caudoviricetes__na;Caudoviricetes__na;Caudoviricetes__na;Myoviridae_s
p._ctGRa7" 0 0 0 0 0 0 0 0 0 0 0 0
0 0 0 0 0 0 0 0 0 0 0 0 0
0 0 0 0 0 0 0 0 0 0 0 0 0
0 0 0 0 0 0 0 0 0 0 2 1 0
0 0 0 1 0 0 0 0 0 0 0 0 0
0 0 0 0 0 0 0 0
"Viruses;Uroviricota;Caudoviricetes;Caudoviricetes__na;Caudoviricetes__na;Caudoviricetes__na;Myoviridae_s
p._ctgXa1" 0 0 0 0 0 0 0 0 0 0 0 0
0 0 0 0 0 0 0 0 0 0 0 0 0
0 0 0 0 0 0 0 0 0 0 0 1 0
0 0 0 0 0 0 0 0 0 0 0 0 0
0 0 0 0 0 0 0 0 0 0 1 0 0
0 0 1 0 0 0 0 0
"Viruses;Uroviricota;Caudoviricetes;Caudoviricetes__na;Caudoviricetes__na;Caudoviricetes__na;Myoviridae_s
p._ctgXL3" 0 0 0 0 0 0 0 0 0 0 0 0
11 17 26 10 50 13 0 0 0 0 1 0 0
0 0 0 0 0 0 0 0 0 0 0 0 0
0 0 0 0 0 0 0 0 0 0 0 0 0
0 0 0 0 0 0 0 0 0 0 0 0 0
0 0 0 0 0 0 0 0
"Viruses;Uroviricota;Caudoviricetes;Caudoviricetes__na;Caudoviricetes__na;Caudoviricetes__na;Myoviridae_s
p._ctHaT25" 0 0 0 0 0 0 0 0 0 0 0 0
5 0 2 0 3 0 0 0 0 0 0 0 0
0 0 0 0 0 0 0 0 0 0 0 0 0
0 0 0 0 0 0 0 0 0 0 0 0 0

0 0 0 0 0 0 0 0 0 0 0 0 0
0 0 0 0 0 0 0 0
"Viruses;Uroviricota;Caudoviricetes;Caudoviricetes__na;Caudoviricetes__na;Caudoviricetes__na;Myoviridae_s
p._ctHIt1" 0 0 0 0 0 0 0 0 0 0 0 0
0 0 1 0 0 0 0 0 0 0 0 0 0
0 0 0 0 0 0 0 0 0 0 0 0 0
0 0 0 0 0 0 0 0 0 0 0 0 1
0 0 0 0 0 0 0 0 0 0 0 0 0
0 1 0 0 0 0 0 0
"Viruses;Uroviricota;Caudoviricetes;Caudoviricetes__na;Caudoviricetes__na;Caudoviricetes__na;Myoviridae_s
p._ctHlk26" 0 0 0 0 0 0 0 0 0 0 0 0
0 0 0 0 0 0 0 0 0 0 0 0 0
0 0 0 0 0 0 0 0 0 0 0 0 0
0 0 0 0 0 0 0 0 0 0 0 0 0
0 0 0 0 1 0 0 0 0 0 0 0 0
0 0 0 0 0 0 0 0
"Viruses;Uroviricota;Caudoviricetes;Caudoviricetes__na;Caudoviricetes__na;Caudoviricetes__na;Myoviridae_s
p._ctHMa1" 0 0 0 0 0 0 0 0 0 0 0 0
0 0 0 0 0 0 3 14 0 0 0 0 6
5 0 0 0 0 0 1 0 0 0 0 6 3
0 0 0 0 5 12 0 0 0 0 1 3 0
0 0 0 2 0 0 0 0 0 3 1 0 1
0 0 0 3 0 0 0 0
"Viruses;Uroviricota;Caudoviricetes;Caudoviricetes__na;Caudoviricetes__na;Caudoviricetes__na;Myoviridae_s
p._cthmz15" 0 0 0 0 0 0 0 0 0 0 0 0
0 0 0 0 0 0 0 0 0 0 0 0 0
0 0 0 0 0 0 0 0 0 0 0 0 0
0 0 0 0 0 0 0 0 0 0 0 0 0
0 0 0 2 1 0 0 0 0 0 0 0 0
0 0 0 0 0 0 0 0
"Viruses;Uroviricota;Caudoviricetes;Caudoviricetes__na;Caudoviricetes__na;Caudoviricetes__na;Myoviridae_s
p._ctHP32" 0 0 0 0 0 0 0 0 0 0 0 0
0 0 0 1 0 0 1 0 0 0 0 0 0
0 0 0 0 0 0 0 0 0 0 0 0 0
0 0 0 0 0 0 0 0 0 0 0 0 0
0 0 0 0 0 0 0 0 0 2 0 0 0
0 0 0 0 0 0 0 0
"Viruses;Uroviricota;Caudoviricetes;Caudoviricetes__na;Caudoviricetes__na;Caudoviricetes__na;Myoviridae_s
p._cthRr4" 0 0 0 0 0 0 0 0 0 0 0 0
7 9 0 2 2 1 0 0 0 0 0 0 0
0 0 0 0 0 0 0 0 0 0 0 0 0
0 0 0 0 0 0 0 0 0 0 0 0 0
0 0 0 0 0 0 0 0 0 0 0 0 0
0 0 0 0 0 0 0 0
"Viruses;Uroviricota;Caudoviricetes;Caudoviricetes__na;Caudoviricetes__na;Caudoviricetes__na;Myoviridae_s
p._ctI7W9" 0 0 0 0 0 0 0 0 0 0 0 0
0 3 0 0 0 0 0 0 0 0 0 0 0
0 0 0 0 0 0 0 0 0 0 0 0 0
0 0 0 0 0 0 0 0 0 0 0 0 0

0 0 0 0 1 0 0 0 0 0 0 0 0
0 0 0 0 0 0 0 0
"Viruses;Uroviricota;Caudoviricetes;Caudoviricetes__na;Caudoviricetes__na;Caudoviricetes__na;Myoviridae_s
p._ctiIS8" 0 0 0 0 0 0 0 0 0 0 0 0
1 0 0 1 0 15 0 0 0 0 0 0 0
0 0 0 0 0 0 0 0 0 0 0 0 1
0 0 0 0 0 0 0 0 0 0 0 0 0
0 0 0 0 0 0 0 0 0 0 0 0 0
0 0 0 1 0 0 0 0
"Viruses;Uroviricota;Caudoviricetes;Caudoviricetes__na;Caudoviricetes__na;Caudoviricetes__na;Myoviridae_s
p._ctISH16" 0 0 0 0 0 0 0 0 0 0 0 0
0 0 0 0 0 0 0 0 0 0 0 0 0
0 0 0 0 0 0 0 0 0 0 0 0 0
0 0 0 0 0 0 0 0 0 0 0 0 0
0 0 0 2 0 0 0 0 0 0 0 0 0
0 0 0 0 0 0 0 0
"Viruses;Uroviricota;Caudoviricetes;Caudoviricetes__na;Caudoviricetes__na;Caudoviricetes__na;Myoviridae_s
p._ctIty1" 0 0 0 0 0 0 0 0 0 3 0 0
0 0 0 0 0 0 0 0 0 0 0 0 0
0 0 0 0 0 0 0 0 0 0 0 0 0
0 0 0 0 0 0 0 0 0 0 0 0 0
0 0 0 0 0 0 0 0 0 0 0 0 0
0 0 0 0 0 0 0 0
"Viruses;Uroviricota;Caudoviricetes;Caudoviricetes__na;Caudoviricetes__na;Caudoviricetes__na;Myoviridae_s
p._ctj994" 0 0 0 0 0 0 0 0 0 0 0 0
0 0 0 0 0 0 0 0 0 0 0 0 0
0 0 0 0 0 0 0 0 0 0 1 0 0
0 0 0 0 0 0 0 0 0 0 0 0 0
0 0 0 0 0 0 0 0 0 0 0 0 0
0 0 0 0 0 0 0 0
"Viruses;Uroviricota;Caudoviricetes;Caudoviricetes__na;Caudoviricetes__na;Caudoviricetes__na;Myoviridae_s
p._ctjH82" 0 0 0 0 0 0 0 0 0 0 0 0
0 0 0 0 0 0 0 1 0 0 0 0 0
0 0 0 0 0 0 0 0 0 0 0 0 0
0 0 0 0 0 0 0 0 0 0 0 0 0
0 0 4 1 1 0 0 0 0 0 0 0 0
0 0 3 7 0 0 0 0
"Viruses;Uroviricota;Caudoviricetes;Caudoviricetes__na;Caudoviricetes__na;Caudoviricetes__na;Myoviridae_s
p._ctK7P4" 0 0 0 0 0 0 0 0 0 0 0 0
0 0 0 0 0 0 0 0 0 0 0 0 0
0 0 0 0 0 0 0 0 0 0 0 0 0
0 0 0 0 0 0 0 0 0 0 0 0 0
0 0 0 2 10 0 0 0 0 0 0 0 0
0 0 0 0 0 0 0 0
"Viruses;Uroviricota;Caudoviricetes;Caudoviricetes__na;Caudoviricetes__na;Caudoviricetes__na;Myoviridae_s
p._ctkfK18" 0 0 0 0 0 0 0 0 0 2 0 0
0 0 0 0 0 0 0 0 0 0 0 0 0
0 0 0 0 0 0 0 0 0 0 1 0 0
0 0 0 16 0 0 0 0 0 0 0 0 0

0 0 0 0 0 0 0 0 0 0 0 0 0
0 0 0 0 0 0 0 0
"Viruses;Uroviricota;Caudoviricetes;Caudoviricetes__na;Caudoviricetes__na;Caudoviricetes__na;Myoviridae_s
p._ctKkB1" 0 0 0 0 0 0 0 0 0 0 0 0
0 0 0 0 0 0 0 0 0 0 0 0 0
0 0 0 0 0 0 0 0 0 0 0 0 0
0 0 0 0 0 0 0 0 0 0 0 0 0
0 0 0 1 1 0 0 0 0 0 0 0 0
0 0 0 0 0 0 0 0
"Viruses;Uroviricota;Caudoviricetes;Caudoviricetes__na;Caudoviricetes__na;Caudoviricetes__na;Myoviridae_s
p._ctkOm7" 0 0 0 0 0 0 0 0 0 0 0 0
2 0 0 0 0 0 1 2 0 0 0 0 0
0 0 0 0 0 4 6 0 0 1 0 3 4
0 0 0 0 0 0 0 0 1 0 3 0 1
0 0 15 3 4 1 0 0 0 2 0 0 0
0 0 5 4 0 0 0 0
"Viruses;Uroviricota;Caudoviricetes;Caudoviricetes__na;Caudoviricetes__na;Caudoviricetes__na;Myoviridae_s
p._ctKZW4" 0 0 0 0 0 0 0 0 0 0 0 0
0 0 0 0 0 0 0 0 0 0 0 0 0
0 0 0 0 0 0 0 0 0 0 0 0 0
0 0 0 10 0 0 0 0 0 0 0 0 0
0 0 0 0 0 0 0 0 0 0 0 0 0
0 0 0 0 0 0 0 0
"Viruses;Uroviricota;Caudoviricetes;Caudoviricetes__na;Caudoviricetes__na;Caudoviricetes__na;Myoviridae_s
p._ctlD98" 0 0 0 0 0 0 0 0 0 0 0 0
0 0 0 0 0 0 0 1 0 0 0 0 0
0 0 0 0 0 0 0 0 0 0 0 0 0
0 0 0 0 0 1 0 0 0 0 0 1 0
0 0 0 0 0 0 0 0 0 0 0 0 0
0 0 0 0 0 0 0 0
"Viruses;Uroviricota;Caudoviricetes;Caudoviricetes__na;Caudoviricetes__na;Caudoviricetes__na;Myoviridae_s
p._ctLGX4" 0 0 0 0 0 0 0 0 0 0 0 0
1 0 0 0 0 0 0 0 0 0 0 0 0
0 0 0 0 0 0 0 0 0 0 0 0 0
0 0 0 0 0 0 0 0 0 0 0 0 0
0 0 0 0 0 0 0 0 0 0 0 0 0
0 0 0 0 0 0 0 0
"Viruses;Uroviricota;Caudoviricetes;Caudoviricetes__na;Caudoviricetes__na;Caudoviricetes__na;Myoviridae_s
p._ctLjW1" 0 0 0 0 0 0 0 0 0 0 0 0
0 0 0 0 0 0 0 0 0 0 0 0 0
0 0 0 0 0 0 0 0 0 0 0 0 0
0 0 0 0 0 1 0 0 0 0 0 0 0
0 0 0 0 0 0 0 0 0 0 0 0 0
0 0 0 0 0 0 0 0
"Viruses;Uroviricota;Caudoviricetes;Caudoviricetes__na;Caudoviricetes__na;Caudoviricetes__na;Myoviridae_s
p._ctlnK45" 0 0 0 0 0 0 0 0 0 0 0 0
0 0 0 0 0 0 0 0 0 0 0 0 0
0 0 0 0 0 0 0 0 0 0 0 1 0
0 0 0 0 0 0 0 0 0 0 0 0 0

0 0 0 0 1 0 0 0 0 0 0 0 0
0 0 0 0 0 0 0 0
"Viruses;Uroviricota;Caudoviricetes;Caudoviricetes__na;Caudoviricetes__na;Caudoviricetes__na;Myoviridae_sp._ctLtm40" 0 0 0 0 0 0 0 0 0 0 0 0
0 0 0 0 0 0 0 0 0 0 0 0 1
0 0 0 0 0 0 0 0 0 0 0 1 0
0 0 0 0 0 0 0 0 0 0 0 0 0
0 0 0 2 0 0 0 0 0 0 0 0 0
0 0 0 0 0 0 0 0
"Viruses;Uroviricota;Caudoviricetes;Caudoviricetes__na;Caudoviricetes__na;Caudoviricetes__na;Myoviridae_sp._ctLx49" 0 0 0 0 0 0 0 0 0 0 0 0
3 7 1 0 0 0 0 0 0 0 0 0 0
0 0 0 0 0 0 2 0 0 0 0 0 0
0 0 0 0 0 0 0 0 0 0 0 0 0
0 0 0 0 0 0 0 0 0 0 0 0 0
0 0 0 0 0 0 0 0
"Viruses;Uroviricota;Caudoviricetes;Caudoviricetes__na;Caudoviricetes__na;Caudoviricetes__na;Myoviridae_sp._ctm8X17" 0 0 0 0 0 0 0 0 0 0 0 0
4 2 0 0 1 0 2 4 1 1 4 0 0
1 0 0 0 0 2 0 0 0 0 0 2 3
0 0 0 0 0 1 0 0 0 0 12 16 0
1 0 0 2 3 0 0 0 0 0 0 0 0
0 0 0 1 0 0 1 0
"Viruses;Uroviricota;Caudoviricetes;Caudoviricetes__na;Caudoviricetes__na;Caudoviricetes__na;Myoviridae_sp._ctMTY370" 0 0 0 0 0 0 0 0 0 0 0 0
0 0 0 0 0 0 0 1 0 1 0 0 3
5 13 10 10 0 0 0 0 0 0 0 0 0
0 0 1 0 2 3 38 7 0 0 0 0 0
0 0 0 0 0 0 0 0 0 0 0 0 0
0 0 0 0 0 0 0 0
"Viruses;Uroviricota;Caudoviricetes;Caudoviricetes__na;Caudoviricetes__na;Caudoviricetes__na;Myoviridae_sp._ctMvU7" 0 0 0 0 0 0 0 0 0 0 0 0
0 0 0 0 0 0 0 0 0 0 0 0 0
0 0 0 0 0 1 0 0 0 0 0 0 0
0 0 0 0 0 0 0 0 0 0 0 0 0
0 0 0 0 0 0 0 0 0 0 0 0 0
0 0 0 3 0 0 0 0
"Viruses;Uroviricota;Caudoviricetes;Caudoviricetes__na;Caudoviricetes__na;Caudoviricetes__na;Myoviridae_sp._ctncN39" 0 0 0 0 0 0 0 0 0 0 0 0
0 0 0 0 0 0 0 0 0 0 0 0 0
0 0 0 0 0 0 0 0 0 0 0 0 0
0 0 0 0 0 0 0 0 0 0 0 0 0
0 0 0 0 0 0 0 0 0 1 0 0 0
0 0 0 0 0 0 0 0
"Viruses;Uroviricota;Caudoviricetes;Caudoviricetes__na;Caudoviricetes__na;Caudoviricetes__na;Myoviridae_sp._ctNDZ29" 0 0 0 0 0 0 0 0 0 0 0 0
0 0 0 0 0 0 0 0 0 0 0 0 0
0 0 0 0 0 0 0 0 0 0 0 2 0
0 0 0 0 0 0 0 0 0 0 0 0 0

0 0 0 0 0 0 0 0 0 0 0 0 0
0 0 0 0 0 0 0 0
"Viruses;Uroviricota;Caudoviricetes;Caudoviricetes__na;Caudoviricetes__na;Caudoviricetes__na;Myoviridae_s
p._ctngn1" 0 0 0 0 0 0 0 0 0 0 0 0
0 0 0 0 0 0 0 0 0 0 0 0 2
0 0 0 0 0 0 1 0 0 0 0 0 0
0 0 0 0 0 0 0 0 0 0 1 0 0
0 0 0 0 0 0 0 0 0 0 0 0 0
0 0 0 0 0 0 0 0
"Viruses;Uroviricota;Caudoviricetes;Caudoviricetes__na;Caudoviricetes__na;Caudoviricetes__na;Myoviridae_s
p._ctNhr24" 0 0 0 0 0 0 0 0 0 0 0 0
1 1 0 1 0 0 0 0 0 0 0 0 0
0 0 0 0 0 4 2 0 0 0 0 0 0
0 0 0 0 0 0 0 0 0 0 0 0 0
0 0 0 0 2 0 0 0 0 0 0 0 0
0 0 0 0 0 0 0 0
"Viruses;Uroviricota;Caudoviricetes;Caudoviricetes__na;Caudoviricetes__na;Caudoviricetes__na;Myoviridae_s
p._ctniE2" 0 0 0 0 0 0 0 0 0 0 0 0
0 0 0 0 0 0 0 0 0 0 0 0 0
0 0 0 0 0 0 0 0 0 0 0 0 0
0 0 0 0 0 0 0 0 0 0 1 0 0
0 0 0 0 0 0 0 0 0 0 0 0 0
0 0 0 0 0 0 0 0
"Viruses;Uroviricota;Caudoviricetes;Caudoviricetes__na;Caudoviricetes__na;Caudoviricetes__na;Myoviridae_s
p._ctNqw6" 0 0 0 0 0 0 0 0 0 0 0 0
0 0 0 0 0 0 0 0 0 0 0 0 0
0 0 0 0 0 0 0 0 0 0 0 0 0
0 0 0 0 0 0 0 0 0 0 0 0 0
0 0 0 1 2 0 0 0 0 2 0 0 0
0 0 0 0 0 0 0 0
"Viruses;Uroviricota;Caudoviricetes;Caudoviricetes__na;Caudoviricetes__na;Caudoviricetes__na;Myoviridae_s
p._ctnzH2" 0 0 0 0 0 0 0 0 0 0 0 0
0 0 0 0 0 0 0 0 0 0 0 0 0
0 0 0 0 0 4 4 0 0 0 0 0 0
0 0 0 0 0 0 0 0 0 0 0 0 0
0 0 2 2 0 0 0 0 0 0 0 0 0
0 0 0 0 0 0 0 0
"Viruses;Uroviricota;Caudoviricetes;Caudoviricetes__na;Caudoviricetes__na;Caudoviricetes__na;Myoviridae_s
p._ctO7l1" 0 0 0 0 0 0 0 0 0 0 0 0
1 0 0 0 0 0 0 1 0 0 0 0 0
0 0 0 0 0 0 0 0 0 0 0 0 0
0 0 0 0 0 0 0 0 0 0 0 0 0
0 0 0 0 0 0 0 0 0 0 0 0 0
0 0 0 0 0 0 0 0
"Viruses;Uroviricota;Caudoviricetes;Caudoviricetes__na;Caudoviricetes__na;Caudoviricetes__na;Myoviridae_s
p._ctoIO8" 0 0 0 0 0 0 0 0 0 0 0 0
0 0 0 0 0 0 0 0 0 0 0 0 13
16 0 0 0 0 2 1 0 0 0 0 1 2
0 0 0 0 0 0 0 0 0 0 0 0 0

0 0 0 1 0 0 0 0 0 4 4 0 0
0 0 3 8 0 0 0 0
"Viruses;Uroviricota;Caudoviricetes;Caudoviricetes__na;Caudoviricetes__na;Caudoviricetes__na;Myoviridae_s
p._ctool15" 0 0 0 0 0 0 0 0 0 0 0 0
0 0 0 0 0 0 0 0 0 0 0 0 0
0 0 0 0 0 0 0 0 0 0 0 0 0
0 0 0 0 0 0 0 0 0 0 0 0 0
0 0 0 0 1 0 0 0 0 0 0 0 0
0 0 0 0 0 0 0 0
"Viruses;Uroviricota;Caudoviricetes;Caudoviricetes__na;Caudoviricetes__na;Caudoviricetes__na;Myoviridae_s
p._ctOpw2" 0 0 0 0 0 0 0 0 0 0 0 0
0 0 0 0 0 0 3 8 0 0 0 0 16
9 2 0 2 0 0 0 0 0 0 0 0 0
0 0 0 0 3 2 4 0 0 0 0 0 0
0 0 3 0 0 0 0 0 0 0 0 0 0
0 0 5 3 0 0 0 0
"Viruses;Uroviricota;Caudoviricetes;Caudoviricetes__na;Caudoviricetes__na;Caudoviricetes__na;Myoviridae_s
p._ctOv05" 0 0 0 0 0 0 0 0 0 0 0 0
0 1 0 0 0 3 0 0 0 0 0 0 0
0 0 0 0 0 0 0 0 0 0 0 0 0
0 0 0 0 0 0 0 0 0 0 0 0 0
0 0 0 0 0 0 0 0 0 0 0 0 0
0 0 0 0 0 0 0 0
"Viruses;Uroviricota;Caudoviricetes;Caudoviricetes__na;Caudoviricetes__na;Caudoviricetes__na;Myoviridae_s
p._ctOyc4" 0 0 0 0 0 0 0 0 0 0 0 0
0 0 0 0 0 0 0 0 0 0 0 0 0
0 0 0 0 0 0 0 0 0 0 0 0 0
0 0 0 0 0 0 0 0 0 0 1 0 0
0 0 0 0 0 0 0 0 0 0 0 0 0
0 0 0 0 0 0 0 0
"Viruses;Uroviricota;Caudoviricetes;Caudoviricetes__na;Caudoviricetes__na;Caudoviricetes__na;Myoviridae_s
p._ctP4M4" 0 0 0 0 0 0 0 0 0 0 0 0
0 2 2 4 7 47 1 2 0 0 0 0 0
0 0 0 0 0 0 0 0 0 0 0 0 0
0 0 0 0 1 0 0 0 0 0 0 1 0
0 0 0 0 0 0 0 0 0 1 0 0 0
0 0 1 3 0 0 0 0
"Viruses;Uroviricota;Caudoviricetes;Caudoviricetes__na;Caudoviricetes__na;Caudoviricetes__na;Myoviridae_s
p._ctp4Q36" 0 0 0 1 0 0 0 0 0 0 0 0
0 0 0 0 0 0 0 0 0 0 0 0 0
0 0 0 0 0 0 0 0 0 0 0 0 0
0 0 0 0 0 0 0 0 0 0 0 0 0
0 0 0 0 0 0 0 0 0 0 0 0 0
0 0 0 0 0 0 0 0
"Viruses;Uroviricota;Caudoviricetes;Caudoviricetes__na;Caudoviricetes__na;Caudoviricetes__na;Myoviridae_s
p._ctP6q2" 0 0 0 0 0 0 0 0 0 0 0 0
0 0 0 0 1 3 0 1 1 0 0 0 0
0 0 0 0 0 0 0 0 0 0 0 0 0
0 0 0 0 0 0 0 0 0 0 0 0 0

0 0 0 0 1 0 0 0 0 0 0 0 0
0 0 0 0 0 0 0 0
"Viruses;Uroviricota;Caudoviricetes;Caudoviricetes__na;Caudoviricetes__na;Caudoviricetes__na;Myoviridae_sp._ctp7F23" 0 0 0 0 0 0 0 0 0 0 0 0
0 0 0 0 0 0 0 1 0 0 0 0 0
0 0 0 0 0 0 0 0 0 0 0 0 0
0 0 0 0 0 0 0 0 0 0 0 0 0
0 0 104 0 0 0 0 0 0 0 0 0 0
0 0 7 6 1 2 0 0
"Viruses;Uroviricota;Caudoviricetes;Caudoviricetes__na;Caudoviricetes__na;Caudoviricetes__na;Myoviridae_sp._ctPGO22" 0 0 0 0 0 0 0 0 0 0 0 0
0 0 0 0 0 0 0 0 0 0 0 0 0
0 0 0 0 0 0 0 0 0 0 0 0 0
0 0 0 0 0 0 0 0 0 0 0 0 0
0 0 0 0 0 0 0 0 0 0 1 0 0
0 0 1 0 0 0 0 0
"Viruses;Uroviricota;Caudoviricetes;Caudoviricetes__na;Caudoviricetes__na;Caudoviricetes__na;Myoviridae_sp._ctplG2" 0 0 0 0 0 0 0 0 0 0 0 0
0 0 0 1 0 12 0 0 0 0 0 0 1
0 18 2 13 0 0 0 0 0 0 0 0 0
0 0 0 0 0 0 0 0 0 0 0 0 0
0 0 0 0 0 0 0 0 0 0 0 0 0
0 0 0 0 0 0 0 0
"Viruses;Uroviricota;Caudoviricetes;Caudoviricetes__na;Caudoviricetes__na;Caudoviricetes__na;Myoviridae_sp._ctPoO4" 0 0 0 0 0 0 0 0 0 0 0 0
2 0 0 0 0 0 0 0 0 0 0 1 1
0 0 0 0 0 0 0 0 0 0 0 0 1
0 0 0 0 0 0 0 0 0 0 0 1 0
0 0 0 0 0 0 0 0 0 0 0 0 0
0 0 0 0 0 0 0 0
"Viruses;Uroviricota;Caudoviricetes;Caudoviricetes__na;Caudoviricetes__na;Caudoviricetes__na;Myoviridae_sp._ctPVE25" 0 0 0 0 0 0 0 0 0 0 0 0
0 0 0 0 0 0 0 0 0 0 0 0 0
0 0 0 0 0 0 0 0 0 0 0 0 0
0 0 0 0 0 0 0 0 0 0 2 0 0
0 0 0 0 0 0 0 0 0 0 0 0 0
0 0 0 0 0 0 0 0
"Viruses;Uroviricota;Caudoviricetes;Caudoviricetes__na;Caudoviricetes__na;Caudoviricetes__na;Myoviridae_sp._ctQ6D10" 0 0 0 0 0 0 0 0 0 0 0 0
0 0 0 0 0 0 0 0 0 0 0 0 4
2 0 0 1 0 0 0 0 0 0 0 1 2
0 0 0 3 0 0 0 0 0 0 0 0 0
0 0 0 0 1 0 0 0 0 0 0 0 0
0 0 0 0 0 0 0 0
"Viruses;Uroviricota;Caudoviricetes;Caudoviricetes__na;Caudoviricetes__na;Caudoviricetes__na;Myoviridae_sp._ctq8k5" 0 0 0 0 0 0 0 0 0 0 0 0
0 0 0 0 0 1 0 0 0 0 0 0 1
1 0 0 0 0 0 0 0 0 0 0 3 3
0 0 0 0 0 0 0 0 0 0 0 0 0

0 0 0 0 0 0 0 0 0 0 0 0 0
0 0 0 0 0 0 0 0
"Viruses;Uroviricota;Caudoviricetes;Caudoviricetes__na;Caudoviricetes__na;Caudoviricetes__na;Myoviridae_s
p._ctq9w2" 0 0 0 0 0 0 0 0 0 0 0 0
0 0 0 0 1 0 0 0 0 0 0 0 0
0 0 0 0 0 0 0 0 0 0 0 1 0
0 0 0 0 0 0 0 0 0 0 0 0 0
0 0 0 0 0 0 0 0 0 0 1 0 0
0 0 0 1 0 0 0 0
"Viruses;Uroviricota;Caudoviricetes;Caudoviricetes__na;Caudoviricetes__na;Caudoviricetes__na;Myoviridae_s
p._ctQf419" 0 0 0 0 0 0 0 0 0 0 0 0
1 3 0 0 0 0 0 1 0 0 0 0 0
0 0 0 0 0 0 0 0 0 0 0 0 0
0 0 0 0 3 0 0 0 0 0 3 2 0
0 0 0 0 0 0 0 0 0 85 23 0 0
0 0 6 2 0 0 0 0
"Viruses;Uroviricota;Caudoviricetes;Caudoviricetes__na;Caudoviricetes__na;Caudoviricetes__na;Myoviridae_s
p._ctqfO1" 0 0 0 0 0 0 0 0 0 0 0 0
0 0 0 0 0 0 0 0 0 0 0 0 0
0 0 0 0 0 0 0 0 0 0 0 0 0
0 0 0 0 0 0 0 0 0 0 0 0 0
0 0 0 0 0 0 0 0 0 0 0 0 0
0 0 1 0 0 0 0 0
"Viruses;Uroviricota;Caudoviricetes;Caudoviricetes__na;Caudoviricetes__na;Caudoviricetes__na;Myoviridae_s
p._ctqgO2" 0 0 0 0 0 0 0 0 0 0 0 0
0 0 0 0 0 0 0 0 0 0 0 0 0
0 0 0 0 0 0 0 0 0 0 0 0 0
0 0 0 0 0 0 0 0 0 0 0 0 0
0 0 0 0 0 0 0 0 0 0 0 0 0
0 0 1 0 0 0 0 0
"Viruses;Uroviricota;Caudoviricetes;Caudoviricetes__na;Caudoviricetes__na;Caudoviricetes__na;Myoviridae_s
p._ctQQg4" 0 0 0 0 0 0 0 0 0 0 0 0
0 0 0 0 0 0 0 0 0 0 0 0 0
0 0 0 0 0 0 0 0 0 0 0 0 0
0 0 0 2 0 0 0 0 8 0 0 0 0
0 0 0 0 0 0 0 0 0 0 0 0 0
0 0 0 0 0 0 0 0
"Viruses;Uroviricota;Caudoviricetes;Caudoviricetes__na;Caudoviricetes__na;Caudoviricetes__na;Myoviridae_s
p._ctQYc56" 0 0 0 0 0 0 0 0 0 0 0 0
0 0 0 1 0 0 0 0 0 0 0 0 0
0 0 0 0 0 0 0 0 0 0 0 2 1
0 0 0 0 0 0 0 0 0 0 0 0 0
0 0 0 0 0 0 0 0 0 0 0 0 0
0 0 0 0 0 0 0 0
"Viruses;Uroviricota;Caudoviricetes;Caudoviricetes__na;Caudoviricetes__na;Caudoviricetes__na;Myoviridae_s
p._ctr0w28" 0 0 0 0 0 0 0 0 0 0 0 0
0 0 7 3 4 28 0 0 0 0 0 0 0
0 0 0 0 0 0 0 0 0 0 0 0 0
0 0 0 0 5 2 12 0 0 0 0 1 0

0 0 0 0 0 0 0 0 0 0 0 0 0
0 0 0 0 0 0 0 0
"Viruses;Uroviricota;Caudoviricetes;Caudoviricetes__na;Caudoviricetes__na;Caudoviricetes__na;Myoviridae_sp._ctrCp2" 0 0 1 0 0 0 0 0 0 0 0 0
1 2 0 0 0 0 0 1 1 0 0 1 2
2 0 0 0 0 0 0 0 0 0 0 1 1
0 0 0 0 2 4 0 0 0 0 0 0 0
0 0 0 0 0 0 0 0 0 0 0 0 0
0 0 1 2 0 0 0 0
"Viruses;Uroviricota;Caudoviricetes;Caudoviricetes__na;Caudoviricetes__na;Caudoviricetes__na;Myoviridae_sp._ctrEx11" 0 0 0 0 0 0 0 0 0 0 0 0
10 45 13 15 2 11 0 0 0 0 0 0 0
0 0 0 0 0 0 0 0 0 0 0 0 0
0 0 0 0 0 0 0 0 0 0 0 0 0
0 0 0 0 0 0 0 0 0 0 0 0 0
0 0 0 0 0 0 0 0
"Viruses;Uroviricota;Caudoviricetes;Caudoviricetes__na;Caudoviricetes__na;Caudoviricetes__na;Myoviridae_sp._ctRrG7" 0 0 0 0 0 0 0 0 0 0 0 0
0 0 0 0 0 0 0 0 0 0 0 0 0
0 0 0 0 0 0 1 0 0 0 0 0 0
0 0 0 0 0 0 0 0 0 0 0 0 0
0 0 0 2 1 0 0 0 0 0 0 0 0
0 0 0 0 0 0 0 0
"Viruses;Uroviricota;Caudoviricetes;Caudoviricetes__na;Caudoviricetes__na;Caudoviricetes__na;Myoviridae_sp._ctSBU9" 0 0 0 0 0 0 0 0 0 0 0 0
7 9 0 0 0 0 0 0 0 0 0 0 0
0 0 0 0 0 0 0 0 0 0 0 0 0
0 0 0 0 0 0 0 0 0 0 0 0 0
0 0 0 0 0 0 0 0 0 1 1 0 0
0 0 4 4 0 0 0 0
"Viruses;Uroviricota;Caudoviricetes;Caudoviricetes__na;Caudoviricetes__na;Caudoviricetes__na;Myoviridae_sp._ctsGI2" 0 0 0 0 0 0 0 0 0 0 0 0
0 0 0 0 0 0 0 0 0 0 0 0 0
0 0 0 0 0 0 0 0 0 0 0 0 0
0 0 0 0 0 0 0 0 0 0 0 0 0
0 0 0 0 0 0 0 0 0 0 0 0 0
0 0 0 1 0 0 0 0
"Viruses;Uroviricota;Caudoviricetes;Caudoviricetes__na;Caudoviricetes__na;Caudoviricetes__na;Myoviridae_sp._ctshb19" 0 0 0 0 0 1 0 0 0 0 0 0
0 0 0 0 0 0 0 0 0 0 0 0 0
0 0 0 0 0 0 0 0 0 0 0 0 0
0 0 0 0 0 0 0 0 0 0 0 0 0
0 0 0 0 0 0 0 0 0 0 0 0 0
0 0 0 0 0 0 0 0
"Viruses;Uroviricota;Caudoviricetes;Caudoviricetes__na;Caudoviricetes__na;Caudoviricetes__na;Myoviridae_sp._ctsIb3" 0 0 0 0 0 0 0 0 0 0 0 0
0 0 0 0 0 0 0 0 0 0 0 0 0
0 0 0 0 0 0 0 0 0 0 0 0 0
0 0 0 0 0 0 0 0 0 0 0 0 0

0 0 0 0 0 0 0 0 0 0 0 0 0
0 0 1 2 0 0 0 0
"Viruses;Uroviricota;Caudoviricetes;Caudoviricetes__na;Caudoviricetes__na;Caudoviricetes__na;Myoviridae_s
p._ctsK93" 0 0 0 0 0 0 0 0 0 0 0 0
0 0 5 0 3 0 0 0 0 0 0 0 0
0 0 0 0 0 0 0 0 0 0 0 0 0
0 0 0 0 0 0 0 0 0 0 0 0 0
0 0 0 0 0 0 0 0 0 0 0 0 0
0 0 0 0 0 0 0 0
"Viruses;Uroviricota;Caudoviricetes;Caudoviricetes__na;Caudoviricetes__na;Caudoviricetes__na;Myoviridae_s
p._ctsQA11" 0 0 0 0 0 0 0 0 0 0 0 0
0 0 0 0 0 0 0 0 0 0 0 0 0
0 0 0 0 0 0 0 0 0 0 0 0 0
0 0 0 0 0 0 0 0 0 0 0 0 0
0 1 0 0 0 0 0 0 0 0 0 0 0
0 0 0 0 0 0 0 0
"Viruses;Uroviricota;Caudoviricetes;Caudoviricetes__na;Caudoviricetes__na;Caudoviricetes__na;Myoviridae_s
p._ctt8G1" 0 0 0 0 0 0 0 0 0 0 0 0
2 1 0 0 0 0 0 1 0 0 0 0 3
1 0 0 1 0 0 0 0 0 0 0 115 254
2 4 0 640 1 0 0 0 0 0 0 1 0
0 5 1 0 0 0 0 0 0 1 0 0 0
0 0 0 1 0 0 0 0
"Viruses;Uroviricota;Caudoviricetes;Caudoviricetes__na;Caudoviricetes__na;Caudoviricetes__na;Myoviridae_s
p._ctTfa5" 0 0 0 0 0 0 0 0 0 0 0 0
0 0 0 0 0 0 0 0 0 0 0 0 1
0 3 0 1 0 0 0 0 0 0 0 0 0
0 0 1 0 3 3 4 0 0 0 2 1 0
0 0 0 0 0 0 0 0 0 0 0 0 0
0 0 0 0 0 0 0 0
"Viruses;Uroviricota;Caudoviricetes;Caudoviricetes__na;Caudoviricetes__na;Caudoviricetes__na;Myoviridae_s
p._ctThM1" 0 0 0 0 0 1 0 0 0 0 0 0
0 0 0 0 0 6 0 0 0 0 0 0 0
0 0 0 0 0 0 0 0 0 0 0 0 0
0 0 0 0 0 0 1 0 14 1 0 0 0
0 0 0 0 0 0 0 0 0 0 0 0 0
0 0 0 0 0 0 0 0
"Viruses;Uroviricota;Caudoviricetes;Caudoviricetes__na;Caudoviricetes__na;Caudoviricetes__na;Myoviridae_s
p._ctTOm1" 0 0 0 0 0 0 0 0 0 0 0 0
0 2 0 1 0 0 0 2 0 0 0 1 6
1 0 0 0 0 1 1 0 0 0 0 0 0
0 0 0 0 0 0 0 0 0 0 0 0 0
0 0 0 0 2 0 0 0 0 0 0 0 0
0 0 0 0 0 0 0 0
"Viruses;Uroviricota;Caudoviricetes;Caudoviricetes__na;Caudoviricetes__na;Caudoviricetes__na;Myoviridae_s
p._cttp71" 0 0 0 0 0 0 0 0 0 0 0 0
0 0 0 0 0 0 0 0 0 0 0 0 0
0 0 0 0 0 0 0 0 0 0 0 0 0
0 0 0 2 0 0 0 0 0 0 0 0 0

0 0 0 0 0 0 0 0 0 0 0 0 0
0 0 0 0 0 0 0 0
"Viruses;Uroviricota;Caudoviricetes;Caudoviricetes__na;Caudoviricetes__na;Caudoviricetes__na;Myoviridae_sp._ctTrm2" 0 0 0 0 0 0 0 0 0 0 0 0
0 1 0 0 0 0 0 0 0 0 0 0 0
0 0 0 0 0 0 0 0 0 0 0 1 4
0 0 0 0 0 0 0 0 0 0 0 0 0
0 0 0 0 0 0 0 0 0 2 0 0 0
0 0 1 1 0 0 0 0
"Viruses;Uroviricota;Caudoviricetes;Caudoviricetes__na;Caudoviricetes__na;Caudoviricetes__na;Myoviridae_sp._cttWQ44" 0 0 0 0 0 0 0 0 0 0 0 0
0 1 1 0 0 0 0 0 0 0 0 0 0
0 0 0 0 0 0 0 0 0 0 0 7 2
2 0 0 0 0 0 0 0 0 0 0 0 0
0 0 0 9 12 0 0 0 0 0 0 0 0
0 0 5 6 0 0 0 0
"Viruses;Uroviricota;Caudoviricetes;Caudoviricetes__na;Caudoviricetes__na;Caudoviricetes__na;Myoviridae_sp._ctu6J18" 0 0 0 0 0 0 0 0 0 0 0 0
0 0 0 0 0 0 0 3 0 1 0 0 0
1 0 0 0 0 0 1 0 0 0 0 0 1
0 0 0 0 1 0 0 0 0 0 0 0 0
0 0 0 5 4 0 0 0 0 0 3 0 0
0 0 3 1 0 0 0 0
"Viruses;Uroviricota;Caudoviricetes;Caudoviricetes__na;Caudoviricetes__na;Caudoviricetes__na;Myoviridae_sp._ctuIn11" 0 0 0 0 0 0 0 0 0 0 0 0
0 0 0 0 0 0 0 0 0 0 0 0 0
0 0 0 0 0 2 2 0 0 3 0 0 0
0 0 0 0 0 0 0 0 0 0 0 0 0
0 0 0 0 0 0 0 0 0 0 0 0 0
0 0 0 0 0 0 0 0
"Viruses;Uroviricota;Caudoviricetes;Caudoviricetes__na;Caudoviricetes__na;Caudoviricetes__na;Myoviridae_sp._ctuSi15" 0 0 0 0 0 0 0 0 0 0 0 0
0 0 0 0 0 0 0 0 0 0 0 0 7
9 0 0 0 0 0 0 0 0 0 0 1 0
0 0 0 0 0 0 0 0 0 0 17 8 0
0 0 0 0 2 0 0 0 0 0 0 0 0
0 0 6 7 0 0 0 0
"Viruses;Uroviricota;Caudoviricetes;Caudoviricetes__na;Caudoviricetes__na;Caudoviricetes__na;Myoviridae_sp._ctUX613" 0 0 0 0 0 0 0 0 0 0 0 0
5 2 0 0 0 0 0 0 0 0 0 0 0
0 0 0 0 0 4 3 0 0 0 0 1 0
0 0 0 0 0 0 0 0 0 0 0 0 0
0 0 0 3 2 0 0 0 0 0 0 0 0
0 0 0 2 0 0 0 0
"Viruses;Uroviricota;Caudoviricetes;Caudoviricetes__na;Caudoviricetes__na;Caudoviricetes__na;Myoviridae_sp._ctv1i11" 0 0 0 0 0 0 0 0 0 0 0 0
0 0 0 0 0 0 1 0 0 0 0 0 0
0 0 0 0 0 0 0 0 0 0 0 0 0
0 0 0 0 0 0 0 0 0 0 0 0 0

0 0 0 0 0 0 0 0 0 0 0 0 0
0 0 0 0 0 0 0 0
"Viruses;Uroviricota;Caudoviricetes;Caudoviricetes__na;Caudoviricetes__na;Caudoviricetes__na;Myoviridae_s
p._ctv9K3" 0 0 0 0 0 0 0 0 0 0 0 0
0 0 0 0 0 0 1 2 0 0 2 0 0
0 0 0 0 0 0 0 0 0 0 0 1 0
0 0 0 0 0 0 0 0 0 0 0 0 0
0 0 0 0 0 0 0 0 0 1 4 0 0
0 0 2 2 0 0 0 0
"Viruses;Uroviricota;Caudoviricetes;Caudoviricetes__na;Caudoviricetes__na;Caudoviricetes__na;Myoviridae_s
p._ctVCj30" 0 0 0 0 0 0 0 0 0 0 0 0
0 0 0 0 0 0 0 0 0 0 0 0 0
0 0 0 0 0 0 1 0 0 0 0 8 1
0 0 0 0 0 0 0 0 21 0 0 0 0
0 0 0 13 19 0 0 0 0 0 0 0 0
0 0 3 6 0 0 0 0
"Viruses;Uroviricota;Caudoviricetes;Caudoviricetes__na;Caudoviricetes__na;Caudoviricetes__na;Myoviridae_s
p._ctVeR24" 0 0 0 0 0 0 0 0 0 0 0 0
0 0 0 0 0 0 0 0 0 0 0 0 0
0 0 0 0 0 1 0 0 0 0 0 0 0
0 0 0 0 0 0 0 0 0 0 0 0 0
0 0 0 4 2 0 0 0 0 0 0 0 0
0 0 0 0 0 0 0 0
"Viruses;Uroviricota;Caudoviricetes;Caudoviricetes__na;Caudoviricetes__na;Caudoviricetes__na;Myoviridae_s
p._ctvns3" 0 0 0 0 0 0 0 0 0 0 0 0
0 0 0 0 0 0 0 0 0 0 0 0 0
0 0 0 0 0 0 0 0 0 0 0 0 0
0 0 0 0 0 1 0 0 0 0 0 0 0
0 0 0 0 0 0 0 0 0 0 0 0 0
0 0 0 0 0 0 0 0
"Viruses;Uroviricota;Caudoviricetes;Caudoviricetes__na;Caudoviricetes__na;Caudoviricetes__na;Myoviridae_s
p._ctW7Z6" 0 0 0 0 0 0 0 0 0 0 0 0
0 0 0 0 0 0 0 0 0 0 0 0 4
0 1 0 0 0 0 0 0 0 0 0 0 0
0 0 0 0 0 0 0 0 0 0 0 0 0
0 0 0 0 0 0 0 0 0 0 0 0 0
0 0 0 0 0 0 0 0
"Viruses;Uroviricota;Caudoviricetes;Caudoviricetes__na;Caudoviricetes__na;Caudoviricetes__na;Myoviridae_s
p._ctWaE18" 0 0 0 0 0 0 0 0 0 0 0 0
0 0 0 0 0 0 0 0 0 0 0 0 0
0 0 0 0 0 0 0 0 0 0 0 0 0
0 0 0 0 0 0 0 0 0 0 1 0 0
0 0 0 0 0 0 0 0 0 0 0 0 0
0 0 0 0 0 0 0 0
"Viruses;Uroviricota;Caudoviricetes;Caudoviricetes__na;Caudoviricetes__na;Caudoviricetes__na;Myoviridae_s
p._ctWPU11" 0 0 0 0 0 0 0 0 0 0 0 0
0 0 0 0 0 0 0 0 0 0 0 0 0
0 0 0 0 0 3 1 0 0 0 0 0 0
0 0 0 0 0 0 0 0 0 0 0 0 0

0 0 0 4 7 0 0 0 0 0 0 0 0
0 0 3 7 0 1 0 0
"Viruses;Uroviricota;Caudoviricetes;Caudoviricetes__na;Caudoviricetes__na;Caudoviricetes__na;Myoviridae_s
p._ctWXg38" 0 0 0 0 0 0 0 0 0 0 0 0
0 0 0 0 0 0 0 0 0 0 0 0 0
0 0 0 0 0 0 0 0 0 0 0 2 1
0 0 0 0 0 0 0 0 0 0 0 0 0
0 0 0 0 0 0 0 0 0 0 0 0 0
0 0 0 0 0 0 0 0
"Viruses;Uroviricota;Caudoviricetes;Caudoviricetes__na;Caudoviricetes__na;Caudoviricetes__na;Myoviridae_s
p._ctX172" 0 0 0 0 0 0 0 0 0 0 0 0
8 3 5 4 3 13 0 0 0 0 0 0 0
0 0 0 0 0 0 0 0 0 0 0 1 0
0 0 0 0 0 0 0 0 0 0 0 0 0
0 0 0 0 0 0 0 0 0 2 0 0 0
0 0 2 0 0 0 0 0
"Viruses;Uroviricota;Caudoviricetes;Caudoviricetes__na;Caudoviricetes__na;Caudoviricetes__na;Myoviridae_s
p._ctxbQ4" 0 0 0 0 0 0 0 0 0 0 0 0
0 0 1 0 0 0 0 0 0 0 0 0 0
0 0 0 0 0 0 0 0 0 0 0 0 0
0 0 0 0 0 0 0 0 0 0 0 0 0
0 0 0 0 0 0 0 0 0 0 0 0 0
0 0 0 0 0 0 0 0
"Viruses;Uroviricota;Caudoviricetes;Caudoviricetes__na;Caudoviricetes__na;Caudoviricetes__na;Myoviridae_s
p._ctxi06" 0 0 0 0 0 0 0 0 0 0 0 0
1 0 0 0 0 0 0 0 0 0 0 0 0
0 0 0 0 0 0 0 0 0 4 0 0 0
0 0 0 0 0 0 0 0 0 0 0 0 0
0 0 0 0 0 0 0 0 0 0 0 0 0
0 0 0 0 0 0 0 0
"Viruses;Uroviricota;Caudoviricetes;Caudoviricetes__na;Caudoviricetes__na;Caudoviricetes__na;Myoviridae_s
p._ctxlX31" 0 0 0 0 0 0 0 0 0 0 0 0
0 1 0 0 0 0 1 0 0 0 0 0 0
0 0 0 0 0 6 5 0 0 0 0 0 0
0 0 0 0 0 0 0 0 0 0 0 0 0
0 0 0 0 0 0 0 0 0 0 0 0 0
0 0 0 0 0 0 0 0
"Viruses;Uroviricota;Caudoviricetes;Caudoviricetes__na;Caudoviricetes__na;Caudoviricetes__na;Myoviridae_s
p._ctxpQ22" 0 0 0 0 0 0 0 0 0 0 0 0
0 0 0 0 0 0 0 0 0 0 0 0 0
0 0 0 0 0 0 0 0 0 0 0 0 0
0 0 0 0 1 2 0 0 0 0 0 0 0
0 0 0 0 0 0 0 0 0 0 0 0 0
0 0 0 0 0 0 0 0
"Viruses;Uroviricota;Caudoviricetes;Caudoviricetes__na;Caudoviricetes__na;Caudoviricetes__na;Myoviridae_s
p._ctXRl20" 0 0 0 0 0 0 0 0 0 0 0 0
0 0 0 0 0 0 0 0 0 0 0 0 0
0 0 0 0 0 0 0 0 0 5 0 0 0
0 0 0 0 0 0 0 0 0 0 0 0 0

0 0 0 0 0 0 0 0 0 0 0 0 0
0 0 1 1 0 0 0 0
"Viruses;Uroviricota;Caudoviricetes;Caudoviricetes__na;Caudoviricetes__na;Caudoviricetes__na;Myoviridae_s
p._ctXVO17" 0 0 0 0 0 0 0 0 0 0 0 0
0 0 0 0 0 0 0 0 0 0 0 0 0
0 0 0 0 0 2 4 0 0 0 0 0 0
0 0 0 0 0 0 0 0 0 0 0 0 0
0 0 0 4 6 0 0 0 0 0 0 0 0
0 0 0 0 0 0 0 0
"Viruses;Uroviricota;Caudoviricetes;Caudoviricetes__na;Caudoviricetes__na;Caudoviricetes__na;Myoviridae_s
p._ctXwe21" 0 0 0 0 0 0 0 0 0 0 0 0
0 0 0 0 0 0 0 0 0 0 0 0 0
0 0 0 0 0 0 0 0 0 0 0 0 3
0 0 0 0 0 0 0 0 0 0 1 1 0
0 0 0 0 0 0 0 0 0 2 0 0 0
0 0 8 2 0 0 0 0
"Viruses;Uroviricota;Caudoviricetes;Caudoviricetes__na;Caudoviricetes__na;Caudoviricetes__na;Myoviridae_s
p._ctY1522" 0 0 0 0 0 0 0 0 0 0 0 0
0 0 0 1 0 0 0 0 0 0 0 0 0
0 0 0 0 0 0 0 0 0 0 0 0 0
0 0 0 0 0 0 0 0 0 0 0 0 0
0 0 0 0 0 0 0 0 0 0 0 0 0
0 0 0 0 0 0 0 0
"Viruses;Uroviricota;Caudoviricetes;Caudoviricetes__na;Caudoviricetes__na;Caudoviricetes__na;Myoviridae_s
p._ctYA416" 0 0 0 0 0 0 0 0 6 0 0 0
0 0 0 0 0 0 0 0 0 0 0 0 0
0 0 0 0 0 0 0 0 0 0 0 0 0
0 0 0 2 0 0 0 0 0 0 0 0 0
0 0 0 0 0 0 0 0 0 0 0 0 0
0 0 0 0 0 0 0 0
"Viruses;Uroviricota;Caudoviricetes;Caudoviricetes__na;Caudoviricetes__na;Caudoviricetes__na;Myoviridae_s
p._ctYGJ17" 0 0 0 0 0 0 0 0 0 0 0 0
0 0 0 0 0 0 0 0 0 0 0 0 3
5 0 0 0 0 0 0 0 0 0 0 2 0
0 0 0 0 0 0 0 0 0 0 2 0 0
0 0 1 1 0 0 0 0 0 4 2 0 0
0 0 1 3 0 0 0 0
"Viruses;Uroviricota;Caudoviricetes;Caudoviricetes__na;Caudoviricetes__na;Caudoviricetes__na;Myoviridae_s
p._ctyhU11" 0 0 0 0 0 0 0 0 0 0 0 0
0 0 0 0 0 0 0 0 0 0 0 0 1
0 1 0 0 0 0 0 0 0 0 0 0 0
0 0 0 0 0 0 0 0 0 0 2 0 4
0 0 0 0 0 0 0 0 0 0 0 0 0
0 0 0 0 0 0 0 0
"Viruses;Uroviricota;Caudoviricetes;Caudoviricetes__na;Caudoviricetes__na;Caudoviricetes__na;Myoviridae_s
p._ctyWv1" 0 0 0 0 0 0 0 0 0 0 0 0
0 0 0 0 0 0 0 0 0 0 0 0 0
0 0 0 0 0 1 0 0 0 0 0 0 0
0 0 0 0 0 0 0 0 0 0 0 0 0

0 0 0 1 0 0 0 0 0 2 4 0 0
0 0 41 29 0 4 0 0
"Viruses;Uroviricota;Caudoviricetes;Caudoviricetes__na;Caudoviricetes__na;Caudoviricetes__na;Myoviridae_sp._ctZ2t4" 0 0 0 0 0 0 0 0 0 0 0 0
1 0 0 0 0 0 0 0 0 0 0 0 0
0 0 0 0 0 0 0 0 0 0 0 0 0
0 0 0 0 0 0 0 0 0 0 0 0 0
0 0 0 0 0 0 0 0 0 0 0 0 0
0 0 0 0 0 0 0 0
"Viruses;Uroviricota;Caudoviricetes;Caudoviricetes__na;Caudoviricetes__na;Caudoviricetes__na;Myoviridae_sp._ctZDd15" 0 0 0 0 0 0 0 0 0 0 0 0
0 0 0 0 0 0 0 0 0 0 0 0 0
0 0 0 0 0 0 0 0 0 0 0 0 0
0 0 0 0 0 2 0 0 0 0 0 0 0
0 0 0 0 1 0 0 0 0 0 0 0 0
0 0 0 0 0 0 0 0
"Viruses;Uroviricota;Caudoviricetes;Caudoviricetes__na;Caudoviricetes__na;Caudoviricetes__na;Myoviridae_sp._ctzS633" 0 0 0 0 0 0 0 0 0 0 0 0
0 0 0 0 0 0 0 0 0 0 0 0 0
0 0 0 0 0 0 0 0 0 0 0 0 0
0 0 0 0 0 0 0 0 0 0 0 0 0
0 0 0 3 0 0 0 0 0 0 0 0 0
0 0 0 0 0 0 0 0
"Viruses;Uroviricota;Caudoviricetes;Caudoviricetes__na;Caudoviricetes__na;Caudoviricetes__na;Myoviridae_sp._ctzwE5" 0 0 0 0 0 0 0 0 0 0 0 0
0 0 0 0 0 0 0 0 0 0 0 0 0
0 0 0 0 0 0 0 0 0 1 0 0 0
0 0 0 0 0 0 0 0 0 0 0 0 0
0 0 0 0 0 0 0 0 0 0 0 0 0
0 0 0 0 0 0 0 0
"Viruses;Uroviricota;Caudoviricetes;Caudoviricetes__na;Caudoviricetes__na;Caudoviricetes__na;Myoviridae_sp._ctzyI3" 0 0 0 0 0 0 0 0 0 0 0 0
0 0 0 0 0 0 4 3 1 2 0 0 0
0 0 0 0 0 0 0 0 0 0 0 0 0
0 0 0 0 0 0 0 0 0 0 1 1 0
0 0 0 0 0 0 0 0 0 0 0 0 0
0 0 0 0 0 0 0 0
"Viruses;Uroviricota;Caudoviricetes;Caudoviricetes__na;Caudoviricetes__na;Caudoviricetes__na;Myoviridae_sp._ctZYN8" 0 0 0 0 0 0 0 0 0 0 0 0
1 0 0 0 0 0 0 0 0 0 0 0 0
0 0 0 0 0 0 0 0 0 0 0 0 0
0 0 0 0 0 0 0 0 0 0 0 0 0
0 0 0 0 0 0 0 0 0 0 0 0 0
0 0 0 0 0 0 0 0
"Viruses;Uroviricota;Caudoviricetes;Caudoviricetes__na;Caudoviricetes__na;Caudoviricetes__na;Nitrincola_phage_1M3-16" 0 0 0 0 5 0 0 0 0 0 0 0
0 0 0 0 0 0 0 0 0 0 0 0 0
0 0 0 0 0 0 0 0 0 0 0 0 0
0 0 0 0 0 0 0 0 0 0 0 0 0

0 0 0 0 0 0 0 0 0 0 0 0 0
0 0 0 0 0 0 0 0
"Viruses;Uroviricota;Caudoviricetes;Caudoviricetes__na;Caudoviricetes__na;Caudoviricetes__na;Nocardia_pha
ge_NTR1" 0 0 1 0 1 0 0 0 0 0 0 0
0 0 0 0 0 0 0 0 0 0 0 0 0
0 0 0 0 0 0 0 0 0 0 0 0 0
0 0 0 0 0 0 0 0 0 0 0 0 0
0 0 0 0 0 0 0 0 0 0 0 0 0
0 0 0 0 0 0 0 0
"Viruses;Uroviricota;Caudoviricetes;Caudoviricetes__na;Caudoviricetes__na;Caudoviricetes__na;Nostoc_phage
_A1" 0 0 0 0 1 0 0 0 0 0 0 0 0
0 0 0 0 0 0 0 0 0 0 0 0 0
0 0 0 0 0 0 0 0 0 0 0 0 0
0 0 0 0 0 0 0 0 0 0 0 0 0
0 0 0 0 0 0 0 0 0 0 0 0 0
0 0 0 0 0 0 0
"Viruses;Uroviricota;Caudoviricetes;Caudoviricetes__na;Caudoviricetes__na;Caudoviricetes__na;Oceanospirillu
m_phage_vB_OsaM_PD0307" 0 0 0 1 0 0 0 0 0 0
0 0 0 0 0 0 0 0 0 0 0 0 0
0 0 0 0 0 0 0 0 0 0 0 0 0
0 0 0 0 0 0 0 0 0 0 0 0 0
0 0 0 0 0 0 0 0 0 0 0 0 0
0 0 0 0 0 0 0 0 0 0
"Viruses;Uroviricota;Caudoviricetes;Caudoviricetes__na;Caudoviricetes__na;Caudoviricetes__na;Ochrobactrum
_phage_vB_OspP_OH" 0 0 1 0 0 0 0 0 0 0 0
0 0 0 0 0 0 0 0 0 0 0 0 0
0 0 0 0 0 0 0 0 0 0 0 0 0
0 0 0 0 0 0 0 0 0 0 0 0 0
0 0 0 0 0 0 0 0 0 0 0 0 0
0 0 0 0 0 0 0 0 0
"Viruses;Uroviricota;Caudoviricetes;Caudoviricetes__na;Caudoviricetes__na;Caudoviricetes__na;Pantoea_phag
e_AH01" 0 0 1 0 0 0 0 0 0 0 0 0
0 0 0 0 0 0 0 0 0 0 0 0 0
0 0 0 0 0 0 0 0 0 0 0 0 0
0 0 0 0 0 0 0 0 0 0 0 0 0
0 0 0 0 0 0 0 0 0 0 0 0 0
0 0 0 0 0 0 0 0
"Viruses;Uroviricota;Caudoviricetes;Caudoviricetes__na;Caudoviricetes__na;Caudoviricetes__na;Pantoea_phag
e_vB_PagM_AAM22" 0 0 0 1 0 0 0 0 0 0 0
0 0 0 0 0 0 0 0 0 0 0 0 0
0 0 0 0 0 0 0 0 0 0 0 2 0
0 0 0 0 0 0 0 0 0 0 1 0 0
0 0 0 0 0 0 0 0 0 0 0 0 0
0 0 0 0 0 0 0 0 0
"Viruses;Uroviricota;Caudoviricetes;Caudoviricetes__na;Caudoviricetes__na;Caudoviricetes__na;Parabacteroide
s_phage_PDS1" 0 0 0 0 0 0 0 0 0 0 0 0
0 0 0 0 0 0 0 0 0 0 0 0 0
0 0 0 0 0 0 0 0 0 0 0 0 1
0 0 0 0 0 0 0 0 0 0 0 0 0

0 0 245 0 0 0 0 0 0 0 0 0 0
0 0 1 1 0 0 0 0
"Viruses;Uroviricota;Caudoviricetes;Caudoviricetes__na;Caudoviricetes__na;Caudoviricetes__na;Parabacteroides_phage_PF672P1" 0 0 0 0 0 0 0 0 0 0 0
0 0 0 0 1 0 0 0 0 0 0 0 0
0 0 0 0 0 0 0 0 0 0 0 0 0
0 0 0 0 0 0 1 0 0 0 0 0 0
0 0 0 0 0 0 0 0 0 0 0 0 0
0 0 0 0 0 0 0 0 0
"Viruses;Uroviricota;Caudoviricetes;Caudoviricetes__na;Caudoviricetes__na;Caudoviricetes__na;Paracoccus_phage_vB_PmaP_KLEP18-1" 0 0 0 2 0 0 0 0 0 0
0 0 0 0 0 0 0 0 0 0 0 0 0
0 0 0 0 0 0 0 0 0 0 0 0 0
0 0 0 0 0 0 0 0 0 0 0 0 0
0 0 0 0 0 0 0 0 0 0 0 0 0
0 0 0 0 0 0 0 0 0 0
"Viruses;Uroviricota;Caudoviricetes;Caudoviricetes__na;Caudoviricetes__na;Caudoviricetes__na;Pararheinheimera_phage_vB_PsoM_KLER1-1" 0 0 0 0 2 0 0 0 0 0
0 0 0 0 0 0 0 0 0 0 0 0 0
0 0 0 0 0 0 0 0 0 0 0 0 0
0 0 0 0 0 0 0 0 0 0 0 0 0
0 0 0 0 0 0 0 0 0 0 0 0 0
0 0 0 0 0 0 0 0 0 0
"Viruses;Uroviricota;Caudoviricetes;Caudoviricetes__na;Caudoviricetes__na;Caudoviricetes__na;Pelagibacter_phage_HTVC008M" 0 0 0 0 4 1 0 0 0 0 0
0 0 0 0 0 0 0 0 0 0 0 0 0
0 0 0 0 0 0 0 0 0 0 0 0 0
0 0 0 0 0 0 0 0 0 0 0 0 0
0 0 0 0 0 0 0 0 0 0 0 0 0
0 0 0 0 0 0 0 0 0
"Viruses;Uroviricota;Caudoviricetes;Caudoviricetes__na;Caudoviricetes__na;Caudoviricetes__na;Phage_33_17"
0 0 1 0 0 0 0 0 0 0 0 0 0
0 0 0 0 0 0 0 0 0 0 0 0 0
0 0 0 0 0 0 0 0 0 0 0 0 0
0 0 0 0 0 0 0 0 0 0 0 0 0
0 0 0 0 0 0 0 0 0 0 0 0 0
0 0 0 0 0 0 0
"Viruses;Uroviricota;Caudoviricetes;Caudoviricetes__na;Caudoviricetes__na;Caudoviricetes__na;Phage_5P_1"
0 0 0 0 1 0 0 0 0 0 0 0 0
0 0 0 0 0 0 0 0 0 0 0 0 0
0 0 0 0 0 0 0 0 0 0 0 0 0
0 0 0 0 0 0 0 0 0 0 0 0 0
0 0 0 0 0 0 0 0 0 0 0 0 0
0 0 0 0 0 0 0
"Viruses;Uroviricota;Caudoviricetes;Caudoviricetes__na;Caudoviricetes__na;Caudoviricetes__na;Phage_5P_2"
0 0 0 0 0 0 0 0 0 0 0 0 0
0 0 0 0 0 0 0 0 0 0 0 0 0
0 0 0 0 0 0 0 0 0 0 0 0 0
0 0 0 0 0 0 0 0 0 0 0 0 0

0 0 0 1 0 0 0 0 0 0 0 0 0
0 0 0 0 0 0 0
"Viruses;Uroviricota;Caudoviricetes;Caudoviricetes__na;Caudoviricetes__na;Caudoviricetes__na;Phage_5P_3"
0 0 0 2 0 3 0 0 0 0 0 0 0
0 0 0 0 0 0 0 0 0 0 0 0 0
0 0 0 0 0 0 0 0 0 0 0 0 0
0 0 0 0 0 0 0 0 1 0 0 0 0
0 0 0 0 0 0 0 0 0 0 0 0 0
0 0 0 0 0 0 0
"Viruses;Uroviricota;Caudoviricetes;Caudoviricetes__na;Caudoviricetes__na;Caudoviricetes__na;Phage_CBW1004C-Prop1" 0 0 0 0 0 1 0 0 0 0 0 0
0 0 0 0 0 0 0 0 0 0 0 0 0
0 0 0 0 0 0 0 0 0 0 0 0 0
0 0 0 0 0 0 0 0 0 0 0 0 0
0 0 0 0 0 0 0 0 0 0 0 0 0
0 0 0 0 0 0 0 0
"Viruses;Uroviricota;Caudoviricetes;Caudoviricetes__na;Caudoviricetes__na;Caudoviricetes__na;Phage_NCTB"
0 0 0 1 0 35 0 0 0 0 0 0 0
0 0 0 0 0 0 0 0 0 0 0 0 0
0 0 0 0 0 0 0 0 0 0 0 0 0
0 0 0 0 0 0 0 0 0 0 0 0 0
0 0 0 0 0 0 0 0 0 0 0 0 0
0 0 0 0 0 0 0
"Viruses;Uroviricota;Caudoviricetes;Caudoviricetes__na;Caudoviricetes__na;Caudoviricetes__na;Plesiomonas_phage_phiP4-7" 0 0 0 0 10 0 0 0 0 0 0 0
0 0 0 0 0 0 0 0 0 0 0 0 0
0 0 0 0 0 0 0 0 0 0 0 0 0
0 0 0 0 0 0 0 0 0 0 0 0 0
0 0 0 0 0 0 0 0 0 0 0 0 0
0 0 0 0 0 0 0 0
"Viruses;Uroviricota;Caudoviricetes;Caudoviricetes__na;Caudoviricetes__na;Caudoviricetes__na;Podoviridae_sp._ct1h53" 0 0 0 0 0 0 0 0 0 0 0 0
0 0 0 0 0 0 3 1 0 0 0 2 0
0 0 0 0 0 0 0 0 0 0 0 0 0
0 0 0 0 0 0 0 0 0 0 1 1 0
0 0 0 0 0 0 0 0 0 1 0 0 0
0 0 0 0 0 0 0 0
"Viruses;Uroviricota;Caudoviricetes;Caudoviricetes__na;Caudoviricetes__na;Caudoviricetes__na;Podoviridae_sp._ct3k57" 0 0 0 0 0 0 0 0 0 0 0 0
0 0 0 0 0 0 0 0 0 0 0 0 0
0 0 0 0 0 0 0 0 0 0 0 0 0
0 0 0 0 0 0 0 0 0 0 0 0 0
0 0 0 0 0 0 0 0 0 9 1 0 0
0 0 0 0 0 0 0 0
"Viruses;Uroviricota;Caudoviricetes;Caudoviricetes__na;Caudoviricetes__na;Caudoviricetes__na;Podoviridae_sp._ct53O25" 0 0 0 0 0 0 0 0 0 0 0 0
0 0 0 0 0 0 0 0 0 0 0 0 0
0 0 0 0 0 0 0 0 0 0 0 0 0
0 0 0 0 0 0 0 0 0 0 0 0 0

0 0 1 0 0 0 0 0 0 0 0 0 0
0 0 0 0 0 0 0 0
"Viruses;Uroviricota;Caudoviricetes;Caudoviricetes__na;Caudoviricetes__na;Caudoviricetes__na;Podoviridae_s
p._ct5O42" 0 0 0 0 0 0 0 0 0 0 0 0
0 2 0 0 0 0 0 0 0 0 0 0 0
0 0 0 0 0 0 0 0 0 1 0 10 6
0 1 0 10 0 0 0 0 0 0 0 0 0
0 0 0 0 0 0 0 0 0 0 0 0 0
0 0 0 0 0 0 0 0
"Viruses;Uroviricota;Caudoviricetes;Caudoviricetes__na;Caudoviricetes__na;Caudoviricetes__na;Podoviridae_s
p._ct7Kl21" 0 0 0 0 0 0 0 0 0 0 0 0
1 0 0 0 0 0 0 0 0 0 0 0 0
0 0 0 0 0 0 0 0 0 0 0 0 0
0 0 0 0 0 0 0 0 0 0 0 0 0
0 0 0 1 2 0 0 0 0 0 0 0 0
0 0 0 0 0 0 0 0
"Viruses;Uroviricota;Caudoviricetes;Caudoviricetes__na;Caudoviricetes__na;Caudoviricetes__na;Podoviridae_s
p._ct8eG6" 0 0 0 0 0 0 0 0 0 0 0 0
0 0 0 0 0 0 0 0 0 0 0 0 0
0 0 0 0 0 0 0 0 0 0 0 0 0
0 0 0 0 0 0 0 0 0 0 1 0 0
0 0 0 0 0 0 0 0 0 0 0 0 0
0 0 0 0 0 0 0 0
"Viruses;Uroviricota;Caudoviricetes;Caudoviricetes__na;Caudoviricetes__na;Caudoviricetes__na;Podoviridae_s
p._ct8Lf7" 0 0 0 0 0 0 0 0 0 0 0 0
6 10 54 51 33 48 0 0 0 0 0 0 3
1 1 0 0 0 3 1 0 0 0 0 0 1
2 0 14 0 38 18 439 93 0 2 5 4 0
0 1 0 0 0 0 0 0 0 0 0 0 0
0 0 0 0 0 0 0 0
"Viruses;Uroviricota;Caudoviricetes;Caudoviricetes__na;Caudoviricetes__na;Caudoviricetes__na;Podoviridae_s
p._ct90d35" 0 0 0 0 0 0 0 0 0 0 0 0
0 0 0 0 0 0 0 0 0 0 0 0 0
0 0 0 0 0 0 0 0 0 0 0 6 3
0 0 0 0 0 0 0 0 0 0 0 0 0
0 0 0 0 1 0 0 0 0 0 0 0 0
0 0 3 5 0 1 0 0
"Viruses;Uroviricota;Caudoviricetes;Caudoviricetes__na;Caudoviricetes__na;Caudoviricetes__na;Podoviridae_s
p._ct9H612" 0 0 0 0 0 0 0 0 0 0 0 0
0 0 0 0 0 0 0 0 0 0 0 0 0
0 0 0 0 0 0 0 0 0 7 0 0 0
0 0 0 0 0 0 0 0 0 0 0 0 0
0 0 0 0 0 0 0 0 0 0 0 0 0
0 0 0 0 0 0 0 0
"Viruses;Uroviricota;Caudoviricetes;Caudoviricetes__na;Caudoviricetes__na;Caudoviricetes__na;Podoviridae_s
p._cta463" 0 0 0 0 0 0 0 0 0 0 0 0
0 0 0 0 0 0 0 0 0 0 0 0 0
0 0 0 0 0 0 0 0 0 0 0 0 0
0 0 0 0 0 0 0 0 0 0 0 0 0

0 0 0 1 0 0 0 0 0 0 0 0 0
0 0 0 0 0 0 0 0
"Viruses;Uroviricota;Caudoviricetes;Caudoviricetes__na;Caudoviricetes__na;Caudoviricetes__na;Podoviridae_sp._ctAmM4" 0 0 0 0 0 0 0 0 0 0 0 0
7 5 1 1 0 0 0 0 0 0 0 0 0
0 0 0 0 0 0 1 0 0 0 0 0 0
0 0 0 0 10 52 0 0 0 0 0 0 0
0 0 0 0 0 0 0 0 0 0 0 0 0
0 0 0 0 0 0 0 0
"Viruses;Uroviricota;Caudoviricetes;Caudoviricetes__na;Caudoviricetes__na;Caudoviricetes__na;Podoviridae_sp._ctaNW81" 0 0 0 0 0 0 0 0 0 0 0 0
1 0 0 0 0 0 0 0 0 0 0 0 0
0 0 0 0 0 0 0 0 0 0 0 0 0
0 0 0 0 0 0 0 0 0 0 0 0 0
0 0 0 0 0 0 0 0 0 0 0 0 0
0 0 0 0 0 0 0 0
"Viruses;Uroviricota;Caudoviricetes;Caudoviricetes__na;Caudoviricetes__na;Caudoviricetes__na;Podoviridae_sp._ctaOY15" 0 0 0 0 0 0 0 0 0 0 0 0
0 0 0 0 0 0 0 0 0 0 0 0 0
0 0 0 0 0 0 0 0 0 0 0 0 0
0 0 0 0 0 0 0 0 0 0 0 0 0
0 0 0 0 1 0 0 0 0 0 0 0 0
0 0 0 0 0 0 0 0
"Viruses;Uroviricota;Caudoviricetes;Caudoviricetes__na;Caudoviricetes__na;Caudoviricetes__na;Podoviridae_sp._ctAV815" 0 0 0 0 0 0 0 0 0 0 0 0
0 0 0 0 0 0 0 0 0 0 0 0 0
0 0 0 0 0 0 0 0 0 0 0 0 0
0 0 0 0 0 0 0 0 0 0 0 0 0
0 0 12 0 0 0 0 0 0 0 0 0 0
0 0 3 7 0 1 0 0
"Viruses;Uroviricota;Caudoviricetes;Caudoviricetes__na;Caudoviricetes__na;Caudoviricetes__na;Podoviridae_sp._ctC2p8" 0 0 0 0 0 0 0 0 0 0 0 0
0 0 0 0 0 0 0 0 0 0 0 0 1
9 1 0 0 0 0 0 0 0 0 0 0 2
0 0 0 0 0 1 0 0 0 0 0 2 0
0 0 0 0 1 0 0 0 0 2 0 0 0
0 0 0 2 0 0 0 0
"Viruses;Uroviricota;Caudoviricetes;Caudoviricetes__na;Caudoviricetes__na;Caudoviricetes__na;Podoviridae_sp._ctcKt3" 0 0 0 0 0 0 0 0 0 0 0 0
0 0 0 0 0 0 0 0 0 0 0 0 0
0 0 0 0 0 0 0 0 0 0 0 0 0
0 0 0 0 0 0 0 0 0 0 0 0 0
0 0 5 0 0 0 0 0 0 0 0 0 0
0 0 0 0 0 0 0 0
"Viruses;Uroviricota;Caudoviricetes;Caudoviricetes__na;Caudoviricetes__na;Caudoviricetes__na;Podoviridae_sp._ctdc61" 0 0 0 0 0 0 0 0 0 0 0 0
0 0 0 0 0 0 0 0 0 0 0 0 0
0 0 0 0 0 0 0 0 0 0 0 0 0
0 0 0 0 0 0 0 0 0 0 0 0 0

0	0	0	0	1	0	0	0	0	0	0	0	0
0	0	0	0	0	0	0	0
"Viruses;Uroviricota;Caudoviricetes;Caudoviricetes__na;Caudoviricetes__na;Caudoviricetes__na;Podoviridae_sp._ctDd04"	0	0	0	0	0	0	0	0	0	0	0	0
6	5	4	3	9	77	0	0	0	0	0	0	0
0	0	0	0	0	0	0	0	0	0	0	0	0
0	0	0	0	0	0	0	0	0	0	0	0	0
0	0	0	0	0	0	0	0	0	0	0	0	0
0	0	0	0	0	0	0	0
"Viruses;Uroviricota;Caudoviricetes;Caudoviricetes__na;Caudoviricetes__na;Caudoviricetes__na;Podoviridae_sp._ctDgT26"	0	0	0	0	0	0	0	0	0	0	0	0
0	0	0	0	0	0	0	0	0	0	0	0	0
1	0	0	0	0	0	0	0	0	0	0	0	0
0	0	0	0	0	0	0	0	0	0	0	0	0
0	0	172	0	0	0	0	0	0	0	0	0	0
0	0	7	12	2	3	0	0
"Viruses;Uroviricota;Caudoviricetes;Caudoviricetes__na;Caudoviricetes__na;Caudoviricetes__na;Podoviridae_sp._ctdKF3"	0	0	0	0	0	0	0	0	0	0	0	0
0	0	0	0	0	1	0	1	0	0	0	0	0
0	0	0	0	0	0	0	0	0	0	0	0	0
0	0	0	0	0	0	0	0	0	0	0	0	0
0	0	0	0	0	0	0	0	0	0	0	0	0
0	0	0	0	0	0	0	0
"Viruses;Uroviricota;Caudoviricetes;Caudoviricetes__na;Caudoviricetes__na;Caudoviricetes__na;Podoviridae_sp._ctdRZ1"	0	0	0	0	0	0	0	0	0	0	0	0
0	0	0	0	0	0	2	5	0	0	0	0	0
0	2	0	0	0	0	0	0	0	0	0	1	0
0	0	0	0	0	0	0	0	0	0	0	0	0
0	0	0	0	2	0	0	0	0	0	0	0	0
0	0	2	1	0	0	0	0
"Viruses;Uroviricota;Caudoviricetes;Caudoviricetes__na;Caudoviricetes__na;Caudoviricetes__na;Podoviridae_sp._ctfAL26"	0	0	0	0	0	0	0	0	0	0	0	0
0	0	0	0	0	0	0	0	0	0	0	0	0
0	0	0	0	0	0	0	0	0	0	0	0	0
0	0	0	0	0	0	0	0	0	0	0	0	0
0	0	0	8	4	0	0	0	0	0	0	0	0
0	0	0	0	0	0	0	0
"Viruses;Uroviricota;Caudoviricetes;Caudoviricetes__na;Caudoviricetes__na;Caudoviricetes__na;Podoviridae_sp._ctFkM10"	0	0	0	0	0	0	0	0	0	0	0	0
0	0	0	0	0	0	0	0	0	0	0	2	28
18	24	4	23	0	12	16	0	0	27	0	0	0
0	0	0	0	0	0	0	0	0	0	0	0	0
0	0	0	0	0	0	0	0	0	0	0	0	0
0	0	0	0	0	0	0	0
"Viruses;Uroviricota;Caudoviricetes;Caudoviricetes__na;Caudoviricetes__na;Caudoviricetes__na;Podoviridae_sp._ctfN46"	0	0	0	0	0	0	0	0	0	0	0	0
0	0	0	0	0	0	0	1	0	1	0	0	0
0	0	0	0	0	0	0	0	0	0	0	0	0
0	0	0	0	0	0	0	0	0	0	0	0	0

0 0 0 0 0 0 0 0 0 28 10 0 0
0 0 0 0 0 0 0 0
"Viruses;Uroviricota;Caudoviricetes;Caudoviricetes__na;Caudoviricetes__na;Caudoviricetes__na;Podoviridae_s
p._cthJQ11" 0 0 0 0 0 0 0 0 0 0 0 0
0 0 3 5 0 0 0 0 0 0 0 0 0
0 0 0 0 0 0 0 0 0 0 0 0 0
0 0 0 0 0 0 0 0 0 0 0 0 0
0 0 0 0 0 0 0 0 0 0 0 0 0
0 0 0 0 0 0 0 0
"Viruses;Uroviricota;Caudoviricetes;Caudoviricetes__na;Caudoviricetes__na;Caudoviricetes__na;Podoviridae_s
p._ctHm32" 0 0 0 0 0 0 0 0 0 0 0 0
3 1 0 0 0 3 5 3 2 0 0 0 3
2 2 0 0 0 5 2 0 0 1 0 2 8
0 0 0 0 6 4 0 0 0 0 6 3 0
0 0 0 2 1 0 0 0 0 5 0 0 0
0 0 1 0 0 0 0 0
"Viruses;Uroviricota;Caudoviricetes;Caudoviricetes__na;Caudoviricetes__na;Caudoviricetes__na;Podoviridae_s
p._ctHMt20" 0 0 0 0 0 0 0 0 0 0 0 0
0 0 0 0 0 0 0 0 0 0 0 0 0
0 0 0 0 0 0 0 0 0 0 0 0 0
0 0 0 0 0 0 0 0 0 0 0 0 0
0 0 0 0 0 0 0 0 0 0 0 0 0
0 0 1 1 0 0 0 0
"Viruses;Uroviricota;Caudoviricetes;Caudoviricetes__na;Caudoviricetes__na;Caudoviricetes__na;Podoviridae_s
p._cthVG1" 0 0 0 0 0 0 0 0 0 0 0 0
4 5 0 1 0 0 0 0 0 0 0 0 0
0 2 0 0 0 1 0 0 0 0 0 0 0
0 0 0 0 0 0 0 0 0 0 2 0 0
0 0 0 0 0 0 0 0 0 0 0 0 0
0 0 1 0 0 0 0 0
"Viruses;Uroviricota;Caudoviricetes;Caudoviricetes__na;Caudoviricetes__na;Caudoviricetes__na;Podoviridae_s
p._ctiHu16" 0 0 0 0 0 0 0 0 0 0 0 0
0 0 0 0 0 0 0 0 0 0 0 0 0
0 0 0 0 0 4 4 0 0 18 0 15 11
5 0 0 290 0 0 0 0 0 0 0 0 0
0 1 0 0 0 0 0 0 0 0 0 0 0
0 0 0 0 0 0 0 0
"Viruses;Uroviricota;Caudoviricetes;Caudoviricetes__na;Caudoviricetes__na;Caudoviricetes__na;Podoviridae_s
p._ctIi724" 0 0 0 0 0 0 0 0 0 0 0 0
0 0 0 0 0 0 0 0 0 0 0 0 0
0 0 0 0 0 0 0 0 0 0 0 0 0
0 0 0 0 0 0 0 0 0 0 0 0 0
0 0 0 1 0 0 0 0 0 0 0 0 0
0 0 0 0 0 0 0 0
"Viruses;Uroviricota;Caudoviricetes;Caudoviricetes__na;Caudoviricetes__na;Caudoviricetes__na;Podoviridae_s
p._ctIi96" 0 0 0 0 0 0 0 0 0 0 0 0
0 0 0 0 0 0 0 0 0 0 0 0 0
0 0 0 0 0 0 0 0 0 0 0 0 0
0 0 0 0 0 0 0 0 0 0 0 0 0

0 0 0 0 0 0 0 0 0 1 1 0 0
0 0 0 0 0 0 0 0
"Viruses;Uroviricota;Caudoviricetes;Caudoviricetes__na;Caudoviricetes__na;Caudoviricetes__na;Podoviridae_s
p._ctiJY10" 0 0 0 0 0 0 0 0 0 0 0 0
0 0 0 0 0 0 0 0 0 0 0 0 0
0 0 0 0 0 0 0 0 0 0 0 0 0
1 0 0 0 0 0 0 0 0 0 0 0 0
0 0 0 0 0 0 0 0 0 0 0 0 0
0 0 0 0 0 0 0 0
"Viruses;Uroviricota;Caudoviricetes;Caudoviricetes__na;Caudoviricetes__na;Caudoviricetes__na;Podoviridae_s
p._ctIKM86" 0 0 0 0 0 0 0 0 0 0 0 0
0 0 0 0 0 0 0 0 0 0 0 0 0
0 0 0 0 0 0 0 0 0 0 2 0 0
0 0 0 0 0 0 0 0 0 0 0 0 0
0 0 0 0 0 0 0 0 0 0 0 0 0
0 0 0 0 0 0 0 0
"Viruses;Uroviricota;Caudoviricetes;Caudoviricetes__na;Caudoviricetes__na;Caudoviricetes__na;Podoviridae_s
p._ctIlO27" 0 0 0 0 0 0 0 0 0 0 0 0
1 2 0 0 0 0 0 0 0 0 0 0 0
0 0 0 0 0 0 0 0 0 0 0 0 0
0 0 0 0 0 0 0 0 0 0 0 0 0
0 0 0 0 0 0 0 0 0 1 0 0 0
0 0 0 0 0 0 0 0
"Viruses;Uroviricota;Caudoviricetes;Caudoviricetes__na;Caudoviricetes__na;Caudoviricetes__na;Podoviridae_s
p._ctIpM11" 0 0 0 0 0 0 0 0 0 0 0 0
0 0 0 0 0 0 3 8 0 0 0 0 1
0 0 0 0 0 0 1 0 0 0 0 0 1
0 0 0 0 2 3 0 0 0 0 0 0 0
0 0 0 0 3 0 0 0 0 0 0 0 0
0 0 2 1 0 0 0 0
"Viruses;Uroviricota;Caudoviricetes;Caudoviricetes__na;Caudoviricetes__na;Caudoviricetes__na;Podoviridae_s
p._ctiwu7" 0 0 0 0 0 0 0 0 0 0 0 0
0 0 0 0 0 0 0 0 0 0 0 0 0
0 0 0 0 0 0 0 0 0 0 0 0 0
0 0 0 0 0 0 0 0 0 0 0 0 0
0 0 0 0 2 0 0 0 0 0 0 0 0
0 0 0 0 0 0 0 0
"Viruses;Uroviricota;Caudoviricetes;Caudoviricetes__na;Caudoviricetes__na;Caudoviricetes__na;Podoviridae_s
p._ctJaJ36" 0 0 0 0 0 0 0 0 0 0 0 0
0 0 0 0 0 0 0 0 0 0 0 0 0
0 0 0 0 0 1 8 0 0 0 0 7 2
0 0 0 1 0 0 0 0 0 0 1 0 0
0 0 0 2 2 0 0 0 0 0 0 0 0
0 0 2 0 0 0 0 0
"Viruses;Uroviricota;Caudoviricetes;Caudoviricetes__na;Caudoviricetes__na;Caudoviricetes__na;Podoviridae_s
p._ctJDl18" 0 0 0 0 0 0 0 0 0 0 0 0
0 0 0 0 0 0 0 0 0 0 0 0 0
0 0 0 0 0 0 0 0 0 0 0 0 0
0 0 0 0 0 0 0 0 0 0 0 0 0

0 0 0 1 0 0 0 0 0 0 0 0 0
0 0 0 0 0 0 0 0
"Viruses;Uroviricota;Caudoviricetes;Caudoviricetes__na;Caudoviricetes__na;Caudoviricetes__na;Podoviridae_s
p._ctjVy23" 0 0 0 0 0 0 0 0 0 0 0 0
0 0 0 0 0 0 0 0 0 0 0 0 0
0 0 0 0 0 0 0 0 0 0 0 0 0
0 0 0 0 0 0 0 0 0 0 0 0 0
0 0 0 1 0 0 0 0 0 0 0 0 0
0 0 0 0 0 0 0 0
"Viruses;Uroviricota;Caudoviricetes;Caudoviricetes__na;Caudoviricetes__na;Caudoviricetes__na;Podoviridae_s
p._ctK5Q1" 0 0 0 0 0 0 0 0 0 0 0 0
0 0 0 0 0 0 0 0 0 0 0 0 0
0 0 1 1 0 0 0 0 0 0 0 0 0
0 0 0 0 0 0 0 0 0 0 0 0 0
0 0 0 0 0 0 0 0 0 0 0 0 0
0 0 0 0 0 0 0 0
"Viruses;Uroviricota;Caudoviricetes;Caudoviricetes__na;Caudoviricetes__na;Caudoviricetes__na;Podoviridae_s
p._ctka020" 0 0 1 0 0 0 0 0 0 0 0 0
0 0 0 0 0 0 0 0 0 0 0 0 0
0 0 0 0 0 0 0 0 0 0 0 0 0
0 0 0 0 0 0 0 0 0 0 0 0 0
0 0 0 0 0 0 0 0 0 0 0 0 0
0 0 0 0 0 0 0 0
"Viruses;Uroviricota;Caudoviricetes;Caudoviricetes__na;Caudoviricetes__na;Caudoviricetes__na;Podoviridae_s
p._ctkBg18" 0 0 0 0 0 0 0 0 0 0 0 0
0 0 0 0 0 0 0 0 0 0 0 0 0
0 0 0 0 0 0 0 0 0 1 0 0 0
0 0 0 0 0 0 0 0 0 0 0 0 0
0 0 0 0 0 0 0 0 0 0 0 0 0
0 0 0 0 0 0 0 0
"Viruses;Uroviricota;Caudoviricetes;Caudoviricetes__na;Caudoviricetes__na;Caudoviricetes__na;Podoviridae_s
p._ctKmJ5" 0 0 0 0 0 0 0 0 0 0 0 0
0 0 0 0 0 0 1 2 0 0 0 2 0
0 1 0 0 0 0 0 0 0 0 0 0 0
0 0 0 0 0 0 0 0 0 0 0 0 0
0 0 0 0 0 0 0 0 0 0 0 0 0
0 0 0 0 0 0 0 0
"Viruses;Uroviricota;Caudoviricetes;Caudoviricetes__na;Caudoviricetes__na;Caudoviricetes__na;Podoviridae_s
p._ctLPy3" 0 0 0 0 0 0 0 0 0 0 0 0
0 0 0 0 0 0 0 0 0 0 0 0 0
0 0 0 0 0 0 0 0 0 0 0 0 2
0 0 0 0 0 0 0 0 0 0 0 0 0
0 1 0 0 0 0 0 0 0 0 0 0 0
0 0 0 0 0 0 0 0
"Viruses;Uroviricota;Caudoviricetes;Caudoviricetes__na;Caudoviricetes__na;Caudoviricetes__na;Podoviridae_s
p._ctn7K25" 0 0 0 0 0 0 0 0 0 0 0 0
0 0 0 0 0 0 1 1 0 0 0 0 0
0 1 0 0 0 0 0 0 0 0 0 0 0
0 0 0 0 0 0 0 0 0 0 0 0 0

0 0 0 0 0 0 0 0 0 0 0 0 0
0 0 0 0 0 0 0 0
"Viruses;Uroviricota;Caudoviricetes;Caudoviricetes__na;Caudoviricetes__na;Caudoviricetes__na;Podoviridae_sp._ctngc57" 0 0 0 0 0 0 0 0 0 0 0 0
0 0 0 0 0 0 0 0 0 0 0 0 0
0 0 0 0 0 1 0 0 0 0 0 0 0
0 0 0 0 0 0 0 0 0 0 0 0 0
0 0 0 0 0 0 0 0 0 0 0 0 0
0 0 0 0 0 0 0 0
"Viruses;Uroviricota;Caudoviricetes;Caudoviricetes__na;Caudoviricetes__na;Caudoviricetes__na;Podoviridae_sp._ctO1718" 0 0 0 0 0 0 0 0 0 0 0 0
0 0 0 0 0 0 0 0 0 0 0 0 0
0 0 0 0 0 0 0 0 0 0 0 1 0
0 0 0 5 0 0 0 0 0 0 0 0 0
0 0 0 2 0 0 0 0 0 0 0 0 0
0 0 0 0 0 0 0 0
"Viruses;Uroviricota;Caudoviricetes;Caudoviricetes__na;Caudoviricetes__na;Caudoviricetes__na;Podoviridae_sp._ctpVR23" 0 0 0 0 0 0 0 0 0 0 0 0
0 0 0 0 0 0 0 0 0 0 0 0 0
0 0 0 0 0 0 0 0 0 0 0 0 0
0 0 0 0 0 0 0 0 0 1 0 0 0
0 0 0 0 0 0 0 0 0 0 0 0 0
0 0 0 0 0 0 0 0
"Viruses;Uroviricota;Caudoviricetes;Caudoviricetes__na;Caudoviricetes__na;Caudoviricetes__na;Podoviridae_sp._ctQuf7" 0 0 0 0 0 0 0 0 0 0 0 0
0 0 0 0 0 0 0 0 0 0 0 0 0
0 0 0 0 0 0 0 0 0 0 0 0 0
0 0 0 0 0 0 0 0 0 0 0 0 0
0 0 0 0 0 0 0 0 0 9 0 0 0
0 0 0 0 0 0 0 0
"Viruses;Uroviricota;Caudoviricetes;Caudoviricetes__na;Caudoviricetes__na;Caudoviricetes__na;Podoviridae_sp._ctQyH19" 0 0 0 0 0 0 0 0 0 0 0 0
0 0 0 0 0 0 0 0 0 0 0 0 0
0 0 0 0 0 0 0 0 0 0 0 0 0
0 0 0 0 0 0 0 0 0 0 0 0 0
0 0 0 0 0 0 0 0 0 0 0 0 0
0 0 0 0 0 0 1 0
"Viruses;Uroviricota;Caudoviricetes;Caudoviricetes__na;Caudoviricetes__na;Caudoviricetes__na;Podoviridae_sp._ctrTa16" 0 0 0 0 4 11 0 0 0 0 0 0
0 0 0 0 0 0 0 0 0 0 0 0 0
0 0 0 0 0 0 0 0 0 0 0 0 0
0 0 0 0 0 0 0 0 0 1 0 0 0
0 0 0 0 0 0 0 0 0 0 0 0 0
0 0 0 0 0 0 0 0
"Viruses;Uroviricota;Caudoviricetes;Caudoviricetes__na;Caudoviricetes__na;Caudoviricetes__na;Podoviridae_sp._ctrTt13" 0 0 0 0 0 0 0 0 0 0 0 0
0 0 0 0 0 0 0 0 0 0 0 0 0
0 0 0 0 0 0 0 0 0 0 0 0 0
0 0 0 0 0 0 0 0 0 0 0 0 0

0	0	0	1	0	0	0	0	0	0	0	0	0
0	0	0	0	0	0	0	0
"Viruses;Uroviricota;Caudoviricetes;Caudoviricetes__na;Caudoviricetes__na;Caudoviricetes__na;Podoviridae_s
p._ctrub15"	0	0	0	0	0	0	0	0	0	0	0	0
0	0	0	0	0	0	0	0	0	0	0	0	0
0	0	0	0	0	0	0	0	0	0	0	0	0
0	0	0	1	0	0	0	0	0	0	0	0	0
0	0	0	0	0	0	0	0	0	15	4	0	0
0	0	0	0	0	0	0	0
"Viruses;Uroviricota;Caudoviricetes;Caudoviricetes__na;Caudoviricetes__na;Caudoviricetes__na;Podoviridae_s
p._ctsaV5"	0	0	0	0	0	0	0	0	0	0	0	0
0	0	0	0	0	0	0	0	0	0	0	0	0
0	0	0	0	0	1	0	0	0	0	0	0	0
0	0	0	0	0	0	0	0	0	0	0	0	0
0	0	0	0	0	0	0	0	0	0	0	0	0
0	0	0	0	0	0	0	0
"Viruses;Uroviricota;Caudoviricetes;Caudoviricetes__na;Caudoviricetes__na;Caudoviricetes__na;Podoviridae_s
p._ctU7u6"	0	0	0	0	0	0	0	0	0	0	0	0
0	0	1	0	0	0	0	0	0	0	0	0	0
0	0	0	0	0	0	0	0	0	0	0	0	0
0	0	0	0	0	0	0	0	0	0	2	1	0
0	0	0	0	0	0	0	0	0	0	0	0	0
0	0	0	1	0	0	0	0
"Viruses;Uroviricota;Caudoviricetes;Caudoviricetes__na;Caudoviricetes__na;Caudoviricetes__na;Podoviridae_s
p._ctUT63"	0	0	0	0	0	0	0	0	0	0	0	0
0	0	0	0	0	0	0	0	0	0	0	0	0
0	0	0	0	0	0	0	0	0	0	0	0	0
0	0	0	0	0	0	0	0	0	0	0	0	0
0	0	0	0	0	0	0	0	0	8	2	0	0
0	0	0	0	0	0	0	0
"Viruses;Uroviricota;Caudoviricetes;Caudoviricetes__na;Caudoviricetes__na;Caudoviricetes__na;Podoviridae_s
p._ctUYJ6"	0	0	0	0	0	0	0	0	0	0	0	0
0	0	0	0	0	0	0	0	0	0	0	0	0
0	0	0	0	0	0	0	0	0	0	0	0	0
0	0	0	0	0	0	0	0	0	0	0	0	0
0	0	0	0	0	0	0	0	0	0	1	0	0
0	0	0	0	0	0	0	0
"Viruses;Uroviricota;Caudoviricetes;Caudoviricetes__na;Caudoviricetes__na;Caudoviricetes__na;Podoviridae_s
p._ctW0z17"	0	0	0	0	0	0	0	0	0	0	0	0
1	1	0	0	0	0	0	1	0	0	0	0	0
0	0	0	0	0	0	0	0	0	0	0	0	0
0	0	0	0	1	3	0	0	0	0	2	2	0
0	0	0	0	0	0	0	0	0	9	2	0	0
0	0	0	1	0	0	0	0
"Viruses;Uroviricota;Caudoviricetes;Caudoviricetes__na;Caudoviricetes__na;Caudoviricetes__na;Podoviridae_s
p._ctWeH21"	0	0	0	0	0	0	0	0	0	0	0	0
0	0	0	0	0	0	0	0	0	0	0	0	0
0	0	0	0	0	0	0	0	0	0	0	0	0
0	0	0	0	0	0	0	0	0	0	0	0	0

0 0 0 1 3 0 0 0 0 0 0 0 0
0 0 9 13 0 0 0 0
"Viruses;Uroviricota;Caudoviricetes;Caudoviricetes__na;Caudoviricetes__na;Caudoviricetes__na;Podoviridae_s
p._ctwJH20" 0 0 0 0 0 0 0 0 0 0 0 0
0 0 0 0 0 0 0 0 0 0 0 0 0
0 0 0 0 0 0 0 0 0 0 0 0 0
0 0 0 0 0 0 0 0 0 3 0 0 0
0 0 0 0 0 0 0 0 0 0 0 0 0
0 0 0 0 0 0 0 0
"Viruses;Uroviricota;Caudoviricetes;Caudoviricetes__na;Caudoviricetes__na;Caudoviricetes__na;Podoviridae_s
p._ctx9R1" 0 0 0 0 0 0 0 0 0 0 0 0
0 0 0 0 0 0 0 0 0 0 0 0 0
0 0 0 0 0 0 0 0 0 0 0 0 0
0 0 0 0 0 0 0 0 0 0 0 0 0
0 0 0 1 0 0 0 0 0 0 0 0 0
0 0 0 0 0 0 0 0
"Viruses;Uroviricota;Caudoviricetes;Caudoviricetes__na;Caudoviricetes__na;Caudoviricetes__na;Podoviridae_s
p._ctXdu7" 1 0 0 0 0 1 0 0 0 0 0 0
7 8 33 37 1 5 0 0 0 0 0 0 0
0 0 0 0 0 0 0 0 0 0 0 0 0
0 0 0 0 0 0 0 0 0 1 7 1 0
0 0 0 0 0 0 0 2 0 0 0 0 0
0 0 0 0 0 0 0 0
"Viruses;Uroviricota;Caudoviricetes;Caudoviricetes__na;Caudoviricetes__na;Caudoviricetes__na;Podoviridae_s
p._ctxkP1" 0 0 1 0 0 0 0 0 0 0 0 0
0 0 0 0 0 0 0 0 0 0 0 0 0
0 0 0 0 0 0 0 0 0 0 0 0 0
0 0 0 0 0 0 0 0 0 0 0 0 0
0 0 0 0 0 0 0 0 0 0 0 0 0
0 0 0 0 0 0 0 0
"Viruses;Uroviricota;Caudoviricetes;Caudoviricetes__na;Caudoviricetes__na;Caudoviricetes__na;Podoviridae_s
p._ctxqo3" 0 0 0 0 0 0 0 0 0 0 0 0
1 0 0 0 0 0 0 0 0 0 0 0 0
0 0 0 0 0 0 0 0 0 0 0 0 0
0 0 0 0 0 0 0 0 0 0 0 0 0
0 0 0 0 0 0 0 0 0 0 0 0 0
0 0 2 2 0 0 0 0
"Viruses;Uroviricota;Caudoviricetes;Caudoviricetes__na;Caudoviricetes__na;Caudoviricetes__na;Podoviridae_s
p._ctY3D12" 0 0 0 0 0 0 0 0 0 0 0 0
0 0 0 0 0 0 0 0 0 0 0 0 0
0 0 0 0 0 0 0 0 0 0 0 0 4
3 0 0 0 0 0 0 0 0 0 0 0 0
0 11 0 0 0 0 0 0 0 0 0 0 0
0 0 0 0 0 0 0 0
"Viruses;Uroviricota;Caudoviricetes;Caudoviricetes__na;Caudoviricetes__na;Caudoviricetes__na;Podoviridae_s
p._cty5g4" 0 0 0 0 0 1 0 0 0 0 0 0
0 0 0 0 0 0 0 0 0 0 0 0 0
0 0 0 0 0 0 0 0 0 0 0 0 0
0 0 0 0 0 0 0 0 0 0 0 0 0

0 0 0 0 0 0 0 0 0 0 0 0 0
0 0 0 0 0 0 0 0
"Viruses;Uroviricota;Caudoviricetes;Caudoviricetes__na;Caudoviricetes__na;Caudoviricetes__na;Podoviridae_s
p._ctZ5d16" 0 0 0 0 0 0 0 0 0 0 0 0
0 1 0 0 0 0 0 0 0 0 0 0 0
0 0 0 0 0 0 0 0 0 0 0 0 0
0 0 0 0 0 0 0 0 0 0 0 0 0
0 0 0 1 0 0 0 0 0 0 0 0 0
0 0 0 0 0 0 0 0
"Viruses;Uroviricota;Caudoviricetes;Caudoviricetes__na;Caudoviricetes__na;Caudoviricetes__na;Podoviridae_s
p._ctz6O13" 0 0 0 0 0 0 0 0 0 0 0 0
0 0 1 0 0 0 0 0 0 0 0 0 0
0 0 0 0 0 0 0 0 0 0 0 0 0
0 0 2 0 0 0 3 2 0 0 0 0 0
0 0 0 0 0 0 0 0 0 0 0 0 0
0 0 0 0 0 0 0 0
"Viruses;Uroviricota;Caudoviricetes;Caudoviricetes__na;Caudoviricetes__na;Caudoviricetes__na;Podoviridae_s
p._ctzXp5" 0 0 0 0 0 0 0 0 0 0 0 0
1 1 0 0 0 0 0 0 0 0 0 0 0
0 0 0 0 0 0 0 0 0 0 0 0 0
0 0 0 0 0 0 0 0 0 0 0 0 0
0 0 0 0 0 0 0 0 0 0 0 0 0
0 0 0 0 0 0 0 0
"Viruses;Uroviricota;Caudoviricetes;Caudoviricetes__na;Caudoviricetes__na;Caudoviricetes__na;Podovirus_SO
G" 0 0 0 0 0 0 0 0 0 0 0 0 0
0 0 0 0 0 0 0 0 0 0 0 0 0
0 0 0 0 0 0 0 0 0 0 0 0 0
0 0 0 0 0 0 0 13 0 0 0 0 0
0 0 0 0 0 0 0 0 0 0 0 0 0
0 0 0 0 0 0 0
"Viruses;Uroviricota;Caudoviricetes;Caudoviricetes__na;Caudoviricetes__na;Caudoviricetes__na;Polaromonas_
phage_Tiera" 0 0 0 1 0 0 0 0 0 0 0 0
0 0 0 0 0 0 0 0 0 0 0 0 0
0 0 0 0 0 0 0 0 0 0 0 0 0
0 0 0 0 0 0 0 0 0 0 0 0 0
0 0 0 0 0 0 0 0 0 0 0 0 0
0 0 0 0 0 0 0 0
"Viruses;Uroviricota;Caudoviricetes;Caudoviricetes__na;Caudoviricetes__na;Caudoviricetes__na;Pontimonas_p
hage_phiPsal1" 0 0 0 0 0 11 0 0 0 0 0 0
0 0 0 0 0 0 0 0 0 0 0 0 0
0 0 0 0 0 0 0 0 0 0 0 0 0
0 0 0 0 0 0 0 0 0 0 0 0 0
0 0 0 0 0 0 0 0 0 0 0 0 0
0 0 0 0 0 0 0 0
"Viruses;Uroviricota;Caudoviricetes;Caudoviricetes__na;Caudoviricetes__na;Caudoviricetes__na;Proteus_phage
_vB_PmiP_RS51pmB" 0 0 0 0 0 0 0 0 0 0 0
0 0 0 0 0 0 0 0 0 0 0 0 0
0 0 0 0 0 0 0 0 0 0 0 0 0
0 0 0 0 0 0 0 0 0 0 0 0 0

0 0 0 0 0 0 0 0 0 0 0 0 0
0 0 0 0 0 0 0 1 0
"Viruses;Uroviricota;Caudoviricetes;Caudoviricetes__na;Caudoviricetes__na;Caudoviricetes__na;Providencia_p
hage_Kokobel2"0 0 1 0 0 0 0 0 0 0 0 0
0 0 0 0 0 0 0 0 0 0 0 0 0
0 0 0 0 0 0 0 0 0 0 0 0 0
0 0 0 0 0 0 0 0 0 0 0 0 0
0 0 0 0 0 0 0 0 0 0 0 0 0
0 0 0 0 0 0 0 0
"Viruses;Uroviricota;Caudoviricetes;Caudoviricetes__na;Caudoviricetes__na;Caudoviricetes__na;Pseudanabaen
a_phage_PA-SR01" 0 0 1 1 2 4 0 0 0 0 0
0 0 0 0 0 0 0 0 0 0 0 0 0
0 0 0 0 0 0 0 0 0 0 0 0 0
0 0 0 0 0 0 0 0 0 0 0 0 0
0 0 0 0 0 0 0 0 0 0 0 0 0
0 0 0 0 0 0 0 0 0
"Viruses;Uroviricota;Caudoviricetes;Caudoviricetes__na;Caudoviricetes__na;Caudoviricetes__na;Pseudanabaen
a_phage_Pam1"2 0 3 5 2 11 0 0 0 0 0 0
0 0 0 0 0 0 0 0 0 0 1 0 0
0 0 0 0 0 0 0 0 0 0 0 0 0
0 0 0 0 0 0 0 0 0 0 0 0 0
0 0 0 0 0 0 0 0 0 0 0 0 0
0 0 0 0 0 0 1 0
"Viruses;Uroviricota;Caudoviricetes;Caudoviricetes__na;Caudoviricetes__na;Caudoviricetes__na;Pseudanabaen
a_phage_Pam2"0 0 0 0 9 3 0 0 0 0 0 0
0 0 0 0 0 0 0 0 0 0 0 0 0
0 0 0 0 0 0 0 0 0 0 0 0 0
0 0 0 0 0 0 0 0 0 1 0 0 0
0 0 0 0 0 0 0 0 0 0 0 0 0
0 0 0 0 0 0 0 0
"Viruses;Uroviricota;Caudoviricetes;Caudoviricetes__na;Caudoviricetes__na;Caudoviricetes__na;Pseudanabaen
a_phage_Pam3"0 0 0 0 0 0 0 0 0 0 0 0
0 0 0 0 2 0 0 0 0 0 0 0 0
0 0 0 0 0 0 0 0 0 0 0 0 0
0 0 0 0 0 0 0 0 0 0 0 0 0
0 0 0 0 0 0 0 0 0 0 0 0 0
0 0 0 0 0 0 0 0
"Viruses;Uroviricota;Caudoviricetes;Caudoviricetes__na;Caudoviricetes__na;Caudoviricetes__na;Pseudanabaen
a_phage_Pam5"0 0 0 4 0 0 0 0 0 0 0 0
0 0 0 0 0 0 0 0 0 0 0 0 0
0 0 0 0 0 0 0 0 0 0 0 0 0
0 0 0 0 0 0 0 0 0 0 0 0 0
0 0 0 0 0 0 0 0 0 0 0 0 0
0 0 0 0 0 0 0 0
"Viruses;Uroviricota;Caudoviricetes;Caudoviricetes__na;Caudoviricetes__na;Caudoviricetes__na;Pseudoaltero
monas_phage_BS5" 0 0 1 0 0 0 0 0 0 0 0
0 0 0 0 0 0 0 0 0 0 0 0 0
0 0 0 0 0 0 0 0 0 0 0 0 0
0 0 0 0 0 0 0 0 0 0 0 0 0

0	0	0	0	0	0	0	0	0	0	0	0	0
0	0	0	0	0	0	0	0	0
"Viruses;Uroviricota;Caudoviricetes;Caudoviricetes__na;Caudoviricetes__na;Caudoviricetes__na;Pseudoaltero
monas_phage_C7"	0	0	0	1	0	0	0	0	0	0	0
0	0	0	0	0	0	0	0	0	0	0	0	0
0	0	0	0	0	0	0	0	0	0	0	0	0
0	0	0	0	0	0	0	0	0	0	0	0	0
0	0	0	0	0	0	0	0	0	0	0	0	0
0	0	0	0	0	0	0	0	0
"Viruses;Uroviricota;Caudoviricetes;Caudoviricetes__na;Caudoviricetes__na;Caudoviricetes__na;Pseudoaltero
monas_phage_H103"	0	0	0	0	10	10	0	0	0	0	0
0	0	0	0	0	0	0	0	0	0	0	0	0
0	0	0	0	0	0	0	0	0	0	0	0	0
0	0	0	0	0	0	0	0	0	0	0	0	0
0	0	0	0	0	0	0	0	0	0	0	0	0
0	0	0	0	0	0	0	0	0
"Viruses;Uroviricota;Caudoviricetes;Caudoviricetes__na;Caudoviricetes__na;Caudoviricetes__na;Pseudoaltero
monas_phage_PHS21"	0	0	1	1	1	0	0	0	0	0	0
0	0	0	0	0	0	0	0	0	0	0	0	0
0	0	0	0	0	0	0	0	0	0	0	0	0
0	0	0	0	0	0	0	0	0	0	0	0	0
0	0	0	0	0	0	0	0	0	0	0	0	0
0	0	0	0	0	0	0	0	0
"Viruses;Uroviricota;Caudoviricetes;Caudoviricetes__na;Caudoviricetes__na;Caudoviricetes__na;Pseudoaltero
monas_phage_XCL1123"	0	0	0	2	22	5	0	0	0	0
0	0	0	0	0	0	0	0	0	0	0	0	0
0	0	0	0	0	0	0	0	0	0	0	0	0
0	0	0	0	0	0	0	0	0	0	28	2	0
0	0	2	0	0	0	0	0	0	0	0	0	0
0	0	0	0	0	0	0	0	0	0
"Viruses;Uroviricota;Caudoviricetes;Caudoviricetes__na;Caudoviricetes__na;Caudoviricetes__na;Pseudomonas
_phage_201phi2-1"	0	0	0	0	0	0	0	0	0	0	0
0	0	0	0	0	0	0	0	0	0	0	0	0
0	0	0	0	0	0	0	0	2	0	0	0	0
0	0	0	0	0	0	0	0	0	0	0	0	0
0	0	0	0	0	0	0	0	0	0	0	0	0
0	0	0	0	0	0	0	0	0
"Viruses;Uroviricota;Caudoviricetes;Caudoviricetes__na;Caudoviricetes__na;Caudoviricetes__na;Pseudomonas
_phage_8P"	1	0	0	0	0	0	0	0	0	0	0	0
0	0	0	0	0	0	0	0	0	0	0	0	0
0	0	0	0	0	0	0	0	0	0	0	0	0
0	0	0	0	0	0	0	0	0	0	0	0	0
0	0	0	0	0	0	0	0	0	0	0	0	0
0	0	0	0	0	0	0	0
"Viruses;Uroviricota;Caudoviricetes;Caudoviricetes__na;Caudoviricetes__na;Caudoviricetes__na;Pseudomonas
_phage_Amjad_SA"	0	1	1	0	1	6	0	0	0	0	0
0	0	0	0	0	0	0	0	0	0	0	0	0
0	0	0	0	0	0	0	0	0	0	0	0	0
0	0	0	0	0	0	0	0	0	0	3	0	0

0 0 0 0 0 0 0 0 0 0 0 0 0
0 0 0 0 0 0 0 0 0
"Viruses;Uroviricota;Caudoviricetes;Caudoviricetes__na;Caudoviricetes__na;Caudoviricetes__na;Pseudomonas_phage_HU1" 0 0 0 1 7 0 0 0 0 0 0 0
0 0 0 0 0 0 0 0 0 0 0 0 0
0 0 0 0 0 0 0 0 0 0 0 0 0
0 0 0 0 0 0 0 0 0 0 0 0 0
0 0 0 0 0 0 0 0 0 0 0 0 0
0 0 0 0 0 0 0 0
"Viruses;Uroviricota;Caudoviricetes;Caudoviricetes__na;Caudoviricetes__na;Caudoviricetes__na;Pseudomonas_phage_MR14" 0 0 1 1 0 1 0 0 0 0 0 0
0 0 0 0 0 0 0 0 0 0 0 0 0
0 0 0 0 0 0 0 0 0 0 0 0 0
0 0 0 0 0 0 0 0 0 0 0 0 0
0 0 0 0 0 0 0 0 0 0 0 0 0
0 0 0 0 0 0 0 0
"Viruses;Uroviricota;Caudoviricetes;Caudoviricetes__na;Caudoviricetes__na;Caudoviricetes__na;Pseudomonas_phage_phiK7A1" 1 0 0 2 0 0 0 60 0 0 0
0 0 0 0 0 0 0 0 0 0 0 0 0
0 0 0 0 0 0 0 0 0 0 0 0 0
0 0 0 0 0 0 0 0 0 0 0 0 0
0 0 0 0 0 0 0 0 0 0 0 0 0
0 0 0 0 0 0 0 0 0
"Viruses;Uroviricota;Caudoviricetes;Caudoviricetes__na;Caudoviricetes__na;Caudoviricetes__na;Pseudomonas_phage_Skulduggery" 1 0 0 0 0 0 0 0 0 0 0
0 0 0 0 0 0 0 0 0 0 0 0 0
0 0 0 0 0 0 0 0 0 0 0 0 0
0 0 0 0 0 0 0 0 0 0 0 0 0
0 0 0 0 0 0 0 0 0 0 0 0 0
0 0 0 0 0 0 0 0 0
"Viruses;Uroviricota;Caudoviricetes;Caudoviricetes__na;Caudoviricetes__na;Caudoviricetes__na;Pseudomonas_phage_UAVern" 0 0 1 1 0 0 0 0 0 0 0
0 0 0 0 0 0 0 0 0 0 0 0 0
0 0 0 0 0 0 0 0 0 0 0 0 0
0 0 0 0 0 0 0 0 0 0 0 0 0
0 0 0 0 0 0 0 0 0 0 0 0 0
0 0 0 0 0 0 0 0 0
"Viruses;Uroviricota;Caudoviricetes;Caudoviricetes__na;Caudoviricetes__na;Caudoviricetes__na;Pseudomonas_phage_vB_PaeM_USP_25" 0 0 0 1 0 0 0 0 0 0
0 0 0 0 0 0 0 0 0 0 0 0 0
0 0 0 0 0 0 0 0 0 0 0 0 0
0 0 0 0 0 0 0 0 0 0 0 0 0
0 0 0 0 0 0 0 0 0 0 0 0 0
0 0 0 0 0 0 0 0 0 0
"Viruses;Uroviricota;Caudoviricetes;Caudoviricetes__na;Caudoviricetes__na;Caudoviricetes__na;Pseudomonas_phage_vB_PcuM__KLEP17-4" 0 0 1 0 1 0 0 0 0 0
0 0 0 0 0 0 0 0 0 0 0 0 0
0 0 0 0 0 0 0 0 0 0 0 0 0
0 0 0 0 0 0 0 0 0 0 0 0 0

0 0 0 0 0 0 0 0 0 0 0 0 0
0 0 0 0 0 0 0 0 0 0
"Viruses;Uroviricota;Caudoviricetes;Caudoviricetes__na;Caudoviricetes__na;Caudoviricetes__na;Pseudomonas_phage_vB_PpS_SYP" 0 0 0 1 0 1 0 0 0 0 0
0 0 0 0 0 0 0 0 0 0 0 0 0
0 0 0 0 0 0 0 0 0 0 0 0 0
0 0 0 0 0 0 0 0 0 0 0 0 0
0 0 0 0 0 0 0 0 0 0 0 0 0
0 0 0 0 0 0 0 0 0
"Viruses;Uroviricota;Caudoviricetes;Caudoviricetes__na;Caudoviricetes__na;Caudoviricetes__na;Pseudomonas_phage_vB_PsaM_M1" 0 1 1 0 18 2 0 0 0 0 0
0 0 0 0 0 0 0 0 0 0 0 0 0
0 0 0 0 0 0 0 0 0 0 0 0 0
0 0 0 0 0 0 0 0 0 0 6 0 0
0 0 1 0 0 0 0 0 0 0 0 0 0
0 0 0 0 0 0 0 1 0
"Viruses;Uroviricota;Caudoviricetes;Caudoviricetes__na;Caudoviricetes__na;Caudoviricetes__na;Pseudomonas_phage_VW-6B" 0 0 0 1 0 0 0 0 0 0 0
0 0 0 0 0 0 0 0 0 0 0 0 0
0 0 0 0 0 0 0 0 0 0 0 0 0
0 0 0 0 0 0 0 0 0 0 0 0 0
0 0 0 0 0 0 0 0 0 0 0 0 0
0 0 0 0 0 0 0 0 0
"Viruses;Uroviricota;Caudoviricetes;Caudoviricetes__na;Caudoviricetes__na;Caudoviricetes__na;Pseudoxanthomonas_phage_PW916" 0 0 0 0 0 0 0 0 1 0 0
0 0 0 0 0 0 0 0 0 0 0 0 0
0 0 0 0 0 0 0 0 0 0 0 0 0
0 0 0 0 0 0 0 0 0 0 0 0 0
0 0 0 0 0 0 0 0 0 0 0 0 0
0 0 0 0 0 0 0 0 0
"Viruses;Uroviricota;Caudoviricetes;Caudoviricetes__na;Caudoviricetes__na;Caudoviricetes__na;Psychrobacillus_phage_Perkons" 0 0 0 0 0 0 0 0 0 0 0
0 0 0 0 0 0 0 0 0 0 0 0 0
0 0 0 0 0 0 0 0 0 0 0 0 0
0 0 0 0 0 0 0 0 0 0 0 0 0
0 0 0 0 0 0 0 0 0 0 2 1 0
0 0 0 0 0 0 0 0 0
"Viruses;Uroviricota;Caudoviricetes;Caudoviricetes__na;Caudoviricetes__na;Caudoviricetes__na;Psychrobacter_phage_D'Alembert" 0 0 0 0 0 1 0 0 0 0 0
0 0 0 0 0 0 0 0 0 0 0 0 0
0 0 0 0 0 0 0 0 0 0 0 2 0
0 0 0 1 0 0 0 0 0 0 0 0 0
0 0 0 0 0 0 0 0 0 0 0 0 0
0 0 0 0 0 0 0 0 0
"Viruses;Uroviricota;Caudoviricetes;Caudoviricetes__na;Caudoviricetes__na;Caudoviricetes__na;Psychrobacter_phage_pOW20-A" 1 0 0 0 1 0 0 0 0 0 0
0 0 0 0 0 0 0 0 0 0 0 0 0
0 0 0 0 0 0 0 0 0 0 0 0 0
0 0 0 0 0 0 0 0 0 0 0 0 0

0 0 0 0 0 0 0 0 0 8 0 0 0
0 0 0 0 0 0 0 0 0
"Viruses;Uroviricota;Caudoviricetes;Caudoviricetes__na;Caudoviricetes__na;Caudoviricetes__na;Psychrobacter_phage_vB_PmaS_Y8A"0 0 0 1 0 11 0 0 0 0 0
0 0 0 0 0 0 1 0 0 0 0 0 0
0 0 0 0 0 0 0 0 0 0 0 0 0
0 0 0 0 0 0 0 0 0 0 0 0 0
0 0 0 0 0 0 0 0 0 0 0 0 0
0 0 0 0 0 0 0 0 0
"Viruses;Uroviricota;Caudoviricetes;Caudoviricetes__na;Caudoviricetes__na;Caudoviricetes__na;Puniceispirillum_phage_HMO-2011" 0 0 0 1 1 2 0 0 0 0 0
0 0 0 0 0 0 1 0 0 0 0 0 0
0 0 0 0 0 0 0 0 0 0 0 0 0
0 0 0 0 0 0 0 0 0 0 4 0 0
0 0 0 0 0 0 0 0 0 0 0 0 0
0 0 0 0 0 0 0 0 0
"Viruses;Uroviricota;Caudoviricetes;Caudoviricetes__na;Caudoviricetes__na;Caudoviricetes__na;Ralstonia_phage_UAM5" 0 0 2 1 0 28 0 0 0 0 0 0
0 0 0 0 0 0 0 0 0 0 0 0 0
0 0 0 0 0 0 0 0 0 0 0 0 0
0 0 0 0 0 0 0 0 0 0 0 0 0
0 0 0 0 0 0 0 0 0 0 0 0 0
0 0 0 0 0 0 2 0
"Viruses;Uroviricota;Caudoviricetes;Caudoviricetes__na;Caudoviricetes__na;Caudoviricetes__na;Rhizobium_phage_16-3" 0 0 1 0 0 0 0 0 0 0 0 0
0 0 0 0 0 0 0 0 0 0 0 0 0
0 0 0 0 0 0 0 0 0 0 0 0 0
0 0 0 0 0 0 0 0 0 0 0 0 0
0 0 0 0 0 0 0 0 0 0 0 0 0
0 0 0 0 0 0 0 0
"Viruses;Uroviricota;Caudoviricetes;Caudoviricetes__na;Caudoviricetes__na;Caudoviricetes__na;Rhizobium_phage_RHph_I1_18" 0 0 0 0 0 0 0 0 0 0 0
0 0 0 0 0 0 0 0 0 0 0 1 0
0 0 0 0 0 0 0 0 0 0 0 0 0
0 0 0 0 0 0 0 0 0 0 0 0 0
0 0 0 0 0 0 0 0 0 0 0 0 0
0 0 0 0 0 0 0 0 0
"Viruses;Uroviricota;Caudoviricetes;Caudoviricetes__na;Caudoviricetes__na;Caudoviricetes__na;Rhizobium_phage_RHph_I3_11" 0 0 1 0 0 8 0 0 0 0 0
0 0 0 0 0 0 0 0 0 0 0 0 0
0 0 0 0 0 0 0 0 0 0 0 0 0
0 0 0 0 0 0 0 0 0 0 0 0 0
0 0 0 0 0 0 0 0 0 0 0 0 0
0 0 0 0 0 0 0 0 0
"Viruses;Uroviricota;Caudoviricetes;Caudoviricetes__na;Caudoviricetes__na;Caudoviricetes__na;Rhizobium_phage_RHph_N3_19" 0 0 0 0 0 0 0 0 0 0 0
0 0 0 0 0 0 0 0 0 0 0 0 0
0 0 0 0 0 0 0 0 0 0 0 0 0
0 0 0 0 0 0 0 0 0 0 1 0 0

0 0 0 0 0 0 0 0 0 0 0 0 0
0 0 0 0 0 0 0 0 0
"Viruses;Uroviricota;Caudoviricetes;Caudoviricetes__na;Caudoviricetes__na;Caudoviricetes__na;Rhizobium_ph
age_RHph_N3_2" 0 0 0 1 0 0 0 0 0 0 0
0 0 0 0 0 0 0 0 0 0 0 0 0
0 0 0 0 0 0 0 0 0 0 0 0 0
0 0 0 0 0 0 0 0 0 0 0 0 0
0 0 0 0 0 0 0 0 0 0 0 0 0
0 0 0 0 0 0 0 0 0
"Viruses;Uroviricota;Caudoviricetes;Caudoviricetes__na;Caudoviricetes__na;Caudoviricetes__na;Rhizobium_ph
age_RHph_N65" 0 1 1 0 0 1 0 0 0 0 0
0 0 0 0 0 0 0 0 0 0 0 0 0
0 0 0 0 0 0 0 0 0 0 0 0 0
0 0 0 0 0 0 0 0 0 0 0 0 0
0 0 0 0 0 0 0 0 0 0 0 0 0
0 0 0 0 0 0 0 0 0
"Viruses;Uroviricota;Caudoviricetes;Caudoviricetes__na;Caudoviricetes__na;Caudoviricetes__na;Rhizobium_ph
age_RHph_TM16" 0 0 0 0 0 0 0 0 0 0 0
0 0 0 0 0 0 0 0 0 0 0 0 0
0 0 0 0 0 0 0 0 0 0 0 2 0
0 0 0 0 0 0 0 0 0 0 0 0 0
0 0 0 0 0 0 0 0 0 0 0 0 0
0 0 0 0 0 0 0 0 0
"Viruses;Uroviricota;Caudoviricetes;Caudoviricetes__na;Caudoviricetes__na;Caudoviricetes__na;Rhizobium_ph
age_RHph_Y1_10" 0 0 1 0 0 0 0 0 0 0 0
0 0 0 0 0 0 0 0 0 0 0 0 0
0 0 0 0 0 0 0 0 0 0 0 0 0
0 0 0 0 0 0 0 0 0 0 0 0 0
0 0 0 0 0 0 0 0 0 0 0 0 0
0 0 0 0 0 0 0 0 0
"Viruses;Uroviricota;Caudoviricetes;Caudoviricetes__na;Caudoviricetes__na;Caudoviricetes__na;Rhizobium_ph
age_RHph_Y60" 0 0 0 1 0 0 0 0 0 0 0
0 0 0 0 0 0 0 0 0 0 0 0 0
0 0 0 0 0 0 0 0 0 0 0 0 0
0 0 0 0 0 0 0 0 0 0 0 0 0
0 0 0 0 0 0 0 0 0 0 0 0 0
0 0 0 0 0 0 0 0 0
"Viruses;Uroviricota;Caudoviricetes;Caudoviricetes__na;Caudoviricetes__na;Caudoviricetes__na;Rhodobacter_
phage_RcKemmy" 0 0 0 1 0 0 0 0 0 0 0
0 0 0 0 0 0 0 0 0 0 0 0 0
0 0 0 0 0 0 0 0 0 0 0 0 0
0 0 0 0 0 0 0 4 0 0 0 0 0
0 0 0 0 0 0 0 0 0 0 0 0 0
0 0 0 0 0 0 0 0 0
"Viruses;Uroviricota;Caudoviricetes;Caudoviricetes__na;Caudoviricetes__na;Caudoviricetes__na;Rhodobacter_
phage_RcSimone-Hastad" 0 0 0 0 0 2 0 0 0 0
0 0 0 0 0 0 0 0 0 0 0 0 0
0 0 0 0 0 0 0 0 0 0 0 0 0
0 0 0 1 0 0 0 0 0 0 0 0 0

0 0 0 0 0 0 0 0 0 0 0 0 0
0 0 0 0 0 0 0 0 0 0
"Viruses;Uroviricota;Caudoviricetes;Caudoviricetes__na;Caudoviricetes__na;Caudoviricetes__na;Rhodobacter_phage_RcZahn" 0 0 0 1 0 0 0 0 0 0 0 0
0 0 0 0 0 0 0 0 0 0 0 0 0
0 0 0 0 0 0 0 0 0 0 0 0 0
0 0 0 0 0 0 0 0 0 0 0 0 0
0 0 0 0 0 0 0 0 0 0 0 0 0
0 0 0 0 0 0 0 0
"Viruses;Uroviricota;Caudoviricetes;Caudoviricetes__na;Caudoviricetes__na;Caudoviricetes__na;Rhodococcus_phage_Mbo2" 0 0 0 0 0 6 0 0 0 0 0 0
0 0 0 0 0 0 0 0 0 0 0 0 0
0 0 0 0 0 0 0 0 0 0 0 0 0
0 0 0 0 0 0 0 0 0 0 0 0 0
0 0 0 0 0 0 0 0 0 0 0 0 0
0 0 0 0 0 0 0 0
"Viruses;Uroviricota;Caudoviricetes;Caudoviricetes__na;Caudoviricetes__na;Caudoviricetes__na;Rhodoferax_phage_P26218" 1 1 6 11 13 36 0 0 0 0 0 0
0 0 0 1 0 0 0 0 0 0 0 0 0
0 0 0 0 0 0 0 0 1 0 0 0 0
0 0 0 54 0 0 0 0 0 0 0 0 0
0 0 0 0 0 0 0 0 0 0 0 0 0
0 0 0 0 0 0 0 0
"Viruses;Uroviricota;Caudoviricetes;Caudoviricetes__na;Caudoviricetes__na;Caudoviricetes__na;Roseobacter_phage_CRP-1" 0 0 1 3 9 1 0 0 0 0 0 0
0 0 0 0 0 0 0 0 0 0 0 0 0
0 0 0 0 0 0 0 0 0 0 0 0 0
0 0 0 3 0 0 0 0 0 2 0 0 0
0 0 0 0 0 0 0 0 0 0 0 0 0
0 0 0 0 0 0 0 0
"Viruses;Uroviricota;Caudoviricetes;Caudoviricetes__na;Caudoviricetes__na;Caudoviricetes__na;Roseobacter_phage_CRP-13" 0 0 0 0 11 4 0 0 0 0 0 0
0 0 0 0 0 0 0 0 0 0 0 1 0
0 0 0 0 0 0 0 0 0 0 0 0 0
0 0 0 0 0 0 0 0 0 0 0 0 0
0 0 0 0 0 0 0 0 0 0 0 0 0
0 0 0 0 0 0 2 0
"Viruses;Uroviricota;Caudoviricetes;Caudoviricetes__na;Caudoviricetes__na;Caudoviricetes__na;Roseobacter_phage_CRP-2" 1 0 0 0 54 3 0 0 0 0 0 0
0 0 0 0 0 0 0 0 0 0 0 0 0
0 0 0 0 0 0 0 0 0 0 0 0 0
0 0 0 0 0 0 0 0 0 0 0 0 0
0 0 0 0 0 0 0 0 0 0 0 0 0
0 0 0 0 0 0 0 0
"Viruses;Uroviricota;Caudoviricetes;Caudoviricetes__na;Caudoviricetes__na;Caudoviricetes__na;Roseobacter_phage_CRP-207" 0 0 1 0 0 12 0 0 0 0 0 0
0 0 0 0 0 0 0 0 0 0 0 0 0
0 0 0 0 0 0 0 0 0 0 0 0 0
0 0 0 0 0 0 0 1 0 0 0 0 0

0 0 0 0 0 0 0 0 0 0 0 0 0
0 0 0 0 0 0 2 0
"Viruses;Uroviricota;Caudoviricetes;Caudoviricetes__na;Caudoviricetes__na;Caudoviricetes__na;Roseobacter_phage_CRP-212" 0 0 0 4 5 0 0 0 0 0 0 0
0 0 0 0 0 0 0 0 0 0 0 0 0
0 0 0 0 0 0 0 0 0 0 0 0 0
0 0 0 0 0 0 0 0 0 2 0 0 0
0 0 0 0 0 0 0 0 0 0 0 0 0
0 0 0 0 0 0 1 0
"Viruses;Uroviricota;Caudoviricetes;Caudoviricetes__na;Caudoviricetes__na;Caudoviricetes__na;Roseobacter_phage_CRP-235" 0 0 1 1 0 0 0 0 0 0 0 0
0 0 0 0 0 0 0 0 0 0 0 0 0
0 0 0 0 0 0 0 0 0 0 0 0 0
0 0 0 0 0 0 0 0 0 1 0 0 0
0 0 0 0 0 0 0 0 0 0 0 0 0
0 0 0 0 0 0 0 0
"Viruses;Uroviricota;Caudoviricetes;Caudoviricetes__na;Caudoviricetes__na;Caudoviricetes__na;Roseobacter_phage_CRP-3" 0 3 0 0 4 4 0 0 0 0 0 0
0 0 0 0 0 0 0 0 0 0 0 0 0
0 0 0 0 0 0 0 0 0 0 0 0 0
0 0 0 0 0 0 0 0 0 0 0 0 0
0 0 0 0 0 0 0 0 0 0 0 0 0
0 0 0 0 0 0 0 0
"Viruses;Uroviricota;Caudoviricetes;Caudoviricetes__na;Caudoviricetes__na;Caudoviricetes__na;Roseobacter_phage_CRP-345" 0 0 0 0 0 19 0 0 0 0 0 0
0 0 0 0 0 0 0 0 0 0 0 0 0
0 0 0 0 0 0 0 0 0 0 0 0 0
0 0 0 0 0 0 0 0 0 0 0 0 0
0 0 0 0 0 0 0 0 0 0 0 0 0
0 0 0 0 0 0 0 0
"Viruses;Uroviricota;Caudoviricetes;Caudoviricetes__na;Caudoviricetes__na;Caudoviricetes__na;Roseobacter_phage_CRP-6" 0 0 0 1 4 4 0 0 0 0 0 0
0 0 0 0 1 0 0 0 0 0 0 1 0
0 0 0 0 0 0 0 0 0 1 0 0 0
0 0 0 0 0 0 0 0 0 0 0 0 0
0 0 0 0 0 0 0 0 0 0 0 0 0
0 0 0 0 0 0 0 0
"Viruses;Uroviricota;Caudoviricetes;Caudoviricetes__na;Caudoviricetes__na;Caudoviricetes__na;Roseobacter_phage_CRP-603" 0 0 1 0 71 0 0 0 0 0 0 0
0 0 0 0 0 0 0 0 0 0 0 0 0
0 0 0 0 0 0 0 0 0 0 0 0 0
0 0 0 0 0 0 0 0 0 2 0 0 0
0 0 0 0 0 0 0 0 0 0 0 0 0
0 0 0 0 0 0 0 0
"Viruses;Uroviricota;Caudoviricetes;Caudoviricetes__na;Caudoviricetes__na;Caudoviricetes__na;Roseobacter_phage_CRP-7" 0 0 0 0 0 5 0 0 0 0 0 0
0 0 0 0 0 0 0 0 0 0 0 0 0
0 0 0 0 0 0 0 0 0 0 0 0 0
0 0 0 0 0 0 0 0 0 3 0 0 0

0 0 0 0 0 0 0 0 0 0 0 0 0
0 0 0 0 0 0 1 0
"Viruses;Uroviricota;Caudoviricetes;Caudoviricetes__na;Caudoviricetes__na;Caudoviricetes__na;Roseobacter_phage_CRP-738" 0 0 0 1 13 2 0 0 0 0 0 0
0 0 0 0 0 0 0 0 0 0 0 1 0
0 0 0 0 0 0 0 0 0 0 0 0 0
0 0 0 0 0 0 0 0 0 0 0 0 0
0 0 0 0 0 0 0 0 0 0 0 0 0
0 0 0 0 0 0 0 0
"Viruses;Uroviricota;Caudoviricetes;Caudoviricetes__na;Caudoviricetes__na;Caudoviricetes__na;Roseobacter_phage_CRP-9" 2 0 0 5 22 4 0 0 0 0 0 0
0 0 0 0 0 0 0 0 0 0 0 0 0
0 0 0 0 0 0 0 0 0 0 0 0 0
0 0 0 0 0 0 0 0 0 0 0 0 0
0 0 0 0 0 0 0 0 0 0 0 0 0
0 0 0 0 0 0 3 0
"Viruses;Uroviricota;Caudoviricetes;Caudoviricetes__na;Caudoviricetes__na;Caudoviricetes__na;Roseobacter_phage_CRP-901" 0 0 0 0 1 0 0 0 0 0 0 0
0 0 0 0 0 0 0 0 0 0 0 0 0
0 0 0 0 0 0 0 0 0 0 0 0 0
0 0 0 0 0 0 0 0 0 0 0 0 0
0 0 0 0 0 0 0 0 0 0 0 0 0
0 0 0 0 0 0 0 0
"Viruses;Uroviricota;Caudoviricetes;Caudoviricetes__na;Caudoviricetes__na;Caudoviricetes__na;Roseobacter_phage_CRP-902" 0 0 0 1 0 0 0 0 0 0 0 0
0 0 0 0 0 0 0 0 0 0 0 0 0
0 0 0 0 0 0 0 0 0 0 0 0 0
0 0 0 0 0 0 0 0 0 0 0 0 0
0 0 1 0 0 0 0 0 0 0 0 0 0
0 0 0 0 0 0 0 0
"Viruses;Uroviricota;Caudoviricetes;Caudoviricetes__na;Caudoviricetes__na;Caudoviricetes__na;Roseobacter_phage_RDJL3" 0 0 0 0 0 0 0 0 0 0 0 0
0 0 0 0 0 0 0 0 0 0 0 0 0
0 0 0 0 0 0 0 0 0 0 0 0 0
0 0 0 0 0 0 0 0 27 0 0 0 0
0 0 0 0 0 0 0 0 0 0 0 0 0
0 0 0 0 0 0 0 0
"Viruses;Uroviricota;Caudoviricetes;Caudoviricetes__na;Caudoviricetes__na;Caudoviricetes__na;Ruminococcus_phage_phiRg519T2" 0 0 0 0 0 0 0 0 0 0 0
0 0 0 0 0 0 0 0 0 0 0 0 0
0 0 0 0 0 0 0 0 0 0 0 0 0
0 0 0 0 0 0 0 1 0 0 0 0 0
0 0 0 0 0 0 0 0 0 0 0 0 0
0 0 0 0 0 0 0 0 0
"Viruses;Uroviricota;Caudoviricetes;Caudoviricetes__na;Caudoviricetes__na;Caudoviricetes__na;Ruminococcus_phage_phiRM10" 0 0 0 0 0 0 0 0 0 0 0
0 0 0 0 0 0 0 0 0 0 0 0 0
0 0 0 0 0 0 0 0 0 0 0 0 0
0 0 0 0 5 0 0 0 0 0 0 0 0

0 0 0 0 0 0 0 0 0 0 0 0 0
0 0 0 0 0 0 0 1 0
"Viruses;Uroviricota;Caudoviricetes;Caudoviricetes__na;Caudoviricetes__na;Caudoviricetes__na;Salicola_phage
_CGphi29" 0 0 0 0 9 0 0 0 0 0 0 0
0 0 0 0 0 0 0 0 0 0 0 0 0
0 0 0 0 0 0 0 0 0 0 0 0 0
0 0 0 0 0 0 0 0 0 1 0 0 0
0 0 0 0 0 0 0 0 0 0 0 0 0
0 0 0 0 0 0 0 0
"Viruses;Uroviricota;Caudoviricetes;Caudoviricetes__na;Caudoviricetes__na;Caudoviricetes__na;Salicola_phage
_SCTP-2" 0 0 1 0 2 0 0 0 0 0 0 0
0 0 0 0 0 0 0 0 0 0 0 0 0
0 0 0 0 0 0 0 0 0 0 0 0 0
0 0 0 0 0 0 0 0 0 0 0 0 0
0 0 0 0 0 0 0 0 0 0 0 0 0
0 0 0 0 0 0 0 0
"Viruses;Uroviricota;Caudoviricetes;Caudoviricetes__na;Caudoviricetes__na;Caudoviricetes__na;Salmonella_ph
age_pSal-SNUABM-01" 0 0 0 0 0 8 0 0 0 0 0
0 0 0 0 0 0 0 0 0 0 0 0 0
0 0 0 0 0 0 0 0 0 0 0 0 0
0 0 0 0 0 0 0 0 0 0 0 0 0
0 0 0 0 0 0 0 0 0 0 0 0 0
0 0 0 0 0 0 0 0 0
"Viruses;Uroviricota;Caudoviricetes;Caudoviricetes__na;Caudoviricetes__na;Caudoviricetes__na;Salmonella_ph
age_SSBI34" 0 0 0 0 0 0 0 0 0 0 0 0
0 0 0 0 0 0 0 0 0 0 0 0 0
0 0 0 0 0 0 0 0 0 0 0 0 0
0 0 0 2 0 0 0 0 0 2 0 0 0
0 0 0 0 0 0 0 0 0 0 0 0 0
0 0 0 0 0 0 0 0
"Viruses;Uroviricota;Caudoviricetes;Caudoviricetes__na;Caudoviricetes__na;Caudoviricetes__na;Salmonella_ph
age_vB_SalM_SA002" 0 0 0 0 4 0 0 0 0 0 0
0 0 0 0 0 0 0 0 0 0 0 0 0
0 0 0 0 0 0 0 0 0 0 0 0 0
0 0 0 0 0 0 0 0 0 0 0 0 0
0 0 0 0 0 0 0 0 0 0 0 0 0
0 0 0 0 0 0 0 0 0
"Viruses;Uroviricota;Caudoviricetes;Caudoviricetes__na;Caudoviricetes__na;Caudoviricetes__na;Serratia_phage
_MQ-4" 0 0 0 0 11 0 0 0 0 0 0 0 0
0 0 0 0 0 0 0 0 0 0 0 0 0
0 0 0 0 0 0 0 0 0 0 0 0 0
0 0 0 0 0 0 0 0 0 0 0 0 0
0 0 0 0 0 0 0 0 0 0 0 0 0
0 0 0 0 0 0 0
"Viruses;Uroviricota;Caudoviricetes;Caudoviricetes__na;Caudoviricetes__na;Caudoviricetes__na;Serratia_phage
_vB_SmaM-ChibiTotoro" 2 0 0 0 0 0 0 0 0 0
0 0 0 0 0 0 0 0 0 0 0 0 0
0 0 0 0 0 0 0 0 0 0 0 0 0
0 0 0 0 0 0 0 0 0 0 0 0 0

0 0 0 0 0 0 0 0 0 0 0 0 0
0 0 0 0 0 0 0 0 0 0
"Viruses;Uroviricota;Caudoviricetes;Caudoviricetes__na;Caudoviricetes__na;Caudoviricetes__na;Shewanella_p
hage_SppYZU01" 0 0 1 0 0 26 0 0 0 0 0
0 0 0 0 0 0 0 0 0 0 0 0 0
0 0 0 0 0 0 0 0 0 0 0 0 0
0 0 0 0 0 0 0 0 0 0 0 0 0
0 0 0 0 0 0 0 0 0 0 0 0 0
0 0 0 0 0 0 0 0 0
"Viruses;Uroviricota;Caudoviricetes;Caudoviricetes__na;Caudoviricetes__na;Caudoviricetes__na;Shewanella_sp
._phage_1/4" 0 0 0 0 0 4 0 0 0 0 0 0
0 0 0 0 0 0 0 0 0 0 0 0 0
0 0 0 0 0 0 0 0 0 0 0 0 0
0 0 0 0 0 0 0 0 0 0 0 0 0
0 0 0 0 0 0 0 0 0 0 0 0 0
0 0 0 0 0 0 0 0
"Viruses;Uroviricota;Caudoviricetes;Caudoviricetes__na;Caudoviricetes__na;Caudoviricetes__na;Shewanella_sp
._phage_1/40" 0 0 0 0 0 1 0 0 0 0 0 0
0 0 0 0 0 0 0 0 0 0 0 0 0
0 0 0 0 0 0 0 0 0 0 0 0 0
0 0 0 0 0 0 0 0 0 0 0 0 0
0 0 0 0 0 0 0 0 0 0 0 0 0
0 0 0 0 0 0 0 0
"Viruses;Uroviricota;Caudoviricetes;Caudoviricetes__na;Caudoviricetes__na;Caudoviricetes__na;Shewanella_sp
._phage_1/41" 0 0 0 0 0 0 0 0 0 0 0 0
0 0 0 0 2 0 0 0 0 0 0 0 0
0 0 0 0 0 0 0 0 0 0 0 0 0
0 0 0 0 0 0 0 0 0 1 0 0 0
0 0 0 0 0 0 0 0 0 0 0 0 0
0 0 0 0 0 0 0 0
"Viruses;Uroviricota;Caudoviricetes;Caudoviricetes__na;Caudoviricetes__na;Caudoviricetes__na;Shewanella_sp
._phage_3/49" 0 0 0 2 0 0 0 0 0 0 0 0
0 0 0 0 0 0 0 0 0 0 0 0 0
0 0 0 0 0 0 0 0 0 0 0 0 0
0 0 0 0 0 0 0 0 0 0 0 0 0
0 0 0 0 0 0 0 0 0 0 0 0 0
0 0 0 0 0 0 0 0
"Viruses;Uroviricota;Caudoviricetes;Caudoviricetes__na;Caudoviricetes__na;Caudoviricetes__na;Sinorhizobium
_phage_phiM6" 0 0 0 2 33 9 0 0 0 0 0 0
0 0 0 0 0 0 0 0 0 0 0 0 0
0 0 0 0 0 0 0 0 0 0 0 0 0
0 0 0 0 0 0 0 0 0 1 0 0 0
0 0 0 0 0 0 0 0 0 0 0 0 0
0 0 0 0 0 0 0 0
"Viruses;Uroviricota;Caudoviricetes;Caudoviricetes__na;Caudoviricetes__na;Caudoviricetes__na;Siphoviridae_s
p._ct0Bp21" 0 0 0 0 0 0 0 0 0 0 0 0
0 0 0 0 0 0 0 0 0 0 0 0 12
4 16 5 15 0 0 0 0 0 0 0 0 0
0 0 0 9 0 0 0 0 0 0 0 0 0

0 1 0 0 0 0 0 0 0 0 0 0 0
0 0 1 1 0 0 0 0
"Viruses;Uroviricota;Caudoviricetes;Caudoviricetes__na;Caudoviricetes__na;Caudoviricetes__na;Siphoviridae_s
p._ct0Ci105" 0 0 0 0 0 0 0 0 0 0 0 0
0 0 0 0 0 0 0 0 0 0 0 0 0
0 0 0 0 0 0 0 0 0 0 0 0 0
0 0 0 0 0 0 0 0 0 0 0 0 0
0 0 0 0 0 0 0 0 0 0 0 0 0
0 0 2 3 0 0 0 0
"Viruses;Uroviricota;Caudoviricetes;Caudoviricetes__na;Caudoviricetes__na;Caudoviricetes__na;Siphoviridae_s
p._ct0eR1" 0 0 0 0 0 0 0 0 0 0 0 0
0 0 0 0 0 0 0 0 0 0 0 0 0
0 0 0 0 0 0 0 0 0 0 0 0 0
0 0 0 0 0 0 0 0 0 0 0 0 1
0 0 0 0 0 0 0 0 0 0 0 0 0
0 0 0 0 0 0 0 0
"Viruses;Uroviricota;Caudoviricetes;Caudoviricetes__na;Caudoviricetes__na;Caudoviricetes__na;Siphoviridae_s
p._ct0hG5" 0 0 0 0 0 0 0 0 0 0 0 0
0 0 0 0 0 0 0 0 0 0 0 0 0
0 0 0 0 0 9 6 0 1 43 0 82 16
4 1 0 74 6 67 2 1 0 0 0 0 0
0 3 0 0 0 0 0 0 0 0 0 0 1
0 0 0 5 0 0 0 0
"Viruses;Uroviricota;Caudoviricetes;Caudoviricetes__na;Caudoviricetes__na;Caudoviricetes__na;Siphoviridae_s
p._ct0UO21" 0 0 0 0 0 0 0 0 0 0 0 0
0 0 0 0 0 0 0 0 0 0 0 0 0
0 0 0 0 0 0 0 0 0 0 0 0 0
0 0 0 0 0 0 0 0 0 0 0 0 0
0 0 0 7 11 0 0 0 0 0 0 0 0
0 0 5 83 0 3 0 0
"Viruses;Uroviricota;Caudoviricetes;Caudoviricetes__na;Caudoviricetes__na;Caudoviricetes__na;Siphoviridae_s
p._ct0Wl9" 0 0 0 0 0 0 0 0 0 0 0 0
0 0 1 0 2 1 0 0 0 0 0 0 0
0 0 0 0 0 0 2 0 0 0 0 0 0
0 0 0 0 1 0 0 0 0 0 0 0 0
0 0 0 0 0 0 0 0 0 0 0 0 0
0 0 0 0 0 0 0 0
"Viruses;Uroviricota;Caudoviricetes;Caudoviricetes__na;Caudoviricetes__na;Caudoviricetes__na;Siphoviridae_s
p._ct0X023" 0 0 0 0 0 0 0 0 0 0 0 0
0 0 0 0 0 0 0 0 0 0 0 0 0
0 0 0 0 0 0 0 0 0 0 0 2 0
0 0 0 0 0 0 0 0 0 0 0 0 0
0 0 0 0 0 0 0 0 0 0 0 0 0
0 0 0 0 0 0 0 0
"Viruses;Uroviricota;Caudoviricetes;Caudoviricetes__na;Caudoviricetes__na;Caudoviricetes__na;Siphoviridae_s
p._ct0Xn2" 0 0 0 0 0 0 0 0 0 0 0 0
0 0 0 0 0 0 0 0 0 0 0 0 0
0 3 0 0 0 0 0 0 0 0 0 0 0
0 0 0 0 0 0 0 0 0 0 0 0 0

0 0 0 0 0 0 0 0 0 0 0 0 0
0 0 0 0 0 0 0 0
"Viruses;Uroviricota;Caudoviricetes;Caudoviricetes__na;Caudoviricetes__na;Caudoviricetes__na;Siphoviridae_sp._ct13O11" 0 0 0 0 0 0 0 0 0 0 0 0
0 0 0 0 0 0 0 0 0 0 0 0 0
0 0 0 0 0 3 1 0 0 2 0 0 0
0 0 0 0 0 0 0 0 0 0 0 0 0
0 0 0 0 0 0 0 0 0 0 0 0 0
0 0 0 0 0 0 0 0
"Viruses;Uroviricota;Caudoviricetes;Caudoviricetes__na;Caudoviricetes__na;Caudoviricetes__na;Siphoviridae_sp._ct16C7" 0 0 0 0 0 0 0 0 0 0 0 0
0 0 0 0 0 0 0 0 0 0 0 0 0
0 1 0 0 0 0 0 0 0 0 0 1 0
0 0 0 0 0 0 0 0 0 0 0 0 0
0 0 0 0 0 0 0 0 0 0 0 0 0
0 0 1 0 0 0 0 0
"Viruses;Uroviricota;Caudoviricetes;Caudoviricetes__na;Caudoviricetes__na;Caudoviricetes__na;Siphoviridae_sp._ct1E017" 0 0 0 0 0 0 0 0 0 0 0 0
0 0 0 0 0 0 0 0 0 0 0 0 4
3 0 0 0 0 0 0 0 0 0 0 0 0
0 0 0 0 0 0 0 0 0 0 0 0 0
0 0 0 0 0 0 0 0 0 0 0 0 0
0 0 0 0 0 0 0 0
"Viruses;Uroviricota;Caudoviricetes;Caudoviricetes__na;Caudoviricetes__na;Caudoviricetes__na;Siphoviridae_sp._ct1Eo1" 0 0 0 0 0 0 0 0 0 0 0 0
99 234 7 7 2 1 0 0 0 0 0 0 0
0 0 0 0 0 0 0 0 0 0 0 0 0
0 0 0 0 0 0 0 0 0 0 0 0 0
0 0 0 0 0 0 0 0 0 0 0 0 0
0 0 0 0 0 0 0 0
"Viruses;Uroviricota;Caudoviricetes;Caudoviricetes__na;Caudoviricetes__na;Caudoviricetes__na;Siphoviridae_sp._ct1gv6" 0 0 0 0 0 0 0 0 0 0 0 0
0 0 0 0 0 0 0 0 0 0 0 0 13
4 13 2 9 1 0 0 0 0 0 0 0 0
0 0 0 0 0 0 0 0 0 0 0 0 0
0 0 0 0 0 0 0 0 0 0 0 0 0
0 0 0 0 0 0 0 0
"Viruses;Uroviricota;Caudoviricetes;Caudoviricetes__na;Caudoviricetes__na;Caudoviricetes__na;Siphoviridae_sp._ct1Tj2" 0 0 0 0 0 0 0 0 0 0 0 0
0 0 0 0 0 0 0 0 0 0 0 0 0
0 0 0 0 0 0 0 0 0 0 0 0 0
0 0 0 0 0 0 0 0 0 0 0 0 0
0 0 0 0 0 0 0 0 0 0 0 0 0
0 0 0 1 0 0 0 0
"Viruses;Uroviricota;Caudoviricetes;Caudoviricetes__na;Caudoviricetes__na;Caudoviricetes__na;Siphoviridae_sp._ct1TR2" 0 0 0 0 0 0 0 0 0 0 0 0
0 0 0 0 0 0 0 0 0 0 0 0 7
6 0 0 0 0 1 0 0 0 0 0 0 0
0 0 0 0 0 0 0 0 0 0 0 0 0

0 0 0 0 0 0 0 0 0 0 0 0 0
0 0 2 0 0 0 0 0
"Viruses;Uroviricota;Caudoviricetes;Caudoviricetes__na;Caudoviricetes__na;Caudoviricetes__na;Siphoviridae_sp._ct1yA16" 0 0 0 0 0 0 0 0 0 0 0 0
0 1 0 0 0 0 0 0 0 0 0 0 0
0 0 0 0 0 0 0 0 0 0 0 0 1
0 0 0 0 0 0 0 0 0 0 0 0 0
0 0 0 2 1 0 0 0 0 0 0 0 0
0 0 0 0 0 0 0 0
"Viruses;Uroviricota;Caudoviricetes;Caudoviricetes__na;Caudoviricetes__na;Caudoviricetes__na;Siphoviridae_sp._ct1Zj2" 0 0 0 0 0 0 0 0 0 0 0 0
0 0 0 0 0 0 0 0 0 0 0 0 0
0 0 0 0 0 0 0 0 0 0 0 0 0
0 0 0 0 0 0 0 0 0 0 0 0 0
0 0 0 2 0 0 0 0 0 0 0 0 0
0 0 0 0 0 0 0 0
"Viruses;Uroviricota;Caudoviricetes;Caudoviricetes__na;Caudoviricetes__na;Caudoviricetes__na;Siphoviridae_sp._ct2hZ16" 0 0 0 0 0 0 0 0 0 0 0 0
1 1 0 0 0 0 0 0 0 0 0 0 0
0 0 0 0 0 0 0 0 0 0 0 0 0
0 0 0 0 0 0 0 0 0 0 0 0 0
0 0 0 0 0 0 0 0 0 0 0 0 0
0 0 0 0 0 0 0 0
"Viruses;Uroviricota;Caudoviricetes;Caudoviricetes__na;Caudoviricetes__na;Caudoviricetes__na;Siphoviridae_sp._ct2QJ10" 0 0 0 0 0 0 0 0 0 0 0 0
0 0 0 0 0 0 0 0 0 0 0 0 0
0 0 0 0 0 0 0 0 0 0 0 0 0
0 0 0 0 0 0 0 0 0 0 0 0 0
0 0 0 0 0 0 0 0 0 0 0 0 0
0 0 0 2 0 0 0 0
"Viruses;Uroviricota;Caudoviricetes;Caudoviricetes__na;Caudoviricetes__na;Caudoviricetes__na;Siphoviridae_sp._ct2vX3" 0 0 0 0 0 0 0 0 0 0 0 0
0 0 0 0 0 0 1 0 0 0 0 0 0
0 0 0 0 0 0 0 0 0 0 0 0 0
0 0 0 0 0 0 0 0 0 0 0 0 0
0 0 0 0 0 0 0 0 0 0 0 0 0
0 0 1 0 0 0 0 0
"Viruses;Uroviricota;Caudoviricetes;Caudoviricetes__na;Caudoviricetes__na;Caudoviricetes__na;Siphoviridae_sp._ct39g3" 0 0 0 0 0 0 0 0 0 0 0 0
0 0 0 0 0 0 0 0 0 0 0 0 0
0 0 0 0 0 0 0 0 0 0 0 1 1
0 0 0 0 0 0 0 0 0 0 0 0 0
0 0 0 0 0 0 0 0 0 0 0 0 0
0 0 0 1 0 0 0 0
"Viruses;Uroviricota;Caudoviricetes;Caudoviricetes__na;Caudoviricetes__na;Caudoviricetes__na;Siphoviridae_sp._ct3an14" 0 0 0 0 0 0 0 0 0 0 0 0
0 0 0 0 0 0 0 0 0 0 0 0 0
0 0 0 0 0 0 0 0 0 0 0 0 0
0 0 0 11 0 0 0 0 0 0 0 0 0

0 0 0 0 0 0 0 0 0 0 0 0 0
0 0 0 0 0 0 0 0
"Viruses;Uroviricota;Caudoviricetes;Caudoviricetes__na;Caudoviricetes__na;Caudoviricetes__na;Siphoviridae_sp._ct3es5" 0 0 0 0 0 0 0 0 0 0 0 0
2 0 1 0 0 0 3 9 0 0 0 0 27
18 0 0 2 0 2 3 0 0 4 0 11 10
0 0 0 0 8 46 0 0 8 0 17 15 0
0 0 0 7 11 0 0 0 0 31 3 0 0
0 0 4 13 0 2 0 0
"Viruses;Uroviricota;Caudoviricetes;Caudoviricetes__na;Caudoviricetes__na;Caudoviricetes__na;Siphoviridae_sp._ct3lF2" 0 0 0 0 0 0 0 0 0 0 0 0
61 211 12 9 0 0 0 0 0 0 0 0 0
0 0 0 0 0 0 0 0 0 0 0 0 0
0 0 0 0 0 0 0 0 0 0 0 0 0
0 0 0 2 0 0 0 0 0 0 0 0 0
0 0 0 0 0 0 0 0
"Viruses;Uroviricota;Caudoviricetes;Caudoviricetes__na;Caudoviricetes__na;Caudoviricetes__na;Siphoviridae_sp._ct3o911" 0 0 0 0 0 0 0 0 0 0 0 0
0 0 0 0 0 0 0 0 0 0 0 0 0
1 0 0 0 0 0 0 0 0 0 0 0 0
0 0 0 0 0 0 0 0 0 0 0 0 0
0 0 0 0 0 0 0 0 0 0 0 0 0
0 0 0 0 0 0 0 0
"Viruses;Uroviricota;Caudoviricetes;Caudoviricetes__na;Caudoviricetes__na;Caudoviricetes__na;Siphoviridae_sp._ct3r22" 0 0 0 0 0 0 0 0 0 0 0 0
0 0 1 0 0 5 0 0 0 0 0 0 0
0 0 0 0 0 0 0 0 0 0 0 0 1
0 0 0 0 0 0 0 0 0 0 0 0 0
0 0 0 0 0 0 0 0 0 55 7 4 12
2 0 3 2 0 0 0 0
"Viruses;Uroviricota;Caudoviricetes;Caudoviricetes__na;Caudoviricetes__na;Caudoviricetes__na;Siphoviridae_sp._ct3yx7" 0 0 0 0 0 0 0 0 0 0 0 0
9 5 0 0 0 0 0 0 0 0 0 0 0
0 0 0 0 0 0 0 0 0 0 0 0 2
0 0 0 0 0 0 0 0 0 0 0 2 0
0 0 0 0 0 0 0 0 0 0 0 0 0
0 0 0 0 0 0 0 0
"Viruses;Uroviricota;Caudoviricetes;Caudoviricetes__na;Caudoviricetes__na;Caudoviricetes__na;Siphoviridae_sp._ct3z32" 0 0 0 0 0 0 0 0 0 0 0 0
0 0 0 0 0 0 0 0 0 0 0 0 0
0 0 0 0 0 54 59 0 0 3 1 0 0
0 0 0 0 0 0 0 0 0 0 0 0 0
0 0 0 0 0 0 0 0 0 0 0 0 0
0 0 0 0 0 0 0 0
"Viruses;Uroviricota;Caudoviricetes;Caudoviricetes__na;Caudoviricetes__na;Caudoviricetes__na;Siphoviridae_sp._ct47J5" 0 0 0 0 0 0 0 0 0 0 0 0
0 0 0 0 0 0 0 0 0 0 0 0 2
2 0 0 0 0 0 0 0 0 1 0 0 0
0 0 0 0 0 0 0 0 0 0 0 1 0

0 0 0 0 1 0 0 0 0 0 0 0 0
0 0 0 0 0 0 0 0
"Viruses;Uroviricota;Caudoviricetes;Caudoviricetes__na;Caudoviricetes__na;Caudoviricetes__na;Siphoviridae_sp._ct47y1" 0 0 0 0 0 0 0 0 0 0 0 0
0 0 0 0 0 0 1 4 0 0 0 0 0
0 0 0 0 0 0 0 0 0 0 0 1 2
0 0 0 0 13 4 8 0 0 0 0 0 0
0 0 0 0 0 0 0 0 0 0 0 0 0
0 0 4 1 0 0 0 0
"Viruses;Uroviricota;Caudoviricetes;Caudoviricetes__na;Caudoviricetes__na;Caudoviricetes__na;Siphoviridae_sp._ct4aE30" 0 0 0 0 0 0 0 0 0 0 0 0
1 0 0 0 0 0 1 2 0 0 0 0 0
0 0 0 0 0 0 0 0 0 0 0 0 0
0 0 0 0 0 0 0 0 0 0 0 0 0
0 0 0 0 1 0 0 0 0 0 0 0 0
0 0 0 1 0 0 0 0
"Viruses;Uroviricota;Caudoviricetes;Caudoviricetes__na;Caudoviricetes__na;Caudoviricetes__na;Siphoviridae_sp._ct4Am4" 0 0 0 0 0 0 0 0 0 0 0 0
0 0 0 0 0 0 0 0 0 0 0 0 0
0 0 0 0 0 1 5 0 0 0 0 24 19
1 2 0 23 0 0 0 0 0 0 0 0 0
0 3 0 0 0 0 0 0 0 0 0 0 0
0 0 1 0 0 0 0 0
"Viruses;Uroviricota;Caudoviricetes;Caudoviricetes__na;Caudoviricetes__na;Caudoviricetes__na;Siphoviridae_sp._ct4be24" 0 0 0 0 0 0 0 0 0 0 0 0
0 0 0 0 0 0 0 0 0 0 0 0 0
0 0 0 0 0 0 1 0 0 0 0 0 1
0 0 0 0 0 0 0 0 0 0 0 0 0
0 0 0 0 0 0 0 0 0 0 0 0 0
0 0 3 2 0 0 0 0
"Viruses;Uroviricota;Caudoviricetes;Caudoviricetes__na;Caudoviricetes__na;Caudoviricetes__na;Siphoviridae_sp._ct4T77" 0 0 0 0 0 0 0 0 0 0 0 0
0 0 0 0 0 0 0 0 0 0 0 0 0
0 0 0 0 0 0 0 0 0 0 5 0 0
0 0 0 0 0 0 0 0 0 0 0 0 0
0 0 0 0 0 0 0 0 0 0 0 0 0
0 0 0 0 0 0 0 0
"Viruses;Uroviricota;Caudoviricetes;Caudoviricetes__na;Caudoviricetes__na;Caudoviricetes__na;Siphoviridae_sp._ct4Uy2" 0 0 0 0 0 0 0 0 0 0 0 0
0 0 0 0 0 0 0 0 0 0 0 0 0
0 0 0 0 0 0 0 0 0 0 0 0 1
0 0 0 0 0 0 0 0 0 0 0 0 0
0 0 0 0 0 0 0 0 0 0 0 0 0
0 0 0 0 0 0 0 0
"Viruses;Uroviricota;Caudoviricetes;Caudoviricetes__na;Caudoviricetes__na;Caudoviricetes__na;Siphoviridae_sp._ct4Z13" 0 0 0 0 0 0 0 0 0 0 0 0
0 0 0 0 0 0 0 1 0 0 0 0 0
0 0 0 0 0 0 0 0 0 0 0 0 2
0 0 0 0 0 1 0 0 0 0 0 0 0

0 0 0 0 0 0 0 0 0 0 0 0 0
0 0 2 0 0 0 0 0
"Viruses;Uroviricota;Caudoviricetes;Caudoviricetes__na;Caudoviricetes__na;Caudoviricetes__na;Siphoviridae_s
p._ct57T16" 0 0 0 0 0 0 0 0 0 0 0 0
3 1 1 1 2 1 0 1 0 0 0 0 0
0 0 0 0 0 0 2 0 0 0 0 1 1
0 0 0 0 0 0 0 0 0 0 11 5 1
4 0 0 0 2 0 0 0 0 0 0 0 0
0 0 0 1 0 0 0 0
"Viruses;Uroviricota;Caudoviricetes;Caudoviricetes__na;Caudoviricetes__na;Caudoviricetes__na;Siphoviridae_s
p._ct58A13" 0 0 0 0 0 0 0 0 0 0 0 0
0 0 0 0 0 0 0 0 0 0 0 0 0
0 0 0 0 0 0 0 0 0 0 0 1 0
0 0 0 0 0 0 0 0 0 0 0 0 0
0 0 0 0 0 0 0 0 0 0 0 0 0
0 0 0 0 0 0 0 0
"Viruses;Uroviricota;Caudoviricetes;Caudoviricetes__na;Caudoviricetes__na;Caudoviricetes__na;Siphoviridae_s
p._ct58H1" 0 0 0 0 0 0 0 0 0 0 0 0
0 0 0 0 0 0 1 2 0 0 0 0 0
0 0 0 0 0 1 0 0 0 0 0 0 0
0 0 0 0 0 0 0 0 0 0 0 0 0
0 0 0 0 0 0 0 0 0 0 0 0 0
0 0 0 0 0 0 0 0
"Viruses;Uroviricota;Caudoviricetes;Caudoviricetes__na;Caudoviricetes__na;Caudoviricetes__na;Siphoviridae_s
p._ct5co22" 0 0 0 0 0 0 0 0 0 0 0 0
0 0 0 1 0 0 0 0 0 0 0 0 0
0 0 0 0 0 0 0 0 0 0 0 0 0
0 0 0 0 0 0 0 0 0 0 1 0 0
0 0 0 1 0 0 0 0 0 0 0 0 0
0 0 0 0 0 0 0 0
"Viruses;Uroviricota;Caudoviricetes;Caudoviricetes__na;Caudoviricetes__na;Caudoviricetes__na;Siphoviridae_s
p._ct5FX1" 0 0 0 0 0 0 0 0 0 0 0 0
2 0 0 0 0 0 0 0 0 0 0 0 0
0 0 0 0 0 0 0 0 0 0 0 0 0
0 0 0 0 0 0 0 0 0 0 0 0 0
0 0 0 0 0 0 0 0 0 0 0 0 0
0 0 0 0 0 0 0 0
"Viruses;Uroviricota;Caudoviricetes;Caudoviricetes__na;Caudoviricetes__na;Caudoviricetes__na;Siphoviridae_s
p._ct5jB2" 0 0 0 0 0 0 0 0 0 0 0 0
1 0 1 0 1 16 0 5 0 2 1 0 0
0 0 0 0 0 0 1 0 0 0 2 0 0
0 0 0 0 1 3 1 0 0 0 2 0 0
0 0 0 5 9 0 0 0 0 1 1 0 0
0 0 2 1 0 0 0 0
"Viruses;Uroviricota;Caudoviricetes;Caudoviricetes__na;Caudoviricetes__na;Caudoviricetes__na;Siphoviridae_s
p._ct5Px37" 0 0 0 0 0 0 0 0 0 0 0 0
0 1 0 0 0 0 0 0 0 0 0 0 0
0 0 0 0 0 3 4 0 1 0 0 0 0
0 0 0 0 0 0 0 0 0 0 0 0 0

0 0 71 1 2 0 0 0 0 0 0 0 0
0 0 5 10 2 4 0 0
"Viruses;Uroviricota;Caudoviricetes;Caudoviricetes__na;Caudoviricetes__na;Caudoviricetes__na;Siphoviridae_s
p._ct5qs5" 0 0 0 0 0 0 0 0 0 0 0 0
0 1 0 2 0 0 0 0 0 0 0 0 0
0 0 0 0 0 0 0 0 0 0 0 0 0
0 0 0 0 0 0 0 0 0 0 3 0 0
0 0 0 0 0 0 0 0 0 0 0 0 0
0 0 0 0 0 0 0 0
"Viruses;Uroviricota;Caudoviricetes;Caudoviricetes__na;Caudoviricetes__na;Caudoviricetes__na;Siphoviridae_s
p._ct5YG1" 0 0 0 0 0 0 0 0 0 0 0 0
1 0 0 0 0 0 0 0 0 0 0 0 0
0 0 0 0 0 0 0 0 0 0 0 0 0
0 0 0 0 0 0 0 0 0 0 0 0 0
0 0 0 0 0 0 0 0 0 0 0 0 0
0 0 0 0 0 0 0 0
"Viruses;Uroviricota;Caudoviricetes;Caudoviricetes__na;Caudoviricetes__na;Caudoviricetes__na;Siphoviridae_s
p._ct5zp6" 0 0 0 0 0 0 0 0 0 0 0 0
0 0 0 0 0 0 0 0 0 0 0 0 0
0 0 0 0 0 0 1 0 0 0 0 0 0
0 0 0 0 0 0 0 0 0 0 0 0 0
0 0 0 0 0 0 0 0 0 0 0 0 0
0 0 0 0 0 0 0 0
"Viruses;Uroviricota;Caudoviricetes;Caudoviricetes__na;Caudoviricetes__na;Caudoviricetes__na;Siphoviridae_s
p._ct6d71" 0 0 0 0 0 0 0 0 0 0 0 0
0 0 0 0 0 0 0 0 0 0 0 0 0
1 0 0 0 0 0 0 0 0 0 0 0 0
0 0 0 0 0 0 0 0 0 0 0 0 1
0 0 0 1 0 0 0 0 0 0 0 0 0
0 0 0 0 0 0 0 0
"Viruses;Uroviricota;Caudoviricetes;Caudoviricetes__na;Caudoviricetes__na;Caudoviricetes__na;Siphoviridae_s
p._ct6DP12" 0 1 0 0 0 0 0 0 0 0 0 0
0 0 0 0 0 0 0 0 0 0 0 0 0
0 0 0 0 0 0 0 0 0 0 0 0 0
0 0 0 0 0 0 0 0 0 0 0 0 0
0 0 0 0 0 0 0 0 0 0 0 0 0
0 0 0 0 0 0 0 0
"Viruses;Uroviricota;Caudoviricetes;Caudoviricetes__na;Caudoviricetes__na;Caudoviricetes__na;Siphoviridae_s
p._ct6GI21" 0 0 0 0 0 0 0 0 0 0 0 0
0 0 0 0 0 0 0 0 0 0 0 0 0
0 0 0 0 0 0 1 0 0 0 0 0 0
0 0 0 0 0 0 0 0 0 0 0 0 0
0 0 0 0 0 0 0 0 0 0 0 0 0
0 0 0 0 0 0 0 0
"Viruses;Uroviricota;Caudoviricetes;Caudoviricetes__na;Caudoviricetes__na;Caudoviricetes__na;Siphoviridae_s
p._ct6JW23" 0 0 0 0 0 0 0 0 0 0 0 0
0 0 0 0 0 0 0 0 0 0 0 0 1
0 0 0 0 0 0 0 0 0 0 0 1 0
0 0 0 0 0 0 0 0 0 0 0 0 0

0 0 0 0 0 0 0 0 0 0 0 0 0
0 0 0 0 0 0 0 0
"Viruses;Uroviricota;Caudoviricetes;Caudoviricetes__na;Caudoviricetes__na;Caudoviricetes__na;Siphoviridae_s
p._ct6Ob18" 0 0 0 0 0 0 0 0 0 0 0 0
0 0 0 0 0 0 0 0 0 0 0 0 0
0 0 0 0 0 2 0 0 0 0 0 0 0
0 0 0 0 0 0 0 0 0 0 0 0 0
0 0 0 0 0 0 0 0 0 0 0 0 0
0 0 0 0 0 0 0 0
"Viruses;Uroviricota;Caudoviricetes;Caudoviricetes__na;Caudoviricetes__na;Caudoviricetes__na;Siphoviridae_s
p._ct6oU4" 0 0 0 0 0 0 0 0 0 0 0 0
0 0 0 0 0 0 0 0 0 0 0 0 0
0 0 0 0 0 0 0 0 0 0 0 0 0
0 0 0 0 0 0 0 0 0 0 0 0 0
0 0 0 1 2 0 0 0 0 0 0 0 0
0 0 0 0 0 0 0 0
"Viruses;Uroviricota;Caudoviricetes;Caudoviricetes__na;Caudoviricetes__na;Caudoviricetes__na;Siphoviridae_s
p._ct6ro14" 0 0 0 0 0 0 0 0 0 0 0 0
0 2 0 0 0 5 0 0 0 0 0 0 0
0 0 0 0 0 0 0 0 0 0 0 0 0
0 0 0 0 0 0 0 0 0 0 0 0 0
0 0 0 0 0 0 0 0 0 1 1 0 0
0 0 0 0 0 0 0 0
"Viruses;Uroviricota;Caudoviricetes;Caudoviricetes__na;Caudoviricetes__na;Caudoviricetes__na;Siphoviridae_s
p._ct6tD39" 0 0 0 0 0 0 0 0 0 0 0 0
1 1 0 0 0 1 0 0 0 0 0 0 0
0 0 0 0 0 0 0 0 0 0 0 0 0
0 0 0 0 0 0 0 0 0 0 0 0 0
0 0 0 0 1 0 0 0 0 0 0 0 0
0 0 0 0 0 0 0 0
"Viruses;Uroviricota;Caudoviricetes;Caudoviricetes__na;Caudoviricetes__na;Caudoviricetes__na;Siphoviridae_s
p._ct73D3" 0 0 0 0 0 0 0 0 0 0 0 0
0 0 0 0 0 0 1 1 0 0 0 0 0
0 0 0 0 0 0 1 0 0 0 0 0 0
0 0 0 0 0 0 0 0 0 0 0 0 0
0 0 0 0 0 0 0 0 0 0 0 0 0
0 0 0 0 0 0 0 0
"Viruses;Uroviricota;Caudoviricetes;Caudoviricetes__na;Caudoviricetes__na;Caudoviricetes__na;Siphoviridae_s
p._ct73V17" 0 0 0 0 0 0 0 0 0 0 0 0
0 0 0 0 0 0 0 1 0 0 0 0 0
0 0 0 0 0 0 0 0 0 0 0 0 0
0 0 0 37 0 0 0 0 0 0 0 0 0
0 0 0 0 0 0 0 0 0 0 0 0 0
0 0 0 1 0 0 0 0
"Viruses;Uroviricota;Caudoviricetes;Caudoviricetes__na;Caudoviricetes__na;Caudoviricetes__na;Siphoviridae_s
p._ct7BG1" 0 0 0 0 0 0 0 0 0 0 0 0
3 9 1 0 0 0 0 0 0 0 0 0 0
0 0 0 0 0 0 0 0 0 0 0 0 0
0 0 0 0 0 0 0 0 0 0 0 0 0

0 0 0 0 0 0 0 0 0 0 0 0 0
0 0 0 0 0 0 0 0
"Viruses;Uroviricota;Caudoviricetes;Caudoviricetes__na;Caudoviricetes__na;Caudoviricetes__na;Siphoviridae_s
p._ct7es18" 0 0 0 0 0 0 0 0 0 0 0 0
1 2 2 3 1 7 1 2 0 0 0 0 0
0 0 0 0 0 0 0 0 0 0 0 0 0
0 0 0 0 0 0 1 0 0 0 0 0 0
0 0 0 1 1 0 0 0 0 0 0 0 0
0 0 0 0 0 0 0 0
"Viruses;Uroviricota;Caudoviricetes;Caudoviricetes__na;Caudoviricetes__na;Caudoviricetes__na;Siphoviridae_s
p._ct7EW56" 0 0 0 0 0 0 0 0 0 0 0 0
0 0 0 0 0 0 0 0 0 0 0 0 0
0 0 0 0 0 0 0 0 0 0 0 0 0
0 0 0 0 0 0 0 0 0 0 0 0 0
0 0 3 0 0 0 0 0 0 0 0 0 0
0 0 0 1 0 0 1 0
"Viruses;Uroviricota;Caudoviricetes;Caudoviricetes__na;Caudoviricetes__na;Caudoviricetes__na;Siphoviridae_s
p._ct7JV2" 0 0 0 0 0 0 0 0 0 0 0 0
0 0 0 0 0 0 0 0 0 0 0 0 0
0 0 0 0 0 0 0 0 0 0 0 0 0
0 0 0 0 0 0 0 0 0 0 0 0 0
0 0 0 0 0 0 0 0 0 1 0 1 0
0 0 0 0 0 0 0 0
"Viruses;Uroviricota;Caudoviricetes;Caudoviricetes__na;Caudoviricetes__na;Caudoviricetes__na;Siphoviridae_s
p._ct7ub6" 0 0 0 0 0 0 0 0 0 0 0 0
0 0 0 0 0 0 0 1 0 0 0 0 0
0 0 0 0 0 0 0 0 0 0 0 0 0
0 0 0 0 0 0 0 0 0 0 0 0 0
0 0 0 0 0 0 0 0 0 0 0 0 0
0 0 0 0 0 0 0 0
"Viruses;Uroviricota;Caudoviricetes;Caudoviricetes__na;Caudoviricetes__na;Caudoviricetes__na;Siphoviridae_s
p._ct7yc1" 0 0 0 0 0 0 0 0 0 0 0 0
0 0 0 0 0 0 0 0 0 0 0 0 0
0 0 0 0 0 0 0 0 0 32 0 0 0
0 0 0 0 0 0 0 0 0 0 0 0 0
0 0 0 0 0 0 0 0 0 0 0 0 0
0 0 0 0 0 0 0 0
"Viruses;Uroviricota;Caudoviricetes;Caudoviricetes__na;Caudoviricetes__na;Caudoviricetes__na;Siphoviridae_s
p._ct8hB11" 0 0 0 0 0 0 0 0 0 0 0 0
0 0 0 0 0 0 0 0 0 0 0 0 0
0 0 0 0 0 0 0 0 0 0 0 0 0
0 0 0 0 0 0 0 0 0 0 0 0 0
0 0 0 1 2 0 0 0 0 0 0 0 0
0 0 0 2 0 0 0 0
"Viruses;Uroviricota;Caudoviricetes;Caudoviricetes__na;Caudoviricetes__na;Caudoviricetes__na;Siphoviridae_s
p._ct8LQ5" 0 0 0 0 0 0 0 0 0 0 0 0
0 0 0 0 0 0 0 0 0 0 0 0 0
0 0 0 0 0 1 0 0 0 0 0 0 0
0 0 0 0 0 0 0 0 0 0 0 0 0

0 0 0 0 0 0 0 0 0 0 0 0 0
0 0 1 3 0 0 0 0
"Viruses;Uroviricota;Caudoviricetes;Caudoviricetes__na;Caudoviricetes__na;Caudoviricetes__na;Siphoviridae_sp._ct8LX107" 0 0 0 0 0 0 0 0 0 0 0 0
0 0 0 0 0 0 0 0 0 0 0 0 0
0 0 0 0 0 3 0 0 0 0 0 44 34
1 4 0 29 0 0 0 0 0 0 0 0 0
0 3 0 0 1 0 0 0 0 0 0 0 0
0 0 0 0 0 0 0 0
"Viruses;Uroviricota;Caudoviricetes;Caudoviricetes__na;Caudoviricetes__na;Caudoviricetes__na;Siphoviridae_sp._ct8NQ14" 0 0 0 0 0 0 0 0 0 0 0 0
0 0 0 0 0 0 0 0 0 0 0 0 10
12 1 0 3 0 2 0 2 0 583 0 3 1
0 0 0 0 0 0 0 0 0 0 0 0 0
0 0 0 0 0 0 0 0 0 0 0 0 0
0 0 0 0 0 0 0 0
"Viruses;Uroviricota;Caudoviricetes;Caudoviricetes__na;Caudoviricetes__na;Caudoviricetes__na;Siphoviridae_sp._ct8rU2" 0 0 0 0 0 0 0 0 0 0 0 0
0 1 0 0 0 0 0 0 0 0 0 0 0
0 0 0 0 0 0 0 0 0 0 0 0 0
0 0 0 0 0 0 0 0 0 0 0 0 0
0 0 0 0 0 0 0 0 0 0 0 0 0
0 0 0 0 0 0 0 0
"Viruses;Uroviricota;Caudoviricetes;Caudoviricetes__na;Caudoviricetes__na;Caudoviricetes__na;Siphoviridae_sp._ct96x5" 0 0 0 0 0 0 0 0 0 0 0 0
0 0 0 0 0 0 0 0 0 0 0 0 0
0 0 0 0 0 0 0 0 0 0 0 0 0
0 0 0 0 0 0 0 0 0 0 0 0 0
0 0 0 0 1 0 0 0 0 0 0 0 0
0 0 0 0 0 0 0 0
"Viruses;Uroviricota;Caudoviricetes;Caudoviricetes__na;Caudoviricetes__na;Caudoviricetes__na;Siphoviridae_sp._ct9lR64" 0 0 0 0 0 0 0 0 0 0 0 0
0 0 0 0 0 0 0 0 0 0 0 0 0
0 0 0 0 0 0 0 0 0 0 0 0 1
0 0 0 0 0 0 0 0 0 0 0 0 0
0 0 0 0 0 0 0 0 0 0 0 0 0
0 0 0 0 0 0 0 0
"Viruses;Uroviricota;Caudoviricetes;Caudoviricetes__na;Caudoviricetes__na;Caudoviricetes__na;Siphoviridae_sp._ct9mC1" 0 0 0 0 0 0 0 0 0 0 0 0
0 1 0 0 0 0 0 1 0 0 0 0 0
0 0 0 0 0 0 0 0 0 0 0 0 0
0 0 0 0 0 0 0 0 0 0 0 0 0
0 0 0 0 0 0 0 0 0 0 0 0 0
0 0 0 0 0 0 0 0
"Viruses;Uroviricota;Caudoviricetes;Caudoviricetes__na;Caudoviricetes__na;Caudoviricetes__na;Siphoviridae_sp._ct9Y44" 0 0 0 0 0 0 0 0 0 0 0 0
0 0 0 0 0 0 0 0 0 0 0 0 0
0 0 0 0 0 11 7 0 0 802 0 0 0
0 0 0 0 0 0 0 0 0 0 0 0 0

0 0 0 1 0 0 0 0 0 0 0 0 0
0 0 8 4 0 0 6 0
"Viruses;Uroviricota;Caudoviricetes;Caudoviricetes__na;Caudoviricetes__na;Caudoviricetes__na;Siphoviridae_s
p._ct9zP9" 0 0 0 0 0 0 0 0 0 0 0 0
0 0 0 0 0 0 0 0 0 0 0 0 0
0 0 0 0 0 0 0 0 0 0 0 0 0
0 0 0 0 0 0 0 0 0 0 0 0 0
0 0 0 0 0 0 0 0 0 0 0 0 0
0 0 1 0 0 0 0 0
"Viruses;Uroviricota;Caudoviricetes;Caudoviricetes__na;Caudoviricetes__na;Caudoviricetes__na;Siphoviridae_s
p._cta6m1" 0 0 0 0 0 0 0 0 0 0 0 0
4 4 0 0 27 76 0 0 0 0 0 0 0
0 0 0 0 0 0 0 0 0 5 0 0 0
0 0 0 0 0 0 0 0 0 0 0 0 0
0 0 0 0 0 0 0 0 0 0 0 0 0
0 0 0 0 0 0 0 0
"Viruses;Uroviricota;Caudoviricetes;Caudoviricetes__na;Caudoviricetes__na;Caudoviricetes__na;Siphoviridae_s
p._ctA995" 0 0 0 0 0 0 0 0 0 0 0 0
0 0 0 0 0 0 0 1 0 0 0 0 0
0 0 0 0 0 0 0 0 0 0 0 0 0
0 0 0 0 0 0 0 0 0 0 0 0 0
0 0 0 0 0 0 0 0 0 0 0 0 0
0 0 0 0 0 0 0 0
"Viruses;Uroviricota;Caudoviricetes;Caudoviricetes__na;Caudoviricetes__na;Caudoviricetes__na;Siphoviridae_s
p._ctaA31" 0 0 0 0 0 0 0 0 0 0 0 0
0 0 0 0 0 0 0 0 0 0 0 0 0
0 0 0 0 0 3 5 16 0 1327 2 0 0
0 0 0 0 0 0 0 0 0 0 0 0 0
0 0 0 0 0 0 0 0 0 0 0 0 0
0 0 0 0 0 0 0 0
"Viruses;Uroviricota;Caudoviricetes;Caudoviricetes__na;Caudoviricetes__na;Caudoviricetes__na;Siphoviridae_s
p._ctacm4" 0 0 0 0 0 0 0 0 0 0 0 0
0 0 0 0 0 0 0 0 0 0 0 0 0
0 0 0 0 0 0 0 0 0 0 0 0 0
0 0 0 0 0 0 0 0 0 0 0 0 0
0 0 0 0 0 0 0 0 0 0 0 0 0
0 0 1 1 0 0 0 0
"Viruses;Uroviricota;Caudoviricetes;Caudoviricetes__na;Caudoviricetes__na;Caudoviricetes__na;Siphoviridae_s
p._ctAeS79" 0 0 0 0 0 0 0 0 0 0 0 0
0 0 0 0 0 0 0 0 0 0 0 0 0
0 0 0 0 0 0 0 0 0 0 0 0 1
0 0 0 0 0 0 0 0 0 0 0 0 0
0 0 0 0 0 0 0 0 0 0 0 0 0
0 0 0 0 0 0 0 0
"Viruses;Uroviricota;Caudoviricetes;Caudoviricetes__na;Caudoviricetes__na;Caudoviricetes__na;Siphoviridae_s
p._ctAFE3" 0 0 0 0 0 0 0 0 0 0 0 0
0 0 0 0 0 0 0 0 0 0 0 0 0
0 0 0 0 0 0 0 0 0 0 0 0 0
0 0 0 0 0 0 0 0 0 0 0 0 0

0 0 0 0 0 0 0 0 0 1 0 0 0
0 0 0 0 0 0 0 0
"Viruses;Uroviricota;Caudoviricetes;Caudoviricetes__na;Caudoviricetes__na;Caudoviricetes__na;Siphoviridae_s
p._ctAnS47" 0 0 0 0 0 0 0 0 0 0 0 0
0 0 0 1 1 2 0 0 0 0 0 0 0
1 0 0 0 0 5 4 0 0 0 0 9 0
0 0 0 0 0 0 0 0 0 0 0 0 0
0 0 0 1 5 0 0 0 0 0 0 0 0
0 0 0 0 0 0 0 0
"Viruses;Uroviricota;Caudoviricetes;Caudoviricetes__na;Caudoviricetes__na;Caudoviricetes__na;Siphoviridae_s
p._ctaqI5" 0 0 0 0 0 0 0 0 0 0 0 0
0 0 0 0 0 0 0 0 0 0 0 0 0
0 0 0 0 0 2 8 6 3 616 2 0 0
0 0 0 0 0 0 0 0 0 0 0 0 0
0 0 0 0 0 0 0 0 0 0 0 0 0
0 0 0 0 0 0 0 0
"Viruses;Uroviricota;Caudoviricetes;Caudoviricetes__na;Caudoviricetes__na;Caudoviricetes__na;Siphoviridae_s
p._ctAUQ2" 0 0 0 0 0 0 0 0 0 0 0 0
1 1 1 2 0 3 15 30 0 0 0 2 0
0 0 0 0 0 0 0 0 0 0 0 0 1
0 0 0 0 13 3 1 0 0 0 0 0 0
0 0 0 0 0 0 0 0 0 0 0 0 0
0 0 0 0 0 0 0 0
"Viruses;Uroviricota;Caudoviricetes;Caudoviricetes__na;Caudoviricetes__na;Caudoviricetes__na;Siphoviridae_s
p._ctAvy12" 0 0 0 0 0 0 0 0 0 0 0 0
0 0 0 0 0 0 0 0 0 0 0 0 0
0 0 0 0 0 0 0 0 0 0 0 3 1
0 0 0 0 0 0 0 0 0 0 0 0 0
0 0 0 0 0 0 0 0 0 1 0 1 0
0 0 0 0 0 0 0 0
"Viruses;Uroviricota;Caudoviricetes;Caudoviricetes__na;Caudoviricetes__na;Caudoviricetes__na;Siphoviridae_s
p._ctb1k4" 0 0 0 0 0 0 0 0 0 0 0 0
0 3 0 0 0 0 1 0 0 0 0 0 2
0 0 0 0 0 0 0 0 0 0 0 0 0
0 0 0 0 0 0 0 0 0 0 0 0 0
0 0 0 0 0 0 0 0 0 0 1 0 0
0 0 0 1 0 0 0 0
"Viruses;Uroviricota;Caudoviricetes;Caudoviricetes__na;Caudoviricetes__na;Caudoviricetes__na;Siphoviridae_s
p._ctB9E3" 0 0 0 0 0 0 0 0 0 0 0 0
0 0 0 0 0 0 0 0 0 0 0 0 0
0 0 0 0 0 0 0 0 0 0 0 0 0
0 0 0 0 0 0 0 0 0 0 0 0 0
0 0 0 0 0 0 0 0 0 0 0 0 0
0 0 2 2 0 1 0 0
"Viruses;Uroviricota;Caudoviricetes;Caudoviricetes__na;Caudoviricetes__na;Caudoviricetes__na;Siphoviridae_s
p._ctB9N2" 0 0 0 0 0 0 0 0 0 0 0 0
0 0 0 0 0 0 0 0 0 0 0 0 0
0 0 0 0 0 0 0 0 0 0 0 1 7
0 0 0 8 0 0 0 0 0 0 0 0 0

0 1 0 0 0 0 0 0 0 0 0 0 0
0 0 0 0 0 0 0 0
"Viruses;Uroviricota;Caudoviricetes;Caudoviricetes__na;Caudoviricetes__na;Caudoviricetes__na;Siphoviridae_sp._ctBAZ2" 0 0 0 0 0 0 0 0 0 0 0 0
0 0 0 0 0 0 0 0 0 0 0 0 0
0 0 0 0 0 0 2 0 0 1 0 0 0
0 0 0 0 0 0 0 0 0 0 0 0 0
0 0 0 0 0 0 0 0 0 0 0 0 0
0 0 0 0 0 0 0 0
"Viruses;Uroviricota;Caudoviricetes;Caudoviricetes__na;Caudoviricetes__na;Caudoviricetes__na;Siphoviridae_sp._ctBCr48" 0 0 0 0 1 0 0 0 0 0 0 0
0 0 0 0 0 0 0 0 0 0 0 0 0
0 0 0 0 0 0 0 0 0 0 0 0 0
0 0 0 0 0 0 0 0 0 0 0 0 0
0 0 0 0 0 0 0 0 0 0 0 0 0
0 0 0 0 0 0 0 0
"Viruses;Uroviricota;Caudoviricetes;Caudoviricetes__na;Caudoviricetes__na;Caudoviricetes__na;Siphoviridae_sp._ctbgC51" 0 0 0 0 0 0 0 0 0 0 0 0
1 0 0 0 0 0 0 0 0 0 0 0 0
0 0 0 0 0 2 3 0 0 0 0 0 0
0 0 0 0 0 0 0 0 0 0 0 0 0
0 0 0 0 0 0 0 0 0 0 0 0 0
0 0 0 0 0 0 0 0
"Viruses;Uroviricota;Caudoviricetes;Caudoviricetes__na;Caudoviricetes__na;Caudoviricetes__na;Siphoviridae_sp._ctBLh2" 0 0 0 0 0 0 0 0 0 0 0 0
11 17 8 7 1 0 5 6 5 1 0 1 9
0 4 0 3 0 0 1 0 0 0 0 5 1
1 0 0 0 1 1 0 0 14 0 4 1 1
2 0 0 17 8 6 3 0 0 0 0 0 0
0 0 12 5 0 3 0 0
"Viruses;Uroviricota;Caudoviricetes;Caudoviricetes__na;Caudoviricetes__na;Caudoviricetes__na;Siphoviridae_sp._ctbrg2" 0 0 0 0 0 0 0 0 0 0 0 0
0 0 0 0 0 0 0 0 0 0 0 0 0
0 0 0 0 0 6 7 0 0 0 0 0 0
0 0 0 0 0 0 0 0 0 0 0 0 0
0 0 0 0 0 0 0 0 0 0 0 0 0
0 0 0 1 0 0 0 0
"Viruses;Uroviricota;Caudoviricetes;Caudoviricetes__na;Caudoviricetes__na;Caudoviricetes__na;Siphoviridae_sp._ctBtS10" 0 0 0 0 0 0 0 0 0 0 0 0
0 0 0 0 0 0 0 0 0 0 0 0 0
0 0 0 0 0 0 0 0 0 0 0 1 1
0 0 0 0 0 0 0 0 0 0 0 0 0
0 0 0 1 3 0 0 0 0 0 0 0 0
0 0 0 0 0 0 0 0
"Viruses;Uroviricota;Caudoviricetes;Caudoviricetes__na;Caudoviricetes__na;Caudoviricetes__na;Siphoviridae_sp._ctBtT10" 0 0 0 0 0 0 0 0 0 0 0 0
0 0 0 0 0 0 0 0 0 0 0 0 0
0 0 0 0 0 0 0 0 0 0 0 0 0
0 0 0 0 0 0 0 0 0 0 1 0 0

0 0 0 0 0 0 0 0 0 0 0 0 0
0 0 0 0 0 0 0 0
"Viruses;Uroviricota;Caudoviricetes;Caudoviricetes__na;Caudoviricetes__na;Caudoviricetes__na;Siphoviridae_sp._ctc6d98" 0 0 0 0 0 0 0 0 0 0 0 0
0 0 0 0 0 0 0 0 0 0 0 0 1
0 0 0 0 0 0 0 0 0 0 0 0 0
0 0 0 0 0 0 0 0 0 0 0 0 0
0 0 0 0 0 0 0 0 0 0 0 0 0
0 0 0 0 0 0 0 0
"Viruses;Uroviricota;Caudoviricetes;Caudoviricetes__na;Caudoviricetes__na;Caudoviricetes__na;Siphoviridae_sp._ctCb814" 0 0 0 0 0 0 0 0 0 0 0 0
0 0 0 0 0 0 0 0 0 0 0 0 0
0 0 0 0 0 0 0 0 0 0 0 3 3
1 0 0 0 0 0 0 0 0 0 0 0 0
0 2 0 0 0 0 0 0 0 0 0 0 0
0 0 0 0 0 0 0 0
"Viruses;Uroviricota;Caudoviricetes;Caudoviricetes__na;Caudoviricetes__na;Caudoviricetes__na;Siphoviridae_sp._ctCCX1" 0 0 1 0 0 0 0 0 1 0 0 0
25 17 9 4 0 1 10 9 4 3 1 2 11
4 1 0 0 0 4 1 0 0 0 0 9 8
0 0 0 0 53 34 1 0 0 0 7 6 1
0 0 0 2 4 0 0 0 0 25 3 0 0
0 0 33 17 0 5 0 0
"Viruses;Uroviricota;Caudoviricetes;Caudoviricetes__na;Caudoviricetes__na;Caudoviricetes__na;Siphoviridae_sp._ctCIv11" 0 0 0 0 0 0 0 0 0 0 0 0
0 0 0 0 0 0 3 19 1 0 16 1 0
0 0 0 0 0 0 1 0 0 0 0 0 1
0 0 0 0 0 0 0 0 0 0 0 0 0
0 0 0 0 1 0 0 0 0 19 15 1 0
0 0 1 3 0 0 0 0
"Viruses;Uroviricota;Caudoviricetes;Caudoviricetes__na;Caudoviricetes__na;Caudoviricetes__na;Siphoviridae_sp._ctcj91" 0 0 0 0 0 0 0 0 0 0 0 0
1 0 0 0 0 2 1 0 0 0 0 0 0
1 0 0 0 0 0 0 0 0 0 0 0 0
0 0 0 0 0 0 0 0 27 0 1 2 0
0 0 0 1 1 0 0 0 0 0 0 0 0
0 0 0 0 0 0 0 0
"Viruses;Uroviricota;Caudoviricetes;Caudoviricetes__na;Caudoviricetes__na;Caudoviricetes__na;Siphoviridae_sp._ctCjJ10" 0 0 0 0 0 0 0 0 0 0 0 0
1 0 0 0 0 0 0 0 0 0 0 0 0
0 0 0 2 0 0 1 0 0 0 0 2 2
0 0 0 0 0 0 0 0 0 0 1 0 0
0 0 0 1 0 0 0 0 0 0 0 0 0
0 0 0 0 0 0 0 0
"Viruses;Uroviricota;Caudoviricetes;Caudoviricetes__na;Caudoviricetes__na;Caudoviricetes__na;Siphoviridae_sp._ctcK97" 0 0 0 0 0 0 0 0 0 0 0 0
0 0 0 0 0 0 0 0 0 0 0 0 0
0 0 0 0 0 0 0 0 0 0 0 0 0
0 0 1 0 0 0 19 3 0 0 5 1 10

0 0 0 0 0 0 0 0 0 1 0 0 2
0 0 0 0 0 0 1 0
"Viruses;Uroviricota;Caudoviricetes;Caudoviricetes__na;Caudoviricetes__na;Caudoviricetes__na;Siphoviridae_s
p._ctClL93" 0 0 0 0 0 0 0 0 0 0 0 0
0 0 0 0 1 0 0 0 0 0 0 0 0
0 0 0 0 0 0 0 0 0 0 0 0 0
0 0 0 0 0 0 0 0 0 0 0 0 0
0 0 0 0 0 0 0 0 0 0 0 0 0
0 0 0 0 0 0 0 0
"Viruses;Uroviricota;Caudoviricetes;Caudoviricetes__na;Caudoviricetes__na;Caudoviricetes__na;Siphoviridae_s
p._ctCsv15" 0 0 0 0 0 0 0 0 0 0 0 0
0 0 0 0 0 0 0 0 0 0 0 0 0
0 0 0 0 0 0 0 0 0 0 0 0 0
0 0 0 0 0 0 0 0 0 0 0 0 0
0 0 0 2 2 0 0 0 0 0 0 0 0
0 0 0 0 0 0 0 0
"Viruses;Uroviricota;Caudoviricetes;Caudoviricetes__na;Caudoviricetes__na;Caudoviricetes__na;Siphoviridae_s
p._ctcUB23" 0 0 0 0 0 0 0 0 0 0 0 0
0 0 2 0 4 0 0 0 0 0 0 0 0
0 0 0 0 0 0 0 0 0 0 0 0 0
0 0 0 0 0 0 0 0 0 0 2 1 0
0 0 0 0 0 0 0 0 0 0 0 0 0
0 0 0 0 0 0 0 0
"Viruses;Uroviricota;Caudoviricetes;Caudoviricetes__na;Caudoviricetes__na;Caudoviricetes__na;Siphoviridae_s
p._ctCUc43" 0 0 0 0 0 0 0 0 0 0 0 0
0 0 0 0 0 0 0 0 0 0 0 0 0
0 0 0 0 0 0 0 0 0 0 0 0 0
0 0 0 0 0 0 0 0 0 0 0 0 0
0 0 0 0 1 0 0 0 0 0 0 0 0
0 0 0 0 0 0 0 0
"Viruses;Uroviricota;Caudoviricetes;Caudoviricetes__na;Caudoviricetes__na;Caudoviricetes__na;Siphoviridae_s
p._ctcuE16" 0 0 0 0 0 0 0 0 0 0 0 0
0 0 0 0 0 0 0 0 0 0 0 0 0
0 0 0 0 0 0 0 0 0 0 0 39 10
0 0 0 0 0 0 0 0 0 0 0 0 0
0 0 0 0 0 0 0 0 0 0 0 0 0
0 0 0 0 0 0 0 0
"Viruses;Uroviricota;Caudoviricetes;Caudoviricetes__na;Caudoviricetes__na;Caudoviricetes__na;Siphoviridae_s
p._ctcx61" 0 0 0 0 0 0 0 0 0 0 0 0
0 0 0 0 0 0 0 0 0 0 0 0 0
0 0 0 0 0 0 0 0 0 0 0 0 0
0 0 0 0 0 0 0 0 0 0 0 0 0
0 0 0 0 0 0 0 0 0 111 13 34 86
4 0 0 0 0 0 0 0
"Viruses;Uroviricota;Caudoviricetes;Caudoviricetes__na;Caudoviricetes__na;Caudoviricetes__na;Siphoviridae_s
p._ctD2Q91" 0 0 0 0 0 0 0 0 0 0 0 0
0 0 0 0 0 0 0 0 0 0 0 0 0
0 0 0 0 0 0 0 0 0 0 0 0 0
0 0 0 17 0 0 0 0 0 0 0 0 0

0 0 0 0 0 0 0 0 0 0 0 0 0
0 0 0 0 0 0 0 0
"Viruses;Uroviricota;Caudoviricetes;Caudoviricetes__na;Caudoviricetes__na;Caudoviricetes__na;Siphoviridae_s
p._ctD6g5" 0 0 0 0 0 0 0 0 0 0 0 0
1 0 0 0 0 0 0 0 0 0 0 0 0
0 0 0 0 0 3 0 0 0 0 0 0 0
0 0 0 0 0 0 0 0 0 0 0 0 0
0 0 0 11 13 0 0 0 0 0 0 0 0
0 0 0 0 0 0 0 0
"Viruses;Uroviricota;Caudoviricetes;Caudoviricetes__na;Caudoviricetes__na;Caudoviricetes__na;Siphoviridae_s
p._ctd9R8" 0 0 0 0 0 0 0 0 0 0 0 0
0 0 0 0 0 0 0 0 0 0 0 0 0
0 0 0 0 0 0 0 0 0 0 0 6 1
0 0 0 0 0 0 0 0 0 0 0 0 0
0 0 0 0 0 0 0 0 0 0 0 0 0
0 0 0 1 0 3 0 0
"Viruses;Uroviricota;Caudoviricetes;Caudoviricetes__na;Caudoviricetes__na;Caudoviricetes__na;Siphoviridae_s
p._ctdau33" 0 0 0 0 0 0 0 0 0 0 0 0
0 0 0 0 0 0 0 0 0 0 0 0 0
0 0 0 0 0 0 0 0 0 0 0 7 3
0 1 0 5 0 0 0 0 0 0 0 0 0
0 0 0 0 0 0 0 0 0 0 0 0 0
0 0 0 0 0 0 0 0
"Viruses;Uroviricota;Caudoviricetes;Caudoviricetes__na;Caudoviricetes__na;Caudoviricetes__na;Siphoviridae_s
p._ctDCt3" 0 0 0 0 0 0 0 0 0 0 0 0
0 0 0 0 0 0 0 0 0 0 0 0 0
0 0 0 0 0 0 0 0 0 0 0 0 0
0 0 0 0 0 0 0 0 0 0 0 0 0
0 0 0 0 0 0 0 0 0 0 0 0 0
0 0 1 2 0 0 0 0
"Viruses;Uroviricota;Caudoviricetes;Caudoviricetes__na;Caudoviricetes__na;Caudoviricetes__na;Siphoviridae_s
p._ctDcW16" 1 0 0 0 0 0 0 0 0 0 0 0
0 1 0 0 0 0 1 0 1 0 0 0 0
0 0 0 0 0 0 0 0 0 0 0 0 1
0 0 1 0 7 8 3 0 0 0 1 0 0
0 0 0 0 1 0 0 0 0 0 0 0 0
0 0 1 2 0 0 0 0
"Viruses;Uroviricota;Caudoviricetes;Caudoviricetes__na;Caudoviricetes__na;Caudoviricetes__na;Siphoviridae_s
p._ctDDY10" 0 0 0 0 0 0 0 0 0 0 0 0
0 0 0 0 0 0 0 0 0 0 0 0 0
0 0 0 0 0 0 0 0 0 0 0 1 1
0 0 0 0 0 0 0 0 0 0 0 0 0
0 0 0 4 0 0 0 0 0 0 1 0 0
0 0 0 0 0 0 0 0
"Viruses;Uroviricota;Caudoviricetes;Caudoviricetes__na;Caudoviricetes__na;Caudoviricetes__na;Siphoviridae_s
p._ctdEk19" 0 0 0 3 39 18 0 0 0 0 0 0
0 0 0 0 0 1 0 0 0 0 0 0 0
0 2 0 0 0 0 0 0 0 0 0 0 0
0 0 0 1 0 0 0 0 0 0 0 0 0

0 0 0 0 0 0 0 0 0 0 0 0 0
0 0 0 0 0 0 0 0
"Viruses;Uroviricota;Caudoviricetes;Caudoviricetes__na;Caudoviricetes__na;Caudoviricetes__na;Siphoviridae_sp._ctDEW4" 0 0 0 0 0 0 0 0 0 0 0 0
0 0 0 0 0 0 0 0 0 0 0 0 0
0 0 0 0 0 0 0 0 0 0 1 0 0
0 0 0 0 0 0 0 0 0 0 0 0 0
0 0 0 0 0 0 0 0 0 0 0 0 0
0 0 0 0 0 0 0 0
"Viruses;Uroviricota;Caudoviricetes;Caudoviricetes__na;Caudoviricetes__na;Caudoviricetes__na;Siphoviridae_sp._ctdHi7" 0 0 0 0 0 0 0 0 0 0 0 0
0 0 0 0 0 0 0 0 0 0 0 0 0
0 0 0 0 0 0 0 0 0 0 0 0 0
0 0 0 0 0 0 0 0 0 0 0 0 0
0 0 0 0 3 1 0 0 0 0 0 0 0
0 0 0 1 0 0 0 0
"Viruses;Uroviricota;Caudoviricetes;Caudoviricetes__na;Caudoviricetes__na;Caudoviricetes__na;Siphoviridae_sp._ctDhw1" 0 0 0 0 0 0 0 0 0 0 0 0
18 14 17 27 163 385 0 0 0 0 8 0 0
0 0 0 0 0 0 0 1 0 38 0 0 0
0 0 0 0 0 0 0 0 0 0 0 0 0
1 0 0 0 0 0 0 0 0 0 0 0 0
0 0 0 0 0 0 0 0
"Viruses;Uroviricota;Caudoviricetes;Caudoviricetes__na;Caudoviricetes__na;Caudoviricetes__na;Siphoviridae_sp._ctdLA8" 0 0 0 0 0 0 0 0 0 0 0 0
0 0 0 0 0 0 0 0 0 0 0 0 0
0 0 0 0 0 0 0 0 0 0 0 0 0
0 0 0 0 0 0 0 0 0 0 0 0 0
0 0 0 1 0 0 0 0 0 0 0 0 0
0 0 0 0 0 0 0 0
"Viruses;Uroviricota;Caudoviricetes;Caudoviricetes__na;Caudoviricetes__na;Caudoviricetes__na;Siphoviridae_sp._ctDlU28" 0 0 0 0 0 0 0 0 0 0 0 0
1 0 0 0 1 4 1 2 0 0 0 0 0
0 0 0 0 0 2 1 0 0 0 0 0 0
0 0 0 0 0 0 0 0 0 0 0 0 0
0 0 0 0 0 0 0 0 0 0 0 0 0
0 0 0 0 0 0 0 0
"Viruses;Uroviricota;Caudoviricetes;Caudoviricetes__na;Caudoviricetes__na;Caudoviricetes__na;Siphoviridae_sp._ctdm01" 0 0 0 0 0 0 0 0 0 0 0 0
0 0 0 0 0 0 0 0 0 0 0 0 0
0 0 0 0 0 0 0 0 0 0 0 0 0
0 0 0 0 0 0 0 0 0 0 0 0 0
0 0 0 0 0 0 0 0 0 0 0 0 0
0 0 3 3 0 0 0 0
"Viruses;Uroviricota;Caudoviricetes;Caudoviricetes__na;Caudoviricetes__na;Caudoviricetes__na;Siphoviridae_sp._ctDMf1" 0 0 0 0 0 0 0 0 0 0 0 0
0 0 0 0 0 0 0 0 0 0 0 0 0
0 0 0 0 0 0 0 0 0 0 0 0 0
0 0 0 0 0 0 0 0 0 0 0 0 0

0 0 1 0 0 0 0 0 0 0 0 0 0
0 0 0 0 0 0 0 0
"Viruses;Uroviricota;Caudoviricetes;Caudoviricetes__na;Caudoviricetes__na;Caudoviricetes__na;Siphoviridae_s
p._ctDmQ3" 0 0 0 0 0 0 0 0 0 0 0 0
0 0 0 0 0 0 0 0 0 0 0 0 0
9 0 0 0 0 0 0 0 0 0 0 0 0
0 0 0 0 0 0 0 0 0 0 15 28 0
1 0 0 3 3 0 0 0 0 0 0 0 0
0 0 1 0 0 0 0 0
"Viruses;Uroviricota;Caudoviricetes;Caudoviricetes__na;Caudoviricetes__na;Caudoviricetes__na;Siphoviridae_s
p._ctdmY20" 0 0 0 0 0 0 0 0 0 0 0 0
0 0 0 0 0 0 0 0 0 0 0 0 9
23 0 0 0 0 0 0 0 0 0 0 1 1
0 0 0 0 0 0 0 0 0 0 29 30 0
0 0 0 1 0 0 0 0 0 0 0 0 0
0 0 2 16 0 4 0 0
"Viruses;Uroviricota;Caudoviricetes;Caudoviricetes__na;Caudoviricetes__na;Caudoviricetes__na;Siphoviridae_s
p._ctDo63" 0 0 0 0 0 0 0 0 0 0 0 0
0 0 0 0 0 0 0 0 0 0 0 0 0
0 0 0 0 0 1 1 0 0 0 0 0 0
0 0 0 0 0 0 0 0 0 0 0 0 0
0 0 0 0 0 0 0 0 0 0 0 0 0
0 0 0 1 0 0 0 0
"Viruses;Uroviricota;Caudoviricetes;Caudoviricetes__na;Caudoviricetes__na;Caudoviricetes__na;Siphoviridae_s
p._ctDsE1" 0 0 0 0 0 0 0 0 0 0 0 0
0 0 0 0 0 0 0 0 0 0 0 0 0
0 0 0 0 0 0 0 0 0 0 0 0 0
0 0 0 0 0 0 0 0 0 0 0 0 0
0 0 1 0 0 0 0 0 0 0 0 0 0
0 0 0 0 0 1 0 0
"Viruses;Uroviricota;Caudoviricetes;Caudoviricetes__na;Caudoviricetes__na;Caudoviricetes__na;Siphoviridae_s
p._ctdvJ3" 0 0 0 0 0 0 0 0 0 0 0 0
0 0 0 0 0 0 1 0 0 0 0 0 1
1 5 0 0 0 0 0 0 0 0 0 0 0
0 0 0 0 0 0 0 0 0 0 0 0 0
0 0 0 1 9 0 0 0 0 0 0 0 0
0 0 0 1 0 0 0 0
"Viruses;Uroviricota;Caudoviricetes;Caudoviricetes__na;Caudoviricetes__na;Caudoviricetes__na;Siphoviridae_s
p._ctDXu9" 0 0 0 0 0 0 0 0 0 0 0 0
0 12 0 0 0 5 0 0 0 0 0 0 0
0 0 0 0 0 0 0 0 0 0 0 0 0
0 0 0 0 0 2 0 0 0 0 2 7 0
0 0 0 0 0 0 0 1 0 0 0 0 0
0 0 0 0 0 0 2 0
"Viruses;Uroviricota;Caudoviricetes;Caudoviricetes__na;Caudoviricetes__na;Caudoviricetes__na;Siphoviridae_s
p._ctdYc1" 0 0 0 0 0 0 0 0 0 0 0 0
0 0 0 0 0 0 0 0 0 0 0 0 0
0 0 0 0 0 27 45 0 0 1 0 0 0
0 0 0 0 0 0 0 0 0 0 0 0 0

0 0 0 1 0 0 0 0 0 0 0 0 0
0 0 0 0 0 0 0 0
"Viruses;Uroviricota;Caudoviricetes;Caudoviricetes__na;Caudoviricetes__na;Caudoviricetes__na;Siphoviridae_s
p._ctdzB12" 0 0 0 0 0 0 0 0 0 0 0 0
0 0 0 0 0 0 0 0 0 0 0 0 0
0 0 0 0 0 0 0 0 0 0 0 0 0
0 0 0 0 0 0 0 0 0 0 1 0 0
0 0 0 0 0 0 0 0 0 0 0 0 0
0 0 0 0 0 0 0 0
"Viruses;Uroviricota;Caudoviricetes;Caudoviricetes__na;Caudoviricetes__na;Caudoviricetes__na;Siphoviridae_s
p._cte421" 0 0 0 0 0 0 0 0 0 0 0 0
1 0 0 0 0 0 0 0 0 0 0 0 0
0 0 0 0 0 0 0 0 0 0 0 0 0
0 0 0 0 0 0 0 0 0 0 0 0 0
0 0 0 0 0 0 0 0 0 0 0 0 0
0 0 0 0 0 0 0 0
"Viruses;Uroviricota;Caudoviricetes;Caudoviricetes__na;Caudoviricetes__na;Caudoviricetes__na;Siphoviridae_s
p._ctE6L85" 0 0 0 0 0 0 0 0 0 0 0 0
4 4 2 2 7 0 0 0 0 0 0 0 0
0 0 0 0 0 11 19 0 0 0 0 2 0
0 0 0 0 1 0 0 0 0 0 0 0 0
0 0 0 49 69 0 0 0 0 0 0 0 0
0 0 9 16 0 1 0 0
"Viruses;Uroviricota;Caudoviricetes;Caudoviricetes__na;Caudoviricetes__na;Caudoviricetes__na;Siphoviridae_s
p._ctedi74" 0 0 0 0 0 0 0 0 0 0 0 0
1 0 0 0 0 0 0 0 0 0 0 0 0
0 0 0 0 0 0 1 0 0 0 0 0 0
0 0 0 0 0 0 0 0 0 0 0 0 0
0 0 0 0 0 0 0 0 0 0 0 0 0
0 0 0 0 0 0 0 0
"Viruses;Uroviricota;Caudoviricetes;Caudoviricetes__na;Caudoviricetes__na;Caudoviricetes__na;Siphoviridae_s
p._cteDy1" 1 0 0 0 0 0 0 0 0 0 0 0
0 1 1 0 0 0 6 6 5 2 0 0 0
0 0 0 0 0 1 1 0 0 0 0 0 2
0 0 0 0 14 9 0 0 0 0 0 0 0
0 0 0 4 1 0 0 0 0 39 12 0 0
0 0 0 0 0 0 0 0
"Viruses;Uroviricota;Caudoviricetes;Caudoviricetes__na;Caudoviricetes__na;Caudoviricetes__na;Siphoviridae_s
p._cteEJ17" 0 1 0 0 0 0 0 0 0 0 0 0
0 0 0 0 0 0 0 0 0 0 0 0 0
0 0 0 0 0 1 0 0 0 0 0 0 0
0 0 0 0 0 0 0 0 0 0 0 0 0
0 0 0 0 0 0 0 0 0 0 0 0 0
0 0 2 3 0 1 0 0
"Viruses;Uroviricota;Caudoviricetes;Caudoviricetes__na;Caudoviricetes__na;Caudoviricetes__na;Siphoviridae_s
p._ctEEM24" 0 0 0 0 0 0 0 0 0 0 0 0
0 0 0 0 0 0 0 0 0 0 0 0 0
0 0 0 0 0 0 0 0 0 0 0 0 0
0 0 0 0 0 0 0 0 0 0 2 4 0

0 0 0 0 0 0 0 0 0 0 0 0 0
0 0 0 2 0 0 0 0
"Viruses;Uroviricota;Caudoviricetes;Caudoviricetes__na;Caudoviricetes__na;Caudoviricetes__na;Siphoviridae_s
p._cteEQ43" 0 0 0 0 0 0 0 0 0 0 0 0
0 0 0 0 0 0 0 0 0 0 0 0 0
0 0 0 0 0 0 0 0 0 0 0 1 0
0 0 0 0 0 0 0 0 0 0 0 0 0
0 0 0 0 0 0 0 0 0 0 0 0 0
0 0 0 0 0 0 0 0
"Viruses;Uroviricota;Caudoviricetes;Caudoviricetes__na;Caudoviricetes__na;Caudoviricetes__na;Siphoviridae_s
p._ctEfY6" 0 0 0 0 0 0 0 0 0 0 0 0
1 2 0 1 0 2 2 0 0 0 0 0 0
0 0 0 0 0 0 0 0 0 0 0 0 0
0 0 0 0 10 4 0 0 0 0 0 0 0
0 0 0 1 0 0 0 0 0 1 0 0 0
0 0 0 0 0 0 0 0
"Viruses;Uroviricota;Caudoviricetes;Caudoviricetes__na;Caudoviricetes__na;Caudoviricetes__na;Siphoviridae_s
p._cteHV32" 0 0 0 0 0 0 0 0 0 0 0 0
0 0 0 0 0 0 0 0 0 0 0 0 0
2 0 0 0 0 0 0 0 0 0 0 0 0
0 0 0 0 0 0 0 0 0 0 0 0 0
0 0 0 0 0 0 0 0 0 0 0 0 0
0 0 0 0 0 0 0 0
"Viruses;Uroviricota;Caudoviricetes;Caudoviricetes__na;Caudoviricetes__na;Caudoviricetes__na;Siphoviridae_s
p._ctEIp38" 0 0 0 0 0 0 0 0 0 0 0 0
0 0 0 0 0 0 0 0 0 0 0 0 0
0 0 0 0 0 1 2 0 0 0 0 0 0
0 0 0 0 0 0 0 0 0 0 0 0 0
0 0 0 0 0 0 0 0 0 0 0 0 0
0 0 0 0 0 0 0 0
"Viruses;Uroviricota;Caudoviricetes;Caudoviricetes__na;Caudoviricetes__na;Caudoviricetes__na;Siphoviridae_s
p._ctEJG5" 0 0 0 0 0 0 0 0 0 0 0 0
0 0 0 0 0 0 0 0 0 0 0 0 1
0 0 0 0 0 0 0 0 0 0 0 0 0
0 0 0 0 0 1 0 0 0 0 0 0 0
0 0 0 0 0 0 0 0 0 1 0 0 0
0 0 0 1 0 0 0 0
"Viruses;Uroviricota;Caudoviricetes;Caudoviricetes__na;Caudoviricetes__na;Caudoviricetes__na;Siphoviridae_s
p._ctEJj1" 0 0 0 0 0 0 0 0 0 0 0 0
0 0 0 0 0 0 4 7 2 0 0 0 0
1 0 0 0 0 0 0 0 0 0 0 1 2
0 0 0 0 0 5 0 0 0 0 0 0 0
0 0 0 0 0 0 0 0 0 3 1 0 0
0 0 0 4 0 0 0 0
"Viruses;Uroviricota;Caudoviricetes;Caudoviricetes__na;Caudoviricetes__na;Caudoviricetes__na;Siphoviridae_s
p._ctEkS11" 0 0 0 0 0 0 0 0 0 0 0 0
0 0 0 0 0 0 0 0 0 0 0 0 0
0 0 0 0 0 0 0 0 0 0 0 0 0
0 0 0 0 0 0 1 0 0 0 0 0 0

0	0	0	0	0	0	0	0	0	0	0	0	0
0	0	1	2	0	0	0	0
"Viruses;Uroviricota;Caudoviricetes;Caudoviricetes__na;Caudoviricetes__na;Caudoviricetes__na;Siphoviridae_s
p._ctENB54"	0	0	0	0	0	0	0	0	0	0	0	0
0	0	0	0	0	0	0	0	0	0	1	0	0
0	0	0	0	0	0	0	0	0	0	0	0	0
0	0	0	0	0	0	0	0	0	0	0	0	0
0	0	0	0	0	0	0	0	0	0	0	0	0
0	0	0	0	0	0	0	0
"Viruses;Uroviricota;Caudoviricetes;Caudoviricetes__na;Caudoviricetes__na;Caudoviricetes__na;Siphoviridae_s
p._cteNz1"	0	0	0	0	0	0	0	0	0	0	0	0
0	0	0	0	0	0	0	0	0	0	0	0	0
0	0	0	0	0	0	0	0	0	0	0	0	0
0	0	0	0	0	0	0	0	0	0	1	0	0
0	0	0	0	0	0	0	0	0	0	0	0	0
0	0	0	0	0	0	0	0
"Viruses;Uroviricota;Caudoviricetes;Caudoviricetes__na;Caudoviricetes__na;Caudoviricetes__na;Siphoviridae_s
p._ctEqU3"	0	0	0	0	0	0	0	0	0	0	0	0
0	0	0	0	0	0	0	0	0	0	0	0	0
0	0	0	0	0	0	0	0	0	0	0	1	2
0	0	0	0	0	0	0	0	0	0	0	0	0
0	0	0	0	0	0	0	0	0	0	0	0	0
0	0	0	0	0	0	0	0
"Viruses;Uroviricota;Caudoviricetes;Caudoviricetes__na;Caudoviricetes__na;Caudoviricetes__na;Siphoviridae_s
p._ctES717"	0	0	0	0	0	0	0	0	0	0	0	0
0	0	0	0	0	0	0	1	0	0	0	0	0
0	0	0	0	0	0	0	0	0	0	0	0	0
0	0	0	0	0	0	0	0	0	0	0	0	0
0	0	0	24	25	0	0	0	0	0	0	0	0
0	0	0	0	0	0	0	0
"Viruses;Uroviricota;Caudoviricetes;Caudoviricetes__na;Caudoviricetes__na;Caudoviricetes__na;Siphoviridae_s
p._ctETQ12"	0	0	0	0	0	0	0	0	0	0	0	0
0	0	0	0	0	0	0	0	0	0	0	0	0
0	0	0	0	0	0	0	0	0	0	0	0	0
0	0	0	0	0	0	0	0	0	0	1	0	0
0	0	3	0	0	0	0	0	0	0	0	0	0
0	0	10	6	0	2	0	0
"Viruses;Uroviricota;Caudoviricetes;Caudoviricetes__na;Caudoviricetes__na;Caudoviricetes__na;Siphoviridae_s
p._ctF2K4"	0	0	0	0	0	0	0	0	0	0	0	0
0	0	0	0	0	0	0	0	0	0	0	0	0
0	0	0	0	0	0	0	0	0	0	0	0	0
0	0	0	0	0	0	0	0	0	0	0	0	0
0	0	0	0	1	0	0	0	0	0	0	0	0
0	0	0	0	0	0	0	0
"Viruses;Uroviricota;Caudoviricetes;Caudoviricetes__na;Caudoviricetes__na;Caudoviricetes__na;Siphoviridae_s
p._ctf8W5"	0	0	0	0	0	0	0	0	0	0	0	0
0	2	0	0	0	0	0	2	0	0	0	0	2
0	0	0	0	0	0	0	0	0	0	0	0	2
0	0	0	0	0	0	0	0	0	0	2	4	0

0 0 0 1 2 0 0 0 0 1 0 0 0
0 0 0 0 0 0 0 0
"Viruses;Uroviricota;Caudoviricetes;Caudoviricetes__na;Caudoviricetes__na;Caudoviricetes__na;Siphoviridae_sp._ctfbh2" 0 0 0 0 0 0 0 0 0 0 0 0
0 0 0 0 0 0 0 0 0 0 0 0 0
0 0 0 0 0 0 0 0 0 0 0 0 0
0 0 0 0 0 0 0 0 0 0 0 0 0
0 0 0 9 16 0 0 0 0 1 1 0 0
0 0 7 4 0 0 0 0
"Viruses;Uroviricota;Caudoviricetes;Caudoviricetes__na;Caudoviricetes__na;Caudoviricetes__na;Siphoviridae_sp._ctfDm7" 0 0 0 0 0 0 0 0 0 0 0 0
0 0 0 0 0 0 0 0 0 0 0 0 0
0 0 0 0 0 0 0 0 0 0 0 0 0
0 0 0 0 0 0 0 0 0 0 0 0 0
0 0 2 0 0 0 0 0 0 0 0 0 0
0 0 0 0 0 0 0 0
"Viruses;Uroviricota;Caudoviricetes;Caudoviricetes__na;Caudoviricetes__na;Caudoviricetes__na;Siphoviridae_sp._ctfeV1" 0 0 0 0 0 0 0 0 0 0 0 0
5 3 0 1 0 0 5 6 2 0 0 0 5
7 1 0 1 0 4 5 0 0 0 0 0 2
0 0 0 0 43 28 15 1 0 0 2 0 0
0 0 1 0 0 0 0 0 0 149 36 9 11
1 0 10 4 1 0 0 0
"Viruses;Uroviricota;Caudoviricetes;Caudoviricetes__na;Caudoviricetes__na;Caudoviricetes__na;Siphoviridae_sp._ctFIm6" 0 0 0 0 0 0 0 0 0 0 0 0
0 0 0 0 0 0 0 0 0 0 0 0 0
0 0 0 0 0 0 0 0 0 0 0 1 0
0 0 0 0 0 0 0 0 0 0 0 0 0
0 0 0 0 0 0 0 0 0 0 0 0 0
0 0 0 0 0 0 0 0
"Viruses;Uroviricota;Caudoviricetes;Caudoviricetes__na;Caudoviricetes__na;Caudoviricetes__na;Siphoviridae_sp._ctFn287" 0 0 0 0 0 0 0 0 0 0 0 0
0 0 0 0 0 0 0 0 0 0 0 0 0
0 0 0 0 0 0 1 0 0 0 0 0 0
0 0 0 0 0 0 0 0 0 0 0 0 0
0 0 0 0 0 0 0 0 0 0 0 0 0
0 0 0 0 0 0 0 0
"Viruses;Uroviricota;Caudoviricetes;Caudoviricetes__na;Caudoviricetes__na;Caudoviricetes__na;Siphoviridae_sp._ctFPV4" 0 0 0 0 0 0 0 0 0 0 0 0
0 0 0 0 0 2 0 0 0 0 0 0 0
0 0 0 0 0 0 0 0 0 0 0 0 0
0 0 0 0 0 0 0 0 0 0 0 0 0
0 0 0 0 0 0 0 0 0 1 1 0 0
0 0 0 0 0 0 0 0
"Viruses;Uroviricota;Caudoviricetes;Caudoviricetes__na;Caudoviricetes__na;Caudoviricetes__na;Siphoviridae_sp._ctFSL3" 0 0 0 0 0 0 0 0 0 0 0 0
0 1 0 0 0 0 0 0 0 0 0 0 0
0 0 0 0 0 0 0 0 0 0 0 1 0
0 0 0 0 0 0 0 0 0 0 0 0 0

0 0 0 3 2 0 0 0 0 0 0 0 0
0 0 0 0 0 0 0 0
"Viruses;Uroviricota;Caudoviricetes;Caudoviricetes__na;Caudoviricetes__na;Caudoviricetes__na;Siphoviridae_sp._ctfza2" 0 0 0 0 0 0 0 0 0 0 0 0
0 0 0 0 2 0 0 0 0 0 0 0 3
4 0 0 0 0 0 0 0 0 0 0 0 1
0 0 0 0 0 0 0 0 0 0 0 0 0
0 0 0 0 2 0 0 0 0 0 0 0 0
0 0 0 0 0 0 0 0
"Viruses;Uroviricota;Caudoviricetes;Caudoviricetes__na;Caudoviricetes__na;Caudoviricetes__na;Siphoviridae_sp._ctg0K17" 0 0 0 0 0 0 0 0 0 0 0 0
0 0 0 0 0 0 0 0 0 0 0 0 0
0 0 0 0 0 0 0 0 0 0 0 12 8
0 2 0 0 0 0 0 0 0 0 0 0 0
0 0 0 0 3 0 0 0 0 0 0 0 0
0 0 9 18 0 1 0 0
"Viruses;Uroviricota;Caudoviricetes;Caudoviricetes__na;Caudoviricetes__na;Caudoviricetes__na;Siphoviridae_sp._ctG7D9" 0 0 0 0 0 0 0 0 0 0 0 0
0 0 0 0 0 0 0 0 0 0 0 0 0
0 0 0 0 0 0 0 0 0 0 0 0 0
0 0 0 0 1 0 0 0 0 0 0 0 0
0 0 0 0 0 0 0 0 0 0 0 0 0
0 0 0 0 0 0 0 0
"Viruses;Uroviricota;Caudoviricetes;Caudoviricetes__na;Caudoviricetes__na;Caudoviricetes__na;Siphoviridae_sp._ctg8V11" 0 0 0 0 0 0 0 0 0 0 0 0
0 0 0 0 0 0 0 0 0 0 0 0 0
0 0 0 0 0 0 0 0 0 0 0 0 0
0 0 0 0 0 0 0 0 0 0 0 0 0
0 0 0 1 3 0 0 0 0 0 0 0 0
0 0 2 1 0 1 0 0
"Viruses;Uroviricota;Caudoviricetes;Caudoviricetes__na;Caudoviricetes__na;Caudoviricetes__na;Siphoviridae_sp._ctGa111" 0 0 0 0 0 0 0 0 0 0 0 0
0 0 0 0 0 0 0 0 0 0 0 0 0
0 0 0 0 0 0 0 0 0 0 0 3 5
0 0 0 0 0 0 0 0 0 0 0 0 1
0 0 0 0 0 0 0 0 0 0 0 0 0
0 0 0 0 0 0 0 0
"Viruses;Uroviricota;Caudoviricetes;Caudoviricetes__na;Caudoviricetes__na;Caudoviricetes__na;Siphoviridae_sp._ctGas1" 0 0 0 0 0 0 0 0 0 0 0 0
0 0 0 0 0 0 0 0 0 0 0 0 0
0 0 0 0 0 0 0 0 0 0 0 0 0
0 0 0 0 0 0 1 0 0 0 0 0 0
0 0 0 0 2 0 0 0 0 0 0 0 0
0 0 0 0 0 0 0 0
"Viruses;Uroviricota;Caudoviricetes;Caudoviricetes__na;Caudoviricetes__na;Caudoviricetes__na;Siphoviridae_sp._ctgaY24" 0 0 0 0 0 0 0 0 0 0 0 0
1 0 1 0 9 0 0 0 0 0 0 0 0
0 0 0 0 0 0 0 0 0 0 0 0 8
0 0 0 0 0 0 0 0 0 0 0 0 0

0 0 0 0 0 0 0 0 0 0 0 0 0
0 0 0 0 0 0 0 0
"Viruses;Uroviricota;Caudoviricetes;Caudoviricetes__na;Caudoviricetes__na;Caudoviricetes__na;Siphoviridae_s
p._ctgBD49" 0 0 0 3 2 0 0 0 0 0 0 0
1 0 0 0 0 0 0 0 0 0 0 0 0
0 0 0 0 0 0 0 0 0 0 0 0 0
0 0 0 0 0 0 0 0 0 0 0 0 0
0 1 0 0 0 0 0 0 0 0 0 0 0
0 0 0 0 0 0 0 0
"Viruses;Uroviricota;Caudoviricetes;Caudoviricetes__na;Caudoviricetes__na;Caudoviricetes__na;Siphoviridae_s
p._ctgbm9" 0 0 0 0 0 0 0 0 0 0 0 0
0 0 0 0 0 0 0 0 0 0 0 0 0
0 0 0 0 0 6 1 0 0 0 0 0 0
0 0 0 0 0 0 0 0 0 0 0 0 0
0 0 0 0 0 0 0 0 0 0 0 0 0
0 0 0 0 0 0 0 0
"Viruses;Uroviricota;Caudoviricetes;Caudoviricetes__na;Caudoviricetes__na;Caudoviricetes__na;Siphoviridae_s
p._ctGDt6" 0 0 0 0 0 0 0 0 0 0 0 0
0 0 0 0 0 0 0 0 0 0 0 0 0
0 0 0 0 0 2 0 0 0 0 0 0 1
0 0 0 0 0 1 10 0 0 0 0 0 0
0 0 21 0 0 0 0 0 0 0 0 0 0
0 0 2 8 0 0 0 0
"Viruses;Uroviricota;Caudoviricetes;Caudoviricetes__na;Caudoviricetes__na;Caudoviricetes__na;Siphoviridae_s
p._ctGFb30" 0 0 0 0 0 0 0 0 0 0 0 0
0 0 0 0 0 0 0 0 0 0 0 0 1
0 0 0 0 0 0 0 0 0 0 0 2 0
0 0 0 0 0 0 0 0 0 0 0 0 0
0 0 0 0 3 0 0 0 0 0 0 0 0
0 0 0 0 0 0 0 0
"Viruses;Uroviricota;Caudoviricetes;Caudoviricetes__na;Caudoviricetes__na;Caudoviricetes__na;Siphoviridae_s
p._ctgmM3" 0 0 0 0 0 0 0 0 0 0 0 0
0 0 0 0 0 0 0 0 0 0 0 0 0
0 0 0 0 0 0 0 0 0 2 0 0 0
0 0 0 0 0 1 0 0 0 0 0 0 0
0 0 0 0 0 0 0 0 0 0 0 0 0
0 0 0 0 0 0 0 0
"Viruses;Uroviricota;Caudoviricetes;Caudoviricetes__na;Caudoviricetes__na;Caudoviricetes__na;Siphoviridae_s
p._ctGMq5" 0 0 0 0 0 0 0 0 0 0 0 0
0 0 0 0 0 0 0 0 0 0 0 0 0
0 0 0 0 0 0 0 0 0 0 0 0 1
0 0 0 0 0 0 0 0 0 0 0 0 0
0 0 0 0 0 0 0 0 0 0 0 0 0
0 0 0 0 0 0 0 0
"Viruses;Uroviricota;Caudoviricetes;Caudoviricetes__na;Caudoviricetes__na;Caudoviricetes__na;Siphoviridae_s
p._ctgN495" 0 0 0 0 0 0 0 0 0 0 0 0
0 0 0 0 0 0 0 0 0 0 0 0 0
0 0 0 0 0 0 0 0 0 0 0 0 0
0 0 0 0 0 0 0 0 0 0 0 0 0

0 0 663 0 0 0 0 0 0 0 0 0 0
0 0 0 0 0 3 0 0
"Viruses;Uroviricota;Caudoviricetes;Caudoviricetes__na;Caudoviricetes__na;Caudoviricetes__na;Siphoviridae_sp._ctGoR6" 0 0 0 0 0 0 0 0 0 0 0 0
0 0 0 0 0 0 0 0 0 0 0 0 0
0 0 0 0 0 0 0 0 0 0 0 0 0
0 0 0 0 0 0 0 0 0 0 0 0 0
0 0 0 0 0 0 0 0 0 0 0 0 0
0 0 1 0 0 0 0 0
"Viruses;Uroviricota;Caudoviricetes;Caudoviricetes__na;Caudoviricetes__na;Caudoviricetes__na;Siphoviridae_sp._ctGQT3" 0 0 0 0 0 0 0 0 0 0 0 0
0 0 0 0 0 0 0 0 0 0 0 0 0
0 0 0 0 0 0 0 0 0 0 0 0 0
0 0 0 0 0 0 0 0 0 0 0 0 0
0 0 0 5 2 0 0 0 0 0 0 0 0
0 0 0 0 0 0 0 0
"Viruses;Uroviricota;Caudoviricetes;Caudoviricetes__na;Caudoviricetes__na;Caudoviricetes__na;Siphoviridae_sp._ctgyq20" 0 0 0 0 0 0 0 0 0 0 0 0
0 0 0 0 0 0 0 1 0 0 0 0 0
0 0 0 0 0 0 0 0 0 0 0 0 0
0 0 0 0 0 1 0 0 0 0 0 0 0
0 0 0 0 0 0 0 0 0 0 0 0 0
0 0 0 1 0 0 0 0
"Viruses;Uroviricota;Caudoviricetes;Caudoviricetes__na;Caudoviricetes__na;Caudoviricetes__na;Siphoviridae_sp._ctGyV19" 0 0 0 0 0 0 0 0 0 0 0 0
0 0 0 0 0 0 0 0 0 0 0 0 0
0 0 0 0 0 0 0 0 0 0 0 2 0
0 0 0 0 0 0 0 0 0 0 0 0 0
0 0 0 0 0 0 0 0 0 0 0 0 0
0 0 0 0 0 0 0 0
"Viruses;Uroviricota;Caudoviricetes;Caudoviricetes__na;Caudoviricetes__na;Caudoviricetes__na;Siphoviridae_sp._ctH1110" 0 0 0 0 0 0 0 0 0 0 0 0
0 0 0 0 0 0 0 0 0 0 0 0 0
0 0 0 0 0 0 0 0 0 0 0 0 0
1 0 0 0 0 0 0 0 0 0 0 0 0
0 0 0 0 0 0 0 0 0 0 0 0 0
0 0 0 0 0 0 0 0
"Viruses;Uroviricota;Caudoviricetes;Caudoviricetes__na;Caudoviricetes__na;Caudoviricetes__na;Siphoviridae_sp._cth2082" 0 0 0 0 0 0 0 0 0 0 0 0
0 0 0 0 0 0 0 0 0 0 0 0 0
0 0 0 0 0 0 0 0 0 0 0 0 0
0 0 0 0 0 0 0 0 0 0 0 0 0
0 0 0 1 0 0 0 0 0 0 0 0 0
0 0 0 0 0 0 0 0
"Viruses;Uroviricota;Caudoviricetes;Caudoviricetes__na;Caudoviricetes__na;Caudoviricetes__na;Siphoviridae_sp._ctH8e11" 0 0 0 0 0 0 0 0 0 0 0 0
0 0 0 0 0 0 0 0 0 0 0 0 0
0 0 0 0 0 0 0 0 0 0 0 0 0
0 0 0 0 8 4 0 0 0 0 0 0 0

0 0 41 0 0 0 0 0 0 0 0 0 0
0 0 1 0 1 0 0 0
"Viruses;Uroviricota;Caudoviricetes;Caudoviricetes__na;Caudoviricetes__na;Caudoviricetes__na;Siphoviridae_sp._cthae16" 0 0 0 0 0 0 0 0 0 0 0 0
0 0 0 0 0 0 0 0 0 0 0 0 1
0 0 0 0 0 0 0 0 0 0 0 0 0
0 0 0 0 0 0 0 0 0 0 0 0 0
0 0 0 1 0 0 0 0 0 0 0 0 0
0 0 0 0 0 0 0 0
"Viruses;Uroviricota;Caudoviricetes;Caudoviricetes__na;Caudoviricetes__na;Caudoviricetes__na;Siphoviridae_sp._ctHAs12" 0 0 0 0 0 0 0 0 0 0 0 0
0 0 0 0 0 0 0 0 0 0 0 0 0
0 0 0 0 0 0 0 0 0 0 1 0 0
0 0 0 0 0 0 0 0 0 0 0 0 0
0 0 0 0 0 0 0 0 0 0 0 0 0
0 0 2 1 0 0 0 0
"Viruses;Uroviricota;Caudoviricetes;Caudoviricetes__na;Caudoviricetes__na;Caudoviricetes__na;Siphoviridae_sp._cthBp9" 0 0 0 0 0 0 0 0 0 0 0 0
0 0 0 0 0 0 0 0 0 0 0 0 1
0 0 0 0 0 0 0 0 0 0 0 0 0
0 0 0 0 0 0 0 0 0 0 0 0 0
0 0 0 0 0 0 0 0 0 0 0 0 0
0 0 0 0 0 0 0 0
"Viruses;Uroviricota;Caudoviricetes;Caudoviricetes__na;Caudoviricetes__na;Caudoviricetes__na;Siphoviridae_sp._cthEJ2" 0 0 0 0 0 0 0 0 0 0 0 0
0 0 0 0 0 0 0 0 0 0 0 0 0
1 0 0 0 0 0 0 0 0 0 0 0 0
0 0 0 0 0 0 0 0 0 0 0 0 0
0 0 0 0 0 0 0 0 0 1 0 0 0
0 0 0 0 0 0 0 0
"Viruses;Uroviricota;Caudoviricetes;Caudoviricetes__na;Caudoviricetes__na;Caudoviricetes__na;Siphoviridae_sp._ctHeV6" 0 0 0 0 0 0 0 0 0 0 0 0
0 0 0 0 0 0 0 0 0 0 0 0 0
0 0 0 0 0 1 0 0 0 0 0 0 0
0 0 0 0 0 0 0 0 0 0 0 0 0
0 0 0 0 0 0 0 0 0 0 0 0 0
0 0 0 0 0 0 0 0
"Viruses;Uroviricota;Caudoviricetes;Caudoviricetes__na;Caudoviricetes__na;Caudoviricetes__na;Siphoviridae_sp._cthh925" 0 0 0 0 0 0 0 0 0 0 0 0
0 0 0 0 0 0 0 0 0 0 0 0 0
0 0 0 0 0 0 1 0 0 0 0 0 0
0 0 0 0 0 0 0 0 0 0 0 0 0
0 0 0 0 0 0 0 0 0 0 0 0 0
0 0 0 0 0 0 0 0
"Viruses;Uroviricota;Caudoviricetes;Caudoviricetes__na;Caudoviricetes__na;Caudoviricetes__na;Siphoviridae_sp._ctHhH6" 0 0 0 0 0 0 0 0 0 0 0 0
0 0 0 0 0 0 0 0 0 0 0 0 0
0 0 0 0 0 0 0 0 0 0 0 0 0
0 0 0 0 0 0 0 0 0 0 0 1 0

0 0 0 0 0 0 0 0 0 0 0 0 0
0 0 0 0 0 0 0 0
"Viruses;Uroviricota;Caudoviricetes;Caudoviricetes__na;Caudoviricetes__na;Caudoviricetes__na;Siphoviridae_s
p._ctHiz26" 0 0 0 0 0 0 0 0 0 0 0 0
0 0 0 0 0 0 0 0 0 0 0 0 0
0 0 0 0 0 50 16 0 0 14 0 0 0
0 0 0 0 0 0 0 0 0 0 0 0 0
0 0 0 0 0 0 0 0 0 0 0 0 0
0 0 0 0 0 0 0 0
"Viruses;Uroviricota;Caudoviricetes;Caudoviricetes__na;Caudoviricetes__na;Caudoviricetes__na;Siphoviridae_s
p._cthjx9" 0 0 0 0 0 0 0 0 0 0 0 0
0 0 0 0 0 0 0 0 0 0 0 0 0
0 0 0 0 0 56 43 0 0 5 0 0 0
0 0 0 0 0 0 0 0 0 0 0 0 0
0 0 0 4 5 0 0 0 0 0 0 0 0
0 0 0 0 0 0 0 0
"Viruses;Uroviricota;Caudoviricetes;Caudoviricetes__na;Caudoviricetes__na;Caudoviricetes__na;Siphoviridae_s
p._ctHjy10" 0 0 0 0 0 0 0 0 0 0 0 0
0 0 0 0 0 0 0 0 0 0 0 0 0
0 0 0 0 0 0 1 0 1 0 0 0 0
0 0 0 0 1 0 0 0 0 0 0 0 0
0 0 0 0 0 0 0 0 0 0 0 0 0
0 0 0 0 0 0 0 0
"Viruses;Uroviricota;Caudoviricetes;Caudoviricetes__na;Caudoviricetes__na;Caudoviricetes__na;Siphoviridae_s
p._ctHMI2" 0 0 0 0 0 2 0 0 0 0 0 0
0 0 0 0 0 0 0 0 0 0 0 0 0
0 0 0 0 0 0 0 0 0 0 0 0 0
0 0 0 0 0 0 0 0 0 0 0 0 0
0 0 0 0 0 0 0 0 0 0 0 0 0
0 0 0 0 0 0 0 0
"Viruses;Uroviricota;Caudoviricetes;Caudoviricetes__na;Caudoviricetes__na;Caudoviricetes__na;Siphoviridae_s
p._ctHn727" 0 0 0 0 0 0 0 0 0 0 0 0
0 0 0 0 0 0 0 0 0 0 0 0 0
0 0 0 0 0 2 5 0 0 0 0 0 0
0 0 0 0 0 0 0 0 0 0 0 0 0
0 0 0 11 15 0 0 0 0 0 0 0 0
0 0 1 7 0 0 0 0
"Viruses;Uroviricota;Caudoviricetes;Caudoviricetes__na;Caudoviricetes__na;Caudoviricetes__na;Siphoviridae_s
p._ctHNH2" 0 0 0 0 0 0 0 0 0 0 0 0
0 0 0 0 0 0 0 0 0 0 0 0 0
0 0 0 0 0 0 0 0 0 0 0 0 0
0 0 0 9 0 0 0 0 0 0 0 0 0
0 0 0 0 0 0 0 0 0 0 0 0 0
0 0 0 0 0 0 0 0
"Viruses;Uroviricota;Caudoviricetes;Caudoviricetes__na;Caudoviricetes__na;Caudoviricetes__na;Siphoviridae_s
p._cthrG7" 0 0 0 0 0 0 0 0 0 0 0 0
0 0 0 0 0 0 0 0 0 0 0 0 0
0 0 0 0 0 0 0 0 0 0 0 0 0
0 0 0 0 0 0 0 0 0 0 1 1 0

0 0 0 0 0 0 0 0 0 2 0 0 0
0 0 0 0 0 0 0 0
"Viruses;Uroviricota;Caudoviricetes;Caudoviricetes__na;Caudoviricetes__na;Caudoviricetes__na;Siphoviridae_s
p._cthRu26" 0 0 0 0 0 0 0 0 0 0 0 0
0 0 0 0 0 0 0 0 0 0 0 0 0
0 0 0 0 0 0 0 0 0 0 0 1 0
0 1 0 0 0 0 0 0 0 0 0 0 0
0 0 0 0 0 0 0 0 0 0 0 0 0
0 0 0 0 0 0 0 0
"Viruses;Uroviricota;Caudoviricetes;Caudoviricetes__na;Caudoviricetes__na;Caudoviricetes__na;Siphoviridae_s
p._cthu813" 0 0 0 0 0 0 0 0 0 0 0 0
1 0 0 0 0 0 0 0 0 0 0 0 0
0 0 0 0 0 0 0 0 0 0 0 0 0
0 0 0 0 0 0 0 0 0 0 0 0 0
0 0 0 0 1 0 0 0 0 0 0 0 0
0 0 1 1 0 0 0 0
"Viruses;Uroviricota;Caudoviricetes;Caudoviricetes__na;Caudoviricetes__na;Caudoviricetes__na;Siphoviridae_s
p._ctHxr66" 0 0 0 0 0 0 0 0 0 0 0 0
0 0 0 0 0 0 0 0 0 0 0 0 0
0 0 0 0 0 0 0 0 0 0 0 1 0
0 0 0 0 0 0 0 0 0 0 0 0 0
0 0 0 0 0 0 0 0 0 0 0 0 0
0 0 0 0 0 0 0 0
"Viruses;Uroviricota;Caudoviricetes;Caudoviricetes__na;Caudoviricetes__na;Caudoviricetes__na;Siphoviridae_s
p._cti0B23" 0 0 0 0 0 0 0 0 0 0 0 0
0 0 0 0 0 0 0 0 0 0 0 0 0
0 0 1 0 0 0 0 0 0 0 0 0 0
0 0 0 0 2 0 0 0 0 0 0 0 0
0 0 0 0 0 0 0 0 0 0 0 0 0
0 0 2 1 0 0 0 0
"Viruses;Uroviricota;Caudoviricetes;Caudoviricetes__na;Caudoviricetes__na;Caudoviricetes__na;Siphoviridae_s
p._cti6K1" 0 0 0 0 0 0 0 0 0 0 0 0
0 0 0 0 0 0 0 0 0 0 0 0 30
10 113 15 62 3 0 0 0 0 0 0 0 0
0 0 0 0 0 0 0 0 0 0 0 0 0
0 0 0 0 0 0 0 0 0 0 0 0 0
0 0 0 0 0 0 0 0
"Viruses;Uroviricota;Caudoviricetes;Caudoviricetes__na;Caudoviricetes__na;Caudoviricetes__na;Siphoviridae_s
p._ctIbi23" 0 0 0 0 0 0 0 0 0 0 0 0
0 0 0 0 0 0 0 0 0 0 0 0 0
0 0 0 0 0 0 0 0 0 0 0 0 0
0 0 0 0 1 0 0 0 0 0 0 0 0
0 0 0 1 1 0 0 0 0 0 1 1 0
0 0 0 2 0 0 0 0
"Viruses;Uroviricota;Caudoviricetes;Caudoviricetes__na;Caudoviricetes__na;Caudoviricetes__na;Siphoviridae_s
p._ctigT3" 0 0 0 0 0 0 0 0 0 0 0 0
0 2 0 0 0 0 0 0 0 0 0 0 0
0 0 0 0 0 0 0 0 0 0 0 0 0
0 0 0 0 0 0 0 0 0 0 6 6 0

0 0 0 0 0 0 0 0 0 0 0 0 0
0 0 0 0 0 0 0 0
"Viruses;Uroviricota;Caudoviricetes;Caudoviricetes__na;Caudoviricetes__na;Caudoviricetes__na;Siphoviridae_sp._ctiMP24" 0 0 0 0 0 0 0 0 0 0 0 0
0 0 0 0 0 0 0 0 0 0 0 0 0
0 0 0 0 0 0 0 0 0 0 0 3 0
0 0 0 0 0 0 0 0 0 0 0 0 0
0 0 0 4 3 0 0 0 0 0 0 0 0
0 0 1 2 0 0 0 0
"Viruses;Uroviricota;Caudoviricetes;Caudoviricetes__na;Caudoviricetes__na;Caudoviricetes__na;Siphoviridae_sp._ctiMX17" 0 0 0 0 0 0 0 0 0 0 0 0
0 0 0 0 0 0 0 0 0 0 0 0 0
0 0 0 0 0 0 0 0 0 0 0 0 0
0 0 0 0 0 0 0 0 0 0 0 0 0
0 0 0 0 0 0 0 0 0 0 0 0 0
0 0 1 3 0 0 0 0
"Viruses;Uroviricota;Caudoviricetes;Caudoviricetes__na;Caudoviricetes__na;Caudoviricetes__na;Siphoviridae_sp._ctitf6" 0 0 0 0 0 0 0 0 0 0 0 0
0 0 0 0 0 0 0 0 0 0 0 0 10
19 0 0 0 0 0 0 0 0 0 0 1 0
0 0 0 0 0 0 0 0 0 0 0 0 0
0 0 0 0 0 0 0 0 0 0 0 0 0
0 0 1 1 0 0 0 0
"Viruses;Uroviricota;Caudoviricetes;Caudoviricetes__na;Caudoviricetes__na;Caudoviricetes__na;Siphoviridae_sp._ctiuu37" 0 0 0 0 0 0 0 0 0 0 0 0
5 2 0 1 0 0 1 2 0 0 1 0 3
6 0 0 0 0 1 0 0 0 0 0 6 4
0 0 0 0 0 1 0 0 0 0 2 3 0
0 0 0 4 1 0 0 0 0 2 3 0 0
0 0 1 5 0 0 1 0
"Viruses;Uroviricota;Caudoviricetes;Caudoviricetes__na;Caudoviricetes__na;Caudoviricetes__na;Siphoviridae_sp._ctiuX7" 0 0 0 0 0 0 0 0 0 0 0 0
0 0 0 0 0 0 0 0 0 0 0 0 0
0 0 0 0 0 0 0 0 0 0 0 0 0
0 0 0 0 0 0 0 0 0 0 0 0 0
0 0 0 0 1 0 0 0 0 0 0 0 0
0 0 0 0 0 0 0 0
"Viruses;Uroviricota;Caudoviricetes;Caudoviricetes__na;Caudoviricetes__na;Caudoviricetes__na;Siphoviridae_sp._ctIwT7" 0 0 0 0 0 0 0 0 0 0 0 0
0 1 1 2 0 2 0 0 0 0 0 0 0
0 0 0 0 0 0 0 0 0 0 0 3 0
0 0 0 0 0 0 0 0 0 0 0 0 0
0 0 0 0 0 0 0 0 0 0 0 0 0
0 0 0 0 0 0 0 0
"Viruses;Uroviricota;Caudoviricetes;Caudoviricetes__na;Caudoviricetes__na;Caudoviricetes__na;Siphoviridae_sp._ctIyp7" 0 0 0 0 0 0 0 0 0 0 0 0
0 0 0 0 0 0 0 0 0 0 0 0 0
0 0 0 0 0 0 0 0 0 0 0 3 0
0 0 0 0 0 0 0 0 0 0 0 0 0

0 0 0 0 0 0 0 0 0 0 0 0 0
0 0 0 0 0 0 0 0
"Viruses;Uroviricota;Caudoviricetes;Caudoviricetes__na;Caudoviricetes__na;Caudoviricetes__na;Siphoviridae_s
p._ctJ0s2" 0 0 0 0 0 0 0 0 0 0 0 0
0 0 0 0 0 0 0 0 0 0 0 0 0
0 0 0 0 0 0 0 0 0 0 0 0 0
0 0 0 5 0 0 0 0 0 0 0 0 0
0 0 0 0 0 0 0 0 0 0 0 0 0
0 0 0 0 0 0 0 0
"Viruses;Uroviricota;Caudoviricetes;Caudoviricetes__na;Caudoviricetes__na;Caudoviricetes__na;Siphoviridae_s
p._ctJ3t72" 0 0 1 0 0 0 0 0 0 0 0 0
0 0 0 0 0 0 0 0 0 0 0 0 0
0 0 0 0 0 0 0 0 0 0 0 0 0
0 0 0 0 0 0 0 0 0 0 0 0 0
0 0 0 0 0 0 0 0 0 0 0 0 0
0 0 0 0 0 0 0 0
"Viruses;Uroviricota;Caudoviricetes;Caudoviricetes__na;Caudoviricetes__na;Caudoviricetes__na;Siphoviridae_s
p._ctj6w2" 0 0 0 0 0 0 0 0 0 0 0 0
0 0 0 0 0 0 0 0 0 0 0 0 0
0 0 0 0 0 0 0 0 0 0 0 0 0
0 0 0 0 0 0 0 0 0 0 0 0 0
0 0 0 0 2 0 0 0 0 0 0 0 0
0 0 0 0 0 0 0 0
"Viruses;Uroviricota;Caudoviricetes;Caudoviricetes__na;Caudoviricetes__na;Caudoviricetes__na;Siphoviridae_s
p._ctj7f2" 0 0 0 0 0 0 0 0 0 0 3 0
0 0 0 0 0 0 0 0 0 0 0 0 0
0 0 0 0 0 0 0 0 0 0 0 0 0
0 0 0 0 0 0 0 0 0 0 0 0 0
0 0 0 0 0 0 0 0 0 0 0 0 0
0 0 0 0 0 0 0 0
"Viruses;Uroviricota;Caudoviricetes;Caudoviricetes__na;Caudoviricetes__na;Caudoviricetes__na;Siphoviridae_s
p._ctJ7x27" 0 0 0 0 0 0 0 0 0 0 0 0
0 0 0 0 0 0 0 0 0 0 0 0 0
0 0 0 0 0 0 0 0 0 0 0 0 0
0 0 0 1 0 0 0 0 0 0 0 0 0
0 0 0 0 0 0 0 0 0 0 0 0 0
0 0 0 0 0 0 0 0
"Viruses;Uroviricota;Caudoviricetes;Caudoviricetes__na;Caudoviricetes__na;Caudoviricetes__na;Siphoviridae_s
p._ctj912" 0 0 0 0 0 0 0 0 0 0 0 0
0 0 0 0 0 0 0 0 0 0 0 0 0
0 0 0 0 0 0 0 0 0 0 0 0 0
0 0 0 4 0 0 0 0 0 0 0 0 0
0 0 0 0 0 0 0 0 0 0 0 0 0
0 0 0 0 0 0 0 0
"Viruses;Uroviricota;Caudoviricetes;Caudoviricetes__na;Caudoviricetes__na;Caudoviricetes__na;Siphoviridae_s
p._ctJbC4" 0 0 0 0 0 0 0 0 0 0 0 0
0 0 0 0 0 0 0 0 0 0 0 0 1
1 0 0 0 0 0 1 0 0 0 0 0 1
0 0 0 0 0 5 0 0 0 0 4 3 0

0 0 0 1 0 0 0 0 0 0 0 0 0
0 0 0 0 0 0 0 0
"Viruses;Uroviricota;Caudoviricetes;Caudoviricetes__na;Caudoviricetes__na;Caudoviricetes__na;Siphoviridae_sp._ctjbm8" 0 0 0 0 0 0 0 0 0 0 0 0
0 0 0 0 0 0 0 0 0 0 0 0 0
0 0 0 0 0 0 0 0 0 1 0 0 0
0 0 0 0 0 0 0 0 0 0 0 0 0
0 0 0 0 3 0 0 0 0 2 0 0 0
0 0 2 1 0 0 0 0
"Viruses;Uroviricota;Caudoviricetes;Caudoviricetes__na;Caudoviricetes__na;Caudoviricetes__na;Siphoviridae_sp._ctjdk2" 0 1 0 0 0 0 0 0 0 0 0 0
0 0 0 0 0 0 7 4 0 0 0 0 0
0 0 0 0 0 2 6 0 0 0 0 2 1
0 0 0 0 0 0 0 0 0 0 0 0 0
0 0 0 12 14 2 1 0 0 0 0 0 0
0 0 13 7 0 0 0 0
"Viruses;Uroviricota;Caudoviricetes;Caudoviricetes__na;Caudoviricetes__na;Caudoviricetes__na;Siphoviridae_sp._ctjfQ5" 0 0 0 0 0 0 0 0 0 0 0 0
0 0 0 0 0 0 0 0 0 0 0 0 0
0 0 0 0 0 0 0 0 0 0 0 0 0
0 0 0 0 0 0 0 0 1 0 0 0 0
0 0 0 23 40 1 1 0 0 0 0 0 0
0 0 0 1 0 0 0 0
"Viruses;Uroviricota;Caudoviricetes;Caudoviricetes__na;Caudoviricetes__na;Caudoviricetes__na;Siphoviridae_sp._ctJj91" 0 0 0 0 0 0 0 0 0 0 0 0
0 1 0 1 0 0 0 0 0 0 0 0 0
0 0 0 0 0 0 0 0 0 0 0 0 0
0 0 0 0 0 0 0 0 0 0 0 0 0
0 0 0 0 1 0 0 0 0 0 0 0 0
0 0 0 0 0 0 0 0
"Viruses;Uroviricota;Caudoviricetes;Caudoviricetes__na;Caudoviricetes__na;Caudoviricetes__na;Siphoviridae_sp._ctJLl6" 0 0 0 0 0 0 0 0 0 0 0 0
0 0 0 0 0 0 0 0 0 0 0 0 0
0 0 0 0 0 0 0 0 0 0 0 1 1
0 0 0 0 0 0 0 0 0 0 0 0 0
0 0 0 0 0 0 0 0 0 0 0 0 0
0 0 0 0 0 0 0 0
"Viruses;Uroviricota;Caudoviricetes;Caudoviricetes__na;Caudoviricetes__na;Caudoviricetes__na;Siphoviridae_sp._ctJLm32" 0 0 0 0 0 0 0 0 0 0 0 0
0 0 0 0 0 0 0 0 0 0 0 0 0
0 0 0 0 0 0 0 0 0 0 0 0 0
0 0 0 0 0 0 0 0 0 0 5 7 0
0 0 0 0 0 0 0 0 0 1 0 0 0
0 0 0 0 0 0 0 0
"Viruses;Uroviricota;Caudoviricetes;Caudoviricetes__na;Caudoviricetes__na;Caudoviricetes__na;Siphoviridae_sp._ctjRi1" 0 0 0 0 0 0 0 0 0 0 0 0
0 0 0 0 0 0 0 0 0 0 0 0 0
0 0 0 0 0 0 0 0 0 0 0 0 0
0 0 0 0 0 0 0 0 0 0 0 0 0

0 0 0 0 1 0 0 0 0 0 0 0 0
0 0 0 0 0 0 0 0
"Viruses;Uroviricota;Caudoviricetes;Caudoviricetes__na;Caudoviricetes__na;Caudoviricetes__na;Siphoviridae_s
p._ctjsp22" 0 0 0 0 0 0 0 0 0 0 0 0
0 0 0 0 0 0 0 0 0 0 0 0 0
0 0 0 0 0 0 0 0 0 0 0 4 7
0 0 0 0 0 0 0 0 0 0 0 0 0
0 0 0 0 0 0 0 0 0 0 0 0 0
0 0 0 0 0 0 0 0
"Viruses;Uroviricota;Caudoviricetes;Caudoviricetes__na;Caudoviricetes__na;Caudoviricetes__na;Siphoviridae_s
p._ctJT77" 0 0 0 0 0 0 0 0 0 0 0 0
0 0 0 0 0 1 0 0 0 0 0 0 0
0 0 0 0 0 0 0 0 0 0 0 0 0
0 0 0 0 0 0 0 0 0 0 0 0 0
0 0 0 0 0 0 0 0 0 0 0 0 0
0 0 0 0 0 0 0 0
"Viruses;Uroviricota;Caudoviricetes;Caudoviricetes__na;Caudoviricetes__na;Caudoviricetes__na;Siphoviridae_s
p._ctJZ725" 0 0 0 0 0 0 0 0 0 0 0 0
0 0 0 0 0 0 0 0 0 0 0 0 0
0 0 0 0 0 0 0 0 0 0 0 0 0
0 0 0 0 0 0 0 0 0 0 0 0 0
0 0 0 0 1 0 0 0 0 0 0 0 0
0 0 0 0 0 0 0 0
"Viruses;Uroviricota;Caudoviricetes;Caudoviricetes__na;Caudoviricetes__na;Caudoviricetes__na;Siphoviridae_s
p._ctk4d14" 0 0 0 0 0 0 0 0 0 0 0 0
0 0 0 0 0 0 0 0 0 0 0 0 1
3 0 0 0 0 0 0 0 0 0 0 0 0
0 0 0 0 0 0 0 0 0 0 0 0 0
0 0 0 0 0 0 0 0 0 0 0 0 0
0 0 0 0 0 0 0 0
"Viruses;Uroviricota;Caudoviricetes;Caudoviricetes__na;Caudoviricetes__na;Caudoviricetes__na;Siphoviridae_s
p._ctkcl3" 0 0 0 0 0 0 0 0 0 0 0 0
0 0 0 0 0 0 6 7 0 0 0 1 0
0 0 0 0 0 0 0 0 0 0 0 0 0
0 0 0 0 0 0 0 0 0 0 0 0 0
0 0 0 0 0 0 0 0 0 0 0 0 0
0 0 0 0 0 0 0 0
"Viruses;Uroviricota;Caudoviricetes;Caudoviricetes__na;Caudoviricetes__na;Caudoviricetes__na;Siphoviridae_s
p._ctKeG8" 0 0 0 0 0 0 0 0 0 0 0 0
0 0 0 0 0 0 0 0 0 0 0 0 0
0 0 0 0 0 0 1 0 0 5 0 0 0
0 0 0 0 0 0 0 0 0 0 0 0 0
0 0 0 0 0 0 0 0 0 0 0 0 0
0 0 0 0 0 0 0 0
"Viruses;Uroviricota;Caudoviricetes;Caudoviricetes__na;Caudoviricetes__na;Caudoviricetes__na;Siphoviridae_s
p._ctkfT29" 0 0 0 0 0 0 0 0 0 0 0 0
0 0 0 0 0 0 0 0 0 0 0 0 0
0 0 0 0 0 0 0 0 0 0 0 1 3
0 0 0 0 0 0 0 0 0 0 0 0 0

0 0 0 0 0 0 0 0 0 0 0 0 0
0 0 0 0 0 0 0 0
"Viruses;Uroviricota;Caudoviricetes;Caudoviricetes__na;Caudoviricetes__na;Caudoviricetes__na;Siphoviridae_s
p._ctkfY9" 0 0 0 0 0 0 0 0 0 0 0 0
0 0 0 0 0 0 0 0 0 0 0 0 0
0 0 0 0 0 0 0 0 0 0 0 0 0
0 0 0 1 0 0 0 0 0 0 0 0 0
0 0 0 0 0 0 0 0 0 0 0 0 0
0 0 0 0 0 0 0 0
"Viruses;Uroviricota;Caudoviricetes;Caudoviricetes__na;Caudoviricetes__na;Caudoviricetes__na;Siphoviridae_s
p._ctkI07" 0 0 0 0 0 0 0 0 0 0 0 0
0 0 0 0 0 0 0 0 0 0 0 0 0
0 0 0 0 0 0 0 0 0 0 0 0 0
0 0 0 0 0 0 0 0 0 0 0 0 0
0 0 0 0 0 0 0 0 0 0 0 0 0
0 0 1 3 0 0 0 0
"Viruses;Uroviricota;Caudoviricetes;Caudoviricetes__na;Caudoviricetes__na;Caudoviricetes__na;Siphoviridae_s
p._ctkJH11" 0 0 0 0 0 0 0 0 0 0 0 0
1 0 0 0 1 0 0 0 0 0 0 0 0
0 0 0 0 0 0 0 0 0 0 0 0 0
0 0 0 0 0 0 0 0 0 0 0 0 0
0 0 0 0 1 0 0 0 0 0 0 0 0
0 0 0 0 0 0 0 0
"Viruses;Uroviricota;Caudoviricetes;Caudoviricetes__na;Caudoviricetes__na;Caudoviricetes__na;Siphoviridae_s
p._ctkkB9" 0 0 0 0 0 0 0 0 0 0 0 0
2 1 0 0 0 0 0 0 0 0 0 0 0
0 0 0 0 0 0 0 0 0 0 0 0 0
0 0 0 0 0 0 0 0 0 0 0 0 0
0 0 0 0 0 0 0 0 0 0 0 0 0
0 0 0 0 0 0 0 0
"Viruses;Uroviricota;Caudoviricetes;Caudoviricetes__na;Caudoviricetes__na;Caudoviricetes__na;Siphoviridae_s
p._ctkKt3" 0 0 0 0 0 0 0 0 0 0 0 0
0 1 0 0 0 0 0 0 0 0 0 0 0
0 0 0 0 0 0 1 0 0 0 0 0 0
0 0 0 0 0 0 0 0 0 0 0 0 0
0 0 0 1 0 0 0 0 0 0 0 0 0
0 0 0 0 0 0 0 0
"Viruses;Uroviricota;Caudoviricetes;Caudoviricetes__na;Caudoviricetes__na;Caudoviricetes__na;Siphoviridae_s
p._ctksc2" 0 0 0 0 0 0 0 0 0 0 0 0
0 0 0 0 0 0 0 0 0 0 0 0 0
0 0 0 0 0 0 0 0 0 0 0 0 0
0 0 0 0 0 0 0 0 0 0 0 0 0
0 0 0 0 0 1 0 0 0 0 0 0 0
0 0 0 0 0 0 0 0
"Viruses;Uroviricota;Caudoviricetes;Caudoviricetes__na;Caudoviricetes__na;Caudoviricetes__na;Siphoviridae_s
p._ctkV91" 0 0 0 0 0 0 0 0 0 0 42 0
0 0 0 0 0 0 0 0 0 0 0 0 0
0 0 0 0 0 0 0 0 0 0 3 0 0
0 0 0 0 0 0 0 0 8 0 0 0 0

0 0 0 0 0 0 0 0 0 0 0 0 0
0 0 0 0 0 0 0 0
"Viruses;Uroviricota;Caudoviricetes;Caudoviricetes__na;Caudoviricetes__na;Caudoviricetes__na;Siphoviridae_sp._ctKvA22" 0 0 0 0 0 0 0 0 0 0 0 0
0 0 0 0 0 0 0 0 0 0 0 0 0
0 0 0 0 0 1 0 0 0 0 0 0 0
0 0 1 0 2 7 2 0 0 0 0 0 0
0 0 0 0 0 0 0 0 0 0 0 0 0
0 0 0 0 0 0 0 0
"Viruses;Uroviricota;Caudoviricetes;Caudoviricetes__na;Caudoviricetes__na;Caudoviricetes__na;Siphoviridae_sp._ctKXi8" 0 0 0 0 0 0 0 0 0 0 0 0
0 0 0 1 0 0 0 0 0 0 0 0 0
0 0 0 0 0 0 0 0 0 0 0 0 0
0 0 0 0 0 0 0 0 0 0 7 1 0
0 0 0 0 0 0 0 0 0 0 0 0 0
0 0 0 0 0 0 0 0
"Viruses;Uroviricota;Caudoviricetes;Caudoviricetes__na;Caudoviricetes__na;Caudoviricetes__na;Siphoviridae_sp._ctKy93" 0 0 0 0 0 0 0 0 0 0 0 0
0 0 0 0 0 0 0 0 0 0 0 0 0
0 0 0 0 0 0 0 0 0 0 0 0 0
0 0 0 0 0 0 0 0 0 0 0 0 2
0 0 0 0 0 0 0 5 0 0 0 0 0
0 0 0 0 0 0 12 0
"Viruses;Uroviricota;Caudoviricetes;Caudoviricetes__na;Caudoviricetes__na;Caudoviricetes__na;Siphoviridae_sp._ctkyH28" 0 0 0 0 0 0 0 0 0 0 0 0
0 0 0 0 0 0 0 0 0 0 0 0 0
0 0 0 0 0 0 0 0 0 0 0 0 0
0 0 0 0 0 0 0 0 0 0 0 0 0
0 0 0 0 0 0 0 0 0 0 0 0 0
0 0 1 0 0 0 0 0
"Viruses;Uroviricota;Caudoviricetes;Caudoviricetes__na;Caudoviricetes__na;Caudoviricetes__na;Siphoviridae_sp._ctl0E3" 0 0 0 0 0 0 0 0 0 0 0 0
0 0 0 0 0 0 0 0 0 0 0 0 0
0 0 0 0 0 0 0 0 0 0 0 0 0
0 0 0 0 0 0 0 0 0 0 0 0 0
0 0 0 0 0 0 0 0 0 0 0 0 0
0 0 0 0 0 0 1 0
"Viruses;Uroviricota;Caudoviricetes;Caudoviricetes__na;Caudoviricetes__na;Caudoviricetes__na;Siphoviridae_sp._ctL0q1" 0 0 0 0 0 0 0 0 0 0 0 0
0 0 0 0 0 0 0 0 0 0 0 0 0
2 0 0 0 0 0 0 0 0 2 0 1 0
0 0 0 0 0 0 0 0 0 0 0 1 0
0 0 0 0 0 0 0 0 0 0 0 0 0
0 0 2 0 0 0 0 0
"Viruses;Uroviricota;Caudoviricetes;Caudoviricetes__na;Caudoviricetes__na;Caudoviricetes__na;Siphoviridae_sp._ctL4w2" 0 0 0 0 0 0 0 0 0 0 0 0
0 0 0 0 0 0 0 0 0 0 0 0 0
0 0 0 0 0 0 0 0 0 0 0 0 0
0 0 2 0 0 0 2 0 0 0 0 0 0

0 0 0 0 0 0 0 0 0 0 0 0 0
0 0 0 0 0 0 0 0
"Viruses;Uroviricota;Caudoviricetes;Caudoviricetes__na;Caudoviricetes__na;Caudoviricetes__na;Siphoviridae_sp._ctL5G6" 0 0 0 0 0 0 0 0 0 0 0 0
0 0 0 0 0 0 0 0 0 0 0 0 4
3 0 0 0 0 1 4 0 0 0 0 0 2
0 0 0 0 0 0 0 0 0 0 1 4 0
0 0 0 3 11 0 0 0 0 3 1 0 0
0 0 0 1 0 0 0 0
"Viruses;Uroviricota;Caudoviricetes;Caudoviricetes__na;Caudoviricetes__na;Caudoviricetes__na;Siphoviridae_sp._ctl617" 0 0 0 0 0 0 0 0 0 0 0 0
0 0 0 0 0 0 0 0 0 0 0 0 0
0 0 0 0 0 0 0 0 0 0 0 0 0
0 0 0 0 0 0 0 0 0 0 0 0 0
0 0 0 0 0 0 0 0 0 0 0 0 0
0 0 0 1 0 0 0 0
"Viruses;Uroviricota;Caudoviricetes;Caudoviricetes__na;Caudoviricetes__na;Caudoviricetes__na;Siphoviridae_sp._ctL7J9" 0 0 0 0 0 0 0 0 0 0 0 0
0 0 0 0 0 0 0 0 0 0 0 0 0
0 0 0 0 0 0 0 0 0 0 0 2 0
0 0 0 0 0 0 0 0 0 0 0 0 0
0 0 0 0 0 0 0 0 0 0 0 0 0
0 0 0 0 0 0 0 0
"Viruses;Uroviricota;Caudoviricetes;Caudoviricetes__na;Caudoviricetes__na;Caudoviricetes__na;Siphoviridae_sp._ctLAw30" 0 0 0 0 0 0 0 0 0 0 0 0
0 0 0 0 0 0 0 0 0 0 0 0 0
0 0 0 0 0 0 0 0 0 0 0 1 0
0 0 0 0 0 0 0 0 0 0 9 8 7
0 1 0 0 0 0 0 0 0 0 0 0 0
0 0 0 0 0 0 0 0
"Viruses;Uroviricota;Caudoviricetes;Caudoviricetes__na;Caudoviricetes__na;Caudoviricetes__na;Siphoviridae_sp._ctLeh52" 0 0 0 0 0 0 0 0 0 0 0 0
0 0 0 0 0 0 0 0 0 0 0 0 0
0 0 0 0 0 0 0 0 0 0 0 0 0
0 0 0 0 0 0 0 0 0 0 0 0 0
0 0 260 0 0 0 0 0 0 0 0 0 0
0 0 0 1 0 0 0 0
"Viruses;Uroviricota;Caudoviricetes;Caudoviricetes__na;Caudoviricetes__na;Caudoviricetes__na;Siphoviridae_sp._ctLgc23" 0 0 0 0 0 0 0 0 0 0 0 0
0 0 0 0 0 0 0 0 0 0 0 0 0
0 0 0 0 0 0 0 0 0 0 0 0 0
0 0 0 0 0 0 0 0 0 0 0 0 0
0 0 0 0 0 0 0 0 0 0 0 0 0
0 0 1 1 0 0 0 0
"Viruses;Uroviricota;Caudoviricetes;Caudoviricetes__na;Caudoviricetes__na;Caudoviricetes__na;Siphoviridae_sp._ctlHU7" 0 0 0 0 0 0 0 0 0 0 0 0
0 1 0 2 2 1 0 2 0 0 0 0 0
0 0 0 0 0 0 0 0 0 0 0 1 0
0 0 1 0 3 1 1 0 0 0 1 1 0

0 0 0 1 0 0 0 0 0 0 0 0 0
0 0 0 0 0 0 0 0
"Viruses;Uroviricota;Caudoviricetes;Caudoviricetes__na;Caudoviricetes__na;Caudoviricetes__na;Siphoviridae_sp._ctlIg4" 0 0 1 0 0 0 0 0 0 0 0 0
0 0 0 0 0 0 1 0 0 0 0 0 0
0 0 0 0 0 0 0 0 0 0 0 0 0
0 0 0 0 0 0 0 0 0 0 0 0 0
0 0 0 0 0 0 0 0 0 0 0 0 0
0 0 0 0 0 0 0 0
"Viruses;Uroviricota;Caudoviricetes;Caudoviricetes__na;Caudoviricetes__na;Caudoviricetes__na;Siphoviridae_sp._ctLKT1" 0 0 0 0 0 0 0 0 0 0 0 0
0 0 0 0 0 0 0 0 0 0 0 0 0
0 0 0 0 0 0 0 0 0 0 0 0 0
0 0 0 0 0 0 0 0 0 0 0 0 0
0 0 0 0 0 0 0 0 0 0 0 0 0
0 0 0 3 0 0 0 0
"Viruses;Uroviricota;Caudoviricetes;Caudoviricetes__na;Caudoviricetes__na;Caudoviricetes__na;Siphoviridae_sp._ctLmu1" 0 0 0 0 0 0 0 0 0 0 0 0
0 1 0 0 0 0 0 0 0 0 0 0 1
0 0 0 0 0 0 0 0 0 0 0 1 2
0 0 0 0 0 0 0 0 0 0 0 0 0
0 0 0 0 1 0 0 0 0 0 0 0 0
0 0 1 4 0 0 0 0
"Viruses;Uroviricota;Caudoviricetes;Caudoviricetes__na;Caudoviricetes__na;Caudoviricetes__na;Siphoviridae_sp._ctLnP14" 0 0 0 0 0 0 0 0 0 0 0 0
0 0 0 0 0 0 0 0 0 0 0 0 0
0 0 0 0 0 0 0 0 0 0 0 0 0
0 0 0 0 0 0 0 0 0 0 1 0 0
0 0 0 0 0 0 0 0 0 0 0 0 0
0 0 0 0 0 0 0 0
"Viruses;Uroviricota;Caudoviricetes;Caudoviricetes__na;Caudoviricetes__na;Caudoviricetes__na;Siphoviridae_sp._ctLR131" 0 0 0 0 0 0 0 0 0 0 0 0
0 0 0 0 0 0 0 0 0 0 0 0 0
0 0 0 0 0 0 0 0 0 0 0 0 0
0 0 0 0 0 0 0 0 0 0 0 0 0
0 0 0 0 0 0 0 0 0 0 0 0 0
0 0 4 7 0 1 0 0
"Viruses;Uroviricota;Caudoviricetes;Caudoviricetes__na;Caudoviricetes__na;Caudoviricetes__na;Siphoviridae_sp._ctLsx2" 0 0 0 0 0 0 0 0 0 0 0 0
0 0 0 0 0 0 0 0 0 0 0 0 0
0 0 0 0 0 0 0 0 0 0 0 8 2
0 0 0 4 0 0 0 0 0 0 0 0 0
0 1 0 0 0 0 0 0 0 0 0 0 0
0 0 0 0 0 0 0 0
"Viruses;Uroviricota;Caudoviricetes;Caudoviricetes__na;Caudoviricetes__na;Caudoviricetes__na;Siphoviridae_sp._ctLUY1" 0 0 0 0 0 0 0 0 0 0 0 0
0 0 0 0 0 0 0 0 0 0 0 0 0
0 0 0 0 0 0 0 0 0 0 0 2 0
0 0 0 0 0 0 0 0 0 0 0 0 0

0 0 0 0 1 0 0 0 0 2 0 0 0
0 0 0 0 0 0 0 0
"Viruses;Uroviricota;Caudoviricetes;Caudoviricetes__na;Caudoviricetes__na;Caudoviricetes__na;Siphoviridae_s
p._ctM3g2" 0 0 0 0 0 0 0 0 0 0 0 0
0 0 0 0 0 0 0 0 0 0 0 0 0
0 0 0 0 0 0 2 0 0 0 0 0 0
0 0 0 0 0 0 0 0 0 0 0 0 0
0 0 0 3 0 0 0 0 0 0 0 0 0
0 0 0 0 0 0 0 0
"Viruses;Uroviricota;Caudoviricetes;Caudoviricetes__na;Caudoviricetes__na;Caudoviricetes__na;Siphoviridae_s
p._ctM4P7" 0 0 0 0 5 0 0 0 0 0 0 0
0 0 0 0 0 0 0 0 0 0 0 0 0
0 0 0 0 0 0 0 0 0 0 0 0 0
0 0 0 0 0 0 0 0 0 0 0 0 0
0 0 0 0 0 0 0 0 0 0 0 0 0
0 0 0 0 0 0 0 0
"Viruses;Uroviricota;Caudoviricetes;Caudoviricetes__na;Caudoviricetes__na;Caudoviricetes__na;Siphoviridae_s
p._ctM6i4" 0 0 0 0 0 0 0 0 0 0 0 0
0 0 0 0 0 0 0 0 0 0 0 0 0
0 0 0 0 0 0 0 0 0 0 0 0 0
0 0 0 0 0 0 0 0 0 0 0 0 0
0 0 0 1 0 0 0 0 0 0 0 0 0
0 0 0 4 0 0 1 0
"Viruses;Uroviricota;Caudoviricetes;Caudoviricetes__na;Caudoviricetes__na;Caudoviricetes__na;Siphoviridae_s
p._ctm8l1" 0 0 0 0 0 0 0 0 0 0 0 0
0 0 0 0 0 0 0 0 0 0 0 0 0
0 0 0 0 0 0 0 0 0 0 0 0 0
0 0 0 0 2 0 0 0 0 0 0 0 0
0 0 0 1 2 0 0 0 0 0 0 0 0
0 0 0 0 0 0 0 0
"Viruses;Uroviricota;Caudoviricetes;Caudoviricetes__na;Caudoviricetes__na;Caudoviricetes__na;Siphoviridae_s
p._ctMAv2" 0 0 0 0 0 0 0 0 0 0 0 0
0 0 0 0 0 0 0 0 0 0 0 0 0
0 0 0 0 0 0 0 0 0 0 0 0 0
0 0 0 0 0 0 0 0 0 0 0 0 0
0 0 0 0 0 0 0 0 0 0 0 0 0
0 0 1 1 0 0 0 0
"Viruses;Uroviricota;Caudoviricetes;Caudoviricetes__na;Caudoviricetes__na;Caudoviricetes__na;Siphoviridae_s
p._ctMCY8" 0 0 0 0 0 0 0 0 0 0 0 0
2 0 0 0 0 0 0 0 0 0 0 0 2
0 0 0 0 0 0 0 0 0 0 0 0 1
0 0 0 0 0 0 0 0 0 0 29 20 0
0 0 0 0 0 0 0 0 0 11 5 0 0
0 0 0 0 0 0 0 0
"Viruses;Uroviricota;Caudoviricetes;Caudoviricetes__na;Caudoviricetes__na;Caudoviricetes__na;Siphoviridae_s
p._ctMgg26" 0 0 0 0 0 0 0 0 0 0 0 0
0 0 0 0 0 0 0 0 0 0 0 0 1
0 0 0 0 0 0 0 0 0 0 0 0 0
0 0 0 0 0 0 0 0 0 0 0 0 0

0 0 0 0 0 0 0 0 0 0 0 0 0
0 0 0 0 0 0 0 0
"Viruses;Uroviricota;Caudoviricetes;Caudoviricetes__na;Caudoviricetes__na;Caudoviricetes__na;Siphoviridae_sp._ctmHK1" 0 0 0 0 0 0 0 0 0 0 0 0
0 0 0 0 0 0 0 0 0 0 0 0 0
0 0 0 0 0 0 0 0 0 0 0 0 0
0 0 0 2 0 0 0 0 0 0 0 0 0
0 0 0 0 0 0 0 0 0 0 0 0 0
0 0 0 0 0 0 0 0
"Viruses;Uroviricota;Caudoviricetes;Caudoviricetes__na;Caudoviricetes__na;Caudoviricetes__na;Siphoviridae_sp._ctmIh35" 0 0 0 0 0 0 0 0 0 0 0 0
0 0 0 0 0 0 0 0 0 0 0 0 0
0 0 0 0 0 0 0 0 0 0 0 0 0
0 0 0 96 0 0 0 0 0 0 0 0 0
0 0 0 0 0 0 0 0 0 0 0 0 0
0 0 0 0 0 0 1 0
"Viruses;Uroviricota;Caudoviricetes;Caudoviricetes__na;Caudoviricetes__na;Caudoviricetes__na;Siphoviridae_sp._ctMM521" 0 0 0 0 0 0 0 0 0 0 0 0
1 0 1 0 0 0 1 3 0 0 0 0 0
0 0 0 0 0 0 0 0 0 0 0 0 0
0 0 0 0 0 0 0 0 0 0 1 0 0
0 0 0 1 1 0 0 0 0 0 0 0 0
0 0 0 0 0 0 0 0
"Viruses;Uroviricota;Caudoviricetes;Caudoviricetes__na;Caudoviricetes__na;Caudoviricetes__na;Siphoviridae_sp._ctmpG14" 0 0 0 0 0 0 0 0 0 0 0 0
0 0 0 0 0 0 0 0 0 0 0 0 0
0 0 0 0 0 0 0 0 0 0 0 0 0
0 0 0 0 0 0 0 0 0 0 6 0 2
0 0 0 0 0 0 0 0 0 0 0 0 0
0 0 0 0 0 0 0 0
"Viruses;Uroviricota;Caudoviricetes;Caudoviricetes__na;Caudoviricetes__na;Caudoviricetes__na;Siphoviridae_sp._ctMq01" 0 0 0 0 0 0 0 0 0 0 0 0
0 0 0 0 0 0 0 0 0 0 0 0 0
0 0 0 0 0 0 0 0 0 0 0 0 0
0 0 0 1 0 0 0 0 0 0 0 0 0
0 0 0 0 0 0 0 0 0 0 0 0 0
0 0 0 0 0 0 0 0
"Viruses;Uroviricota;Caudoviricetes;Caudoviricetes__na;Caudoviricetes__na;Caudoviricetes__na;Siphoviridae_sp._ctmqi22" 0 0 0 0 0 0 0 0 0 0 0 0
0 0 0 0 0 0 0 0 0 0 0 0 0
0 0 0 0 0 0 1 0 0 0 0 0 1
0 0 0 0 0 0 0 0 0 0 0 0 0
0 0 0 1 2 0 0 0 0 0 0 0 0
0 0 0 0 0 0 0 0
"Viruses;Uroviricota;Caudoviricetes;Caudoviricetes__na;Caudoviricetes__na;Caudoviricetes__na;Siphoviridae_sp._ctMRT7" 0 0 0 0 0 0 0 0 0 0 0 0
0 0 0 0 0 0 0 0 0 0 0 0 0
0 0 0 0 0 0 1 0 0 0 0 0 0
0 0 0 0 0 0 0 0 0 0 0 0 0

0 0 2 0 0 0 0 0 0 0 0 0 0
0 0 0 0 0 0 0 0
"Viruses;Uroviricota;Caudoviricetes;Caudoviricetes__na;Caudoviricetes__na;Caudoviricetes__na;Siphoviridae_s
p._ctMsr1" 0 0 0 0 0 0 0 0 0 0 0 0
0 0 0 0 0 0 0 0 0 0 0 0 0
0 0 0 0 0 0 0 0 0 16 0 0 0
0 0 0 0 0 0 0 0 0 0 0 0 0
0 0 0 0 0 0 0 0 0 0 0 0 0
0 0 0 0 0 0 0 0
"Viruses;Uroviricota;Caudoviricetes;Caudoviricetes__na;Caudoviricetes__na;Caudoviricetes__na;Siphoviridae_s
p._ctmTU3" 0 0 0 0 0 0 0 0 0 0 0 0
0 0 0 0 0 0 0 0 0 0 0 0 16
14 21 3 16 0 0 0 0 0 0 0 0 1
0 0 0 0 0 0 0 0 0 0 0 0 0
0 0 0 0 0 0 0 0 0 0 0 0 0
0 0 0 0 0 0 0 0
"Viruses;Uroviricota;Caudoviricetes;Caudoviricetes__na;Caudoviricetes__na;Caudoviricetes__na;Siphoviridae_s
p._ctMYd37" 0 0 0 0 0 0 0 0 0 0 0 0
0 0 0 0 0 0 0 0 0 0 0 0 0
0 0 0 0 0 0 0 0 0 0 0 0 0
0 0 0 0 0 0 0 0 0 0 0 0 0
0 0 1 0 0 0 0 0 0 0 0 0 0
0 0 0 0 0 0 0 0
"Viruses;Uroviricota;Caudoviricetes;Caudoviricetes__na;Caudoviricetes__na;Caudoviricetes__na;Siphoviridae_s
p._ctMYJ33" 0 0 0 0 0 0 0 0 0 0 0 0
0 0 0 0 0 0 0 0 0 0 0 0 0
0 0 0 0 0 0 1 0 0 0 0 0 0
0 0 0 0 0 0 0 0 0 0 0 0 0
0 0 0 0 0 0 0 0 0 0 0 0 0
0 0 0 0 0 0 0 0
"Viruses;Uroviricota;Caudoviricetes;Caudoviricetes__na;Caudoviricetes__na;Caudoviricetes__na;Siphoviridae_s
p._ctmYS12" 0 0 0 0 0 0 0 0 0 0 0 0
0 0 0 0 0 0 0 0 0 0 0 1 0
0 0 0 0 0 3 2 0 0 6 0 0 0
0 0 0 0 0 0 0 0 0 0 0 0 0
0 0 0 3 3 0 0 0 0 0 0 0 0
0 0 0 0 0 0 0 0
"Viruses;Uroviricota;Caudoviricetes;Caudoviricetes__na;Caudoviricetes__na;Caudoviricetes__na;Siphoviridae_s
p._ctneY2" 0 0 0 0 0 0 0 0 0 0 0 0
6 0 0 0 0 0 1 3 0 0 0 0 0
0 0 0 0 0 0 1 0 0 0 0 0 2
0 0 0 0 0 0 0 0 0 0 0 0 0
0 0 0 5 0 0 0 0 0 0 0 0 0
0 0 0 0 0 0 0 0
"Viruses;Uroviricota;Caudoviricetes;Caudoviricetes__na;Caudoviricetes__na;Caudoviricetes__na;Siphoviridae_s
p._ctngK14" 0 0 0 0 0 0 0 0 0 0 0 0
0 0 0 0 0 0 0 0 0 0 0 0 0
0 0 0 0 0 1 3 0 0 0 0 0 0
0 0 0 0 0 0 0 0 0 0 0 0 0

0 0 0 0 1 0 0 0 0 0 0 0 0
0 0 0 0 0 0 0 0
"Viruses;Uroviricota;Caudoviricetes;Caudoviricetes__na;Caudoviricetes__na;Caudoviricetes__na;Siphoviridae_s
p._ctNHj22" 0 0 0 0 0 0 0 0 0 0 0 0
0 0 0 0 0 0 0 0 0 0 0 0 0
0 0 0 0 0 0 0 0 0 0 0 0 0
0 0 0 0 0 0 0 0 0 0 0 0 0
0 0 0 0 0 0 0 0 0 0 0 0 0
0 0 0 1 0 0 1 0
"Viruses;Uroviricota;Caudoviricetes;Caudoviricetes__na;Caudoviricetes__na;Caudoviricetes__na;Siphoviridae_s
p._ctNiB4" 0 0 0 1 0 0 0 0 0 0 0 0
1 0 0 1 0 1 0 0 0 0 0 0 0
1 0 0 0 0 0 0 0 0 0 0 0 0
0 0 0 0 0 0 0 0 0 0 2 0 0
0 0 0 1 0 0 0 0 0 2 1 0 0
0 0 0 1 0 0 0 0
"Viruses;Uroviricota;Caudoviricetes;Caudoviricetes__na;Caudoviricetes__na;Caudoviricetes__na;Siphoviridae_s
p._ctNLX12" 0 0 0 0 0 0 0 0 0 0 0 0
0 0 0 0 0 0 0 1 0 0 0 0 0
0 0 0 0 0 1 0 0 0 0 0 0 0
0 0 0 0 0 0 0 0 0 0 0 0 0
0 0 0 0 0 0 0 0 0 0 0 0 0
0 0 0 0 0 0 0 0
"Viruses;Uroviricota;Caudoviricetes;Caudoviricetes__na;Caudoviricetes__na;Caudoviricetes__na;Siphoviridae_s
p._ctnN38" 0 0 0 0 0 0 0 0 0 0 0 0
0 0 0 0 0 0 0 0 0 0 0 0 0
0 0 0 0 0 0 0 0 0 1 0 10 15
0 0 0 0 0 0 0 0 0 0 0 0 0
0 0 0 0 0 0 0 0 0 0 0 0 0
0 0 0 0 0 0 0 0
"Viruses;Uroviricota;Caudoviricetes;Caudoviricetes__na;Caudoviricetes__na;Caudoviricetes__na;Siphoviridae_s
p._ctnNB1" 0 0 0 0 0 0 0 0 0 0 0 0
1 0 0 0 1 0 0 0 0 0 0 0 0
0 0 0 0 0 0 0 0 0 0 0 0 1
0 0 0 0 0 0 0 0 0 0 0 0 0
0 0 0 0 0 0 0 0 0 0 0 0 0
0 0 0 0 0 0 0 0
"Viruses;Uroviricota;Caudoviricetes;Caudoviricetes__na;Caudoviricetes__na;Caudoviricetes__na;Siphoviridae_s
p._ctNnX9" 0 0 0 0 0 0 0 0 0 0 0 0
0 0 0 0 0 0 0 0 0 0 0 0 0
0 0 0 0 0 0 0 0 0 0 0 0 0
0 0 0 0 0 0 0 0 0 0 0 0 0
0 0 0 0 2 0 0 0 0 0 0 0 0
0 0 0 0 0 0 0 0
"Viruses;Uroviricota;Caudoviricetes;Caudoviricetes__na;Caudoviricetes__na;Caudoviricetes__na;Siphoviridae_s
p._ctnoo6" 0 0 0 0 0 0 0 0 0 0 0 0
0 0 0 0 0 0 0 0 0 0 0 0 0
0 0 0 0 0 0 0 0 0 0 0 0 0
0 0 0 0 0 0 0 0 0 0 0 0 0

0 0 0 0 0 0 0 0 0 0 0 0 0
0 0 1 0 0 0 0 0
"Viruses;Uroviricota;Caudoviricetes;Caudoviricetes__na;Caudoviricetes__na;Caudoviricetes__na;Siphoviridae_sp._ctnPP24" 0 0 0 0 0 0 0 0 0 0 0 0
0 0 0 0 0 0 0 0 0 0 0 0 0
0 0 0 0 0 0 0 0 0 0 0 0 0
0 0 0 0 0 0 0 0 0 0 0 0 0
0 0 0 0 2 0 0 0 0 0 0 0 0
0 0 0 0 0 0 0 0
"Viruses;Uroviricota;Caudoviricetes;Caudoviricetes__na;Caudoviricetes__na;Caudoviricetes__na;Siphoviridae_sp._ctnpt50" 0 0 0 0 0 0 0 0 0 0 0 0
0 0 1 0 0 0 0 0 0 0 0 0 0
0 0 0 0 0 0 0 0 0 0 0 0 0
0 0 0 0 0 0 0 0 0 0 0 0 0
0 0 0 0 0 0 0 0 0 0 0 0 0
0 0 0 0 0 0 0 0
"Viruses;Uroviricota;Caudoviricetes;Caudoviricetes__na;Caudoviricetes__na;Caudoviricetes__na;Siphoviridae_sp._ctNwR4" 0 0 0 0 0 0 0 0 0 0 0 0
1 0 0 0 0 0 0 0 0 0 0 0 0
1 0 0 0 0 0 0 0 0 0 0 1 1
0 0 0 0 1 0 0 0 0 0 0 0 0
0 0 0 1 1 0 0 0 0 1 0 0 0
0 0 1 0 0 0 0 0
"Viruses;Uroviricota;Caudoviricetes;Caudoviricetes__na;Caudoviricetes__na;Caudoviricetes__na;Siphoviridae_sp._ctNyC15" 0 0 0 0 0 0 0 0 0 0 0 0
0 0 0 0 0 0 0 0 0 0 0 0 0
0 0 0 0 0 0 0 0 0 0 0 0 0
0 0 0 0 0 0 0 0 0 0 0 0 0
0 0 0 0 0 0 0 0 0 0 0 0 0
0 0 0 0 0 1 0 0
"Viruses;Uroviricota;Caudoviricetes;Caudoviricetes__na;Caudoviricetes__na;Caudoviricetes__na;Siphoviridae_sp._ctNZc11" 0 0 0 0 0 0 0 0 0 0 0 0
1 1 0 0 0 0 0 0 0 0 0 0 0
0 0 0 0 0 0 0 0 0 0 0 0 0
0 0 0 0 0 0 0 0 0 0 0 0 0
0 0 0 0 0 0 0 0 0 0 0 0 0
0 0 0 0 0 0 0 0
"Viruses;Uroviricota;Caudoviricetes;Caudoviricetes__na;Caudoviricetes__na;Caudoviricetes__na;Siphoviridae_sp._cto3L1" 0 0 0 0 0 0 0 0 0 0 0 0
0 0 0 0 0 0 2 2 0 0 0 0 1
2 0 1 0 0 0 0 0 0 0 0 0 0
0 0 1 0 43 134 1 1 0 0 2 3 0
0 0 0 0 0 0 0 0 0 1 3 0 0
0 0 0 1 0 0 0 0
"Viruses;Uroviricota;Caudoviricetes;Caudoviricetes__na;Caudoviricetes__na;Caudoviricetes__na;Siphoviridae_sp._cto6l14" 0 0 0 0 0 0 0 0 0 0 0 0
1 1 0 1 0 0 0 0 0 0 0 0 0
0 0 0 0 0 0 0 0 0 0 0 0 0
0 0 0 0 0 0 0 0 0 0 0 0 0

0 0 0 0 0 0 0 0 0 0 0 0 0
0 0 0 1 0 0 0 0
"Viruses;Uroviricota;Caudoviricetes;Caudoviricetes__na;Caudoviricetes__na;Caudoviricetes__na;Siphoviridae_s
p._ctOba29" 0 0 0 0 0 0 0 0 0 0 0 0
0 1 0 0 0 0 5 3 0 0 0 0 0
0 0 0 0 0 0 0 0 0 0 0 0 0
0 0 0 0 0 0 0 0 0 0 0 4 0
0 0 0 4 3 0 0 0 0 3 1 0 0
0 0 6 2 0 0 0 0
"Viruses;Uroviricota;Caudoviricetes;Caudoviricetes__na;Caudoviricetes__na;Caudoviricetes__na;Siphoviridae_s
p._ctoD011" 0 0 0 0 0 0 0 0 0 0 0 0
0 0 0 0 0 0 0 0 0 0 0 0 0
0 0 0 0 0 0 0 0 0 0 0 0 2
0 0 0 0 0 1 0 0 0 0 0 0 0
0 0 0 2 0 0 0 0 0 0 0 0 0
0 0 0 0 0 0 0 0
"Viruses;Uroviricota;Caudoviricetes;Caudoviricetes__na;Caudoviricetes__na;Caudoviricetes__na;Siphoviridae_s
p._ctoD91" 0 1 0 0 0 0 0 0 0 0 0 0
0 0 0 0 0 0 0 0 0 0 0 0 0
0 0 0 0 0 0 0 0 0 0 0 75 190
0 1 0 1 0 0 0 0 0 0 0 0 0
0 2 52 0 0 0 0 0 0 0 0 0 0
0 0 14 17 1 3 0 0
"Viruses;Uroviricota;Caudoviricetes;Caudoviricetes__na;Caudoviricetes__na;Caudoviricetes__na;Siphoviridae_s
p._ctoiA13" 0 0 0 0 0 0 0 0 0 0 0 0
0 0 0 0 0 1 0 0 0 0 0 0 0
1 0 0 0 0 0 0 0 0 0 0 0 0
0 0 0 0 0 0 0 0 0 0 0 0 0
0 0 0 0 0 0 0 0 0 0 0 0 0
0 0 0 0 0 0 0 0
"Viruses;Uroviricota;Caudoviricetes;Caudoviricetes__na;Caudoviricetes__na;Caudoviricetes__na;Siphoviridae_s
p._ctoiW10" 0 0 0 0 0 0 0 0 0 0 0 0
0 0 0 0 0 0 0 0 0 0 0 0 0
0 0 0 0 0 0 0 0 0 0 0 4 5
0 0 0 2 0 0 0 0 0 0 0 0 0
0 0 0 4 6 0 0 0 0 0 0 0 0
0 0 0 0 0 0 0 0
"Viruses;Uroviricota;Caudoviricetes;Caudoviricetes__na;Caudoviricetes__na;Caudoviricetes__na;Siphoviridae_s
p._ctOkv13" 0 0 0 0 0 0 0 0 0 0 0 0
0 0 0 0 0 0 0 0 0 0 0 0 0
0 0 0 0 0 0 0 0 0 0 0 0 0
0 0 1 0 0 0 5 0 0 0 0 0 0
0 0 0 0 0 0 0 0 0 0 0 0 0
0 0 0 0 0 0 0 0
"Viruses;Uroviricota;Caudoviricetes;Caudoviricetes__na;Caudoviricetes__na;Caudoviricetes__na;Siphoviridae_s
p._ctomJ2" 0 0 0 0 0 0 0 0 0 0 0 0
0 0 0 0 0 0 0 0 0 0 0 0 0
0 0 0 0 0 0 0 0 0 1 0 0 0
0 0 0 0 0 0 0 0 0 0 0 0 0

0 0 0 0 0 0 0 0 0 0 0 0 0
0 0 0 0 0 3 0 0
"Viruses;Uroviricota;Caudoviricetes;Caudoviricetes__na;Caudoviricetes__na;Caudoviricetes__na;Siphoviridae_sp._ctoMP27" 0 0 0 0 0 0 0 0 0 0 0 0
0 0 0 0 0 0 0 0 0 0 0 0 0
0 0 0 0 0 0 0 0 0 0 0 1 3
0 1 0 0 0 0 0 0 0 0 0 0 0
0 0 0 0 0 0 0 0 0 0 0 0 0
0 0 0 0 0 0 0 0
"Viruses;Uroviricota;Caudoviricetes;Caudoviricetes__na;Caudoviricetes__na;Caudoviricetes__na;Siphoviridae_sp._ctoNj20" 0 0 0 0 0 0 0 0 0 0 0 0
0 0 0 0 0 0 0 0 0 0 0 0 0
0 0 0 0 0 0 0 0 0 0 0 6 9
0 0 0 0 0 0 0 0 0 0 0 0 0
0 4 0 0 0 0 0 0 0 0 0 0 0
0 0 0 0 0 0 0 0
"Viruses;Uroviricota;Caudoviricetes;Caudoviricetes__na;Caudoviricetes__na;Caudoviricetes__na;Siphoviridae_sp._ctoof1" 0 0 0 0 0 0 0 0 0 0 0 0
0 0 0 0 0 0 0 0 0 0 0 0 0
0 0 0 0 0 0 0 0 0 0 0 0 1
0 0 0 11 0 0 0 0 0 0 0 0 0
0 0 0 0 1 0 0 0 0 0 0 0 0
0 0 0 3 0 0 0 0
"Viruses;Uroviricota;Caudoviricetes;Caudoviricetes__na;Caudoviricetes__na;Caudoviricetes__na;Siphoviridae_sp._ctoOf8" 0 0 0 0 0 0 0 0 0 0 0 0
0 0 0 0 0 0 0 0 0 0 0 0 1
0 0 0 0 0 0 0 0 0 0 0 0 0
0 0 0 0 0 0 0 0 0 0 0 0 0
0 0 0 0 0 0 0 0 0 0 0 0 0
0 0 0 0 0 0 0 0
"Viruses;Uroviricota;Caudoviricetes;Caudoviricetes__na;Caudoviricetes__na;Caudoviricetes__na;Siphoviridae_sp._ctoRD1" 0 0 0 0 0 0 0 0 0 0 0 0
0 0 0 0 0 0 0 0 0 0 0 0 0
0 0 0 0 0 2 5 0 0 0 0 0 0
0 0 0 0 0 0 0 0 0 0 0 0 0
0 0 0 0 0 0 0 0 0 0 0 0 0
0 0 0 0 0 0 0 0
"Viruses;Uroviricota;Caudoviricetes;Caudoviricetes__na;Caudoviricetes__na;Caudoviricetes__na;Siphoviridae_sp._ctOVO10" 0 0 0 0 0 0 0 0 0 0 0 0
0 0 0 0 0 0 0 0 0 0 0 0 0
0 0 0 0 0 0 0 0 0 0 0 0 0
0 0 0 0 0 0 0 0 0 0 0 0 0
0 0 0 0 0 0 0 0 0 0 0 0 0
0 0 0 0 0 1 0 0
"Viruses;Uroviricota;Caudoviricetes;Caudoviricetes__na;Caudoviricetes__na;Caudoviricetes__na;Siphoviridae_sp._ctoWO12" 0 0 0 0 0 0 0 0 0 0 0 0
0 0 0 0 0 0 0 0 0 0 0 0 0
1 0 0 0 0 0 2 0 0 0 0 5 3
0 0 0 0 0 0 0 0 0 0 0 0 0

0 0 0 3 2 0 0 0 0 0 0 0 0
0 0 0 0 0 0 0 0
"Viruses;Uroviricota;Caudoviricetes;Caudoviricetes__na;Caudoviricetes__na;Caudoviricetes__na;Siphoviridae_s
p._ctoyo6" 0 0 0 0 8 0 0 0 0 0 0 0
2 0 0 0 0 0 2 0 0 0 1 0 0
0 0 0 0 0 3 5 0 0 0 0 1 0
0 0 0 0 0 0 0 0 0 0 0 0 0
0 0 0 2 8 0 0 0 0 0 0 0 0
0 0 0 0 0 0 0 0
"Viruses;Uroviricota;Caudoviricetes;Caudoviricetes__na;Caudoviricetes__na;Caudoviricetes__na;Siphoviridae_s
p._ctP0x5" 0 0 0 0 0 0 0 0 0 0 0 0
0 0 0 0 0 0 0 5 0 0 0 0 0
1 0 0 0 0 0 1 0 0 0 0 2 8
0 0 0 0 1 0 2 0 0 0 0 0 0
0 0 0 0 0 0 0 0 0 0 0 0 1
0 0 9 16 0 0 0 0
"Viruses;Uroviricota;Caudoviricetes;Caudoviricetes__na;Caudoviricetes__na;Caudoviricetes__na;Siphoviridae_s
p._ctP6113" 0 0 0 0 0 0 0 0 0 0 0 0
1 8 2 1 0 0 0 0 0 0 0 0 2
0 0 0 0 0 0 0 0 0 0 0 0 0
0 0 0 0 0 3 0 0 0 0 0 0 0
0 0 0 0 2 0 0 0 0 0 0 0 0
0 0 1 1 0 0 0 0
"Viruses;Uroviricota;Caudoviricetes;Caudoviricetes__na;Caudoviricetes__na;Caudoviricetes__na;Siphoviridae_s
p._ctP6p7" 0 0 0 0 0 0 0 0 0 0 0 0
1 3 0 0 0 5 2 2 1 0 0 1 0
0 0 0 0 0 0 0 0 0 0 0 0 0
0 0 0 0 0 0 0 0 0 0 0 0 0
0 0 0 0 0 0 0 0 0 0 0 0 0
0 0 3 2 0 0 0 0
"Viruses;Uroviricota;Caudoviricetes;Caudoviricetes__na;Caudoviricetes__na;Caudoviricetes__na;Siphoviridae_s
p._ctPAi1" 0 0 0 0 0 0 0 0 0 0 0 0
9 8 0 0 1 0 3 2 0 0 0 0 17
9 1 2 0 0 4 1 0 0 0 0 0 3
0 0 0 0 4 3 0 0 0 0 7 8 0
0 0 0 2 7 0 0 0 0 4 2 1 0
0 0 3 2 0 0 0 0
"Viruses;Uroviricota;Caudoviricetes;Caudoviricetes__na;Caudoviricetes__na;Caudoviricetes__na;Siphoviridae_s
p._ctPB44" 0 0 0 0 0 0 0 0 0 0 0 0
0 0 0 0 0 0 0 0 0 0 0 0 5
8 0 0 0 0 0 0 0 0 0 0 0 0
0 0 0 0 0 0 0 0 0 0 17 11 1
0 0 0 1 2 0 0 0 0 3 1 0 0
0 0 0 0 0 0 0 0
"Viruses;Uroviricota;Caudoviricetes;Caudoviricetes__na;Caudoviricetes__na;Caudoviricetes__na;Siphoviridae_s
p._ctpbb7" 0 0 0 0 0 0 0 0 0 0 0 0
0 0 0 0 0 0 0 0 0 0 0 0 0
0 0 0 0 0 0 0 0 0 0 0 0 0
0 0 0 0 0 0 0 0 0 0 0 0 0

0 0 0 0 0 0 0 0 0 0 0 0 0
0 0 1 3 0 1 0 0
"Viruses;Uroviricota;Caudoviricetes;Caudoviricetes__na;Caudoviricetes__na;Caudoviricetes__na;Siphoviridae_s
p._ctpbe1" 0 0 0 0 0 0 0 0 0 0 0 0
3 1 0 1 0 0 1 4 0 0 0 0 0
0 0 0 0 0 0 0 0 0 0 0 11 4
0 0 0 0 0 0 0 0 0 0 2 1 0
0 0 0 1 0 0 0 0 0 0 0 0 0
0 0 0 0 0 0 0 0
"Viruses;Uroviricota;Caudoviricetes;Caudoviricetes__na;Caudoviricetes__na;Caudoviricetes__na;Siphoviridae_s
p._ctpCx1" 0 0 0 0 0 0 0 0 0 0 0 0
0 0 0 0 0 2 6 6 0 0 0 0 0
0 0 0 0 0 0 0 0 0 0 0 0 0
0 0 0 0 0 0 0 0 0 0 0 0 0
0 0 0 0 0 0 0 0 0 0 0 0 0
0 0 9 15 0 0 0 0
"Viruses;Uroviricota;Caudoviricetes;Caudoviricetes__na;Caudoviricetes__na;Caudoviricetes__na;Siphoviridae_s
p._ctPEx3" 0 0 0 0 0 0 0 0 0 0 0 0
3 1 4 1 0 0 7 11 1 0 0 0 0
0 0 0 0 0 1 0 0 0 0 0 0 0
0 0 0 0 0 0 0 0 0 0 0 0 0
0 0 0 0 0 0 0 0 0 0 0 0 0
0 0 1 2 0 0 0 0
"Viruses;Uroviricota;Caudoviricetes;Caudoviricetes__na;Caudoviricetes__na;Caudoviricetes__na;Siphoviridae_s
p._ctpnN3" 0 0 0 0 0 0 0 0 0 0 0 0
6 11 1 2 0 0 0 0 0 0 0 0 0
0 0 0 0 0 0 0 0 0 0 0 0 0
0 0 0 0 0 0 0 0 0 0 0 0 0
0 0 0 0 0 0 0 0 0 0 0 0 0
0 0 0 0 0 0 0 0
"Viruses;Uroviricota;Caudoviricetes;Caudoviricetes__na;Caudoviricetes__na;Caudoviricetes__na;Siphoviridae_s
p._ctPrm3" 0 0 0 0 0 0 0 0 0 0 0 0
0 0 0 0 0 0 0 0 0 0 0 0 0
0 0 0 0 0 0 0 0 0 0 0 0 0
0 0 0 0 0 0 0 0 0 0 0 0 0
0 0 0 0 0 0 0 0 0 0 0 0 0
0 0 4 3 0 0 0 0
"Viruses;Uroviricota;Caudoviricetes;Caudoviricetes__na;Caudoviricetes__na;Caudoviricetes__na;Siphoviridae_s
p._ctPui28" 0 0 0 0 0 0 0 0 0 0 0 0
0 0 0 0 0 0 0 0 0 0 0 0 0
0 0 0 0 0 1 0 0 0 0 0 0 0
0 0 0 0 0 0 0 0 0 0 0 0 0
0 0 0 0 0 0 0 0 0 0 0 0 0
0 0 0 0 0 0 0 0
"Viruses;Uroviricota;Caudoviricetes;Caudoviricetes__na;Caudoviricetes__na;Caudoviricetes__na;Siphoviridae_s
p._ctPyh10" 0 0 0 0 0 0 0 0 0 0 0 0
0 0 0 0 0 0 0 0 0 0 0 0 0
0 0 0 0 0 5 3 0 0 0 0 61 8
0 0 0 0 0 0 0 0 0 0 0 0 0

0 0 0 2 2 0 0 0 0 0 0 0 0
0 0 34 38 0 9 0 0
"Viruses;Uroviricota;Caudoviricetes;Caudoviricetes__na;Caudoviricetes__na;Caudoviricetes__na;Siphoviridae_s
p._ctqA315" 0 0 0 0 0 0 0 0 0 0 0 0
0 0 0 0 0 0 0 0 0 0 0 0 0
0 0 0 0 0 0 0 0 0 0 0 0 0
0 0 0 0 0 0 0 0 0 0 0 0 0
0 0 0 0 0 0 0 0 0 1 1 0 0
0 0 1 1 0 0 0 0
"Viruses;Uroviricota;Caudoviricetes;Caudoviricetes__na;Caudoviricetes__na;Caudoviricetes__na;Siphoviridae_s
p._ctqBc4" 0 0 0 0 1 0 0 0 0 0 0 0
0 0 0 0 0 0 0 0 0 0 0 0 0
0 0 0 0 0 0 0 0 0 0 0 0 0
0 0 0 0 0 0 0 0 0 0 0 0 0
0 0 0 0 0 0 0 0 0 0 0 0 0
0 0 0 0 0 0 0 0
"Viruses;Uroviricota;Caudoviricetes;Caudoviricetes__na;Caudoviricetes__na;Caudoviricetes__na;Siphoviridae_s
p._ctqBH20" 0 0 0 0 0 0 0 0 0 0 0 0
0 0 0 0 7 10 0 0 0 0 0 0 0
0 0 0 0 0 0 0 0 0 0 0 0 0
0 0 0 0 0 1 1 0 0 0 0 0 0
0 0 0 0 0 0 0 0 0 0 0 0 0
0 0 0 0 0 0 0 0
"Viruses;Uroviricota;Caudoviricetes;Caudoviricetes__na;Caudoviricetes__na;Caudoviricetes__na;Siphoviridae_s
p._ctqcj14" 0 0 0 0 0 0 0 0 0 0 0 0
0 0 0 0 0 0 0 0 0 0 0 0 0
0 0 0 0 0 0 0 0 0 0 0 9 4
1 0 0 0 0 0 0 0 0 0 0 0 0
0 1 0 0 0 0 0 0 0 0 0 0 0
0 0 0 0 0 0 0 0
"Viruses;Uroviricota;Caudoviricetes;Caudoviricetes__na;Caudoviricetes__na;Caudoviricetes__na;Siphoviridae_s
p._ctQHO9" 0 0 0 0 0 0 0 0 0 0 0 0
0 0 0 0 0 0 0 0 1 0 1 0 0
0 0 0 0 0 0 0 0 0 0 0 0 0
0 0 0 0 0 0 0 0 0 0 0 0 0
0 0 0 0 0 0 0 0 0 0 0 0 0
0 0 0 0 0 0 0 0
"Viruses;Uroviricota;Caudoviricetes;Caudoviricetes__na;Caudoviricetes__na;Caudoviricetes__na;Siphoviridae_s
p._ctqK313" 0 0 0 0 0 0 0 0 0 0 0 0
0 0 0 0 0 0 0 0 0 0 0 0 1
0 0 0 0 0 0 0 0 0 0 0 0 0
0 1 0 0 0 0 0 0 0 0 0 0 0
0 0 0 0 0 0 0 0 0 0 0 0 0
0 0 0 0 0 0 0 0
"Viruses;Uroviricota;Caudoviricetes;Caudoviricetes__na;Caudoviricetes__na;Caudoviricetes__na;Siphoviridae_s
p._ctQLz13" 0 0 0 0 0 0 0 0 0 0 0 0
0 0 0 0 0 0 0 0 0 0 0 0 0
0 0 0 0 0 0 0 0 0 0 0 0 0
0 0 0 0 0 0 0 0 0 0 0 0 0

0 0 0 0 0 0 0 0 0 0 1 0 0
0 0 0 1 0 0 0 0
"Viruses;Uroviricota;Caudoviricetes;Caudoviricetes__na;Caudoviricetes__na;Caudoviricetes__na;Siphoviridae_s
p._ctqOv4" 0 0 0 0 0 0 0 0 0 0 0 0
0 3 0 0 0 0 0 0 0 0 0 0 0
0 0 0 0 0 0 0 0 0 0 0 0 0
0 0 0 0 0 1 0 0 0 0 0 0 0
0 0 0 0 0 0 0 0 0 0 0 0 0
0 0 0 0 0 0 0 0
"Viruses;Uroviricota;Caudoviricetes;Caudoviricetes__na;Caudoviricetes__na;Caudoviricetes__na;Siphoviridae_s
p._ctqPo10" 0 0 0 0 0 0 0 0 0 0 0 0
0 1 0 0 0 0 0 0 0 0 0 0 0
0 0 0 0 0 0 0 0 0 0 0 0 0
1 1 0 0 0 0 0 0 0 0 0 0 0
0 0 0 0 0 0 0 0 0 0 0 0 0
0 0 0 0 0 0 0 0
"Viruses;Uroviricota;Caudoviricetes;Caudoviricetes__na;Caudoviricetes__na;Caudoviricetes__na;Siphoviridae_s
p._ctqpo8" 0 0 0 0 0 0 0 0 0 0 0 0
12 6 5 0 0 20 0 0 0 1 0 0 0
0 0 0 0 0 0 0 0 0 0 0 0 0
0 0 0 0 2 0 0 0 0 0 0 0 0
0 0 0 9 9 0 0 0 0 0 0 0 0
0 0 0 2 1 0 0 0
"Viruses;Uroviricota;Caudoviricetes;Caudoviricetes__na;Caudoviricetes__na;Caudoviricetes__na;Siphoviridae_s
p._ctQqU1" 0 0 0 0 0 0 0 0 0 0 0 0
1 0 1 0 0 0 0 0 0 0 0 0 5
10 1 0 0 0 0 0 0 0 0 0 1 1
0 0 0 0 0 1 0 0 0 0 3 3 0
0 0 0 0 6 0 0 0 0 2 1 0 0
0 0 6 6 3 0 0 0
"Viruses;Uroviricota;Caudoviricetes;Caudoviricetes__na;Caudoviricetes__na;Caudoviricetes__na;Siphoviridae_s
p._ctqtA1" 0 0 0 0 0 0 0 0 0 0 0 0
0 0 0 0 0 0 1 0 1 0 0 0 0
1 1 0 0 0 0 0 0 0 0 0 0 0
0 0 0 0 0 0 0 0 0 0 0 0 0
0 1 0 1 0 0 0 0 0 0 0 0 0
0 0 0 0 0 0 0 0
"Viruses;Uroviricota;Caudoviricetes;Caudoviricetes__na;Caudoviricetes__na;Caudoviricetes__na;Siphoviridae_s
p._ctquf9" 0 0 0 0 0 0 0 0 0 0 0 0
1 0 0 0 0 0 0 0 0 0 0 0 0
0 0 0 0 0 0 0 0 0 0 0 0 0
0 0 0 0 0 0 0 0 0 0 0 0 0
0 0 0 0 0 0 0 0 0 0 0 0 0
0 0 0 0 0 0 0 0
"Viruses;Uroviricota;Caudoviricetes;Caudoviricetes__na;Caudoviricetes__na;Caudoviricetes__na;Siphoviridae_s
p._ctqwO1" 0 0 0 0 0 0 0 0 0 0 0 0
0 0 0 0 0 0 0 0 0 0 0 0 0
0 0 0 0 0 0 0 0 0 0 0 0 0
0 0 0 0 0 0 0 0 0 0 0 0 0

0 0 0 0 1 0 0 0 0 0 0 0 0
0 0 0 0 0 0 0 0
"Viruses;Uroviricota;Caudoviricetes;Caudoviricetes__na;Caudoviricetes__na;Caudoviricetes__na;Siphoviridae_sp._ctr2f5" 0 0 0 0 0 0 0 0 0 0 0 0
0 0 0 0 0 0 0 0 0 0 0 0 0
0 0 0 0 0 0 0 0 0 0 0 0 0
0 0 0 0 0 0 0 0 0 0 0 0 0
0 0 0 0 0 0 0 0 0 0 0 0 0
0 0 1 3 0 0 0 0
"Viruses;Uroviricota;Caudoviricetes;Caudoviricetes__na;Caudoviricetes__na;Caudoviricetes__na;Siphoviridae_sp._ctr4Z12" 0 0 0 0 0 0 0 0 0 0 0 0
0 0 0 0 0 0 0 0 0 0 0 0 0
0 0 0 0 0 3 4 0 0 1 0 0 0
0 0 0 1 0 0 0 0 0 0 0 0 0
0 0 0 0 0 0 0 0 0 0 0 0 0
0 0 0 0 0 0 0 0
"Viruses;Uroviricota;Caudoviricetes;Caudoviricetes__na;Caudoviricetes__na;Caudoviricetes__na;Siphoviridae_sp._ctr8v12" 0 0 0 0 0 0 0 0 0 0 0 0
0 0 0 0 0 0 0 0 0 0 0 0 0
0 0 0 0 0 0 0 0 0 0 0 4 2
0 0 0 8 0 0 0 0 0 0 0 0 0
0 0 1 0 0 0 0 0 0 0 0 0 0
0 0 1 0 0 0 0 0
"Viruses;Uroviricota;Caudoviricetes;Caudoviricetes__na;Caudoviricetes__na;Caudoviricetes__na;Siphoviridae_sp._ctRCE13" 0 0 0 0 0 0 0 0 0 0 0 0
0 0 0 0 0 0 0 0 0 0 0 0 0
0 0 0 0 0 0 0 0 0 0 0 0 0
0 0 0 0 0 0 0 0 0 0 0 0 0
0 0 0 0 1 0 0 0 0 0 0 0 0
0 0 0 0 0 0 0 0
"Viruses;Uroviricota;Caudoviricetes;Caudoviricetes__na;Caudoviricetes__na;Caudoviricetes__na;Siphoviridae_sp._ctRcp9" 0 0 0 0 0 0 0 0 0 0 0 0
0 0 0 0 0 0 0 0 0 0 0 0 0
3 0 0 0 0 3 1 0 0 0 0 0 0
0 0 0 0 0 0 0 0 0 0 0 0 0
0 0 0 0 1 0 0 0 0 0 0 0 0
0 0 0 0 0 0 0 0
"Viruses;Uroviricota;Caudoviricetes;Caudoviricetes__na;Caudoviricetes__na;Caudoviricetes__na;Siphoviridae_sp._ctrCv3" 0 0 0 0 0 0 0 0 0 0 0 0
0 0 0 0 0 0 0 0 0 0 0 0 0
0 0 0 0 0 8 9 0 0 2 1 0 0
0 0 0 0 0 0 0 0 0 0 0 0 0
0 0 0 1 1 0 0 0 0 0 0 0 0
0 0 0 0 0 0 0 0
"Viruses;Uroviricota;Caudoviricetes;Caudoviricetes__na;Caudoviricetes__na;Caudoviricetes__na;Siphoviridae_sp._ctREU2" 0 0 0 0 0 0 0 0 0 0 0 0
0 0 0 0 0 0 0 3 0 0 0 0 0
0 0 0 0 0 0 2 0 0 0 0 0 0
0 0 0 0 0 0 0 0 0 0 0 0 0

0 0 0 0 1 0 0 0 0 3 0 0 0
0 0 0 0 0 0 0 0
"Viruses;Uroviricota;Caudoviricetes;Caudoviricetes__na;Caudoviricetes__na;Caudoviricetes__na;Siphoviridae_sp._ctrfD19" 0 1 0 0 0 0 0 0 0 0 0 0
0 1 0 0 0 0 1 1 0 0 0 0 2
1 0 0 0 0 0 0 0 0 0 0 3 1
0 0 0 0 1 0 0 0 0 0 1 0 0
0 0 0 1 1 0 0 0 0 1 1 0 0
0 0 0 6 0 0 0 0
"Viruses;Uroviricota;Caudoviricetes;Caudoviricetes__na;Caudoviricetes__na;Caudoviricetes__na;Siphoviridae_sp._ctrG012" 0 0 0 0 0 0 0 0 0 0 0 0
0 0 0 0 0 0 0 0 0 0 0 0 27
13 14 1 1 0 0 0 0 0 0 0 0 0
0 0 0 0 0 0 0 0 0 0 0 0 0
0 0 0 0 0 0 0 0 0 0 0 0 0
0 0 0 0 0 0 0 0
"Viruses;Uroviricota;Caudoviricetes;Caudoviricetes__na;Caudoviricetes__na;Caudoviricetes__na;Siphoviridae_sp._ctRg61" 0 0 0 0 0 0 0 0 0 0 0 0
0 0 0 0 0 0 0 0 0 0 0 0 0
0 0 0 0 0 1 0 0 0 0 0 0 0
0 0 0 0 0 0 0 0 0 0 0 0 0
0 0 0 1 0 0 0 0 0 0 0 0 0
0 0 0 0 0 0 0 0
"Viruses;Uroviricota;Caudoviricetes;Caudoviricetes__na;Caudoviricetes__na;Caudoviricetes__na;Siphoviridae_sp._ctRg81" 0 0 0 0 0 0 0 0 0 0 0 0
0 0 0 0 0 0 0 0 0 0 0 0 0
0 0 0 0 0 0 0 0 0 0 0 0 0
0 0 0 0 0 0 0 0 0 0 1 0 0
0 0 0 0 0 0 0 0 0 2 0 0 0
0 0 0 0 0 0 0 0
"Viruses;Uroviricota;Caudoviricetes;Caudoviricetes__na;Caudoviricetes__na;Caudoviricetes__na;Siphoviridae_sp._ctrgQ8" 0 0 0 0 0 0 0 0 0 0 0 0
3 3 0 0 0 0 5 9 7 5 0 1 2
9 1 0 1 0 4 1 0 0 0 0 2 3
0 0 0 0 38 14 0 1 0 0 18 8 0
0 0 0 0 0 0 0 0 0 80 18 0 0
0 0 7 3 0 0 0 0
"Viruses;Uroviricota;Caudoviricetes;Caudoviricetes__na;Caudoviricetes__na;Caudoviricetes__na;Siphoviridae_sp._ctrgt10" 0 0 0 0 0 0 0 0 0 0 0 0
0 0 0 0 0 0 0 0 0 0 0 0 0
0 0 0 0 0 0 0 0 0 0 0 0 0
0 0 0 0 0 1 0 0 0 0 0 0 0
0 0 0 0 0 0 0 0 0 0 0 0 0
0 0 1 0 0 0 0 0
"Viruses;Uroviricota;Caudoviricetes;Caudoviricetes__na;Caudoviricetes__na;Caudoviricetes__na;Siphoviridae_sp._ctRGt12" 0 0 0 0 0 0 0 0 0 0 0 0
0 1 0 0 0 0 1 2 0 0 0 0 0
0 0 0 0 0 0 0 0 0 0 0 1 0
0 0 0 0 0 1 0 0 0 0 0 1 0

0 0 0 0 2 0 0 0 0 0 1 0 0
0 0 2 0 0 0 0 0
"Viruses;Uroviricota;Caudoviricetes;Caudoviricetes__na;Caudoviricetes__na;Caudoviricetes__na;Siphoviridae_s
p._ctrKX6" 0 0 0 0 0 0 0 0 0 0 0 0
2 0 0 0 0 0 0 0 0 0 0 0 0
0 0 0 0 0 0 0 0 0 0 0 0 0
0 0 0 0 0 0 0 0 0 0 0 0 0
0 0 0 13 11 0 0 0 0 0 0 0 0
0 0 0 0 0 0 0 0
"Viruses;Uroviricota;Caudoviricetes;Caudoviricetes__na;Caudoviricetes__na;Caudoviricetes__na;Siphoviridae_s
p._ctRlj31" 0 0 0 0 0 0 0 0 0 0 0 0
0 0 0 0 0 0 0 0 0 0 0 0 0
0 0 0 0 0 1 1 0 0 1 0 0 0
0 0 0 0 0 0 0 0 0 0 0 0 0
0 0 0 2 0 0 0 0 0 0 1 0 0
0 0 1 0 0 0 0 0
"Viruses;Uroviricota;Caudoviricetes;Caudoviricetes__na;Caudoviricetes__na;Caudoviricetes__na;Siphoviridae_s
p._ctrok7" 0 0 0 0 0 0 0 0 0 0 0 0
0 0 0 0 0 0 0 0 0 0 0 0 0
0 3 0 4 0 0 0 0 0 0 0 0 0
0 0 0 0 0 2 8 0 0 0 0 0 0
0 0 0 0 1 0 0 0 0 0 0 0 0
0 0 0 1 0 0 0 0
"Viruses;Uroviricota;Caudoviricetes;Caudoviricetes__na;Caudoviricetes__na;Caudoviricetes__na;Siphoviridae_s
p._ctRon5" 0 0 0 0 1 0 0 0 0 0 0 0
2 1 0 0 0 0 4 6 0 0 0 0 0
3 0 0 0 0 1 1 0 0 0 0 6 1
0 0 0 0 0 0 0 0 0 0 1 1 0
0 0 0 0 0 0 0 0 0 2 5 0 0
0 0 4 6 0 0 0 0
"Viruses;Uroviricota;Caudoviricetes;Caudoviricetes__na;Caudoviricetes__na;Caudoviricetes__na;Siphoviridae_s
p._ctrpM6" 0 0 0 0 0 0 0 0 0 0 0 0
0 0 0 0 0 0 0 0 0 0 0 0 0
0 0 0 0 0 12 6 0 0 0 0 0 0
0 0 0 0 0 0 0 0 0 0 0 0 0
0 0 0 2 2 0 0 0 0 0 0 0 0
0 0 0 0 0 0 0 0
"Viruses;Uroviricota;Caudoviricetes;Caudoviricetes__na;Caudoviricetes__na;Caudoviricetes__na;Siphoviridae_s
p._ctRuT6" 0 0 0 0 0 0 0 0 0 0 0 0
0 0 0 0 0 0 0 0 0 0 0 0 0
0 0 0 0 0 0 0 0 0 5 0 3 0
0 0 0 0 0 0 0 0 0 0 0 0 0
0 0 0 0 0 0 0 0 0 0 0 0 0
0 0 0 0 0 0 0 0
"Viruses;Uroviricota;Caudoviricetes;Caudoviricetes__na;Caudoviricetes__na;Caudoviricetes__na;Siphoviridae_s
p._ctS0613" 0 0 0 0 0 0 0 0 0 0 0 0
0 0 0 0 0 0 0 0 0 0 0 0 0
0 0 0 0 0 0 0 0 0 0 0 0 0
0 0 0 0 0 0 0 0 0 0 0 0 0

0 0 0 0 0 0 0 0 0 0 0 0 0
0 0 1 1 0 0 0 0
"Viruses;Uroviricota;Caudoviricetes;Caudoviricetes__na;Caudoviricetes__na;Caudoviricetes__na;Siphoviridae_sp._ctS1E53" 0 0 0 0 0 0 0 0 0 0 0 0
0 0 0 0 0 0 0 0 0 0 0 0 0
0 0 0 0 0 0 0 0 0 0 0 2 0
0 0 0 0 0 0 0 0 0 0 0 0 0
0 0 0 0 0 0 0 0 0 0 0 0 0
0 0 1 1 0 0 0 0
"Viruses;Uroviricota;Caudoviricetes;Caudoviricetes__na;Caudoviricetes__na;Caudoviricetes__na;Siphoviridae_sp._ctS2049" 0 0 0 0 0 0 0 0 0 0 0 0
0 0 0 0 0 0 0 0 0 0 0 0 0
0 0 0 0 0 0 0 0 0 0 0 2 0
0 0 0 0 0 0 0 0 0 0 0 0 0
0 0 0 0 0 0 0 0 0 0 0 0 0
0 0 0 0 0 0 0 0
"Viruses;Uroviricota;Caudoviricetes;Caudoviricetes__na;Caudoviricetes__na;Caudoviricetes__na;Siphoviridae_sp._ctS3r5" 0 0 0 0 0 0 0 0 0 0 0 0
1 0 0 0 0 0 0 0 0 0 0 0 0
0 0 0 0 0 0 0 0 0 0 0 0 0
0 0 0 0 0 0 0 0 0 0 0 0 0
0 0 0 0 0 0 0 0 0 0 0 0 0
0 0 0 0 0 0 0 0
"Viruses;Uroviricota;Caudoviricetes;Caudoviricetes__na;Caudoviricetes__na;Caudoviricetes__na;Siphoviridae_sp._ctSA812" 0 0 0 0 0 0 0 0 0 0 0 0
3 5 0 0 0 0 6 1 0 0 0 1 3
2 0 0 0 0 0 1 0 0 0 0 0 0
0 0 0 0 0 0 0 0 0 0 2 1 0
0 0 0 3 10 0 0 0 0 0 0 0 0
0 0 0 1 0 0 0 0
"Viruses;Uroviricota;Caudoviricetes;Caudoviricetes__na;Caudoviricetes__na;Caudoviricetes__na;Siphoviridae_sp._ctsAY3" 0 0 0 0 0 0 0 0 0 0 0 0
2 3 0 0 0 0 0 0 0 0 0 0 2
0 0 0 0 0 0 0 0 0 0 0 0 0
0 0 0 0 0 0 0 0 0 0 0 0 0
0 0 0 2 5 0 0 0 0 0 0 0 0
0 0 0 1 0 0 0 0
"Viruses;Uroviricota;Caudoviricetes;Caudoviricetes__na;Caudoviricetes__na;Caudoviricetes__na;Siphoviridae_sp._ctsfh5" 0 0 0 0 0 0 0 0 0 0 0 0
0 0 0 0 0 0 0 0 0 0 0 0 0
0 0 0 0 0 0 0 0 0 0 0 0 0
0 0 0 0 0 0 0 0 0 0 0 0 0
0 0 0 1 0 0 0 0 0 0 0 0 0
0 0 0 0 0 0 0 0
"Viruses;Uroviricota;Caudoviricetes;Caudoviricetes__na;Caudoviricetes__na;Caudoviricetes__na;Siphoviridae_sp._ctsgc4" 0 0 0 0 0 0 0 1 0 0 0 0
0 0 0 0 0 0 0 0 0 0 0 0 0
0 0 0 0 0 0 0 0 0 0 0 0 0
0 0 0 0 0 0 0 0 0 0 0 0 0

0 0 0 0 0 0 0 0 0 0 0 0 0
0 0 0 0 0 0 0 0
"Viruses;Uroviricota;Caudoviricetes;Caudoviricetes__na;Caudoviricetes__na;Caudoviricetes__na;Siphoviridae_s
p._ctsIQ24" 0 0 0 0 0 0 0 0 0 0 0 0
1 0 0 0 0 0 0 0 0 0 0 0 0
0 0 0 0 0 0 0 0 0 0 0 0 0
0 0 0 0 0 0 0 0 0 0 0 0 0
0 0 0 0 0 0 0 0 0 0 0 0 0
0 0 0 0 0 0 0 0
"Viruses;Uroviricota;Caudoviricetes;Caudoviricetes__na;Caudoviricetes__na;Caudoviricetes__na;Siphoviridae_s
p._ctSMg55" 0 0 0 0 12 0 0 0 0 0 0 0
0 0 0 0 0 0 0 0 0 0 0 0 0
0 0 0 0 0 0 0 0 0 0 0 1 0
0 0 0 19 0 0 0 0 0 0 0 0 0
0 1 0 1 0 0 0 0 0 0 0 0 0
0 0 0 0 0 0 0 0
"Viruses;Uroviricota;Caudoviricetes;Caudoviricetes__na;Caudoviricetes__na;Caudoviricetes__na;Siphoviridae_s
p._ctsMn4" 0 0 0 0 0 0 0 0 0 0 0 0
0 0 0 0 0 0 0 0 0 0 0 0 3
2 6 0 0 0 0 0 0 0 0 0 0 0
0 0 0 0 0 0 0 0 0 0 0 0 0
0 0 0 0 0 0 0 0 0 0 0 0 0
0 0 1 0 0 0 0 0
"Viruses;Uroviricota;Caudoviricetes;Caudoviricetes__na;Caudoviricetes__na;Caudoviricetes__na;Siphoviridae_s
p._ctsoB6" 0 0 0 0 0 0 0 0 0 0 0 0
2 1 0 0 0 0 0 1 0 0 0 0 0
1 0 0 0 0 1 0 0 0 0 0 4 2
0 0 0 0 0 0 0 0 0 0 10 14 1
0 0 0 3 7 0 0 0 0 2 0 0 0
0 0 0 5 0 0 0 0
"Viruses;Uroviricota;Caudoviricetes;Caudoviricetes__na;Caudoviricetes__na;Caudoviricetes__na;Siphoviridae_s
p._ctSOv1" 0 0 0 0 0 0 0 0 0 0 0 0
0 2 0 0 0 0 0 0 0 0 0 0 1
0 0 0 0 0 0 0 0 0 0 0 0 0
0 0 0 0 0 0 0 0 0 0 0 1 0
0 0 0 0 0 0 0 0 0 0 0 0 0
0 0 0 0 0 0 0 0
"Viruses;Uroviricota;Caudoviricetes;Caudoviricetes__na;Caudoviricetes__na;Caudoviricetes__na;Siphoviridae_s
p._ctss15" 0 0 0 0 0 0 0 0 0 0 0 0
0 0 0 0 0 0 0 0 0 0 0 0 0
0 0 0 0 0 0 0 0 0 0 0 0 0
0 0 0 0 0 0 0 0 0 0 0 0 0
0 0 0 0 0 0 0 0 0 0 0 0 0
0 0 1 0 0 0 0 0
"Viruses;Uroviricota;Caudoviricetes;Caudoviricetes__na;Caudoviricetes__na;Caudoviricetes__na;Siphoviridae_s
p._ctSuy3" 0 0 0 0 0 0 0 0 0 0 0 0
0 0 0 0 0 0 0 0 0 0 0 0 0
0 0 0 0 0 0 0 0 0 0 0 3 3
0 0 0 0 0 0 0 0 0 0 0 0 0

0 0 0 0 0 0 0 0 0 0 0 0 0
0 0 0 0 0 0 0 0
"Viruses;Uroviricota;Caudoviricetes;Caudoviricetes__na;Caudoviricetes__na;Caudoviricetes__na;Siphoviridae_sp._ctSx228" 0 0 0 0 0 0 0 0 0 0 0 0
0 0 1 0 0 0 0 0 0 0 0 0 0
0 0 0 0 0 0 0 0 0 0 0 0 0
0 0 0 0 0 0 1 0 0 0 0 0 0
0 0 0 0 0 0 0 0 0 0 0 0 0
0 0 0 0 0 0 0 0
"Viruses;Uroviricota;Caudoviricetes;Caudoviricetes__na;Caudoviricetes__na;Caudoviricetes__na;Siphoviridae_sp._ctsxw88" 0 0 0 0 0 0 0 0 0 0 0 0
0 0 0 0 0 0 0 0 0 0 1 0 0
0 0 0 0 0 0 0 0 0 0 0 0 0
0 0 0 0 0 0 0 0 0 0 0 0 0
0 0 0 0 0 0 0 0 0 0 0 0 0
0 0 0 0 0 0 0 0
"Viruses;Uroviricota;Caudoviricetes;Caudoviricetes__na;Caudoviricetes__na;Caudoviricetes__na;Siphoviridae_sp._ctsYb1" 0 0 0 0 0 0 0 0 0 0 0 0
0 0 0 0 0 0 0 0 0 0 0 0 0
0 0 0 0 0 0 0 0 0 0 0 0 0
0 0 0 0 0 0 0 0 0 0 3 0 0
0 0 0 0 0 0 0 0 0 1 0 0 0
0 0 0 0 0 0 0 0
"Viruses;Uroviricota;Caudoviricetes;Caudoviricetes__na;Caudoviricetes__na;Caudoviricetes__na;Siphoviridae_sp._ctt0c4" 0 0 0 0 0 0 0 0 0 0 0 0
0 0 0 1 0 0 0 0 0 0 0 0 0
0 0 0 0 0 0 0 0 0 0 0 0 0
0 0 0 0 0 0 0 0 0 0 0 0 0
0 0 0 0 0 0 0 0 0 0 0 0 0
0 0 0 0 0 0 0 0
"Viruses;Uroviricota;Caudoviricetes;Caudoviricetes__na;Caudoviricetes__na;Caudoviricetes__na;Siphoviridae_sp._cttb18" 0 0 0 0 0 0 0 0 0 0 0 0
0 0 0 0 0 0 0 0 0 0 0 0 0
0 0 0 0 0 0 0 0 0 0 0 0 0
0 0 0 0 0 0 0 0 0 1 0 0 0
0 0 0 0 0 0 0 0 0 0 0 0 0
0 0 0 0 0 0 0 0
"Viruses;Uroviricota;Caudoviricetes;Caudoviricetes__na;Caudoviricetes__na;Caudoviricetes__na;Siphoviridae_sp._ctTBd21" 0 0 0 0 0 0 0 0 0 0 0 0
0 0 0 0 0 0 0 0 0 0 0 0 2
2 0 0 0 0 0 0 0 0 0 0 0 0
0 0 0 0 0 1 0 0 0 0 0 0 0
0 0 0 0 0 0 0 0 0 0 0 0 0
0 0 0 0 0 0 0 0
"Viruses;Uroviricota;Caudoviricetes;Caudoviricetes__na;Caudoviricetes__na;Caudoviricetes__na;Siphoviridae_sp._ctTC45" 0 0 0 0 0 0 0 0 0 0 0 0
0 1 4 0 1 0 0 0 0 0 0 0 1
4 0 0 0 0 0 2 0 0 0 0 0 0
0 0 0 0 0 7 9 0 0 0 0 0 0

0 0 0 3 8 0 0 0 0 1 1 0 0
0 0 0 1 0 0 0 0
"Viruses;Uroviricota;Caudoviricetes;Caudoviricetes__na;Caudoviricetes__na;Caudoviricetes__na;Siphoviridae_s
p._cttDR14" 0 0 0 0 0 0 0 0 0 0 0 0
2 0 0 0 0 0 0 0 0 0 0 0 0
0 0 0 0 0 0 0 0 0 0 0 0 0
0 0 0 0 0 0 0 0 0 0 0 0 0
0 0 0 0 0 0 0 0 0 0 0 0 0
0 0 0 0 0 0 0 0
"Viruses;Uroviricota;Caudoviricetes;Caudoviricetes__na;Caudoviricetes__na;Caudoviricetes__na;Siphoviridae_s
p._cttFh17" 0 0 0 0 1 0 0 0 0 0 0 0
0 0 0 0 0 0 0 1 0 0 0 0 0
0 2 0 2 0 0 1 0 0 0 0 0 1
0 0 0 0 0 0 2 0 0 0 0 1 0
0 0 0 0 0 0 0 0 0 2 3 0 0
0 0 0 0 0 0 0 0
"Viruses;Uroviricota;Caudoviricetes;Caudoviricetes__na;Caudoviricetes__na;Caudoviricetes__na;Siphoviridae_s
p._cttG313" 0 0 0 0 0 0 0 0 0 0 0 0
0 0 0 0 0 0 0 0 0 0 0 0 0
0 0 0 0 0 0 0 0 0 0 3 0 0
0 0 0 0 0 0 0 0 0 0 1 0 0
0 0 0 0 0 0 0 0 0 0 0 0 0
0 0 0 0 0 0 0 0
"Viruses;Uroviricota;Caudoviricetes;Caudoviricetes__na;Caudoviricetes__na;Caudoviricetes__na;Siphoviridae_s
p._ctTgb17" 0 0 0 0 0 0 0 0 0 0 0 0
0 0 0 0 0 0 0 0 0 0 0 0 0
0 0 0 0 0 2 2 0 0 0 0 0 0
0 0 0 0 9 81 2 1 0 0 38 29 0
0 0 0 0 0 0 0 0 0 4 1 0 0
0 0 0 1 0 0 0 0
"Viruses;Uroviricota;Caudoviricetes;Caudoviricetes__na;Caudoviricetes__na;Caudoviricetes__na;Siphoviridae_s
p._ctTkm23" 0 0 0 0 0 0 0 0 0 0 0 0
1 5 0 0 0 0 0 0 0 0 0 0 0
0 0 0 0 0 0 0 0 0 0 0 0 0
0 0 0 0 0 0 0 0 0 0 0 0 0
0 0 0 0 0 0 0 0 0 0 0 0 0
0 0 0 0 0 0 0 0
"Viruses;Uroviricota;Caudoviricetes;Caudoviricetes__na;Caudoviricetes__na;Caudoviricetes__na;Siphoviridae_s
p._cttma3" 0 0 0 0 0 0 0 0 0 0 0 0
0 0 0 0 0 0 0 0 0 0 0 0 0
0 0 0 0 0 0 1 0 0 0 0 0 0
0 0 0 0 0 0 0 0 0 0 0 0 0
0 0 0 0 0 0 0 0 0 0 0 0 0
0 0 0 0 0 0 0 0
"Viruses;Uroviricota;Caudoviricetes;Caudoviricetes__na;Caudoviricetes__na;Caudoviricetes__na;Siphoviridae_s
p._ctTPJ4" 0 0 0 0 0 0 0 0 0 0 0 0
0 0 0 0 0 0 0 0 0 0 0 0 0
0 0 0 0 0 0 0 0 0 0 0 0 0
1 0 0 0 0 0 0 0 0 0 0 0 0

0 0 0 0 0 0 0 0 0 0 0 0 0
0 0 0 0 0 0 0 0
"Viruses;Uroviricota;Caudoviricetes;Caudoviricetes__na;Caudoviricetes__na;Caudoviricetes__na;Siphoviridae_s
p._cttpk5" 0 0 0 0 0 0 0 0 0 0 0 0
0 0 0 0 0 0 0 0 0 0 0 0 0
0 0 0 0 0 0 0 0 0 0 0 0 0
0 0 0 0 0 0 0 0 0 0 0 0 0
0 0 0 2 1 0 0 0 0 0 0 0 0
0 0 0 0 0 0 0 0
"Viruses;Uroviricota;Caudoviricetes;Caudoviricetes__na;Caudoviricetes__na;Caudoviricetes__na;Siphoviridae_s
p._ctTqe2" 0 0 0 0 0 0 0 0 0 0 0 0
0 0 0 0 0 0 0 0 0 0 0 0 0
0 0 0 0 0 0 0 0 0 0 0 24 0
0 0 0 0 0 0 0 0 0 0 0 0 0
0 0 0 0 0 0 0 0 0 0 0 0 0
0 0 7 8 0 0 0 0
"Viruses;Uroviricota;Caudoviricetes;Caudoviricetes__na;Caudoviricetes__na;Caudoviricetes__na;Siphoviridae_s
p._ctTrb4" 0 0 0 0 0 0 0 0 0 0 0 0
0 0 0 0 0 0 2 1 0 0 0 0 0
0 0 0 0 0 1 1 0 0 0 0 11 2
0 0 0 0 0 0 0 0 0 0 0 0 0
0 0 0 0 1 0 0 0 0 50 18 0 0
0 0 1 3 0 0 0 0
"Viruses;Uroviricota;Caudoviricetes;Caudoviricetes__na;Caudoviricetes__na;Caudoviricetes__na;Siphoviridae_s
p._cttuu15" 0 0 0 0 0 0 0 0 0 0 0 0
0 0 0 0 0 0 0 0 0 0 0 0 0
0 0 0 0 0 0 0 0 0 0 0 0 0
0 0 2 0 0 1 0 0 0 0 0 1 0
0 0 0 0 0 0 0 0 0 0 0 0 0
0 0 2 3 0 0 0 0
"Viruses;Uroviricota;Caudoviricetes;Caudoviricetes__na;Caudoviricetes__na;Caudoviricetes__na;Siphoviridae_s
p._cttWj13" 0 0 0 0 0 0 0 0 0 0 0 0
0 0 0 0 0 0 0 0 0 0 0 0 0
0 0 0 0 0 17 24 0 0 1 0 41 29
0 0 0 0 0 0 0 0 0 0 1 0 0
0 1 0 0 1 3 0 0 0 0 0 0 0
0 0 0 0 0 0 0 0
"Viruses;Uroviricota;Caudoviricetes;Caudoviricetes__na;Caudoviricetes__na;Caudoviricetes__na;Siphoviridae_s
p._ctTXt1" 0 0 0 0 0 0 0 0 0 0 0 0
0 0 0 0 0 0 0 0 0 0 0 0 0
0 0 0 0 0 0 0 0 0 0 0 0 0
0 0 0 0 6 5 1 0 0 0 0 0 0
0 0 0 0 0 0 0 0 0 0 0 0 0
0 0 0 0 0 0 0 0
"Viruses;Uroviricota;Caudoviricetes;Caudoviricetes__na;Caudoviricetes__na;Caudoviricetes__na;Siphoviridae_s
p._ctu061" 0 0 0 0 0 0 0 0 0 0 0 0
0 1 0 0 0 0 0 0 0 0 0 0 0
0 0 0 0 0 0 0 0 0 0 0 0 0
0 0 0 0 0 0 0 0 0 0 0 0 0

0 0 0 0 0 0 0 0 0 0 0 0 0
0 0 0 0 0 0 0 0
"Viruses;Uroviricota;Caudoviricetes;Caudoviricetes__na;Caudoviricetes__na;Caudoviricetes__na;Siphoviridae_s
p._ctu3K14" 0 0 0 0 0 0 0 0 0 0 0 0
0 0 0 0 0 0 0 0 0 0 0 0 0
0 0 0 0 0 0 1 0 0 0 0 0 0
0 0 0 0 0 0 0 0 0 0 4 2 0
0 0 0 0 1 0 0 0 0 0 0 0 0
0 0 0 0 0 0 0 0
"Viruses;Uroviricota;Caudoviricetes;Caudoviricetes__na;Caudoviricetes__na;Caudoviricetes__na;Siphoviridae_s
p._ctu9a31" 0 0 0 0 0 0 0 0 0 0 0 0
0 0 0 0 0 0 0 0 0 0 0 0 0
0 0 0 0 0 0 0 0 0 0 0 0 0
0 0 0 0 2 1 2 0 0 0 0 0 0
0 0 8 4 2 0 0 0 0 0 0 0 0
0 0 1 0 0 0 0 0
"Viruses;Uroviricota;Caudoviricetes;Caudoviricetes__na;Caudoviricetes__na;Caudoviricetes__na;Siphoviridae_s
p._ctUGR26" 0 0 0 0 0 0 0 0 0 0 0 0
0 0 0 0 0 0 0 0 0 0 0 0 0
0 0 0 0 0 0 0 0 0 0 0 0 0
0 0 0 0 0 0 0 0 0 0 0 0 0
0 0 0 0 1 0 0 0 0 0 0 0 0
0 0 0 0 0 0 0 0
"Viruses;Uroviricota;Caudoviricetes;Caudoviricetes__na;Caudoviricetes__na;Caudoviricetes__na;Siphoviridae_s
p._ctUi914" 0 0 0 0 0 0 0 0 0 0 0 0
0 0 0 0 0 0 0 0 0 0 0 0 0
0 0 0 0 0 0 0 0 0 0 0 0 0
0 0 0 0 0 0 0 0 0 0 0 0 0
0 0 0 1 0 0 0 0 0 0 0 0 0
0 0 0 0 0 0 0 0
"Viruses;Uroviricota;Caudoviricetes;Caudoviricetes__na;Caudoviricetes__na;Caudoviricetes__na;Siphoviridae_s
p._ctUir1" 0 0 0 0 0 0 0 0 0 0 0 0
0 0 0 0 0 0 0 0 0 0 0 0 0
0 0 0 0 0 0 3 0 0 0 0 0 0
0 0 0 0 0 0 0 0 0 0 0 0 0
0 0 0 1 0 0 0 0 0 0 0 0 0
0 0 0 0 0 0 0 0
"Viruses;Uroviricota;Caudoviricetes;Caudoviricetes__na;Caudoviricetes__na;Caudoviricetes__na;Siphoviridae_s
p._ctUoe7" 0 0 0 0 0 0 0 0 0 0 0 0
0 0 0 0 0 0 0 0 0 0 0 0 0
0 0 0 0 0 0 0 0 0 0 0 0 0
0 0 0 0 0 0 0 0 0 0 0 0 0
0 0 0 0 0 0 0 0 0 0 0 0 0
0 0 3 1 0 0 0 0
"Viruses;Uroviricota;Caudoviricetes;Caudoviricetes__na;Caudoviricetes__na;Caudoviricetes__na;Siphoviridae_s
p._ctuOq1" 0 0 0 0 0 0 0 0 0 0 0 0
0 0 0 0 0 0 2 0 0 0 0 0 2
1 0 0 0 0 0 0 0 0 0 0 0 0
0 0 0 0 0 0 0 0 0 0 0 0 0

0 0 0 0 0 0 0 0 0 4 0 0 0
0 0 1 0 0 0 0 0
"Viruses;Uroviricota;Caudoviricetes;Caudoviricetes__na;Caudoviricetes__na;Caudoviricetes__na;Siphoviridae_sp._ctur44" 0 0 0 0 0 0 0 0 0 0 0 0
0 0 0 0 0 2 0 0 0 0 0 0 0
0 0 0 0 0 0 0 0 0 0 0 0 0
0 0 0 0 0 0 0 0 0 0 0 0 0
0 0 0 0 1 0 0 0 0 0 0 0 0
0 0 0 0 0 0 0 0
"Viruses;Uroviricota;Caudoviricetes;Caudoviricetes__na;Caudoviricetes__na;Caudoviricetes__na;Siphoviridae_sp._ctutT7" 0 0 0 0 0 0 0 0 0 0 0 0
0 0 0 0 0 0 0 0 0 0 0 0 0
0 0 0 0 0 0 0 0 0 0 0 0 0
0 0 0 0 0 0 0 0 0 0 0 0 0
0 0 0 0 1 0 0 0 0 0 0 0 0
0 0 0 0 0 0 0 0
"Viruses;Uroviricota;Caudoviricetes;Caudoviricetes__na;Caudoviricetes__na;Caudoviricetes__na;Siphoviridae_sp._ctuyW65" 0 0 0 0 0 0 0 0 1 0 0 0
0 0 0 0 0 0 0 0 0 0 0 0 0
0 0 0 0 0 0 0 0 0 0 0 0 0
0 0 0 0 0 0 0 0 0 0 0 0 0
0 0 0 0 0 0 0 0 0 0 0 0 0
0 0 0 0 0 0 0 0
"Viruses;Uroviricota;Caudoviricetes;Caudoviricetes__na;Caudoviricetes__na;Caudoviricetes__na;Siphoviridae_sp._ctv2R2" 0 0 0 0 0 0 0 0 0 0 0 0
0 0 0 0 0 0 0 0 0 0 0 0 1
5 0 0 0 0 0 0 0 0 79 0 0 0
0 0 0 0 0 0 0 0 0 0 0 0 0
0 0 0 0 0 0 0 0 0 0 0 0 0
0 0 0 0 0 0 0 0
"Viruses;Uroviricota;Caudoviricetes;Caudoviricetes__na;Caudoviricetes__na;Caudoviricetes__na;Siphoviridae_sp._ctvbt38" 0 0 0 0 0 0 0 0 0 0 0 0
0 0 0 0 0 0 0 0 0 0 0 0 0
0 0 0 0 0 0 0 0 0 0 0 0 0
0 0 0 0 0 0 0 0 0 0 0 0 0
0 0 0 0 0 0 0 0 0 0 0 0 0
0 0 0 4 0 0 0 0
"Viruses;Uroviricota;Caudoviricetes;Caudoviricetes__na;Caudoviricetes__na;Caudoviricetes__na;Siphoviridae_sp._ctvBz3" 0 0 0 0 0 0 0 0 0 0 0 0
0 0 0 0 0 0 6 26 1 0 1 0 0
0 0 0 0 0 0 1 0 0 0 0 0 0
0 0 0 0 0 0 0 0 0 0 0 0 0
0 0 0 0 0 0 0 0 0 0 0 0 0
0 0 0 0 0 0 0 0
"Viruses;Uroviricota;Caudoviricetes;Caudoviricetes__na;Caudoviricetes__na;Caudoviricetes__na;Siphoviridae_sp._ctvf68" 0 0 0 0 0 0 0 0 0 0 0 0
0 0 0 0 0 0 0 0 0 0 0 0 0
0 0 0 0 0 0 0 0 0 0 0 4 0
0 0 0 0 0 0 0 0 0 0 0 0 0

0 0 0 0 1 0 0 0 0 0 0 0 0
0 0 0 0 0 0 0 0
"Viruses;Uroviricota;Caudoviricetes;Caudoviricetes__na;Caudoviricetes__na;Caudoviricetes__na;Siphoviridae_sp._ctvFN21" 0 0 0 0 0 0 0 0 0 0 0 0
0 0 0 0 0 0 0 0 0 0 0 0 0
0 0 0 0 0 0 0 0 0 0 0 0 0
0 0 0 0 0 0 0 0 0 0 0 0 0
0 0 0 0 0 0 0 0 0 0 0 0 0
0 0 0 1 0 0 0 0
"Viruses;Uroviricota;Caudoviricetes;Caudoviricetes__na;Caudoviricetes__na;Caudoviricetes__na;Siphoviridae_sp._ctvhu9" 0 0 0 0 0 0 0 0 0 0 0 0
3 6 2 0 0 7 0 2 0 0 0 0 0
1 0 0 0 0 2 3 0 0 0 0 17 2
0 0 0 0 0 0 0 0 0 0 0 1 0
0 0 0 4 5 0 0 0 0 0 0 0 0
0 0 4 8 0 0 0 0
"Viruses;Uroviricota;Caudoviricetes;Caudoviricetes__na;Caudoviricetes__na;Caudoviricetes__na;Siphoviridae_sp._ctvI513" 0 0 0 0 0 0 0 0 0 0 0 0
0 0 0 0 0 0 0 0 0 0 0 0 0
0 0 0 0 0 0 0 0 0 0 0 0 0
0 0 0 0 0 0 0 0 0 0 0 0 0
0 0 1 0 0 0 0 0 0 0 0 0 0
0 0 0 0 0 0 0 0
"Viruses;Uroviricota;Caudoviricetes;Caudoviricetes__na;Caudoviricetes__na;Caudoviricetes__na;Siphoviridae_sp._ctVJE9" 0 0 0 0 0 0 0 0 0 0 0 0
1 0 0 0 0 0 0 0 0 0 0 0 0
1 0 0 0 0 0 0 0 0 0 0 0 1
0 0 0 0 1 0 0 0 0 0 0 0 0
0 0 0 10 15 0 0 0 0 0 0 0 0
0 0 0 0 0 0 0 0
"Viruses;Uroviricota;Caudoviricetes;Caudoviricetes__na;Caudoviricetes__na;Caudoviricetes__na;Siphoviridae_sp._ctvNP11" 0 0 0 0 0 0 0 0 0 0 0 0
0 0 0 0 0 0 0 0 0 0 0 0 0
0 0 0 0 0 0 0 0 0 0 0 0 0
0 0 0 0 0 0 0 0 0 0 0 0 0
0 0 0 0 1 0 0 0 0 0 1 0 0
0 0 0 0 0 0 0 0
"Viruses;Uroviricota;Caudoviricetes;Caudoviricetes__na;Caudoviricetes__na;Caudoviricetes__na;Siphoviridae_sp._ctvok7" 0 0 0 0 0 0 0 0 0 0 0 0
1 3 1 0 2 0 0 0 0 0 0 0 0
0 0 0 1 0 0 0 0 0 0 0 0 0
0 0 0 0 0 0 0 0 0 0 0 0 0
0 0 0 0 2 0 0 0 0 0 0 0 0
0 0 1 0 0 0 0 0
"Viruses;Uroviricota;Caudoviricetes;Caudoviricetes__na;Caudoviricetes__na;Caudoviricetes__na;Siphoviridae_sp._ctVOP12" 0 0 0 0 0 0 0 0 0 0 0 0
0 0 0 0 0 0 1 0 0 0 0 0 0
3 1 0 0 0 0 0 0 0 0 0 0 0
0 0 0 0 0 0 0 0 0 0 0 0 0

0 0 0 1 1 0 0 0 0 0 0 0 0
0 0 0 2 0 0 0 0
"Viruses;Uroviricota;Caudoviricetes;Caudoviricetes__na;Caudoviricetes__na;Caudoviricetes__na;Siphoviridae_s
p._ctVqj4" 0 0 0 0 0 0 0 0 0 0 0 0
0 0 0 0 0 0 0 1 0 0 0 0 0
0 0 0 0 0 0 0 0 0 0 0 0 0
0 0 0 0 4 5 3 0 0 0 0 0 0
0 0 0 0 0 0 0 0 0 0 0 0 0
0 0 0 0 0 0 0 0
"Viruses;Uroviricota;Caudoviricetes;Caudoviricetes__na;Caudoviricetes__na;Caudoviricetes__na;Siphoviridae_s
p._ctvS314" 0 0 0 0 0 0 0 0 0 0 0 0
0 0 0 0 0 0 0 0 0 0 0 0 0
0 0 0 0 0 0 0 0 0 0 0 0 0
0 0 0 1 0 0 0 0 0 0 0 0 0
0 0 0 0 0 0 0 0 0 0 0 0 0
0 0 0 0 0 0 0 0
"Viruses;Uroviricota;Caudoviricetes;Caudoviricetes__na;Caudoviricetes__na;Caudoviricetes__na;Siphoviridae_s
p._ctvuW5" 0 0 0 0 0 0 0 0 0 0 0 0
0 0 0 0 0 0 0 0 0 0 0 0 0
0 0 0 0 0 0 0 0 0 0 0 1 6
0 0 0 0 12 5 9 1 0 0 0 0 0
0 0 0 0 0 0 0 0 0 0 0 0 0
0 0 0 0 0 0 0 0
"Viruses;Uroviricota;Caudoviricetes;Caudoviricetes__na;Caudoviricetes__na;Caudoviricetes__na;Siphoviridae_s
p._ctvWR21" 0 0 0 0 0 0 0 0 0 0 0 0
0 0 0 0 0 0 0 0 0 0 0 0 0
0 0 0 0 0 1 0 0 0 0 0 0 0
0 0 0 0 0 0 0 0 0 0 0 0 0
0 0 0 0 0 0 0 0 0 0 0 0 0
0 0 0 0 0 0 0 0
"Viruses;Uroviricota;Caudoviricetes;Caudoviricetes__na;Caudoviricetes__na;Caudoviricetes__na;Siphoviridae_s
p._ctvxh7" 0 0 0 0 0 0 0 0 0 0 0 0
0 0 0 0 0 4 0 0 0 0 0 0 0
0 0 0 0 0 0 0 0 0 0 0 0 0
0 0 0 0 0 0 0 0 0 0 0 0 0
0 0 0 7 4 0 0 0 0 0 0 0 0
0 0 0 0 0 0 0 0
"Viruses;Uroviricota;Caudoviricetes;Caudoviricetes__na;Caudoviricetes__na;Caudoviricetes__na;Siphoviridae_s
p._ctvyM23" 0 0 0 0 0 0 0 0 0 0 0 0
0 0 0 0 0 0 0 0 0 0 0 0 0
0 0 0 0 0 0 0 0 0 0 0 0 0
0 0 0 0 0 0 0 0 0 0 0 0 0
0 0 0 1 0 0 0 0 0 0 0 0 0
0 0 0 0 0 0 0 0
"Viruses;Uroviricota;Caudoviricetes;Caudoviricetes__na;Caudoviricetes__na;Caudoviricetes__na;Siphoviridae_s
p._ctW4q29" 0 0 0 0 0 0 0 0 0 0 0 0
0 0 0 0 0 0 0 0 0 0 0 0 0
1 1 0 0 0 0 1 0 0 0 0 2 0
0 0 0 87 0 0 0 0 0 0 0 0 0

0 0 1 0 2 0 0 0 0 0 0 0 0
0 0 2 1 0 1 0 0
"Viruses;Uroviricota;Caudoviricetes;Caudoviricetes__na;Caudoviricetes__na;Caudoviricetes__na;Siphoviridae_s
p._ctw757" 0 0 0 0 0 0 0 0 0 0 0 0
3 3 0 0 0 0 0 0 0 0 0 0 0
0 0 0 0 0 0 0 0 0 0 0 0 0
0 0 0 0 0 0 0 0 0 0 0 0 0
0 0 0 0 0 0 0 0 0 0 0 0 0
0 0 0 0 0 0 0 0
"Viruses;Uroviricota;Caudoviricetes;Caudoviricetes__na;Caudoviricetes__na;Caudoviricetes__na;Siphoviridae_s
p._ctWBz6" 0 0 0 0 0 0 0 0 0 0 0 0
0 0 0 0 0 0 0 0 0 0 0 0 0
0 0 0 0 0 0 0 0 0 0 0 0 0
0 0 0 0 0 0 0 0 0 0 0 0 0
0 0 101 0 0 0 0 0 0 0 0 0 0
0 0 0 4 0 0 0 0
"Viruses;Uroviricota;Caudoviricetes;Caudoviricetes__na;Caudoviricetes__na;Caudoviricetes__na;Siphoviridae_s
p._ctWdm1" 0 0 0 0 0 0 0 0 0 0 0 0
0 0 0 0 0 0 0 1 0 0 0 0 0
0 0 0 0 0 0 0 0 0 0 0 0 0
0 0 0 0 0 0 0 0 0 0 3 40 0
0 0 0 0 0 0 0 0 0 0 0 0 0
0 0 0 0 0 0 0 0
"Viruses;Uroviricota;Caudoviricetes;Caudoviricetes__na;Caudoviricetes__na;Caudoviricetes__na;Siphoviridae_s
p._ctWDo30" 0 0 0 0 0 0 0 0 0 0 0 0
0 0 0 0 0 0 0 0 0 0 0 0 0
0 0 0 0 0 0 0 0 0 0 0 0 0
0 0 0 0 0 1 1 0 0 0 0 0 0
0 0 0 0 0 0 0 0 0 0 0 0 0
0 0 0 0 0 0 0 0
"Viruses;Uroviricota;Caudoviricetes;Caudoviricetes__na;Caudoviricetes__na;Caudoviricetes__na;Siphoviridae_s
p._ctwDU14" 0 0 0 0 0 0 0 0 0 0 0 0
0 0 0 0 0 0 0 0 0 0 0 0 0
0 0 0 0 0 0 1 0 0 0 0 0 0
0 0 0 0 0 0 0 0 0 0 0 0 0
0 0 0 0 0 0 0 0 0 0 0 0 0
0 0 0 0 0 0 0 0
"Viruses;Uroviricota;Caudoviricetes;Caudoviricetes__na;Caudoviricetes__na;Caudoviricetes__na;Siphoviridae_s
p._ctWf32" 0 0 0 0 1 0 0 0 0 0 0 0
0 0 0 0 0 0 0 0 0 0 0 0 0
0 0 0 0 0 0 0 0 0 0 0 0 0
0 0 0 0 0 0 0 0 0 0 0 0 0
0 0 0 0 0 0 0 0 0 0 0 0 0
0 0 0 0 0 0 0 0
"Viruses;Uroviricota;Caudoviricetes;Caudoviricetes__na;Caudoviricetes__na;Caudoviricetes__na;Siphoviridae_s
p._ctwfx1" 0 0 0 0 0 0 0 0 0 0 0 0
0 0 0 0 0 0 0 0 0 0 0 0 0
0 0 0 0 0 0 0 0 0 0 0 0 0
0 0 0 0 0 0 0 0 0 0 0 0 0

0 0 0 0 0 0 0 0 0 0 0 0 0
0 0 0 3 0 0 0 0
"Viruses;Uroviricota;Caudoviricetes;Caudoviricetes__na;Caudoviricetes__na;Caudoviricetes__na;Siphoviridae_s
p._ctwHj1" 0 0 0 0 0 0 0 0 0 0 0 0
0 0 0 0 0 0 0 0 0 0 0 0 0
0 0 0 0 0 0 0 0 0 0 0 3 1
0 0 0 0 0 0 0 0 0 0 0 0 0
0 0 0 4 3 0 0 0 0 0 0 0 0
0 0 0 0 0 0 0 0
"Viruses;Uroviricota;Caudoviricetes;Caudoviricetes__na;Caudoviricetes__na;Caudoviricetes__na;Siphoviridae_s
p._ctWhl2" 0 0 0 0 0 0 0 0 0 0 0 0
0 0 0 0 0 0 0 0 0 0 0 0 57
62 2 0 0 0 0 0 0 0 0 0 6 6
0 0 0 0 0 1 0 0 0 0 1 0 0
0 0 0 1 1 0 0 0 0 0 0 0 0
0 0 10 42 0 2 0 0
"Viruses;Uroviricota;Caudoviricetes;Caudoviricetes__na;Caudoviricetes__na;Caudoviricetes__na;Siphoviridae_s
p._ctWKa2" 0 0 0 0 0 0 0 0 0 0 0 0
0 0 0 0 0 0 0 0 0 0 0 0 0
0 0 0 0 0 0 0 0 0 0 0 0 0
0 0 0 0 0 0 0 0 0 0 0 0 0
0 0 2 0 0 0 0 0 0 0 0 0 0
0 0 0 0 0 0 0 0
"Viruses;Uroviricota;Caudoviricetes;Caudoviricetes__na;Caudoviricetes__na;Caudoviricetes__na;Siphoviridae_s
p._ctWlk2" 0 0 0 0 0 0 0 0 0 0 0 0
0 0 0 0 0 0 0 0 0 0 0 0 0
0 0 0 0 0 0 0 0 0 0 0 2 1
0 0 0 0 78 53 2 0 0 0 0 0 0
0 0 5 0 0 0 0 0 0 0 0 0 0
0 0 3 0 0 1 0 0
"Viruses;Uroviricota;Caudoviricetes;Caudoviricetes__na;Caudoviricetes__na;Caudoviricetes__na;Siphoviridae_s
p._ctwns66" 0 0 0 0 0 0 0 0 0 0 0 0
11 5 26 18 5 17 36 42 1 1 1 0 0
0 2 0 0 0 2 1 0 0 0 0 0 0
0 0 2 0 26 13 2 0 0 0 0 0 0
0 0 0 0 0 0 0 0 0 2 1 0 0
0 0 0 0 0 0 0 0
"Viruses;Uroviricota;Caudoviricetes;Caudoviricetes__na;Caudoviricetes__na;Caudoviricetes__na;Siphoviridae_s
p._ctwQg18" 0 0 0 0 0 0 0 0 0 0 0 0
0 0 0 0 0 0 1 0 0 0 0 0 0
1 0 0 0 0 0 0 0 0 0 0 7 5
0 0 0 0 0 0 0 0 0 0 0 0 0
0 0 37 0 1 0 0 0 0 0 0 0 0
0 0 5 2 0 0 0 0
"Viruses;Uroviricota;Caudoviricetes;Caudoviricetes__na;Caudoviricetes__na;Caudoviricetes__na;Siphoviridae_s
p._ctwQT14" 0 0 0 0 0 0 0 0 0 0 0 0
0 0 0 0 0 0 0 0 0 0 0 0 0
0 0 0 0 0 0 0 0 0 0 0 1 1
0 0 0 0 0 0 0 0 0 0 0 0 0

0 0 0 0 0 0 0 0 0 0 0 0 0
0 0 0 0 0 0 0 0
"Viruses;Uroviricota;Caudoviricetes;Caudoviricetes__na;Caudoviricetes__na;Caudoviricetes__na;Siphoviridae_sp._ctwWa4" 0 0 0 0 0 0 0 0 0 0 0 0
3 0 1 0 0 0 2 9 0 0 0 0 0
0 0 0 0 0 2 2 0 0 0 0 0 1
0 0 0 0 0 0 0 0 0 0 1 1 0
0 0 0 1 2 0 0 0 0 0 0 0 0
0 0 0 1 0 0 0 0
"Viruses;Uroviricota;Caudoviricetes;Caudoviricetes__na;Caudoviricetes__na;Caudoviricetes__na;Siphoviridae_sp._ctWWc42" 0 0 0 0 0 0 0 0 0 0 0 0
0 0 0 0 0 0 0 0 0 0 0 0 0
0 0 0 0 0 0 0 0 0 2 0 0 0
0 0 0 0 0 0 0 0 0 0 0 0 0
0 1 0 0 0 0 0 0 0 0 0 0 0
0 0 1 0 0 1 0 0
"Viruses;Uroviricota;Caudoviricetes;Caudoviricetes__na;Caudoviricetes__na;Caudoviricetes__na;Siphoviridae_sp._ctwYi19" 0 0 0 0 0 0 0 0 0 0 0 0
0 0 0 0 0 0 0 0 0 0 0 0 0
0 0 0 0 0 0 0 0 0 0 0 0 0
0 0 0 2 0 0 0 0 0 0 0 0 0
0 0 0 0 0 0 0 0 0 0 0 0 0
0 0 0 0 0 0 0 0
"Viruses;Uroviricota;Caudoviricetes;Caudoviricetes__na;Caudoviricetes__na;Caudoviricetes__na;Siphoviridae_sp._ctwzt2" 0 0 0 0 0 0 0 0 0 0 0 0
2 3 0 0 0 0 0 0 0 0 0 0 0
0 0 0 0 0 0 0 0 0 0 0 0 0
0 0 0 0 0 0 0 0 0 0 0 0 0
0 0 0 0 0 0 0 0 0 0 0 0 0
0 0 0 0 0 0 0 0
"Viruses;Uroviricota;Caudoviricetes;Caudoviricetes__na;Caudoviricetes__na;Caudoviricetes__na;Siphoviridae_sp._ctx254" 0 0 0 0 0 0 0 0 0 0 0 0
0 0 0 0 0 0 0 1 0 0 0 0 10
10 0 0 0 0 1 0 0 0 0 0 5 4
0 0 0 0 0 0 0 0 0 0 0 0 0
0 0 0 2 2 0 0 0 0 2 1 0 0
0 0 0 0 0 0 0 0
"Viruses;Uroviricota;Caudoviricetes;Caudoviricetes__na;Caudoviricetes__na;Caudoviricetes__na;Siphoviridae_sp._ctX2e5" 0 0 0 0 0 0 0 0 0 0 0 0
0 0 0 0 0 0 0 0 0 0 0 0 0
0 0 0 0 0 0 0 0 0 0 0 0 0
0 0 0 0 0 0 0 0 0 0 0 0 0
0 0 0 0 0 0 0 0 0 0 0 0 0
0 0 0 0 0 1 0 0
"Viruses;Uroviricota;Caudoviricetes;Caudoviricetes__na;Caudoviricetes__na;Caudoviricetes__na;Siphoviridae_sp._ctx7r16" 0 0 0 0 0 0 0 0 0 0 0 0
0 0 0 0 0 0 0 0 0 0 0 0 0
0 0 0 0 0 0 4 0 0 0 0 0 0
0 0 0 0 0 0 0 0 0 0 0 0 0

0 0 0 0 0 0 0 0 0 0 0 0 0
0 0 0 3 0 0 0 0
"Viruses;Uroviricota;Caudoviricetes;Caudoviricetes__na;Caudoviricetes__na;Caudoviricetes__na;Siphoviridae_sp._ctX8T1" 0 0 0 0 0 0 0 0 0 0 0 0
0 0 0 0 0 0 0 0 0 0 0 0 0
0 0 0 0 0 3 0 0 0 0 0 5 2
0 0 0 0 0 0 0 0 0 0 0 0 0
0 0 0 0 0 0 0 0 0 0 0 0 0
0 0 0 0 0 0 0 0
"Viruses;Uroviricota;Caudoviricetes;Caudoviricetes__na;Caudoviricetes__na;Caudoviricetes__na;Siphoviridae_sp._ctXbO14" 0 0 0 0 0 0 0 0 0 0 0 0
2 0 0 0 0 0 4 8 0 1 0 0 0
0 0 0 0 0 0 0 0 0 0 0 0 0
0 0 0 0 0 0 0 0 0 0 1 0 0
0 0 0 0 0 0 0 0 0 0 0 0 0
0 0 0 0 0 0 0 0
"Viruses;Uroviricota;Caudoviricetes;Caudoviricetes__na;Caudoviricetes__na;Caudoviricetes__na;Siphoviridae_sp._ctxc31" 0 0 0 0 0 13 0 0 0 0 0 0
0 0 0 0 0 0 0 0 0 0 0 0 0
0 0 0 0 0 0 0 0 0 0 0 0 0
0 0 0 0 0 0 0 0 0 0 0 0 0
0 0 3 0 0 0 0 0 0 39 3 6 3
0 0 137 101 0 9 0 0
"Viruses;Uroviricota;Caudoviricetes;Caudoviricetes__na;Caudoviricetes__na;Caudoviricetes__na;Siphoviridae_sp._ctxdc10" 0 0 1 0 0 0 0 0 0 0 0 0
0 0 0 0 0 0 0 0 0 0 0 0 0
0 0 0 0 0 0 0 0 0 0 0 0 0
0 0 0 0 0 0 0 0 0 0 0 0 0
0 0 1 0 0 0 0 0 0 0 0 0 0
0 0 2 4 0 1 0 0
"Viruses;Uroviricota;Caudoviricetes;Caudoviricetes__na;Caudoviricetes__na;Caudoviricetes__na;Siphoviridae_sp._ctxfQ4" 0 0 0 0 0 0 0 0 0 0 0 0
1 0 0 0 0 0 0 1 0 0 0 0 0
0 0 0 0 0 0 0 0 0 0 0 0 0
0 0 0 0 0 0 0 0 0 0 0 0 0
0 0 0 0 0 0 0 0 0 0 0 0 0
0 0 0 0 0 0 0 0
"Viruses;Uroviricota;Caudoviricetes;Caudoviricetes__na;Caudoviricetes__na;Caudoviricetes__na;Siphoviridae_sp._ctxjx4" 0 0 0 0 0 0 0 0 0 0 0 0
0 0 0 0 0 0 0 0 0 0 0 0 0
0 0 0 0 0 0 0 0 0 0 0 0 0
0 0 0 0 0 0 0 0 0 0 0 0 0
0 0 0 10 22 0 0 0 0 0 0 0 0
0 0 0 0 0 0 0 0
"Viruses;Uroviricota;Caudoviricetes;Caudoviricetes__na;Caudoviricetes__na;Caudoviricetes__na;Siphoviridae_sp._ctxMM9" 0 0 0 0 0 0 0 0 0 0 0 0
0 0 0 0 0 0 0 0 0 0 0 0 0
0 0 0 0 0 0 0 0 0 0 0 0 0
0 0 0 0 0 0 0 0 0 0 0 0 0

0 0 0 0 1 0 0 0 0 0 0 0 0
0 0 0 0 0 0 0 0
"Viruses;Uroviricota;Caudoviricetes;Caudoviricetes__na;Caudoviricetes__na;Caudoviricetes__na;Siphoviridae_s
p._ctXPh6" 0 0 0 0 0 0 0 0 0 0 0 0
0 0 0 0 0 0 0 0 0 0 0 0 4
0 2 0 0 0 0 0 0 0 0 0 0 0
0 0 0 0 0 0 0 0 0 0 0 0 0
0 0 0 0 0 0 0 0 0 0 0 0 0
0 0 0 0 0 0 0 0
"Viruses;Uroviricota;Caudoviricetes;Caudoviricetes__na;Caudoviricetes__na;Caudoviricetes__na;Siphoviridae_s
p._ctXQ92" 0 0 0 0 0 0 0 0 0 0 0 0
0 0 0 0 0 0 0 0 0 0 0 0 1
5 0 0 0 0 2 1 0 0 0 0 1 3
0 0 0 0 0 0 0 0 0 0 0 0 0
0 0 0 0 3 0 0 0 0 0 2 0 0
0 0 0 4 0 0 0 0
"Viruses;Uroviricota;Caudoviricetes;Caudoviricetes__na;Caudoviricetes__na;Caudoviricetes__na;Siphoviridae_s
p._ctXQq5" 0 0 0 0 0 0 0 0 0 0 0 0
0 0 0 0 0 0 0 0 0 0 0 0 1
0 0 0 0 0 0 0 0 0 0 0 0 0
0 0 0 0 0 0 0 0 0 0 0 0 0
0 0 0 0 0 0 0 0 0 0 0 0 0
0 0 0 0 0 0 0 0
"Viruses;Uroviricota;Caudoviricetes;Caudoviricetes__na;Caudoviricetes__na;Caudoviricetes__na;Siphoviridae_s
p._ctxS04" 0 0 0 0 0 0 0 0 0 0 0 0
0 0 0 0 0 0 2 2 0 0 0 0 0
0 0 0 0 0 0 0 0 0 0 0 0 0
0 0 0 0 1 3 0 0 0 0 0 1 0
0 0 0 0 0 0 0 0 0 0 0 0 0
0 0 0 0 0 0 0 0
"Viruses;Uroviricota;Caudoviricetes;Caudoviricetes__na;Caudoviricetes__na;Caudoviricetes__na;Siphoviridae_s
p._ctxvK3" 0 0 0 0 0 0 0 0 0 0 0 0
0 0 0 0 0 0 0 9 0 0 1 0 0
0 0 0 0 0 3 3 0 0 0 0 1 0
0 0 0 0 0 0 0 0 0 0 0 0 0
0 0 0 11 15 0 0 0 0 0 0 0 0
0 0 0 0 0 0 5 0
"Viruses;Uroviricota;Caudoviricetes;Caudoviricetes__na;Caudoviricetes__na;Caudoviricetes__na;Siphoviridae_s
p._ctxyw6" 0 0 0 0 0 0 0 0 0 0 0 0
0 0 0 0 0 0 0 0 0 0 0 0 0
0 0 0 0 0 0 0 0 0 0 0 0 0
0 0 0 4 0 0 0 0 0 0 0 0 0
0 0 0 0 0 0 0 0 0 0 0 0 0
0 0 0 0 0 0 0 0
"Viruses;Uroviricota;Caudoviricetes;Caudoviricetes__na;Caudoviricetes__na;Caudoviricetes__na;Siphoviridae_s
p._ctXzK3" 0 0 0 0 0 0 0 0 0 0 0 0
0 0 0 0 0 0 0 0 0 0 0 0 0
0 0 0 0 0 0 0 0 0 0 0 0 0
0 0 0 0 0 0 0 0 0 0 0 0 1

0 0 0 0 0 0 0 0 0 0 0 0 0
0 0 0 0 0 0 0 0
"Viruses;Uroviricota;Caudoviricetes;Caudoviricetes__na;Caudoviricetes__na;Caudoviricetes__na;Siphoviridae_s
p._ctXZQ9" 0 0 0 0 0 0 0 0 0 0 0 0
0 0 0 0 0 0 0 0 0 0 0 0 0
0 0 0 0 0 0 0 0 0 0 0 0 0
0 0 0 2 0 0 0 0 0 0 0 0 0
0 1 0 0 0 0 0 0 0 0 0 0 0
0 0 0 0 0 0 0 0
"Viruses;Uroviricota;Caudoviricetes;Caudoviricetes__na;Caudoviricetes__na;Caudoviricetes__na;Siphoviridae_s
p._ctXZx16" 0 0 0 0 0 0 0 0 0 0 0 0
0 0 0 0 0 0 0 0 0 0 0 0 0
0 0 0 0 0 1 0 0 0 0 0 0 0
0 0 0 0 1 0 1 0 0 0 0 0 0
0 0 0 0 0 0 0 0 0 0 0 0 0
0 0 1 0 0 0 0 0
"Viruses;Uroviricota;Caudoviricetes;Caudoviricetes__na;Caudoviricetes__na;Caudoviricetes__na;Siphoviridae_s
p._cty4Z2" 0 0 0 0 0 0 0 0 0 0 0 0
0 0 0 0 0 0 0 0 0 0 0 0 1
0 0 0 0 0 0 0 0 0 0 0 0 0
0 0 0 0 0 0 0 0 0 0 0 0 0
0 0 0 0 0 0 0 0 0 0 0 0 0
0 0 0 0 0 0 0 0
"Viruses;Uroviricota;Caudoviricetes;Caudoviricetes__na;Caudoviricetes__na;Caudoviricetes__na;Siphoviridae_s
p._ctYBm1" 0 0 0 0 0 0 0 0 0 0 0 0
0 0 0 0 0 0 0 0 0 0 0 0 0
0 0 0 0 0 0 0 0 0 0 1 0 0
0 0 0 0 0 0 0 0 0 0 0 0 0
0 0 0 0 0 0 0 0 0 0 0 0 0
0 0 0 0 0 0 0 0
"Viruses;Uroviricota;Caudoviricetes;Caudoviricetes__na;Caudoviricetes__na;Caudoviricetes__na;Siphoviridae_s
p._ctYcY12" 0 0 0 0 0 0 0 0 0 0 0 0
0 0 0 0 0 0 0 0 0 0 0 0 2
2 0 0 0 0 0 0 0 0 0 0 0 0
0 0 0 0 0 0 0 0 0 0 0 0 0
0 0 0 0 0 0 0 0 0 0 0 0 0
0 0 0 0 0 0 0 0
"Viruses;Uroviricota;Caudoviricetes;Caudoviricetes__na;Caudoviricetes__na;Caudoviricetes__na;Siphoviridae_s
p._ctyHC15" 0 0 0 0 0 0 0 0 0 0 0 0
1 0 0 0 0 0 0 0 0 0 0 0 2
4 0 0 0 0 0 2 0 0 0 0 2 2
0 0 0 0 0 0 0 0 0 0 0 1 0
0 0 0 0 1 0 0 0 0 0 0 1 0
0 0 3 3 0 0 0 0
"Viruses;Uroviricota;Caudoviricetes;Caudoviricetes__na;Caudoviricetes__na;Caudoviricetes__na;Siphoviridae_s
p._ctyjS2" 0 0 0 0 0 0 0 0 0 0 0 0
0 0 0 0 0 0 0 0 0 0 0 0 0
0 0 0 0 0 0 0 0 0 0 0 0 0
0 0 0 0 0 0 0 0 0 0 0 0 0

0 0 0 0 0 0 0 0 0 2 0 0 0
0 0 0 0 0 0 0 0
"Viruses;Uroviricota;Caudoviricetes;Caudoviricetes__na;Caudoviricetes__na;Caudoviricetes__na;Siphoviridae_s
p._ctYKh4" 0 0 0 0 0 0 0 0 0 0 0 0
2 2 0 0 0 0 0 0 0 0 1 0 0
0 0 0 0 0 2 3 0 0 0 0 5 3
0 0 0 2 0 0 0 0 0 0 0 0 0
0 1 0 0 0 0 0 0 0 0 0 0 0
0 0 0 0 0 0 0 0
"Viruses;Uroviricota;Caudoviricetes;Caudoviricetes__na;Caudoviricetes__na;Caudoviricetes__na;Siphoviridae_s
p._ctylc9" 0 0 0 0 0 0 0 0 0 0 0 0
0 0 0 0 0 0 0 0 0 0 0 0 0
0 0 0 0 0 0 0 0 0 2 0 0 0
0 0 0 0 0 0 0 0 0 0 0 0 0
0 0 0 2 3 0 0 0 0 0 0 0 0
0 0 0 0 0 0 0 0
"Viruses;Uroviricota;Caudoviricetes;Caudoviricetes__na;Caudoviricetes__na;Caudoviricetes__na;Siphoviridae_s
p._ctyNQ5" 0 0 0 0 0 0 0 0 0 0 0 0
0 0 0 0 0 0 0 0 0 0 0 0 0
0 0 0 0 0 0 0 0 0 0 0 0 1
0 0 0 0 0 0 0 0 0 0 0 0 0
0 0 0 0 0 0 0 0 0 0 0 0 0
0 0 0 0 0 0 0 0
"Viruses;Uroviricota;Caudoviricetes;Caudoviricetes__na;Caudoviricetes__na;Caudoviricetes__na;Siphoviridae_s
p._ctyvQ1" 0 0 0 1 0 0 0 0 0 0 0 0
0 0 0 0 0 0 0 0 0 0 0 0 0
0 0 0 0 0 0 0 0 0 0 0 0 0
0 0 0 0 0 0 0 0 0 0 0 0 0
0 0 0 1 0 0 0 0 0 0 0 0 0
0 0 0 0 0 0 0 0
"Viruses;Uroviricota;Caudoviricetes;Caudoviricetes__na;Caudoviricetes__na;Caudoviricetes__na;Siphoviridae_s
p._ctZ0X1" 0 0 0 0 0 0 0 0 0 0 0 0
0 0 0 0 0 0 1 1 0 0 0 0 0
0 0 0 0 0 0 0 0 0 0 0 0 1
0 0 0 0 0 0 0 0 0 0 0 4 0
0 0 0 1 0 0 0 0 0 2 0 0 0
0 0 0 0 0 0 0 0
"Viruses;Uroviricota;Caudoviricetes;Caudoviricetes__na;Caudoviricetes__na;Caudoviricetes__na;Siphoviridae_s
p._ctZCK1" 0 0 0 0 0 0 0 0 0 0 0 0
0 0 0 0 0 4 0 0 0 0 0 0 0
0 0 0 0 0 0 0 1 0 166 0 0 0
0 0 0 32 0 0 0 0 0 0 0 0 0
0 0 0 0 0 0 0 0 0 6 2 0 1
0 1 0 0 0 0 0 0
"Viruses;Uroviricota;Caudoviricetes;Caudoviricetes__na;Caudoviricetes__na;Caudoviricetes__na;Siphoviridae_s
p._ctZD11" 0 0 0 0 0 0 0 0 0 0 0 0
0 2 0 0 3 9 0 0 0 0 0 0 0
0 0 0 0 0 0 2 0 0 0 0 0 2
0 1 0 0 0 0 0 0 0 0 0 0 0

0 0 0 0 1 0 0 0 0 0 0 0 0
0 0 0 0 0 0 0 0
"Viruses;Uroviricota;Caudoviricetes;Caudoviricetes__na;Caudoviricetes__na;Caudoviricetes__na;Siphoviridae_s
p._ctZE52" 0 0 0 0 0 0 0 0 0 0 0 0
0 0 0 0 1 0 0 0 0 0 0 0 0
0 0 0 0 0 0 0 0 0 0 0 0 0
0 0 0 0 0 0 0 0 0 0 0 0 0
0 0 0 0 0 0 0 0 0 0 0 0 0
0 0 0 0 0 0 0 0
"Viruses;Uroviricota;Caudoviricetes;Caudoviricetes__na;Caudoviricetes__na;Caudoviricetes__na;Siphoviridae_s
p._ctzEj35" 0 0 0 0 0 0 0 0 0 0 0 0
0 0 0 0 0 0 23 27 0 1 0 0 2
1 0 0 0 0 0 0 0 0 0 0 0 0
0 0 0 0 5 3 0 0 0 0 0 0 0
0 0 0 0 0 0 0 0 0 0 0 0 0
0 0 0 0 0 0 0 0
"Viruses;Uroviricota;Caudoviricetes;Caudoviricetes__na;Caudoviricetes__na;Caudoviricetes__na;Siphoviridae_s
p._ctZgu8" 0 0 0 0 0 0 0 0 0 0 0 0
0 0 0 0 0 0 0 0 0 0 0 0 0
1 0 0 0 0 0 0 0 0 0 0 0 0
0 0 0 0 0 0 0 0 0 0 0 0 0
0 0 0 0 0 0 0 0 0 0 0 0 0
0 0 0 0 0 0 0 0
"Viruses;Uroviricota;Caudoviricetes;Caudoviricetes__na;Caudoviricetes__na;Caudoviricetes__na;Siphoviridae_s
p._ctZHD14" 0 0 0 0 0 0 0 0 0 0 0 0
0 0 0 0 0 0 0 0 0 0 0 0 0
0 0 0 0 0 0 0 0 0 0 0 0 0
0 0 0 0 0 0 0 0 0 0 0 0 0
0 0 0 0 0 0 0 0 0 0 0 0 0
0 0 0 1 0 0 0 0
"Viruses;Uroviricota;Caudoviricetes;Caudoviricetes__na;Caudoviricetes__na;Caudoviricetes__na;Siphoviridae_s
p._ctzm5103" 0 0 0 0 0 0 0 0 0 0 0 0
0 0 0 0 0 0 0 0 0 0 0 0 0
0 0 0 0 0 0 0 0 0 0 0 1 3
0 0 0 0 0 0 0 0 0 0 0 0 0
0 0 0 0 0 0 0 0 0 0 0 0 0
0 0 0 0 0 0 0 0
"Viruses;Uroviricota;Caudoviricetes;Caudoviricetes__na;Caudoviricetes__na;Caudoviricetes__na;Siphoviridae_s
p._ctzMZ8" 0 0 0 0 0 0 0 0 0 0 0 0
0 0 0 0 0 0 0 0 0 0 0 0 3
6 0 0 0 0 0 0 0 0 0 0 0 3
0 0 0 0 0 0 0 0 0 0 0 0 0
0 0 0 2 2 0 0 0 0 0 0 0 0
0 0 0 0 0 0 0 0
"Viruses;Uroviricota;Caudoviricetes;Caudoviricetes__na;Caudoviricetes__na;Caudoviricetes__na;Siphoviridae_s
p._ctZpP9" 0 0 0 0 0 0 0 0 0 0 0 0
0 0 0 0 0 0 0 0 0 0 0 0 0
0 0 0 0 0 0 0 0 0 0 0 0 1
0 0 0 0 0 0 0 0 0 0 0 5 0

0 0 0 0 0 0 0 0 0 0 0 0 0
0 0 0 0 0 0 0 0
"Viruses;Uroviricota;Caudoviricetes;Caudoviricetes__na;Caudoviricetes__na;Caudoviricetes__na;Siphoviridae_s
p._ctzr51" 0 0 0 0 0 0 0 0 0 0 0 0
0 0 0 0 0 0 0 0 0 0 0 0 0
0 0 0 0 0 0 0 0 0 0 0 0 0
0 0 0 0 0 0 0 0 0 0 0 0 0
0 0 0 50 85 1 0 0 0 0 0 0 0
0 0 0 0 0 1 0 0
"Viruses;Uroviricota;Caudoviricetes;Caudoviricetes__na;Caudoviricetes__na;Caudoviricetes__na;Siphoviridae_s
p._ctzrC10" 0 0 0 0 0 0 0 0 0 0 0 0
0 1 1 0 0 0 1 1 0 0 0 0 0
0 0 0 0 0 0 1 0 0 0 0 1 0
1 0 0 0 0 0 0 0 0 0 0 0 0
0 0 0 3 5 0 0 0 0 0 0 0 0
0 0 1 2 0 0 0 0
"Viruses;Uroviricota;Caudoviricetes;Caudoviricetes__na;Caudoviricetes__na;Caudoviricetes__na;Siphoviridae_s
p._ctzrk10" 0 0 0 0 0 0 0 0 0 0 0 0
0 0 0 0 0 0 0 0 0 0 0 0 0
0 0 0 0 0 0 0 0 0 0 0 0 0
0 0 0 0 0 0 0 0 0 1 0 0 0
0 0 0 0 0 0 0 0 0 0 0 0 0
0 0 0 0 0 0 0 0
"Viruses;Uroviricota;Caudoviricetes;Caudoviricetes__na;Caudoviricetes__na;Caudoviricetes__na;Siphoviridae_s
p._ctzVd36" 0 0 0 0 0 0 0 0 0 0 0 0
0 0 0 0 0 0 0 0 0 0 0 0 0
0 0 0 0 0 1 1 0 0 0 0 0 0
0 0 0 0 0 0 0 0 0 0 0 0 0
0 0 0 0 1 0 0 0 0 0 0 0 0
0 0 0 0 0 0 0 0
"Viruses;Uroviricota;Caudoviricetes;Caudoviricetes__na;Caudoviricetes__na;Caudoviricetes__na;Siphoviridae_s
p._ctZYQ8" 0 0 0 0 2 0 0 0 0 0 0 0
0 0 0 0 0 0 0 0 0 0 0 0 0
0 0 0 0 0 0 0 0 0 0 0 0 0
0 0 0 0 0 0 0 0 0 0 0 0 0
0 0 0 0 0 0 0 0 0 0 0 0 0
0 0 0 0 0 0 0 0
"Viruses;Uroviricota;Caudoviricetes;Caudoviricetes__na;Caudoviricetes__na;Caudoviricetes__na;Siphoviridae_s
p._ctZZK17" 0 0 0 0 0 0 0 0 0 0 0 0
7 4 0 0 1 0 0 0 0 0 0 0 0
0 0 0 0 0 0 0 0 0 0 0 0 1
0 0 0 0 0 0 0 0 0 0 0 0 0
0 0 0 3 1 0 0 0 0 0 0 0 0
0 0 0 2 0 0 0 0
"Viruses;Uroviricota;Caudoviricetes;Caudoviricetes__na;Caudoviricetes__na;Caudoviricetes__na;Siphovirus_L
N-2020-2" 0 0 0 0 0 0 0 0 0 0 0 0
0 0 0 0 0 0 0 0 0 0 0 0 0
0 0 0 0 0 0 0 0 0 0 0 0 0
0 0 0 0 0 0 0 0 0 0 1 0 2

0 0 0 0 0 0 0 0 0 0 0 0 0
0 0 0 0 0 0 0 0
"Viruses;Uroviricota;Caudoviricetes;Caudoviricetes__na;Caudoviricetes__na;Caudoviricetes__na;Staphylococcus_phage_IME1354_01" 0 0 0 1 0 0 0 0 0 0 0
0 0 0 0 0 0 0 0 0 0 0 0 0
0 0 0 0 0 0 0 0 0 0 0 0 0
0 0 0 0 0 0 0 0 0 0 0 0 0
0 0 0 0 0 0 0 0 0 0 0 0 0
0 0 0 0 0 0 0 0 0
"Viruses;Uroviricota;Caudoviricetes;Caudoviricetes__na;Caudoviricetes__na;Caudoviricetes__na;Staphylococcus_phage_vB_StaM_SA1"0 0 0 0 0 0 0 0 0 0 0
0 0 0 0 0 0 0 0 0 0 0 0 0
0 0 0 0 0 0 0 0 0 0 0 0 0
0 0 0 0 0 0 0 0 0 0 0 0 0
0 0 1 0 0 0 0 0 0 0 0 0 0
0 0 0 0 0 0 0 0 0
"Viruses;Uroviricota;Caudoviricetes;Caudoviricetes__na;Caudoviricetes__na;Caudoviricetes__na;Stenotrophomonas_phage_BUCT555" 0 0 0 0 0 3 0 0 0 0 0
0 0 0 0 0 0 0 0 0 0 0 0 0
0 0 0 0 0 0 0 0 0 0 0 0 0
0 0 0 0 0 0 0 0 0 0 0 0 0
0 0 0 0 0 0 0 0 0 0 0 0 0
0 0 0 0 0 0 0 0 0
"Viruses;Uroviricota;Caudoviricetes;Caudoviricetes__na;Caudoviricetes__na;Caudoviricetes__na;Stenotrophomonas_phage_CUB19" 0 0 0 0 7 0 0 0 0 0 0
0 0 0 0 0 0 0 0 0 0 0 0 0
0 0 0 0 0 0 0 0 0 0 0 0 0
0 0 0 0 0 0 0 0 0 0 0 0 0
0 0 0 0 0 0 0 0 0 0 0 0 0
0 0 0 0 0 0 0 0 0
"Viruses;Uroviricota;Caudoviricetes;Caudoviricetes__na;Caudoviricetes__na;Caudoviricetes__na;Stenotrophomonas_phage_Silvanus" 0 0 1 0 0 0 0 0 0 0 0
0 0 0 0 0 0 0 0 0 0 0 0 0
0 0 0 0 0 0 0 0 0 0 0 0 0
0 0 0 0 0 0 0 0 0 0 0 0 0
0 0 0 0 0 0 0 0 0 0 0 0 0
0 0 0 0 0 0 0 0 0
"Viruses;Uroviricota;Caudoviricetes;Caudoviricetes__na;Caudoviricetes__na;Caudoviricetes__na;Stenotrophomonas_phage_vB_SM_ytsc_ply2008005c" 0 0 0 0 0 1 0 0 0
1 0 0 0 0 0 0 0 0 0 0 0 0
0 0 0 0 0 0 0 0 0 0 0 0 0
0 0 0 0 0 0 0 0 0 0 0 5 0
0 0 0 0 0 0 0 0 3 1 0 0 0
0 0 0 0 0 0 0 0 0 0 0
"Viruses;Uroviricota;Caudoviricetes;Caudoviricetes__na;Caudoviricetes__na;Caudoviricetes__na;Stenotrophomonas_phage_vB_SmaS-AXL_3" 0 0 0 0 7 0 0 0 0 0
0 0 0 0 0 0 0 0 0 0 0 0 0
0 0 0 0 0 0 0 0 0 0 0 0 0
0 0 0 0 0 0 0 0 0 0 0 0 0

0 0 0 0 0 0 0 0 0 0 0 0 0
0 0 0 0 0 0 0 0 0 0
"Viruses;Uroviricota;Caudoviricetes;Caudoviricetes__na;Caudoviricetes__na;Caudoviricetes__na;Stenotrophom
onas_phage_vB_SmaS-DLP_6" 0 0 0 0 1 0 0 0 0 0
0 0 0 0 0 0 0 0 0 0 0 0 0
0 0 0 0 0 0 0 0 0 0 0 0 0
0 0 0 0 0 0 0 0 0 0 0 1 0
0 0 0 0 0 0 0 0 0 0 0 0 0
0 0 0 0 0 0 0 0 0 0
"Viruses;Uroviricota;Caudoviricetes;Caudoviricetes__na;Caudoviricetes__na;Caudoviricetes__na;Streptococcus_
phage_CHPC1198" 0 0 0 0 0 0 0 0 0 0 0
0 0 0 0 0 0 0 0 0 0 0 0 0
0 0 0 0 0 0 0 0 0 0 0 0 0
0 0 0 0 0 0 0 0 0 0 0 0 0
0 0 0 0 1 0 0 0 0 0 0 0 0
0 0 0 0 0 0 0 0 0
"Viruses;Uroviricota;Caudoviricetes;Caudoviricetes__na;Caudoviricetes__na;Caudoviricetes__na;Streptococcus_
phage_CHPC926" 0 0 1 0 0 0 0 0 0 0 0
0 0 0 0 0 0 0 0 0 0 0 0 0
0 0 0 0 0 0 0 0 0 0 0 0 0
0 0 0 0 0 0 0 0 0 0 0 0 0
0 0 0 0 0 0 0 0 0 0 0 0 0
0 0 0 0 0 0 0 0 0
"Viruses;Uroviricota;Caudoviricetes;Caudoviricetes__na;Caudoviricetes__na;Caudoviricetes__na;Streptococcus_
phage_Javan191" 0 0 0 0 0 0 0 0 0 0 0
0 0 0 0 0 0 0 0 0 0 0 0 0
0 0 0 0 0 0 0 0 0 0 0 0 0
1 0 0 0 0 0 0 0 0 0 0 0 0
0 0 0 0 0 0 0 0 0 0 1 0 0
0 0 0 0 0 0 0 0 0
"Viruses;Uroviricota;Caudoviricetes;Caudoviricetes__na;Caudoviricetes__na;Caudoviricetes__na;Streptococcus_
phage_SpSL1" 0 0 0 0 0 0 0 0 0 0 0 0
0 0 0 0 0 0 0 0 0 0 0 0 0
0 0 0 0 0 0 0 0 0 0 2 0 0
0 0 0 0 0 0 0 0 0 0 0 0 0
0 0 0 0 0 0 0 0 0 0 0 0 0
0 0 0 0 0 0 0 0
"Viruses;Uroviricota;Caudoviricetes;Caudoviricetes__na;Caudoviricetes__na;Caudoviricetes__na;Streptomyces_
phage_LibertyBell" 0 0 0 0 0 0 0 0 0 0 0
0 0 0 0 0 0 0 0 0 0 0 0 0
0 0 0 0 0 0 0 0 0 0 0 0 0
0 0 0 0 0 0 0 0 0 0 0 0 0
0 0 0 0 0 0 0 0 0 0 0 0 0
0 0 0 0 0 0 0 2 0
"Viruses;Uroviricota;Caudoviricetes;Caudoviricetes__na;Caudoviricetes__na;Caudoviricetes__na;Sulfitobacter_
phage_phiGT1" 0 0 1 1 0 0 0 0 0 0 0 0
0 0 0 0 0 0 0 0 0 0 0 0 0
0 0 0 0 0 0 0 0 0 0 0 0 0
0 0 0 0 0 0 0 0 0 1 0 0 0

0 0 0 0 0 0 0 0 0 0 0 0 0
0 0 0 0 0 1 0 0
"Viruses;Uroviricota;Caudoviricetes;Caudoviricetes__na;Caudoviricetes__na;Caudoviricetes__na;Synechococcus_phage_BUCT-ZZ01" 0 0 0 1 0 3 0 0 0 0 0
0 0 0 0 0 0 0 0 0 0 0 0 0
0 0 0 0 0 0 0 0 0 0 0 0 0
0 0 0 0 0 0 0 0 0 0 0 0 0
0 0 0 0 0 0 0 0 0 0 0 0 0
0 0 0 0 0 0 0 0 0
"Viruses;Uroviricota;Caudoviricetes;Caudoviricetes__na;Caudoviricetes__na;Caudoviricetes__na;Synechococcus_phage_MRHenn-2013a" 0 0 0 0 1 0 0 0 0 0
0 0 0 0 0 0 0 0 0 0 0 0 0
0 0 0 0 0 0 0 0 0 0 0 0 0
0 0 0 0 0 0 0 0 0 0 0 0 0
0 0 0 0 0 0 0 0 0 0 0 0 0
0 0 0 0 0 0 0 0 0 0
"Viruses;Uroviricota;Caudoviricetes;Caudoviricetes__na;Caudoviricetes__na;Caudoviricetes__na;Synechococcus_phage_S-B68" 0 0 0 0 0 0 0 0 0 0 0 0
0 0 0 0 0 0 0 0 0 0 0 0 0
0 0 0 0 0 0 0 0 0 0 0 0 0
0 0 0 0 0 0 0 0 0 1 0 0 0
0 0 0 0 0 0 0 0 0 0 0 0 0
0 0 0 0 0 0 0 0
"Viruses;Uroviricota;Caudoviricetes;Caudoviricetes__na;Caudoviricetes__na;Caudoviricetes__na;Synechococcus_phage_S-CBS2" 0 0 1 0 2 0 0 0 0 0 0
0 0 0 0 0 0 0 0 0 0 0 0 0
0 0 0 0 0 0 0 0 0 0 0 0 0
0 0 0 0 0 0 0 0 0 0 0 0 0
0 0 0 0 0 0 0 0 0 0 0 0 0
0 0 0 0 0 0 0 0 0
"Viruses;Uroviricota;Caudoviricetes;Caudoviricetes__na;Caudoviricetes__na;Caudoviricetes__na;Synechococcus_phage_S-CBS3" 0 0 0 1 0 0 0 0 0 0 0
0 0 0 0 0 0 0 0 0 0 0 0 0
0 0 0 0 0 0 0 0 0 0 0 0 0
0 0 0 0 0 0 0 0 0 0 0 0 0
0 0 0 0 0 0 0 0 0 0 0 0 0
0 0 0 0 0 0 0 0 0
"Viruses;Uroviricota;Caudoviricetes;Caudoviricetes__na;Caudoviricetes__na;Caudoviricetes__na;Synechococcus_phage_S-CRM01" 0 0 0 0 0 0 0 0 0 0 0
0 0 0 0 0 0 0 0 0 0 0 0 0
0 0 0 0 0 0 0 0 0 0 0 0 0
0 0 0 0 8 0 0 0 0 0 0 0 0
0 0 0 0 0 0 0 0 0 0 0 0 0
0 0 0 0 0 0 0 0 0
"Viruses;Uroviricota;Caudoviricetes;Caudoviricetes__na;Caudoviricetes__na;Caudoviricetes__na;Synechococcus_phage_S-H1" 1 0 1 1 0 1 0 0 0 0 0 0
0 0 0 0 0 0 0 0 0 0 0 0 0
0 0 0 0 0 0 0 0 0 0 0 0 0
0 0 0 0 0 0 0 0 0 0 0 0 0

0 0 0 0 0 1 0 0 0 0 0 0 0
0 0 0 0 0 0 0 0
"Viruses;Uroviricota;Caudoviricetes;Caudoviricetes__na;Caudoviricetes__na;Caudoviricetes__na;Synechococcus_phage_S-LBS1"0 0 3 1 0 1 0 0 0 0 0 0
0 0 0 0 0 0 0 0 0 0 0 0 0
0 0 0 0 0 0 0 0 0 0 0 0 0
0 0 0 0 0 0 0 0 0 0 0 0 0
0 0 0 0 0 0 0 0 0 0 0 0 0
0 0 0 0 0 0 0 0
"Viruses;Uroviricota;Caudoviricetes;Caudoviricetes__na;Caudoviricetes__na;Caudoviricetes__na;Synechococcus_phage_Yong-M3-232" 0 0 0 0 0 0 0 0 0 0 0
0 0 0 0 0 0 3 0 0 0 0 0 0
0 0 0 0 0 0 0 0 0 0 0 0 0
0 0 0 0 0 0 0 0 0 0 0 0 0
0 0 0 0 0 0 0 0 0 0 0 0 0
0 0 0 0 0 0 0 0 0
"Viruses;Uroviricota;Caudoviricetes;Caudoviricetes__na;Caudoviricetes__na;Caudoviricetes__na;Tenacibaculum_phage_JQ" 0 0 0 0 7 27 0 0 0 0 0 0
0 0 0 0 0 0 0 0 0 0 0 0 0
0 0 0 0 0 0 0 0 0 0 0 0 0
1 0 0 0 0 0 0 0 0 0 0 0 0
0 0 0 0 0 0 0 0 0 0 0 0 0
0 0 0 0 0 0 0 0
"Viruses;Uroviricota;Caudoviricetes;Caudoviricetes__na;Caudoviricetes__na;Caudoviricetes__na;Tsukamurella_phage_TPA4" 0 0 0 0 0 1 0 0 0 0 0 0
0 0 0 0 0 0 0 0 0 0 0 0 0
0 0 0 0 0 0 0 0 0 0 0 0 0
0 0 0 0 0 0 0 0 0 0 0 0 0
0 0 0 0 0 0 0 0 0 0 0 0 0
0 0 0 0 0 0 0 0
"Viruses;Uroviricota;Caudoviricetes;Caudoviricetes__na;Caudoviricetes__na;Caudoviricetes__na;uncultured_Caudovirales_phage" 111 82 634 1651 8455 4850 0 0 1 21 0
0 1 2 10 9 70 121 1 0 9 3 38 95
1 0 3 2 0 6 0 0 5 1 195 70 4
0 3 5 13 1637 0 2 49 12 118 1286 0 0
8 10 14 37 2 7 9 2 0 240 6 0 1
3 0 3 6 1 1 0 190 0
"Viruses;Uroviricota;Caudoviricetes;Caudoviricetes__na;Caudoviricetes__na;Caudoviricetes__na;uncultured_cyanomyovirus" 0 3 0 0 3 2 0 0 0 0 0 0
0 0 0 0 0 0 0 0 0 0 0 0 0
0 0 0 0 0 0 0 0 0 0 0 0 0
0 0 0 0 0 0 0 0 0 0 0 0 0
0 0 0 0 0 0 0 0 0 0 0 0 0
0 0 0 0 0 0 0 0
"Viruses;Uroviricota;Caudoviricetes;Caudoviricetes__na;Caudoviricetes__na;Caudoviricetes__na;uncultured_Mediterranean_phage_uvDeep-CGR0-AD1-C123" 0 0 0 1 0 0 0 0
0 0 0 0 0 0 0 0 0 0 0 0 0
0 0 0 0 0 0 0 0 0 0 0 0 0
0 0 0 0 0 0 0 0 0 0 0 0 0

0 0 0 0 0 0 0 0 0 0 0 0 0
0 0 0 0 0 0 0 0 0 0 0 0
"Viruses;Uroviricota;Caudoviricetes;Caudoviricetes__na;Caudoviricetes__na;Caudoviricetes__na;uncultured_Mediterranean_phage_uvDeep-CGR0-AD1-C239" 0 0 0 0 6 0 0 0
0 0 0 0 0 0 0 0 0 0 0 0 0
0 0 0 0 0 0 0 0 0 0 0 0 0
0 0 0 0 0 0 0 0 0 0 0 0 0
0 0 0 0 0 0 0 0 0 0 0 0 0
0 0 0 0 0 0 0 0 0 0 0 0
"Viruses;Uroviricota;Caudoviricetes;Caudoviricetes__na;Caudoviricetes__na;Caudoviricetes__na;uncultured_Mediterranean_phage_uvDeep-CGR1-KM17-C101" 0 0 0 1 2 0 0
0 0 0 0 0 0 0 0 0 0 0 0 0
0 0 0 0 0 0 0 0 0 0 0 0 0
0 0 0 0 0 0 0 0 0 0 0 0 0
0 0 0 0 0 0 0 0 0 0 0 0 0
0 0 0 0 0 0 0 0 0 0 0 2 0
"Viruses;Uroviricota;Caudoviricetes;Caudoviricetes__na;Caudoviricetes__na;Caudoviricetes__na;uncultured_Mediterranean_phage_uvDeep-CGR2-KM21-C345" 0 0 1 0 2 0 0
0 0 0 0 0 0 0 0 0 0 0 0 0
0 0 0 0 0 0 0 0 0 0 0 0 0
0 0 0 0 0 0 0 0 0 0 0 0 0
0 0 0 0 0 0 0 0 0 0 0 0 0
0 0 0 0 0 0 0 0 0 0 0 0 0
"Viruses;Uroviricota;Caudoviricetes;Caudoviricetes__na;Caudoviricetes__na;Caudoviricetes__na;uncultured_Mediterranean_phage_uvMED" 0 0 3 16 75 54 0 0 0 2
0 0 4 3 4 5 6 9 0 0 2 0 1
1 0 0 0 0 0 0 0 0 0 0 0 2
0 0 0 0 0 6 0 0 1 0 0 29 0
0 0 0 0 0 0 0 0 0 0 126 0 0
0 0 0 0 0 0 0 0 1 0
"Viruses;Uroviricota;Caudoviricetes;Caudoviricetes__na;Caudoviricetes__na;Caudoviricetes__na;uncultured_T7-like_podovirus" 0 0 0 0 8 0 0 0 0 0 0
0 0 0 0 0 1 0 0 0 0 0 0 0
0 0 0 0 0 0 0 0 0 0 0 0 0
0 0 0 0 0 0 0 0 0 0 2 0 0
0 0 0 0 0 0 0 0 0 0 0 0 0
0 0 0 0 0 0 0 0 0
"Viruses;Uroviricota;Caudoviricetes;Caudoviricetes__na;Caudoviricetes__na;Caudoviricetes__na;Vibrio_phage_1.033.O._10N.222.49.B8" 0 0 0 1 0 0 0 0 0 0 0
0 0 0 0 0 0 0 0 0 0 0 0 0
0 0 0 0 0 0 0 0 0 0 0 0 0
0 0 0 0 0 0 0 0 0 0 0 0 0
0 0 0 0 0 0 0 0 0 0 0 0 0
0 0 0 0 0 0 0 0 0
"Viruses;Uroviricota;Caudoviricetes;Caudoviricetes__na;Caudoviricetes__na;Caudoviricetes__na;Vibrio_phage_1.052.A._10N.286.46.C3" 0 0 0 0 4 0 0 0 0 0 0
0 0 0 0 0 0 0 0 0 0 0 0 0
0 0 0 0 0 0 0 0 0 0 0 0 0
0 0 0 0 0 0 0 0 0 0 0 0 0

0	0	0	0	0	0	0	0	0	0	0	0	0
0	0	0	0	0	0	0	0	0
"Viruses;Uroviricota;Caudoviricetes;Caudoviricetes__na;Caudoviricetes__na;Caudoviricetes__na;Vibrio_phage_
1.076.O._10N.286.51.B7"	0	0	0	0	0	9	0	0	0	0	0
0	0	0	0	0	0	0	0	0	0	0	0	0
0	0	0	0	0	0	0	0	0	0	0	0	0
0	0	0	0	0	0	0	0	0	0	0	0	0
0	0	0	0	0	0	0	0	0	0	0	0	0
0	0	0	0	0	0	0	0	0
"Viruses;Uroviricota;Caudoviricetes;Caudoviricetes__na;Caudoviricetes__na;Caudoviricetes__na;Vibrio_phage_
1.084.O._10N.261.49.F5"	0	0	0	0	0	0	0	0	0	0	0
0	0	0	0	0	0	0	0	0	0	0	0	0
0	0	0	0	0	0	0	0	0	0	0	0	0
0	0	0	0	0	0	0	0	0	0	1	0	0
0	0	0	0	0	0	0	0	0	0	0	0	0
0	0	0	0	0	0	0	0	0
"Viruses;Uroviricota;Caudoviricetes;Caudoviricetes__na;Caudoviricetes__na;Caudoviricetes__na;Vibrio_phage_
1.123.O._10N.286.48.F3"	0	0	0	0	2	1	0	0	0	0	0
0	0	0	0	0	0	0	0	0	0	0	0	0
0	0	0	0	0	0	0	0	0	0	0	0	0
0	0	0	0	2	0	0	0	0	0	0	0	0
0	0	0	0	0	0	0	0	0	0	0	0	0
0	0	0	0	0	0	0	0	0
"Viruses;Uroviricota;Caudoviricetes;Caudoviricetes__na;Caudoviricetes__na;Caudoviricetes__na;Vibrio_phage_
1.127.O._10N.286.52.E12"	0	0	1	0	0	0	0	0	0	0
0	0	0	0	0	0	0	0	0	0	0	0	0
0	0	0	0	0	0	0	0	0	0	0	0	0
0	0	0	0	0	0	0	0	0	0	0	0	0
0	0	0	0	0	0	0	0	0	0	0	0	0
0	0	0	0	0	0	0	0	0	0
"Viruses;Uroviricota;Caudoviricetes;Caudoviricetes__na;Caudoviricetes__na;Caudoviricetes__na;Vibrio_phage_
1.133.O._10N.222.51.E4"	0	0	0	2	0	0	0	0	0	0	0
0	0	0	0	0	0	0	0	0	0	0	0	0
0	0	0	0	0	0	0	0	0	0	0	0	0
0	0	0	0	0	0	0	0	0	0	0	0	0
0	0	0	0	0	0	0	0	0	0	0	0	0
0	0	0	0	0	0	0	0	0
"Viruses;Uroviricota;Caudoviricetes;Caudoviricetes__na;Caudoviricetes__na;Caudoviricetes__na;Vibrio_phage_
1.154.O._10N.222.52.B12"	0	0	0	1	0	0	0	0	0	0
0	0	0	0	0	0	0	0	0	0	0	0	0
0	0	0	0	0	0	0	0	0	0	0	0	0
0	0	0	0	0	0	0	0	0	0	0	0	0
0	0	0	0	0	0	0	0	0	0	0	0	0
0	0	0	0	0	0	0	0	0	0
"Viruses;Uroviricota;Caudoviricetes;Caudoviricetes__na;Caudoviricetes__na;Caudoviricetes__na;Vibrio_phage_
1.191.O._10N.286.52.B4"	0	0	0	0	0	0	0	0	0	0	0
0	0	0	0	0	0	0	0	0	0	0	0	0
0	0	0	0	0	0	0	0	0	0	0	0	0
0	0	0	0	0	0	0	0	0	0	0	0	0

0 0 0 0 0 0 0 0 0 0 0 0 0
0 0 0 0 0 0 0 3 0
"Viruses;Uroviricota;Caudoviricetes;Caudoviricetes__na;Caudoviricetes__na;Caudoviricetes__na;Vibrio_phage_
1.213.O._10N.222.54.F10" 0 0 0 0 1 2 0 0 0 0
0 0 0 0 0 0 0 0 0 0 0 0 0
0 0 0 0 0 0 0 0 0 0 0 0 0
0 0 0 0 0 0 0 0 0 0 0 0 0
0 0 0 0 0 0 0 0 0 0 0 0 0
0 0 0 0 0 0 0 0 0 0
"Viruses;Uroviricota;Caudoviricetes;Caudoviricetes__na;Caudoviricetes__na;Caudoviricetes__na;Vibrio_phage_
1.262.O._10N.286.51.A9"0 0 0 0 5 1 0 0 0 0 0
0 0 0 0 0 0 0 0 0 0 0 0 0
0 0 0 0 0 0 0 0 0 0 0 0 0
0 0 0 0 0 0 0 0 0 0 0 0 0
0 0 0 0 0 0 0 0 0 0 0 0 0
0 0 0 0 0 0 0 0 0
"Viruses;Uroviricota;Caudoviricetes;Caudoviricetes__na;Caudoviricetes__na;Caudoviricetes__na;Vibrio_phage_
11895-B1" 0 0 0 1 0 0 0 0 0 0 0 0
0 0 0 0 0 0 0 0 0 0 0 0 0
0 0 0 0 0 0 0 0 0 0 0 0 0
0 0 0 0 0 0 0 0 0 0 0 0 0
0 0 0 0 0 0 0 0 0 0 0 0 0
0 0 0 0 0 0 0 0
"Viruses;Uroviricota;Caudoviricetes;Caudoviricetes__na;Caudoviricetes__na;Caudoviricetes__na;Vibrio_phage_
2.275.O._10N.286.54.E11" 0 0 0 0 0 113 0 0 0 0
0 0 0 0 0 0 0 0 0 0 0 0 0
0 0 0 0 0 0 0 0 0 0 0 0 0
0 0 0 0 0 0 0 0 0 0 0 0 0
0 0 0 0 0 0 0 0 0 0 0 0 0
0 0 0 0 0 0 0 0 0 0
"Viruses;Uroviricota;Caudoviricetes;Caudoviricetes__na;Caudoviricetes__na;Caudoviricetes__na;Vibrio_phage_
24" 0 0 0 0 2 0 0 0 0 0 0 0 0
0 0 0 0 0 0 0 0 0 0 0 0 0
0 0 0 0 0 0 0 0 0 0 0 0 0
0 0 0 0 0 0 0 0 0 0 0 0 0
0 0 0 0 0 0 0 0 0 0 0 0 0
0 0 0 0 0 0 0
"Viruses;Uroviricota;Caudoviricetes;Caudoviricetes__na;Caudoviricetes__na;Caudoviricetes__na;Vibrio_phage_
LP.1" 0 0 0 1 16 0 0 0 0 0 0 0 0
0 0 0 0 0 0 0 0 0 0 2 0 0
0 0 0 0 0 0 0 0 0 2 0 0 0
0 0 0 0 0 0 0 0 10 0 0 0 0
0 0 0 0 0 0 0 0 0 0 0 0 0
0 0 0 0 0 0 0
"Viruses;Uroviricota;Caudoviricetes;Caudoviricetes__na;Caudoviricetes__na;Caudoviricetes__na;Vibrio_phage_
LV6" 0 0 0 0 18 0 0 0 0 0 0 0 0
0 0 0 0 0 0 0 0 0 0 0 0 0
0 0 0 0 0 0 0 0 0 0 0 0 0
0 0 0 0 0 0 0 0 0 0 0 0 0

0 0 0 0 0 0 0 0 0 0 0 0 0
0 0 0 0 0 0 0
"Viruses;Uroviricota;Caudoviricetes;Caudoviricetes__na;Caudoviricetes__na;Caudoviricetes__na;Vibrio_phage_
Phriendly" 0 0 0 0 16 1 0 0 0 0 0 0
0 0 0 0 0 0 0 0 0 0 0 0 0
0 0 0 0 0 0 0 0 0 0 0 0 0
0 0 0 0 0 0 0 0 0 0 0 0 0
0 0 0 0 0 0 0 0 0 0 0 0 0
0 0 0 0 0 0 0 0
"Viruses;Uroviricota;Caudoviricetes;Caudoviricetes__na;Caudoviricetes__na;Caudoviricetes__na;Vibrio_phage_
PS25B.1" 0 0 0 0 1 0 0 0 0 0 0 0
0 0 0 0 0 0 0 0 0 0 0 0 0
0 0 0 0 0 0 0 0 0 0 0 0 0
0 0 0 0 0 0 0 0 0 0 0 0 0
0 0 0 0 0 0 0 0 0 0 0 0 0
0 0 0 0 0 0 0 0
"Viruses;Uroviricota;Caudoviricetes;Caudoviricetes__na;Caudoviricetes__na;Caudoviricetes__na;Vibrio_phage_
PS34B.1" 0 0 0 0 0 1 0 0 0 0 0 0
0 0 0 0 0 0 0 0 0 0 0 0 0
0 0 0 0 0 0 0 0 0 0 0 0 0
0 0 0 0 0 0 0 0 0 0 0 0 0
0 0 0 0 0 0 0 0 0 0 0 0 0
0 0 0 0 0 0 0 0
"Viruses;Uroviricota;Caudoviricetes;Caudoviricetes__na;Caudoviricetes__na;Caudoviricetes__na;Vibrio_phage_
PVA1" 0 0 1 2 0 0 0 0 0 0 0 0 0
0 0 0 0 0 0 0 0 0 0 0 0 0
0 0 0 0 0 0 0 0 0 0 0 0 0
0 0 0 0 0 0 0 0 0 0 0 0 0
0 0 0 0 0 0 0 0 0 0 0 0 0
0 0 0 0 0 0 0
"Viruses;Uroviricota;Caudoviricetes;Caudoviricetes__na;Caudoviricetes__na;Caudoviricetes__na;Vibrio_phage_
pYD21-A" 0 0 0 0 1 0 0 0 0 0 0 0
0 0 0 0 0 0 0 0 0 0 0 0 0
0 0 0 0 0 0 0 0 0 0 0 0 0
0 0 0 0 0 0 0 0 0 0 0 0 0
0 0 0 0 0 0 0 0 0 0 0 0 0
0 0 0 0 0 0 0 0
"Viruses;Uroviricota;Caudoviricetes;Caudoviricetes__na;Caudoviricetes__na;Caudoviricetes__na;Vibrio_phage_
vB_VhaM_VH-8" 0 0 0 0 0 1 0 0 0 0 0
0 0 0 0 0 0 0 0 0 0 0 0 0
0 0 0 0 0 0 0 0 0 0 0 0 0
0 0 0 0 0 0 0 0 0 0 3 0 0
0 0 0 0 0 0 0 0 0 0 0 0 0
0 0 0 0 0 0 0 0 0
"Viruses;Uroviricota;Caudoviricetes;Caudoviricetes__na;Caudoviricetes__na;Caudoviricetes__na;Vibrio_phage_
vB_VnaS-L3" 0 0 2 0 6 0 0 0 0 0 0 0
0 0 0 0 0 0 0 0 0 0 0 0 0
0 0 0 0 0 0 0 0 0 0 0 0 0
0 0 0 0 0 0 0 0 0 0 0 0 0

0 0 0 0 0 0 0 0 0 0 0 0 0
0 0 0 0 0 0 0 0
"Viruses;Uroviricota;Caudoviricetes;Caudoviricetes__na;Caudoviricetes__na;Caudoviricetes__na;Vibrio_phage_vB_VpaM_R16F" 0 0 0 0 1 0 0 0 0 0 0
0 0 0 0 0 0 0 0 0 0 0 0 0
0 0 0 0 0 0 0 0 0 0 0 0 0
0 0 0 0 0 0 0 0 0 0 0 0 0
0 0 0 0 0 0 0 0 0 0 0 0 0
0 0 0 0 0 0 0 0 0
"Viruses;Uroviricota;Caudoviricetes;Caudoviricetes__na;Caudoviricetes__na;Caudoviricetes__na;Vibrio_phage_vB_VpP_BT-1011" 0 0 0 0 0 1 0 0 0 0 0
0 0 0 0 0 0 0 0 0 0 0 0 0
0 0 0 0 0 0 0 0 0 0 0 0 0
0 0 0 0 0 0 0 0 0 0 0 0 0
0 0 0 0 0 0 0 0 0 0 0 0 0
0 0 0 0 0 0 0 0 0
"Viruses;Uroviricota;Caudoviricetes;Caudoviricetes__na;Caudoviricetes__na;Caudoviricetes__na;Vibrio_phage_Vp_R1" 0 0 0 0 0 5 0 0 0 0 0 0 0
0 0 0 0 0 0 0 0 0 0 0 0 0
0 0 0 0 0 0 0 0 0 0 0 0 0
0 0 0 0 0 0 0 0 0 0 0 0 0
0 0 0 0 0 0 0 0 0 0 0 0 0
0 0 0 0 0 0 0
"Viruses;Uroviricota;Caudoviricetes;Caudoviricetes__na;Caudoviricetes__na;Caudoviricetes__na;Vibrio_phage_VpaJT_1" 0 0 0 0 7 0 0 0 0 0 0 0
0 0 0 0 0 0 0 0 0 0 0 0 0
0 0 0 0 0 0 0 0 0 0 0 0 0
0 0 0 0 0 0 0 0 0 0 0 0 0
0 0 0 0 0 0 0 0 0 0 0 0 0
0 0 0 0 0 0 0 0
"Viruses;Uroviricota;Caudoviricetes;Caudoviricetes__na;Caudoviricetes__na;Caudoviricetes__na;Vibrio_phage_VPMCC14" 0 0 0 0 0 2 0 0 0 0 0 0
0 0 0 0 0 0 0 0 0 0 0 0 0
0 0 0 0 0 0 0 0 0 0 0 0 0
0 0 0 0 0 0 0 0 0 0 0 0 0
0 0 0 0 0 0 0 0 0 0 0 0 0
0 0 0 0 0 0 0 0
"Viruses;Uroviricota;Caudoviricetes;Caudoviricetes__na;Caudoviricetes__na;Caudoviricetes__na;Vibrio_phage_VPp1" 0 0 0 0 3 0 0 0 0 0 0 0 0
0 0 0 0 0 0 0 0 0 0 0 0 0
0 0 0 0 0 0 0 0 0 0 0 0 0
0 0 0 0 0 0 0 0 0 0 0 0 0
0 0 0 0 0 0 0 0 0 0 0 0 0
0 0 0 0 0 0 0
"Viruses;Uroviricota;Caudoviricetes;Caudoviricetes__na;Caudoviricetes__na;Caudoviricetes__na;Vibrio_phage_VvAW1" 0 0 0 6 0 1 0 0 0 0 0 0
0 0 0 0 0 0 0 0 0 0 0 0 0
0 0 0 0 0 0 0 0 0 0 0 0 0
0 0 0 0 0 0 0 0 0 0 0 0 0

0 0 0 0 0 0 0 0 0 0 0 0 0
0 0 0 0 0 0 0 0
"Viruses;Uroviricota;Caudoviricetes;Caudoviricetes__na;Caudoviricetes__na;Caudoviricetes__na;Virus_Rctr41k
" 0 0 0 2 62 0 0 0 0 0 0 0 0
0 0 0 0 0 0 0 0 0 0 0 0 0
0 0 0 0 0 0 0 0 1 0 0 0 0
0 0 0 0 0 0 0 0 1 0 0 0 0
0 0 0 0 0 0 0 0 0 0 0 0 0
0 0 0 0 0 0 0
"Viruses;Uroviricota;Caudoviricetes;Caudoviricetes__na;Caudoviricetes__na;Caudoviricetes__na;Xanthomonas_
phage_vB_XciM_LucasX" 0 0 0 1 0 1 0 0 0 0
0 0 0 0 0 0 0 0 0 0 0 0 0
0 0 0 0 0 0 0 0 0 0 0 0 0
0 0 0 0 0 0 0 0 0 0 0 0 0
0 0 0 0 0 0 0 0 0 0 0 0 0
0 0 0 0 0 0 0 0 0 0
"Viruses;Uroviricota;Caudoviricetes;Caudoviricetes__na;Caudoviricetes__na;Caudoviricetes__na;Xanthomonas_
phage_X1" 0 0 0 1 0 0 0 0 0 0 0 0
0 0 0 0 0 0 0 0 0 0 0 0 0
0 0 0 0 0 0 0 0 0 0 0 0 0
0 0 0 0 0 0 0 0 0 0 0 0 0
0 0 0 0 0 0 0 0 0 0 0 0 0
0 0 0 0 0 0 0 0
"Viruses;Uroviricota;Caudoviricetes;Caudoviricetes__na;Caudoviricetes__na;Caudoviricetes__na;Xanthomonas_
phage_XacN1" 0 0 0 2 1 14 0 0 0 0 0 0
0 0 0 0 0 0 0 0 0 0 0 0 0
0 0 0 0 0 0 0 0 0 1 1 0 0
0 0 0 0 0 0 0 0 0 0 0 0 0
0 0 0 0 0 0 0 0 0 0 0 0 0
0 0 0 0 0 0 0 0
"Viruses;Uroviricota;Caudoviricetes;Caudoviricetes__na;Caudoviricetes__na;Caudoviricetes__na;Xanthomonas_
phage_Xoo-sp13" 0 0 0 3 14 0 0 0 0 0 0
0 0 0 0 0 0 0 0 0 0 0 0 0
0 0 0 0 0 0 0 0 0 0 0 0 0
0 0 0 0 0 0 0 0 0 0 0 0 0
0 0 0 0 0 0 0 0 0 0 0 0 0
0 0 0 0 0 0 0 1 0
"Viruses;Uroviricota;Caudoviricetes;Caudoviricetes__na;Caudoviricetes__na;Caudoviricetes__na;Yersinia_phag
e_fEV-1" 0 0 0 0 0 1 0 0 0 0 0 0
0 0 0 0 0 0 0 0 0 0 0 0 0
0 0 0 0 0 0 0 0 0 0 0 0 0
0 0 0 0 0 0 0 0 0 0 0 0 0
0 0 0 0 0 0 0 0 0 0 0 0 0
0 0 0 0 0 0 0 0
"Viruses;Uroviricota;Caudoviricetes;Caudoviricetes__na;Caudoviricetes__na;Caudoviricetes__na;Yersinia_phag
e_YerA41" 0 0 0 0 1 0 0 0 0 0 0 0
0 0 0 0 0 0 0 0 0 0 0 0 0
0 0 0 0 0 0 0 0 0 0 0 0 0
0 0 0 0 0 0 0 0 0 0 0 0 0

0 0 0 0 0 0 0 0 0 0 0 0 0
0 0 0 0 0 0 0 0
"Viruses;Uroviricota;Caudoviricetes;Caudoviricetes__na;Caudoviricetes__na;Cbastvirus;Cbastvirus_ST" 0
0 1 1 0 22 0 0 0 0 0 0 0 0
0 0 0 0 0 0 0 0 0 0 0 0 0
0 0 0 0 0 0 0 0 0 0 0 0 0
0 0 0 0 0 0 0 0 0 0 0 0 0
0 0 0 0 0 0 0 0 0 0 0 0 0
0 0 0 0 1 0
"Viruses;Uroviricota;Caudoviricetes;Caudoviricetes__na;Caudoviricetes__na;Cbastvirus;Tenacibaculum_phage_
Larrie" 0 0 0 0 6 0 0 0 0 0 0 0 0
0 0 0 0 0 0 0 0 0 0 0 0 0
0 0 0 0 0 0 0 0 0 0 0 0 0
0 0 0 0 0 0 0 0 0 0 0 0 0
0 0 0 0 0 0 0 0 0 0 0 0 0
0 0 0 0 0 0 0
"Viruses;Uroviricota;Caudoviricetes;Caudoviricetes__na;Caudoviricetes__na;Ceduovirus;Ceduovirus__uc"
0 0 0 0 0 0 0 0 0 0 0 0 1
0 2 2 10 46 0 0 0 0 2 0 0 0
0 0 0 0 0 0 0 0 1 0 0 0 0
0 4 0 0 1 5 1 0 0 6 1 10 1
0 0 16 28 0 0 0 0 0 0 0 0 0
0 0 0 0 0 0 0
"Viruses;Uroviricota;Caudoviricetes;Caudoviricetes__na;Caudoviricetes__na;Ceduovirus;Ceduovirus_bIBB14"
0 0 0 0 0 0 0 0 0 0 0 0 0
0 0 0 0 0 0 0 0 0 0 0 0 0
0 0 0 0 0 0 0 0 1 0 0 0 0
0 0 0 0 0 0 0 0 0 0 0 0 0
0 0 2 6 0 0 0 0 0 0 0 0 0
0 0 0 0 0 0 0
"Viruses;Uroviricota;Caudoviricetes;Caudoviricetes__na;Caudoviricetes__na;Ceduovirus;Ceduovirus_bIBBA3"
0 0 0 0 0 0 0 0 0 0 0 0 1
0 0 0 0 0 0 0 0 0 0 0 0 0
0 0 0 0 0 0 0 0 0 0 0 0 0
0 0 0 0 0 1 0 0 0 0 1 0 0
0 0 1 2 0 0 0 0 0 0 0 0 0
0 0 0 0 0 0 0
"Viruses;Uroviricota;Caudoviricetes;Caudoviricetes__na;Caudoviricetes__na;Ceduovirus;Ceduovirus_bIBBAm4
" 0 0 0 0 0 0 0 0 0 0 0 0 0
0 0 0 0 0 0 0 0 0 0 0 0 0
0 0 0 0 0 1 0 0 0 0 0 0 0
0 0 0 0 0 2 0 0 0 0 0 2 0
0 0 1 0 0 0 0 0 0 0 0 0 0
0 0 0 0 0 0 0
"Viruses;Uroviricota;Caudoviricetes;Caudoviricetes__na;Caudoviricetes__na;Ceduovirus;Ceduovirus_bIBBE1"
0 0 0 0 0 0 0 0 0 0 0 0 0
0 0 0 0 0 0 0 0 0 0 0 0 0
0 0 0 0 0 0 0 0 0 0 0 0 0
0 1 0 0 0 0 0 0 0 0 0 0 0

0 0 0 0 0 0 0 0 0 0 0 0 0
0 0 0 0 0 1 0
"Viruses;Uroviricota;Caudoviricetes;Caudoviricetes__na;Caudoviricetes__na;Ceduovirus;Ceduovirus_bIBBL12"
0 0 0 0 0 0 0 0 0 0 0 0 0
0 0 0 0 0 0 0 0 0 0 0 0 0
0 0 0 0 0 0 0 0 0 0 0 0 0
0 0 0 0 0 0 0 0 0 0 0 0 0
0 0 0 1 0 0 0 0 0 0 0 0 0
0 0 0 0 0 0 0
"Viruses;Uroviricota;Caudoviricetes;Caudoviricetes__na;Caudoviricetes__na;Ceduovirus;Ceduovirus_bIBBp64"
0 0 0 0 0 0 0 0 0 0 0 0 0
0 0 0 0 0 0 0 0 0 0 0 0 0
0 0 0 0 0 0 0 0 0 0 0 0 0
0 0 0 0 0 0 0 0 0 0 0 0 0
0 0 8 6 0 0 0 0 0 0 0 0 0
0 0 0 0 0 1 0
"Viruses;Uroviricota;Caudoviricetes;Caudoviricetes__na;Caudoviricetes__na;Ceduovirus;Ceduovirus_bIL67"
0 0 0 0 0 0 0 0 0 0 0 0 0
0 0 0 0 0 0 0 0 0 0 0 0 0
0 0 0 0 0 0 0 0 0 0 0 0 0
0 1 0 0 0 0 0 0 0 0 0 0 0
0 0 0 0 0 0 0 0 0 0 0 0 0
0 0 0 0 0 0 0
"Viruses;Uroviricota;Caudoviricetes;Caudoviricetes__na;Caudoviricetes__na;Ceduovirus;Ceduovirus_blBB94p4
" 0 0 0 0 0 0 0 0 0 0 0 0 0
0 0 0 0 3 0 0 0 0 0 0 0 0
0 0 0 0 0 0 0 0 0 0 0 0 0
0 0 0 0 0 0 0 0 0 0 0 0 0
0 0 0 1 0 0 0 0 0 0 0 0 0
0 0 0 0 0 0 0
"Viruses;Uroviricota;Caudoviricetes;Caudoviricetes__na;Caudoviricetes__na;Ceduovirus;Ceduovirus_c2"0
0 0 0 0 0 0 0 0 0 0 0 0 0
0 1 0 0 0 0 0 0 0 0 0 0 0
0 0 0 0 0 0 0 0 0 0 0 0 0
0 0 0 0 0 0 0 0 0 0 4 0 0
0 8 11 0 0 0 0 0 0 0 0 0 0
0 0 0 0 0 0
"Viruses;Uroviricota;Caudoviricetes;Caudoviricetes__na;Caudoviricetes__na;Ceduovirus;Ceduovirus_CHPC102
0" 0 0 0 0 0 0 0 0 0 0 0 0 0
0 0 0 0 0 0 0 0 0 0 0 0 0
0 0 0 0 0 0 0 0 0 0 0 0 0
0 0 0 0 0 1 0 0 0 0 0 1 0
0 0 2 7 0 0 0 0 0 0 0 0 0
0 0 0 0 0 0 0
"Viruses;Uroviricota;Caudoviricetes;Caudoviricetes__na;Caudoviricetes__na;Ceduovirus;Ceduovirus_CHPC116
" 0 0 0 0 0 0 0 0 0 0 0 0 0
0 1 0 0 0 0 0 0 0 0 0 0 0
0 0 0 0 0 0 0 0 0 0 0 0 0
0 0 0 0 0 2 0 0 0 0 0 1 0

0 0 0 4 0 0 0 0 0 0 0 0 0
0 0 0 0 0 0 0
"Viruses;Uroviricota;Caudoviricetes;Caudoviricetes__na;Caudoviricetes__na;Ceduovirus;Ceduovirus_CHPC117
0" 0 0 0 0 0 0 0 0 0 0 0 0 0
0 0 0 0 0 0 0 0 0 0 0 0 0
0 0 0 0 0 0 0 0 0 0 0 0 0
0 1 0 0 0 4 3 0 0 2 1 14 0
0 0 3 7 0 0 0 0 0 0 0 0 0
0 0 0 0 0 0 0
"Viruses;Uroviricota;Caudoviricetes;Caudoviricetes__na;Caudoviricetes__na;Ceduovirus;Ceduovirus_CHPC118
2" 0 0 0 0 0 0 0 0 0 0 0 0 0
0 0 0 0 0 0 0 0 0 0 0 0 0
0 0 0 0 0 0 0 0 0 0 0 0 0
0 0 0 0 0 0 0 0 0 0 0 0 0
0 0 0 1 0 0 0 0 0 0 0 0 0
0 0 0 0 0 0 0
"Viruses;Uroviricota;Caudoviricetes;Caudoviricetes__na;Caudoviricetes__na;Ceduovirus;Ceduovirus_CHPC118
3" 0 0 0 0 0 0 0 0 0 0 0 0 1
0 0 0 0 0 0 0 0 0 0 0 0 0
0 0 0 0 0 0 0 0 0 0 0 0 0
0 0 0 0 0 0 0 0 0 0 0 0 0
0 0 0 0 0 0 0 0 0 0 0 0 0
0 0 0 0 0 0 0
"Viruses;Uroviricota;Caudoviricetes;Caudoviricetes__na;Caudoviricetes__na;Ceduovirus;Ceduovirus_CHPC122
" 0 0 0 0 0 0 0 0 0 0 0 0 0
0 0 0 0 0 0 0 0 0 0 0 0 0
0 0 0 0 0 0 0 0 0 0 0 0 0
0 0 0 0 0 0 0 0 0 0 0 1 0
0 0 3 2 0 0 0 0 0 0 0 0 0
0 0 0 0 0 0 0
"Viruses;Uroviricota;Caudoviricetes;Caudoviricetes__na;Caudoviricetes__na;Ceduovirus;Ceduovirus_CHPC124
2" 0 0 0 0 0 0 0 0 0 0 0 0 0
0 0 0 0 0 0 0 0 0 0 0 0 0
0 0 0 0 0 0 0 0 0 0 0 0 0
0 0 0 0 0 3 0 0 0 3 1 0 1
0 0 3 3 0 0 0 0 1 0 0 0 0
0 0 0 0 0 0 0
"Viruses;Uroviricota;Caudoviricetes;Caudoviricetes__na;Caudoviricetes__na;Ceduovirus;Ceduovirus_CHPC966
" 0 0 0 0 0 0 0 0 0 0 0 0 0
0 0 1 0 0 0 0 0 0 0 0 0 0
0 0 0 0 0 0 0 0 0 0 0 0 0
0 0 0 0 0 0 0 0 0 0 0 0 0
0 0 0 0 0 0 0 0 0 0 0 0 0
0 0 0 0 0 0 0
"Viruses;Uroviricota;Caudoviricetes;Caudoviricetes__na;Caudoviricetes__na;Ceduovirus;Ceduovirus_CHPC967
" 0 0 0 0 0 0 0 0 0 0 0 0 0
0 0 0 0 0 0 0 0 0 0 0 0 0
0 0 0 0 0 0 0 0 0 0 0 0 0
0 0 0 0 0 1 0 0 0 0 0 0 0

0 0 0 0 0 0 0 0 0 0 0 0 0
0 0 0 0 0 0 0
"Viruses;Uroviricota;Caudoviricetes;Caudoviricetes__na;Caudoviricetes__na;Ceduovirus;Ceduovirus_CHPC972
" 0 0 0 0 0 0 0 0 0 0 0 0 0
0 0 0 0 0 0 0 0 0 0 0 0 0
0 0 0 0 0 0 0 0 0 0 0 0 0
0 0 0 0 0 0 0 0 0 0 0 0 0
0 0 0 1 0 0 0 0 0 0 0 0 0
0 0 0 0 0 0 0
"Viruses;Uroviricota;Caudoviricetes;Caudoviricetes__na;Caudoviricetes__na;Ceduovirus;Ceduovirus_cv05802"
0 0 0 0 0 0 0 0 0 0 0 0 0
0 0 0 0 0 0 0 0 0 0 0 0 0
0 0 0 0 0 0 0 0 0 0 0 0 0
0 0 0 0 0 0 0 0 0 0 0 0 0
0 0 2 2 0 0 0 0 0 0 0 0 0
0 0 0 0 0 0 0
"Viruses;Uroviricota;Caudoviricetes;Caudoviricetes__na;Caudoviricetes__na;Ceduovirus;Ceduovirus_cv20R03
M" 0 0 0 0 0 0 0 0 0 0 0 0 0
0 0 0 0 1 0 0 0 0 0 0 0 0
0 0 0 0 0 0 0 0 0 0 0 0 0
0 0 0 0 0 1 0 0 0 1 0 2 0
0 0 3 1 0 0 0 0 0 0 0 0 0
0 0 0 0 0 0 0
"Viruses;Uroviricota;Caudoviricetes;Caudoviricetes__na;Caudoviricetes__na;Ceduovirus;Ceduovirus_cv37203"
0 0 0 0 0 0 0 0 0 0 0 0 0
0 0 0 0 0 0 0 0 0 0 0 0 0
0 0 0 0 0 0 0 0 0 0 0 0 0
0 0 0 0 0 2 0 0 0 0 0 2 0
0 0 7 14 0 0 0 0 0 0 0 0 0
0 0 0 0 0 0 0
"Viruses;Uroviricota;Caudoviricetes;Caudoviricetes__na;Caudoviricetes__na;Ceduovirus;Ceduovirus_cv50102"
0 0 0 0 0 0 0 0 0 0 0 0 0
0 0 0 0 0 0 0 0 0 0 0 0 0
0 0 0 0 0 0 0 0 0 0 0 0 0
0 0 0 0 0 0 0 0 0 0 0 1 0
0 0 1 0 0 0 0 0 0 0 0 0 0
0 0 0 0 0 0 0
"Viruses;Uroviricota;Caudoviricetes;Caudoviricetes__na;Caudoviricetes__na;Ceduovirus;Ceduovirus_cv50504"
0 0 0 0 0 0 0 0 0 0 0 0 0
0 0 0 0 0 0 0 0 0 0 0 0 0
0 0 0 0 0 0 0 0 0 0 0 0 0
0 0 0 0 0 0 0 0 0 0 0 0 0
0 0 1 3 0 0 0 0 0 0 0 0 0
0 0 0 0 0 0 0
"Viruses;Uroviricota;Caudoviricetes;Caudoviricetes__na;Caudoviricetes__na;Ceduovirus;Ceduovirus_cv62402"
0 0 0 0 0 0 0 0 0 0 0 0 0
0 0 0 0 0 0 0 0 0 0 0 0 0
0 0 0 0 0 0 0 0 0 0 0 0 0
0 0 0 0 0 0 0 0 0 0 0 0 0

0 0 0 3 0 0 0 0 0 0 0 0 0
0 0 0 0 0 0 0
"Viruses;Uroviricota;Caudoviricetes;Caudoviricetes__na;Caudoviricetes__na;Ceduovirus;Ceduovirus_cv62403"
0 0 0 0 0 0 0 0 0 0 0 0 0
0 0 0 0 0 0 0 0 0 0 0 0 0
0 0 0 0 0 0 0 0 0 0 0 0 0
0 0 0 0 0 0 0 0 0 0 0 0 0
0 0 0 2 0 0 0 0 0 0 0 0 0
0 0 0 0 0 0 0
"Viruses;Uroviricota;Caudoviricetes;Caudoviricetes__na;Caudoviricetes__na;Ceduovirus;Ceduovirus_cv62606"
0 0 0 0 0 0 0 0 0 0 0 0 1
1 1 0 1 0 0 0 0 0 0 0 0 0
0 0 0 0 0 0 0 0 0 0 0 0 0
0 0 0 0 0 0 0 0 0 0 0 0 0
0 0 3 2 0 0 0 0 0 0 0 0 0
0 0 0 0 0 0 0
"Viruses;Uroviricota;Caudoviricetes;Caudoviricetes__na;Caudoviricetes__na;Ceduovirus;Ceduovirus_D4412"
0 0 0 0 0 0 0 0 0 0 0 0 0
0 0 0 0 0 0 0 0 0 0 0 0 0
0 0 0 0 0 0 0 0 0 0 0 0 0
0 0 0 0 0 0 0 0 0 0 0 0 0
0 0 2 0 0 0 0 0 0 0 0 0 0
0 0 0 0 0 0 0
"Viruses;Uroviricota;Caudoviricetes;Caudoviricetes__na;Caudoviricetes__na;Ceduovirus;Ceduovirus_LacS15"
0 0 0 0 0 0 0 0 0 0 0 0 0
0 0 0 0 0 0 0 0 0 0 0 0 0
0 0 0 0 0 0 0 0 0 0 0 0 0
0 0 0 0 0 5 0 0 0 0 0 0 0
0 0 2 3 0 0 0 0 0 0 0 0 0
0 0 0 0 0 0 0
"Viruses;Uroviricota;Caudoviricetes;Caudoviricetes__na;Caudoviricetes__na;Ceduovirus;Ceduovirus_M5938"
0 0 0 0 0 0 0 0 0 0 0 0 0
0 0 0 0 0 0 0 0 0 0 0 0 0
0 0 0 0 0 0 0 0 0 0 0 0 0
0 0 0 0 0 1 0 0 0 0 0 0 0
0 0 3 1 0 0 1 0 0 0 0 0 0
0 0 0 0 0 0 0
"Viruses;Uroviricota;Caudoviricetes;Caudoviricetes__na;Caudoviricetes__na;Ceduovirus;Ceduovirus_PCB1"
0 0 0 0 0 0 0 0 0 0 0 0 0
0 1 0 0 2 0 0 0 0 0 0 0 0
0 0 0 0 0 0 0 0 0 0 0 0 0
0 0 0 0 0 0 0 0 0 0 0 0 0
0 0 0 0 0 0 0 0 0 0 0 0 0
0 0 0 0 0 0 0
"Viruses;Uroviricota;Caudoviricetes;Caudoviricetes__na;Caudoviricetes__na;Ceduovirus;Lactococcus_phage_52
05F" 0 0 0 0 0 0 0 0 0 0 0 0 0
0 0 0 0 0 0 0 0 0 0 0 0 0
0 0 0 0 0 0 0 0 0 0 0 0 0
0 0 0 0 0 0 1 0 0 0 0 0 0

0 0 0 0 0 0 0 0 0 0 0 0 0
0 0 0 0 0 0 0
"Viruses;Uroviricota;Caudoviricetes;Caudoviricetes__na;Caudoviricetes__na;Ceduovirus;Lactococcus_phage_BIM_BV-114" 0 0 0 0 0 0 0 0 0 0 0 0
0 0 0 0 1 0 0 0 0 0 0 0 0
0 0 0 0 0 0 0 0 0 0 0 0 0
0 0 0 0 0 0 3 0 0 0 2 0 1
0 0 0 5 9 0 0 0 0 0 0 0 0
0 0 0 0 0 0 0 0
"Viruses;Uroviricota;Caudoviricetes;Caudoviricetes__na;Caudoviricetes__na;Ceduovirus;Lactococcus_phage_CB17" 0 0 0 0 0 0 0 0 0 0 0 0 0
0 0 0 0 0 0 0 0 0 0 0 0 0
0 0 0 0 0 0 0 0 0 0 0 0 0
0 0 0 0 0 0 0 0 0 0 0 0 0
0 0 1 2 0 0 0 0 0 0 0 0 0
0 0 0 0 0 0 0
"Viruses;Uroviricota;Caudoviricetes;Caudoviricetes__na;Caudoviricetes__na;Ceduovirus;Lactococcus_phage_P001" 0 0 0 0 0 0 0 0 0 0 0 0 0
0 0 0 0 0 0 0 0 0 0 0 0 0
0 0 0 0 0 0 0 0 0 0 0 0 0
0 0 0 0 0 1 0 0 0 0 0 0 0
0 0 0 0 0 0 0 0 0 0 0 0 0
0 0 0 0 0 0 0
"Viruses;Uroviricota;Caudoviricetes;Caudoviricetes__na;Caudoviricetes__na;Cequinquevirus;Cequinquevirus__uc" 0 0 0 0 0 0 0 0 0 0 0 0 0
0 0 0 0 0 0 0 0 0 0 0 0 0
0 0 0 0 0 0 0 0 0 0 0 0 0
0 0 0 0 0 0 0 0 0 0 0 0 0
0 4 0 0 0 0 0 0 0 0 0 0 0
0 2 0 0 0 0 0
"Viruses;Uroviricota;Caudoviricetes;Caudoviricetes__na;Caudoviricetes__na;Cequinquevirus;Cequinquevirus_Ld25A" 0 0 0 0 0 0 0 0 0 0 0 0 0
0 0 0 0 0 0 0 0 0 0 0 0 0
0 0 0 0 0 0 0 0 0 0 0 0 0
0 0 0 0 0 0 0 0 0 0 0 0 0
0 84 0 0 0 0 0 0 0 0 0 0 0
0 2 3 1 1 0 0
"Viruses;Uroviricota;Caudoviricetes;Caudoviricetes__na;Caudoviricetes__na;Certrevirus;Certrevirus__uc"
0 0 0 0 7 1 0 0 0 0 0 0 0
0 0 0 0 0 0 0 0 0 0 0 0 0
0 0 0 0 0 0 0 0 0 0 0 0 0
0 0 0 0 0 0 0 0 0 0 0 0 0
0 0 0 0 0 0 0 0 0 0 0 0 0
0 0 0 0 0 0 0
"Viruses;Uroviricota;Caudoviricetes;Caudoviricetes__na;Caudoviricetes__na;Certrevirus;Certrevirus_phiTE"
0 0 0 0 0 5 0 0 0 0 0 0 0
0 0 0 0 0 0 0 0 0 0 0 0 0
0 0 0 0 0 0 0 0 0 0 0 0 0
0 0 0 0 0 0 0 0 0 0 0 0 0

0 0 0 0 0 0 0 0 0 0 0 0 0
0 0 0 0 0 0 0
"Viruses;Uroviricota;Caudoviricetes;Caudoviricetes__na;Caudoviricetes__na;Chakrabartyvirus;Chakrabartyviru
s_pf16" 0 0 0 2 0 0 0 0 0 1 0 0 0
0 0 0 0 0 0 0 0 0 0 0 0 0
0 0 0 0 0 0 0 0 0 0 0 0 0
0 0 0 0 0 0 0 0 0 0 0 0 0
0 0 0 0 0 0 0 0 0 0 0 0 0
0 0 0 0 0 0 0
"Viruses;Uroviricota;Caudoviricetes;Caudoviricetes__na;Caudoviricetes__na;Cheoctovirus;Cheoctovirus__uc"
1 0 0 0 0 4 0 0 0 0 0 0 0
0 0 0 0 0 0 0 0 0 0 0 0 0
0 0 0 0 0 0 0 0 0 0 0 0 0
0 0 4 0 0 0 0 0 1 0 0 0 0
0 0 0 0 0 0 0 0 0 0 0 0 0
0 0 0 0 0 0 0
"Viruses;Uroviricota;Caudoviricetes;Caudoviricetes__na;Caudoviricetes__na;Colossusvirus;Colossusvirus_colos
sus" 0 0 0 0 0 0 0 0 0 0 0 0 0
0 0 0 0 0 0 0 0 0 0 0 0 0
0 0 0 0 0 0 0 0 1 0 0 0 0
0 0 0 0 0 1 2 0 0 0 0 0 0
0 0 0 0 0 0 0 0 0 0 0 0 0
0 0 0 0 0 0 0
"Viruses;Uroviricota;Caudoviricetes;Caudoviricetes__na;Caudoviricetes__na;Cornellvirus;Salmonella_phage_Sh
emara" 0 0 1 0 0 0 0 0 0 0 0 0 0
0 0 0 0 0 0 0 0 0 0 0 0 0
0 0 0 0 0 0 0 0 0 0 0 0 0
0 0 0 0 0 0 0 0 1 0 0 0 0
0 0 0 0 0 0 0 0 0 0 0 0 0
0 0 0 0 0 0 0
"Viruses;Uroviricota;Caudoviricetes;Caudoviricetes__na;Caudoviricetes__na;Cronusvirus;Dinoroseobacter_pha
ge_vB_DshS-R4C" 0 0 1 0 0 0 0 0 0 0 0
0 0 0 0 0 0 0 0 0 0 0 0 0
0 0 0 0 0 0 0 0 0 0 0 0 0
0 0 0 0 0 0 0 0 0 0 0 0 0
0 0 0 0 0 0 0 0 0 0 0 0 0
0 0 0 0 0 0 0 0 0
"Viruses;Uroviricota;Caudoviricetes;Caudoviricetes__na;Caudoviricetes__na;Delepquintavirus;Delepquintaviru
s__uc" 0 0 0 0 14 0 0 0 0 0 0 0 0
0 0 0 0 0 0 0 0 0 0 0 0 0
0 0 0 0 0 0 0 0 0 0 0 0 0
0 0 0 0 0 0 0 0 0 0 0 0 0
0 0 0 0 0 0 0 0 0 0 0 0 0
0 0 0 0 0 0 0
"Viruses;Uroviricota;Caudoviricetes;Caudoviricetes__na;Caudoviricetes__na;Delepquintavirus;Stenotrophomon
as_phage_vB_SmaS_DLP_3" 0 0 0 1 0 0 0 0 0 0
0 0 0 0 0 0 0 0 0 0 0 0 0
0 0 0 0 0 0 0 0 0 0 0 0 0
0 0 0 0 0 0 0 0 0 0 0 0 0

0 0 0 0 0 0 0 0 0 0 0 0 0
0 0 0 0 0 0 0 0 0 0
"Viruses;Uroviricota;Caudoviricetes;Caudoviricetes__na;Caudoviricetes__na;Derbicusvirus;Cronobacter_phage
_CR5" 0 0 0 0 0 0 0 0 0 0 0 0 0
0 0 0 0 0 0 0 0 0 0 0 0 0
0 0 0 0 0 0 0 0 0 0 0 0 0
0 0 0 0 0 1 0 0 0 0 0 0 0
0 0 0 0 0 0 0 0 0 0 0 0 0
0 0 0 0 0 0 0
"Viruses;Uroviricota;Caudoviricetes;Caudoviricetes__na;Caudoviricetes__na;Deseoctovirus;Escherichia_phage_
S192" 0 0 1 0 0 0 0 0 0 0 0 0 0
0 0 0 0 0 0 0 0 0 0 0 0 0
0 0 0 0 0 0 0 0 0 0 0 0 0
0 0 0 0 0 0 0 0 0 0 0 0 0
0 0 0 0 0 0 0 0 0 0 0 0 0
0 0 0 0 0 0 0
"Viruses;Uroviricota;Caudoviricetes;Caudoviricetes__na;Caudoviricetes__na;Detrevirus;Pseudomonas_phage_P
sp6" 0 0 0 0 0 1 0 0 0 0 0 0 0
0 0 0 0 0 0 0 0 0 0 0 0 0
0 0 0 0 0 0 0 0 0 0 0 0 0
0 0 0 0 0 0 0 0 0 0 0 0 0
0 0 0 0 0 0 0 0 0 0 0 0 0
0 0 0 0 0 0 0
"Viruses;Uroviricota;Caudoviricetes;Caudoviricetes__na;Caudoviricetes__na;Dismasvirus;Dismasvirus__uc"
0 0 0 0 0 0 0 0 0 0 0 0 0
0 0 0 0 0 0 0 0 0 0 0 0 0
0 0 0 0 0 0 0 0 0 0 0 0 0
0 0 0 0 0 0 0 7 0 0 0 0 0
0 0 0 0 0 0 0 0 0 0 0 0 0
0 0 0 0 0 0 0
"Viruses;Uroviricota;Caudoviricetes;Caudoviricetes__na;Caudoviricetes__na;Dismasvirus;Microbacterium_phag
e_AvGardian" 0 0 0 0 0 0 0 0 0 0 0 0
0 0 0 0 0 0 0 0 0 0 0 0 0
0 0 0 0 0 0 0 0 0 1 0 0 0
0 0 0 0 0 0 0 0 0 0 0 0 0
0 0 0 0 0 0 0 0 0 0 0 0 0
0 0 0 0 0 0 0 0
"Viruses;Uroviricota;Caudoviricetes;Caudoviricetes__na;Caudoviricetes__na;Donellivirus;Donellivirus_gee"
0 0 0 0 0 1 0 0 0 0 0 0 0
0 0 0 0 0 0 0 0 0 0 0 0 0
0 0 0 0 0 0 0 0 0 0 0 0 0
0 0 0 0 0 0 0 0 0 0 0 0 0
0 0 0 0 0 0 0 0 0 0 0 0 0
0 0 0 0 0 0 0
"Viruses;Uroviricota;Caudoviricetes;Caudoviricetes__na;Caudoviricetes__na;Douglaswolinvirus;Lactiplantibacil
lus_phage_Gut-P1" 0 0 0 0 0 0 0 0 0 0 0
0 0 0 0 0 0 0 0 1 2 3 0 0
0 0 0 0 0 0 0 0 0 0 0 0 0
0 0 0 0 0 0 0 0 0 0 0 0 0

0 0 0 0 0 0 0 0 0 0 0 0 0
0 0 0 0 0 0 0 0 0
"Viruses;Uroviricota;Caudoviricetes;Caudoviricetes__na;Caudoviricetes__na;Efquatrovirus;Efquatrovirus__uc"
0 0 0 0 0 0 0 0 0 0 0 0 0
0 0 0 0 0 0 0 0 0 0 0 0 0
0 0 0 0 0 0 0 0 0 0 0 0 0
0 0 0 0 0 0 0 0 1 0 0 0 0
0 0 0 0 0 0 0 0 0 0 0 0 0
0 0 0 0 0 0 0
"Viruses;Uroviricota;Caudoviricetes;Caudoviricetes__na;Caudoviricetes__na;Efquatrovirus;Efquatrovirus_AL3"
0 0 1 0 0 0 0 0 0 0 0 0 0
0 0 0 0 0 0 0 0 0 0 0 0 0
0 0 0 0 0 0 0 0 0 0 0 0 0
0 0 0 0 0 0 0 0 0 0 0 0 0
0 0 0 0 0 0 0 0 0 0 0 0 0
0 0 0 0 0 0 0
"Viruses;Uroviricota;Caudoviricetes;Caudoviricetes__na;Caudoviricetes__na;Eiauvirus;Eiauvirus_eiAU" 0
0 2 0 0 0 0 0 0 0 0 0 0 0
0 0 0 0 0 0 0 0 0 0 0 0 0
0 0 0 0 0 0 0 0 0 0 0 0 0
0 0 0 0 0 0 0 0 0 0 0 0 0
0 0 0 0 0 0 0 0 0 0 0 0 0
0 0 0 0 0 0
"Viruses;Uroviricota;Caudoviricetes;Caudoviricetes__na;Caudoviricetes__na;Eisenstarkvirus;Eisenstarkvirus_L7
" 0 0 0 1 0 0 0 0 0 0 0 0 0
0 0 0 0 0 0 0 0 0 0 0 0 0
0 0 0 0 0 0 0 0 0 0 0 0 0
0 0 0 0 0 0 0 0 0 0 0 0 0
0 0 0 0 0 0 0 0 0 0 0 0 0
0 0 0 0 0 0 0
"Viruses;Uroviricota;Caudoviricetes;Caudoviricetes__na;Caudoviricetes__na;Elemovirus;Elemovirus__uc"
0 0 0 1 1 6 0 0 0 0 0 0 0
0 0 0 0 1 0 0 0 0 0 0 0 0
0 0 0 0 0 0 0 0 0 0 0 0 0
0 0 0 0 0 0 0 0 2 0 0 0 0
0 0 0 0 0 0 0 0 0 0 0 0 0
0 0 0 0 0 0 0
"Viruses;Uroviricota;Caudoviricetes;Caudoviricetes__na;Caudoviricetes__na;Elmenteitavirus;Elmenteitavirus_e
v125" 0 0 0 1 0 0 0 0 0 0 0 0 0
0 0 0 0 0 0 0 0 0 0 0 0 0
0 0 0 0 0 0 0 0 0 0 0 0 0
0 0 0 0 0 0 0 0 0 0 0 0 0
0 0 0 0 0 0 0 0 0 0 0 0 0
0 0 0 0 0 0 0
"Viruses;Uroviricota;Caudoviricetes;Caudoviricetes__na;Caudoviricetes__na;Emalynvirus;Gordonia_phage_Gi
KK" 0 0 0 0 0 0 0 0 0 0 0 0 0
0 0 0 0 0 0 0 0 0 0 0 0 0
0 0 0 0 0 0 0 0 0 0 0 0 0
0 0 0 0 0 0 0 0 0 0 0 0 0

0 0 0 1 1 0 0 0 0 0 0 0 0
0 0 0 0 0 0 0
"Viruses;Uroviricota;Caudoviricetes;Caudoviricetes__na;Caudoviricetes__na;Emdodecavirus;Emdodecavirus__uc" 0 0 0 0 0 0 0 0 0 0 0 0 0
0 0 0 0 0 0 0 0 0 0 0 0 0
0 0 0 0 0 0 0 0 0 0 0 0 0
0 0 0 0 0 0 0 0 5 0 0 0 0
0 0 0 0 0 0 0 0 0 0 0 0 0
0 0 0 0 0 0 0
"Viruses;Uroviricota;Caudoviricetes;Caudoviricetes__na;Caudoviricetes__na;Eurybiavirus;Eurybiavirus_PHM2"
0 0 0 0 1 0 0 0 0 0 0 0 0
0 0 0 0 0 0 0 0 0 0 0 0 0
0 0 0 0 0 0 0 0 0 0 0 0 0
0 0 0 0 0 0 0 0 0 0 0 0 0
0 0 0 0 0 0 0 0 0 0 0 0 0
0 0 0 0 0 0 0
"Viruses;Uroviricota;Caudoviricetes;Caudoviricetes__na;Caudoviricetes__na;Felixounavirus;Felixounavirus__uc" 0 4 0 0 0 0 0 0 0 0 0 0 0
0 0 0 0 0 0 0 0 0 0 0 0 0
0 0 0 0 0 0 0 0 0 0 0 0 0
0 0 0 0 0 0 0 53 0 0 0 0 0
0 0 0 0 0 0 0 0 0 0 0 0 0
0 0 0 0 1 0 0
"Viruses;Uroviricota;Caudoviricetes;Caudoviricetes__na;Caudoviricetes__na;Feofaniavirus;Feofaniavirus_Eho59" 0 0 1 0 0 0 0 0 0 0 0 0 0
0 0 0 0 0 0 0 0 0 0 0 0 0
0 0 0 0 0 0 0 0 0 0 0 0 0
0 0 0 0 0 0 0 0 0 0 0 0 0
0 0 0 0 0 0 0 0 0 0 0 0 0
0 0 0 0 0 0 0
"Viruses;Uroviricota;Caudoviricetes;Caudoviricetes__na;Caudoviricetes__na;Ficleduovirus;Ficleduovirus__uc"
0 0 0 2 2 8 0 0 0 0 0 0 0
0 0 0 0 0 0 0 0 0 0 0 0 0
0 0 0 1 0 0 0 0 0 0 0 0 0
0 0 0 0 0 0 0 0 0 0 0 0 0
0 0 0 0 0 0 0 0 0 0 0 0 0
0 0 0 0 0 1 0
"Viruses;Uroviricota;Caudoviricetes;Caudoviricetes__na;Caudoviricetes__na;Ficleduovirus;Ficleduovirus_FCL2" 0 0 0 0 1 0 0 0 0 0 0 0 0
0 0 0 0 0 0 0 0 0 0 0 0 0
0 0 0 0 0 0 0 0 0 0 0 0 0
0 0 0 0 0 0 0 0 0 0 0 0 0
0 0 0 0 0 0 0 0 0 0 0 0 0
0 0 0 0 0 0 0
"Viruses;Uroviricota;Caudoviricetes;Caudoviricetes__na;Caudoviricetes__na;Ficleduovirus;Flavobacterium_phage_FL-1" 0 0 0 1 94 4 0 0 0 0 0 0
0 0 0 0 0 2 0 0 0 0 0 0 0
0 1 0 0 0 0 0 0 0 0 0 0 0
0 0 0 0 0 0 0 0 0 2 0 0 0

0 0 0 0 0 0 0 0 0 0 0 0 0
0 0 0 0 0 0 4 0
"Viruses;Uroviricota;Caudoviricetes;Caudoviricetes__na;Caudoviricetes__na;Fipvunavirus;Fipvunavirus__uc"
1 1 0 1 2 0 0 0 0 0 0 0 0
0 0 0 0 0 0 0 0 0 0 0 0 0
0 0 0 0 0 0 0 0 0 0 0 0 0
0 0 0 0 0 0 0 0 5 0 0 0 0
0 0 0 0 0 0 0 0 0 0 0 0 0
0 0 0 0 0 1 0
"Viruses;Uroviricota;Caudoviricetes;Caudoviricetes__na;Caudoviricetes__na;Fipvunavirus;Fipvunavirus_Fpv4"
0 0 0 1 0 2 0 0 0 0 0 0 0
0 0 0 0 0 0 0 0 0 0 0 0 0
0 0 0 0 0 0 0 0 0 0 0 0 0
0 0 0 0 0 0 0 0 0 0 0 0 0
0 0 0 0 0 0 0 0 0 0 0 0 0
0 0 0 0 0 0 0
"Viruses;Uroviricota;Caudoviricetes;Caudoviricetes__na;Caudoviricetes__na;Firingavirus;Firingavirus_RSK1"
0 0 0 1 0 0 0 0 0 0 0 0 0
0 0 0 0 0 0 0 0 0 0 0 0 0
0 0 0 0 0 0 0 0 0 0 0 0 0
0 0 0 0 0 0 0 0 0 0 0 0 0
0 0 0 0 0 0 0 0 0 0 0 0 0
0 0 0 0 0 0 0
"Viruses;Uroviricota;Caudoviricetes;Caudoviricetes__na;Caudoviricetes__na;Fishburnevirus;Fishburnevirus__u
c" 0 0 0 1 0 0 0 0 0 0 0 0 0
0 0 0 0 0 0 0 0 0 0 0 0 0
0 0 0 0 0 0 0 0 0 0 0 0 0
0 0 0 0 0 0 0 0 0 0 0 0 0
0 0 0 0 0 0 0 0 0 0 0 0 0
0 0 0 0 0 0 0
"Viruses;Uroviricota;Caudoviricetes;Caudoviricetes__na;Caudoviricetes__na;Fletchervirus;Fletchervirus__uc"
0 0 0 0 0 3 0 0 0 0 0 0 0
0 0 0 0 0 0 0 0 0 0 0 0 0
0 0 0 0 0 0 0 0 0 0 0 0 0
0 0 0 0 0 0 0 0 0 0 0 0 0
0 0 0 0 0 0 0 0 0 0 0 0 0
0 0 0 0 0 0 0
"Viruses;Uroviricota;Caudoviricetes;Caudoviricetes__na;Caudoviricetes__na;Fromanvirus;Fromanvirus__uc"
0 0 2 1 0 0 0 0 0 0 0 0 0
0 0 0 0 0 0 0 0 0 0 0 0 0
0 0 0 0 0 0 0 0 1 0 0 0 0
0 0 0 0 0 0 0 0 0 0 0 0 0
0 0 0 0 0 0 0 0 0 0 0 0 0
0 0 0 0 0 0 0
"Viruses;Uroviricota;Caudoviricetes;Caudoviricetes__na;Caudoviricetes__na;Fromanvirus;Mycobacterium_pha
ge_Makemake" 0 0 0 0 1 0 0 0 0 0 0 0
0 0 0 0 0 0 0 0 0 0 0 0 0
0 0 0 0 0 0 0 0 0 0 0 0 0
0 0 0 0 0 0 0 0 0 0 0 0 0

0 0 0 0 0 0 0 0 0 0 0 0 0
0 0 0 0 0 0 0 0
"Viruses;Uroviricota;Caudoviricetes;Caudoviricetes__na;Caudoviricetes__na;Gaiavirus;Gaiavirus__uc" 0
0 1 2 13 1 0 0 0 0 0 0 0 0
0 0 0 0 0 0 0 0 0 0 0 0 0
0 0 0 0 0 0 0 0 0 0 0 0 0
0 0 0 0 0 0 0 3 0 0 0 0 0
0 0 0 0 0 0 0 0 0 0 0 0 0
0 0 0 0 2 0
"Viruses;Uroviricota;Caudoviricetes;Caudoviricetes__na;Caudoviricetes__na;Gaiavirus;Gaiavirus_gaia" 0
0 0 0 0 0 0 0 0 0 0 0 0 0
0 0 0 0 0 0 0 0 0 0 0 0 0
0 0 0 0 0 0 0 0 0 0 0 0 0
0 1 0 0 0 0 0 0 0 0 0 0 0
0 0 0 0 0 0 0 0 0 0 0 0 0
0 0 0 0 0 0
"Viruses;Uroviricota;Caudoviricetes;Caudoviricetes__na;Caudoviricetes__na;Galaxyvirus;Galaxyvirus_galaxy"
0 0 0 0 0 0 0 0 0 0 0 0 0
0 0 0 0 0 0 0 0 0 0 0 0 0
0 0 0 0 0 0 0 0 1 0 0 0 0
0 0 0 0 0 0 0 0 0 0 0 0 0
0 0 0 0 0 0 0 0 0 0 0 0 0
0 0 0 0 0 0 0
"Viruses;Uroviricota;Caudoviricetes;Caudoviricetes__na;Caudoviricetes__na;Gervaisevirus;Gervaisevirus__uc"
0 0 0 2 0 0 0 0 0 0 0 0 0
0 0 0 0 0 0 0 0 0 0 0 0 0
0 0 0 0 0 0 0 0 0 0 0 0 0
0 0 0 0 0 0 0 0 0 0 0 0 0
0 0 0 0 0 0 0 0 0 0 0 0 0
0 0 0 0 0 0 0
"Viruses;Uroviricota;Caudoviricetes;Caudoviricetes__na;Caudoviricetes__na;Gervaisevirus;Ralstonia_phage_p2
110" 0 0 0 0 1 0 0 0 0 0 0 0 0
0 0 0 0 0 0 0 0 0 0 0 0 0
0 0 0 0 0 0 0 0 0 0 0 0 0
0 0 0 0 0 0 0 0 0 0 0 0 0
0 0 0 0 0 0 0 0 0 0 0 0 0
0 0 0 0 0 0 0
"Viruses;Uroviricota;Caudoviricetes;Caudoviricetes__na;Caudoviricetes__na;Gervaisevirus;Ralstonia_phage_RP
ZH3" 0 0 0 0 1 0 0 0 0 0 0 0 0
0 0 0 0 0 0 0 0 0 0 0 0 0
0 0 0 0 0 0 0 0 0 0 0 0 0
0 0 0 0 0 0 0 0 0 0 0 0 0
0 0 0 0 0 0 0 0 0 0 0 0 0
0 0 0 0 0 0 0
"Viruses;Uroviricota;Caudoviricetes;Caudoviricetes__na;Caudoviricetes__na;Gilsonvirus;Streptomyces_phage_
Moab" 0 0 0 0 0 1 0 0 0 0 0 0 0
0 0 0 0 0 0 0 0 0 0 0 0 0
0 0 0 0 0 0 0 0 0 0 0 0 0
0 0 0 0 0 0 0 0 0 0 0 0 0

0 0 0 0 0 0 0 0 0 0 0 0 0
0 0 0 0 0 0 0
"Viruses;Uroviricota;Caudoviricetes;Caudoviricetes__na;Caudoviricetes__na;Godonkavirus;Godonkavirus_god
onK" 0 0 0 1 0 6 0 0 0 0 0 0 0
0 0 0 0 0 0 0 0 0 0 0 0 0
0 0 0 0 0 0 0 0 0 0 0 0 0
0 0 0 0 0 0 0 0 0 0 0 0 0
0 0 0 0 0 0 0 0 0 0 0 0 0
0 0 0 0 0 0 0
"Viruses;Uroviricota;Caudoviricetes;Caudoviricetes__na;Caudoviricetes__na;Gordonvirus;Gordonvirus__uc"
0 0 0 0 9 0 0 0 0 0 0 0 0
0 0 0 0 0 0 0 0 0 0 0 0 1
0 0 0 0 0 0 0 0 0 0 0 0 0
0 0 0 0 0 0 0 0 0 0 0 0 0
1 0 0 0 0 0 0 0 0 0 0 0 0
0 0 0 0 0 0 0
"Viruses;Uroviricota;Caudoviricetes;Caudoviricetes__na;Caudoviricetes__na;Harrisonburgvirus;Harrisonburgvi
rus_persinger" 0 0 0 0 0 2 0 0 0 0 0 0
0 0 0 0 0 0 0 0 0 0 1 0 0
0 0 0 0 0 0 0 0 0 0 0 0 0
0 0 0 0 0 0 0 0 0 0 0 0 0
0 0 0 0 0 0 0 0 0 0 0 0 0
0 0 0 0 0 0 0 0
"Viruses;Uroviricota;Caudoviricetes;Caudoviricetes__na;Caudoviricetes__na;Hattifnattvirus;Hattifnattvirus_hat
tifnatt" 0 0 0 0 0 0 0 0 0 0 0 0 0
0 0 0 0 0 0 0 0 0 0 0 0 0
0 0 0 0 0 0 0 0 0 0 0 0 0
0 0 0 0 0 1 0 0 0 0 0 0 0
0 0 0 0 0 0 0 0 0 0 0 0 0
0 0 0 0 0 1 0
"Viruses;Uroviricota;Caudoviricetes;Caudoviricetes__na;Caudoviricetes__na;Helsingorvirus;Helsingorvirus_Cb
a181" 0 0 1 1 0 0 0 0 0 0 0 0 0
0 0 0 0 0 0 0 0 0 0 0 0 0
0 0 0 0 0 0 0 0 0 0 0 0 0
0 0 0 0 0 0 0 0 0 0 0 0 0
0 0 0 0 0 0 0 0 0 0 0 0 0
0 0 0 0 0 0 0
"Viruses;Uroviricota;Caudoviricetes;Caudoviricetes__na;Caudoviricetes__na;Immutovirus;Immutovirus__uc"
0 1 1 7 99 17 0 0 0 0 0 0 0
0 0 2 0 0 0 0 0 0 0 0 0 0
0 0 0 0 0 0 0 0 0 3 0 0 0
0 0 0 0 0 0 0 0 12 0 0 0 0
0 0 0 0 0 0 0 45 0 0 0 0 0
0 0 0 0 0 3 0
"Viruses;Uroviricota;Caudoviricetes;Caudoviricetes__na;Caudoviricetes__na;Immutovirus;Immutovirus_immut
o" 0 1 0 3 7 10 0 0 0 0 0 0 0
0 0 0 0 0 0 0 0 0 0 1 0 0
0 0 0 0 0 0 0 0 0 0 0 0 0
0 0 0 0 0 0 0 0 4 0 0 0 0

0 0 0 0 0 0 0 0 0 0 0 0 0
0 0 0 0 0 0 0
"Viruses;Uroviricota;Caudoviricetes;Caudoviricetes__na;Caudoviricetes__na;Incheonvirus;Incheonvirus_P12002
L" 0 0 1 0 4 0 0 0 0 0 0 0 0
0 0 0 0 0 0 0 0 0 0 0 0 0
0 0 0 0 0 0 0 0 0 0 0 0 0
0 0 0 0 0 0 0 0 0 0 0 0 0
0 0 0 0 0 0 0 0 0 0 0 0 0
0 0 0 0 0 0 0
"Viruses;Uroviricota;Caudoviricetes;Caudoviricetes__na;Caudoviricetes__na;Incheonvirus;Incheonvirus_P12002
S" 0 0 0 0 0 2 0 0 0 0 0 0 0
0 0 0 0 0 0 0 0 0 0 0 0 0
0 0 0 0 0 0 0 0 0 0 0 0 0
0 0 0 0 0 0 0 0 0 0 0 0 0
0 0 0 0 0 0 0 0 0 0 0 0 0
0 0 0 0 0 0 0
"Viruses;Uroviricota;Caudoviricetes;Caudoviricetes__na;Caudoviricetes__na;Iodovirus;Iodovirus_PLPE" 0
0 0 1 0 5 0 0 0 0 0 0 0 0
0 0 0 0 0 0 0 0 0 0 0 0 0
0 0 0 0 0 0 0 0 0 0 0 0 0
0 0 0 0 0 0 0 0 0 0 0 0 0
0 0 0 0 0 0 0 0 0 0 0 0 0
0 0 0 0 0 0
"Viruses;Uroviricota;Caudoviricetes;Caudoviricetes__na;Caudoviricetes__na;Ionavirus;Ionavirus_W14" 0
0 0 0 0 3 0 0 0 0 0 0 0 0
0 0 0 0 0 0 0 0 0 0 0 0 0
0 0 0 0 0 0 0 0 0 0 0 0 0
0 0 0 0 0 0 0 0 0 0 0 0 0
0 0 0 0 0 0 0 0 0 0 0 0 0
0 0 0 0 0 0
"Viruses;Uroviricota;Caudoviricetes;Caudoviricetes__na;Caudoviricetes__na;Jamesmcgillvirus;Jamesmcgillvirus
__uc" 0 0 0 0 0 0 0 0 0 0 0 0 0
0 0 0 0 0 0 0 0 0 0 0 0 0
0 0 0 0 0 0 0 0 0 2 0 0 0
0 0 0 0 0 0 0 0 0 0 0 0 0
0 0 0 0 0 0 0 0 0 0 0 0 0
0 0 0 0 0 0 0
"Viruses;Uroviricota;Caudoviricetes;Caudoviricetes__na;Caudoviricetes__na;Jedunavirus;Klebsiella_phage_BU
CT_49532" 0 0 1 0 0 0 0 0 0 0 0 0
0 0 0 0 0 0 0 0 0 0 0 0 0
0 0 0 0 0 0 0 0 0 0 0 0 0
0 0 0 0 0 0 0 0 0 0 0 0 0
0 0 0 0 0 0 0 0 0 0 0 0 0
0 0 0 0 0 0 0 0
"Viruses;Uroviricota;Caudoviricetes;Caudoviricetes__na;Caudoviricetes__na;Jedunavirus;Klebsiella_phage_vB_
KpnM_15-38_KLPPOU148" 0 0 0 0 0 0 0 0 0 0
0 0 0 0 0 0 0 0 0 0 0 0 0
0 0 0 0 0 0 0 0 0 0 0 0 0
0 0 0 0 0 0 0 0 0 0 23 0 0

0 0 0 0 0 0 0 0 0 0 0 0 0
0 0 0 0 0 0 0 0 0 0
"Viruses;Uroviricota;Caudoviricetes;Caudoviricetes__na;Caudoviricetes__na;Jedunavirus;Klebsiella_phage_VL
CpiM12a" 0 0 0 0 0 0 0 0 0 0 0 0
0 0 0 0 0 0 0 0 0 0 0 0 0
0 0 0 0 0 0 0 0 0 0 0 0 0
0 0 1 0 0 0 0 0 0 0 0 0 0
0 0 0 0 0 0 0 0 0 0 0 0 0
0 0 0 0 0 0 0 0
"Viruses;Uroviricota;Caudoviricetes;Caudoviricetes__na;Caudoviricetes__na;Jerseyvirus;Jerseyvirus__uc"0
1 0 0 0 0 0 0 0 0 0 0 0 0
0 0 0 0 0 0 0 0 0 0 0 0 1
0 0 0 0 0 1 0 0 0 0 1 0 0
0 0 0 0 0 1 10 0 0 0 0 0 0
0 0 0 0 0 0 0 0 0 0 0 0 0
0 0 0 0 0 0
"Viruses;Uroviricota;Caudoviricetes;Caudoviricetes__na;Caudoviricetes__na;Jerseyvirus;Salmonella_phage_SeF
1" 1 1 0 0 0 0 0 0 0 0 0 0 0
0 0 0 0 0 0 0 0 0 0 0 0 0
0 0 0 0 0 0 0 0 0 0 0 0 0
0 0 0 0 0 0 2 0 0 0 0 0 0
0 0 0 0 0 0 0 0 0 0 0 0 0
0 0 0 0 0 0 0
"Viruses;Uroviricota;Caudoviricetes;Caudoviricetes__na;Caudoviricetes__na;Jouyvirus;Escherichia_phage_21"
0 0 0 0 0 0 0 0 0 0 0 0 0
0 0 0 0 0 0 0 0 0 0 0 0 0
0 0 0 0 0 0 0 0 0 0 0 0 0
0 0 0 0 0 0 0 0 0 0 0 0 0
0 0 0 0 0 0 0 0 7 0 0 0 0
0 0 0 0 0 0 0
"Viruses;Uroviricota;Caudoviricetes;Caudoviricetes__na;Caudoviricetes__na;Jouyvirus;Jouyvirus_ev207" 0
0 0 0 0 0 0 0 0 0 0 0 0 0
0 0 0 0 0 0 0 0 0 0 0 0 0
0 0 0 0 0 0 0 0 0 0 0 0 0
0 0 0 0 0 0 0 0 0 0 0 0 0
0 0 0 0 0 0 0 3 0 0 0 0 0
0 0 0 0 0 0
"Viruses;Uroviricota;Caudoviricetes;Caudoviricetes__na;Caudoviricetes__na;Julieunavirus;Julieunavirus__uc"
0 0 0 0 0 0 0 0 0 0 0 0 0
0 0 0 0 0 0 0 0 0 0 0 0 0
0 0 0 0 0 0 0 0 0 0 0 0 0
0 0 0 0 0 0 0 0 0 0 0 0 0
0 0 0 0 0 0 0 24 0 0 0 0 0
0 0 0 0 0 0 0
"Viruses;Uroviricota;Caudoviricetes;Caudoviricetes__na;Caudoviricetes__na;Karimacvirus;Streptomyces_phage
_Tomas" 0 0 0 0 0 0 0 0 0 0 0 0
0 0 0 0 0 0 0 0 0 0 0 0 0
0 0 0 0 0 0 1 0 0 0 0 0 0
0 0 0 0 0 0 0 0 0 0 0 0 0

0 0 0 0 0 0 0 0 0 0 0 0 0
0 0 0 0 0 0 0 0
"Viruses;Uroviricota;Caudoviricetes;Caudoviricetes__na;Caudoviricetes__na;Kelquatrovirus;Kelquatrovirus_KL
4" 0 0 0 1 0 0 0 0 0 0 0 0 0
0 0 0 0 0 0 0 0 0 0 0 0 0
0 0 0 0 0 0 0 0 0 0 0 0 0
0 0 0 0 0 0 0 0 0 0 0 0 0
0 0 0 0 0 0 0 0 0 0 0 0 0
0 0 0 0 0 0 0
"Viruses;Uroviricota;Caudoviricetes;Caudoviricetes__na;Caudoviricetes__na;Kenoshavirus;Kenoshavirus_ohmy
ward" 0 0 0 0 4 0 0 0 0 0 0 0 0
0 0 0 0 0 0 0 0 0 0 0 0 0
0 0 0 0 0 0 0 0 0 0 0 0 0
0 0 0 0 0 0 0 0 0 0 0 0 0
0 0 0 0 0 0 0 0 0 0 0 0 0
0 0 0 0 0 0 0
"Viruses;Uroviricota;Caudoviricetes;Caudoviricetes__na;Caudoviricetes__na;Kilunavirus;Kilunavirus_KL1"
0 0 0 0 0 2 0 0 0 0 0 0 0
0 0 0 0 0 0 0 0 0 0 0 0 0
0 0 0 0 0 0 0 0 0 0 0 0 0
0 0 0 0 0 0 0 0 0 0 0 0 0
0 0 0 0 0 0 0 0 0 0 0 0 0
0 0 0 0 0 0 0
"Viruses;Uroviricota;Caudoviricetes;Caudoviricetes__na;Caudoviricetes__na;Kleczkowskavirus;Kleczkowskavir
us__uc" 0 1 0 1 0 0 0 0 0 0 0 0 0
0 0 0 0 0 0 0 0 0 0 0 0 0
0 0 0 0 0 0 0 0 0 0 0 0 0
0 0 0 0 0 0 0 0 0 0 0 0 0
0 0 0 0 0 0 0 0 0 0 0 0 0
0 0 0 0 0 0 0
"Viruses;Uroviricota;Caudoviricetes;Caudoviricetes__na;Caudoviricetes__na;Kolesnikvirus;Erwinia_phage_phi
Ea116" 0 0 0 0 0 0 0 0 0 0 0 0 0
0 0 0 0 0 0 0 0 0 0 0 0 0
0 0 0 0 0 0 0 0 0 0 0 0 0
0 0 1 0 0 0 0 0 0 0 0 0 0
0 0 0 0 0 0 0 0 0 0 0 0 0
0 0 0 0 0 0 0
"Viruses;Uroviricota;Caudoviricetes;Caudoviricetes__na;Caudoviricetes__na;Kolesnikvirus;Erwinia_phage_Ros
cha1" 0 0 0 0 0 0 0 0 0 0 0 0 0
0 0 0 0 0 0 0 0 0 0 0 0 0
0 0 0 0 0 0 0 0 0 1 0 0 0
0 0 2 0 0 0 0 0 0 0 0 0 0
0 0 0 0 0 0 0 0 0 0 0 0 0
0 0 0 0 0 0 0
"Viruses;Uroviricota;Caudoviricetes;Caudoviricetes__na;Caudoviricetes__na;Kolesnikvirus;Kolesnikvirus__uc"
0 0 5 0 0 0 0 0 0 0 0 0 0
0 0 0 0 0 0 0 0 0 0 0 0 0
1 0 0 0 0 0 0 0 0 36 0 0 0
0 0 40 0 0 0 0 33 0 0 0 0 0

0 2 0 0 0 0 0 19 0 0 0 0 0
0 0 0 0 0 0 0
"Viruses;Uroviricota;Caudoviricetes;Caudoviricetes__na;Caudoviricetes__na;Kolesnikvirus;Kolesnikvirus_M7"
0 0 1 0 0 0 0 0 0 0 0 0 0
0 0 0 0 0 0 0 0 0 0 0 0 0
0 0 0 0 0 0 0 0 0 11 0 0 0
0 0 8 0 0 0 0 13 1 0 0 0 0
0 0 0 0 0 0 0 0 0 0 0 0 0
0 0 0 0 0 0 0
"Viruses;Uroviricota;Caudoviricetes;Caudoviricetes__na;Caudoviricetes__na;Korravirus;Korravirus__uc" 0
0 0 0 0 0 0 0 0 0 0 0 0 0
0 0 0 0 0 0 0 0 0 0 0 0 0
0 0 0 0 0 0 0 0 2 0 0 0 0
0 0 0 0 0 0 0 0 0 0 0 0 0
0 0 0 0 0 0 0 0 0 0 0 0 0
0 0 0 0 0 0
"Viruses;Uroviricota;Caudoviricetes;Caudoviricetes__na;Caudoviricetes__na;Kostyavirus;Kostyavirus__uc"
0 0 0 1 0 0 0 0 0 0 0 0 0
0 0 0 0 0 0 0 0 0 0 0 0 0
0 0 0 0 0 0 0 0 0 0 0 0 0
0 0 0 0 0 0 0 0 0 0 0 0 0
0 0 0 0 0 0 0 0 0 0 0 0 0
0 0 0 0 0 0 0
"Viruses;Uroviricota;Caudoviricetes;Caudoviricetes__na;Caudoviricetes__na;Kostyavirus;Mycobacterium_phag
e_Lilac" 0 0 0 0 12 0 0 0 0 0 0 0 0
0 0 0 0 0 0 0 0 0 0 0 0 0
0 0 0 0 0 0 0 0 0 0 0 0 0
0 0 0 0 0 0 0 0 0 0 0 0 0
0 0 0 0 0 0 0 0 0 0 0 0 0
0 0 0 0 0 0 0
"Viruses;Uroviricota;Caudoviricetes;Caudoviricetes__na;Caudoviricetes__na;Krylovvirus;Krylovvirus_tf" 0
0 0 0 5 0 0 0 0 0 0 0 0 0
0 0 0 0 0 0 0 0 0 0 0 0 0
0 0 0 0 0 0 0 0 0 0 0 0 0
0 0 0 0 0 0 0 0 0 0 0 0 0
0 0 0 0 0 0 0 0 0 0 0 0 0
0 0 0 0 0 0
"Viruses;Uroviricota;Caudoviricetes;Caudoviricetes__na;Caudoviricetes__na;Kunmingvirus;Kunmingvirus__uc"
0 0 0 0 2 6 0 0 0 0 0 0 0
0 0 0 0 0 0 0 0 0 0 0 0 0
0 0 0 0 0 0 0 0 0 0 0 0 0
0 0 0 0 0 0 0 0 0 0 0 0 0
0 0 0 0 0 0 0 0 0 0 0 0 0
0 0 0 0 0 0 0
"Viruses;Uroviricota;Caudoviricetes;Caudoviricetes__na;Caudoviricetes__na;Kunmingvirus;Kunmingvirus_kv4
D05" 0 0 0 1 0 4 0 0 0 0 0 0 0
0 0 0 0 0 0 0 0 0 0 0 0 0
0 0 0 0 0 0 0 0 0 0 0 0 0
0 0 0 0 0 0 0 0 0 0 0 0 0

0 0 0 0 0 0 0 0 0 0 0 0 0
0 0 0 0 0 0 0
"Viruses;Uroviricota;Caudoviricetes;Caudoviricetes__na;Caudoviricetes__na;Kuravirus;Kuravirus__uc" 0
0 0 0 0 0 0 0 0 0 0 0 0 0
0 0 0 0 0 0 0 0 0 0 0 0 0
0 0 0 0 0 0 0 0 0 0 0 0 0
0 0 0 0 1 0 0 0 0 0 0 0 0
0 0 0 0 0 0 0 0 0 0 0 0 0
0 0 0 0 0 0
"Viruses;Uroviricota;Caudoviricetes;Caudoviricetes__na;Caudoviricetes__na;Lacusarxvirus;Lacusarxvirus_lacus
arx" 0 0 0 0 1 0 0 0 0 0 0 0 0
0 0 0 0 0 0 0 0 0 0 0 0 0
0 0 0 0 0 0 0 0 0 0 0 0 0
0 0 0 0 0 0 0 0 1 0 0 0 0
0 0 0 0 0 0 0 0 0 0 0 0 0
0 0 0 0 0 0 0
"Viruses;Uroviricota;Caudoviricetes;Caudoviricetes__na;Caudoviricetes__na;Lahexavirus;Lahexavirus__uc"
0 0 0 0 27 5 0 0 0 0 0 0 0
0 0 0 0 0 0 0 0 0 0 0 0 0
0 0 0 0 0 0 0 0 0 2 0 0 0
0 0 0 0 0 0 0 0 0 0 0 0 0
0 0 0 0 0 0 0 0 0 0 0 0 0
0 0 0 0 0 0 0
"Viruses;Uroviricota;Caudoviricetes;Caudoviricetes__na;Caudoviricetes__na;Lambdavirus;Enterobacteria_phag
e_LC159" 0 0 0 0 0 0 0 0 0 0 0 0
0 0 0 0 0 0 0 0 0 0 0 0 0
0 0 0 0 0 0 0 0 0 0 0 0 0
0 0 0 0 0 0 0 0 16 0 0 0 0
0 0 0 0 0 0 0 0 0 0 0 0 0
0 0 0 0 0 0 0 0
"Viruses;Uroviricota;Caudoviricetes;Caudoviricetes__na;Caudoviricetes__na;Lambdavirus;Lambdavirus_lambd
a" 0 0 0 0 0 0 0 0 0 0 0 0 0
0 0 0 0 0 0 0 0 0 0 0 0 0
0 0 0 0 0 1 0 0 0 0 0 0 0
0 0 0 0 0 0 0 0 0 0 0 0 0
0 0 0 0 0 0 0 0 0 0 0 0 0
0 0 0 0 0 0 0
"Viruses;Uroviricota;Caudoviricetes;Caudoviricetes__na;Caudoviricetes__na;Leonardvirus;Leonardvirus__uc"
0 0 0 1 0 0 0 0 0 0 0 0 0
0 0 0 0 0 0 0 0 0 0 0 0 0
0 0 0 0 0 0 0 0 0 0 0 0 0
0 0 0 0 0 0 0 0 0 0 0 0 0
0 0 0 0 0 0 0 0 0 0 0 0 0
0 0 0 0 0 0 0
"Viruses;Uroviricota;Caudoviricetes;Caudoviricetes__na;Caudoviricetes__na;Lessievirus;Burkholderia_phage_C
SP3" 0 0 0 5 50 4 0 0 0 0 0 0 0
0 0 0 0 0 0 0 0 0 0 0 0 0
0 0 0 0 0 0 0 0 0 0 0 0 0
0 0 0 0 0 0 0 0 2 0 0 0 0

0 0 0 0 0 0 0 0 0 0 0 0 0
0 0 0 0 0 0 0
"Viruses;Uroviricota;Caudoviricetes;Caudoviricetes__na;Caudoviricetes__na;Lessievirus;Burkholderia_phage_J
C1" 0 0 0 0 15 0 0 0 0 0 0 0 0
0 0 0 0 0 0 0 0 0 0 0 0 0
0 0 0 0 0 0 0 0 0 0 0 0 0
0 0 0 0 0 0 0 0 0 0 0 0 0
0 0 0 0 0 0 0 0 0 0 0 0 0
0 0 0 0 0 0 0
"Viruses;Uroviricota;Caudoviricetes;Caudoviricetes__na;Caudoviricetes__na;Lessievirus;Lessievirus__uc"
0 0 0 0 48 3 0 0 0 0 0 0 0
0 0 0 0 0 0 0 0 0 0 0 0 0
0 0 0 0 0 0 0 0 0 0 0 0 0
0 0 0 0 0 0 0 0 0 0 0 0 0
0 0 0 0 0 0 0 0 0 0 0 0 0
0 0 0 0 0 1 0
"Viruses;Uroviricota;Caudoviricetes;Caudoviricetes__na;Caudoviricetes__na;Lessievirus;Lessievirus_bcepil02"
0 0 0 0 0 0 0 0 0 0 0 0 0
0 0 0 0 2 0 0 0 0 0 0 0 0
0 0 0 0 0 0 0 0 0 0 0 0 0
0 0 0 0 0 0 0 0 0 0 0 0 0
0 0 0 0 0 0 0 0 0 0 0 0 0
0 0 0 0 0 0 0
"Viruses;Uroviricota;Caudoviricetes;Caudoviricetes__na;Caudoviricetes__na;Lessievirus;Lessievirus_DC1"
0 0 0 0 1 0 0 0 0 0 0 0 0
0 0 0 0 0 0 0 0 0 0 0 0 0
0 0 0 0 0 0 0 0 0 0 0 0 0
0 0 0 0 0 0 0 0 0 0 0 0 0
0 0 0 0 0 0 0 0 0 0 0 0 0
0 0 0 0 0 0 0
"Viruses;Uroviricota;Caudoviricetes;Caudoviricetes__na;Caudoviricetes__na;Lightbulbvirus;Lightbulbvirus_Cb
a172" 0 0 0 0 0 4 0 0 0 0 0 0 0
0 0 0 0 0 0 0 0 0 0 0 0 0
0 0 0 0 0 0 0 0 0 0 0 0 0
0 0 0 0 0 0 0 0 0 0 0 0 0
0 0 0 0 0 0 0 0 0 0 0 0 0
0 0 0 0 0 0 0
"Viruses;Uroviricota;Caudoviricetes;Caudoviricetes__na;Caudoviricetes__na;Lightbulbvirus;Lightbulbvirus_Cb
a41" 0 0 0 0 0 0 0 0 0 0 0 0 0
0 0 0 0 0 0 0 0 0 0 0 0 0
0 0 0 0 0 0 0 0 0 0 0 0 0
0 0 0 0 0 0 0 0 0 0 0 0 3
0 0 0 0 0 0 0 0 0 0 0 0 0
0 0 0 0 0 0 0
"Viruses;Uroviricota;Caudoviricetes;Caudoviricetes__na;Caudoviricetes__na;Likavirus;Likavirus__uc" 0
0 0 0 0 0 0 0 0 0 0 0 0 0
0 0 0 0 0 0 0 0 0 0 0 0 0
0 0 0 0 0 0 0 0 0 0 0 0 0
0 1 0 0 0 0 0 0 0 0 0 0 0

0 0 0 0 0 0 0 0 0 0 0 0 0
0 0 0 0 0 0
"Viruses;Uroviricota;Caudoviricetes;Caudoviricetes__na;Caudoviricetes__na;Lillamyvirus;Lillamyvirus__uc"
0 0 0 3 5 32 0 0 0 0 0 0 0
0 0 0 0 0 0 0 0 0 0 0 0 0
0 0 0 0 0 0 0 0 0 0 0 0 0
0 0 1 0 0 0 0 0 7 0 0 0 0
0 0 0 0 0 0 0 0 0 0 0 0 0
0 0 0 0 0 2 0
"Viruses;Uroviricota;Caudoviricetes;Caudoviricetes__na;Caudoviricetes__na;Lillamyvirus;Lillamyvirus_morran
" 0 0 0 0 0 6 0 0 0 0 0 0 0
0 0 0 0 0 0 0 0 0 0 0 0 0
0 0 0 0 0 0 0 0 0 0 0 0 0
0 0 0 0 0 0 0 0 0 0 0 0 0
0 0 0 0 0 0 0 0 0 0 0 0 0
0 0 0 0 0 0 0
"Viruses;Uroviricota;Caudoviricetes;Caudoviricetes__na;Caudoviricetes__na;Limdunavirus;Leuconostoc_phage
_Diderot" 0 0 0 0 0 0 0 0 0 0 0 0
0 0 0 0 0 0 0 0 0 0 0 0 0
0 0 0 0 0 0 0 0 0 0 0 0 0
0 0 0 0 0 0 0 0 0 0 0 0 0
0 0 0 3 3 0 0 0 0 0 0 0 0
0 0 0 0 0 0 0 0
"Viruses;Uroviricota;Caudoviricetes;Caudoviricetes__na;Caudoviricetes__na;Limdunavirus;Limdunavirus__uc"
0 0 0 0 0 0 0 0 0 0 0 0 0
0 0 0 0 0 0 0 0 0 0 0 0 0
0 0 0 0 0 0 0 0 0 0 0 0 0
0 0 0 0 0 0 0 0 0 0 0 0 0
0 0 21 24 0 0 0 0 0 0 0 0 0
0 0 0 0 0 0 0
"Viruses;Uroviricota;Caudoviricetes;Caudoviricetes__na;Caudoviricetes__na;Limdunavirus;Limdunavirus_LDG
" 0 0 0 0 0 0 0 0 0 0 0 0 0
0 0 0 0 0 0 0 0 0 0 0 0 0
0 0 0 0 0 0 0 0 0 0 0 0 0
0 0 0 0 0 0 0 0 0 0 0 0 0
0 0 0 2 0 0 0 0 0 0 0 0 0
0 0 0 0 0 0 0
"Viruses;Uroviricota;Caudoviricetes;Caudoviricetes__na;Caudoviricetes__na;Limdunavirus;Limdunavirus_Lmd
1" 0 0 0 0 0 0 0 0 0 0 0 0 0
0 0 0 0 0 0 0 0 0 0 0 0 0
0 0 0 0 0 0 0 0 0 0 0 0 0
0 0 0 0 0 0 0 0 0 0 0 0 0
0 0 5 4 0 0 0 0 0 0 0 0 0
0 0 0 0 0 0 0
"Viruses;Uroviricota;Caudoviricetes;Caudoviricetes__na;Caudoviricetes__na;Limdunavirus;Limdunavirus_LN0
3" 0 0 0 0 0 0 0 0 0 0 0 0 0
0 0 0 0 0 0 0 0 0 0 0 0 0
0 0 0 0 0 0 0 0 0 0 0 0 0
0 0 0 0 0 0 0 0 0 0 0 0 0

0 0 2 0 0 0 0 0 0 0 0 0 0
0 0 0 0 0 0 0
"Viruses;Uroviricota;Caudoviricetes;Caudoviricetes__na;Caudoviricetes__na;Limdunavirus;Limdunavirus_LN0
4" 0 0 0 0 0 0 0 0 0 0 0 0 0
0 0 0 0 0 0 0 0 0 0 0 0 0
0 0 0 0 0 0 0 0 0 0 0 0 0
0 0 0 0 0 0 0 0 0 0 0 0 0
0 0 2 1 0 0 0 0 0 0 0 0 0
0 0 0 0 0 0 0
"Viruses;Uroviricota;Caudoviricetes;Caudoviricetes__na;Caudoviricetes__na;Limdunavirus;Limdunavirus_P965
" 0 0 0 0 0 0 0 0 0 0 0 0 0
0 0 0 0 0 0 0 0 0 0 0 0 0
0 0 0 0 0 0 0 0 0 0 0 0 0
0 0 0 0 0 0 0 0 0 0 0 0 0
0 0 1 0 0 0 0 0 0 0 0 0 0
0 0 0 0 0 0 0
"Viruses;Uroviricota;Caudoviricetes;Caudoviricetes__na;Caudoviricetes__na;Luckybarnesvirus;Luckybarnesvir
us_luckybarnes" 0 0 0 0 0 0 0 0 0 2 0
0 0 0 0 0 0 0 0 0 0 0 0 0
0 0 0 0 0 0 0 0 0 0 0 0 0
0 0 0 0 0 0 0 0 0 0 0 0 0
0 0 0 0 0 0 0 0 0 0 0 0 0
0 0 0 0 0 0 0 0 0
"Viruses;Uroviricota;Caudoviricetes;Caudoviricetes__na;Caudoviricetes__na;Mapvirus;Mapvirus__uc" 0
1 0 0 0 0 0 0 0 0 0 0 0 0
0 0 0 0 0 0 0 0 0 0 0 0 0
0 0 0 0 0 0 0 0 0 0 1 0 0
0 0 0 0 0 0 0 0 0 0 0 0 0
0 0 0 0 0 0 0 0 0 0 0 0 0
0 0 0 0 0 0
"Viruses;Uroviricota;Caudoviricetes;Caudoviricetes__na;Caudoviricetes__na;Menderavirus;Menderavirus__uc"
0 0 0 2 0 0 0 0 0 0 0 0 0
0 0 0 0 0 0 0 0 0 0 0 0 0
0 0 0 0 0 0 0 0 0 0 0 0 0
0 0 0 0 0 0 0 4 0 0 0 0 0
0 0 0 0 0 0 0 0 0 0 0 0 0
0 0 0 0 0 0 0
"Viruses;Uroviricota;Caudoviricetes;Caudoviricetes__na;Caudoviricetes__na;Metrivirus;Metrivirus_ME3"0
0 0 0 0 0 0 0 0 1 0 0 0 0
0 0 0 0 0 0 0 0 0 0 0 0 0
0 0 0 0 0 0 0 0 0 0 0 0 0
0 0 0 0 0 0 0 0 0 0 0 0 0
0 0 0 0 0 0 0 0 0 0 0 0 0
0 0 0 0 0 0
"Viruses;Uroviricota;Caudoviricetes;Caudoviricetes__na;Caudoviricetes__na;Micavirus;Micavirus_Mica" 0
0 0 1 1 0 0 0 0 0 0 0 0 0
0 0 0 0 0 0 0 0 0 0 0 0 0
0 0 0 0 0 0 0 0 0 0 0 0 0
0 0 0 0 0 0 0 0 0 0 0 0 0

0 0 0 0 0 0 0 0 0 0 0 0 0
0 0 0 0 0 0
"Viruses;Uroviricota;Caudoviricetes;Caudoviricetes__na;Caudoviricetes__na;Mieseafarmvirus;Mieseafarmvirus_RSL1" 0 0 0 1 0 0 0 0 0 0 0 0 0
0 0 0 0 0 0 0 0 0 0 0 0 0
0 0 0 0 0 0 0 0 0 0 0 0 0
0 0 0 0 0 0 0 0 0 0 0 0 0
0 0 0 0 0 0 0 0 0 0 0 0 0
0 0 0 0 0 0 0
"Viruses;Uroviricota;Caudoviricetes;Caudoviricetes__na;Caudoviricetes__na;Mimasvirus;Xanthomonas_phage_XbC2" 0 0 0 0 0 0 0 0 0 0 0 0 0
0 0 0 0 0 0 0 0 0 0 0 0 0
0 0 0 0 0 0 0 0 0 0 0 0 0
0 0 0 0 0 0 0 0 0 0 0 0 0
0 0 0 0 0 0 0 8 0 0 0 0 0
0 0 0 0 0 0 0
"Viruses;Uroviricota;Caudoviricetes;Caudoviricetes__na;Caudoviricetes__na;Moabitevirus;Serratia_phage_vB_SmaS-Totoro" 0 0 0 0 3 0 0 0 0 0 0 0
0 0 0 0 0 0 0 0 0 0 0 0 0
0 0 0 0 0 0 0 0 0 0 0 0 0
0 0 0 0 0 0 0 0 0 0 0 0 0
0 0 0 0 0 0 0 0 0 0 0 0 0
0 0 0 0 0 0 0 0
"Viruses;Uroviricota;Caudoviricetes;Caudoviricetes__na;Caudoviricetes__na;Moineauvirus;Moineauvirus__uc"
0 0 0 0 0 0 0 0 0 0 0 0 0
0 0 0 0 0 0 0 0 0 0 0 0 0
0 0 0 0 0 0 0 0 0 0 0 0 0
0 0 0 0 0 0 0 0 0 0 0 0 0
0 0 2 1 0 0 0 0 0 0 0 0 0
0 1 0 0 0 0 0
"Viruses;Uroviricota;Caudoviricetes;Caudoviricetes__na;Caudoviricetes__na;Moineauvirus;Streptococcus_phage_CHPC1034" 0 0 0 0 0 0 0 0 0 0 0 0
0 0 0 0 0 0 0 0 0 0 0 0 0
0 0 0 0 0 0 0 0 0 0 0 0 0
0 0 0 0 0 0 0 0 0 0 0 0 0
0 0 0 0 0 0 0 0 0 0 1 0 0
0 0 0 0 0 0 0 0
"Viruses;Uroviricota;Caudoviricetes;Caudoviricetes__na;Caudoviricetes__na;Moineauvirus;Streptococcus_phage_CHPC1041" 0 0 0 0 0 0 0 0 0 0 0 0
0 0 0 0 0 0 0 0 0 0 0 0 0
0 0 0 0 0 0 0 0 0 0 0 0 0
0 0 0 0 0 0 0 0 0 0 0 0 1
0 0 0 0 0 0 0 0 0 0 0 0 0
0 0 0 0 0 0 0 0
"Viruses;Uroviricota;Caudoviricetes;Caudoviricetes__na;Caudoviricetes__na;Moineauvirus;Streptococcus_phage_SFi18" 0 0 0 0 0 0 0 0 0 0 0 0
0 0 0 0 0 1 0 0 0 0 0 0 0
0 0 0 0 0 0 0 0 0 0 0 0 0
0 0 0 0 0 0 0 0 0 0 0 0 0

0 0 0 0 0 0 0 0 0 0 0 0 0
0 0 0 0 0 0 0 0
"Viruses;Uroviricota;Caudoviricetes;Caudoviricetes__na;Caudoviricetes__na;Mooglevirus;Mooglevirus__uc"
0 0 0 2 0 0 0 0 0 0 0 0 0
0 0 0 0 0 0 0 0 0 0 0 0 0
0 0 0 0 0 0 0 0 0 0 0 0 0
0 0 0 0 0 0 0 0 0 0 0 0 0
0 0 0 0 0 0 0 0 0 0 0 0 0
0 0 0 0 0 0 0
"Viruses;Uroviricota;Caudoviricetes;Caudoviricetes__na;Caudoviricetes__na;Mudcatvirus;Arthrobacter_phage_
Correa" 0 0 0 0 0 1 0 0 0 0 0 0 0
0 0 0 0 0 0 0 0 0 0 0 0 0
0 0 0 0 0 0 0 0 0 0 0 0 0
0 0 0 0 0 0 0 0 0 0 0 0 0
0 0 0 0 0 0 0 0 0 0 0 0 0
0 0 0 0 0 0 0
"Viruses;Uroviricota;Caudoviricetes;Caudoviricetes__na;Caudoviricetes__na;Muminvirus;Muminvirus__uc"
0 0 1 1 14 0 0 0 0 0 0 0 0
0 0 0 1 1 0 0 0 0 0 1 0 0
0 0 0 0 0 0 0 0 0 0 0 0 0
0 0 0 0 0 0 0 0 0 0 0 0 0
0 0 0 0 0 0 0 0 0 0 0 0 0
0 0 0 0 0 0 0
"Viruses;Uroviricota;Caudoviricetes;Caudoviricetes__na;Caudoviricetes__na;Muminvirus;Muminvirus_filifjonk
" 0 0 0 1 38 16 0 0 0 0 0 0 0
0 0 0 0 0 0 0 0 0 0 0 0 0
0 0 0 0 0 0 0 0 0 0 0 0 0
0 0 0 0 0 0 0 0 0 0 0 0 0
0 0 0 0 0 0 0 0 0 0 0 0 0
0 0 0 0 0 0 0
"Viruses;Uroviricota;Caudoviricetes;Caudoviricetes__na;Caudoviricetes__na;Muminvirus;Muminvirus_mymlan
" 0 0 0 2 18 0 0 0 0 0 0 0 0
0 0 0 0 0 0 0 0 0 0 0 0 0
0 0 0 0 0 0 0 0 0 0 0 0 0
0 0 0 0 0 0 0 0 0 0 0 0 0
0 0 0 0 0 0 0 0 0 0 0 0 0
0 0 0 0 0 0 0
"Viruses;Uroviricota;Caudoviricetes;Caudoviricetes__na;Caudoviricetes__na;Muminvirus;Muminvirus_snusmu
m" 0 0 0 1 0 0 0 0 0 0 0 0 0
0 0 0 0 0 0 0 0 0 0 0 0 0
0 0 0 0 0 0 0 0 0 0 0 0 0
0 0 0 0 0 0 0 0 0 0 0 0 0
0 0 0 0 0 0 0 0 0 0 0 0 0
0 0 0 0 0 0 0
"Viruses;Uroviricota;Caudoviricetes;Caudoviricetes__na;Caudoviricetes__na;Muminvirus;Muminvirus_tooticki"
0 0 2 0 1 1 0 0 0 0 0 0 0
0 0 0 2 0 0 0 0 0 0 0 0 0
0 0 0 0 0 0 0 0 0 0 0 0 0
0 0 0 0 0 0 0 0 2 0 0 0 0

0 0 0 0 0 0 0 0 0 0 0 0 0
0 0 0 0 0 0 0
"Viruses;Uroviricota;Caudoviricetes;Caudoviricetes__na;Caudoviricetes__na;Mushuvirus;Mushuvirus_mushu"
0 0 0 0 0 0 0 0 0 0 0 0 0
0 0 0 0 0 5 1 0 0 0 0 0 0
0 0 0 0 0 1 0 0 0 0 1 0 0
0 0 0 0 1 0 0 0 0 0 0 0 0
0 0 0 0 0 0 0 0 1 0 0 0 0
0 2 2 0 0 0 0
"Viruses;Uroviricota;Caudoviricetes;Caudoviricetes__na;Caudoviricetes__na;Mydovirus;Mydovirus_KpS8"
0 0 0 0 0 0 0 0 0 0 0 0 0
0 0 0 0 0 0 0 0 0 0 0 0 0
0 0 0 0 0 0 0 0 0 0 0 0 0
0 0 0 0 0 0 0 0 5 0 0 0 0
0 0 0 0 0 0 0 0 0 0 0 0 0
0 0 0 0 0 0 0
"Viruses;Uroviricota;Caudoviricetes;Caudoviricetes__na;Caudoviricetes__na;Myradeevirus;Myradeevirus_Myra
Dee" 0 0 0 0 1 0 0 0 0 0 0 0 0
0 0 0 0 0 0 0 0 0 0 0 0 0
0 0 0 0 0 0 0 0 0 0 0 0 0
0 0 0 0 0 0 0 0 0 0 0 0 0
0 0 0 0 0 0 0 0 0 0 0 0 0
0 0 0 0 0 0 0
"Viruses;Uroviricota;Caudoviricetes;Caudoviricetes__na;Caudoviricetes__na;Myrnavirus;Myranavirus_phabba"
0 0 1 0 15 0 0 0 0 0 0 0 0
0 0 0 0 0 0 0 0 0 0 0 0 0
0 0 0 0 0 0 0 0 0 0 0 0 0
0 0 0 0 0 0 0 0 0 0 0 0 0
0 0 0 0 0 0 0 0 0 0 0 0 0
0 0 0 0 0 0 0
"Viruses;Uroviricota;Caudoviricetes;Caudoviricetes__na;Caudoviricetes__na;Nanhaivirus;Nanhaivirus_D5C"
0 0 0 1 0 0 0 0 0 0 0 0 0
0 0 0 0 0 0 0 0 0 0 0 0 0
0 0 0 0 0 0 0 0 0 0 0 0 0
0 0 0 0 0 0 0 0 1 0 0 0 0
0 0 0 0 0 0 0 0 0 0 0 0 0
0 0 0 0 0 0 0
"Viruses;Uroviricota;Caudoviricetes;Caudoviricetes__na;Caudoviricetes__na;Nickievirus;Nickievirus__uc"
0 0 0 0 0 0 0 0 0 0 0 0 0
0 0 0 0 0 0 0 0 0 0 0 0 0
0 0 0 0 0 0 0 0 0 0 0 0 0
0 0 0 0 0 0 0 0 0 0 0 0 0
0 0 0 0 0 0 0 0 0 0 0 0 0
0 0 0 0 0 1 0
"Viruses;Uroviricota;Caudoviricetes;Caudoviricetes__na;Caudoviricetes__na;Nipunavirus;Nipunavirus__uc"
1 1 3 0 0 0 0 0 0 0 0 0 0
0 0 0 0 0 0 0 0 0 0 0 0 0
0 0 0 0 0 0 0 0 0 0 0 0 0
0 0 0 0 0 0 0 0 0 0 0 0 0

0 0 0 0 0 0 0 0 0 0 0 0 0
0 0 0 0 0 0 0
"Viruses;Uroviricota;Caudoviricetes;Caudoviricetes__na;Caudoviricetes__na;Nipunavirus;Nipunavirus_PaMx2
5" 0 0 0 0 0 2 0 0 0 0 0 0 0
0 0 0 0 0 0 0 0 0 0 0 0 0
0 0 0 0 0 0 0 0 0 0 0 0 0
0 0 0 0 0 0 0 0 0 0 0 0 0
0 0 0 0 0 0 0 0 0 0 0 0 0
0 0 0 0 0 0 0
"Viruses;Uroviricota;Caudoviricetes;Caudoviricetes__na;Caudoviricetes__na;Nipunavirus;Pseudomonas_phage
_JG054" 0 0 0 0 0 0 0 0 0 0 0 0 0
0 0 0 0 0 0 0 0 0 0 0 0 0
0 0 0 0 0 0 0 0 0 0 0 0 0
0 0 0 0 0 1 0 0 0 0 0 0 0
0 0 0 0 0 0 0 0 0 0 0 0 0
0 0 0 0 0 0 0
"Viruses;Uroviricota;Caudoviricetes;Caudoviricetes__na;Caudoviricetes__na;Nipunavirus;Pseudomonas_phage
_pPA-3099-2aT.3" 0 0 0 0 0 1 0 0 0 0 0
0 0 0 0 0 0 0 0 0 0 0 0 0
0 0 0 0 0 0 0 0 0 0 0 0 0
0 0 0 0 0 0 0 0 0 0 0 0 0
0 0 0 0 0 0 0 0 0 0 0 0 0
0 0 0 0 0 0 0 0 0
"Viruses;Uroviricota;Caudoviricetes;Caudoviricetes__na;Caudoviricetes__na;Noxifervirus;Noxifervirus_noxifer"
0 0 0 1 0 0 0 0 0 0 0 0 0
0 0 0 0 0 0 0 0 0 0 0 0 0
0 0 0 0 0 0 0 0 0 0 0 0 0
0 0 0 0 0 0 0 0 0 0 0 0 0
0 0 0 0 0 0 0 0 0 0 0 0 0
0 0 0 0 0 0 0
"Viruses;Uroviricota;Caudoviricetes;Caudoviricetes__na;Caudoviricetes__na;Obolenskvirus;Acinetobacter_phag
e_Bphi-R1888" 0 0 0 1 0 0 0 0 0 0 0 0
0 0 0 0 0 0 0 0 0 0 0 0 0
0 0 0 0 0 0 0 0 0 0 0 0 0
0 0 0 0 0 0 0 0 0 2 0 0 0
0 0 0 0 0 0 0 0 0 0 0 0 0
0 0 0 0 0 0 0 0
"Viruses;Uroviricota;Caudoviricetes;Caudoviricetes__na;Caudoviricetes__na;Obolenskvirus;Acinetobacter_phag
e_Cato" 0 0 0 0 0 0 0 0 0 0 0 0 0
0 0 0 0 0 0 0 0 0 0 0 0 0
0 0 0 0 0 0 0 0 0 0 0 0 0
0 0 0 0 0 0 0 0 0 0 0 0 0
0 0 0 0 0 0 0 0 0 0 0 0 0
0 0 0 0 0 2 0
"Viruses;Uroviricota;Caudoviricetes;Caudoviricetes__na;Caudoviricetes__na;Obolenskvirus;Staphylococcus_ph
age_XC1" 0 0 0 0 0 0 0 0 0 1 0 0
0 0 0 0 0 0 0 0 0 0 0 0 0
0 0 0 0 0 0 0 0 0 0 0 0 0
0 0 0 0 0 0 0 0 0 0 0 0 0

0 0 0 0 0 0 0 0 0 0 0 0 0
0 0 0 0 0 0 0 0
"Viruses;Uroviricota;Caudoviricetes;Caudoviricetes__na;Caudoviricetes__na;Oengusvirus;Oengusvirus_oengus
" 0 0 0 0 0 0 0 0 0 0 0 0 0
0 0 0 0 0 0 0 0 0 0 0 5 9
0 0 0 0 0 0 0 0 0 0 0 0 0
0 0 0 0 0 0 0 0 0 0 0 0 0
0 0 0 1 0 0 0 0 0 0 0 0 0
0 0 0 0 0 0 0
"Viruses;Uroviricota;Caudoviricetes;Caudoviricetes__na;Caudoviricetes__na;Omegavirus;Omegavirus__uc"
0 0 0 0 1 0 0 0 0 0 0 0 0
0 0 0 0 0 0 0 0 0 0 0 0 0
0 0 0 0 0 0 0 0 0 0 0 0 0
0 0 0 0 0 0 0 0 0 0 0 0 0
0 0 0 0 0 0 0 0 0 0 0 0 0
0 0 0 0 0 0 0
"Viruses;Uroviricota;Caudoviricetes;Caudoviricetes__na;Caudoviricetes__na;Oneupvirus;Oneupvirus_oneup"
0 0 0 0 0 1 0 0 0 0 0 0 0
0 0 0 0 0 0 0 0 0 0 0 0 0
0 0 0 0 0 0 0 0 0 0 0 0 0
0 0 0 0 0 0 0 0 0 0 0 0 0
0 0 0 0 0 0 0 0 0 0 0 0 0
0 0 0 0 0 0 0
"Viruses;Uroviricota;Caudoviricetes;Caudoviricetes__na;Caudoviricetes__na;Orchidvirus;Orchidvirus__uc"
0 0 0 0 0 32 0 0 0 0 0 0 0
0 0 0 0 0 0 0 0 0 0 0 0 0
0 0 0 0 0 0 0 0 0 0 0 0 0
0 0 0 0 0 0 0 0 0 0 0 0 0
0 0 0 0 0 0 0 0 0 0 0 0 0
0 0 0 0 0 0 0
"Viruses;Uroviricota;Caudoviricetes;Caudoviricetes__na;Caudoviricetes__na;Otagovirus;Otagovirus__uc"
0 0 0 0 0 1 0 0 0 0 0 0 0
0 0 0 0 0 0 0 0 0 0 0 0 0
0 0 0 0 0 0 0 0 0 0 0 0 0
0 0 0 0 0 0 0 0 0 0 0 0 0
0 0 0 0 0 0 0 0 0 0 0 0 0
0 0 0 0 0 0 0
"Viruses;Uroviricota;Caudoviricetes;Caudoviricetes__na;Caudoviricetes__na;Otagovirus;Pseudomonas_phage_
Astolliot" 0 0 0 0 0 0 0 0 0 1 0 0
0 0 0 0 0 0 0 0 0 0 0 0 0
0 0 0 0 0 0 0 0 0 0 0 0 0
0 0 0 0 0 0 0 0 0 0 0 0 0
0 0 0 0 0 0 0 0 0 0 0 0 0
0 0 0 0 0 0 0 0
"Viruses;Uroviricota;Caudoviricetes;Caudoviricetes__na;Caudoviricetes__na;Otagovirus;Pseudomonas_phage_
PN09" 0 0 0 0 0 0 0 0 0 0 0 0 0
0 0 0 0 0 0 0 0 0 0 0 0 0
0 0 0 0 0 0 0 0 0 0 0 0 0
0 0 0 0 0 0 1 0 0 0 0 0 0

0 0 0 0 0 0 0 0 0 0 0 0 0
0 0 0 0 0 0 0
"Viruses;Uroviricota;Caudoviricetes;Caudoviricetes__na;Caudoviricetes__na;Pahexavirus;Cutibacterium_phage
_FD1" 0 0 0 0 0 0 0 0 0 0 1 0 0
0 0 0 0 0 0 0 0 0 0 0 0 0
0 0 0 0 0 0 0 0 0 0 0 0 0
0 0 0 0 0 0 0 0 0 0 0 0 0
0 0 0 0 0 0 0 0 0 0 0 0 0
0 0 0 0 0 0 0
"Viruses;Uroviricota;Caudoviricetes;Caudoviricetes__na;Caudoviricetes__na;Pahexavirus;Cutibacterium_phage
_FD3" 0 0 0 0 0 1 0 0 0 0 139 0 0
0 0 0 0 0 0 0 0 0 0 0 0 0
0 0 0 0 0 0 0 0 0 4 0 0 0
0 0 1 0 0 0 0 0 0 0 0 0 0
0 0 0 0 0 0 0 2 0 0 0 0 0
0 0 0 0 0 0 0
"Viruses;Uroviricota;Caudoviricetes;Caudoviricetes__na;Caudoviricetes__na;Pahexavirus;Cutibacterium_phage
_P104B"0 0 0 0 0 0 0 0 0 0 14 0 0
0 0 0 0 0 0 0 0 0 0 0 0 0
0 0 0 0 0 0 0 0 0 0 0 0 0
0 0 0 0 0 0 0 0 0 0 0 0 0
0 0 0 0 0 0 0 0 0 0 0 0 0
0 0 0 0 0 0 0
"Viruses;Uroviricota;Caudoviricetes;Caudoviricetes__na;Caudoviricetes__na;Pahexavirus;Cutibacterium_phage
_P107A" 0 0 0 0 2 0 0 0 0 0 11 0
0 0 0 0 0 0 0 0 0 0 0 0 0
0 0 0 0 0 0 0 0 0 0 0 0 0
0 0 0 0 0 0 0 0 0 1 0 0 0
0 0 0 0 0 0 0 0 0 0 0 0 0
0 0 0 0 0 0 0 0
"Viruses;Uroviricota;Caudoviricetes;Caudoviricetes__na;Caudoviricetes__na;Pahexavirus;Cutibacterium_phage
_P108C" 0 0 0 0 0 0 0 0 0 1 0 0
0 0 0 0 0 0 0 0 0 0 0 0 0
0 0 0 0 0 0 0 0 0 0 0 0 0
0 0 0 0 0 0 0 0 0 0 0 0 0
0 0 0 0 0 0 0 0 0 0 0 0 0
0 0 0 0 0 0 0 0
"Viruses;Uroviricota;Caudoviricetes;Caudoviricetes__na;Caudoviricetes__na;Pahexavirus;Cutibacterium_phage
_PAP1-1" 0 0 0 0 0 0 0 0 0 0 2 0
0 0 0 0 0 0 0 0 0 0 0 0 0
0 0 0 0 0 0 0 0 0 0 0 0 0
0 0 0 0 0 0 0 0 0 0 0 0 0
0 0 0 0 0 0 0 0 0 0 0 0 0
0 0 0 0 0 0 0 0
"Viruses;Uroviricota;Caudoviricetes;Caudoviricetes__na;Caudoviricetes__na;Pahexavirus;Cutibacterium_phage
_PAVL21" 0 0 0 0 0 0 0 0 0 0 38 0
0 0 0 0 0 0 0 0 0 0 0 0 0
0 0 0 0 0 0 0 0 0 0 2 0 0
0 0 0 6 0 0 0 0 4 0 0 0 0

0 0 0 0 0 0 0 0 0 0 0 0 0
0 0 0 0 0 0 0 0
"Viruses;Uroviricota;Caudoviricetes;Caudoviricetes__na;Caudoviricetes__na;Pahexavirus;Cutibacterium_phage_PAVL33" 0 0 0 0 0 0 0 0 0 0 1 0
0 0 0 0 0 0 0 0 0 0 0 0 0
0 0 0 0 0 0 0 0 0 0 0 0 0
0 0 0 0 0 0 0 0 0 0 0 0 0
0 0 0 0 0 0 0 0 0 0 0 0 0
0 0 0 0 0 0 0 0
"Viruses;Uroviricota;Caudoviricetes;Caudoviricetes__na;Caudoviricetes__na;Pahexavirus;Cutibacterium_phage_PAVL34" 0 0 0 0 0 0 0 0 0 0 7 0
0 0 0 0 0 0 0 0 0 0 0 0 0
0 0 0 0 0 0 0 0 0 0 0 0 0
0 0 0 0 0 0 0 0 0 0 0 0 0
0 0 0 0 0 0 0 0 0 0 0 0 0
0 0 0 0 0 0 0 0
"Viruses;Uroviricota;Caudoviricetes;Caudoviricetes__na;Caudoviricetes__na;Pahexavirus;Cutibacterium_phage_Y3Z" 0 0 0 0 0 0 0 0 0 0 4 0 0
0 0 0 0 0 0 0 0 0 0 0 0 0
0 0 0 0 0 0 0 0 0 0 0 0 0
0 0 1 0 0 0 0 0 0 0 0 0 0
0 0 0 0 0 0 0 0 0 0 0 0 0
0 0 0 0 0 0 0
"Viruses;Uroviricota;Caudoviricetes;Caudoviricetes__na;Caudoviricetes__na;Pahexavirus;Pahexavirus__uc"
0 0 0 2 50 0 2 0 2 5 505 26 1
0 0 0 0 1 0 0 0 0 0 0 0 0
0 0 0 0 0 0 0 0 5 70 0 0 0
0 0 16 0 0 1 0 48 8 0 0 0 0
0 0 0 0 0 0 0 0 0 0 0 0 0
0 0 0 0 0 0 0
"Viruses;Uroviricota;Caudoviricetes;Caudoviricetes__na;Caudoviricetes__na;Pahexavirus;Pahexavirus_attacne"
0 0 2 0 0 0 0 0 0 0 0 0 0
0 0 0 0 0 0 0 0 0 0 0 0 0
0 0 0 0 0 0 0 0 0 2 0 0 0
0 0 0 0 0 0 0 0 0 0 0 0 0
0 0 0 0 0 0 0 0 0 0 0 0 0
0 0 0 0 0 0 0
"Viruses;Uroviricota;Caudoviricetes;Caudoviricetes__na;Caudoviricetes__na;Pahexavirus;Pahexavirus_lauchelly" 0 0 0 0 0 0 0 0 0 0 30 0 0
0 0 0 0 0 0 0 0 0 0 0 0 0
0 0 0 0 0 0 0 0 0 5 0 0 0
0 0 0 0 0 0 0 0 0 0 0 0 0
0 0 0 0 0 0 0 0 0 0 0 0 0
0 0 0 0 0 0 0
"Viruses;Uroviricota;Caudoviricetes;Caudoviricetes__na;Caudoviricetes__na;Pahexavirus;Pahexavirus_mrAK"
0 0 0 0 0 0 0 0 0 0 1 0 0
0 0 0 0 0 0 0 0 0 0 0 0 0
0 0 0 0 0 0 0 0 0 0 0 0 0
0 0 0 0 0 0 0 0 0 0 0 0 0

0 0 0 0 0 0 0 0 0 0 0 0 0
0 0 0 0 0 0 0
"Viruses;Uroviricota;Caudoviricetes;Caudoviricetes__na;Caudoviricetes__na;Pahexavirus;Pahexavirus_ourobor
os" 0 0 0 0 0 0 0 0 0 0 6 0 0
0 0 0 0 0 0 0 0 0 0 0 0 0
0 0 0 0 0 0 0 0 0 3 0 0 0
0 0 0 0 0 0 0 0 0 0 0 0 0
0 0 0 0 0 0 0 0 0 0 0 0 0
0 0 0 0 0 0 0
"Viruses;Uroviricota;Caudoviricetes;Caudoviricetes__na;Caudoviricetes__na;Pahexavirus;Pahexavirus_P1001"
0 0 0 0 0 0 0 0 0 0 0 0 0
0 0 0 0 0 0 0 0 0 0 0 0 0
0 0 0 0 0 0 0 0 0 3 0 0 0
0 0 0 0 0 0 0 0 0 0 0 0 0
0 0 0 0 0 0 0 0 0 0 0 0 0
0 0 0 0 0 0 0
"Viruses;Uroviricota;Caudoviricetes;Caudoviricetes__na;Caudoviricetes__na;Pahexavirus;Pahexavirus_P100A"
0 0 0 0 0 0 0 0 0 0 1 0 0
0 0 0 0 0 0 0 0 0 0 0 0 0
0 0 0 0 0 0 0 0 0 3 0 0 0
0 0 0 0 0 0 0 0 0 0 0 0 0
0 0 0 0 0 0 0 0 0 0 0 0 0
0 0 0 0 0 0 0
"Viruses;Uroviricota;Caudoviricetes;Caudoviricetes__na;Caudoviricetes__na;Pahexavirus;Pahexavirus_P100D"
0 0 0 0 0 0 0 0 0 0 4 0 0
0 0 0 0 0 0 0 0 0 0 0 0 0
0 0 0 0 0 0 0 0 0 2 0 0 0
0 0 0 0 0 0 0 0 0 0 0 0 0
0 0 0 0 0 0 0 0 0 0 0 0 0
0 0 0 0 0 0 0
"Viruses;Uroviricota;Caudoviricetes;Caudoviricetes__na;Caudoviricetes__na;Pahexavirus;Pahexavirus_P101A"
0 0 0 0 0 0 0 0 0 0 1 0 0
0 0 0 0 0 0 0 0 0 0 0 0 0
0 0 0 0 0 0 0 0 0 0 0 0 0
0 0 4 0 0 0 0 0 0 0 0 0 0
0 0 0 0 0 0 0 0 0 0 0 0 0
0 0 0 0 0 0 0
"Viruses;Uroviricota;Caudoviricetes;Caudoviricetes__na;Caudoviricetes__na;Pahexavirus;Pahexavirus_P104A"
0 0 0 0 0 0 0 0 0 0 27 0 0
0 0 0 0 0 0 0 0 0 0 0 0 0
0 0 0 0 0 0 0 0 0 0 0 0 0
0 0 0 0 0 0 0 6 0 0 0 0 0
0 0 0 0 0 0 0 0 0 0 0 0 0
0 0 0 0 0 0 0
"Viruses;Uroviricota;Caudoviricetes;Caudoviricetes__na;Caudoviricetes__na;Pahexavirus;Pahexavirus_P105"
0 0 0 0 1 0 0 0 0 0 13 0 0
0 0 0 0 0 0 0 0 0 1 0 0 0
0 0 0 0 0 0 0 0 0 3 0 0 0
0 0 0 0 0 0 0 0 0 0 0 0 0

0	0	0	0	0	0	0	0	0	0	0	0	0
0	0	0	0	0	0	0
"Viruses;Uroviricota;Caudoviricetes;Caudoviricetes__na;Caudoviricetes__na;Pahexavirus;Pahexavirus_P11"
0	0	0	0	0	0	0	0	0	0	2	0	0
0	0	0	0	0	0	0	0	0	0	0	0	0
0	0	0	0	0	0	0	0	0	0	0	0	0
0	0	0	0	0	0	0	0	0	0	0	0	0
0	0	0	0	0	0	0	0	0	0	0	0	0
0	0	0	0	0	0	0
"Viruses;Uroviricota;Caudoviricetes;Caudoviricetes__na;Caudoviricetes__na;Pahexavirus;Pahexavirus_PA6"
0	0	0	0	0	0	0	0	0	0	25	0	0
0	0	0	0	0	0	0	0	0	0	0	0	0
0	0	0	0	0	0	0	0	0	0	0	0	0
0	0	2	0	0	0	0	0	0	0	0	0	0
0	0	0	0	0	0	0	0	0	0	0	0	0
0	0	0	0	0	0	0
"Viruses;Uroviricota;Caudoviricetes;Caudoviricetes__na;Caudoviricetes__na;Pahexavirus;Pahexavirus_pacnes2
01215"	0	0	0	0	1	0	0	0	0	1	2	0	0
0	0	0	0	0	0	0	0	0	0	0	0	0
0	0	0	0	0	0	0	0	0	1	0	0	0
0	0	3	0	0	0	0	0	0	0	0	0	0
0	0	0	0	0	0	0	0	0	0	0	0	0
0	0	0	0	0	0	0
"Viruses;Uroviricota;Caudoviricetes;Caudoviricetes__na;Caudoviricetes__na;Pahexavirus;Pahexavirus_PAD20"
0	0	0	0	0	0	0	0	0	0	0	0	0
0	0	0	0	0	0	0	0	0	0	0	0	0
0	0	0	0	0	0	0	0	0	4	0	0	0
0	0	12	0	0	0	0	0	0	0	0	0	0
0	0	0	0	0	0	0	0	0	0	0	0	0
0	0	0	0	0	0	0
"Viruses;Uroviricota;Caudoviricetes;Caudoviricetes__na;Caudoviricetes__na;Pahexavirus;Pahexavirus_PAS50"
0	0	0	0	0	0	0	0	0	0	3	0	0
0	0	0	0	0	0	0	0	0	0	0	0	0
0	0	0	0	0	0	0	0	0	0	0	0	0
0	0	0	0	0	0	0	0	2	0	0	0	0
0	0	0	0	0	0	0	1	0	0	0	0	0
0	0	0	0	0	0	0
"Viruses;Uroviricota;Caudoviricetes;Caudoviricetes__na;Caudoviricetes__na;Pahexavirus;Pahexavirus_PHL009
M11"	0	0	0	0	1	0	0	0	0	0	0	0	0
0	0	0	0	0	0	0	0	0	0	0	0	0
0	0	0	0	0	0	0	0	0	0	0	0	0
0	0	0	0	0	0	0	0	0	0	0	0	0
0	0	0	0	0	0	0	0	0	0	0	0	0
0	0	0	0	0	0	0
"Viruses;Uroviricota;Caudoviricetes;Caudoviricetes__na;Caudoviricetes__na;Pahexavirus;Pahexavirus_PHL025
M00"	0	0	0	0	0	0	0	0	0	0	0	0	0
0	0	0	0	0	0	0	0	0	0	0	0	0
0	0	0	0	0	0	0	0	0	1	0	0	0
0	0	0	0	0	0	0	0	0	0	0	0	0

0 0 0 0 0 0 0 0 0 0 0 0 0
0 0 0 0 0 0 0
"Viruses;Uroviricota;Caudoviricetes;Caudoviricetes__na;Caudoviricetes__na;Pahexavirus;Pahexavirus_PHL041
M10" 0 0 1 0 0 0 0 0 0 0 6 0 0
0 0 0 0 0 0 0 0 0 0 0 0 0
0 0 0 0 0 0 0 0 0 5 0 0 0
0 0 0 0 0 0 0 0 0 0 0 0 0
0 0 0 0 0 0 0 0 0 0 0 0 0
0 0 0 0 0 0 0
"Viruses;Uroviricota;Caudoviricetes;Caudoviricetes__na;Caudoviricetes__na;Pahexavirus;Pahexavirus_PHL060
L00" 0 0 0 0 0 0 0 0 0 0 1 0 0
0 0 0 0 0 0 0 0 0 0 0 0 0
0 0 0 0 0 0 0 0 0 0 0 0 0
0 0 0 0 0 0 0 0 0 0 0 0 0
0 0 0 0 0 0 0 0 0 0 0 0 0
0 0 0 0 0 0 0
"Viruses;Uroviricota;Caudoviricetes;Caudoviricetes__na;Caudoviricetes__na;Pahexavirus;Pahexavirus_PHL067
M01" 0 0 0 0 0 0 0 0 0 0 3 0 0
0 0 0 0 0 0 0 0 0 0 0 0 0
0 0 0 0 0 0 0 0 0 0 0 0 0
0 0 0 0 0 0 0 0 0 0 0 0 0
0 0 0 0 0 0 0 0 0 0 0 0 0
0 0 0 0 0 0 0
"Viruses;Uroviricota;Caudoviricetes;Caudoviricetes__na;Caudoviricetes__na;Pahexavirus;Pahexavirus_PHL070
N00" 0 0 0 0 0 0 0 0 0 0 5 0 0
0 0 0 0 0 0 0 0 0 0 0 0 0
0 0 0 0 0 0 0 0 0 4 0 0 0
0 0 0 0 0 0 0 0 0 0 0 0 0
0 0 0 0 0 0 0 0 0 0 0 0 0
0 0 0 0 0 0 0
"Viruses;Uroviricota;Caudoviricetes;Caudoviricetes__na;Caudoviricetes__na;Pahexavirus;Pahexavirus_PHL071
N05" 0 0 0 0 0 0 0 0 0 0 0 0 0
0 0 0 0 0 0 0 0 0 0 0 0 0
0 0 0 0 0 0 0 0 0 1 0 0 0
0 0 0 0 0 0 0 0 0 0 0 0 0
0 0 0 0 0 0 0 0 0 0 0 0 0
0 0 0 0 0 0 0
"Viruses;Uroviricota;Caudoviricetes;Caudoviricetes__na;Caudoviricetes__na;Pahexavirus;Pahexavirus_PHL092
M00" 0 0 0 0 0 0 0 0 0 0 1 0 0
0 0 0 0 0 0 0 0 0 0 0 0 0
0 0 0 0 0 0 0 0 0 0 0 0 0
0 0 0 0 0 0 0 0 0 0 0 0 0
0 0 0 0 0 0 0 0 0 0 0 0 0
0 0 0 0 0 0 0
"Viruses;Uroviricota;Caudoviricetes;Caudoviricetes__na;Caudoviricetes__na;Pahexavirus;Pahexavirus_PHL095
N00" 0 0 0 0 0 0 0 0 0 0 0 0 0
0 0 0 0 0 0 0 0 0 0 0 0 0
0 0 0 0 0 0 0 0 0 2 0 0 0
0 0 0 0 0 0 0 0 0 0 0 0 0

0 0 0 0 0 0 0 0 0 0 0 0 0
0 0 0 0 0 0 0
"Viruses;Uroviricota;Caudoviricetes;Caudoviricetes__na;Caudoviricetes__na;Pahexavirus;Pahexavirus_PHL111
M01" 0 0 0 0 0 0 0 0 0 0 5 0 0
0 0 0 0 0 0 0 0 0 0 0 0 0
0 0 0 0 0 0 0 0 0 0 0 0 0
0 0 2 0 0 0 0 0 0 0 0 0 0
0 0 0 0 0 0 0 0 0 0 0 0 0
0 0 0 0 0 0 0
"Viruses;Uroviricota;Caudoviricetes;Caudoviricetes__na;Caudoviricetes__na;Pahexavirus;Pahexavirus_PHL112
N00" 0 0 0 0 0 0 0 0 0 0 0 0 0
0 0 0 0 0 0 0 0 0 0 0 0 0
0 0 0 0 0 0 0 0 0 3 0 0 0
0 0 0 0 0 0 0 0 0 0 0 0 0
0 0 0 0 0 0 0 0 0 0 0 0 0
0 0 0 0 0 0 0
"Viruses;Uroviricota;Caudoviricetes;Caudoviricetes__na;Caudoviricetes__na;Pahexavirus;Pahexavirus_PHL113
M01" 0 0 0 0 0 0 0 0 1 0 4 0 0
0 0 0 0 0 0 0 0 0 0 0 0 0
0 0 0 0 0 0 0 0 0 0 0 0 0
0 0 0 0 0 0 0 0 0 0 0 0 0
0 0 0 0 0 0 0 0 0 0 0 0 0
0 0 0 0 0 0 0
"Viruses;Uroviricota;Caudoviricetes;Caudoviricetes__na;Caudoviricetes__na;Pahexavirus;Pahexavirus_PHL114
L00" 0 0 0 0 0 0 0 0 0 0 20 0 0
0 0 0 0 0 0 0 0 0 0 0 0 0
0 0 0 0 0 0 0 0 0 0 0 0 0
0 0 0 0 0 0 0 0 0 0 0 0 0
0 0 0 0 0 0 0 0 0 0 0 0 0
0 0 0 0 0 0 0
"Viruses;Uroviricota;Caudoviricetes;Caudoviricetes__na;Caudoviricetes__na;Pahexavirus;Pahexavirus_PHL116
M00" 0 0 0 0 0 0 0 0 0 0 21 0 0
0 0 0 0 0 0 0 0 0 0 0 0 0
0 0 0 0 0 0 0 0 0 0 0 0 0
0 0 0 0 0 0 0 2 0 0 0 0 0
0 0 0 0 0 0 0 0 0 0 0 0 0
0 0 0 0 0 0 0
"Viruses;Uroviricota;Caudoviricetes;Caudoviricetes__na;Caudoviricetes__na;Pahexavirus;Pahexavirus_PHL152
M00" 0 0 0 0 0 0 0 0 0 0 9 0 0
0 0 0 0 0 0 0 0 0 0 0 0 0
0 0 0 0 0 0 0 0 0 0 0 0 0
0 0 0 0 0 0 0 0 0 0 0 0 0
0 0 0 0 0 0 0 0 0 0 0 0 0
0 0 0 0 0 0 0
"Viruses;Uroviricota;Caudoviricetes;Caudoviricetes__na;Caudoviricetes__na;Pahexavirus;Pahexavirus_PHL171
M01" 0 0 0 0 1 0 0 0 0 0 0 0 0
0 0 0 0 0 0 0 0 0 0 0 0 0
0 0 0 0 0 0 0 0 0 1 0 0 0
0 0 0 0 0 0 0 0 0 0 0 0 0

0 0 0 0 0 0 0 0 0 0 0 0 0
0 0 0 0 0 0 0
"Viruses;Uroviricota;Caudoviricetes;Caudoviricetes__na;Caudoviricetes__na;Pahexavirus;Pahexavirus_PHL179M00" 0 0 0 0 0 0 0 0 0 0 2 0 0
0 0 0 0 0 0 0 0 0 0 0 0 0
0 0 0 0 0 0 0 0 0 0 0 0 0
0 0 0 0 0 0 0 0 0 0 0 0 0
0 0 0 0 0 0 0 0 0 0 0 0 0
0 0 0 0 0 0 0
"Viruses;Uroviricota;Caudoviricetes;Caudoviricetes__na;Caudoviricetes__na;Pahexavirus;Pahexavirus_PHL301M00" 0 0 0 0 0 0 0 0 0 0 1 0 0
0 0 0 0 0 0 0 0 0 0 0 0 0
0 0 0 0 0 0 0 0 0 0 0 0 0
0 0 0 0 0 0 0 0 0 0 0 0 0
0 0 0 0 0 0 0 0 0 0 0 0 0
0 0 0 0 0 0 0
"Viruses;Uroviricota;Caudoviricetes;Caudoviricetes__na;Caudoviricetes__na;Pahexavirus;Pahexavirus_PHL308M00" 0 0 0 0 0 0 0 0 0 0 28 0 0
0 0 0 0 0 0 0 0 0 0 0 0 0
0 0 0 0 0 0 0 0 0 2 0 0 0
0 0 0 0 0 0 0 0 0 0 0 0 0
0 0 0 0 0 0 0 1 0 0 0 0 0
0 0 0 0 0 0 0
"Viruses;Uroviricota;Caudoviricetes;Caudoviricetes__na;Caudoviricetes__na;Pahexavirus;Pahexavirus_procrass1" 0 0 0 0 0 0 0 0 0 0 0 0 0
0 0 0 0 0 0 0 0 0 0 0 0 0
0 0 0 0 0 0 0 0 0 0 0 0 0
0 0 1 0 0 0 0 0 0 0 0 0 0
0 0 0 0 0 0 0 0 0 0 0 0 0
0 0 0 0 0 0 0
"Viruses;Uroviricota;Caudoviricetes;Caudoviricetes__na;Caudoviricetes__na;Pahexavirus;Pahexavirus_SKKY"
0 0 0 0 0 0 0 0 0 0 0 0 0
0 0 0 0 0 0 0 0 0 0 0 0 0
0 0 0 0 0 0 0 0 0 1 0 0 0
0 0 0 0 0 0 0 0 0 0 0 0 0
0 0 0 0 0 0 0 0 0 0 0 0 0
0 0 0 0 0 0 0
"Viruses;Uroviricota;Caudoviricetes;Caudoviricetes__na;Caudoviricetes__na;Pahexavirus;Pahexavirus_solid"
0 0 0 0 0 0 0 0 0 0 31 0 0
0 0 0 0 0 0 0 0 0 0 0 0 0
0 0 0 0 0 0 0 0 0 0 0 0 0
0 0 1 0 0 0 0 0 0 0 0 0 0
0 0 0 0 0 0 0 0 0 0 0 0 0
0 0 0 0 0 0 0
"Viruses;Uroviricota;Caudoviricetes;Caudoviricetes__na;Caudoviricetes__na;Pahexavirus;Pahexavirus_stormborn" 0 0 0 0 0 0 0 0 0 0 2 0 0
0 0 0 0 0 0 0 0 0 0 0 0 0
0 0 0 0 0 0 0 0 0 3 0 0 0
0 0 0 0 0 0 0 0 0 0 0 0 0

0 0 0 0 0 0 0 0 0 0 0 0 0
0 0 0 0 0 0 0
"Viruses;Uroviricota;Caudoviricetes;Caudoviricetes__na;Caudoviricetes__na;Pahexavirus;Propionibacterium_p
hage_Aquarius"0 0 0 0 0 0 0 0 0 0 0 0
0 0 0 0 0 0 0 0 0 0 0 0 0
0 0 0 0 0 0 0 0 0 0 3 0 0
0 0 0 0 0 0 0 0 0 0 0 0 0
0 0 0 0 0 0 0 0 0 0 0 0 0
0 0 0 0 0 0 0 0
"Viruses;Uroviricota;Caudoviricetes;Caudoviricetes__na;Caudoviricetes__na;Pahexavirus;Propionibacterium_p
hage_BruceLethal" 0 0 0 0 0 0 0 0 0 0 5
0 0 0 0 0 0 0 0 0 0 0 0 0
0 0 0 0 0 0 0 0 0 0 0 0 0
0 0 0 0 0 0 0 0 0 0 1 0 0
0 0 0 0 0 0 0 0 0 0 0 0 0
0 0 0 0 0 0 0 0 0
"Viruses;Uroviricota;Caudoviricetes;Caudoviricetes__na;Caudoviricetes__na;Pahexavirus;Propionibacterium_p
hage_Cota" 0 0 0 0 0 0 0 0 0 0 7 0
0 0 0 0 0 0 0 0 0 0 0 0 0
0 0 0 0 0 0 0 0 0 0 0 0 0
0 0 0 0 0 0 0 0 0 0 0 0 0
0 0 0 0 0 0 0 0 0 0 0 0 0
0 0 0 0 0 0 0 0
"Viruses;Uroviricota;Caudoviricetes;Caudoviricetes__na;Caudoviricetes__na;Pahexavirus;Propionibacterium_p
hage_DrParker"0 0 0 0 0 0 0 0 0 0 4 0
0 0 0 0 0 0 0 0 0 0 0 0 0
0 0 0 0 0 0 0 0 0 0 1 0 0
0 0 0 0 0 0 0 0 0 0 0 0 0
0 0 0 0 0 0 0 0 0 0 0 0 0
0 0 0 0 0 0 0 0
"Viruses;Uroviricota;Caudoviricetes;Caudoviricetes__na;Caudoviricetes__na;Pahexavirus;Propionibacterium_p
hage_Enoki" 0 0 0 0 0 0 0 0 0 0 0 0
0 0 0 0 0 0 0 0 0 0 0 0 0
0 0 0 0 0 0 0 0 0 0 3 0 0
0 0 0 0 0 0 0 0 0 1 0 0 0
0 0 0 0 0 0 0 0 0 0 0 0 0
0 0 0 0 0 0 0 0
"Viruses;Uroviricota;Caudoviricetes;Caudoviricetes__na;Caudoviricetes__na;Pahexavirus;Propionibacterium_p
hage_Leviosa" 0 0 0 0 1 0 0 0 0 0 7 0
0 0 0 0 0 0 0 0 0 0 0 0 0
0 0 0 0 0 0 0 0 0 1 3 0 0
0 0 0 0 0 0 0 0 0 0 0 0 0
0 0 0 0 0 0 0 0 0 0 0 0 0
0 0 0 0 0 0 0 0
"Viruses;Uroviricota;Caudoviricetes;Caudoviricetes__na;Caudoviricetes__na;Pahexavirus;Propionibacterium_p
hage_LilBandit"0 0 0 0 0 0 0 0 0 0 11 0
0 0 0 0 0 0 0 0 0 0 0 0 0
0 0 0 0 0 0 0 0 0 0 0 0 0
0 0 0 1 0 0 0 0 0 0 0 0 0

0 0 0 0 0 0 0 0 0 0 0 0 0
0 0 0 0 0 0 0 0
"Viruses;Uroviricota;Caudoviricetes;Caudoviricetes__na;Caudoviricetes__na;Pahexavirus;Propionibacterium_p
hage_MEAK" 0 0 0 0 0 0 0 0 0 0 1 0
0 0 0 0 0 0 0 0 0 0 0 0 0
0 0 0 0 0 0 0 0 0 0 0 0 0
0 0 0 0 0 0 0 0 0 0 0 0 0
0 0 0 0 0 0 0 0 0 0 0 0 0
0 0 0 0 0 0 0 0
"Viruses;Uroviricota;Caudoviricetes;Caudoviricetes__na;Caudoviricetes__na;Pahexavirus;Propionibacterium_p
hage_PA1-14" 0 0 0 0 0 0 0 0 0 0 3 0
0 0 0 0 0 0 0 0 0 0 0 0 0
0 0 0 0 0 0 0 0 0 0 0 0 0
0 0 0 0 0 0 0 0 0 0 0 0 0
0 0 0 0 0 0 0 0 0 0 0 0 0
0 0 0 0 0 0 0 0
"Viruses;Uroviricota;Caudoviricetes;Caudoviricetes__na;Caudoviricetes__na;Pahexavirus;Propionibacterium_p
hage_pa27" 0 0 0 0 0 0 0 0 0 0 4 0
0 0 0 0 0 0 0 0 0 0 0 0 0
0 0 0 0 0 0 0 0 0 0 0 0 0
0 0 0 0 0 0 0 0 0 1 0 0 0
0 0 0 0 0 0 0 0 0 0 0 0 0
0 0 0 0 0 0 0 0
"Viruses;Uroviricota;Caudoviricetes;Caudoviricetes__na;Caudoviricetes__na;Pahexavirus;Propionibacterium_p
hage_pa28" 0 0 0 0 0 0 0 0 0 0 10 0
0 0 0 0 0 0 0 0 0 0 0 0 0
0 0 0 0 0 0 0 0 0 0 0 0 0
0 0 0 0 0 0 0 0 0 0 0 0 0
0 0 0 0 0 0 0 0 0 0 0 0 0
0 0 0 0 0 0 0 0
"Viruses;Uroviricota;Caudoviricetes;Caudoviricetes__na;Caudoviricetes__na;Pahexavirus;Propionibacterium_p
hage_pa35" 0 0 0 0 0 0 0 0 0 0 4 0
0 0 0 0 0 0 0 0 0 0 0 0 0
0 0 0 0 0 0 0 0 0 0 0 0 0
0 0 0 0 0 0 0 0 3 0 0 0 0
0 0 0 0 0 0 0 0 0 0 0 0 0
0 0 0 0 0 0 0 0
"Viruses;Uroviricota;Caudoviricetes;Caudoviricetes__na;Caudoviricetes__na;Pahexavirus;Propionibacterium_p
hage_pa63" 0 0 0 0 0 0 0 0 0 0 1 0
0 0 0 0 0 0 0 0 0 0 0 0 0
0 0 0 0 0 0 0 0 0 0 1 0 0
0 0 0 0 0 0 0 0 0 0 0 0 0
0 0 0 0 0 0 0 0 0 0 0 0 0
0 0 0 0 0 0 0 0
"Viruses;Uroviricota;Caudoviricetes;Caudoviricetes__na;Caudoviricetes__na;Pahexavirus;Propionibacterium_p
hage_PAC3" 0 0 0 0 0 0 0 0 0 0 2 0
0 0 0 0 0 0 0 0 0 0 0 0 0
0 0 0 0 0 0 0 0 0 0 0 0 0
0 0 0 0 0 0 0 0 0 0 0 0 0

0	0	0	0	0	0	0	0	0	0	0	0	0
0	0	0	0	0	0	0	0
"Viruses;Uroviricota;Caudoviricetes;Caudoviricetes__na;Caudoviricetes__na;Pahexavirus;Propionibacterium_phage_PAC9"	0	0	0	0	0	0	0	0	0	0	16	0
0	0	0	0	0	0	0	0	0	0	0	0	0
0	0	0	0	0	0	0	0	0	0	0	0	0
0	0	0	0	0	0	0	0	0	0	0	0	0
0	0	0	0	0	0	0	0	0	0	0	0	0
0	0	0	0	0	0	0	0
"Viruses;Uroviricota;Caudoviricetes;Caudoviricetes__na;Caudoviricetes__na;Pahexavirus;Propionibacterium_phage_PacnesP1"0	0	0	0	0	0	0	0	0	0	1	0
0	0	0	0	0	0	0	0	0	0	0	0	0
0	0	0	0	0	0	0	0	0	0	0	0	0
0	0	0	0	0	0	0	0	0	0	0	0	0
0	0	0	0	0	0	0	0	0	0	0	0	0
0	0	0	0	0	0	0	0
"Viruses;Uroviricota;Caudoviricetes;Caudoviricetes__na;Caudoviricetes__na;Pahexavirus;Propionibacterium_phage_PaP11-13"0	0	0	0	0	0	0	0	0	0	3	0
0	0	0	0	0	0	0	0	0	0	0	0	0
0	0	0	0	0	0	0	0	0	0	1	0	0
0	0	0	0	0	0	0	0	0	0	0	0	0
0	0	0	0	0	0	0	0	0	0	0	0	0
0	0	0	0	0	0	0	0
"Viruses;Uroviricota;Caudoviricetes;Caudoviricetes__na;Caudoviricetes__na;Pahexavirus;Propionibacterium_phage_QueenBey"	0	0	0	0	0	0	0	0	0	0	0
0	0	0	0	0	0	0	0	0	0	0	0	0
0	0	0	0	0	0	0	0	0	0	0	0	0
0	0	0	0	0	0	0	0	0	0	1	0	0
0	0	0	0	0	0	0	0	0	0	0	0	0
0	0	0	0	0	0	0	0	0
"Viruses;Uroviricota;Caudoviricetes;Caudoviricetes__na;Caudoviricetes__na;Pahexavirus;Propionibacterium_phage_TCUCAP1"	0	0	0	0	0	0	0	0	0	0	5
0	0	0	0	0	0	0	0	0	0	0	0	0
0	0	0	0	0	0	0	0	0	0	0	2	0
0	0	0	0	0	0	0	0	0	0	0	0	0
0	0	0	0	0	0	0	0	0	0	0	0	0
0	0	0	0	0	0	0	0	0
"Viruses;Uroviricota;Caudoviricetes;Caudoviricetes__na;Caudoviricetes__na;Pakpunavirus;Pakpunavirus__uc"
0	0	0	0	0	4	0	0	0	0	0	0	0
0	0	0	0	0	0	0	0	0	0	0	0	0
0	0	0	0	0	0	0	0	0	0	0	0	0
0	0	0	0	0	0	0	0	0	0	0	0	0
0	0	0	0	0	0	0	0	0	0	0	0	0
0	0	0	0	0	0	0
"Viruses;Uroviricota;Caudoviricetes;Caudoviricetes__na;Caudoviricetes__na;Pakpunavirus;Pseudomonas_phage_EM"	0	0	0	0	0	0	0	0	0	0	0	0	0
0	0	0	0	0	0	0	0	0	0	0	0	0
0	0	0	0	0	0	0	0	0	0	0	0	0
0	0	0	0	0	0	0	0	2	0	0	0	0

0 0 0 0 0 0 0 0 0 0 0 0 0
0 0 0 0 0 0 0
"Viruses;Uroviricota;Caudoviricetes;Caudoviricetes__na;Caudoviricetes__na;Parlovirus;Parlovirus_parlo"
0 0 0 0 0 0 0 0 0 0 0 0 0
0 0 0 0 0 0 0 0 0 0 1 0 0
0 0 0 0 0 0 0 0 0 0 0 0 0
0 0 0 0 0 0 0 0 0 0 0 0 0
0 0 0 0 0 0 0 0 0 0 0 0 0
0 0 0 0 0 0 0
"Viruses;Uroviricota;Caudoviricetes;Caudoviricetes__na;Caudoviricetes__na;Pbunavirus;Pbunavirus__uc"
0 0 0 0 0 0 0 0 0 0 0 0 0
0 0 0 0 0 0 0 0 0 0 0 0 0
0 0 0 0 0 0 0 0 0 0 0 0 0
0 0 0 0 0 0 0 0 3 0 0 0 0
0 0 0 0 0 0 0 0 0 0 0 0 0
0 0 0 0 0 0 0
"Viruses;Uroviricota;Caudoviricetes;Caudoviricetes__na;Caudoviricetes__na;Peeveelvirus;Peeveelvirus_tp3101"
0 0 0 0 0 0 0 0 0 0 0 0 0
0 0 0 0 0 0 0 0 0 0 0 0 0
0 0 0 0 0 0 0 0 0 0 0 0 0
0 0 1 0 0 0 0 0 0 0 0 0 0
0 0 0 0 0 0 0 0 0 0 0 0 0
0 0 0 0 0 0 0
"Viruses;Uroviricota;Caudoviricetes;Caudoviricetes__na;Caudoviricetes__na;Pepyhexavirus;Pepyhexavirus__uc
" 0 0 0 0 0 1 0 0 0 0 0 0 0
0 0 0 0 0 0 0 0 0 0 0 0 0
0 0 0 0 0 0 0 0 1 0 0 0 0
0 0 0 0 0 0 0 0 0 0 0 0 0
0 0 0 0 0 0 0 0 0 0 0 0 0
0 0 0 0 0 0 0
"Viruses;Uroviricota;Caudoviricetes;Caudoviricetes__na;Caudoviricetes__na;Pepyhexavirus;Pepyhexavirus_poc
o6" 0 0 0 0 2 1 0 0 0 0 0 0 0
0 0 0 0 0 0 0 0 0 0 0 0 0
0 0 0 0 0 0 0 0 0 0 0 0 0
0 0 9 0 0 0 0 0 0 0 0 0 0
0 0 0 0 0 0 0 0 0 0 0 0 0
0 0 0 0 0 0 0
"Viruses;Uroviricota;Caudoviricetes;Caudoviricetes__na;Caudoviricetes__na;Petsuvirus;Pseudomonas_phage_O
BP" 0 0 0 2 0 0 0 0 0 0 0 0 0
0 0 0 0 0 0 0 0 0 0 0 0 0
0 0 0 0 0 0 0 0 0 0 0 0 0
0 0 0 0 0 0 0 0 0 0 0 0 0
0 0 0 0 0 0 0 0 0 0 0 0 0
0 0 0 0 0 0 0
"Viruses;Uroviricota;Caudoviricetes;Caudoviricetes__na;Caudoviricetes__na;Phabquatrovirus;Phabquatrovirus_
PHB04" 0 0 0 1 0 0 0 0 0 0 0 0 0
0 0 0 0 0 0 0 0 0 0 0 0 0
0 0 0 0 0 0 0 0 0 0 0 0 0
0 0 0 0 0 0 0 0 0 0 0 0 0

0 0 0 0 0 0 0 0 0 0 0 0 0
0 0 0 0 0 0 0
"Viruses;Uroviricota;Caudoviricetes;Caudoviricetes__na;Caudoviricetes__na;Phapecoctavirus;Phapecoctavirus_
_uc" 0 0 0 0 0 0 0 0 0 0 0 0 0
0 0 0 0 0 0 0 0 0 0 0 0 0
0 0 0 0 0 0 0 0 0 0 0 0 0
0 0 0 0 0 0 0 0 1 0 0 0 0
0 0 0 0 0 0 0 0 0 0 0 0 0
0 0 0 0 0 0 0
"Viruses;Uroviricota;Caudoviricetes;Caudoviricetes__na;Caudoviricetes__na;Plateaulakevirus;Aeromonas_phag
e_phiA047" 0 0 1 0 0 0 0 0 0 2 0 0
0 0 0 0 0 0 0 0 0 0 0 0 0
0 0 0 0 0 0 0 0 0 0 0 0 0
0 0 0 0 0 0 0 0 0 0 0 0 0
0 0 0 0 0 0 0 0 0 0 0 0 0
0 0 0 0 0 0 0 0
"Viruses;Uroviricota;Caudoviricetes;Caudoviricetes__na;Caudoviricetes__na;Plateaulakevirus;Plateaulakevirus_
_uc" 0 0 0 0 0 0 0 0 0 0 0 0 0
0 0 0 0 0 0 0 0 0 0 0 0 0
0 0 0 0 0 0 0 0 0 0 0 0 0
0 0 0 0 0 0 0 0 2 0 0 0 0
0 0 0 0 0 0 0 0 0 0 0 0 0
0 0 0 0 0 0 0
"Viruses;Uroviricota;Caudoviricetes;Caudoviricetes__na;Caudoviricetes__na;Plateaulakevirus;Pseudaeromonas
_phage_vB_PpeM__KLEP7" 0 0 0 0 1 14 0 0 0 0
0 0 0 0 0 0 0 0 0 0 0 0 0
0 0 0 0 0 0 0 0 0 0 0 0 0
0 0 0 0 0 0 0 0 0 0 0 0 0
0 0 0 0 0 0 0 0 0 0 0 0 0
0 0 0 0 0 0 0 0 4 0
"Viruses;Uroviricota;Caudoviricetes;Caudoviricetes__na;Caudoviricetes__na;Polybotosvirus;Polybotosvirus_At
uph07" 0 0 2 0 5 0 0 0 0 0 0 0 0
0 0 0 0 0 0 0 0 0 0 0 0 0
0 0 0 0 0 0 0 0 0 0 0 0 0
0 0 0 0 0 0 0 0 0 0 0 0 0
0 0 0 0 0 0 0 0 0 0 0 0 0
0 0 0 0 0 0 0
"Viruses;Uroviricota;Caudoviricetes;Caudoviricetes__na;Caudoviricetes__na;Popoffvirus;Popoffvirus_pv56"
0 0 1 0 0 0 0 0 0 0 0 0 0
0 0 0 0 0 0 0 0 0 0 0 0 0
0 0 0 0 0 0 0 0 0 0 0 0 0
0 0 0 0 0 0 0 0 1 0 0 0 0
0 0 0 0 0 0 0 0 0 0 0 0 0
0 0 0 0 0 0 0
"Viruses;Uroviricota;Caudoviricetes;Caudoviricetes__na;Caudoviricetes__na;Pukovnikvirus;Pukovnikvirus__uc
" 0 0 0 1 0 0 0 0 0 0 0 0 0
0 0 0 0 0 0 0 0 0 0 0 0 0
0 0 0 0 0 0 0 0 0 0 0 0 0
0 0 0 0 0 0 0 0 0 0 0 0 0

0 0 0 0 0 0 0 0 0 0 0 0 0
0 0 0 0 0 0 0
"Viruses;Uroviricota;Caudoviricetes;Caudoviricetes__na;Caudoviricetes__na;Pukovnikvirus;Pukovnikvirus_ironman" 0 0 0 0 0 0 0 0 0 0 0 0 0
0 0 0 0 0 0 0 0 0 0 0 0 0
0 0 0 0 0 0 0 0 0 0 0 0 0
0 0 0 0 0 0 0 0 0 0 0 0 0
0 0 1 0 0 0 0 0 0 0 0 0 0
0 0 0 0 0 0 0
"Viruses;Uroviricota;Caudoviricetes;Caudoviricetes__na;Caudoviricetes__na;Qingdaovirus;Pseudoalteromonas_phage_PH357" 0 0 0 0 0 0 0 0 0 0 0 0
0 0 0 0 0 0 0 0 0 0 0 0 0
0 0 0 0 0 0 0 0 0 0 0 0 0
0 0 1 0 0 0 0 0 0 0 0 0 0
0 0 0 0 0 0 0 0 0 0 0 0 0
0 0 0 0 0 0 0 0
"Viruses;Uroviricota;Caudoviricetes;Caudoviricetes__na;Caudoviricetes__na;Questintvirus;Questintvirus_Q54"
0 0 0 0 0 0 0 0 0 0 0 0 0
0 1 0 0 0 0 0 0 0 0 0 0 0
0 0 0 0 0 0 0 0 0 0 0 0 0
0 0 0 0 0 0 0 0 0 0 0 0 0
0 0 0 0 0 0 0 0 0 0 0 0 0
0 0 0 0 0 0 0
"Viruses;Uroviricota;Caudoviricetes;Caudoviricetes__na;Caudoviricetes__na;Raharianneviruss;Raharianneviruss_raharianne" 0 0 0 0 8 0 0 0 0 0 0 0
0 0 0 0 0 0 0 0 0 0 0 0 0
0 0 0 0 0 0 0 0 0 0 0 0 0
0 0 0 0 0 0 0 0 0 0 0 0 0
0 0 0 0 0 0 0 0 0 0 0 0 0
0 0 0 0 0 0 0 0
"Viruses;Uroviricota;Caudoviricetes;Caudoviricetes__na;Caudoviricetes__na;Rauchvirus;Rauchvirus__uc"
0 0 0 0 0 0 0 0 0 1 0 0 0
0 0 0 0 0 0 0 0 0 0 0 0 0
0 0 0 0 0 0 0 0 0 0 0 0 0
0 0 0 0 0 0 0 0 0 0 0 0 0
0 0 0 0 0 0 0 0 0 0 0 0 0
0 0 0 0 0 0 0
"Viruses;Uroviricota;Caudoviricetes;Caudoviricetes__na;Caudoviricetes__na;Ravarandavirus;Ravarandavirus__uc" 0 0 0 0 0 0 0 0 0 0 0 0 0
0 0 0 0 0 0 0 0 0 0 0 0 0
0 0 0 0 0 0 0 0 0 2 0 0 0
0 0 0 0 0 0 0 0 0 0 0 0 0
0 0 0 0 0 0 0 0 0 0 0 0 0
0 0 0 0 0 0 0
"Viruses;Uroviricota;Caudoviricetes;Caudoviricetes__na;Caudoviricetes__na;Rigallicvirus;Rhizobium_phage_RHph_X2_24" 0 1 0 1 11 0 0 0 0 0 0 0
0 0 0 0 0 0 0 0 0 0 0 0 0
0 0 0 0 0 0 0 0 0 0 0 0 0
0 0 0 0 0 0 0 0 0 1 0 0 0

0 0 0 0 0 0 0 0 0 0 0 0 0
0 0 0 0 0 0 0 0
"Viruses;Uroviricota;Caudoviricetes;Caudoviricetes__na;Caudoviricetes__na;Rigallicvirus;Rigallicvirus_P106B"
1 0 0 0 1 2 0 0 0 0 0 0 0
0 0 0 0 0 0 0 0 0 0 0 0 0
0 0 0 0 0 0 0 0 0 0 0 0 0
0 0 0 0 0 0 0 0 0 0 0 0 0
0 0 0 0 0 0 0 0 0 0 0 0 0
0 0 0 0 0 0 0
"Viruses;Uroviricota;Caudoviricetes;Caudoviricetes__na;Caudoviricetes__na;Rimavirus;Rimavirus__uc" 0
0 0 0 0 0 0 0 0 0 0 0 0 0
0 0 0 0 0 0 0 0 0 0 0 0 0
0 0 0 0 0 0 0 28 0 0 0 0 0
0 0 0 0 0 0 0 0 0 0 0 0 0
0 0 0 0 0 0 0 0 0 0 0 0 0
0 0 0 0 0 0
"Viruses;Uroviricota;Caudoviricetes;Caudoviricetes__na;Caudoviricetes__na;Rimavirus;Streptomyces_phage_GirlPower" 0 0 0 0 0 0 0 0 0 0 0 0
0 0 0 0 0 0 0 0 0 0 0 0 0
0 0 0 0 0 0 0 0 0 0 0 0 0
0 0 0 0 0 0 1 0 0 0 0 0 0
0 0 0 0 0 0 0 0 0 0 0 0 0
0 0 0 0 0 0 0 0
"Viruses;Uroviricota;Caudoviricetes;Caudoviricetes__na;Caudoviricetes__na;Ripduovirus;Ripduovirus_RP12"
0 0 0 0 1 1 0 0 0 0 0 0 0
0 0 0 0 0 0 0 0 0 0 0 0 0
0 0 0 0 0 0 0 0 0 0 0 0 0
0 0 0 0 0 0 0 0 0 0 0 0 0
0 0 0 0 0 0 0 0 0 0 0 0 0
0 0 0 0 0 0 0
"Viruses;Uroviricota;Caudoviricetes;Caudoviricetes__na;Caudoviricetes__na;Risingsunvirus;Risingsunvirus_risingsun" 0 0 0 0 0 0 0 0 0 0 0 0 0
0 0 0 0 0 0 0 0 0 0 0 0 0
0 0 0 0 0 0 0 0 0 0 0 0 0
0 0 5 0 0 0 0 0 0 0 0 0 0
0 0 0 0 0 0 0 0 0 0 0 0 0
0 0 0 0 0 0 0
"Viruses;Uroviricota;Caudoviricetes;Caudoviricetes__na;Caudoviricetes__na;Ronaldovirus;Ronaldovirus__uc"
0 0 0 0 0 27 0 0 0 3 0 0 0
0 0 0 0 0 0 0 0 0 0 0 0 0
0 0 0 0 0 0 0 0 0 0 0 0 0
0 0 0 0 0 0 0 0 0 0 0 0 0
0 0 0 0 0 0 0 0 0 0 0 0 0
0 0 0 0 0 0 0
"Viruses;Uroviricota;Caudoviricetes;Caudoviricetes__na;Caudoviricetes__na;Salmondvirus;Salmondvirus__uc"
0 0 0 0 0 0 0 0 0 0 0 0 0
0 0 0 0 0 0 0 0 0 0 0 0 0
0 0 0 0 0 0 0 1 0 0 0 0 0
0 0 0 0 0 0 0 0 0 0 0 0 0

0 0 0 0 0 0 0 0 0 0 0 0 0
0 0 0 0 0 0 0
"Viruses;Uroviricota;Caudoviricetes;Caudoviricetes__na;Caudoviricetes__na;Samistivirus;Samistivirus_daubens
ki" 0 0 0 0 0 12 0 0 0 0 0 0 0
0 0 0 0 0 0 0 0 0 0 0 0 0
0 0 0 0 0 0 0 0 0 0 0 0 0
0 0 0 0 0 0 0 0 1 0 0 0 0
0 0 0 0 0 0 0 0 0 0 0 0 0
0 0 0 0 0 0 0
"Viruses;Uroviricota;Caudoviricetes;Caudoviricetes__na;Caudoviricetes__na;Samistivirus;Samistivirus_mildred
21" 0 0 0 1 0 5 0 0 0 0 0 0 0
0 0 0 0 0 0 0 0 0 0 0 0 0
0 0 0 0 0 0 0 0 0 0 0 0 0
0 0 0 0 0 0 0 0 0 0 0 0 0
0 0 0 0 0 0 0 0 0 0 0 0 0
0 0 0 0 0 0 0
"Viruses;Uroviricota;Caudoviricetes;Caudoviricetes__na;Caudoviricetes__na;Samunavirus;Samunavirus__uc"
0 0 0 0 2 0 0 0 0 0 0 0 0
0 0 0 0 0 0 0 0 0 0 0 0 0
0 0 0 0 0 0 0 0 0 0 0 0 0
0 0 0 0 0 0 0 0 0 0 0 0 0
0 0 0 0 0 0 0 0 0 0 0 0 0
0 0 0 0 0 0 0
"Viruses;Uroviricota;Caudoviricetes;Caudoviricetes__na;Caudoviricetes__na;Sandinevirus;Sandinevirus_BK5T"
0 0 0 0 0 0 0 0 0 0 0 0 0
0 0 0 0 0 0 0 0 0 0 0 0 0
0 0 0 0 0 1 0 0 0 0 0 0 0
0 0 0 0 0 0 0 0 0 0 0 0 0
0 0 1 1 0 0 0 0 0 0 0 0 0
0 0 0 0 0 0 0
"Viruses;Uroviricota;Caudoviricetes;Caudoviricetes__na;Caudoviricetes__na;Sarumanvirus;Sarumanvirus__uc"
0 0 0 0 0 0 0 0 0 0 0 0 0
0 0 0 0 0 0 1 0 0 0 0 0 0
0 0 0 0 0 0 0 0 0 0 0 0 0
0 0 0 0 0 0 0 0 0 0 0 0 0
0 0 0 0 0 0 0 0 0 0 0 0 0
0 0 0 0 0 0 0
"Viruses;Uroviricota;Caudoviricetes;Caudoviricetes__na;Caudoviricetes__na;Scapunavirus;Scapunavirus__uc"
0 0 0 0 0 1 0 0 0 0 0 0 0
0 0 0 0 0 0 0 0 0 0 0 0 0
0 0 0 0 0 0 0 0 0 0 0 0 0
0 0 0 0 0 0 0 0 0 0 0 0 0
0 0 0 0 0 0 0 0 0 0 0 0 0
0 0 0 0 0 0 0
"Viruses;Uroviricota;Caudoviricetes;Caudoviricetes__na;Caudoviricetes__na;Scapunavirus;Scapunavirus_scap1"
0 0 0 1 0 3 0 0 0 0 0 0 0
0 0 0 0 0 0 0 0 0 0 0 0 0
0 0 0 0 0 0 0 0 0 0 0 0 0
0 0 0 0 0 0 0 0 0 0 0 0 0

0 0 0 0 0 0 0 0 0 0 0 0 0
0 0 0 0 0 0 0
"Viruses;Uroviricota;Caudoviricetes;Caudoviricetes__na;Caudoviricetes__na;Scapunavirus;Streptomyces_phage
_Doxi13" 0 0 0 0 0 5 0 0 0 0 0 0
0 0 0 0 0 0 0 0 0 0 0 0 0
0 0 0 0 0 0 0 0 0 0 0 0 0
0 0 0 0 0 0 0 0 0 0 0 0 0
0 0 0 0 0 0 0 0 0 0 0 0 0
0 0 0 0 0 0 0 0
"Viruses;Uroviricota;Caudoviricetes;Caudoviricetes__na;Caudoviricetes__na;Secretariatvirus;Gordonia_phage_S
chwartz33" 0 0 0 0 1 0 0 0 0 0 0 0
0 0 0 0 0 0 0 0 0 0 0 0 0
0 0 0 0 0 0 0 0 0 0 0 0 0
0 0 0 0 0 0 0 0 0 0 0 0 0
0 0 0 0 0 0 0 0 0 0 0 0 0
0 0 0 0 0 0 0 0
"Viruses;Uroviricota;Caudoviricetes;Caudoviricetes__na;Caudoviricetes__na;Secretariatvirus;Secretariatvirus__
uc" 0 0 0 0 0 8 0 0 0 0 0 0 0
0 0 0 0 0 0 0 0 0 0 0 0 0
0 0 0 0 0 0 0 0 0 0 0 0 0
0 0 0 0 0 0 0 0 0 0 0 0 0
0 0 0 0 0 0 0 0 0 0 0 0 0
0 0 0 0 0 0 0
"Viruses;Uroviricota;Caudoviricetes;Caudoviricetes__na;Caudoviricetes__na;Seunavirus;Seunavirus_GAP31"
0 0 0 0 0 2 0 0 0 0 0 0 0
0 0 0 0 0 0 0 0 0 0 0 0 0
0 0 0 0 0 0 0 0 0 0 0 0 0
0 0 0 0 0 0 0 0 6 0 0 0 0
0 0 0 0 0 0 0 0 0 0 0 0 0
0 0 0 0 0 0 0
"Viruses;Uroviricota;Caudoviricetes;Caudoviricetes__na;Caudoviricetes__na;Sextaecvirus;Sextaecvirus__uc"
0 0 0 0 0 0 0 0 0 0 2 0 0
0 0 0 0 0 0 0 0 0 0 0 0 0
0 0 0 0 0 0 0 0 0 0 0 0 0
0 0 0 0 0 0 0 0 0 0 0 0 0
0 0 0 0 0 0 0 0 0 0 0 0 0
0 0 0 0 0 0 0
"Viruses;Uroviricota;Caudoviricetes;Caudoviricetes__na;Caudoviricetes__na;Sextaecvirus;Staphylococcus_phag
e_PMBT8" 0 0 0 0 6 0 0 0 0 0 0 0
0 0 0 0 0 0 0 0 0 0 0 0 0
0 0 0 0 0 0 0 0 0 0 0 0 0
0 0 0 0 0 0 0 0 0 0 0 0 0
0 0 0 0 0 0 0 0 0 0 0 0 0
0 0 0 0 0 0 0 0
"Viruses;Uroviricota;Caudoviricetes;Caudoviricetes__na;Caudoviricetes__na;Sextaecvirus;Staphylococcus_phag
e_vB_SepS_BE02" 0 0 0 0 0 0 0 0 0 0 0
0 0 0 0 0 0 0 0 0 0 0 0 0
0 0 0 0 0 0 0 0 0 0 0 0 0
0 0 0 0 0 0 0 0 0 0 0 0 0

0 0 0 0 0 0 0 0 0 69 0 0 0
0 0 0 0 0 0 0 0 0
"Viruses;Uroviricota;Caudoviricetes;Caudoviricetes__na;Caudoviricetes__na;Sextaecvirus;Staphylococcus_phag
e_vB_SepS_BE22" 1 0 0 0 0 0 0 0 0 0 0
0 0 0 0 0 0 0 0 0 0 0 0 0
0 0 0 0 0 0 0 0 0 0 0 2 0
0 0 0 0 0 0 0 0 0 0 0 0 0
0 0 0 0 0 0 0 0 0 0 0 0 0
0 0 0 0 0 0 0 0 0
"Viruses;Uroviricota;Caudoviricetes;Caudoviricetes__na;Caudoviricetes__na;Shandongvirus;Shandongvirus_H1
01" 0 0 0 0 9 0 0 0 0 0 0 0 0
0 0 0 0 0 0 0 0 0 0 0 0 0
0 0 0 0 0 0 0 0 0 0 0 0 0
0 0 0 0 0 0 0 0 0 0 0 0 0
0 0 0 0 0 0 0 0 0 0 0 0 0
0 0 0 0 0 0 0
"Viruses;Uroviricota;Caudoviricetes;Caudoviricetes__na;Caudoviricetes__na;Shirahamavirus;Shirahamavirus_P
Tm1" 0 0 0 0 0 0 0 0 0 0 0 0 0
0 0 0 0 0 0 0 0 0 0 0 0 0
0 0 0 0 0 0 0 0 0 0 0 0 0
0 0 0 0 0 0 0 0 0 0 0 0 0
0 0 0 0 0 0 0 0 0 0 0 0 0
0 0 0 0 0 1 0
"Viruses;Uroviricota;Caudoviricetes;Caudoviricetes__na;Caudoviricetes__na;Skarprettervirus;Skarprettervirus_
skarpretter" 0 0 0 0 0 0 0 0 0 0 0 0
0 0 0 0 0 0 0 0 0 0 0 0 0
0 0 0 0 0 0 0 0 0 0 0 0 0
0 0 0 0 0 0 0 0 0 0 0 0 0
6 0 0 0 0 0 0 0 0 0 0 0 0
0 0 0 0 0 0 0 0
"Viruses;Uroviricota;Caudoviricetes;Caudoviricetes__na;Caudoviricetes__na;Skunavirus;Lactococcus_lactis_pha
ge_645" 0 0 0 0 0 0 0 0 0 0 0 0 0
0 0 0 0 0 0 0 0 0 0 0 0 0
0 0 0 0 0 0 0 0 0 0 0 0 0
0 0 0 0 0 0 0 0 0 0 0 0 0
0 0 0 0 0 0 0 0 1 1 0 0 0
0 0 0 0 0 0 0
"Viruses;Uroviricota;Caudoviricetes;Caudoviricetes__na;Caudoviricetes__na;Skunavirus;Lactococcus_phage_93
6_group_phage_Phi129" 0 0 0 0 0 0 0 0 0 0 0
0 0 0 0 0 0 0 0 0 0 0 0 0
0 0 0 0 0 0 0 0 0 0 13 0 0
0 0 0 0 0 0 0 0 0 0 0 0 0
0 0 0 0 0 1 0 0 0 0 0 0 0
0 0 0 0 0 0 0 0 0
"Viruses;Uroviricota;Caudoviricetes;Caudoviricetes__na;Caudoviricetes__na;Skunavirus;Lactococcus_phage_93
6_group_phage_Phi4" 0 0 0 0 0 0 0 0 0 0 0
0 0 0 0 0 0 0 0 0 0 0 0 0
0 0 0 0 0 0 0 0 0 0 0 0 0
0 0 0 0 0 0 0 0 0 0 0 0 0

0 0 0 0 0 0 0 0 0 0 3 0 0
0 0 0 0 0 0 0 0 0
"Viruses;Uroviricota;Caudoviricetes;Caudoviricetes__na;Caudoviricetes__na;Skunavirus;Lactococcus_phage_93
6_group_phage_PhiA1127" 0 0 0 0 0 0 0 0 0 0
0 0 0 0 0 0 0 0 0 0 0 0 0
0 0 0 0 0 0 0 0 0 0 0 4 0
0 0 0 0 0 0 0 0 0 0 0 0 0
0 0 0 0 0 0 0 0 0 0 0 3 2
0 0 0 0 0 0 0 0 0 0
"Viruses;Uroviricota;Caudoviricetes;Caudoviricetes__na;Caudoviricetes__na;Skunavirus;Lactococcus_phage_A
V09" 0 0 0 0 0 0 0 0 0 0 0 0 0
0 0 0 0 0 0 0 0 0 0 0 0 0
0 0 0 0 0 0 0 0 0 0 0 0 0
0 0 0 0 0 0 0 0 0 0 0 0 0
0 0 0 0 0 0 0 0 2 0 0 1 0
0 0 0 0 0 0 0
"Viruses;Uroviricota;Caudoviricetes;Caudoviricetes__na;Caudoviricetes__na;Skunavirus;Lactococcus_phage_FB
14" 0 0 0 0 7 1 0 0 0 0 0 0 0
0 0 0 0 0 0 0 0 0 0 0 0 0
0 0 0 0 0 0 0 0 0 0 0 0 0
0 0 0 0 0 0 0 0 0 0 0 0 0
0 0 0 0 0 0 0 0 0 0 0 0 0
0 0 0 0 0 0 0
"Viruses;Uroviricota;Caudoviricetes;Caudoviricetes__na;Caudoviricetes__na;Skunavirus;Lactococcus_phage_G
L7" 0 0 0 0 0 0 0 0 0 0 0 0 0
0 0 0 0 0 0 0 0 0 0 0 0 0
0 0 0 0 1 0 0 0 8 0 0 0 0
0 0 0 0 0 0 0 0 0 0 0 0 0
0 0 0 0 0 0 0 0 6 0 0 1 0
0 0 0 0 0 0 0
"Viruses;Uroviricota;Caudoviricetes;Caudoviricetes__na;Caudoviricetes__na;Skunavirus;Lactococcus_phage_G
P13" 0 0 0 0 0 0 0 0 0 0 0 0 0
0 0 0 0 6 0 0 0 0 0 0 0 0
0 0 0 0 0 0 0 0 0 0 0 0 0
0 0 0 0 0 0 0 0 0 0 0 0 0
0 0 0 0 0 0 0 0 3 0 0 0 0
0 0 0 0 0 0 0
"Viruses;Uroviricota;Caudoviricetes;Caudoviricetes__na;Caudoviricetes__na;Skunavirus;Lactococcus_phage_LP
0004c" 0 0 0 0 0 0 0 0 0 0 0 0 0
0 0 0 0 0 0 0 0 0 0 0 0 0
0 0 0 0 0 0 1 0 0 0 0 0 0
0 0 0 0 0 0 0 0 0 0 0 0 0
0 0 0 0 0 0 0 0 0 0 0 0 0
0 0 0 0 0 0 0
"Viruses;Uroviricota;Caudoviricetes;Caudoviricetes__na;Caudoviricetes__na;Skunavirus;Lactococcus_phage_Q3
7" 0 0 0 0 0 0 0 0 0 0 0 0 0
0 0 0 0 0 0 0 0 0 0 0 0 0
0 0 0 0 1 0 0 0 0 0 0 0 0
0 0 0 0 0 0 0 0 0 0 0 0 0

0 0 0 0 0 0 0 0 0 0 0 0 0
0 0 0 0 0 0 0
"Viruses;Uroviricota;Caudoviricetes;Caudoviricetes__na;Caudoviricetes__na;Skunavirus;Lactococcus_phage_R
H10" 0 0 0 0 0 0 0 0 0 0 0 0 0
0 0 0 0 0 0 0 0 0 0 0 0 0
0 0 0 0 0 0 0 0 0 0 0 0 0
0 0 0 0 0 0 0 0 0 0 0 0 0
0 0 0 0 0 0 0 0 1 0 0 0 0
0 0 0 0 0 0 0
"Viruses;Uroviricota;Caudoviricetes;Caudoviricetes__na;Caudoviricetes__na;Skunavirus;Skunavirus__uc"
0 0 0 0 0 0 0 0 0 0 0 0 0
1 1 1 1 14 0 0 0 0 0 0 0 0
0 0 0 0 9 10 4 0 623 0 0 0 0
1 0 6 0 0 0 0 10 0 0 0 0 0
3 0 9 20 0 0 0 0 204 45 13 40 2
0 0 0 0 0 0 0
"Viruses;Uroviricota;Caudoviricetes;Caudoviricetes__na;Caudoviricetes__na;Skunavirus;Skunavirus_A16"
0 0 0 0 0 0 0 0 0 0 0 0 0
0 0 0 0 0 0 0 0 0 0 0 0 0
0 0 0 0 0 0 0 0 0 0 0 0 0
0 0 0 0 0 0 0 0 0 0 0 0 0
0 0 0 0 0 0 0 0 3 0 1 1 0
0 0 0 0 0 0 0
"Viruses;Uroviricota;Caudoviricetes;Caudoviricetes__na;Caudoviricetes__na;Skunavirus;Skunavirus_ASCC191"
0 0 0 0 0 0 0 0 0 0 0 0 0
0 0 0 0 0 0 0 0 0 0 0 0 0
0 0 0 0 0 0 0 0 0 0 0 0 0
0 0 0 0 0 0 0 0 0 0 0 0 0
0 0 0 0 0 0 0 0 3 1 0 0 0
0 0 0 0 0 0 0
"Viruses;Uroviricota;Caudoviricetes;Caudoviricetes__na;Caudoviricetes__na;Skunavirus;Skunavirus_ASCC273"
0 0 0 0 0 0 0 0 0 0 0 0 0
0 0 0 0 0 0 0 0 0 0 0 0 0
0 0 0 0 0 0 0 0 1 0 0 0 0
0 0 0 0 0 0 0 0 0 0 0 0 0
0 0 0 0 0 0 0 0 0 0 0 0 0
0 0 0 0 0 0 0
"Viruses;Uroviricota;Caudoviricetes;Caudoviricetes__na;Caudoviricetes__na;Skunavirus;Skunavirus_ASCC532"
0 0 0 0 0 0 0 0 0 0 0 0 0
0 0 0 0 0 0 0 0 0 0 0 0 0
0 0 0 0 0 0 0 0 0 0 0 0 0
0 0 0 0 0 0 0 0 0 0 0 0 0
0 0 0 0 0 0 0 0 1 0 0 0 0
0 0 0 0 0 0 0
"Viruses;Uroviricota;Caudoviricetes;Caudoviricetes__na;Caudoviricetes__na;Skunavirus;Skunavirus_B1127"
0 0 0 0 0 0 0 0 0 0 0 0 0
0 0 0 0 1 0 0 0 0 0 0 0 0
0 0 0 0 0 0 0 0 3 0 0 0 0
0 0 0 0 0 0 0 0 0 0 0 0 0

0 0 1 0 0 0 0 0 0 0 0 1 0
0 0 0 0 0 0 0
"Viruses;Uroviricota;Caudoviricetes;Caudoviricetes__na;Caudoviricetes__na;Skunavirus;Skunavirus_bibb29"
0 0 0 0 0 0 0 0 0 0 0 0 0
0 0 0 0 0 0 0 0 0 0 0 0 0
0 0 0 0 0 0 0 0 0 0 0 0 0
0 0 0 0 0 0 0 0 0 0 0 0 0
0 0 1 0 0 0 0 0 0 0 0 0 0
0 0 0 0 0 0 0
"Viruses;Uroviricota;Caudoviricetes;Caudoviricetes__na;Caudoviricetes__na;Skunavirus;Skunavirus_caseusJM1
" 0 0 0 0 0 0 0 0 0 0 0 0 0
0 0 0 0 0 0 0 0 0 0 0 0 0
0 0 0 0 0 0 0 0 0 0 0 0 0
0 0 0 0 0 0 0 0 0 0 0 0 0
0 0 0 1 0 0 0 0 1 0 0 0 0
0 0 0 0 0 0 0
"Viruses;Uroviricota;Caudoviricetes;Caudoviricetes__na;Caudoviricetes__na;Skunavirus;Skunavirus_CB13"
0 0 0 0 0 0 0 0 0 0 0 0 0
0 0 0 0 0 0 0 0 0 0 0 0 0
0 0 0 0 1 0 0 0 0 0 0 0 0
0 0 0 0 0 0 0 0 0 0 0 0 0
0 0 0 3 0 0 0 0 4 0 0 0 0
0 0 0 0 0 0 0
"Viruses;Uroviricota;Caudoviricetes;Caudoviricetes__na;Caudoviricetes__na;Skunavirus;Skunavirus_CB19"
0 0 0 0 0 0 0 0 0 0 0 0 0
0 0 0 0 0 0 0 0 0 0 0 0 0
0 0 0 0 0 0 0 0 0 0 0 0 0
0 0 0 0 0 0 0 0 0 0 0 0 0
0 0 0 0 0 0 0 0 0 1 0 0 0
0 0 0 0 0 0 0
"Viruses;Uroviricota;Caudoviricetes;Caudoviricetes__na;Caudoviricetes__na;Skunavirus;Skunavirus_CHPC129"
0 0 0 0 0 0 0 0 0 0 0 0 0
0 0 0 0 0 0 0 0 0 0 0 0 0
0 0 0 0 0 1 0 0 1 0 0 0 0
0 0 0 0 0 0 0 0 0 0 0 0 0
0 0 0 0 0 0 0 0 0 0 0 0 0
0 0 0 0 0 0 0
"Viruses;Uroviricota;Caudoviricetes;Caudoviricetes__na;Caudoviricetes__na;Skunavirus;Skunavirus_CHPC362"
0 0 0 0 0 0 0 0 0 0 0 0 0
0 0 0 0 4 0 0 0 0 0 0 0 0
0 0 0 0 0 0 0 0 0 0 0 0 0
0 0 0 0 0 0 0 0 0 0 0 0 0
0 0 0 1 0 0 0 0 0 0 0 0 0
0 0 0 0 0 0 0
"Viruses;Uroviricota;Caudoviricetes;Caudoviricetes__na;Caudoviricetes__na;Skunavirus;Skunavirus_CHPC52"
0 0 0 0 0 0 0 0 0 0 0 0 0
0 0 0 0 0 0 0 0 0 0 0 0 0
0 0 0 0 0 0 0 0 8 0 0 0 0
0 0 0 0 0 0 0 0 0 0 0 0 0

0 0 0 3 0 0 0 0 0 0 0 0 0
0 0 0 0 0 0 0
"Viruses;Uroviricota;Caudoviricetes;Caudoviricetes__na;Caudoviricetes__na;Skunavirus;Skunavirus_CHPC781"
0 0 0 0 0 0 0 0 0 0 0 0 0
0 0 0 0 0 0 0 0 0 0 0 0 0
0 0 0 0 0 0 0 0 0 0 0 0 0
0 0 0 0 0 0 0 0 0 0 0 0 0
0 0 0 0 0 0 0 0 3 0 0 0 0
0 0 0 0 0 0 0
"Viruses;Uroviricota;Caudoviricetes;Caudoviricetes__na;Caudoviricetes__na;Skunavirus;Skunavirus_CHPC958"
0 0 0 0 0 0 0 0 0 0 0 0 0
0 0 0 0 0 0 0 0 0 0 0 0 0
0 0 0 0 0 0 0 0 0 0 0 0 0
0 0 0 0 0 0 0 0 0 0 0 0 0
0 0 0 1 0 0 0 0 1 0 0 0 0
0 0 0 0 0 0 0
"Viruses;Uroviricota;Caudoviricetes;Caudoviricetes__na;Caudoviricetes__na;Skunavirus;Skunavirus_CHPC959"
0 0 0 0 0 0 0 0 0 0 0 0 0
0 0 0 0 0 0 0 0 0 0 0 0 0
0 0 0 0 0 0 0 0 2 0 0 0 0
0 0 0 0 0 0 0 0 0 0 0 0 0
0 0 0 0 0 0 0 0 3 0 1 0 0
0 0 0 0 0 0 0
"Viruses;Uroviricota;Caudoviricetes;Caudoviricetes__na;Caudoviricetes__na;Skunavirus;Skunavirus_CHPC964"
0 0 0 0 0 0 0 0 0 0 0 0 0
0 0 0 0 0 0 0 0 0 0 0 0 0
0 0 0 0 0 0 0 0 0 0 0 0 0
0 0 0 0 0 0 0 0 0 0 0 0 0
0 0 0 0 0 0 0 0 1 0 0 0 0
0 0 0 0 0 0 0
"Viruses;Uroviricota;Caudoviricetes;Caudoviricetes__na;Caudoviricetes__na;Skunavirus;Skunavirus_CHPC965"
0 0 0 0 0 0 0 0 0 0 0 0 0
0 0 0 0 0 0 0 0 0 0 0 0 0
0 0 0 0 0 0 0 0 0 0 0 0 0
0 0 0 0 0 0 0 0 0 0 0 0 0
0 0 0 0 0 0 0 0 1 0 0 1 0
0 0 0 0 0 0 0
"Viruses;Uroviricota;Caudoviricetes;Caudoviricetes__na;Caudoviricetes__na;Skunavirus;Skunavirus_E1127"
0 0 0 0 0 0 0 0 0 0 0 0 0
0 0 0 0 0 0 0 0 0 0 0 0 0
0 0 0 0 0 0 0 0 0 0 0 0 0
0 0 0 0 0 0 0 0 0 0 0 0 0
0 0 0 0 0 0 0 0 1 0 0 0 0
0 0 0 0 0 0 0
"Viruses;Uroviricota;Caudoviricetes;Caudoviricetes__na;Caudoviricetes__na;Skunavirus;Skunavirus_F0139"
0 0 0 0 0 0 0 0 0 0 0 0 0
0 0 0 0 0 0 0 0 0 0 0 0 0
0 0 0 0 0 0 0 0 2 0 0 0 0
0 0 0 0 0 0 0 0 0 0 0 0 0

0 0 0 1 0 0 0 0 5 0 0 1 0
0 0 0 0 0 0 0
"Viruses;Uroviricota;Caudoviricetes;Caudoviricetes__na;Caudoviricetes__na;Skunavirus;Skunavirus_fd13"
0 0 0 0 0 0 0 0 0 0 0 0 0
0 0 0 1 5 0 0 0 0 0 0 0 0
0 0 0 0 0 0 0 0 0 0 0 0 0
0 0 0 0 0 0 0 0 0 0 0 0 0
0 0 0 0 0 0 0 0 2 1 0 2 0
0 0 0 0 0 0 0
"Viruses;Uroviricota;Caudoviricetes;Caudoviricetes__na;Caudoviricetes__na;Skunavirus;Skunavirus_i0139"
0 0 0 0 0 0 0 0 0 0 0 0 0
0 0 0 0 0 0 2 0 0 0 0 0 0
0 0 0 0 0 0 0 0 4 0 0 0 0
0 0 0 0 0 0 0 0 0 0 0 0 0
0 0 0 0 0 0 0 0 6 0 0 0 0
0 0 0 0 0 0 0
"Viruses;Uroviricota;Caudoviricetes;Caudoviricetes__na;Caudoviricetes__na;Skunavirus;Skunavirus_JF1"0
0 0 0 0 0 0 0 0 0 0 0 0 0
0 0 0 0 0 0 0 0 0 0 0 0 0
0 0 0 0 0 0 0 0 0 0 0 0 0
0 0 0 0 0 0 0 0 0 0 0 0 0
0 0 0 0 0 0 0 3 0 2 0 0 0
0 0 0 0 0 0
"Viruses;Uroviricota;Caudoviricetes;Caudoviricetes__na;Caudoviricetes__na;Skunavirus;Skunavirus_jm2"
0 0 0 0 0 0 0 0 0 0 0 0 0
0 0 0 0 0 0 0 0 0 0 0 0 0
0 0 0 0 0 0 0 0 0 0 0 0 0
0 0 0 0 0 0 0 0 0 0 0 0 0
0 0 0 0 0 0 0 0 2 0 0 0 0
0 0 0 0 0 0 0
"Viruses;Uroviricota;Caudoviricetes;Caudoviricetes__na;Caudoviricetes__na;Skunavirus;Skunavirus_jm3"
0 0 0 0 0 0 0 0 0 0 0 0 0
0 0 0 0 0 0 0 0 0 0 0 0 0
0 0 0 0 0 2 0 0 0 0 0 0 0
0 0 0 0 0 0 0 0 0 0 0 0 0
0 0 0 0 0 0 0 0 1 0 0 0 0
0 0 0 0 0 0 0
"Viruses;Uroviricota;Caudoviricetes;Caudoviricetes__na;Caudoviricetes__na;Skunavirus;Skunavirus_L18"
0 0 0 0 0 0 0 0 0 0 0 0 0
1 0 0 0 0 0 0 0 0 0 0 0 0
0 0 0 0 0 0 0 0 0 0 0 0 0
0 0 0 0 0 0 0 0 0 0 0 0 0
0 0 0 0 0 0 0 0 0 0 0 0 0
0 0 0 0 0 0 0
"Viruses;Uroviricota;Caudoviricetes;Caudoviricetes__na;Caudoviricetes__na;Skunavirus;Skunavirus_Lj" 0
0 0 0 0 0 0 0 0 0 0 0 0 0
0 0 0 0 0 0 0 0 0 0 0 0 0
0 0 0 0 0 0 0 0 0 0 0 0 0
0 0 0 0 0 0 0 0 0 0 0 0 0

0 1 0 0 0 0 0 1 0 0 0 0 0
0 0 0 0 0 0
"Viruses;Uroviricota;Caudoviricetes;Caudoviricetes__na;Caudoviricetes__na;Skunavirus;Skunavirus_LP0903"
0 0 0 0 0 0 0 0 0 0 0 0 0
0 0 0 0 0 0 0 0 0 0 0 0 0
0 0 0 0 0 0 0 0 1 0 0 0 0
0 0 0 0 0 0 0 0 0 0 0 0 0
0 0 0 0 0 0 0 0 0 0 0 0 0
0 0 0 0 0 0 0
"Viruses;Uroviricota;Caudoviricetes;Caudoviricetes__na;Caudoviricetes__na;Skunavirus;Skunavirus_LP1502a"
0 0 0 0 0 0 0 0 0 0 0 0 0
0 0 0 0 0 0 0 0 0 0 0 0 0
0 0 0 0 0 0 1 0 1 0 0 0 0
0 0 0 0 0 0 0 0 0 0 0 0 0
0 0 0 0 0 0 0 0 1 1 0 1 0
0 0 0 0 0 0 0
"Viruses;Uroviricota;Caudoviricetes;Caudoviricetes__na;Caudoviricetes__na;Skunavirus;Skunavirus_LP8511"
0 0 0 0 0 0 0 0 0 0 0 0 0
0 0 0 0 0 0 0 0 0 0 0 0 0
0 0 0 0 0 0 0 0 2 0 0 0 0
0 0 0 0 0 0 0 0 0 0 0 0 0
0 0 0 0 0 0 0 0 0 0 0 0 0
0 0 0 0 0 0 0
"Viruses;Uroviricota;Caudoviricetes;Caudoviricetes__na;Caudoviricetes__na;Skunavirus;Skunavirus_MV10L"
0 0 0 0 0 0 0 0 0 0 0 0 0
0 0 0 0 1 0 0 0 0 0 0 0 0
0 0 0 0 1 0 0 0 0 0 0 0 0
0 0 0 0 0 0 0 0 0 0 0 0 0
0 0 0 0 0 0 0 0 0 1 0 1 0
0 0 0 0 0 0 0
"Viruses;Uroviricota;Caudoviricetes;Caudoviricetes__na;Caudoviricetes__na;Skunavirus;Skunavirus_P008"
0 0 0 0 0 0 0 0 0 0 0 0 0
0 0 0 0 0 0 0 0 0 0 0 0 0
0 0 0 0 0 0 0 0 0 0 0 0 0
0 0 0 0 0 0 0 0 0 0 0 0 0
0 0 0 1 0 0 0 0 0 1 0 0 0
0 0 0 0 0 0 0
"Viruses;Uroviricota;Caudoviricetes;Caudoviricetes__na;Caudoviricetes__na;Skunavirus;Skunavirus_P1532"
0 0 0 0 0 0 0 0 0 0 0 0 0
0 0 0 0 0 0 0 0 0 0 0 0 0
0 0 0 0 0 0 0 0 3 0 0 0 0
0 0 0 0 0 0 0 0 0 0 0 0 0
0 0 0 0 0 0 0 0 1 0 1 0 0
0 0 0 0 0 0 0
"Viruses;Uroviricota;Caudoviricetes;Caudoviricetes__na;Caudoviricetes__na;Skunavirus;Skunavirus_P475"
0 0 0 0 0 0 0 0 0 0 0 0 0
0 0 0 0 0 0 0 0 0 0 0 0 0
0 0 0 0 0 0 0 0 1 0 0 0 0
0 0 0 0 0 0 0 0 0 0 0 0 0

0 0 0 0 0 0 0 0 0 0 0 0 0
0 0 0 0 0 0 0
"Viruses;Uroviricota;Caudoviricetes;Caudoviricetes__na;Caudoviricetes__na;Skunavirus;Skunavirus_P656"
0 0 0 0 0 0 0 0 0 0 0 0 0
0 0 0 0 0 0 0 0 0 0 0 0 0
0 0 0 0 0 0 0 0 0 0 0 0 0
0 0 0 0 0 0 0 0 0 0 0 0 0
0 0 0 0 0 0 0 0 2 0 0 0 0
0 0 0 0 0 0 0
"Viruses;Uroviricota;Caudoviricetes;Caudoviricetes__na;Caudoviricetes__na;Skunavirus;Skunavirus_R31"
0 0 0 0 0 0 0 0 0 0 0 0 0
0 0 0 0 0 0 0 0 0 0 0 0 0
0 0 0 0 0 0 0 0 0 0 0 0 0
0 0 0 0 0 0 0 0 0 0 0 0 0
0 0 0 0 0 0 0 0 3 0 0 0 0
0 0 0 0 0 0 0
"Viruses;Uroviricota;Caudoviricetes;Caudoviricetes__na;Caudoviricetes__na;Skunavirus;Skunavirus_S0139"
0 0 0 0 0 0 0 0 0 0 0 0 1
0 0 0 0 0 0 0 0 0 0 0 0 0
0 0 0 0 0 0 0 0 0 0 0 0 0
0 0 0 0 0 0 0 0 0 0 0 0 0
0 0 0 0 0 0 0 0 12 0 1 1 0
0 0 0 0 0 0 0
"Viruses;Uroviricota;Caudoviricetes;Caudoviricetes__na;Caudoviricetes__na;Skunavirus;Skunavirus_sk1"
0 0 0 0 0 0 0 0 0 0 0 0 0
0 0 0 0 0 0 0 0 0 0 0 0 0
0 0 0 0 0 0 0 0 1 0 0 0 0
0 0 0 0 0 0 0 0 0 0 0 0 0
0 0 0 0 0 0 0 0 5 0 0 1 0
0 0 0 0 0 0 0
"Viruses;Uroviricota;Caudoviricetes;Caudoviricetes__na;Caudoviricetes__na;Skunavirus;Skunavirus_Sl4"0
0 0 0 0 0 0 0 0 0 0 0 0 0
0 0 0 0 0 0 0 0 0 0 0 0 0
0 0 0 0 0 0 0 0 0 0 0 0 0
0 0 0 0 0 0 0 0 0 0 0 0 0
0 0 0 0 0 0 0 4 1 0 0 0 0
0 0 0 0 0 0
"Viruses;Uroviricota;Caudoviricetes;Caudoviricetes__na;Caudoviricetes__na;Skunavirus;Skunavirus_sv05601"
0 0 0 0 0 0 0 0 0 0 0 0 0
0 0 0 0 0 0 0 0 0 0 0 0 0
0 0 0 0 0 0 0 0 2 0 0 0 0
0 0 0 0 0 0 0 0 0 0 0 0 0
0 0 0 0 0 0 0 0 0 0 0 0 0
0 0 0 0 0 0 0
"Viruses;Uroviricota;Caudoviricetes;Caudoviricetes__na;Caudoviricetes__na;Skunavirus;Skunavirus_sv10W18"
0 0 0 0 0 0 0 0 0 0 0 0 0
0 0 0 0 14 0 0 0 0 0 0 0 0
0 0 0 0 0 0 0 0 0 0 0 0 0
0 0 0 0 0 0 0 0 0 0 0 0 0

0 0 0 0 0 0 0 0 0 0 0 0 0
0 0 0 0 0 0 0
"Viruses;Uroviricota;Caudoviricetes;Caudoviricetes__na;Caudoviricetes__na;Skunavirus;Skunavirus_sv13W11L
" 0 0 0 0 0 0 0 0 0 0 0 0 0
0 0 0 0 0 0 0 0 0 0 0 0 0
0 0 0 0 0 0 0 0 0 0 0 0 0
0 0 0 0 0 0 0 0 0 0 0 0 0
0 0 0 0 0 0 0 0 1 0 0 0 0
0 0 0 0 0 0 0
"Viruses;Uroviricota;Caudoviricetes;Caudoviricetes__na;Caudoviricetes__na;Skunavirus;Skunavirus_sv155"
0 0 0 0 0 0 0 0 0 0 0 0 0
0 0 0 0 0 0 0 0 0 0 0 0 0
0 0 0 0 0 0 0 0 14 0 0 0 0
0 0 0 0 0 0 0 0 0 0 0 0 0
0 0 1 1 0 0 0 0 0 0 0 0 0
0 0 0 0 0 0 0
"Viruses;Uroviricota;Caudoviricetes;Caudoviricetes__na;Caudoviricetes__na;Skunavirus;Skunavirus_sv16802"
0 0 0 0 0 0 0 0 0 0 0 0 0
0 0 0 0 5 0 0 0 0 0 0 0 0
0 0 0 0 0 0 0 0 0 0 0 0 0
0 0 0 0 0 0 0 0 0 0 0 0 0
0 0 2 0 0 0 0 0 5 0 0 0 0
0 0 0 0 0 0 0
"Viruses;Uroviricota;Caudoviricetes;Caudoviricetes__na;Caudoviricetes__na;Skunavirus;Skunavirus_sv16W23"
0 0 0 0 0 0 0 0 0 0 0 0 0
0 0 0 0 0 0 0 0 0 0 0 0 0
0 0 0 0 0 0 0 0 0 0 0 0 0
0 0 0 0 0 0 0 0 0 0 0 0 0
0 0 0 0 0 0 0 0 1 0 0 0 0
0 0 0 0 0 0 0
"Viruses;Uroviricota;Caudoviricetes;Caudoviricetes__na;Caudoviricetes__na;Skunavirus;Skunavirus_sv30804"
0 0 0 0 0 0 0 0 0 0 0 0 0
0 0 0 0 0 0 0 0 0 0 0 0 0
0 0 0 0 0 0 0 0 0 0 0 0 0
0 0 0 0 0 0 0 0 0 0 0 0 0
0 0 1 1 0 0 0 0 3 0 0 0 0
0 0 0 0 0 0 0
"Viruses;Uroviricota;Caudoviricetes;Caudoviricetes__na;Caudoviricetes__na;Skunavirus;Skunavirus_sv340"
0 0 0 0 0 0 0 0 0 0 0 0 0
0 0 0 0 0 0 0 0 1 0 0 0 0
0 0 0 0 0 0 0 0 0 0 0 0 0
0 0 0 0 0 0 0 0 0 0 0 0 0
0 0 0 0 0 0 0 0 1 0 0 0 0
0 0 0 0 0 0 0
"Viruses;Uroviricota;Caudoviricetes;Caudoviricetes__na;Caudoviricetes__na;Skunavirus;Skunavirus_sv37201"
0 0 0 0 0 0 0 0 0 0 0 0 0
0 0 0 0 0 0 0 0 0 0 0 0 0
0 0 0 0 0 0 0 0 3 0 0 0 0
0 0 0 0 0 0 0 0 0 0 0 0 0

0 0 1 0 0 0 0 0 1 0 0 0 0
0 0 0 0 0 0 0
"Viruses;Uroviricota;Caudoviricetes;Caudoviricetes__na;Caudoviricetes__na;Skunavirus;Skunavirus_sv38507"
0 0 0 0 0 0 0 0 0 0 0 0 0
0 0 0 0 0 0 0 0 0 0 0 0 0
0 0 0 0 1 0 0 0 0 0 0 0 0
0 0 0 0 0 0 0 0 0 0 0 0 0
0 0 0 0 0 0 0 0 1 0 0 0 0
0 0 0 0 0 0 0
"Viruses;Uroviricota;Caudoviricetes;Caudoviricetes__na;Caudoviricetes__na;Skunavirus;Skunavirus_sv3R16S"
0 0 0 0 0 0 0 0 0 0 0 0 0
0 0 0 0 0 0 0 0 0 0 0 0 0
0 0 0 0 0 0 0 0 0 0 0 0 0
0 0 0 0 0 0 0 0 0 0 0 0 0
0 0 0 1 0 0 0 0 1 0 0 0 0
0 0 0 0 0 0 0
"Viruses;Uroviricota;Caudoviricetes;Caudoviricetes__na;Caudoviricetes__na;Skunavirus;Skunavirus_sv42"
0 0 0 0 0 0 0 0 0 0 0 0 0
0 0 0 0 0 0 0 0 0 0 0 0 0
0 0 0 0 1 0 0 0 0 0 0 0 0
0 0 0 0 0 0 0 0 0 0 0 0 0
0 0 0 0 0 0 0 0 0 0 0 0 0
0 0 0 0 0 0 0
"Viruses;Uroviricota;Caudoviricetes;Caudoviricetes__na;Caudoviricetes__na;Skunavirus;Skunavirus_sv51701"
0 0 0 0 0 0 0 0 0 0 0 0 0
0 0 0 0 0 0 0 0 0 0 0 0 0
0 0 0 0 0 0 0 0 0 0 0 0 0
0 0 0 0 0 0 0 0 0 0 0 0 0
0 0 0 0 0 0 0 0 1 1 0 1 0
0 0 0 0 0 0 0
"Viruses;Uroviricota;Caudoviricetes;Caudoviricetes__na;Caudoviricetes__na;Skunavirus;Skunavirus_sv56301"
0 0 0 0 0 0 0 0 0 0 0 0 0
0 0 0 0 0 0 0 0 0 0 0 0 0
0 0 0 0 0 0 0 0 1 0 0 0 0
0 0 0 0 0 0 0 0 0 0 0 0 0
0 0 0 0 0 0 0 0 0 0 0 0 0
0 0 0 0 0 0 0
"Viruses;Uroviricota;Caudoviricetes;Caudoviricetes__na;Caudoviricetes__na;Skunavirus;Skunavirus_sv57001"
0 0 0 0 0 0 0 0 0 0 0 0 0
0 0 0 0 0 0 0 0 0 0 0 0 0
0 0 0 0 0 0 0 0 6 0 0 0 0
0 0 0 0 0 0 0 0 0 0 0 0 0
0 0 0 0 0 0 0 0 4 1 0 0 0
0 0 0 0 0 0 0
"Viruses;Uroviricota;Caudoviricetes;Caudoviricetes__na;Caudoviricetes__na;Skunavirus;Skunavirus_sv62605"
0 0 0 0 0 0 0 0 0 0 0 0 0
0 0 0 0 0 0 0 0 0 0 0 0 0
0 0 0 0 0 1 0 0 1 0 0 0 0
0 0 0 0 0 0 0 0 0 0 0 0 0

0 0 0 0 0 0 0 0 0 0 0 0 0
0 0 0 0 0 0 0
"Viruses;Uroviricota;Caudoviricetes;Caudoviricetes__na;Caudoviricetes__na;Skunavirus;Skunavirus_sv63302"
0 0 0 0 0 0 0 0 0 0 0 0 0
0 0 0 0 0 0 0 0 0 0 0 0 0
0 0 0 0 0 0 0 0 6 0 1 0 0
0 0 0 0 0 0 0 0 0 0 0 0 0
0 0 0 0 0 0 0 0 1 0 0 0 0
0 0 0 0 0 0 0
"Viruses;Uroviricota;Caudoviricetes;Caudoviricetes__na;Caudoviricetes__na;Skunavirus;Skunavirus_sv66901"
0 0 0 0 0 0 0 0 0 0 0 0 0
0 0 0 0 0 0 0 0 0 0 0 0 0
0 0 0 0 0 0 0 0 1 0 0 0 0
0 0 0 0 0 0 0 0 0 0 0 0 0
0 0 0 0 0 0 0 0 5 0 0 1 0
0 0 0 0 0 0 0
"Viruses;Uroviricota;Caudoviricetes;Caudoviricetes__na;Caudoviricetes__na;Skunavirus;Skunavirus_sv7"
0 0 0 0 0 0 0 0 0 0 0 0 0
0 0 0 0 0 0 0 0 0 0 0 0 0
0 0 0 0 2 0 1 0 64 0 0 0 0
0 0 0 0 0 0 0 0 0 0 0 0 0
0 0 0 0 0 0 0 0 6 0 0 3 0
0 0 0 0 0 0 0
"Viruses;Uroviricota;Caudoviricetes;Caudoviricetes__na;Caudoviricetes__na;Skunavirus;Skunavirus_sv712"
0 0 0 0 0 0 0 0 0 0 0 0 0
0 0 0 0 0 0 0 0 0 0 0 0 0
0 0 0 0 0 0 0 1 1 0 0 0 0
0 0 0 0 0 0 0 0 0 0 0 0 0
0 0 0 0 0 0 0 0 0 0 0 0 0
0 0 0 0 0 0 0
"Viruses;Uroviricota;Caudoviricetes;Caudoviricetes__na;Caudoviricetes__na;Skunavirus;Skunavirus_sv79201"
0 0 0 0 0 0 0 0 0 0 0 0 0
0 0 0 0 0 0 0 1 0 0 0 0 0
0 0 0 0 0 0 0 0 0 0 0 0 0
0 0 0 0 0 0 0 0 0 0 0 0 0
0 0 0 0 0 0 0 0 1 2 1 0 0
0 0 0 0 0 0 0
"Viruses;Uroviricota;Caudoviricetes;Caudoviricetes__na;Caudoviricetes__na;Skunavirus;Skunavirus_sv88605"
0 0 0 0 0 0 0 0 0 0 0 0 0
0 0 0 0 0 0 0 0 0 0 0 0 0
0 0 0 0 0 2 0 0 0 0 0 0 0
0 0 0 0 0 0 0 0 0 0 0 0 0
0 0 0 1 0 0 0 0 0 0 0 0 0
0 0 0 0 0 0 0
"Viruses;Uroviricota;Caudoviricetes;Caudoviricetes__na;Caudoviricetes__na;Skunavirus;Skunavirus_sv91127"
0 0 0 0 0 0 0 0 0 0 0 0 0
0 0 0 0 0 0 0 0 0 0 0 0 0
0 0 0 0 0 0 0 0 0 0 0 0 0
0 0 0 0 0 0 0 0 0 0 0 0 0

0 0 0 0 0 0 0 0 0 0 0 1 0
0 0 0 0 0 0 0
"Viruses;Uroviricota;Caudoviricetes;Caudoviricetes__na;Caudoviricetes__na;Skunavirus;Skunavirus_sv96401"
0 0 0 0 0 0 0 0 0 0 0 0 0
0 0 0 0 0 0 0 0 0 0 0 0 0
0 0 0 0 0 0 0 0 0 0 0 0 0
0 0 0 0 0 0 0 22 0 0 0 0 0
0 0 0 0 0 0 0 0 1 0 0 1 0
0 0 0 0 0 0 0
"Viruses;Uroviricota;Caudoviricetes;Caudoviricetes__na;Caudoviricetes__na;Skunavirus;Skunavirus_sv96403"
0 0 0 0 0 0 0 0 0 0 0 0 0
0 0 0 0 1 0 0 0 0 0 0 0 0
0 0 0 0 0 0 0 0 1 0 0 0 0
0 0 0 0 0 0 0 0 0 0 0 0 0
0 0 0 0 0 0 0 0 2 0 0 0 0
0 0 0 0 0 0 0
"Viruses;Uroviricota;Caudoviricetes;Caudoviricetes__na;Caudoviricetes__na;Sourvirus;Sourvirus__uc" 0
0 0 0 0 0 0 0 0 0 0 0 0 0
0 0 0 0 0 0 1 0 0 0 0 0 0
0 0 0 0 0 0 0 0 0 0 0 0 0
0 0 0 0 0 0 0 0 0 0 0 0 0
0 0 0 0 0 0 0 0 0 0 0 0 0
0 0 0 0 0 0
"Viruses;Uroviricota;Caudoviricetes;Caudoviricetes__na;Caudoviricetes__na;Sourvirus;Sourvirus_sour" 0
0 0 1 0 0 0 0 0 0 0 0 0 0
0 0 0 0 0 0 0 0 0 0 0 0 0
0 0 0 0 0 0 0 0 0 0 0 0 0
0 0 0 0 0 0 0 0 0 0 0 0 0
0 0 0 0 0 0 0 0 0 0 0 0 0
0 0 0 0 0 0
"Viruses;Uroviricota;Caudoviricetes;Caudoviricetes__na;Caudoviricetes__na;Spbetavirus;Bacillus_phage_BUCT
083" 0 1 0 0 0 0 0 0 0 0 0 0 0
0 0 0 0 0 0 0 0 0 0 0 0 0
0 0 0 0 0 0 0 0 0 0 0 0 0
0 0 0 0 0 0 1 0 0 0 0 0 0
0 0 0 0 0 0 0 0 0 0 0 0 0
0 0 0 0 0 0 0
"Viruses;Uroviricota;Caudoviricetes;Caudoviricetes__na;Caudoviricetes__na;Spbetavirus;Bacillus_phage_phi3T"
0 3 0 0 0 0 0 0 0 0 0 0 0
0 0 0 0 0 0 0 0 0 0 0 0 0
0 0 0 0 0 0 0 0 0 0 0 0 0
0 0 0 0 0 0 0 0 0 0 0 0 0
0 0 0 0 0 0 0 0 0 0 0 0 0
0 0 0 0 0 0 0
"Viruses;Uroviricota;Caudoviricetes;Caudoviricetes__na;Caudoviricetes__na;Squashvirus;Squashvirus__uc"
0 0 0 0 0 0 0 0 1 0 0 0 0
0 0 0 0 0 0 0 0 0 0 0 0 0
0 0 0 0 0 0 0 0 0 0 0 0 0
0 0 0 0 0 0 0 0 0 0 0 0 0

0 0 0 0 0 0 0 0 0 0 0 0 0
0 0 0 0 0 0 0
"Viruses;Uroviricota;Caudoviricetes;Caudoviricetes__na;Caudoviricetes__na;Stormageddonvirus;Stormageddon
virus__uc" 0 0 0 0 0 0 0 0 0 3 0 0
0 0 0 0 0 0 0 0 0 0 0 0 0
0 0 0 0 0 0 0 0 0 0 0 0 0
0 0 0 0 0 0 0 0 0 0 0 0 0
0 0 0 0 0 0 0 0 0 0 0 0 0
0 0 0 0 0 0 0 0
"Viruses;Uroviricota;Caudoviricetes;Caudoviricetes__na;Caudoviricetes__na;Takahashivirus;Takahashivirus_PB
S1" 0 0 0 0 5 1 0 0 0 0 0 0 0
0 0 0 0 0 0 0 0 0 0 0 0 0
0 0 0 0 0 0 0 0 0 0 0 0 0
0 0 0 0 0 0 0 0 0 0 0 0 0
0 0 0 0 0 0 0 0 0 0 0 0 0
0 0 0 0 0 0 0
"Viruses;Uroviricota;Caudoviricetes;Caudoviricetes__na;Caudoviricetes__na;Tamkungvirus;Tamkungvirus_ST4
" 0 0 0 0 4 0 0 0 0 0 0 0 0
0 0 0 0 0 0 0 0 0 0 0 0 0
0 0 0 0 0 0 0 0 0 0 0 0 0
0 0 0 0 0 0 0 0 0 0 0 0 0
0 0 0 0 0 0 0 0 0 0 0 0 0
0 0 0 0 0 0 0
"Viruses;Uroviricota;Caudoviricetes;Caudoviricetes__na;Caudoviricetes__na;Tanisvirus;Tanisvirus__uc" 0
0 0 0 0 0 0 0 0 0 0 0 0 0
0 0 0 0 0 0 0 0 0 0 0 0 0
0 0 0 0 0 0 0 1 0 0 0 0 0
0 0 0 0 0 0 0 0 0 0 0 0 0
0 0 0 0 0 0 0 0 0 0 0 0 0
0 0 0 0 0 0
"Viruses;Uroviricota;Caudoviricetes;Caudoviricetes__na;Caudoviricetes__na;Tanisvirus;Tanisvirus_tanis"
0 0 0 0 1 0 0 0 0 0 0 0 0
0 0 0 0 0 0 0 0 0 0 0 0 0
0 0 0 0 0 0 0 0 0 0 0 0 0
0 0 0 0 0 0 0 0 0 0 0 0 0
0 0 0 0 0 0 0 0 0 0 0 0 0
0 0 0 0 0 0 0
"Viruses;Uroviricota;Caudoviricetes;Caudoviricetes__na;Caudoviricetes__na;Tijeunavirus;Tijeunavirus_TJE1"
0 0 0 2 4 0 0 0 0 0 0 0 0
0 0 0 0 0 0 0 0 0 0 0 0 0
0 0 0 0 0 0 0 0 0 0 0 0 0
0 0 0 0 0 0 0 0 0 0 0 0 0
0 0 0 0 0 0 0 0 0 0 0 0 0
0 0 0 0 0 0 0
"Viruses;Uroviricota;Caudoviricetes;Caudoviricetes__na;Caudoviricetes__na;Titanvirus;Titanvirus__uc" 0
0 0 1 0 0 0 0 0 0 0 0 0 0
0 0 0 0 0 0 0 0 0 0 0 0 0
0 0 0 0 0 0 0 0 0 0 0 0 0
0 0 0 0 0 0 0 0 0 0 0 0 0

0 0 0 0 0 0 0 0 0 0 0 0 0
0 0 0 0 0 0
"Viruses;Uroviricota;Caudoviricetes;Caudoviricetes__na;Caudoviricetes__na;Toutatisvirus;Toutatisvirus_toutati
s" 0 0 0 0 0 0 0 0 0 0 0 0 0
0 0 0 0 0 0 0 0 0 0 0 0 0
0 0 0 0 0 0 0 0 0 0 0 0 0
0 0 0 0 0 0 0 0 0 0 0 0 0
0 0 0 0 0 0 0 0 5 2 0 0 0
0 0 0 0 0 0 0
"Viruses;Uroviricota;Caudoviricetes;Caudoviricetes__na;Caudoviricetes__na;Traversvirus;Traversvirus__uc"
0 0 0 0 0 0 0 0 0 0 0 0 0
0 0 0 0 0 0 0 0 0 0 0 0 0
0 0 0 0 0 1 0 0 0 0 0 0 0
0 0 0 0 0 0 0 0 0 0 0 0 0
0 0 0 0 0 0 0 0 5 2 0 0 0
0 0 0 0 0 0 0
"Viruses;Uroviricota;Caudoviricetes;Caudoviricetes__na;Caudoviricetes__na;Traversvirus;Traversvirus_min27"
0 0 0 0 0 0 0 0 0 0 0 0 0
0 0 0 0 0 0 0 0 0 0 0 0 0
0 0 0 0 0 0 0 0 0 0 0 0 0
0 0 0 0 0 0 0 0 0 0 0 0 0
0 0 0 0 0 0 0 0 1 0 0 0 0
0 0 0 0 0 0 0
"Viruses;Uroviricota;Caudoviricetes;Caudoviricetes__na;Caudoviricetes__na;Traversvirus;Traversvirus_tv933W
" 0 0 0 0 0 0 0 0 0 0 0 0 0
0 0 0 0 0 0 0 0 0 0 0 0 0
0 0 0 0 0 0 0 0 0 0 0 0 0
0 0 0 0 0 0 0 0 0 0 0 0 0
0 0 0 0 0 0 0 0 1 0 0 0 0
0 0 0 0 0 0 0
"Viruses;Uroviricota;Caudoviricetes;Caudoviricetes__na;Caudoviricetes__na;Trinavirus;Rhodococcus_phage_Ni
ceHouse" 0 0 0 1 0 0 0 0 0 0 0 0
0 0 0 0 0 0 0 0 0 0 0 0 0
0 0 0 0 0 0 0 0 0 0 0 0 0
0 0 0 0 0 0 0 0 0 0 0 0 0
0 0 0 0 0 0 0 0 0 0 0 0 0
0 0 0 0 0 0 0 0
"Viruses;Uroviricota;Caudoviricetes;Caudoviricetes__na;Caudoviricetes__na;Uetakevirus;Enterobacter_phage_T
yrion" 0 0 0 0 0 0 0 0 0 0 0 0 0
0 0 0 0 0 0 0 0 0 0 0 0 0
0 0 0 0 0 0 0 0 0 0 0 0 0
0 0 0 0 0 0 0 0 0 0 0 0 0
0 0 0 0 0 0 0 0 2 0 0 0 0
0 0 0 0 0 0 0
"Viruses;Uroviricota;Caudoviricetes;Caudoviricetes__na;Caudoviricetes__na;Uetakevirus;Uetakevirus__uc"
0 0 0 0 0 0 0 0 0 0 0 0 0
0 0 0 0 0 0 0 0 0 0 0 0 0
0 0 0 0 0 0 0 0 0 0 0 0 0
0 0 0 0 0 0 0 0 0 0 0 0 0

0 0 0 0 0 0 0 0 8 0 0 0 0
0 0 0 0 0 0 0
"Viruses;Uroviricota;Caudoviricetes;Caudoviricetes__na;Caudoviricetes__na;Unaquatrovirus;Unaquatrovirus__uc" 0 0 0 0 0 0 0 0 0 0 0 0 0
0 0 1 0 8 0 0 0 0 0 0 0 0
0 0 0 0 0 0 0 0 0 0 0 0 0
0 0 0 0 0 0 0 0 0 0 0 0 0
0 0 0 0 0 0 0 0 0 0 0 0 0
0 0 0 0 0 0 0
"Viruses;Uroviricota;Caudoviricetes;Caudoviricetes__na;Caudoviricetes__na;Unaquatrovirus;Unaquatrovirus_Ln9" 0 0 0 0 0 0 0 0 0 0 0 0 0
0 0 0 0 7 0 0 0 0 0 0 0 0
0 0 0 0 0 0 0 0 0 0 0 0 0
0 0 0 0 0 0 0 0 0 0 0 0 0
0 0 0 0 0 0 0 0 0 0 0 0 0
0 0 0 0 0 0 0
"Viruses;Uroviricota;Caudoviricetes;Caudoviricetes__na;Caudoviricetes__na;Unaquatrovirus;Unaquatrovirus_uv1A4" 0 0 0 0 0 0 0 0 0 0 0 0 0
0 0 0 0 2 0 0 0 0 0 0 0 0
0 0 0 0 0 0 0 0 0 0 0 0 0
0 0 0 0 0 0 0 0 0 0 0 0 0
0 0 0 0 0 0 0 0 0 0 0 0 0
0 0 0 0 0 0 0
"Viruses;Uroviricota;Caudoviricetes;Caudoviricetes__na;Caudoviricetes__na;Vashvirus;Streptomyces_phage_Euratis" 0 0 0 0 3 0 0 0 0 0 0 0 0
0 0 0 0 0 0 0 0 0 0 0 0 0
0 0 0 0 0 0 0 0 0 0 0 0 0
0 0 0 0 0 0 0 0 0 0 0 0 0
0 0 0 0 0 0 0 0 0 0 0 0 0
0 0 0 0 0 0 0
"Viruses;Uroviricota;Caudoviricetes;Caudoviricetes__na;Caudoviricetes__na;Vedamuthuvirus;Vedamuthuvirus__uc" 0 0 0 0 0 0 0 0 0 0 2 0 0
0 0 0 0 0 0 0 0 0 0 0 0 0
0 0 0 0 0 0 0 0 0 0 0 0 0
0 0 1 0 0 0 0 0 0 0 0 0 0
0 0 0 0 0 0 0 0 0 0 0 0 0
0 0 0 0 0 0 0
"Viruses;Uroviricota;Caudoviricetes;Caudoviricetes__na;Caudoviricetes__na;Vhulanivirus;Vhulanivirus_Shpa"
0 0 0 1 0 2 0 0 0 0 0 0 0
0 0 0 0 0 0 0 0 0 0 0 0 0
0 0 0 0 0 1 0 0 0 0 0 0 0
0 0 0 0 0 0 0 0 1 0 0 0 0
0 0 0 1 0 0 0 0 0 0 0 0 0
0 0 0 0 0 0 0
"Viruses;Uroviricota;Caudoviricetes;Caudoviricetes__na;Caudoviricetes__na;Weaselvirus;Weaselvirus_weasel"
0 0 0 0 0 0 0 0 0 0 0 0 0
0 0 0 0 0 0 0 0 0 0 0 0 0
0 0 0 0 0 0 0 0 0 0 0 0 0
0 0 0 0 0 0 0 0 7 0 0 0 0

0 0 0 0 0 0 0 0 0 0 0 0 0
0 0 0 0 0 0 0
"Viruses;Uroviricota;Caudoviricetes;Caudoviricetes__na;Caudoviricetes__na;Xuquatrovirus;Xuquatrovirus__uc"
0 0 0 1 0 0 0 0 0 0 0 0 0
0 0 0 0 0 0 0 0 0 0 0 0 0
0 0 0 0 0 0 0 0 0 0 0 0 0
0 0 0 0 0 0 0 0 6 0 0 0 0
0 0 0 0 0 0 0 0 0 0 0 0 0
0 0 0 0 0 0 0
"Viruses;Uroviricota;Caudoviricetes;Caudoviricetes__na;Chaseviridae;Faunusvirus;Faunusvirus_faunus" 0
0 0 0 0 7 0 0 0 0 0 0 0 0
0 0 0 0 0 0 0 0 0 0 0 0 0
0 0 0 0 0 0 0 0 0 0 0 0 0
0 0 0 0 0 0 0 0 0 0 0 0 0
0 0 0 0 0 0 0 0 0 0 0 0 0
0 0 0 0 0 0
"Viruses;Uroviricota;Caudoviricetes;Caudoviricetes__na;Chaseviridae;Sabourvirus;Sabourvirus_sv4HA13"
0 0 0 0 0 1 0 0 0 0 0 0 0
0 0 0 0 0 0 0 0 0 0 0 0 0
0 0 0 0 0 0 0 0 0 0 0 0 0
0 0 0 0 0 0 0 0 0 0 0 0 0
0 0 0 0 0 0 0 0 0 0 0 0 0
0 0 0 0 0 0 0
"Viruses;Uroviricota;Caudoviricetes;Caudoviricetes__na;Chaseviridae;Suwonvirus;Suwonvirus__uc" 0
0 0 0 0 0 0 0 0 0 0 0 0 0
0 0 0 0 0 0 0 0 3 0 0 0 0
0 0 0 0 0 0 0 0 0 0 0 0 0
0 0 0 0 0 0 0 0 0 0 0 0 0
0 0 0 0 0 0 0 0 0 0 0 0 0
0 0 0 0 0 0
"Viruses;Uroviricota;Caudoviricetes;Caudoviricetes__na;Demerecviridae;Demerecviridae__uc;Demerecviridae_
_uc" 0 4 0 1 33 0 0 0 0 0 0 0 0
0 0 0 0 0 0 0 0 0 0 0 1 0
0 0 0 0 0 0 0 0 0 3 0 0 0
0 0 2 0 0 0 0 0 0 0 0 0 0
0 0 0 0 0 0 0 0 0 0 0 0 1
0 0 0 0 0 1 0
"Viruses;Uroviricota;Caudoviricetes;Caudoviricetes__na;Demerecviridae;Epseptimavirus;Epseptimavirus_Sw2"
0 0 0 0 0 0 0 0 0 0 0 0 0
0 0 0 0 0 0 0 0 0 0 0 0 0
0 0 0 0 0 0 0 0 0 0 0 0 0
0 0 0 0 0 0 1 0 0 0 0 0 0
0 0 0 0 0 0 0 0 0 0 0 0 0
0 0 0 0 0 0 0
"Viruses;Uroviricota;Caudoviricetes;Caudoviricetes__na;Demerecviridae;Jesfedecavirus;Jesfedecavirus__uc"
1 0 0 0 0 0 0 0 0 0 0 0 0
0 0 0 0 0 0 0 0 0 0 0 0 0
0 0 0 0 0 0 0 0 0 0 0 0 0
0 0 0 0 0 0 0 0 0 0 0 0 0

0 0 0 0 0 0 0 0 0 0 0 0 0
0 0 0 0 0 0 0
"Viruses;Uroviricota;Caudoviricetes;Caudoviricetes__na;Demerecviridae;Novosibvirus;Novosibvirus__uc"
0 0 1 0 0 0 0 0 0 0 0 0 0
0 0 0 0 0 0 0 0 0 0 0 0 0
0 0 0 0 0 0 0 0 0 0 0 0 0
0 0 0 0 0 0 0 0 0 0 0 0 0
0 0 0 0 0 0 0 0 0 0 0 0 0
0 0 0 0 0 0 0
"Viruses;Uroviricota;Caudoviricetes;Caudoviricetes__na;Demerecviridae;Pogseptimavirus;Pogseptimavirus__uc
" 0 0 1 0 0 0 0 0 0 0 0 0 0
0 0 0 0 0 0 0 0 0 0 0 0 0
0 0 0 0 0 0 0 0 0 0 0 0 0
0 0 0 0 0 0 0 0 0 0 0 0 0
0 0 0 0 0 0 0 0 0 0 0 0 0
0 0 0 0 0 0 0
"Viruses;Uroviricota;Caudoviricetes;Caudoviricetes__na;Demerecviridae;Pogseptimavirus;Pogseptimavirus_Vs
pSw1" 0 1 0 0 0 0 0 0 0 0 0 0 0
0 0 0 0 0 0 0 0 0 0 0 0 0
0 0 0 0 0 0 0 0 0 0 0 0 0
0 0 0 0 0 0 0 0 0 0 0 0 0
0 0 0 0 0 0 0 0 0 0 0 0 0
0 0 0 0 0 0 0
"Viruses;Uroviricota;Caudoviricetes;Caudoviricetes__na;Demerecviridae;Tequintavirus;Tequintavirus__uc"
0 9 0 0 0 0 0 0 0 0 0 0 0
0 0 0 0 0 0 0 0 0 0 1 1 1
0 0 0 0 0 0 0 0 0 11 0 1 0
0 0 6 0 0 0 0 8 0 0 0 0 0
0 0 0 0 0 0 0 53 0 0 0 0 0
0 0 0 1 3 0 0
"Viruses;Uroviricota;Caudoviricetes;Caudoviricetes__na;Demerecviridae;Tequintavirus;Tequintavirus_SP3"
0 0 0 0 0 0 0 0 0 0 0 0 0
0 0 0 0 0 0 0 0 0 0 0 0 0
0 0 0 0 0 0 0 0 1 0 0 0 0
0 0 0 0 0 0 0 0 0 0 0 0 0
0 0 0 0 0 0 0 0 0 0 0 0 0
0 0 0 0 0 0 0
"Viruses;Uroviricota;Caudoviricetes;Caudoviricetes__na;Drexlerviridae;Eclunavirus;Enterobacter_phage_Phc"
0 0 0 0 0 0 0 0 0 0 0 0 0
0 0 0 0 0 0 0 0 0 0 0 0 0
0 0 0 0 0 0 0 0 0 0 0 0 0
0 0 0 0 0 0 0 0 2 0 0 0 0
0 0 0 0 0 0 0 0 0 0 0 0 0
0 0 0 0 0 0 0
"Viruses;Uroviricota;Caudoviricetes;Caudoviricetes__na;Drexlerviridae;Webervirus;Webervirus__uc" 0
1 0 0 0 0 0 0 0 0 0 0 0 0
0 0 0 0 0 0 0 0 0 0 0 0 0
0 0 0 0 0 0 0 0 0 0 0 0 0
0 0 0 0 0 0 0 0 0 0 0 0 0

0 0 0 0 0 0 0 0 0 0 0 0 0
0 0 0 0 0 0
"Viruses;Uroviricota;Caudoviricetes;Caudoviricetes__na;Forsetiviridae;Freyavirus;Freyavirus__uc" 0
0 0 0 2 0 0 0 0 0 0 0 0 0
0 0 0 0 0 0 0 0 0 0 0 0 0
0 0 0 0 0 0 0 0 0 0 0 0 0
0 0 0 0 0 0 0 0 0 0 0 0 0
0 0 0 0 0 0 0 0 0 0 0 0 0
0 0 0 0 0 0
"Viruses;Uroviricota;Caudoviricetes;Caudoviricetes__na;Helgolandviridae;Leefvirus;Leefvirus_Leef" 0
0 0 0 0 1 0 0 0 0 0 0 0 0
0 0 0 0 0 0 0 0 0 0 0 0 0
0 0 0 0 0 0 0 0 0 0 0 0 0
0 0 0 0 0 0 0 0 0 0 0 0 0
0 0 0 0 0 0 0 0 0 0 0 0 0
0 0 0 0 0 0
"Viruses;Uroviricota;Caudoviricetes;Caudoviricetes__na;Herelleviridae;Baoshanvirus;Baoshanvirus__uc" 0
0 0 0 0 0 0 0 0 0 0 0 0 0
0 0 0 0 0 0 0 0 0 6 0 0 0
0 0 0 0 0 0 0 0 0 0 0 0 0
0 0 0 0 0 0 0 0 0 0 0 0 0
0 0 0 0 0 0 0 0 0 0 0 0 0
0 0 0 0 0 0
"Viruses;Uroviricota;Caudoviricetes;Caudoviricetes__na;Herelleviridae;Baoshanvirus;Staphylococcus_phage_vB
_SauM_JDF86" 0 0 0 0 0 0 0 0 0 0 0 0
0 0 0 0 0 0 0 0 0 0 0 1 0
0 0 0 0 0 0 0 0 0 0 0 0 0
0 0 0 0 0 0 0 0 0 0 0 0 0
0 0 0 0 0 0 0 0 0 0 0 0 0
0 0 0 0 0 0 0 0
"Viruses;Uroviricota;Caudoviricetes;Caudoviricetes__na;Herelleviridae;Baoshanvirus;Staphylococcus_phage_vB
_SsapH-Golestan101-M"0 0 0 0 0 0 0 0 0 0 0
0 0 0 0 0 0 0 0 0 0 0 0 4
0 0 0 0 0 0 0 0 0 0 0 0 0
0 0 0 0 0 0 0 0 0 0 0 0 0
0 0 0 0 0 0 0 0 0 0 0 0 0
0 0 0 0 0 0 0 0 0
"Viruses;Uroviricota;Caudoviricetes;Caudoviricetes__na;Herelleviridae;Bequatrovirus;Bacillus_phage_vB_BcgM
" 0 0 0 0 0 0 0 0 0 0 0 0 0
0 0 0 0 0 0 0 0 0 0 0 0 0
0 0 0 0 0 0 0 0 0 0 0 0 0
0 0 0 0 0 0 0 0 0 0 0 0 0
0 0 1 0 0 0 0 0 0 0 0 0 0
0 0 0 0 0 0 0
"Viruses;Uroviricota;Caudoviricetes;Caudoviricetes__na;Herelleviridae;Caeruleovirus;Caeruleovirus__uc"
0 0 0 0 1 0 0 0 0 0 0 0 0
0 0 0 0 0 0 0 0 0 0 0 0 0
0 0 0 0 0 0 0 0 0 0 0 0 0
0 0 0 0 0 0 0 0 0 0 0 0 0

0 0 0 1 0 0 0 0 0 0 0 0 0
0 0 0 0 0 0 0
"Viruses;Uroviricota;Caudoviricetes;Caudoviricetes__na;Herelleviridae;Caeruleovirus;Caeruleovirus_Bc431"
0 0 0 1 0 2 0 0 0 0 0 0 0
0 0 0 0 0 0 0 0 0 0 0 0 0
0 0 0 0 0 0 0 0 0 0 0 0 0
0 0 0 0 0 0 0 0 0 0 0 0 0
0 0 0 0 0 0 0 0 0 0 0 0 0
0 0 0 0 0 0 0
"Viruses;Uroviricota;Caudoviricetes;Caudoviricetes__na;Herelleviridae;Caeruleovirus;Caeruleovirus_JBP901"
0 0 0 0 0 0 0 0 0 0 0 0 0
0 0 0 0 0 0 0 0 0 0 0 0 0
0 0 0 0 0 0 0 0 0 0 0 0 0
0 0 0 0 0 0 0 0 0 0 0 0 0
0 0 0 1 0 0 0 0 0 0 0 0 0
0 0 0 0 0 0 0
"Viruses;Uroviricota;Caudoviricetes;Caudoviricetes__na;Herelleviridae;Eldridgevirus;Eldridgevirus_eldridge"
0 0 0 0 44 5 0 0 0 0 0 0 0
0 0 0 0 0 0 0 0 0 0 0 0 0
0 0 0 0 0 0 0 0 0 0 0 0 0
0 0 0 0 0 0 0 0 0 0 0 0 0
0 0 0 0 0 0 0 0 0 0 0 0 0
0 0 0 0 0 0 0
"Viruses;Uroviricota;Caudoviricetes;Caudoviricetes__na;Herelleviridae;Herelleviridae__na;Herelleviridae_sp."
1 2 4 2 0 0 0 0 2 0 0 0 93
78 42 44 20 88 100 111 111 110 550 56 6 5
7 0 0 0 32 21 0 0 4 1 269 108 30
8 2 70 29 28 13 0 45 0 61 23 16 3
65 383 39 83 1 1 17 0 45 21 4 8 1
0 22 42 1 6 1 0
"Viruses;Uroviricota;Caudoviricetes;Caudoviricetes__na;Herelleviridae;Herelleviridae__na;Herelleviridae_sp._cttEB8" 0 0 0 0 0 0 0 0 0 0 0 0 0
0 0 0 0 0 0 0 0 0 0 0 0 0
0 0 0 0 0 0 0 0 0 0 1 14 0
0 0 0 0 0 0 0 0 0 0 0 0 0
0 0 0 0 0 0 0 0 2 0 0 2 0
0 0 0 0 0 0 0
"Viruses;Uroviricota;Caudoviricetes;Caudoviricetes__na;Herelleviridae;Herelleviridae__na;Phage_f2b1" 0
0 0 0 0 0 0 0 0 0 0 0 0 0
0 0 0 0 0 0 0 0 0 0 0 0 0
0 0 0 0 0 0 0 0 0 0 0 0 0
0 0 0 0 0 0 0 0 0 0 0 0 0
0 0 0 0 0 0 0 0 1 0 0 0 0
0 0 0 0 0 0
"Viruses;Uroviricota;Caudoviricetes;Caudoviricetes__na;Herelleviridae;Herelleviridae__uc;Herelleviridae__uc"
0 0 0 0 0 2 0 0 0 0 0 0 0
0 0 0 0 0 0 0 0 0 0 1 0 0
0 0 0 0 0 0 0 0 0 0 0 0 0
0 0 0 0 0 0 0 0 0 0 0 0 0

0 0 0 0 0 0 0 0 0 0 0 0 0
0 0 0 0 0 0 0
"Viruses;Uroviricota;Caudoviricetes;Caudoviricetes__na;Herelleviridae;Kayvirus;Kayvirus__uc" 0 0
0 0 0 0 0 0 0 0 0 0 0 0 0
0 0 0 0 0 0 0 0 0 0 0 0 0
0 0 0 0 0 0 0 2 0 0 0 0 0
0 0 0 0 0 0 0 0 0 0 0 0 0
0 0 0 0 0 0 0 0 0 0 0 0 0
0 0 0 0 0
"Viruses;Uroviricota;Caudoviricetes;Caudoviricetes__na;Herelleviridae;Kochikohdavirus;Enterococcus_phage_1
56" 0 0 0 0 0 0 0 0 0 0 0 0 0
0 0 0 0 0 0 0 0 0 0 0 0 0
0 0 0 0 0 0 0 0 0 0 0 0 0
0 0 0 0 0 0 0 0 0 0 0 0 0
0 0 4 2 0 0 0 0 0 0 0 0 0
0 0 0 0 0 0 0
"Viruses;Uroviricota;Caudoviricetes;Caudoviricetes__na;Herelleviridae;Matervirus;Matervirus_mater" 0
0 0 1 0 0 0 0 0 0 0 0 0 0
0 0 0 0 0 0 0 0 0 0 0 0 0
0 0 0 0 0 0 0 0 0 0 0 0 0
0 0 0 0 0 0 0 0 0 0 0 0 0
0 0 0 0 0 0 0 0 0 0 0 0 0
0 0 0 0 0 0
"Viruses;Uroviricota;Caudoviricetes;Caudoviricetes__na;Herelleviridae;Nitunavirus;Bacillus_phage_035JT004"
0 0 0 0 0 0 0 0 0 0 0 0 0
0 0 0 0 0 0 0 0 0 0 0 0 0
0 0 0 0 0 0 0 0 0 0 0 0 0
0 0 0 0 0 0 0 0 0 0 0 0 0
0 0 0 0 0 0 0 0 0 1 0 0 0
0 0 0 0 0 0 0
"Viruses;Uroviricota;Caudoviricetes;Caudoviricetes__na;Herelleviridae;Schiekvirus;Enterococcus_phage_iF6"
0 0 0 0 0 0 0 0 0 0 0 0 0
0 0 0 0 0 0 0 0 0 0 0 0 0
0 0 0 0 0 1 0 0 0 0 0 0 0
0 0 0 0 0 0 0 0 0 0 0 0 0
0 0 0 0 0 0 0 0 0 0 0 0 0
0 0 0 0 0 0 0
"Viruses;Uroviricota;Caudoviricetes;Caudoviricetes__na;Herelleviridae;Sepunavirus;Sepunavirus__uc" 0
0 0 0 0 0 0 0 0 0 0 0 0 0
0 0 0 0 0 0 0 0 0 0 0 0 0
0 0 0 0 0 0 0 0 0 0 0 0 0
0 0 0 0 0 0 0 0 0 0 0 0 0
0 0 0 0 0 0 57 0 0 0 0 0 0
0 0 0 0 0 0
"Viruses;Uroviricota;Caudoviricetes;Caudoviricetes__na;Herelleviridae;Sepunavirus;Staphylococcus_phage_Tw
illingate" 0 0 0 0 0 0 0 0 0 0 0 0
0 0 0 0 0 0 0 0 0 0 0 0 0
0 0 0 0 0 0 0 0 0 0 0 0 0
0 0 0 0 0 0 0 0 11 0 0 0 0

0 0 0 0 0 0 0 0 0 0 0 0 0
0 0 0 0 0 0 0 0
"Viruses;Uroviricota;Caudoviricetes;Caudoviricetes__na;Herelleviridae;Silviavirus;Silviavirus__uc" 0
0 1 0 0 0 0 0 0 1 0 0 0 0
0 0 0 0 0 0 0 0 0 0 0 0 0
0 0 0 0 0 0 0 0 0 0 0 0 0
0 11 0 0 0 0 0 0 0 0 0 0 0
0 0 0 0 0 0 0 0 0 0 0 0 0
0 0 0 0 0 0
"Viruses;Uroviricota;Caudoviricetes;Caudoviricetes__na;Herelleviridae;Siminovitchvirus;Siminovitchvirus__uc"
0 0 1 0 0 0 0 0 0 0 0 0 0
0 0 0 0 0 0 0 0 0 0 0 0 0
0 0 0 0 0 0 0 0 0 0 0 0 0
0 0 0 0 0 0 0 0 0 0 0 0 0
0 0 0 0 0 0 0 0 0 0 0 0 0
0 0 0 0 0 0 0
"Viruses;Uroviricota;Caudoviricetes;Caudoviricetes__na;Herelleviridae;Tsarbombavirus;Bacillus_phage_QCM8"
0 0 0 0 0 21 0 0 0 0 0 0 0
0 0 0 0 0 0 0 0 0 0 0 0 0
0 0 0 0 0 0 0 0 0 0 0 0 0
0 0 0 0 0 0 0 0 0 0 0 0 0
0 0 0 0 0 0 0 0 0 0 0 0 0
0 0 0 0 0 0 0
"Viruses;Uroviricota;Caudoviricetes;Caudoviricetes__na;Herelleviridae;Tsarbombavirus;Bacillus_phage_vB_Bsp
M_MarvelLand" 0 0 0 0 0 1 0 0 0 0 0
0 0 0 0 0 0 0 0 0 0 0 0 0
0 0 0 0 0 0 0 0 0 0 0 0 0
0 0 0 0 0 0 0 0 0 0 0 0 0
0 0 0 0 0 0 0 0 0 0 0 0 0
0 0 0 0 0 0 0 0 0
"Viruses;Uroviricota;Caudoviricetes;Caudoviricetes__na;Kyanoviridae;Atlauavirus;Atlauavirus__uc" 0
0 0 0 0 3 0 0 0 0 0 0 0 0
0 0 0 0 0 0 0 0 0 0 0 0 0
0 0 0 0 0 0 0 0 0 0 0 0 0
0 0 0 0 0 0 0 0 0 0 0 0 0
0 0 0 0 0 0 0 0 0 0 0 0 0
0 0 0 0 0 0
"Viruses;Uroviricota;Caudoviricetes;Caudoviricetes__na;Kyanoviridae;Kyanoviridae__na;Cyanophage_P-
RSM6" 0 0 0 0 0 0 0 0 0 1 0 0 0
0 0 0 0 0 0 0 0 0 0 0 0 0
0 0 0 0 0 0 0 0 0 0 0 0 0
0 0 0 0 0 0 0 0 0 0 0 0 0
0 0 0 0 0 0 0 0 0 0 0 0 0
0 0 0 0 0 0 0
"Viruses;Uroviricota;Caudoviricetes;Caudoviricetes__na;Kyanoviridae;Kyanoviridae__na;Synechococcus_phage
_S-BM3" 0 0 1 0 0 0 0 0 0 0 0 0
0 0 0 0 0 0 0 0 0 0 0 0 0
0 0 0 0 0 0 0 0 0 0 0 0 0
0 0 0 0 0 0 0 0 0 0 0 0 0

0 0 0 0 0 0 0 0 0 0 0 0 0
0 0 0 0 0 0 0 0
"Viruses;Uroviricota;Caudoviricetes;Caudoviricetes__na;Kyanoviridae;Kyanoviridae__na;Synechococcus_phage_S-CREM1" 0 0 0 0 0 12 0 0 0 0 0 0
0 0 0 0 0 0 0 0 0 0 0 0 0
0 0 0 0 0 0 0 0 0 0 0 0 0
0 0 0 0 0 0 0 0 0 0 0 0 0
0 0 0 0 0 0 0 0 0 0 0 0 0
0 0 0 0 0 0 0 0
"Viruses;Uroviricota;Caudoviricetes;Caudoviricetes__na;Kyanoviridae;Kyanoviridae__na;Synechococcus_phage_S-SRM01" 0 0 0 0 0 0 0 0 0 0 0 0
0 0 0 0 0 0 0 0 0 0 0 0 0
0 0 0 0 0 0 0 0 0 0 0 0 0
0 0 0 0 0 0 1 0 0 0 0 0 0
0 0 0 0 0 0 0 0 0 0 0 0 0
0 0 0 0 0 0 0 0
"Viruses;Uroviricota;Caudoviricetes;Caudoviricetes__na;Kyanoviridae;Kyanoviridae__uc;Kyanoviridae__uc"
0 0 0 0 1 0 0 0 0 2 0 0 0
0 0 0 0 0 0 0 0 0 0 0 0 0
0 0 0 0 0 0 0 0 4 0 0 0 0
0 0 0 0 0 0 0 0 0 0 0 0 0
0 0 0 0 0 0 0 0 0 0 0 0 0
0 0 0 0 0 0 0
"Viruses;Uroviricota;Caudoviricetes;Caudoviricetes__na;Kyanoviridae;Palaemonvirus;Palaemonvirus_pssm7"
0 0 0 0 5 0 0 0 0 0 0 0 0
0 0 0 0 0 0 0 0 0 0 0 0 0
0 0 0 0 0 0 0 0 0 0 0 0 0
0 0 0 0 0 0 0 0 0 0 0 0 0
0 0 0 0 0 0 0 0 0 0 0 0 0
0 0 0 0 0 0 0
"Viruses;Uroviricota;Caudoviricetes;Caudoviricetes__na;Kyanoviridae;Ronodorvirus;Ronodorvirus_pssm3"
0 0 0 0 1 0 0 0 0 0 0 0 0
0 0 0 0 0 0 0 0 0 0 0 0 0
0 0 0 0 0 0 0 0 0 0 0 0 0
0 0 0 0 0 0 0 0 0 0 0 0 0
0 0 0 0 0 0 0 0 0 0 0 0 0
0 0 0 0 0 0 0
"Viruses;Uroviricota;Caudoviricetes;Caudoviricetes__na;Mesyanzhinovviridae;Keylargovirus;Keylargovirus_JL001" 0 0 0 2 0 0 0 0 0 0 0 0 0
0 0 0 0 0 0 0 0 0 1 0 0 0
0 0 0 0 0 0 0 0 0 0 0 0 0
0 0 0 0 0 0 0 0 0 0 0 0 0
0 0 0 0 0 0 0 0 0 0 0 0 0
0 0 0 0 0 0 0
"Viruses;Uroviricota;Caudoviricetes;Caudoviricetes__na;Mesyanzhinovviridae;Mesyanzhinovviridae__na;Janthinobacterium_phage_vB_JliM-Donnerlittchen" 0 0 0 1 0 0 0 0
0 0 0 0 0 0 0 1 0 0 0 0 0
0 0 0 0 0 0 0 0 0 0 0 0 0
1 0 0 0 0 0 0 0 0 0 0 0 0

0 0 0 0 0 0 0 0 0 0 0 0 0
0 0 0 0 0 0 0 0 0 0 0 0
"Viruses;Uroviricota;Caudoviricetes;Caudoviricetes__na;Mesyanzhinovviridae;Mesyanzhinovviridae__na;Pseudomonas_phage_Epa19" 0 0 2 0 0 0 0 0 0 0
0 0 0 0 0 0 0 0 0 0 0 0 0
0 0 0 0 0 0 0 0 0 0 0 0 0
0 0 0 0 0 0 0 0 0 0 0 0 0
0 0 0 0 0 0 0 0 0 0 0 0 0
0 0 0 0 0 0 0 0 0 0
"Viruses;Uroviricota;Caudoviricetes;Caudoviricetes__na;Mesyanzhinovviridae;Mesyanzhinovviridae__na;Stenotrophomonas_phage_A1432" 0 0 0 0 0 0 0 0 0 0
0 0 0 0 0 0 0 0 0 0 0 0 0
0 0 0 0 0 0 0 0 0 0 0 0 0
0 0 0 0 0 1 0 0 0 0 0 0 0
0 0 0 0 0 0 0 0 0 0 0 0 0
0 0 0 0 0 0 0 0 0 0
"Viruses;Uroviricota;Caudoviricetes;Caudoviricetes__na;Mesyanzhinovviridae;Mesyanzhinovviridae__na;Stenotrophomonas_phage_Sonora" 0 0 0 0 0 1 0 0 0 0
0 0 0 0 0 0 0 0 0 0 0 0 0
0 0 0 0 0 0 0 0 0 0 0 0 0
0 0 0 0 0 0 0 0 0 0 0 0 0
0 0 0 0 0 0 0 0 0 0 0 0 0
0 0 0 0 0 0 0 0 0 0
"Viruses;Uroviricota;Caudoviricetes;Caudoviricetes__na;Mesyanzhinovviridae;Mesyanzhinovviridae__uc;Mesyanzhinovviridae__uc" 0 0 0 0 5 6 0 0 0 0 0
0 0 0 0 0 0 0 0 0 0 0 0 0
0 0 0 0 0 0 0 0 0 0 0 0 0
0 0 0 0 0 0 0 0 0 0 1 0 0
0 0 0 0 0 0 0 0 0 0 0 0 0
0 0 0 0 0 0 0 0 0
"Viruses;Uroviricota;Caudoviricetes;Caudoviricetes__na;Mesyanzhinovviridae;Vojvodinavirus;Vojvodinavirus__uc" 0 0 0 1 5 2 0 0 0 0 0 0 0
0 0 0 0 0 0 0 0 0 0 0 0 0
0 0 0 0 0 0 0 0 0 0 0 0 0
0 0 0 0 0 0 0 0 0 0 0 0 0
0 0 0 0 0 0 0 0 0 0 0 0 0
0 0 0 0 0 0 0
"Viruses;Uroviricota;Caudoviricetes;Caudoviricetes__na;Mesyanzhinovviridae;Xooduovirus;Xooduovirus_Xoosp2" 0 0 0 0 0 0 0 0 2 0 0 0 0
0 0 0 0 0 0 0 0 0 0 0 0 0
0 0 0 0 0 0 0 0 0 0 0 0 0
0 0 0 0 0 0 0 0 0 0 0 0 0
0 0 0 0 0 0 0 0 0 0 0 0 0
0 0 0 0 0 0 0
"Viruses;Uroviricota;Caudoviricetes;Caudoviricetes__na;Molycolviridae;Mollyvirus;Mollyvirus__uc" 0
0 0 0 2 0 0 0 0 0 0 0 0 0
0 0 0 0 0 0 0 0 0 0 0 0 0
0 0 0 0 0 0 0 0 0 0 0 0 0
0 0 0 0 0 0 0 0 0 0 0 0 0

0 0 0 0 0 0 0 0 0 0 0 0 0
0 0 0 0 0 0
"Viruses;Uroviricota;Caudoviricetes;Caudoviricetes__na;Pachyviridae;Bacelvirus;Bacelvirus_phi46tres" 0
0 0 0 0 0 0 0 0 0 0 0 0 0
0 0 0 0 0 0 0 0 0 0 0 0 0
0 0 0 0 0 0 0 0 0 0 0 0 0
0 0 0 0 0 0 0 0 0 0 0 0 1
0 0 0 0 0 0 0 0 0 0 0 0 0
0 0 0 0 0 0
"Viruses;Uroviricota;Caudoviricetes;Caudoviricetes__na;Pachyviridae;Baltivirus;Baltivirus_phi19tres" 0
0 0 0 16 0 0 0 0 0 0 0 0 0
0 0 0 0 0 0 0 0 0 0 0 0 0
0 0 0 0 0 0 0 0 0 0 0 0 0
0 0 0 0 0 0 0 0 0 0 0 0 0
0 0 0 0 0 0 0 0 0 0 0 0 0
0 0 0 0 0 0
"Viruses;Uroviricota;Caudoviricetes;Caudoviricetes__na;Peduoviridae;Peduoviridae__na;Escherichia_phage_vB
_EcoM-813R9" 0 0 0 1 0 0 0 0 0 0 0 0
0 0 0 0 0 0 1 0 0 0 0 0 0
0 0 0 0 0 0 0 0 0 0 0 0 0
0 0 0 0 0 0 0 0 0 0 0 0 0
0 0 0 0 0 0 0 0 0 7 1 0 0
0 0 0 0 0 0 0 0
"Viruses;Uroviricota;Caudoviricetes;Caudoviricetes__na;Peduoviridae;Peduovirus;Escherichia_phage_vB_EcoM
-720R8" 0 0 0 0 0 0 0 0 0 0 0 0 0
0 0 0 0 0 0 0 0 0 0 0 0 0
0 0 0 0 0 0 0 0 0 0 0 0 0
0 0 0 0 0 0 0 0 0 0 0 0 0
0 0 0 0 0 0 0 0 13 2 0 0 0
0 0 0 0 0 0 0
"Viruses;Uroviricota;Caudoviricetes;Caudoviricetes__na;Peduoviridae;Quadragintavirus;Quadragintavirus_ev1
29" 0 0 0 1 0 0 0 0 0 0 0 0 0
0 0 0 0 0 0 0 0 0 0 0 0 0
0 0 0 0 0 0 0 0 0 0 0 0 0
0 0 0 0 0 0 0 0 0 0 0 0 0
0 0 0 0 0 0 0 0 0 0 0 0 0
0 0 0 0 0 0 0
"Viruses;Uroviricota;Caudoviricetes;Caudoviricetes__na;Peduoviridae;Xuanwuvirus;Xuanwuvirus_xv503458"
0 0 0 0 0 0 0 0 0 0 0 0 0
0 0 0 0 0 0 0 0 0 0 0 0 0
0 0 0 0 0 0 0 0 0 0 0 0 0
0 0 0 0 0 0 0 0 0 0 0 0 0
0 0 0 0 0 0 0 0 1 0 0 0 0
0 0 0 0 0 0 0
"Viruses;Uroviricota;Caudoviricetes;Caudoviricetes__na;Rountreeviridae;Andhravirus;Andhravirus__uc"0
0 0 0 0 0 0 0 0 0 27 0 0 0
0 0 0 0 0 0 0 0 0 0 0 0 0
0 0 0 0 0 0 0 0 0 0 0 0 0
0 0 0 0 0 0 51 0 0 0 0 0 0

0 0 0 0 0 0 0 0 0 0 0 0 0

0 0 0 0 0 0

"Viruses;Uroviricota;Caudoviricetes;Caudoviricetes__na;Rountreeviridae;Andhravirus;Andhravirus_andhra"

0 0 0 0 0 0 0 0 0 0 26 0 0

0 0 0 0 0 0 0 0 0 0 0 0 0

0 0 0 0 0 0 0 0 0 0 0 0 0

0 0 0 0 0 0 0 17 0 0 0 0 0

0 0 0 0 0 0 0 0 0 0 0 0 0

0 0 0 0 0 0 0

"Viruses;Uroviricota;Caudoviricetes;Caudoviricetes__na;Rountreeviridae;Andhravirus;Andhravirus_St134"

0 0 0 0 0 0 0 0 0 0 0 0 0

0 0 0 0 0 0 0 0 0 0 0 0 0

0 0 0 0 0 0 0 0 0 0 0 0 0

0 0 0 0 0 0 0 72 0 0 0 0 0

0 0 0 0 0 0 0 0 0 0 0 0 0

0 0 0 0 0 0 0

"Viruses;Uroviricota;Caudoviricetes;Caudoviricetes__na;Rountreeviridae;Andhravirus;Staphylococcus_phage_v

B_SepP_BE03" 0 0 0 0 0 0 0 0 0 0 0 0

0 0 0 0 0 0 0 0 0 0 0 0 0

0 0 0 0 0 0 0 0 0 0 1 0 0

0 0 0 0 0 0 0 0 0 0 0 0 0

0 0 0 0 0 0 0 0 0 0 0 0 0

0 0 0 0 0 0 0 0

"Viruses;Uroviricota;Caudoviricetes;Caudoviricetes__na;Rountreeviridae;Andhravirus;Staphylococcus_phage_v

B_SurP-PSU3" 0 0 0 0 0 0 0 0 0 0 0 0

0 0 0 0 0 0 0 0 0 0 0 0 0

0 0 0 0 0 0 0 0 0 0 2 0 0

0 0 0 0 0 0 0 0 23 0 0 0 0

0 0 0 0 0 0 0 0 0 0 0 0 0

0 0 0 0 0 0 0 0

"Viruses;Uroviricota;Caudoviricetes;Caudoviricetes__na;Salasmaviridae;Beecentumtrevirus;Beecentumtrevirus_

_uc" 0 0 0 0 0 0 0 0 0 0 0 0 0

0 0 0 0 0 0 2 0 0 0 0 0 0

0 0 0 0 0 0 0 0 0 0 0 0 0

0 0 0 0 0 0 0 0 0 0 0 0 0

0 5 0 0 0 0 0 0 0 0 0 0 0

0 1 0 0 0 0 0

"Viruses;Uroviricota;Caudoviricetes;Caudoviricetes__na;Salasmaviridae;Beecentumtrevirus;Beecentumtrevirus_

B103" 0 0 0 0 0 0 0 0 0 0 0 0 0

0 0 0 0 0 0 0 0 0 0 0 0 0

0 0 0 0 0 0 0 0 0 0 0 0 0

0 0 0 0 0 0 0 0 0 0 0 0 0

0 0 1 0 0 0 0 0 0 0 0 0 0

0 0 0 0 0 0 0

"Viruses;Uroviricota;Caudoviricetes;Caudoviricetes__na;Salasmaviridae;Gaunavirus;Gaunavirus_GA1" 0

0 0 0 0 0 0 0 0 0 0 0 0 0

0 0 0 0 0 0 0 0 0 0 0 0 0

0 0 0 0 0 0 0 0 0 0 0 0 0

0 0 0 0 0 0 0 1 0 0 0 0 0

0 0 0 0 0 0 0 0 0 0 0 0 0
0 0 0 0 0 0
"Viruses;Uroviricota;Caudoviricetes;Caudoviricetes__na;Salasmaviridae;Gaunavirus;Gaunavirus_SRT01hs"
0 0 0 0 0 0 0 0 0 0 0 0 0
0 0 0 0 0 0 0 0 0 0 0 0 0
0 0 0 0 0 0 0 0 0 0 0 0 0
0 0 0 0 0 0 0 0 2 0 0 0 0
0 0 0 0 0 0 0 0 0 0 0 0 0
0 0 0 0 0 0 0
"Viruses;Uroviricota;Caudoviricetes;Caudoviricetes__na;Salasmaviridae;Salasmaviridae__na;Bacillus_phage_vB
_BaeroP_SYYB1" 0 0 0 0 0 0 0 0 0 0 0
0 0 0 0 0 0 0 0 0 0 0 0 0
0 0 0 0 0 0 0 0 0 0 0 0 0
0 0 0 0 0 0 0 0 0 0 1 0 0
0 0 0 0 0 0 0 0 0 0 0 0 0
0 0 0 0 0 0 0 0 0
"Viruses;Uroviricota;Caudoviricetes;Caudoviricetes__na;Salasmaviridae;Salasmaviridae__uc;Salasmaviridae__u
c" 0 0 0 0 0 0 0 0 0 0 0 0 0
0 0 0 0 0 0 0 0 0 0 0 0 0
0 0 0 0 0 0 0 0 0 0 0 0 0
0 0 0 0 0 0 0 0 0 0 0 0 0
0 1 0 0 0 0 0 0 0 0 0 0 0
0 0 0 0 0 0 0
"Viruses;Uroviricota;Caudoviricetes;Caudoviricetes__na;Salasmaviridae;Salasvirus;Salasvirus__uc" 0
0 0 0 0 0 0 0 0 0 0 0 0 0
0 0 0 2 0 0 0 0 0 0 0 0 0
0 0 0 0 0 0 0 0 0 0 0 0 0
0 0 0 0 0 0 0 0 0 0 0 0 0
0 0 0 0 0 0 0 0 0 0 0 0 0
0 0 0 0 0 0
"Viruses;Uroviricota;Caudoviricetes;Caudoviricetes__na;Schitoviridae;Aoqinvirus;Aoqinvirus_RD1410W101"
0 0 1 0 0 9 0 0 0 0 0 0 0
0 0 0 0 0 0 0 2 0 0 0 0 0
0 0 0 0 0 0 0 0 0 0 0 0 0
0 0 0 0 0 0 0 0 0 0 0 0 0
0 0 0 0 0 0 0 0 0 0 0 0 0
0 0 0 0 0 0 0
"Viruses;Uroviricota;Caudoviricetes;Caudoviricetes__na;Schitoviridae;Aorunvirus;Aorunvirus__uc" 0
0 0 0 0 3 0 0 0 0 0 0 0 0
0 0 0 0 0 0 0 0 0 0 0 0 0
0 0 0 0 0 0 0 0 0 0 0 0 0
0 0 0 0 0 0 0 0 0 0 0 0 0
0 0 0 0 0 0 0 0 0 0 0 0 0
0 0 0 0 0 0
"Viruses;Uroviricota;Caudoviricetes;Caudoviricetes__na;Schitoviridae;Baltimorevirus;Baltimorevirus_DFL12"
0 0 0 0 0 1 0 0 0 0 0 0 0
0 0 0 0 0 0 0 0 0 0 0 0 0
0 0 0 0 0 0 0 0 0 0 0 0 0
0 0 0 0 0 0 0 0 0 0 0 0 0

0 0 0 0 0 0 0 0 0 0 0 0 0
0 0 0 0 0 0 0
"Viruses;Uroviricota;Caudoviricetes;Caudoviricetes__na;Schitoviridae;Cbunavirus;Cbunavirus__uc" 0
0 1 0 1 0 0 0 0 0 0 0 0 0
0 0 0 0 0 0 0 0 0 0 0 0 0
0 0 0 0 0 0 0 0 0 0 0 0 0
0 0 0 0 0 0 0 0 0 0 0 0 0
0 0 0 0 0 0 0 0 0 0 0 0 0
0 0 0 0 0 0
"Viruses;Uroviricota;Caudoviricetes;Caudoviricetes__na;Schitoviridae;Cbunavirus;Cbunavirus_A41" 0
0 0 0 0 1 0 0 0 0 0 0 0 0
0 0 0 0 0 0 0 0 0 0 0 0 0
0 0 0 0 0 0 0 0 0 0 0 0 0
0 0 0 0 0 0 0 0 0 0 0 0 0
0 0 0 0 0 0 0 0 0 0 0 0 0
0 0 0 0 0 0
"Viruses;Uroviricota;Caudoviricetes;Caudoviricetes__na;Schitoviridae;Dongdastvirus;Dongdastvirus_Axy24"
0 0 0 1 0 0 0 0 0 0 0 0 0
0 0 0 0 0 0 0 0 0 0 0 0 0
0 0 0 0 0 0 0 0 0 0 0 0 0
0 0 0 0 0 0 0 0 0 0 0 0 0
0 0 0 0 0 0 0 0 0 0 0 0 0
0 0 0 0 0 0 0
"Viruses;Uroviricota;Caudoviricetes;Caudoviricetes__na;Schitoviridae;Eceepunavirus;Eceepunavirus_EcP1"
0 0 0 0 8 0 0 0 0 0 0 0 0
0 0 0 0 0 0 0 0 0 0 0 0 0
0 0 0 0 0 0 0 0 0 0 0 0 0
0 0 0 0 0 0 0 0 0 0 0 0 0
0 0 0 0 0 0 0 0 0 0 0 0 0
0 0 0 0 0 0 0
"Viruses;Uroviricota;Caudoviricetes;Caudoviricetes__na;Schitoviridae;Electravirus;Electravirus_Sbp1" 0
0 0 0 6 0 0 0 0 0 0 0 0 0
0 0 0 0 0 0 0 0 0 0 0 0 0
0 0 0 0 0 0 0 0 0 0 0 0 0
0 0 0 0 0 0 0 0 0 0 0 0 0
0 0 0 0 0 0 0 0 0 0 0 0 0
0 0 0 0 0 0
"Viruses;Uroviricota;Caudoviricetes;Caudoviricetes__na;Schitoviridae;Enquatrovirus;Enquatrovirus__uc"0
0 0 0 0 0 0 0 0 0 0 0 0 0
0 0 0 0 0 0 0 0 0 0 0 0 0
0 0 0 0 0 0 0 0 3 0 0 0 0
0 0 0 0 0 0 0 1 0 0 0 0 0
0 0 0 0 0 0 0 0 0 0 0 0 0
0 0 0 0 0 0
"Viruses;Uroviricota;Caudoviricetes;Caudoviricetes__na;Schitoviridae;Enquatrovirus;uncultured_N4_virus"
0 0 0 0 0 0 0 0 0 0 0 0 0
0 0 0 0 0 0 0 0 0 0 0 0 0
0 0 0 0 0 0 0 0 0 0 0 0 0
0 0 0 0 0 0 0 0 6 0 0 0 0

0 0 0 0 0 0 0 0 0 0 0 0 0
0 0 0 0 0 0 0
"Viruses;Uroviricota;Caudoviricetes;Caudoviricetes__na;Schitoviridae;Exceevirus;Exceevirus_Xc38" 0
0 0 0 2 0 0 0 0 1 0 0 0 0
0 0 0 0 0 0 0 0 0 0 0 0 0
0 0 0 0 0 0 0 0 0 0 0 0 0
0 0 0 0 0 0 0 0 0 0 0 0 0
0 0 0 0 0 0 0 0 0 0 0 0 0
0 0 0 0 0 0
"Viruses;Uroviricota;Caudoviricetes;Caudoviricetes__na;Schitoviridae;Gamaleyavirus;Gamaleyavirus__uc"
0 0 0 0 0 0 0 0 0 0 0 0 0
0 0 0 0 0 0 0 0 0 0 0 0 0
0 0 0 0 0 0 0 0 0 5 0 0 0
0 0 0 0 0 0 0 0 0 0 0 0 0
0 0 0 0 0 0 0 0 0 0 0 0 0
0 0 0 0 0 0 0
"Viruses;Uroviricota;Caudoviricetes;Caudoviricetes__na;Schitoviridae;Huelvavirus;Huelvavirus_ort11" 0
0 0 0 0 0 0 0 0 0 0 0 0 0
0 0 0 0 0 0 0 0 0 0 0 0 0
0 0 0 0 0 0 0 0 0 0 0 0 0
0 0 0 0 0 0 0 2 0 0 0 0 0
0 0 0 0 0 0 0 0 0 0 0 0 0
0 0 0 0 0 0
"Viruses;Uroviricota;Caudoviricetes;Caudoviricetes__na;Schitoviridae;Johnsonvirus;Johnsonvirus__uc" 4
0 0 0 0 22 0 0 0 0 0 0 0 0
0 0 0 0 0 0 0 0 0 0 0 0 0
0 0 0 0 0 0 0 0 0 0 0 0 0
0 0 0 0 0 0 0 0 0 0 0 0 0
0 0 0 0 0 0 0 0 0 0 0 0 0
0 0 0 0 0 0
"Viruses;Uroviricota;Caudoviricetes;Caudoviricetes__na;Schitoviridae;Johnsonvirus;Johnsonvirus_frozen"
0 0 0 1 0 0 0 0 0 0 0 0 0
0 0 0 0 0 0 0 0 0 0 0 0 0
0 0 0 0 0 0 0 0 0 0 0 0 0
0 0 0 0 0 0 0 0 0 0 0 0 0
0 0 0 0 0 0 0 0 0 0 0 0 0
0 0 0 0 0 0 0
"Viruses;Uroviricota;Caudoviricetes;Caudoviricetes__na;Schitoviridae;Littlefixvirus;Littlefixvirus__uc" 0
0 0 1 0 0 0 0 0 0 0 0 0 0
0 0 0 0 0 0 0 0 0 0 0 0 0
0 0 0 0 0 0 0 0 0 0 0 0 0
0 0 0 0 0 0 0 0 0 0 0 0 0
0 0 0 0 0 0 0 0 0 0 0 0 0
0 0 0 0 0 0
"Viruses;Uroviricota;Caudoviricetes;Caudoviricetes__na;Schitoviridae;Littlefixvirus;Pseudomonas_phage_98Pfl
uR60PP" 0 0 0 1 0 0 0 0 0 0 0 0
0 0 0 0 0 0 0 0 0 0 0 0 0
0 0 0 0 0 0 0 0 0 0 0 0 0
0 0 0 0 0 0 0 0 0 2 0 0 0

0 0 0 0 0 0 0 0 0 0 0 0 0
0 0 0 0 0 0 0 0
"Viruses;Uroviricota;Caudoviricetes;Caudoviricetes__na;Schitoviridae;Litunavirus;Pseudomonas_phage_vB_Pa
eP_FBPa1" 0 0 0 2 0 0 0 0 0 0 0 0
0 0 0 0 0 0 0 0 0 0 0 0 0
0 0 0 0 0 0 0 0 0 0 0 0 0
0 0 0 2 0 0 0 0 0 0 0 0 0
0 0 0 0 0 0 0 0 0 0 0 0 0
0 0 0 0 0 0 0 0
"Viruses;Uroviricota;Caudoviricetes;Caudoviricetes__na;Schitoviridae;Luzseptimavirus;Luzseptimavirus__uc"
0 0 0 0 4 3 0 0 0 0 0 0 0
0 0 0 0 0 0 0 0 0 0 0 0 0
0 0 0 0 0 0 0 0 0 0 0 0 0
0 0 0 0 0 0 0 0 0 0 0 0 0
0 0 0 0 0 0 0 0 0 0 0 0 0
0 0 0 0 0 0 0
"Viruses;Uroviricota;Caudoviricetes;Caudoviricetes__na;Schitoviridae;Luzseptimavirus;Luzseptimavirus_KPP2
1" 0 0 0 0 1 0 0 0 0 0 0 0 0
0 0 0 0 0 0 0 0 0 0 0 0 0
0 0 0 0 0 0 0 0 0 0 0 0 0
0 0 0 0 0 0 0 0 0 0 0 0 0
0 0 0 0 0 0 0 0 0 0 0 0 0
0 0 0 0 0 0 0
"Viruses;Uroviricota;Caudoviricetes;Caudoviricetes__na;Schitoviridae;Matsuvirus;Matsuvirus_pYD6A" 0
0 0 0 0 0 0 0 0 0 0 0 0 0
0 0 0 0 0 0 0 0 0 0 0 0 0
0 0 0 0 0 0 0 0 0 0 0 0 0
0 0 0 0 0 0 0 0 0 0 0 0 0
0 0 0 0 0 0 0 0 0 0 0 0 0
0 0 0 0 1 0
"Viruses;Uroviricota;Caudoviricetes;Caudoviricetes__na;Schitoviridae;Mukerjeevirus;Mukerjeevirus__uc"
0 0 1 0 0 0 0 0 0 0 0 0 0
0 0 0 0 0 0 0 0 0 0 0 0 0
0 0 0 0 0 0 0 0 0 0 0 0 0
0 0 0 0 0 0 0 0 0 0 0 0 0
0 0 0 0 0 0 0 0 0 0 0 0 0
0 0 0 0 0 0 0
"Viruses;Uroviricota;Caudoviricetes;Caudoviricetes__na;Schitoviridae;Pacinivirus;Pacinivirus__uc" 0
0 0 0 2 0 0 0 0 0 0 0 0 0
0 0 0 0 0 0 0 0 0 0 0 0 0
0 0 0 0 0 0 0 0 0 0 0 0 0
0 0 0 0 0 0 0 0 0 0 0 0 0
0 0 0 0 0 0 0 0 0 0 0 0 0
0 0 0 0 0 0
"Viruses;Uroviricota;Caudoviricetes;Caudoviricetes__na;Schitoviridae;Pariacacavirus;Pariacacavirus__uc"
0 0 0 0 1 0 0 0 0 0 0 0 0
0 0 0 0 0 0 0 0 0 0 0 0 0
0 0 0 0 0 0 0 0 0 0 0 0 0
0 0 0 0 0 0 0 0 0 0 0 0 0

0 0 0 0 0 0 0 0 0 0 0 0 0
0 0 0 0 0 0 0
"Viruses;Uroviricota;Caudoviricetes;Caudoviricetes__na;Schitoviridae;Plymouthvirus;Dinoroseobacter_phage_v
B_DshP-R7L" 0 0 0 1 0 0 0 0 0 0 0 0
0 0 0 0 0 0 0 0 0 0 0 0 0
0 0 0 0 0 0 0 0 0 0 0 0 0
0 0 0 0 0 0 0 0 0 0 0 0 0
0 0 0 0 0 0 0 0 0 0 0 0 0
0 0 0 0 0 0 0 0
"Viruses;Uroviricota;Caudoviricetes;Caudoviricetes__na;Schitoviridae;Plymouthvirus;Roseovarius_Plymouth_p
odovirus_1" 0 0 0 0 0 0 0 0 0 0 0 0
0 0 0 0 0 0 0 0 0 0 0 0 0
0 0 0 0 0 0 0 0 0 0 0 0 0
0 0 0 3 0 0 0 0 0 0 0 0 0
0 0 0 0 0 0 0 0 0 0 0 0 0
0 0 0 0 0 0 0 0
"Viruses;Uroviricota;Caudoviricetes;Caudoviricetes__na;Schitoviridae;Pokkenvirus;Pokkenvirus_pokken"
0 0 0 2 3 0 0 0 0 0 0 0 0
0 0 0 0 0 0 0 0 0 0 0 0 0
0 0 0 0 0 0 0 0 0 0 0 0 0
0 0 0 0 0 0 0 0 0 0 0 0 0
0 0 0 0 0 0 0 0 0 0 0 0 0
0 0 0 0 0 0 0
"Viruses;Uroviricota;Caudoviricetes;Caudoviricetes__na;Schitoviridae;Pollockvirus;Pollockvirus_pollock"0
0 0 1 1 0 0 0 0 0 0 0 0 0
0 0 0 0 0 0 0 0 0 0 0 0 0
0 0 0 0 0 0 0 0 0 0 0 0 0
0 0 0 0 0 0 0 0 0 0 0 0 0
0 0 0 0 0 0 0 0 0 0 0 0 0
0 0 0 0 0 0
"Viruses;Uroviricota;Caudoviricetes;Caudoviricetes__na;Schitoviridae;Pomeroyivirus;Pomeroyivirus_V13"
0 0 0 1 0 1 0 0 0 0 0 0 0
0 0 0 0 0 0 0 0 0 0 0 0 0
0 0 0 0 0 0 0 0 0 0 0 0 0
0 0 0 0 0 0 0 0 0 0 0 0 0
0 0 0 0 0 0 0 0 0 0 0 0 0
0 0 0 0 0 0 0
"Viruses;Uroviricota;Caudoviricetes;Caudoviricetes__na;Schitoviridae;Pourcelvirus;Pourcelvirus__uc" 1
0 0 1 0 0 0 0 0 0 0 0 0 0
0 0 0 0 0 0 0 0 0 0 0 0 0
0 0 0 0 0 0 0 0 0 0 0 0 0
0 0 0 0 0 0 0 0 0 0 0 0 0
0 0 0 0 0 0 0 0 0 0 0 0 0
0 0 0 0 0 0
"Viruses;Uroviricota;Caudoviricetes;Caudoviricetes__na;Schitoviridae;Pourcelvirus;Pourcelvirus_Axy10" 0
0 0 0 0 3 0 0 0 0 0 0 0 0
0 0 0 0 0 0 0 0 0 0 0 0 0
0 0 0 0 0 0 0 0 0 0 0 0 0
0 0 0 0 0 0 0 0 0 0 0 0 0

0 0 0 0 0 0 0 0 0 0 0 0 0
0 0 0 0 0 0
"Viruses;Uroviricota;Caudoviricetes;Caudoviricetes__na;Schitoviridae;Raunefjordenvirus;Raunefjordenvirus_C
B2047B"0 0 0 0 3 2 0 0 0 0 0 0 0
0 0 0 0 0 0 0 0 0 0 0 0 0
0 0 0 0 0 0 0 0 0 0 0 0 0
0 0 0 0 0 0 0 0 0 0 0 0 0
0 0 0 0 0 0 0 0 0 0 0 0 0
0 0 0 0 0 0 0
"Viruses;Uroviricota;Caudoviricetes;Caudoviricetes__na;Schitoviridae;Riverridervirus;Riverridervirus_riverride
r" 1 0 0 0 0 0 0 0 0 0 0 0 0
0 0 0 0 0 0 0 0 0 0 0 0 0
0 0 0 0 0 0 0 0 0 0 0 0 0
0 0 4 0 0 0 0 0 6 0 0 0 0
0 0 0 0 0 0 0 0 0 0 0 0 0
0 0 0 0 0 0 0
"Viruses;Uroviricota;Caudoviricetes;Caudoviricetes__na;Schitoviridae;Sanyabayvirus;Sanyabayvirus_DS1410W
s06" 0 0 0 0 0 3 0 0 0 0 0 0 0
0 0 0 0 0 0 0 0 0 0 0 0 0
0 0 0 0 0 0 0 0 0 0 0 0 0
0 0 0 0 0 0 0 0 1 0 0 0 0
0 0 0 0 0 0 0 0 0 0 0 0 0
0 0 0 0 0 0 0
"Viruses;Uroviricota;Caudoviricetes;Caudoviricetes__na;Schitoviridae;Schitoviridae__na;Acinetobacter_phage_
nACB1"0 0 0 0 1 0 0 0 0 0 0 0 0
0 0 0 0 0 0 0 0 0 0 0 0 0
0 0 0 0 0 0 0 0 0 0 0 0 0
0 0 0 0 0 0 0 0 0 0 0 0 0
0 0 0 0 0 0 0 0 0 0 0 0 0
0 0 0 0 0 0 0
"Viruses;Uroviricota;Caudoviricetes;Caudoviricetes__na;Schitoviridae;Schitoviridae__na;Podoviridae_sp._ctda_
1" 0 0 2 2 7 0 0 0 0 0 0 0 0
0 0 0 0 0 0 0 0 0 0 0 0 0
0 0 0 0 0 0 0 0 0 2 0 0 0
0 0 0 0 0 0 0 0 0 0 0 0 0
0 0 0 0 0 0 0 0 0 0 0 0 0
0 0 0 0 0 0 0
"Viruses;Uroviricota;Caudoviricetes;Caudoviricetes__na;Schitoviridae;Schitoviridae__na;Stenotrophomonas_ph
age_C121" 0 0 0 0 8 0 0 0 0 0 0 0
0 0 0 0 0 0 0 0 0 0 0 0 0
0 0 0 0 0 0 0 0 0 0 0 0 0
0 0 0 0 0 0 0 0 0 0 0 0 0
0 0 0 0 0 0 0 0 0 0 0 0 0
0 0 0 0 0 0 0 0
"Viruses;Uroviricota;Caudoviricetes;Caudoviricetes__na;Schitoviridae;Schitoviridae__na;Stenotrophomonas_ph
age_Philippe" 0 0 0 0 0 0 0 0 0 0 0 0
0 0 0 0 0 0 0 0 0 0 0 0 0
0 0 0 0 0 0 0 0 0 0 0 0 0
0 0 0 0 0 0 0 0 0 3 0 0 0

0 0 0 0 0 0 0 0 0 0 0 0 0
0 0 0 0 0 0 0 0
"Viruses;Uroviricota;Caudoviricetes;Caudoviricetes__na;Schitoviridae;Schitoviridae__na;Vibrio_phage_vB_VhaP_PG11" 0 0 1 0 0 2 0 0 0 0 0 0
0 0 0 0 0 0 0 0 0 0 0 0 0
0 0 0 0 0 0 0 0 0 0 0 0 0
0 0 0 0 0 0 0 0 0 1 0 0 0
0 0 0 0 0 0 0 0 0 0 0 0 0
0 0 0 0 0 0 0 0
"Viruses;Uroviricota;Caudoviricetes;Caudoviricetes__na;Schitoviridae;Schitoviridae__uc;Schitoviridae__uc"
0 0 1 2 13 4 0 0 0 0 0 0 0
0 0 0 0 0 0 0 0 0 0 0 0 0
0 0 0 0 0 0 1 0 0 0 0 0 0
0 0 0 0 0 0 0 0 1 0 0 0 0
0 0 0 0 0 0 0 0 0 0 0 0 0
0 0 0 0 0 0 0
"Viruses;Uroviricota;Caudoviricetes;Caudoviricetes__na;Schitoviridae;Stoningtonvirus;Stoningtonvirus_VBP47"
0 0 0 0 4 0 0 0 0 0 0 0 0
0 0 0 0 0 0 0 0 0 0 0 0 0
0 0 0 0 0 0 0 0 0 0 0 0 0
0 0 0 0 0 0 0 0 0 0 0 0 0
0 0 0 0 0 0 0 0 0 0 0 0 0
0 0 0 0 0 0 0
"Viruses;Uroviricota;Caudoviricetes;Caudoviricetes__na;Schitoviridae;Waedenswilvirus;Waedenswilvirus_S6"
0 0 1 0 0 0 0 0 0 0 0 0 0
0 0 0 0 0 0 0 0 0 0 0 0 0
0 0 0 0 0 0 0 0 0 0 0 0 0
0 0 0 0 0 0 0 0 0 0 0 0 0
0 0 0 0 0 0 0 0 0 0 0 0 0
0 0 0 0 0 0 0
"Viruses;Uroviricota;Caudoviricetes;Caudoviricetes__na;Schitoviridae;Yonginvirus;Yonginvirus__uc" 0
0 0 1 0 3 0 0 0 0 0 0 0 0
0 0 0 0 0 0 0 0 0 0 0 0 0
0 0 0 0 0 0 0 0 0 0 0 0 0
0 0 0 0 0 0 0 0 0 0 0 2 0
0 0 0 0 0 0 0 0 0 0 0 0 0
0 0 0 0 0 0
"Viruses;Uroviricota;Caudoviricetes;Caudoviricetes__na;Schitoviridae;Yonginvirus;Yonginvirus_EaP8" 2
0 0 1 1 1 0 0 0 0 0 0 0 0
0 0 0 0 0 0 0 0 0 0 0 0 0
0 0 0 0 0 0 0 0 0 0 0 0 0
0 0 0 0 0 0 0 0 0 0 0 0 0
0 0 0 0 0 0 0 0 0 0 0 0 0
0 0 0 0 0 0
"Viruses;Uroviricota;Caudoviricetes;Caudoviricetes__na;Schitoviridae;Zurivirus;Zurivirus_zuri" 0 0
0 0 1 0 0 0 0 0 0 0 0 0 0
0 0 0 0 0 0 0 0 0 0 0 0 0
0 0 0 0 0 0 0 0 0 0 0 0 0
0 0 0 0 0 0 0 0 0 0 0 0 0

0 0 0 0 0 0 0 0 0 0 0 0 0
0 0 0 0 0
"Viruses;Uroviricota;Caudoviricetes;Caudoviricetes__na;Straboviridae;Angelvirus;Angelvirus__uc" 0
0 0 0 12 0 0 0 0 0 0 0 0 0
0 0 0 0 0 0 0 0 0 0 0 0 0
0 0 0 0 0 0 0 0 0 0 0 0 0
0 0 0 0 0 0 0 0 0 0 0 0 0
0 0 0 0 0 0 0 0 0 0 0 0 0
0 0 0 0 0 0
"Viruses;Uroviricota;Caudoviricetes;Caudoviricetes__na;Straboviridae;Bragavirus;Bragavirus__uc" 0
0 0 0 0 0 0 0 0 0 0 0 0 0
0 0 0 0 0 0 0 0 0 0 0 0 0
0 0 0 0 0 0 0 0 0 0 0 0 0
0 0 0 0 0 0 0 4 0 0 0 0 0
0 0 0 0 0 0 0 0 0 0 0 0 0
0 0 0 0 0 0
"Viruses;Uroviricota;Caudoviricetes;Caudoviricetes__na;Straboviridae;Ceceduovirus;Aeromonas_phage_AP1"
0 0 0 0 0 0 0 0 0 0 0 0 0
0 0 0 0 0 0 0 0 0 0 0 0 0
0 0 0 0 0 0 0 0 0 0 0 0 0
0 0 0 0 0 0 0 0 0 0 0 0 0
0 0 0 0 0 0 0 0 7 2 0 0 0
0 0 0 0 0 0 0
"Viruses;Uroviricota;Caudoviricetes;Caudoviricetes__na;Straboviridae;Ceceduovirus;Ceceduovirus__uc" 0
0 0 0 0 4 0 0 0 0 0 0 0 0
0 0 0 0 0 0 0 0 0 0 0 0 0
0 0 0 0 0 0 0 0 0 0 0 0 0
0 0 0 0 0 0 0 0 0 0 0 0 0
0 0 0 0 0 0 0 0 0 0 0 0 0
0 0 0 0 0 0
"Viruses;Uroviricota;Caudoviricetes;Caudoviricetes__na;Straboviridae;Ishigurovirus;Ishigurovirus_osborne"
0 0 0 0 0 0 0 0 0 0 0 0 0
0 0 0 0 0 0 0 0 0 0 0 0 0
0 0 0 0 0 0 0 0 0 0 0 0 0
0 0 0 0 0 0 0 0 1 0 0 0 0
0 0 0 0 0 0 0 0 0 0 0 0 0
0 0 0 0 0 0 0
"Viruses;Uroviricota;Caudoviricetes;Caudoviricetes__na;Straboviridae;Jiangsuvirus;Jiangsuvirus_pspyzu05"
0 0 0 0 2 0 0 0 0 0 0 0 0
0 0 0 0 0 0 0 0 0 0 0 0 0
0 0 0 0 0 0 0 0 0 0 0 0 0
0 0 0 0 0 0 0 0 0 0 0 0 0
0 0 0 0 0 0 0 0 0 0 0 0 0
0 0 0 0 0 0 0
"Viruses;Uroviricota;Caudoviricetes;Caudoviricetes__na;Straboviridae;Kanagawavirus;Kanagawavirus__uc"
0 0 0 0 0 0 0 0 1 0 0 0 0
0 0 0 0 0 0 0 0 0 0 0 0 0
0 0 0 0 0 0 0 0 0 0 0 0 0
0 0 0 0 0 0 0 0 0 0 0 0 0

0 0 0 0 0 0 0 0 0 0 0 0 0
0 0 0 0 0 0 0
"Viruses;Uroviricota;Caudoviricetes;Caudoviricetes__na;Straboviridae;Krischvirus;Krischvirus_RB49" 0
0 0 0 1 0 0 0 0 0 0 0 0 0
0 0 0 0 0 0 0 0 0 0 0 0 0
0 0 0 0 0 0 0 0 0 0 0 0 0
0 0 0 0 0 0 0 0 0 0 0 0 0
0 0 0 0 0 0 0 0 0 0 0 0 0
0 0 0 0 0 0
"Viruses;Uroviricota;Caudoviricetes;Caudoviricetes__na;Straboviridae;Lasallevirus;Acinetobacter_virus_Acj61"
0 0 0 0 0 0 0 0 0 0 128 3 0
0 0 0 0 0 0 0 0 0 0 0 0 0
0 0 0 0 0 0 0 0 0 0 0 0 0
0 0 0 0 0 0 0 0 0 0 0 0 0
0 0 0 0 0 0 0 0 0 0 0 0 0
0 0 0 0 0 0 0
"Viruses;Uroviricota;Caudoviricetes;Caudoviricetes__na;Straboviridae;Mosigvirus;Mosigvirus__uc" 0
0 0 2 0 0 0 0 0 0 0 0 0 0
0 0 0 0 0 0 0 0 0 0 0 0 0
0 0 0 0 0 0 0 0 0 0 0 0 0
0 0 0 0 0 0 0 0 0 0 0 0 0
0 0 0 0 0 0 0 0 0 0 0 0 0
0 0 0 0 0 0
"Viruses;Uroviricota;Caudoviricetes;Caudoviricetes__na;Straboviridae;Roskildevirus;Roskildevirus_cronus"
0 0 0 0 0 0 0 0 0 0 0 0 0
0 0 0 0 0 0 0 0 0 0 0 0 0
0 0 0 0 0 0 0 0 0 0 0 0 0
0 0 0 0 0 0 0 0 1 0 0 0 0
0 0 0 0 0 0 0 0 0 0 0 0 0
0 0 0 0 0 0 0
"Viruses;Uroviricota;Caudoviricetes;Caudoviricetes__na;Straboviridae;Schizotequatrovirus;Schizotequatrovirus
__uc" 0 0 0 2 0 0 0 0 0 0 0 0 0
0 0 0 0 0 0 0 0 0 0 0 0 0
0 0 0 0 0 0 0 0 0 0 0 0 0
0 0 0 0 0 0 0 0 0 0 0 0 0
0 0 0 0 0 0 0 0 0 0 0 0 0
0 0 0 0 0 0 0
"Viruses;Uroviricota;Caudoviricetes;Caudoviricetes__na;Straboviridae;Straboviridae__na;Acinetobacter_phage_
Ac42" 0 0 0 0 0 0 0 0 0 0 0 0 0
0 0 0 0 0 0 0 0 0 0 0 0 0
0 0 0 0 0 0 0 0 0 0 0 0 0
0 0 0 0 0 0 0 0 1 0 0 0 0
0 0 0 0 0 0 0 0 0 0 0 0 0
0 0 0 0 0 0 0
"Viruses;Uroviricota;Caudoviricetes;Caudoviricetes__na;Straboviridae;Straboviridae__na;Acinetobacter_virus_1
33" 0 0 0 0 0 0 0 0 0 0 0 0 0
0 0 0 0 0 0 0 0 0 0 0 0 0
0 0 0 0 0 0 0 0 0 1 0 0 0
0 0 0 0 0 0 0 0 0 0 0 0 0

0 0 0 0 0 0 0 0 0 0 0 0 0
0 0 0 0 0 0 0
"Viruses;Uroviricota;Caudoviricetes;Caudoviricetes__na;Straboviridae;Straboviridae__na;Panteoa_phage_Phyn
n" 0 0 0 0 2 0 0 0 0 0 0 0 0
0 0 0 0 0 0 0 0 0 0 0 0 0
0 0 0 0 0 0 0 0 0 0 0 0 0
0 0 0 0 0 0 0 0 1 0 0 0 0
0 0 0 0 0 0 0 0 0 0 0 0 0
0 0 0 0 0 0 0
"Viruses;Uroviricota;Caudoviricetes;Caudoviricetes__na;Straboviridae;Straboviridae__na;Providencia_phage_P
STCR6" 0 0 0 0 1 0 0 0 0 0 0 0 0
0 0 0 0 0 0 0 0 0 0 0 0 0
0 0 0 0 0 0 0 0 0 0 0 0 0
0 0 0 0 0 0 0 0 0 0 0 0 0
0 0 0 0 0 0 0 0 0 0 0 0 0
0 0 0 0 0 0 0
"Viruses;Uroviricota;Caudoviricetes;Caudoviricetes__na;Straboviridae;Straboviridae__na;Vibrio_phage_vB_Vm
eM-32" 0 0 0 0 0 0 0 0 0 0 0 0 0
0 0 0 0 0 0 0 0 0 0 0 0 0
0 0 0 0 0 0 0 0 0 0 0 0 0
0 0 0 0 0 0 0 0 3 0 0 0 0
0 0 0 0 0 0 0 0 0 0 0 0 0
0 0 0 0 0 0 0
"Viruses;Uroviricota;Caudoviricetes;Caudoviricetes__na;Straboviridae;Tequatrovirus;T4-like_cyanophage_sp."
0 0 0 0 2 0 0 0 0 0 0 0 0
0 0 0 0 0 0 0 0 0 0 0 0 0
0 0 0 0 0 0 0 0 0 0 0 0 0
0 0 0 0 0 0 0 0 0 0 0 0 0
0 0 0 0 0 0 0 0 0 0 0 0 0
0 0 0 0 0 0 0
"Viruses;Uroviricota;Caudoviricetes;Caudoviricetes__na;Straboviridae;Tequatrovirus;T4virus_environmental_sa
mple" 0 0 0 0 11 0 0 0 0 0 0 0 0
0 0 0 0 0 0 0 0 0 0 0 0 0
0 0 0 0 0 0 0 0 0 0 0 0 0
0 0 0 0 0 0 0 0 0 0 0 0 0
0 0 0 0 0 0 0 0 0 0 0 0 0
0 0 0 0 0 0 0
"Viruses;Uroviricota;Caudoviricetes;Caudoviricetes__na;Straboviridae;Tulanevirus;Tulanevirus__uc" 0
0 0 0 0 1 0 0 0 0 0 0 0 0
0 0 0 0 0 0 0 0 0 0 0 0 0
0 0 0 0 0 0 0 0 0 0 0 0 0
0 0 0 0 0 0 0 0 0 0 0 0 0
0 0 0 0 0 0 0 0 0 0 0 0 0
0 0 0 0 0 0
"Viruses;Uroviricota;Caudoviricetes;Caudoviricetes__na;Vilmaviridae;Lumosvirus;Lumosvirus__uc" 0
0 0 0 0 2 0 0 0 0 0 0 0 0
0 0 0 0 0 0 0 0 0 0 0 0 0
0 0 0 0 0 0 0 0 0 0 0 0 0
0 0 0 0 0 0 0 0 0 0 0 0 0

0 0 0 0 0 0 0 0 0 0 0 0 0
0 0 0 0 0 0
"Viruses;Uroviricota;Caudoviricetes;Caudoviricetes__na;Winoviridae;Pippivirus;Pippivirus__uc" 0 0
0 1 0 0 0 0 0 0 0 0 0 0 0
0 0 0 0 0 0 0 0 0 0 0 0 0
0 0 0 0 0 0 0 0 0 0 0 0 0
0 0 0 0 0 0 0 0 0 0 0 0 0
0 0 0 0 0 0 0 0 0 0 0 0 0
0 0 0 0 0
"Viruses;Uroviricota;Caudoviricetes;Caudoviricetes__na;Winoviridae;Winoviridae__uc;Winoviridae__uc"
0 0 0 0 0 1 0 0 0 0 0 0 0
0 0 0 1 0 0 0 0 0 1 0 0 0
0 0 0 0 0 0 0 0 0 0 0 0 0
0 0 0 0 0 0 0 0 0 0 0 0 0
0 0 0 0 0 0 0 0 0 0 0 0 0
0 0 0 0 0 0 0
"Viruses;Uroviricota;Caudoviricetes;Caudoviricetes__na;Zierdtviridae;Ceetrepovirus;Ceetrepovirus__uc" 0
0 0 0 0 0 0 0 0 0 0 0 0 0
0 0 0 0 0 0 0 0 0 0 0 0 0
0 0 0 0 0 0 0 3 0 0 0 0 0
0 0 0 0 0 0 0 0 0 0 0 0 0
0 0 0 0 0 0 0 0 0 0 0 0 0
0 0 0 0 0 0
"Viruses;Uroviricota;Caudoviricetes;Caudoviricetes__na;Zierdtviridae;Ceetrepovirus;Ceetrepovirus_kimchi1738
" 0 0 0 0 0 0 0 0 0 0 0 0 0
0 0 0 0 0 0 0 0 0 0 0 0 0
0 0 0 0 0 0 0 0 12 0 0 0 0
0 0 0 0 0 0 0 0 0 0 0 0 0
0 0 0 0 0 0 0 0 0 0 0 0 0
0 0 0 0 0 0 0
"Viruses;Uroviricota;Caudoviricetes;Caudoviricetes__na;Zierdtviridae;Ceetrepovirus;Corynebacterium_virus_C
3PO" 1 0 0 0 0 0 0 0 0 0 1 0 0
0 0 0 0 0 0 0 0 0 0 0 0 0
0 0 0 0 0 0 0 0 0 0 0 0 0
0 0 0 0 0 0 0 0 0 0 0 0 0
0 0 0 0 0 0 0 0 0 0 0 0 0
0 0 0 0 0 0 0
"Viruses;Uroviricota;Caudoviricetes;Caudoviricetes__na;Zierdtviridae;Zierdtviridae__na;Rhodococcus_phage_
ChewyVIII" 0 0 0 0 3 0 0 0 0 0 0 0
0 0 0 0 0 0 0 0 0 0 0 0 0
0 0 0 0 0 0 0 0 0 0 0 0 0
0 0 0 0 0 0 0 0 0 0 0 0 0
0 0 0 0 0 0 0 0 0 0 0 0 0
0 0 0 0 0 0 0 0
"Viruses;Uroviricota;Caudoviricetes;Caudoviricetes__na;Zobellviridae;Citrovirus;Citrovirus_coptotermitis"
0 0 1 1 2 0 0 0 0 0 0 0 0
0 0 0 0 0 0 0 0 0 0 0 0 0
0 0 0 0 0 0 0 0 0 0 0 0 0
0 0 0 0 0 0 0 0 0 0 0 0 0

0 0 0 0 0 0 0 0 0 0 0 0 0
0 0 0 0 0 0 0
"Viruses;Uroviricota;Caudoviricetes;Caudoviricetes__na;Zobellviridae;Paundecimvirus;Paundecimvirus__uc"
0 0 0 0 2 0 0 0 0 0 0 0 0
0 0 0 0 0 0 0 0 0 2 0 0 0
0 0 0 0 0 0 0 0 0 0 0 0 0
0 0 0 0 0 0 0 0 0 0 0 0 0
0 0 0 0 0 0 0 0 0 0 0 0 0
0 0 0 0 0 0 0
"Viruses;Uroviricota;Caudoviricetes;Caudoviricetes__na;Zobellviridae;Paundecimvirus;Paundecimvirus_PA11"
1 0 0 1 0 0 0 0 0 0 0 0 0
0 0 0 0 0 0 0 0 0 0 0 0 0
0 0 0 0 0 0 0 0 0 0 0 0 0
0 0 0 0 0 0 0 0 0 0 0 0 0
0 0 0 0 0 0 0 0 0 0 0 0 0
0 0 0 0 0 0 0
"Viruses;Uroviricota;Caudoviricetes;Caudoviricetes__na;Zobellviridae;Salinovirus;Salinovirus_utanense" 1
0 1 2 13 32 0 0 0 0 0 0 0 0
0 0 0 0 0 0 0 0 0 0 0 0 0
0 0 0 0 0 0 0 0 0 0 0 0 0
0 0 0 0 1 0 0 2 0 0 0 0 0
0 0 0 0 0 0 0 0 0 0 0 0 0
0 0 0 0 0 0
"Viruses;Uroviricota;Caudoviricetes;Caudoviricetes__na;Zobellviridae;Veravirus;Roseobacter_phage_CRP-4"
0 0 1 0 11 0 0 0 0 0 0 0 0
0 0 0 0 0 0 0 0 0 0 0 0 0
0 0 0 0 0 0 0 0 0 0 0 0 0
0 0 0 0 0 0 0 0 1 0 0 0 0
0 0 0 0 0 0 0 0 0 0 0 0 0
0 0 0 0 0 0 0
"Viruses;Uroviricota;Caudoviricetes;Caudoviricetes__na;Zobellviridae;Veravirus;Roseobacter_phage_CRP-5"
0 0 0 0 0 0 0 0 0 0 0 0 0
0 0 0 0 0 0 0 0 0 0 0 0 0
0 0 0 0 0 0 0 0 0 0 0 0 0
0 0 0 0 0 0 0 0 0 0 0 0 0
0 0 0 0 0 0 0 0 0 0 0 0 0
0 0 0 0 0 1 0
"Viruses;Uroviricota;Caudoviricetes;Caudoviricetes__na;Zobellviridae;Veravirus;Veravirus__uc" 0 0
0 0 2 10 0 0 0 0 0 0 0 0 0
0 0 0 0 0 0 0 0 0 0 0 0 0
0 0 0 0 0 0 0 0 0 0 0 0 0
0 0 0 0 0 0 0 0 0 0 0 0 0
0 0 0 0 0 0 0 0 0 0 0 0 0
0 0 0 0 0
"Viruses;Uroviricota;Caudoviricetes;Caudoviricetes__na;Zobellviridae;Veravirus;Veravirus_septentrionalis"
0 0 1 0 18 18 0 0 0 0 0 0 0
0 0 0 0 0 0 0 0 0 0 0 0 0
0 0 0 1 0 0 0 0 0 0 0 0 0
0 0 0 0 0 0 0 0 1 0 0 0 0

0 0 0 0 0 0 0 0 0 0 0 0 0
0 0 0 0 0 0 0
"Viruses;Uroviricota;Caudoviricetes;Caudoviricetes__na;Zobellviridae;Zobellviridae__na;Erwinia_phage_Pecta"
0 0 0 1 0 0 0 0 0 0 0 0 0
0 0 0 0 0 0 0 1 0 0 0 0 0
0 0 0 0 0 0 0 0 1 0 0 0 0
0 0 0 0 0 0 0 0 0 0 0 0 0
0 0 0 0 0 0 0 0 0 0 0 0 0
0 0 0 0 0 0 0
"Viruses;Uroviricota;Caudoviricetes;Caudoviricetes__na;Zobellviridae;Zobellviridae__na;Podoviridae_sp._ctbj_
2" 0 0 2 3 15 5 0 0 0 0 0 0 0
0 0 0 0 0 0 0 0 0 0 0 0 0
0 0 0 0 0 0 0 0 0 0 0 0 0
0 0 0 0 0 0 0 0 2 0 0 0 0
0 0 0 0 0 0 0 0 0 0 0 0 0
0 0 0 0 0 0 0
"Viruses;Uroviricota;Caudoviricetes;Caudoviricetes__na;Zobellviridae;Zobellviridae__na;Pseudomonas_phage_
GP100" 1 0 0 1 6 3 0 0 0 0 0 0 0
0 0 0 0 0 0 0 0 0 0 1 0 0
0 0 0 0 0 0 0 0 0 0 0 0 0
0 0 17 0 0 0 0 0 0 0 0 0 0
0 0 0 0 0 0 0 0 0 0 0 0 0
0 0 0 0 0 0 0
"Viruses;Uroviricota;Caudoviricetes;Caudoviricetes__na;Zobellviridae;Zobellviridae__na;Vibrio_phage_172P1"
0 0 0 0 0 0 0 0 0 0 0 0 0
0 0 0 0 0 0 0 0 0 0 1 0 0
0 0 0 0 0 0 0 0 0 0 0 0 0
0 0 0 0 0 0 0 0 0 0 0 0 0
0 0 0 0 0 0 0 0 0 0 0 0 0
0 0 0 0 0 0 0
"Viruses;Uroviricota;Caudoviricetes;Caudoviricetes__na;Zobellviridae;Zobellviridae__na;Vibrio_phage_VP41s3"
0 0 0 0 9 1 0 0 0 0 0 0 0
0 0 0 0 0 0 0 0 0 0 0 0 0
0 0 0 0 0 0 0 0 0 0 0 0 0
0 0 0 0 0 0 0 0 0 0 0 0 0
0 0 0 0 0 0 0 0 0 0 0 0 0
0 0 0 0 0 0 0
"Viruses;Uroviricota;Caudoviricetes;Caudoviricetes__na;Zobellviridae;Zobellviridae__na;Vibrio_virus_VPMCC
5" 0 0 2 0 0 4 0 0 0 0 0 0 0
0 0 0 0 0 0 0 0 0 0 0 0 0
0 0 0 0 0 0 0 0 0 0 0 0 0
0 0 0 0 0 0 0 0 2 0 0 0 0
0 0 0 0 0 0 0 0 0 0 0 0 0
0 0 0 0 0 1 0
"Viruses;Uroviricota;Caudoviricetes;Caudoviricetes__na;Zobellviridae;Zobellviridae__uc;Zobellviridae__uc"
0 0 0 0 3 9 0 0 0 0 0 0 0
0 0 0 0 0 0 0 0 0 0 0 0 0
0 0 0 0 0 0 0 0 0 0 0 0 0
0 0 0 0 0 0 0 0 0 0 0 0 0

0 0 0 0 0 0 0 0 0 0 0 0 0
0 0 0 0 0 0 0
"Viruses;Uroviricota;Caudoviricetes;Caudoviricetes__uc;Caudoviricetes__uc;Caudoviricetes__uc;Caudoviricetes__uc" 10 15 68 89 377 313 0 12 5 16 632 6 631
596 1088 998 1887 4940 726 801 257 287 1478 144 723 604
905 156 672 11 419 463 40 13 6269 139 1813 1290 145
48 170 4302 554 695 913 169 946 141 465 272 293 10
334 4144 783 1140 65 40 24 255 1285 373 90 174 12
2 715 957 27 157 65 0
"Viruses;Uroviricota;Caudoviricetes;Crassvirales;Crassvirales__na;Crassvirales__na;CrAss-like_virus_sp."
0 1 3 4 120 21 0 0 0 0 0 0 284
262 1139 994 448 1520 58 36 40 45 9 2 13 23
51 5 22 0 73 38 0 0 3 3 439 170 54
68 214 1995 63 42 1652 268 31 14 116 49 35 1
196 16 417 694 5 3 0 94 274 63 16 39 2
5 60 63 1 5 9 0
"Viruses;Uroviricota;Caudoviricetes;Crassvirales;Crassvirales__na;Crassvirales__na;CrAss-like_virus_sp._ctOWe7" 0 0 0 0 0 0 0 0 0 0 0
0 0 0 0 0 0 0 3 6 2 3 0 0
0 0 0 0 0 0 0 0 0 0 0 0 0
0 0 0 0 0 0 0 0 0 0 0 0 0
0 0 0 0 0 0 0 0 0 0 0 0 0
0 0 0 0 0 0 0 0 0
"Viruses;Uroviricota;Caudoviricetes;Crassvirales;Crassvirales__na;Crassvirales__na;CrAss-like_virus_sp._ctRQZ5" 0 0 0 0 0 0 0 0 0 0 0
0 0 0 0 0 0 0 0 0 0 0 0 0
0 0 0 0 0 0 0 0 0 0 0 0 0
1 1 0 0 0 0 0 0 0 0 0 0 0
0 0 0 0 0 0 0 0 0 0 0 0 0
0 0 0 0 0 0 0 0 0
"Viruses;Uroviricota;Caudoviricetes;Crassvirales;Crassvirales__na;Crassvirales__na;CrAss-like_virus_sp._ctt4r3" 0 0 0 0 0 0 0 0 0 0 0
0 1 3 15 6 6 22 0 0 0 0 0 0
0 0 0 0 0 0 1 1 0 0 0 0 0
0 0 0 0 0 0 3 0 0 0 0 24 6
10 0 1 0 1 2 0 0 2 0 0 0 0
0 0 0 7 5 0 0 0 0
"Viruses;Uroviricota;Caudoviricetes;Crassvirales;Crassvirales__na;Crassvirales__na;CrAss-like_virus_sp._ctYsL76" 0 0 0 0 0 0 0 0 0 0 0
0 0 0 0 0 0 0 0 0 0 0 0 0
0 0 0 0 0 0 0 0 0 0 0 0 0
0 0 0 0 0 0 0 1 0 0 0 0 0
0 0 0 0 0 0 0 0 0 0 0 0 0
0 0 0 0 0 0 0 0 0
"Viruses;Uroviricota;Caudoviricetes;Crassvirales;Crassvirales__na;Crassvirales__na;Crassvirales_sp._ctvpJ74"
0 0 1 1 0 0 0 0 0 0 0 0 0
0 0 0 0 0 9 28 16 16 23 3 0 2
73 12 34 0 0 0 0 0 1 0 0 1 0
0 0 0 0 0 0 0 0 0 0 0 0 0

0 10 0 0 0 0 0 0 0 0 0 0 0
0 30 33 0 1 0 0
"Viruses;Uroviricota;Caudoviricetes;Crassvirales;Crassvirales__na;Crassvirales__na;uncultured_crAssphage"
0 0 0 0 0 0 0 0 0 0 0 0 0
0 0 0 0 0 0 0 2 1 0 0 0 0
0 0 0 0 0 0 0 0 0 0 0 0 0
0 0 0 0 0 0 0 0 0 0 0 0 0
0 0 0 0 0 0 0 0 0 0 0 0 0
0 3 2 0 0 0 0
"Viruses;Uroviricota;Caudoviricetes;Crassvirales;Crassvirales__uc;Crassvirales__uc;Crassvirales__uc" 1
1 2 0 19 2 0 0 0 0 0 0 47 63
432 347 76 96 3 22 25 17 4 1 2 15 101
4 36 0 0 0 0 0 0 0 11 34 11 1
11 18 0 1 79 14 0 0 49 15 14 0 7
2 6 7 0 0 1 0 0 0 0 0 0 0
20 17 0 0 2 0
"Viruses;Uroviricota;Caudoviricetes;Crassvirales;Crevaviridae;Junduvirus;Junduvirus__uc" 0 0
0 0 0 0 0 0 0 0 0 0 0 0 0
0 0 0 0 0 0 0 0 0 0 0 0 0
0 0 0 0 0 0 0 0 0 0 0 0 0
0 0 0 0 0 0 0 9 0 0 0 0 0
0 0 0 0 0 0 0 0 0 0 0 0 0
0 0 0 0 0
"Viruses;Uroviricota;Caudoviricetes;Crassvirales;Crevaviridae;Junduvirus;Junduvirus_communis" 0
0 0 0 0 0 0 0 0 0 0 0 0 0
0 0 0 0 0 0 0 0 0 0 0 0 0
0 0 0 0 0 0 0 0 0 1 1 1 0
0 0 0 0 0 1 0 0 105 33 27 0 2
0 0 0 0 0 3 0 0 0 0 0 0 0
0 0 0 0 0 0
"Viruses;Uroviricota;Caudoviricetes;Crassvirales;Crevaviridae;Junduvirus;Junduvirus_copri" 0 0
0 0 0 0 0 0 0 0 0 0 0 0 0
0 0 0 0 0 0 0 0 1 0 0 0 0
0 0 0 0 0 0 0 0 0 0 0 0 0
0 0 0 0 0 0 0 2 1 0 0 0 0
0 0 0 0 0 0 0 0 0 0 0 0 0
0 0 0 1 0
"Viruses;Uroviricota;Caudoviricetes;Crassvirales;Crevaviridae;Kingevirus;Kingevirus_communis" 0
0 0 0 0 0 0 0 0 0 0 0 2 0
13 5 1 9 2 1 2 0 0 0 0 0 0
0 0 0 0 0 0 0 0 0 0 0 0 0
0 0 0 0 0 0 0 0 42 9 14 0 0
0 1 5 0 0 4 0 0 0 0 0 0 0
0 0 0 0 0 0
"Viruses;Uroviricota;Caudoviricetes;Crassvirales;Intestiviridae;Carjivirus;Carjivirus_communis" 0 0
0 0 0 0 0 0 0 0 0 0 0 0 0
0 0 0 0 0 0 0 0 0 0 0 0 0
0 0 0 0 0 0 20 0 0 0 0 0 0
0 0 0 0 0 0 0 0 0 0 0 0 1

0 0 0 0 0 0 0 0 0 0 0 0 0
0 0 0 0 0
"Viruses;Uroviricota;Caudoviricetes;Crassvirales;Intestiviridae;Carjivirus;Carjivirus_hominis" 0 0
0 0 0 0 0 0 0 0 0 0 0 0 0
0 0 0 0 1 1 2 0 0 0 0 7 4
10 0 0 0 0 0 0 0 0 0 0 0 0
0 0 0 0 0 0 0 0 0 0 0 0 0
0 0 0 0 0 0 0 0 0 0 0 0 0
1 0 0 0 0
"Viruses;Uroviricota;Caudoviricetes;Crassvirales;Intestiviridae;Dabirmavirus;Dabirmavirus_hominis" 0
0 0 0 0 0 0 0 0 0 0 0 0 0
0 1 0 0 0 0 0 0 0 0 0 0 0
0 0 0 0 0 0 0 0 0 0 0 0 0
0 0 0 0 0 0 0 0 390 91 166 4 0
0 0 0 0 0 1 0 0 0 0 0 0 0
0 0 0 0 15 0
"Viruses;Uroviricota;Caudoviricetes;Crassvirales;Intestiviridae;Delmidovirus;Delmidovirus_copri" 0
0 0 0 0 0 0 0 0 0 0 0 1 0
0 0 0 0 0 0 0 0 0 0 0 0 0
0 0 0 0 0 0 0 0 0 0 0 0 0
0 0 0 0 0 0 0 0 0 0 0 0 0
0 0 0 0 0 0 0 0 0 0 0 0 0
0 0 0 0 0 0
"Viruses;Uroviricota;Caudoviricetes;Crassvirales;Intestiviridae;Diorhovirus;Diorhovirus_copri" 0 0
0 0 0 0 0 0 0 0 0 0 0 0 0
0 0 0 0 0 0 0 0 0 0 1 0 0
0 0 0 0 0 0 0 0 0 0 0 0 0
0 0 0 0 0 0 0 0 0 0 0 0 0
0 0 0 0 0 0 0 0 0 0 0 0 0
0 0 0 1 0
"Viruses;Uroviricota;Caudoviricetes;Crassvirales;Intestiviridae;Diorhovirus;Diorhovirus_intestinalis" 0
0 0 0 0 0 0 0 0 0 0 0 0 0
0 0 0 0 0 0 0 0 0 0 0 4 39
1 10 0 0 0 0 0 0 0 0 0 0 0
0 0 0 0 0 0 0 0 0 0 0 0 0
0 0 0 0 0 0 0 0 0 0 0 0 0
0 0 0 0 0 0
"Viruses;Uroviricota;Caudoviricetes;Crassvirales;Intestiviridae;Endlipuvirus;Endlipuvirus_intestinihominis"
0 0 0 0 0 0 0 0 0 0 0 0 0
0 0 0 0 0 0 0 0 0 0 0 0 0
0 0 0 0 0 0 0 0 0 0 0 1 0
0 0 0 0 0 0 0 0 0 0 0 0 0
0 0 0 0 0 0 0 0 0 0 0 0 0
0 0 0 0 0 0 0
"Viruses;Uroviricota;Caudoviricetes;Crassvirales;Intestiviridae;Hacihdavirus;Hacihdavirus_animalis" 0
0 0 1 0 0 0 0 0 0 0 0 0 0
0 0 0 0 0 0 0 0 0 0 0 0 0
0 0 0 0 0 0 0 0 0 0 0 0 0
0 0 0 0 0 0 0 0 0 0 0 0 0

0	0	0	0	0	0	0	0	0	0	0	0	0
0	0	0	0	0	0
"Viruses;Uroviricota;Caudoviricetes;Crassvirales;Intestiviridae;Intestiviridae__na;CrAssphage_LMMB"	0
0	0	0	0	0	0	0	0	0	0	0	0	0
0	0	0	0	1	12	3	5	0	0	0	4	24
4	18	0	0	0	0	0	0	0	0	0	0	0
0	0	0	0	0	0	0	0	0	0	0	0	0
5	0	0	0	0	0	0	0	0	0	0	0	0
16	19	0	0	0	0
"Viruses;Uroviricota;Caudoviricetes;Crassvirales;Intestiviridae;Intestiviridae__na;CrAssphage_sp._C0526BW15"
0	0	0	0	0	0	0	0	0	0	0	0	0
0	0	0	0	0	1	3	5	6	0	0	0	1
15	0	0	0	0	0	0	0	0	0	0	0	0
0	0	0	0	0	0	0	0	0	0	0	0	0
0	4	1	0	0	0	0	0	0	0	0	0	0
0	24	22	0	0	0	0
"Viruses;Uroviricota;Caudoviricetes;Crassvirales;Intestiviridae;Intestiviridae__na;CrAssphage_sp._C0531BW4"
0	0	0	0	0	0	0	0	0	0	0	0	0
0	0	0	0	0	0	0	0	0	0	0	0	0
2	0	0	0	0	0	0	0	0	0	0	0	0
0	0	0	0	0	0	0	0	0	0	0	0	0
0	0	0	0	0	0	0	0	0	0	0	0	0
0	0	0	0	0	0	0
"Viruses;Uroviricota;Caudoviricetes;Crassvirales;Intestiviridae;Intestiviridae__na;CrAssphage_ZA"	0
0	4	0	0	2	0	0	0	0	0	0	0	0
0	0	0	0	6	36	47	24	4	3	1	5	61
15	29	1	2	0	0	0	5	0	0	0	0	0
0	0	0	0	0	0	0	0	0	0	0	0	0
277	1	0	0	0	0	0	0	0	0	0	0	0
72	95	0	3	0	0
"Viruses;Uroviricota;Caudoviricetes;Crassvirales;Intestiviridae;Intestiviridae__uc;Intestiviridae__uc"	0
0	0	0	0	0	0	0	0	0	0	0	0	0
0	0	0	0	3	22	21	8	8	0	0	2	20
3	12	0	0	0	0	0	0	0	0	0	0	0
0	0	0	0	0	0	0	0	0	0	0	0	0
6	0	0	0	0	0	0	0	0	0	0	0	0
26	41	0	1	0	0
"Viruses;Uroviricota;Caudoviricetes;Crassvirales;Intestiviridae;Jahgtovirus;Jahgtovirus__uc"	0	0
0	0	0	0	0	0	0	0	0	0	0	0	0
0	0	0	0	0	1	0	0	0	0	0	0	0
0	0	0	0	0	0	0	0	0	0	0	0	0
0	0	0	0	0	0	0	0	0	0	0	2	0
0	0	0	0	0	0	0	0	0	0	0	0	0
0	0	0	0	0
"Viruses;Uroviricota;Caudoviricetes;Crassvirales;Intestiviridae;Jahgtovirus;Jahgtovirus_gastrointestinalis"
0	0	1	0	0	0	0	0	0	0	0	0	0
0	0	0	0	0	1	2	1	2	2	0	0	0
0	0	0	0	0	0	0	0	0	0	0	3	3
1	0	0	0	0	0	0	0	0	0	0	0	0

2 0 0 0 0 0 0 0 0 0 0 0 0
0 0 0 0 0 0 0
"Viruses;Uroviricota;Caudoviricetes;Crassvirales;Intestiviridae;Jahgtovirus;Jahgtovirus_intestinihominis" 0
0 0 0 0 0 0 0 0 0 0 0 0 0
0 0 0 0 1 1 3 0 0 0 0 0 0
0 0 0 0 0 0 0 0 0 0 0 0 0
1 0 0 0 0 0 0 0 0 0 0 0 0
0 0 0 0 0 0 0 0 0 0 0 0 1
0 0 0 0 0 0
"Viruses;Uroviricota;Caudoviricetes;Crassvirales;Intestiviridae;Jahgtovirus;Jahgtovirus_secundus" 0
0 0 0 0 0 0 0 0 0 0 0 0 0
0 0 0 0 0 2 2 0 1 0 0 0 0
0 0 0 0 0 0 0 0 0 0 0 0 0
0 0 0 0 0 0 0 0 0 0 0 0 0
0 0 0 0 0 0 0 0 0 0 0 0 0
0 0 0 0 0 0
"Viruses;Uroviricota;Caudoviricetes;Crassvirales;Intestiviridae;Whopevirus;Whopevirus_animalis" 0
0 0 0 0 0 0 0 0 0 0 0 0 0
6 1 0 0 0 0 0 0 0 0 0 0 0
0 0 0 0 0 0 0 0 0 0 0 0 0
0 0 0 0 0 0 0 0 0 0 0 0 0
0 0 0 0 0 0 0 0 0 0 0 0 0
0 0 0 0 0 0
"Viruses;Uroviricota;Caudoviricetes;Crassvirales;Intestiviridae;Wotdevirus;Wotdevirus_murinus" 0
0 0 0 0 0 0 0 0 0 0 0 0 0
0 0 0 0 0 0 0 0 0 0 0 0 0
0 0 0 0 0 0 0 0 0 0 0 0 0
0 0 0 0 0 0 0 0 0 0 0 0 0
0 0 0 0 0 0 0 0 0 0 0 0 0
0 1 0 0 0 0
"Viruses;Uroviricota;Caudoviricetes;Crassvirales;Steigviridae;Akihdevirus;Akihdevirus_balticus"0 0
0 0 4 0 0 0 0 0 0 0 0 0 0
0 0 0 0 0 0 0 0 0 0 0 0 0
0 0 0 0 0 0 0 0 0 0 0 0 0
0 0 0 0 0 0 0 0 0 0 0 0 0
0 0 0 0 0 0 0 0 0 0 0 0 0
0 0 0 0 0
"Viruses;Uroviricota;Caudoviricetes;Crassvirales;Steigviridae;Kahnovirus;Kahnovirus__uc" 0 0
0 0 0 0 0 0 0 0 0 0 0 0 0
0 0 0 0 0 0 0 0 0 0 0 0 0
0 0 0 0 0 0 0 0 0 1 0 0 0
0 0 0 0 0 0 0 0 0 0 0 1 0
0 0 0 0 0 0 0 0 0 0 0 0 0
0 0 0 0 0
"Viruses;Uroviricota;Caudoviricetes;Crassvirales;Steigviridae;Kahnovirus;Kahnovirus_copri" 0 0
0 0 0 0 0 0 0 0 0 0 0 0 0
0 0 0 0 0 0 0 0 0 0 0 0 0
0 0 0 0 0 0 0 0 1 1 0 0 1
0 0 0 11 2 0 0 0 0 0 0 0 0

0 0 0 0 0 0 0 0 0 0 0 0 0
0 0 0 0 0
"Viruses;Uroviricota;Caudoviricetes;Crassvirales;Steigviridae;Kahnovirus;Kahnovirus_oralis" 0 0
0 0 10 0 0 0 0 0 0 0 0 0 0
0 0 0 0 0 0 0 0 0 0 0 0 0
0 0 0 0 0 0 0 0 8 3 0 0 0
0 0 0 0 0 0 0 0 0 0 0 1 0
0 0 0 0 0 0 0 0 0 0 0 0 0
0 0 0 0 0
"Viruses;Uroviricota;Caudoviricetes;Crassvirales;Steigviridae;Kehishuvirus;Kehishuvirus_primarius" 0
0 0 0 22 0 0 0 0 0 0 0 0 0
0 0 0 0 0 0 0 0 0 0 0 0 0
0 0 0 0 0 0 0 0 0 0 0 0 0
0 0 0 0 0 0 0 0 0 0 0 0 0
0 0 0 0 0 0 0 0 0 0 0 0 0
0 0 0 0 0 0
"Viruses;Uroviricota;Caudoviricetes;Crassvirales;Steigviridae;Kehishuvirus;Kehishuvirus_splanchnicus" 0
0 0 0 0 0 0 0 0 0 0 0 0 0
0 2 0 0 0 0 0 0 0 0 0 0 0
0 0 0 0 0 0 0 0 0 0 0 0 0
0 0 0 0 0 0 0 0 0 0 0 0 0
0 0 0 0 0 0 0 0 0 0 0 0 0
0 0 0 0 0 0
"Viruses;Uroviricota;Caudoviricetes;Crassvirales;Steigviridae;Kolpuevirus;Kolpuevirus_coli" 0 0
0 0 0 0 0 0 0 0 0 0 0 0 1
0 1 10 0 0 0 0 0 0 0 0 0 0
0 0 0 0 0 0 0 0 0 0 0 0 0
0 0 0 0 0 0 0 0 0 0 0 0 0
0 0 0 0 0 0 0 0 0 0 0 0 0
0 0 0 0 0
"Viruses;Uroviricota;Caudoviricetes;Crassvirales;Steigviridae;Kolpuevirus;Kolpuevirus_hominis"0 0
0 0 2 0 0 0 0 0 0 0 0 0 0
0 0 0 0 0 0 0 0 0 0 0 0 0
0 0 0 0 0 0 0 0 0 0 0 0 0
0 0 0 0 0 0 0 0 0 0 0 0 0
0 0 0 0 0 0 0 0 0 0 0 0 0
0 0 0 0 0
"Viruses;Uroviricota;Caudoviricetes;Crassvirales;Steigviridae;Lahndsivirus;Lahndsivirus_rarus" 0 0
0 0 0 0 0 0 0 0 0 0 0 0 2
0 0 1 0 0 0 0 0 0 0 0 0 0
0 0 0 0 0 0 0 0 0 0 0 0 0
0 0 0 5 2 0 0 0 0 0 0 0 0
0 0 0 0 0 0 0 0 0 0 0 0 0
0 0 0 0 0
"Viruses;Uroviricota;Caudoviricetes;Crassvirales;Steigviridae;Lebriduvirus;Lebriduvirus_gastrointestinalis"
0 0 0 0 0 0 0 0 0 0 0 0 0
0 0 0 0 0 0 0 0 0 0 0 0 0
5 0 3 0 0 0 0 0 0 0 0 0 0
0 0 0 0 0 0 0 0 0 0 0 0 0

0 0 0 0 0 0 0 0 0 0 0 0 0
0 0 0 0 0 0 0
"Viruses;Uroviricota;Caudoviricetes;Crassvirales;Steigviridae;Mahstovirus;Mahstovirus_faecalis" 0 0
0 1 0 0 0 0 0 0 0 0 0 0 2
1 1 0 0 0 0 0 0 0 0 0 0 0
0 0 0 0 0 0 0 0 0 0 0 0 0
0 0 0 0 0 0 1 0 0 0 0 0 0
0 0 0 0 0 0 0 0 0 0 0 0 0
0 0 0 0 0
"Viruses;Uroviricota;Caudoviricetes;Crassvirales;Suoliviridae;Afonbuvirus;Afonbuvirus__uc" 0 0
0 0 0 0 0 0 0 0 0 0 0 0 0
0 0 0 0 0 0 0 0 0 0 0 0 0
0 0 0 1 1 0 0 0 0 0 0 0 0
0 0 0 0 0 0 0 0 0 0 0 0 0
0 0 0 0 0 0 0 0 0 0 0 0 0
0 0 0 0 0
"Viruses;Uroviricota;Caudoviricetes;Crassvirales;Suoliviridae;Afonbuvirus;Afonbuvirus_faecalis" 0
0 0 0 0 0 0 0 0 0 0 0 0 0
0 0 0 0 0 0 0 0 0 0 0 0 0
0 0 0 1 0 0 0 0 0 0 0 0 0
0 0 0 0 0 0 0 0 0 0 0 0 0
0 0 0 0 0 0 0 0 0 0 0 0 0
0 0 0 0 0 0
"Viruses;Uroviricota;Caudoviricetes;Crassvirales;Suoliviridae;Aurodevirus;Aurodevirus__uc" 0 0
0 0 0 0 0 0 0 0 0 0 0 0 0
0 2 0 0 0 0 0 0 0 0 0 0 0
0 0 0 0 0 0 0 0 0 0 0 0 0
0 0 0 0 0 0 0 0 0 0 0 0 0
0 0 0 0 0 0 0 0 0 0 0 0 0
0 0 0 0 0
"Viruses;Uroviricota;Caudoviricetes;Crassvirales;Suoliviridae;Aurodevirus;Aurodevirus_hiberniae" 0
0 0 1 0 0 0 0 0 0 0 0 0 0
0 0 0 0 0 0 0 0 0 0 0 0 0
0 0 0 0 0 0 0 0 0 0 0 0 0
0 0 0 0 0 0 0 0 0 0 0 0 0
0 0 0 0 0 0 0 0 0 0 0 0 0
0 0 0 0 0 0
"Viruses;Uroviricota;Caudoviricetes;Crassvirales;Suoliviridae;Aurodevirus;Aurodevirus_intestinalis" 0
0 0 0 0 0 0 0 0 0 0 0 0 0
0 0 0 0 1 0 0 0 0 0 0 0 0
0 0 0 0 0 0 0 0 0 0 0 0 0
0 0 0 0 0 0 0 0 0 0 0 0 0
0 0 0 0 0 0 0 0 0 0 0 0 0
0 0 0 0 0 0
"Viruses;Uroviricota;Caudoviricetes;Crassvirales;Suoliviridae;Birpovirus;Birpovirus__uc"0 0 0
0 0 0 0 0 0 0 0 0 0 0 0 0
0 0 0 0 0 0 0 0 0 0 0 0 0
0 0 0 0 0 0 0 0 0 0 0 0 7
0 0 0 0 0 0 0 0 0 0 0 0 0

0 0 0 0 0 0 0 0 0 0 0 0 0
0 0 0 0
"Viruses;Uroviricota;Caudoviricetes;Crassvirales;Suoliviridae;Birpovirus;Birpovirus_hiberniae" 0 0
0 0 0 0 0 0 0 0 0 0 0 0 0
0 0 0 0 0 0 0 0 0 0 0 0 0
0 0 0 0 0 0 0 0 1 3 0 0 0
2 0 0 0 0 0 0 0 0 0 0 0 0
0 0 0 0 0 0 0 0 0 0 0 0 0
0 0 0 0 0
"Viruses;Uroviricota;Caudoviricetes;Crassvirales;Suoliviridae;Birpovirus;Birpovirus_hominis" 0 0
0 0 0 0 0 0 0 0 0 0 0 0 0
0 0 0 0 0 0 0 0 0 0 0 0 0
0 0 0 0 0 0 0 0 1 4 1 0 0
0 0 0 0 0 0 0 0 0 0 0 3 0
0 0 0 0 0 0 0 0 0 0 0 0 0
0 0 0 0 0
"Viruses;Uroviricota;Caudoviricetes;Crassvirales;Suoliviridae;Blohavirus;Blohavirus__uc" 0 0
0 0 0 0 0 0 0 0 0 0 0 0 0
0 0 0 0 0 0 0 0 0 0 0 0 0
0 0 0 0 0 0 0 0 3 4 2 0 0
1 0 0 0 0 0 0 0 0 0 0 4 0
0 0 0 0 0 0 0 0 0 0 0 0 0
0 0 0 0 0
"Viruses;Uroviricota;Caudoviricetes;Crassvirales;Suoliviridae;Blohavirus;Blohavirus_americanus" 0
0 0 0 0 0 0 0 0 0 0 0 0 0
0 0 0 0 0 0 0 0 0 0 0 0 0
0 0 0 0 0 0 0 0 0 0 3 0 0
0 12 0 0 0 0 0 0 0 0 0 0 3
0 0 0 0 0 0 0 0 0 0 0 0 0
0 0 0 0 0 0
"Viruses;Uroviricota;Caudoviricetes;Crassvirales;Suoliviridae;Blohavirus;Blohavirus_faecalis" 0 0
0 0 0 0 0 0 0 0 0 0 0 0 0
0 0 0 0 0 0 0 0 0 0 0 0 0
0 0 0 0 0 0 0 0 3 11 5 2 0
0 0 0 0 0 0 0 0 0 0 0 9 0
0 0 0 0 0 0 0 0 0 0 0 0 0
0 0 0 0 0
"Viruses;Uroviricota;Caudoviricetes;Crassvirales;Suoliviridae;Buchavirus;Buchavirus__uc" 0 0
0 0 0 0 0 0 0 0 0 0 4 6 106
93 52 122 0 0 0 0 0 0 0 0 0 0
0 0 0 0 0 0 0 0 0 0 0 0 0
0 0 0 0 0 0 0 0 0 0 0 0 0
1 0 0 0 0 0 0 0 0 0 0 0 0
0 0 0 0 0
"Viruses;Uroviricota;Caudoviricetes;Crassvirales;Suoliviridae;Buchavirus;Buchavirus_coli" 0 0
0 0 0 0 0 0 0 0 0 0 0 1 7
9 6 4 0 0 0 0 0 0 0 0 0 0
0 0 0 0 0 0 0 0 0 0 0 0 4
0 0 0 9 0 0 0 0 0 0 0 0 0

0 0 0 0 0 0 0 0 0 0 0 0 0
0 0 0 0 0
"Viruses;Uroviricota;Caudoviricetes;Crassvirales;Suoliviridae;Buchavirus;Buchavirus_copri" 0 0
0 0 0 0 0 0 0 0 0 0 29 28 451
416 522 1184 0 0 0 0 10 0 0 0 0 0
0 0 0 0 0 0 0 0 0 0 0 0 0
0 0 0 0 0 0 0 0 0 1 1 0 0
1 0 0 0 1 0 0 0 0 0 0 0 0
0 0 0 12 0
"Viruses;Uroviricota;Caudoviricetes;Crassvirales;Suoliviridae;Buchavirus;Buchavirus_faecalis" 0 0
0 0 0 0 0 0 0 0 0 0 0 3 20
13 18 12 0 0 0 0 0 0 0 0 0 0
0 0 0 0 0 0 0 0 0 0 0 0 0
0 0 0 0 0 0 0 0 0 0 0 0 0
0 1 0 0 0 0 0 0 0 0 0 0 0
0 0 0 0 0
"Viruses;Uroviricota;Caudoviricetes;Crassvirales;Suoliviridae;Buchavirus;Buchavirus_hiberniae" 0 0
0 0 0 0 0 0 0 0 0 0 0 0 6
4 1 0 0 0 0 0 0 0 0 0 0 0
0 0 0 0 0 0 0 0 0 0 0 0 0
0 0 0 0 0 0 0 0 0 0 0 0 0
0 0 0 0 0 0 0 0 0 0 0 0 0
0 0 0 0 0
"Viruses;Uroviricota;Caudoviricetes;Crassvirales;Suoliviridae;Buchavirus;Buchavirus_hominis" 0 0
0 0 0 0 0 0 0 0 0 0 0 1 4
4 0 1 0 0 0 0 0 0 0 0 0 0
0 0 0 0 0 0 0 0 0 0 0 0 0
0 0 0 0 0 0 0 0 0 0 0 0 0
0 0 0 0 0 0 0 0 0 0 0 0 0
0 0 0 0 0
"Viruses;Uroviricota;Caudoviricetes;Crassvirales;Suoliviridae;Buchavirus;Buchavirus_intestinalis" 0
0 0 0 0 0 0 0 0 0 0 0 0 0
3 0 0 2 0 0 0 0 0 0 0 0 0
0 0 0 0 0 0 0 0 0 0 0 0 0
0 0 0 0 0 0 0 0 0 0 0 0 0
0 0 3 0 0 0 0 0 0 0 0 0 0
0 0 0 0 0 0
"Viruses;Uroviricota;Caudoviricetes;Crassvirales;Suoliviridae;Buchavirus;Buchavirus_oralis" 0 0
0 0 0 0 0 0 0 0 0 0 0 0 0
0 0 0 0 2 9 4 1 0 0 0 0 0
0 0 0 0 0 0 0 0 0 0 0 0 0
0 0 0 1 0 0 0 0 0 0 0 0 0
0 0 0 0 0 0 0 0 0 0 0 0 0
0 0 0 0 0
"Viruses;Uroviricota;Caudoviricetes;Crassvirales;Suoliviridae;Buchavirus;Buchavirus_splanchnicus" 0
0 0 0 0 0 0 0 0 0 0 0 0 0
0 0 0 1 0 0 0 0 0 0 0 0 0
0 0 0 0 0 0 0 0 0 0 0 0 0
0 0 0 0 0 0 0 0 0 0 0 0 0

0 0 0 0 0 0 0 0 0 0 0 0 0
0 0 0 0 0 0
"Viruses;Uroviricota;Caudoviricetes;Crassvirales;Suoliviridae;Buorbuivirus;Buorbuivirus_hominis" 0
0 0 0 0 0 0 0 0 0 0 0 0 0
0 0 0 0 0 0 0 0 0 0 0 0 0
0 0 0 0 0 0 0 0 0 0 1 0 0
0 0 0 0 0 0 0 0 0 0 0 0 0
0 0 0 0 0 0 0 0 0 0 0 0 0
0 0 0 0 0 0
"Viruses;Uroviricota;Caudoviricetes;Crassvirales;Suoliviridae;Burzaovirus;Burzaovirus__uc" 0 0
0 0 0 0 0 0 0 0 0 0 0 0 0
0 0 0 0 0 0 0 0 0 0 0 0 0
0 0 0 0 0 0 0 0 0 0 0 0 0
0 0 1 8 0 0 0 0 0 0 0 0 0
0 0 0 0 0 0 0 0 0 0 0 0 0
0 0 0 0 0
"Viruses;Uroviricota;Caudoviricetes;Crassvirales;Suoliviridae;Cacepaovirus;Cacepaovirus_simiae" 0
0 0 0 0 0 0 0 0 0 0 0 0 0
0 0 0 0 0 0 0 0 0 0 0 0 0
0 0 0 0 0 0 0 0 0 0 0 0 0
0 0 0 0 0 0 0 0 0 0 0 0 0
0 0 1 0 0 0 0 0 0 0 0 0 0
0 0 0 0 0 0
"Viruses;Uroviricota;Caudoviricetes;Crassvirales;Suoliviridae;Canhaevirus;Canhaevirus_faecalis"0 0
0 0 0 0 0 0 0 0 0 0 0 0 8
4 0 1 0 0 0 0 0 0 0 0 0 0
0 0 0 0 0 0 16 0 0 0 0 0 0
0 0 0 0 0 0 0 0 0 0 0 0 9
0 0 0 0 0 0 0 0 0 0 0 0 13
8 1 0 0 0
"Viruses;Uroviricota;Caudoviricetes;Crassvirales;Suoliviridae;Canhaevirus;Canhaevirus_hiberniae" 0
0 0 0 0 0 0 0 0 0 0 0 0 0
0 0 0 0 0 0 0 0 0 0 0 0 0
0 0 0 0 0 0 0 0 0 0 0 0 0
0 0 0 0 0 0 0 0 13 3 10 0 0
0 1 0 0 0 0 0 0 0 0 0 0 0
0 0 0 0 0 0
"Viruses;Uroviricota;Caudoviricetes;Crassvirales;Suoliviridae;Cohcovirus;Cohcovirus__uc" 0 0
0 0 0 0 0 0 0 0 0 0 0 0 0
0 0 0 0 0 0 0 0 0 0 0 0 0
0 0 0 0 0 0 0 0 0 0 0 0 0
0 0 0 0 0 0 0 4 0 0 0 0 0
0 0 0 0 0 0 0 0 0 0 0 0 0
0 0 0 0 0
"Viruses;Uroviricota;Caudoviricetes;Crassvirales;Suoliviridae;Cohcovirus;Cohcovirus_hiberniae" 0 0
0 0 0 0 0 0 0 0 0 0 0 0 0
0 0 0 0 0 0 0 0 0 0 0 0 0
0 0 0 0 0 0 0 0 0 0 0 0 0
0 0 0 0 0 0 0 14 7 2 0 0 0

0 0 0 0 0 0 0 0 0 0 0 0 0
0 0 0 0 0
"Viruses;Uroviricota;Caudoviricetes;Crassvirales;Suoliviridae;Cohcovirus;Cohcovirus_splanchnicus" 0
0 0 0 0 0 0 0 0 0 0 0 0 0
0 0 0 0 0 0 0 0 0 0 0 0 0
0 0 0 0 0 0 0 0 0 0 0 0 0
0 0 0 0 0 0 0 0 57 16 31 0 0
0 0 1 0 0 1 0 0 0 0 0 0 0
0 0 0 0 3 0
"Viruses;Uroviricota;Caudoviricetes;Crassvirales;Suoliviridae;Culoivirus;Culoivirus__uc"0 0 0
0 0 0 0 0 0 0 0 0 0 0 1 1
2 0 0 0 0 0 0 0 0 0 0 0 0
0 0 0 0 0 0 0 0 0 0 0 0 0
0 0 0 0 0 0 1 0 1 0 0 0 0
0 0 0 0 0 0 0 0 0 0 0 0 0
0 0 1 0
"Viruses;Uroviricota;Caudoviricetes;Crassvirales;Suoliviridae;Culoivirus;Culoivirus_americanus"0 0
0 0 0 0 0 0 0 0 0 0 0 0 0
0 0 0 0 0 0 0 0 0 0 0 0 0
0 0 0 0 0 0 0 0 0 0 0 0 0
0 0 0 0 0 0 0 0 0 1 0 0 0
0 0 0 0 0 0 0 0 0 0 0 0 1
1 0 0 0 0
"Viruses;Uroviricota;Caudoviricetes;Crassvirales;Suoliviridae;Culoivirus;Culoivirus_faecalis" 0 0
0 0 0 0 0 0 0 0 0 0 0 0 0
0 0 10 0 0 0 0 0 0 0 0 0 0
0 0 0 0 0 0 0 0 0 0 0 0 0
0 0 0 0 0 0 0 0 0 0 0 0 1
0 0 0 0 0 0 0 1 0 0 0 0 1
3 0 0 0 0
"Viruses;Uroviricota;Caudoviricetes;Crassvirales;Suoliviridae;Culoivirus;Culoivirus_intestinalis" 0 0
0 0 0 0 0 0 0 0 0 0 0 0 0
0 0 0 0 0 0 0 0 0 0 0 0 0
0 0 0 0 0 0 0 0 0 0 0 0 0
0 0 0 0 0 0 0 0 0 1 0 0 0
0 0 0 0 0 0 0 0 0 0 0 0 0
0 0 0 0 0
"Viruses;Uroviricota;Caudoviricetes;Crassvirales;Suoliviridae;Suoliviridae__uc;Suoliviridae__uc" 0 0
0 0 0 0 0 0 0 0 0 0 0 0 1
0 2 0 0 0 0 0 0 0 0 0 0 0
0 0 0 0 0 0 0 0 4 9 8 0 0
16 0 0 0 0 0 0 0 0 1 0 2 0
0 0 0 0 0 0 0 0 0 0 0 0 0
0 0 0 0 0
"Viruses;Uroviricota;Caudoviricetes;Kirjokansivirales;Haloferuviridae;Haloferuviridae__na;environmental_Hal
ophage_eHP-29" 0 0 0 1 0 0 0 0 0 0 0
0 0 0 0 0 0 0 0 0 0 0 0 0
0 0 0 0 0 0 0 0 0 0 0 0 0
0 0 0 0 0 0 0 0 0 0 0 0 0

0 0 0 0 0 0 0 0 0 0 0 0 0
0 0 0 0 0 0 0 0 0
"Viruses;Uroviricota;Caudoviricetes;Methanobavirales;Anaerodiviridae;Metforvirus;Metforvirus_Drs3" 0
0 0 1 0 0 0 0 0 0 0 0 0 0
0 0 0 0 0 0 0 0 0 0 0 0 0
0 0 0 0 0 0 0 0 0 0 0 0 0
0 0 0 0 0 0 0 0 0 0 0 0 0
0 0 0 0 0 0 0 0 0 0 0 0 0
0 0 0 0 0 0
"Viruses;Viruses__na;Naldaviricetes;Lefavirales;Baculoviridae;Alphabaculovirus;Autographa_californica_multi
ple_nucleopolyhedrovirus" 0 0 0 0 0 0 0 0 0 0
0 0 0 0 0 0 0 0 0 0 0 0 0
0 0 0 0 0 0 0 0 0 0 0 0 0
0 0 0 0 0 0 0 0 0 0 0 0 0
0 0 0 0 0 0 0 0 0 0 0 0 0
0 0 0 0 0 0 0 0 1 0
"Viruses;Viruses__na;Naldaviricetes;Lefavirales;Baculoviridae;Alphabaculovirus;Orgyia_leucostigma_nucleopol
yhedrovirus" 0 0 0 1 0 0 0 0 0 0 0 0
0 0 0 0 0 0 0 0 0 0 0 0 0
0 0 0 0 0 0 0 0 0 0 0 0 0
0 0 0 0 0 0 0 0 0 0 0 0 0
0 0 0 0 0 0 0 0 0 0 0 0 0
0 0 0 0 0 0 0 0
"Viruses;Viruses__na;Naldaviricetes;Naldaviricetes__na;Nimaviridae;Nimaviridae__na;Hemigrapsus_takanoi_n
imavirus" 0 0 0 0 0 0 0 0 0 0 0 0
0 0 0 0 0 0 0 0 0 0 0 0 0
0 0 0 0 0 0 0 0 0 0 1 0 0
0 0 0 0 0 0 0 0 0 0 0 0 0
0 0 0 0 0 0 0 0 0 0 0 0 0
0 0 0 0 0 0 0 0
"Viruses;Viruses__na;Viruses__na;Viruses__na;Anelloviridae;Alphatorquevirus;Alphatorquevirus_sp." 0
0 0 0 0 0 0 0 0 0 0 0 0 0
0 0 0 0 0 0 0 0 0 0 0 0 0
0 0 0 0 0 0 0 0 0 0 0 0 0
0 0 0 0 0 0 1 0 0 0 0 0 0
0 0 0 0 0 0 0 0 0 0 0 0 0
0 0 0 0 0 0
"Viruses;Viruses__na;Viruses__na;Viruses__na;Anelloviridae;Alphatorquevirus;Torque_teno_virus_13" 1
0 0 0 0 0 0 0 0 0 0 0 0 0
0 0 0 0 0 0 0 0 0 0 0 0 0
0 0 0 0 0 0 0 0 0 0 0 0 0
0 0 0 0 0 0 0 0 0 0 0 0 0
0 0 0 0 0 0 0 0 0 0 0 0 0
0 0 0 0 0 0
"Viruses;Viruses__na;Viruses__na;Viruses__na;Anelloviridae;Alphatorquevirus;Torque_teno_virus_16" 0
1 0 0 0 0 0 0 0 0 0 0 0 0
0 0 0 0 0 0 0 0 0 0 0 0 0
0 0 0 0 0 0 0 0 0 0 0 0 0
0 0 0 0 0 0 0 0 0 0 0 0 0

0 0 0 0 0 0 0 0 0 0 0 0 0
0 0 0 0 0 0
"Viruses;Viruses__na;Viruses__na;Viruses__na;Anelloviridae;Alphatorquevirus;Torque_teno_virus_24" 3
1 0 0 0 0 0 0 0 0 0 0 0 0
0 0 0 0 0 0 0 0 0 0 0 0 0
0 0 0 0 0 0 0 0 0 0 0 0 0
0 0 0 0 0 0 7 0 0 0 0 0 0
0 0 0 0 0 0 0 0 0 0 0 1 0
0 0 0 0 0 0
"Viruses;Viruses__na;Viruses__na;Viruses__na;Anelloviridae;Alphatorquevirus;Torque_teno_virus_3" 0
0 0 0 0 0 0 0 0 0 0 0 0 0
0 0 0 0 0 0 0 0 0 0 0 0 0
0 0 0 0 0 0 0 0 0 0 1 0 0
0 0 0 0 0 0 0 0 0 0 0 0 0
0 0 0 0 0 0 0 0 0 0 0 0 0
0 0 0 0 0 0
"Viruses;Viruses__na;Viruses__na;Viruses__na;Anelloviridae;Anelloviridae__na;Anelloviridae_sp." 14
6 0 0 0 0 0 0 4 0 2 0 0 0
0 0 0 0 0 0 0 0 0 0 0 1 0
0 0 0 0 0 0 0 0 1 0 1 0 0
0 0 0 0 6 5 325 0 0 0 0 0 0
0 0 0 0 0 0 0 0 0 0 0 0 0
0 0 1 0 0 0
"Viruses;Viruses__na;Viruses__na;Viruses__na;Anelloviridae;Anelloviridae__na;Human_anellovirus_zj-ad_1"
0 0 0 0 0 0 0 0 0 0 0 0 0
0 0 0 0 0 0 0 0 0 0 0 0 0
0 0 0 0 0 0 0 0 0 0 0 0 0
0 0 0 0 0 0 0 39 0 0 0 0 0
0 0 0 0 0 0 0 0 0 0 0 0 0
0 0 0 0 0 0 0
"Viruses;Viruses__na;Viruses__na;Viruses__na;Anelloviridae;Anelloviridae__na;Torque_teno_midi_virus"
0 0 0 0 0 0 0 0 1 0 1 0 0
0 0 0 0 0 0 0 0 0 0 0 0 0
0 0 0 0 0 0 0 0 0 0 0 0 0
0 0 0 0 0 0 0 2 0 0 0 0 0
0 0 0 0 0 0 0 0 0 0 0 0 0
0 0 0 0 0 0 0
"Viruses;Viruses__na;Viruses__na;Viruses__na;Anelloviridae;Anelloviridae__na;Torque_teno_virus" 19
11 0 0 0 0 0 0 3 1 0 0 1 0
0 0 0 0 0 0 0 0 1 0 0 0 0
0 0 0 0 0 0 0 0 3 0 1 0 0
0 15 0 0 6 6 428 0 0 0 0 0 0
0 0 0 0 0 0 0 0 0 0 1 0 0
0 0 1 0 0 0
"Viruses;Viruses__na;Viruses__na;Viruses__na;Anelloviridae;Anelloviridae__uc;Anelloviridae__uc" 30
30 0 0 0 0 0 0 4 2 0 0 0 1
0 0 0 0 0 0 0 0 0 0 0 0 0
0 1 1 0 0 1 0 4 10 0 3 0 0
0 2 0 0 12 19 608 0 0 0 0 0 1

1 0 0 0 0 0 0 0 0 0 0 0 0
0 0 0 0 0 0
"Viruses;Viruses__na;Viruses__na;Viruses__na;Anelloviridae;Betatorquevirus;TTV-like_mini_virus" 3
4 0 0 0 0 0 0 2 0 0 0 0 0
0 0 0 0 0 0 0 0 0 0 0 0 0
0 1 0 0 0 0 0 0 1 0 1 0 0
0 5 0 0 0 6 56 0 0 0 0 0 0
1 0 0 0 0 0 0 0 0 0 0 0 0
0 0 0 0 0 0
"Viruses;Viruses__na;Viruses__na;Viruses__na;Anelloviridae;Gammatorquevirus;Gammatorquevirus_sp."
0 0 0 0 0 0 0 0 0 0 0 0 0
0 0 0 0 0 0 0 0 0 0 0 0 0
0 0 0 0 0 0 0 0 0 0 0 0 0
0 0 0 0 0 0 1 0 0 0 0 0 0
0 0 0 0 0 0 0 0 0 0 0 0 0
0 0 0 0 0 0 0
"Viruses;Viruses__na;Viruses__na;Viruses__na;Cruciviridae;Cruciviridae__na;Crucivirus_sp." 0 0
0 8 4 3 0 0 0 0 0 0 0 0 0
0 0 0 0 0 0 0 0 0 0 0 0 0
0 0 0 0 0 0 0 0 0 0 0 0 0
0 0 0 0 0 0 3 0 0 0 0 0 0
0 0 0 0 0 0 0 0 0 0 0 0 0
0 0 0 1 0
"Viruses;Viruses__na;Viruses__na;Viruses__na;Cruciviridae;Cruciviridae__na;Crucivirus-184" 0 0
0 0 0 2 0 0 0 0 0 0 0 0 0
0 0 0 0 0 0 0 0 0 0 0 0 0
0 0 0 0 0 0 0 0 0 0 0 0 0
0 0 0 0 0 0 0 0 0 0 0 0 0
0 0 0 0 0 0 0 0 0 0 0 0 0
0 0 0 0 0
"Viruses;Viruses__na;Viruses__na;Viruses__na;Cruciviridae;Cruciviridae__na;Crucivirus-380" 0 0
0 0 0 1 0 0 0 0 0 0 0 0 0
0 0 0 0 0 0 0 0 2 0 0 0 0
0 0 0 0 0 0 0 0 0 0 0 0 0
0 0 0 0 0 0 2 0 0 0 0 0 0
0 0 0 0 0 0 0 0 0 0 0 0 0
0 0 0 3 0
"Viruses;Viruses__na;Viruses__na;Viruses__na;Cruciviridae;Cruciviridae__na;Crucivirus-387" 0 0
0 1 0 5 0 0 0 0 0 0 0 0 0
0 0 0 0 0 0 0 0 0 0 0 0 0
0 0 0 0 0 0 0 0 0 0 0 0 0
0 0 0 0 0 0 8 0 0 0 0 0 0
0 0 0 0 0 0 0 0 0 0 0 0 0
0 0 0 2 0
"Viruses;Viruses__na;Viruses__na;Viruses__na;Cruciviridae;Cruciviridae__na;Crucivirus-389" 0 0
0 0 0 2 0 0 0 0 0 0 0 0 0
0 0 0 0 0 0 0 0 0 0 0 0 0
0 0 0 0 0 0 0 0 0 0 0 0 0
0 0 0 0 0 0 0 0 0 0 0 0 0

0 0 0 0 0 0 0 0 0 0 0 0 0
0 0 0 0 0
"Viruses;Viruses__na;Viruses__na;Viruses__na;Cruciviridae;Cruciviridae__na;Crucivirus-427" 0 0
1 1 0 3 0 0 0 0 0 0 0 0 0
0 0 0 0 0 0 0 0 0 0 0 0 0
0 0 0 0 0 0 0 0 0 0 0 0 0
0 0 0 0 0 0 0 0 0 0 0 0 0
0 0 0 0 0 0 0 0 0 0 0 0 0
0 0 0 0 0
"Viruses;Viruses__na;Viruses__na;Viruses__na;Cruciviridae;Cruciviridae__na;Crucivirus-432" 0 0
0 0 0 0 0 0 0 0 0 0 0 0 0
0 0 0 0 0 0 0 0 1 0 0 0 0
0 0 0 0 0 0 0 0 0 0 0 0 0
0 0 0 0 0 0 0 0 0 0 0 0 0
0 0 0 0 0 0 0 0 0 0 0 0 0
0 0 0 0 0
"Viruses;Viruses__na;Viruses__na;Viruses__na;Cruciviridae;Cruciviridae__na;Crucivirus-468" 0 0
6 30 331 131 0 0 0 0 0 0 0 0 0
0 1 13 0 0 0 0 1 2 0 0 0 0
0 1 0 0 0 0 2 0 0 0 0 0 0
5 0 0 0 0 0 6 0 0 0 0 1 0
0 0 0 0 0 0 0 0 0 0 0 0 0
0 0 0 2 0
"Viruses;Viruses__na;Viruses__na;Viruses__na;Cruciviridae;Cruciviridae__na;Crucivirus-479" 0 0
3 11 101 25 0 0 0 0 0 0 0 0 0
0 0 0 0 0 0 0 0 0 0 0 0 0
0 0 0 0 0 0 0 0 0 0 0 0 0
0 0 0 0 0 0 1 0 0 0 0 0 0
0 0 0 0 0 0 0 0 0 0 0 0 0
0 0 0 0 0
"Viruses;Viruses__na;Viruses__na;Viruses__na;Cruciviridae;Cruciviridae__na;Crucivirus-93" 0 0
0 1 0 0 0 0 0 0 0 0 0 0 0
0 0 0 0 0 0 0 0 0 0 0 0 0
0 0 0 0 0 0 0 0 0 0 0 0 0
0 0 0 0 0 0 0 0 0 0 0 0 0
0 0 0 0 0 0 0 0 0 0 0 0 0
0 0 0 0 0
"Viruses;Viruses__na;Viruses__na;Viruses__na;Cruciviridae;Cruciviridae__na;Crucivirus-
like_circular_genetic_element-85" 0 0 6 14 58 10 0 0 0
0 0 0 0 0 0 0 0 0 0 0 0 0
0 0 0 0 0 0 0 0 0 0 0 0 1
0 0 0 0 0 0 0 0 0 0 0 0 25
0 0 0 0 0 1 0 0 0 0 0 0 0
0 0 0 0 0 0 0 0 0 0 0
"Viruses;Viruses__na;Viruses__na;Viruses__na;Cruciviridae;Cruciviridae__uc;Cruciviridae__uc" 0 0
2 2 10 0 0 0 0 0 0 0 0 0 0
0 0 0 0 0 0 0 0 0 0 0 0 0
0 0 0 0 0 0 0 0 0 0 0 0 0
0 0 0 0 0 0 4 0 0 0 0 0 0

0 0 0 0 0 0 0 0 0 0 0 0 0
0 0 0 0 0
"Viruses;Viruses__na;Viruses__na;Viruses__na;Pithoviridae;Pithoviridae__na;Pithovirus_LCPAC201" 0
0 0 0 0 7 0 0 0 0 0 0 0 0
0 0 0 0 0 0 0 0 0 0 0 0 0
0 0 0 0 0 0 0 0 0 0 0 0 0
0 0 0 0 0 0 0 0 0 0 0 0 0
0 0 0 0 0 0 0 0 0 0 0 0 0
0 0 0 0 0 0
"Viruses;Viruses__na;Viruses__na;Viruses__na;Pithoviridae;Pithoviridae__na;Pithovirus_LCPAC304" 0
0 0 0 0 2 0 0 0 0 0 0 0 0
0 0 0 0 0 0 0 0 0 0 0 0 0
0 0 0 0 0 0 0 0 0 0 0 0 0
0 0 0 0 0 0 0 0 0 0 0 0 0
0 0 0 0 0 0 0 0 0 0 0 0 0
0 0 0 0 0 0
"Viruses;Viruses__na;Viruses__na;Viruses__na;Viruses__na;Pandoravirus;Pandoravirus__uc" 0 0
0 0 0 0 0 0 0 0 0 0 0 0 0
0 0 0 0 0 0 0 0 0 0 0 0 0
0 0 0 0 0 0 0 0 0 0 0 0 0
0 0 0 0 0 0 0 0 0 0 0 0 0
0 0 0 0 0 1 0 0 0 0 0 0 0
0 0 0 0 0
"Viruses;Viruses__na;Viruses__na;Viruses__na;Viruses__na;Viruses__na;Agaricus_bisporus_virus_7" 0
0 0 0 0 0 0 0 0 0 0 0 0 0
0 0 0 0 0 0 0 0 0 0 0 0 0
0 0 0 0 0 0 0 0 0 0 0 1 0
0 0 0 0 0 0 0 0 0 0 0 0 0
0 0 0 0 0 0 0 0 0 0 0 0 0
0 0 0 0 0 0
"Viruses;Viruses__na;Viruses__na;Viruses__na;Viruses__na;Viruses__na;Bacteriophage_sp." 23 34
193 26 937 218 1 0 12 5 63 0 5429 4773 10823
10367 3945 24260 3095 4593 1838 1513 865 424 4093 5325 4420 722
3011 41 1469 1425 45 13 7267 123 3943 5162 256 165 366
16384 2163 2736 4595 867 485 150 4689 2720 2803 225 419 35823
4204 6363 113 63 772 625 16030 4874 166 246 51 11 5039
6829 79 631 24354 1
"Viruses;Viruses__na;Viruses__na;Viruses__na;Viruses__na;Viruses__na;Bacteroides_phage_BS162P1" 0
0 0 0 0 0 0 0 0 0 0 0 0 1
0 0 0 0 0 0 0 0 0 0 0 0 0
0 0 0 0 0 0 0 0 0 0 0 0 0
0 0 0 0 0 0 0 0 0 0 0 0 0
0 0 0 0 0 0 0 0 0 0 0 0 0
0 0 0 0 0 0
"Viruses;Viruses__na;Viruses__na;Viruses__na;Viruses__na;Viruses__na;Blackfly_DNA_Virus_12" 0
0 0 0 6 6 0 0 0 0 0 0 0 0
0 0 0 0 0 0 0 0 0 0 0 0 0
0 0 0 0 0 0 0 0 0 0 0 0 0
0 0 0 0 0 0 0 1 0 0 0 0 0

0 0 0 0 0 0 0 0 0 0 0 0 0
0 0 0 0 0 0
"Viruses;Viruses__na;Viruses__na;Viruses__na;Viruses__na;Viruses__na;Blackfly_DNA_Virus_15" 0
0 0 0 0 0 0 0 0 0 0 0 0 0
0 0 0 0 0 0 0 0 0 0 0 0 0
0 0 0 0 0 0 0 0 0 0 0 0 0
0 0 0 0 1 0 0 0 0 0 0 0 0
0 0 0 0 0 0 0 0 0 0 0 0 0
0 0 0 0 0 0
"Viruses;Viruses__na;Viruses__na;Viruses__na;Viruses__na;Viruses__na;Blackfly_multicomponent_virus_1"
0 0 0 0 1 4 0 0 0 0 0 0 0
0 0 0 0 0 0 0 0 0 0 0 0 0
0 0 0 0 0 0 0 0 0 0 0 0 0
0 0 0 0 0 0 0 0 1 0 0 0 0
0 0 0 0 0 0 0 0 0 0 0 0 0
0 0 0 0 0 0 0
"Viruses;Viruses__na;Viruses__na;Viruses__na;Viruses__na;Viruses__na;Chimeric_virus_14" 0 0
20 66 154 54 0 0 0 0 0 0 0 0 1
1 2 0 0 0 0 0 0 0 0 0 0 0
0 1 0 0 0 0 4 0 0 0 0 0 0
0 0 0 2 0 0 30 0 0 0 1 0 0
0 0 0 0 0 0 0 0 0 0 0 0 0
0 0 1 6 0
"Viruses;Viruses__na;Viruses__na;Viruses__na;Viruses__na;Viruses__na;Chlorobiaceae_phage_CV-1-33" 0
0 1 0 1 0 0 0 0 0 0 0 0 0
0 0 0 0 0 0 0 0 0 0 0 0 0
0 0 0 0 0 0 0 0 0 0 0 0 0
0 1 0 0 0 0 0 0 0 0 0 0 0
0 0 0 0 0 0 0 0 0 0 0 0 0
0 0 0 0 0 0
"Viruses;Viruses__na;Viruses__na;Viruses__na;Viruses__na;Viruses__na;Circular_genetic_element_sp." 0
0 0 0 2 0 0 0 0 0 0 0 0 0
0 0 0 0 0 0 0 0 0 0 0 0 0
0 0 0 0 0 0 0 0 0 0 0 0 0
0 3 0 0 0 0 0 1 0 0 0 0 0
0 0 0 0 0 0 0 0 0 0 0 0 0
0 0 0 0 0 0
"Viruses;Viruses__na;Viruses__na;Viruses__na;Viruses__na;Viruses__na;Diporeia-associated_CRESS-DNA_virus_LH481" 0 0 0 0 4 0 0 0 0 0 0
0 0 0 0 0 0 0 0 0 0 0 0 0
0 0 0 0 0 0 0 0 0 0 0 0 0
0 0 0 0 0 0 0 0 0 0 0 0 0
0 0 0 0 0 0 0 0 0 0 0 0 0
0 0 0 0 0 0 0 0 0
"Viruses;Viruses__na;Viruses__na;Viruses__na;Viruses__na;Viruses__na;DNA_virus_sp." 0 0
0 0 1 0 0 0 0 0 0 0 0 0 0
0 0 0 0 0 0 0 0 0 0 0 0 0
0 0 0 0 0 0 0 0 0 0 0 0 0
0 0 0 0 0 0 0 0 0 0 0 0 0

0 0 0 0 0 0 0 0 0 0 0 0 0
0 0 0 0 0
"Viruses;Viruses__na;Viruses__na;Viruses__na;Viruses__na;Viruses__na;DP_phage" 0 0 0
0 0 0 0 0 0 0 0 0 0 0 0 0
0 0 0 0 0 0 0 0 0 0 0 0 0
0 0 0 0 0 0 0 0 0 0 0 0 0
0 0 0 0 0 0 0 0 0 0 0 0 4
4 0 0 0 0 0 0 0 0 0 0 0 0
0 0 0 0
"Viruses;Viruses__na;Viruses__na;Viruses__na;Viruses__na;Viruses__na;Dragonfly_larvae_associated_circular_
virus-1" 0 0 0 0 10 0 0 0 0 0 0 0 0
0 0 0 0 0 0 0 0 0 0 0 0 0
0 0 0 0 0 0 0 0 0 0 0 0 0
0 0 0 0 0 0 0 0 0 0 0 0 0
0 0 0 0 0 0 0 0 0 0 0 0 0
0 0 0 0 0 0 0
"Viruses;Viruses__na;Viruses__na;Viruses__na;Viruses__na;Viruses__na;Dragonfly_larvae_associated_circular_
virus-2" 0 0 0 1 6 0 0 0 0 0 0 0 0
0 0 0 0 0 0 0 0 0 0 0 0 0
0 0 0 0 0 0 0 0 0 0 0 0 0
0 0 0 0 0 0 0 0 0 0 0 0 0
0 0 0 0 0 0 0 0 0 0 0 0 0
0 0 0 0 0 0 0
"Viruses;Viruses__na;Viruses__na;Viruses__na;Viruses__na;Viruses__na;Duck_faeces_associated_circular_DNA
_virus_1" 0 0 0 2 0 0 0 0 0 0 0 0
0 0 0 0 0 0 0 0 0 0 0 0 0
0 0 0 0 0 0 0 0 0 0 0 0 0
0 0 0 0 0 0 0 0 0 0 0 0 0
0 0 0 0 0 0 0 0 0 0 0 0 0
0 0 0 0 0 0 0 0
"Viruses;Viruses__na;Viruses__na;Viruses__na;Viruses__na;Viruses__na;Faustovirus" 0 0 1
0 0 0 0 0 0 0 0 0 0 0 0 0
0 0 0 0 0 0 0 0 0 0 0 0 0
0 0 0 0 0 0 0 0 0 0 0 0 0
0 0 0 0 0 0 0 0 0 0 0 0 0
0 0 0 0 0 0 0 0 0 0 0 0 0
0 0 0 0
"Viruses;Viruses__na;Viruses__na;Viruses__na;Viruses__na;Viruses__na;HP38A_virus" 0 0 1
2 0 3 0 0 0 0 0 0 0 0 0 0
0 0 0 0 0 0 0 0 0 0 0 0 0
0 0 0 0 0 0 0 0 0 0 0 0 0
0 0 0 0 0 0 0 0 0 0 0 0 0
0 0 0 0 0 0 0 0 0 0 0 0 0
0 0 0 0
"Viruses;Viruses__na;Viruses__na;Viruses__na;Viruses__na;Viruses__na;HP38B_virus" 0 0 0
0 0 2 0 0 0 0 0 0 0 0 0 0
0 0 0 0 0 0 0 0 0 0 0 0 0
0 0 0 0 0 0 0 0 0 0 0 0 5
0 0 0 0 0 0 0 0 0 0 0 0 0

0 0 0 0 0 0 0 0 0 0 0 0 0
0 0 0 0
"Viruses;Viruses__na;Viruses__na;Viruses__na;Viruses__na;Viruses__na;Hubei_tombus-like_virus_8" 0
0 0 1 0 0 0 0 0 0 0 0 0 0
0 0 0 0 0 0 0 0 0 0 0 0 0
0 0 0 0 0 0 0 0 0 0 0 0 0
0 0 0 0 0 0 0 0 0 0 0 0 0
0 0 0 0 0 0 0 0 0 0 0 0 0
0 0 0 0 0 0
"Viruses;Viruses__na;Viruses__na;Viruses__na;Viruses__na;Viruses__na;Hudisavirus_sp." 0 0
0 0 0 0 0 0 0 0 0 0 0 0 0
0 0 0 0 0 0 0 0 0 0 0 0 0
0 0 0 0 0 0 0 2 0 0 0 0 0
0 0 0 0 0 0 0 0 0 0 0 0 0
0 0 0 0 0 0 0 0 0 0 0 0 0
0 0 0 0 0
"Viruses;Viruses__na;Viruses__na;Viruses__na;Viruses__na;Viruses__na;Lactococcus_phage_bIL311" 0
0 0 0 0 0 0 0 0 0 0 0 0 0
0 0 0 0 0 0 0 0 0 0 0 0 0
0 0 0 0 1 0 0 0 0 0 0 0 0
0 0 0 0 0 0 0 0 0 0 0 0 0
0 0 0 0 0 0 0 0 0 0 0 0 0
0 0 0 0 0 0
"Viruses;Viruses__na;Viruses__na;Viruses__na;Viruses__na;Viruses__na;Lactococcus_phage_bIL312" 0
0 0 0 0 0 0 0 0 0 0 0 0 0
0 0 0 0 0 0 0 0 0 0 0 0 0
0 0 0 0 1 0 0 0 0 0 0 0 0
0 0 0 0 0 0 0 0 0 0 0 0 0
0 0 1 0 0 0 0 0 0 0 0 0 0
0 0 0 0 0 0
"Viruses;Viruses__na;Viruses__na;Viruses__na;Viruses__na;Viruses__na;Lake_Sarah-
associated_circular_molecule_8" 0 0 0 0 7 0 0 0 0 0
0 0 0 0 0 0 0 0 0 0 0 0 0
0 0 0 0 0 0 0 0 0 0 0 0 0
0 0 0 0 0 0 0 0 0 0 0 0 0
0 0 0 0 0 0 0 0 0 0 0 0 0
0 0 0 0 0 0 0 0 0 0
"Viruses;Viruses__na;Viruses__na;Viruses__na;Viruses__na;Viruses__na;Lake_Sarah-associated_circular_virus-
28" 0 0 0 0 0 4 0 0 0 0 0 0 0
0 0 0 0 0 0 0 0 0 0 0 0 0
0 0 0 0 0 0 0 0 0 0 0 0 0
0 0 4 0 0 0 0 0 0 0 0 0 0
0 0 0 0 0 0 0 0 0 0 0 0 0
0 0 0 0 0 0 0
"Viruses;Viruses__na;Viruses__na;Viruses__na;Viruses__na;Viruses__na;Lake_Sarah-associated_circular_virus-
41" 0 0 0 0 10 0 0 0 0 0 0 0 0
0 0 0 0 0 0 0 0 0 0 0 0 0
0 0 0 0 0 0 0 0 0 0 0 0 0
0 0 0 0 0 0 0 0 0 0 0 0 0

0 0 0 0 0 0 0 0 0 0 0 0 0
0 0 0 0 0 0 0
"Viruses;Viruses__na;Viruses__na;Viruses__na;Viruses__na;Viruses__na;McMurdo_Ice_Shelf_pond-associated_circular_DNA_virus-3" 0 0 0 0 0 0 0 0 0
0 0 0 0 0 0 0 0 0 0 0 0 0
0 0 0 0 0 0 0 0 0 0 0 0 6
0 0 0 0 0 0 0 0 0 0 0 0 0
0 0 0 0 0 0 0 0 0 0 0 0 0
0 0 0 0 0 0 0 0 0 0 0
"Viruses;Viruses__na;Viruses__na;Viruses__na;Viruses__na;Viruses__na;Methanosarcina_virus_MetMV" 0
0 0 0 1 0 0 0 0 0 0 0 0 0
0 0 0 0 0 0 0 0 0 0 0 0 0
0 0 0 0 0 0 0 0 0 0 0 0 0
0 0 0 0 0 0 0 0 0 0 0 0 0
0 0 0 0 0 0 0 0 0 0 0 0 0
0 0 0 0 0 0
"Viruses;Viruses__na;Viruses__na;Viruses__na;Viruses__na;Viruses__na;Ochrobactrum_phage_ORM_20"0
0 0 0 41 0 0 0 0 0 0 0 0 0
0 0 0 0 0 0 0 0 0 0 0 0 0
0 0 0 0 0 0 0 0 0 0 0 0 0
0 0 0 0 0 0 0 0 0 0 0 0 0
0 0 0 0 0 0 0 0 0 0 0 0 0
0 0 0 0 0 0
"Viruses;Viruses__na;Viruses__na;Viruses__na;Viruses__na;Viruses__na;Octadecabacter_Antarctic_DB_virus_2"
0 0 0 3 3 0 0 0 0 0 0 0 0
0 0 0 0 0 0 0 0 0 0 0 0 0
0 0 0 0 0 0 0 0 0 0 0 0 0
0 0 0 0 0 0 0 0 0 0 0 0 0
0 0 0 0 0 0 0 0 0 0 0 0 0
0 0 0 0 0 0 0
"Viruses;Viruses__na;Viruses__na;Viruses__na;Viruses__na;Viruses__na;Paraglaciecola_Antarctic_JLT_virus_2"
0 0 0 1 0 0 0 0 0 0 0 0 0
0 0 0 0 0 0 0 0 0 0 0 0 0
0 0 0 0 0 0 0 0 0 0 0 0 0
0 0 0 0 0 0 0 0 0 0 0 0 0
0 0 0 0 0 0 0 0 0 0 0 0 0
0 0 0 0 0 0 0
"Viruses;Viruses__na;Viruses__na;Viruses__na;Viruses__na;Viruses__na;Phage_sp._ctcqm2" 0 0
0 0 0 0 0 0 0 0 0 0 0 0 0
0 0 0 1 2 0 0 0 0 0 0 0 0
0 0 0 0 0 0 0 0 0 1 0 0 0
0 1 3 0 0 0 0 0 0 0 0 1 0
0 0 0 0 0 0 0 0 0 0 0 0 0
0 0 0 0 0
"Viruses;Viruses__na;Viruses__na;Viruses__na;Viruses__na;Viruses__na;Phage_sp._ctez94" 0 0
0 0 0 0 0 0 0 0 0 0 0 0 0
0 0 11 0 0 0 0 0 0 0 0 0 0
0 0 0 0 0 0 0 0 0 0 0 0 0
9 0 0 0 0 0 0 0 0 0 0 0 0

0 1 0 0 0 0 0 0 0 0 0 0 0
1 0 0 0 0
"Viruses;Viruses__na;Viruses__na;Viruses__na;Viruses__na;Viruses__na;Phage_sp._ctfRs3" 0 0
0 0 0 0 0 0 0 0 0 0 0 0 0
0 0 0 0 0 0 0 0 0 0 0 0 0
0 0 0 0 0 0 0 0 3 14 0 0 0
0 0 0 0 0 0 0 0 0 0 0 0 0
0 0 0 0 0 0 0 0 0 0 0 0 0
1 0 0 0 0
"Viruses;Viruses__na;Viruses__na;Viruses__na;Viruses__na;Viruses__na;Phage_sp._ctIHi3" 0 0
0 0 0 0 0 0 0 0 0 0 23 13 1
2 0 0 0 0 0 0 0 0 0 0 0 0
0 0 4 4 0 0 0 0 1 2 0 0 0
0 0 0 0 0 0 0 0 0 0 0 0 0
0 1 0 0 0 0 0 0 0 0 0 0 0
1 0 0 0 0
"Viruses;Viruses__na;Viruses__na;Viruses__na;Viruses__na;Viruses__na;Phage_sp._ctqZP6" 0 0
0 0 0 0 0 0 0 0 0 0 0 0 0
0 0 0 0 0 0 0 0 0 0 0 0 0
0 1 0 0 0 0 0 0 0 0 50 29 0
10035 0 0 0 0 0 0 0 0 0 0 184 0
1 0 0 0 0 0 0 0 0 0 0 0 0
0 0 0 0 0
"Viruses;Viruses__na;Viruses__na;Viruses__na;Viruses__na;Viruses__na;Phage_sp._ctrsQ3" 0 0
0 0 0 0 0 0 0 0 0 0 0 0 0
0 0 0 0 0 0 0 0 0 0 0 0 0
0 0 0 0 0 0 0 0 3 5 0 2 0
0 0 0 0 0 0 0 0 0 0 0 0 0
5 11 0 0 0 0 0 0 0 0 0 0 11
9 0 0 0 0
"Viruses;Viruses__na;Viruses__na;Viruses__na;Viruses__na;Viruses__na;Phage_sp._ctSLR2" 0 0
0 0 0 0 0 0 0 0 0 0 0 0 0
0 0 0 0 0 0 0 0 0 0 0 0 0
0 0 0 0 0 0 0 0 1 1 0 0 0
0 0 0 0 0 0 0 0 0 0 0 0 0
0 0 0 0 0 0 0 0 0 0 0 0 0
0 0 0 0 0
"Viruses;Viruses__na;Viruses__na;Viruses__na;Viruses__na;Viruses__na;Phage_sp._ctucZ11" 0 0
0 0 0 0 0 0 0 0 0 0 0 0 0
0 0 0 0 0 0 0 0 0 0 0 0 0
0 0 0 0 0 0 0 0 0 0 0 0 0
0 0 0 0 0 0 0 0 0 0 0 0 0
1 0 0 0 0 0 0 0 0 0 0 0 0
0 0 0 0 0
"Viruses;Viruses__na;Viruses__na;Viruses__na;Viruses__na;Viruses__na;Phage_sp._ctWVj20" 0 0
0 0 0 0 0 0 0 0 0 0 0 0 0
0 0 0 0 0 0 0 0 0 0 0 0 0
0 0 0 0 0 0 0 0 0 0 0 0 0
0 0 0 0 0 0 0 0 0 0 0 0 0

1 0 0 0 0 0 0 0 0 0 0 0 0
0 0 0 0 0
"Viruses;Viruses__na;Viruses__na;Viruses__na;Viruses__na;Viruses__na;Phage_sp._ctXnn1" 0 0
0 0 0 0 0 0 0 0 0 0 0 0 0
0 0 0 0 0 0 0 0 0 0 0 0 0
0 0 0 0 0 0 0 0 3 1 0 0 0
0 0 0 0 0 0 0 0 0 0 0 0 0
0 0 0 0 0 0 0 1 0 0 0 0 0
0 0 0 0 0
"Viruses;Viruses__na;Viruses__na;Viruses__na;Viruses__na;Viruses__na;Phage_sp._cty4N14" 0 0
0 0 0 0 0 0 0 0 0 0 2 0 0
0 0 0 0 0 0 0 0 0 0 1 0 0
0 0 0 0 0 0 0 0 5 6 0 0 0
0 0 0 0 0 0 0 0 0 0 0 0 0
0 0 0 0 0 0 0 0 0 0 0 0 0
0 0 0 0 0
"Viruses;Viruses__na;Viruses__na;Viruses__na;Viruses__na;Viruses__na;Prokaryotic_dsDNA_virus_sp." 8
7 34 73 353 335 0 2 0 5 0 0 0 0
0 0 1 2 0 0 0 0 5 9 0 0 0
0 1 0 0 0 0 1 4 4 0 0 0 0
0 79 0 0 0 0 22 50 0 0 0 1 1
0 0 0 0 0 0 26 6 1 3 3 0 0
0 0 1 0 10 0
"Viruses;Viruses__na;Viruses__na;Viruses__na;Viruses__na;Viruses__na;Pseudomonas_phage_PPAT" 0
0 0 1 2 1 0 0 0 0 0 0 0 0
0 0 0 0 0 0 0 0 0 0 0 0 0
0 0 0 0 0 0 0 0 0 0 0 0 0
0 0 0 0 0 0 0 0 0 0 0 0 0
0 0 0 0 0 0 0 0 0 0 0 0 0
0 0 0 0 0 0
"Viruses;Viruses__na;Viruses__na;Viruses__na;Viruses__na;Viruses__na;Reptile-
associated_circular_DNA_molecule" 0 0 0 0 0 0 0 0 0
0 0 0 0 0 0 0 0 2 0 0 0 0
0 0 0 0 0 0 0 0 0 0 0 0 0
0 0 0 0 0 0 0 0 0 0 0 0 0
0 0 0 0 0 0 0 0 0 0 0 0 0
0 0 0 0 0 0 0 0 0 0 0
"Viruses;Viruses__na;Viruses__na;Viruses__na;Viruses__na;Viruses__na;Riboviria_sp." 0 0 0
0 0 12 0 0 0 0 0 0 0 0 12 8
7 0 0 0 0 0 0 0 0 0 0 0 0
0 0 0 0 0 0 0 0 0 0 0 0 0
0 0 0 0 0 0 0 0 0 0 0 0 0
0 0 0 0 0 0 0 0 0 0 0 0 0
0 0 0 0
"Viruses;Viruses__na;Viruses__na;Viruses__na;Viruses__na;Viruses__na;Salmonella_phage_Arash" 0
0 0 0 0 0 0 0 0 0 0 0 0 0
0 0 1 0 0 0 0 0 0 0 0 0 0
0 0 0 0 0 0 0 0 0 0 0 0 0
0 0 0 0 0 0 0 0 0 0 0 0 0

0 0 0 0 0 0 0 0 0 0 0 0 0
0 0 0 0 0 0
"Viruses;Viruses__na;Viruses__na;Viruses__na;Viruses__na;Viruses__na;Sewage-associated_circular_DNA_molecule" 0 0 4 3 98 24 0 0 0
0 0 0 0 0 0 0 1 0 0 0 0 0
0 0 0 0 0 0 0 3 0 0 0 0 0
0 0 0 0 0 0 0 0 0 1 0 0 22
0 0 1 0 0 1 0 0 0 0 0 0 0
0 0 0 0 0 0 0 0 0 0 0
"Viruses;Viruses__na;Viruses__na;Viruses__na;Viruses__na;Viruses__na;Sewage-associated_circular_DNA_virus-14" 0 0 1 2 1 8 0 0 0
0 0 0 0 0 0 0 0 0 0 0 0 0
0 0 0 0 0 0 0 0 0 0 0 0 0
0 0 0 0 0 0 0 0 0 0 0 0 0
0 0 0 0 0 0 0 0 0 0 0 0 0
0 0 0 0 0 0 0 0 0 0 0
"Viruses;Viruses__na;Viruses__na;Viruses__na;Viruses__na;Viruses__na;Sewage-associated_circular_DNA_virus-19" 0 0 0 0 0 1 0 0 0
0 0 0 0 0 0 0 0 0 0 0 0 0
0 1 0 0 0 0 0 0 0 0 0 0 0
0 0 0 0 0 0 0 0 0 0 0 0 1
0 0 0 0 0 0 0 0 0 0 0 0 0
0 0 0 0 0 0 0 0 0 0 0
"Viruses;Viruses__na;Viruses__na;Viruses__na;Viruses__na;Viruses__na;Sewage-associated_circular_DNA_virus-20" 0 0 0 1 0 9 0 0 0
0 0 0 0 0 0 0 0 0 0 0 0 0
0 0 0 0 0 0 0 0 0 0 0 0 0
0 0 0 0 0 0 0 0 0 0 0 0 3
0 0 0 0 0 0 0 0 0 0 0 0 0
0 0 0 0 0 0 0 0 0 0 0
"Viruses;Viruses__na;Viruses__na;Viruses__na;Viruses__na;Viruses__na;Sewage-associated_circular_DNA_virus-28" 0 0 0 1 0 0 0 0 0
0 0 0 0 0 0 0 0 0 0 0 0 0
0 0 0 0 0 0 0 0 0 0 0 0 0
0 0 0 0 0 0 0 0 0 0 0 0 0
0 0 0 0 0 0 0 0 0 0 0 0 0
0 0 0 0 0 0 0 0 0 0 0
"Viruses;Viruses__na;Viruses__na;Viruses__na;Viruses__na;Viruses__na;Sewage-associated_circular_DNA_virus-8" 0 0 0 0 0 0 0 0 0
0 0 0 0 0 0 0 0 0 0 0 0 0
0 0 0 0 0 0 0 0 0 0 0 0 0
0 0 0 0 0 0 0 0 0 0 0 0 1
0 0 0 0 0 0 0 0 0 0 0 0 0
0 0 0 0 0 0 0 0 0 0 0
"Viruses;Viruses__na;Viruses__na;Viruses__na;Viruses__na;Viruses__na;Skeletonema_virus_LDF-2015a" 0
0 0 1 0 0 0 0 0 0 0 0 0 0
0 0 0 0 0 0 0 0 0 0 0 0 0
0 0 0 0 0 0 0 0 0 0 0 0 0
0 0 0 0 0 0 0 0 0 0 0 0 0

0 0 0 0 0 0 0 0 0 0 0 0 0
0 0 0 0 0 0
"Viruses;Viruses__na;Viruses__na;Viruses__na;Viruses__na;Viruses__na;ssDNA_virus_sp." 0 0
0 8 12 15 0 0 0 0 0 0 0 0 0
0 0 0 0 0 0 0 0 0 0 0 0 0
0 0 0 0 0 0 0 0 0 0 0 0 0
0 0 0 0 0 0 8 0 0 0 0 0 0
0 0 0 0 0 0 0 0 0 0 0 0 0
0 0 0 0 0
"Viruses;Viruses__na;Viruses__na;Viruses__na;Viruses__na;Viruses__na;Streptococcus_satellite_phage_Javan32
7" 0 0 0 0 0 0 0 0 0 0 0 0 0
0 0 0 0 0 0 0 0 0 0 0 0 0
0 0 0 0 0 0 0 0 0 0 0 0 0
0 0 0 0 0 0 0 1 0 0 0 0 0
0 0 0 0 0 0 0 0 0 0 0 0 0
0 0 0 0 0 0 0
"Viruses;Viruses__na;Viruses__na;Viruses__na;Viruses__na;Viruses__na;Streptococcus_satellite_phage_Javan36
0" 0 0 0 0 0 0 0 0 0 0 0 0 0
0 0 0 0 0 0 0 0 0 0 0 0 0
0 0 0 0 0 0 0 0 0 0 0 0 0
0 0 0 0 0 0 0 0 0 0 0 0 0
0 0 0 0 0 0 0 0 0 0 0 0 0
0 0 0 0 0 1 0
"Viruses;Viruses__na;Viruses__na;Viruses__na;Viruses__na;Viruses__na;Streptomyces_phage_Dagobah" 0
1 0 0 0 0 0 0 0 0 0 0 0 0
0 0 0 0 0 0 0 0 0 0 0 0 0
0 0 0 0 0 0 0 0 0 0 0 0 0
0 0 0 0 0 0 0 0 0 0 0 0 0
0 0 0 0 0 0 0 0 0 0 0 0 0
0 0 0 0 0 0
"Viruses;Viruses__na;Viruses__na;Viruses__na;Viruses__na;Viruses__na;Synechococcus_phage_S-EIVl" 0
0 0 0 11 0 0 0 0 0 0 0 0 0
0 0 0 0 0 0 0 0 0 0 0 0 0
0 0 0 0 0 0 0 0 0 0 0 0 0
0 0 0 0 0 0 0 0 0 0 0 0 0
0 0 0 0 0 0 0 0 0 0 0 0 0
0 0 0 0 0 0
"Viruses;Viruses__na;Viruses__na;Viruses__na;Viruses__na;Viruses__na;Tick-
associated_circular_DNA_satellite" 0 0 8 17 131 22 0 0 0
0 0 0 0 0 0 0 1 15 0 0 0 0
0 0 0 0 0 0 0 0 0 0 0 0 0
0 0 0 1 0 0 0 0 0 1 0 0 1
0 0 0 0 0 0 0 0 0 0 0 0 0
0 0 0 0 0 0 0 0 0 1 0
"Viruses;Viruses__na;Viruses__na;Viruses__na;Viruses__na;Viruses__na;Tick-associated_circular_DNA_virus"
1 2 165 522 2030 5624 0 0 0 0 0 0 0
0 0 0 37 38 0 0 1 0 9 16 0 0
2 0 0 1 0 0 0 0 17 5 0 0 0
2 6 257 0 0 15 3 20 203 0 0 1 0

1 47 0 0 0 0 0 0 0 0 0 0 0
0 0 0 0 0 84 0
"Viruses;Viruses__na;Viruses__na;Viruses__na;Viruses__na;Viruses__na;uncultured_archaeal_virus" 0
0 0 0 0 7 0 0 0 0 0 0 0 0
0 0 0 0 0 0 0 0 0 0 0 0 0
0 0 0 0 0 0 0 0 0 0 0 0 0
0 0 0 0 0 0 0 0 0 0 0 0 0
0 0 0 0 0 0 0 0 0 0 0 0 0
0 0 0 0 0 0
"Viruses;Viruses__na;Viruses__na;Viruses__na;Viruses__na;Viruses__na;uncultured_cyanophage" 1
0 0 2 17 1 0 0 0 1 0 0 0 0
0 0 0 1 0 0 0 0 0 0 0 0 0
0 0 0 0 0 0 0 0 0 0 0 0 0
0 1 0 0 0 0 0 0 0 0 0 0 0
0 0 0 0 0 0 0 0 0 0 0 0 0
0 0 0 0 0 0
"Viruses;Viruses__na;Viruses__na;Viruses__na;Viruses__na;Viruses__na;uncultured_marine_phage" 0
0 0 0 13 21 0 0 0 0 0 0 0 0
0 0 0 0 0 0 0 0 0 0 0 0 0
0 0 0 0 0 0 0 0 0 0 0 0 0
0 0 0 0 0 0 0 0 0 0 0 0 1
0 0 0 0 0 0 0 0 0 0 0 0 0
1 0 0 0 0 0
"Viruses;Viruses__na;Viruses__na;Viruses__na;Viruses__na;Viruses__na;uncultured_marine_virus" 1
3 4 14 84 64 0 0 0 0 0 0 1 0
0 0 4 0 0 0 0 0 2 0 0 0 0
0 0 1 0 0 0 0 0 0 0 0 0 0
0 0 0 0 0 1 0 14 0 0 0 0 1
0 0 0 0 0 0 0 0 0 0 0 0 0
0 0 0 0 6 0
"Viruses;Viruses__na;Viruses__na;Viruses__na;Viruses__na;Viruses__na;uncultured_Mediterranean_phage"
0 0 0 0 8 1 0 0 0 0 0 0 0
0 0 0 0 0 0 0 0 0 0 0 0 0
0 0 0 0 0 0 0 0 0 0 0 0 0
0 0 0 0 0 0 0 0 4 0 0 0 0
0 0 0 0 0 0 0 0 0 0 0 0 0
0 0 0 0 0 2 0
"Viruses;Viruses__na;Viruses__na;Viruses__na;Viruses__na;Viruses__na;uncultured_phage" 1 0
1 3 2 4 0 0 0 0 0 0 0 0 0
0 0 0 0 0 0 0 0 0 0 0 0 0
0 0 0 0 0 0 0 0 0 0 0 0 0
0 0 0 4 0 0 3 0 0 0 0 0 0
0 0 0 0 0 0 0 0 0 0 0 0 0
0 0 0 0 0
"Viruses;Viruses__na;Viruses__na;Viruses__na;Viruses__na;Viruses__na;uncultured_virus" 7 2
16 52 398 236 0 0 0 0 0 0 0 0 2
0 0 0 0 0 0 0 1 7 0 0 0 0
0 0 0 0 0 0 1 0 0 0 0 0 1
12 0 0 1 0 10 39 0 0 0 1 1 1

0 0 0 0 0 0 0 0 0 0 0 0 0
0 0 0 7 0
"Viruses;Viruses__na;Viruses__na;Viruses__na;Viruses__na;Viruses__na;Variovorax_phage_VAC_51" 0
0 0 0 1 0 0 0 0 0 0 0 0 0
0 0 1 0 0 0 0 0 0 0 0 0 0
0 0 0 0 0 0 0 0 0 0 0 0 0
0 0 0 0 0 0 0 0 0 0 0 0 0
0 0 0 0 0 0 0 0 0 0 0 0 0
0 0 0 0 0 0
"Viruses;Viruses__na;Viruses__na;Viruses__na;Viruses__na;Viruses__na;Vibrio_phage_VH-P22" 0 0
0 1 0 0 0 0 0 0 0 0 0 0 0
0 0 0 0 0 0 0 0 0 0 0 0 0
0 0 0 0 0 0 0 0 0 0 0 0 0
0 0 0 0 0 0 0 0 0 0 0 0 0
0 0 0 0 0 0 0 0 0 0 0 0 0
0 0 0 0 0
"Viruses;Viruses__na;Viruses__na;Viruses__na;Viruses__na;Viruses__na;Virus_NIOZ-UU157" 0 3
2 4 184 47 0 0 0 0 0 0 0 0 0
0 0 0 0 0 0 0 0 0 0 0 0 0
0 0 0 0 0 0 0 0 0 0 0 0 0
0 0 0 0 0 0 10 0 0 0 0 0 0
0 0 0 0 0 0 0 0 0 0 0 0 0
0 0 0 1 0
"Viruses;Viruses__na;Viruses__na;Viruses__na;Viruses__na;Viruses__na;Virus_Rctr71" 0 0 0
0 0 0 0 0 0 0 0 0 0 0 0 0
0 0 0 0 0 0 0 0 0 0 0 0 0
0 0 0 0 0 0 0 0 0 0 0 0 0
0 0 0 0 0 3 0 0 0 0 0 0 0
0 0 0 0 0 0 0 0 0 0 0 0 0
0 0 0 0
"Viruses;Viruses__na;Viruses__na;Viruses__na;Viruses__na;Viruses__na;Virus_sp." 0 0 0
0 0 0 0 0 0 0 0 2 0 0 0 0
0 0 0 0 0 0 0 0 0 0 0 0 0
0 0 0 0 0 0 0 0 0 0 0 0 0
0 0 0 0 0 0 0 0 0 1 0 0 0
0 0 0 0 0 0 0 0 0 0 0 0 0
0 0 0 0
"Viruses;Viruses__na;Viruses__na;Viruses__na;Viruses__na;Viruses__na;virus_sp._ct1Hk25" 0 0
0 0 0 0 0 0 0 0 0 0 0 0 0
0 0 0 0 0 0 0 0 0 0 0 0 0
0 0 0 0 0 0 0 0 0 0 0 0 0
0 0 0 0 0 0 0 0 0 0 0 0 0
0 0 0 0 0 0 0 0 0 0 0 0 0
1 0 0 0 0
"Viruses;Viruses__na;Viruses__na;Viruses__na;Viruses__na;Viruses__na;virus_sp._ct5rm7" 0 0
0 0 0 0 0 0 0 0 0 0 24 10 11
15 21 14 20 14 1 3 1 0 1 1 3 0
0 0 0 1 0 0 0 0 2 6 0 2 0
0 18 10 0 0 0 0 1 0 0 0 0 0

8 6 0 0 0 0 2 1 0 0 0 0 2
1 0 0 0 0
"Viruses;Viruses__na;Viruses__na;Viruses__na;Viruses__na;Viruses__na;virus_sp._ct6Ax4" 0 0
0 0 0 0 0 0 0 0 0 0 0 0 0
0 0 0 0 0 0 0 0 0 0 0 0 0
0 0 0 0 0 0 0 0 0 0 0 0 0
0 0 0 0 0 0 0 0 0 0 0 0 0
0 0 0 0 0 0 0 0 0 0 0 0 2
3 0 1 0 0
"Viruses;Viruses__na;Viruses__na;Viruses__na;Viruses__na;Viruses__na;virus_sp._ct6GG30" 0 0
0 0 0 0 0 0 0 0 0 0 0 0 0
0 0 0 0 0 0 0 0 0 0 0 0 0
0 0 0 0 0 0 0 0 1 0 0 0 0
0 0 0 0 0 0 0 0 0 0 0 0 0
0 0 0 0 0 0 0 0 0 0 0 0 0
0 0 0 0 0
"Viruses;Viruses__na;Viruses__na;Viruses__na;Viruses__na;Viruses__na;virus_sp._ct6zc1" 0 0
0 0 0 0 0 0 0 0 0 0 0 0 0
0 0 0 0 0 0 0 0 0 0 0 0 0
0 0 0 0 0 0 0 0 0 0 0 0 0
0 0 0 0 0 0 0 0 0 0 0 0 0
0 4 0 0 0 0 0 0 0 0 0 0 0
0 0 0 0 0
"Viruses;Viruses__na;Viruses__na;Viruses__na;Viruses__na;Viruses__na;virus_sp._ct9pU4" 0 0
0 0 0 0 0 0 0 0 0 0 0 0 6
3 2 7 0 0 0 0 0 0 0 0 0 0
0 0 0 0 0 0 0 0 0 0 0 0 0
0 0 0 0 0 0 0 0 0 0 0 0 0
2 2 0 0 0 0 0 0 0 0 0 0 0
0 0 0 0 0
"Viruses;Viruses__na;Viruses__na;Viruses__na;Viruses__na;Viruses__na;Virus_sp._ctAgr11" 0 0
0 0 0 0 0 0 0 0 0 0 0 0 0
0 0 0 1 0 0 0 0 0 0 0 0 0
0 0 0 0 0 0 0 0 0 0 0 0 0
0 0 0 0 0 0 0 0 0 0 0 0 0
0 0 0 0 0 0 0 0 0 0 0 0 0
0 0 0 0 0
"Viruses;Viruses__na;Viruses__na;Viruses__na;Viruses__na;Viruses__na;virus_sp._ctah610" 0 0
0 0 0 0 0 0 0 0 0 0 0 0 0
0 0 0 0 0 0 0 0 0 0 0 0 0
0 0 0 0 0 0 0 0 0 0 0 0 0
0 0 0 0 0 0 0 0 0 0 0 0 0
0 0 0 0 0 0 0 0 0 0 0 0 0
1 0 0 0 0
"Viruses;Viruses__na;Viruses__na;Viruses__na;Viruses__na;Viruses__na;virus_sp._ctBS918" 0 0
0 0 0 0 0 0 0 0 0 0 0 0 0
0 0 0 0 0 0 0 0 0 0 0 0 0
0 0 0 0 0 0 0 0 0 0 1 0 0
0 0 0 0 0 0 0 0 0 0 0 0 0

0 0 0 0 0 0 0 0 0 0 0 0 0
0 0 0 0 0
"Viruses;Viruses__na;Viruses__na;Viruses__na;Viruses__na;Viruses__na;virus_sp._ctCsQ3" 0 0
0 0 0 0 0 0 0 0 0 0 0 0 0
0 0 0 0 0 0 0 0 0 0 0 0 0
0 0 0 0 0 0 0 0 1 0 0 0 1
0 2 3 1 2 0 0 0 0 0 0 0 365
1 1 0 0 0 0 0 2 0 0 0 0 3
3 0 4 0 0
"Viruses;Viruses__na;Viruses__na;Viruses__na;Viruses__na;Viruses__na;virus_sp._ctd0M1" 0 0
0 0 0 0 0 0 0 0 0 0 2 3 1
1 0 0 0 0 0 0 0 0 0 0 0 0
0 0 0 0 0 0 0 0 7 4 0 0 0
0 1 1 0 0 0 0 0 0 0 0 0 0
0 4 0 0 0 0 0 0 0 0 0 0 0
0 0 0 0 0
"Viruses;Viruses__na;Viruses__na;Viruses__na;Viruses__na;Viruses__na;virus_sp._ctE0n6" 0 0
0 0 0 0 0 0 0 0 0 0 0 0 0
0 0 0 0 0 0 0 0 0 0 0 0 0
0 0 0 0 0 0 0 0 0 0 0 0 0
0 0 0 0 0 0 0 0 0 0 0 0 0
0 3 0 0 0 0 0 0 0 0 0 0 0
0 0 0 0 0
"Viruses;Viruses__na;Viruses__na;Viruses__na;Viruses__na;Viruses__na;virus_sp._ctEfN2" 0 0
0 0 0 0 0 0 0 0 0 0 0 0 0
0 0 0 0 0 0 0 2 0 6 9 19 1
27 2 0 0 0 0 0 0 0 0 0 0 0
0 0 0 0 0 0 0 0 0 0 0 0 0
0 0 0 0 0 0 0 1 0 0 0 0 0
0 0 0 0 0
"Viruses;Viruses__na;Viruses__na;Viruses__na;Viruses__na;Viruses__na;virus_sp._ctFlR8" 0 0
0 0 0 0 0 0 0 0 0 0 0 0 0
0 0 0 0 0 0 0 0 0 0 0 0 0
0 0 0 0 0 0 0 0 0 0 0 0 0
0 0 0 0 0 0 0 0 0 0 0 0 65
0 0 0 0 0 0 0 0 0 0 0 0 3
4 0 4 0 0
"Viruses;Viruses__na;Viruses__na;Viruses__na;Viruses__na;Viruses__na;virus_sp._ctHG14" 0 0
0 0 0 0 0 0 0 0 0 0 0 2 0
0 0 0 0 0 0 0 0 0 0 1 0 0
0 0 0 0 0 0 4 0 0 0 0 0 0
0 0 1 0 0 0 0 2 0 0 0 0 0
0 1 0 0 0 0 0 0 0 0 0 0 0
2 0 0 0 0
"Viruses;Viruses__na;Viruses__na;Viruses__na;Viruses__na;Viruses__na;virus_sp._ctiha2" 0 1
0 0 0 0 0 0 0 0 0 0 0 0 0
0 0 0 0 2 1 0 0 0 0 0 0 0
0 0 0 0 0 0 0 0 0 0 0 0 0
0 0 1 0 0 0 0 0 0 0 0 0 0

0 0 0 0 0 0 0 0 0 0 0 0 2
0 0 0 0 0
"Viruses;Viruses__na;Viruses__na;Viruses__na;Viruses__na;Viruses__na;virus_sp._ctIVh9" 0 0
0 0 0 0 0 0 0 0 0 0 0 0 0
0 0 0 0 0 0 0 0 0 0 0 0 0
0 0 0 0 0 0 0 0 0 0 0 0 0
0 0 0 0 0 0 0 1 0 0 0 0 0
0 0 0 0 0 0 0 0 0 0 0 0 0
0 0 0 0 0
"Viruses;Viruses__na;Viruses__na;Viruses__na;Viruses__na;Viruses__na;virus_sp._ctJLD79" 0 0
0 0 0 0 0 0 0 0 0 0 0 0 0
0 0 1 0 0 0 0 0 0 0 0 0 0
0 0 1 2 0 0 0 0 0 0 0 0 0
0 0 0 0 0 0 0 0 0 0 0 0 0
2 1 0 0 0 0 0 0 0 0 0 0 0
0 0 0 0 0
"Viruses;Viruses__na;Viruses__na;Viruses__na;Viruses__na;Viruses__na;virus_sp._ctLpa4" 0 0
0 0 0 0 0 0 0 0 0 0 0 0 1
1 0 0 1 0 1 1 0 0 1 1 0 0
0 0 3 1 0 0 0 0 0 0 0 0 0
0 2 1 0 0 0 0 1 0 0 0 0 0
0 0 0 0 0 0 9 1 0 0 0 0 0
0 0 0 0 0
"Viruses;Viruses__na;Viruses__na;Viruses__na;Viruses__na;Viruses__na;virus_sp._ctLTC15" 0 0
0 0 0 0 0 0 0 0 0 0 0 0 0
0 0 0 0 0 0 0 0 0 0 0 0 0
0 0 0 0 0 0 0 0 0 0 0 0 0
0 0 0 0 0 0 0 0 0 0 0 0 8
0 0 0 0 0 0 0 0 0 0 0 0 0
1 0 0 0 0
"Viruses;Viruses__na;Viruses__na;Viruses__na;Viruses__na;Viruses__na;virus_sp._ctML55" 0 0
0 0 0 0 0 0 0 0 0 0 38 28 197
168 111 134 0 0 0 0 0 0 0 0 0 0
0 0 0 0 0 0 0 1 0 0 0 0 69
0 77 53 1262 267 0 9 0 0 0 0 0 0
0 0 0 0 0 0 0 0 0 0 0 0 0
0 0 0 0 0
"Viruses;Viruses__na;Viruses__na;Viruses__na;Viruses__na;Viruses__na;virus_sp._ctmTa7" 0 0
0 0 0 0 0 0 0 0 0 0 0 0 0
0 0 0 1 0 0 0 0 0 0 0 0 0
0 0 1 0 0 0 0 0 2 2 0 0 0
0 0 0 0 0 0 0 0 0 1 0 0 0
1 1 0 0 0 0 1 2 0 0 0 0 4
5 0 0 0 0
"Viruses;Viruses__na;Viruses__na;Viruses__na;Viruses__na;Viruses__na;virus_sp._ctpeS3" 0 0
0 0 0 0 0 0 0 0 0 0 0 0 0
0 0 0 0 0 0 0 1 0 0 0 0 0
0 0 0 0 0 0 1 0 0 0 0 0 0
0 0 0 0 0 0 0 0 0 0 0 0 0

0 0 0 0 0 0 0 0 0 0 0 0 0
0 0 0 0 0
"Viruses;Viruses__na;Viruses__na;Viruses__na;Viruses__na;Viruses__na;virus_sp._ctPLL24" 0 0
0 0 0 0 0 0 0 0 0 0 0 4 0
0 0 0 0 0 0 0 0 0 0 0 0 0
0 0 0 0 0 0 0 0 0 0 0 0 0
0 0 0 0 0 0 0 0 0 0 0 0 0
0 0 0 0 0 0 0 0 0 0 0 0 0
0 0 0 0 0
"Viruses;Viruses__na;Viruses__na;Viruses__na;Viruses__na;Viruses__na;virus_sp._ctqq75" 0 0
0 0 0 0 0 0 0 0 0 0 0 0 0
0 0 0 0 0 0 0 0 0 0 0 0 0
0 0 0 0 0 0 0 0 0 0 0 0 0
0 3 5 0 0 0 0 0 0 0 0 0 0
0 0 0 0 0 0 0 0 0 0 0 0 0
0 0 0 0 0
"Viruses;Viruses__na;Viruses__na;Viruses__na;Viruses__na;Viruses__na;virus_sp._ctQSn51" 0 0
0 0 0 0 0 0 0 0 0 0 0 0 0
0 0 0 0 0 0 0 0 0 0 0 0 0
0 0 0 0 0 0 0 0 1 0 0 0 0
0 0 0 0 0 0 0 0 0 0 0 0 0
0 0 0 0 0 0 0 0 0 0 0 0 0
0 0 0 0 0
"Viruses;Viruses__na;Viruses__na;Viruses__na;Viruses__na;Viruses__na;virus_sp._ctr1v16" 0 0
0 0 0 0 0 0 0 0 0 0 0 0 0
0 0 0 0 1 0 0 0 0 1 1 0 0
0 0 0 4 0 0 0 0 3 7 0 0 0
0 0 0 0 0 0 0 0 0 0 0 0 0
64 88 0 0 0 0 0 0 0 0 0 0 28
36 0 0 0 0
"Viruses;Viruses__na;Viruses__na;Viruses__na;Viruses__na;Viruses__na;virus_sp._ctReX5" 0 0
0 0 0 0 0 0 0 0 0 0 0 0 0
0 0 0 2 1 0 0 1 2 1 4 1 0
0 0 0 0 0 0 2 0 3 7 0 0 0
0 1 0 0 0 0 0 0 1 2 0 0 0
4 8 0 0 0 0 14 1 0 0 0 0 1
0 0 0 0 0
"Viruses;Viruses__na;Viruses__na;Viruses__na;Viruses__na;Viruses__na;virus_sp._ctRTq15" 0 0
0 0 0 0 0 0 0 0 0 0 0 0 0
0 0 0 0 0 0 0 0 0 0 0 0 0
0 0 0 0 0 0 0 0 0 0 0 0 0
0 0 0 0 0 0 0 0 0 0 0 0 12
0 0 0 0 0 0 0 0 0 0 0 0 1
3 0 0 0 0
"Viruses;Viruses__na;Viruses__na;Viruses__na;Viruses__na;Viruses__na;virus_sp._ctRwG8" 0 0
0 0 0 0 0 0 0 0 0 0 0 0 0
0 0 0 0 0 0 0 0 0 0 0 0 0
0 0 0 0 0 0 0 0 0 0 0 0 0
0 0 0 0 0 0 0 0 0 0 0 0 0

0 0 0 0 0 0 0 0 0 0 0 0 0
1 0 0 0 0
"Viruses;Viruses__na;Viruses__na;Viruses__na;Viruses__na;Viruses__na;virus_sp._ctusH3" 0 0
0 0 0 0 0 0 0 0 0 0 0 0 0
0 0 0 0 0 0 0 0 0 0 0 0 0
0 0 0 0 0 0 0 0 0 0 0 0 0
0 0 0 0 0 0 0 0 0 0 0 0 0
1 0 0 0 0 0 0 0 0 0 0 0 0
0 0 0 0 0
"Viruses;Viruses__na;Viruses__na;Viruses__na;Viruses__na;Viruses__na;virus_sp._ctuWX8" 0 0
0 0 0 0 0 0 0 0 0 0 0 0 0
0 0 0 0 0 0 0 0 0 0 0 0 0
0 0 0 0 0 0 0 0 0 0 0 0 0
0 1 0 0 0 0 0 1 0 0 0 0 0
0 0 0 0 0 0 5 0 0 0 0 0 1
0 0 0 0 0
"Viruses;Viruses__na;Viruses__na;Viruses__na;Viruses__na;Viruses__na;virus_sp._ctv2g1" 0 0
0 0 0 0 0 0 0 0 0 0 0 0 0
0 0 0 6 3 2 2 0 5 0 0 0 0
0 0 0 0 0 0 0 0 0 0 0 0 0
0 0 0 0 0 0 0 0 0 0 0 0 0
0 0 0 0 0 0 1 0 0 0 0 0 0
0 0 0 0 0
"Viruses;Viruses__na;Viruses__na;Viruses__na;Viruses__na;Viruses__na;virus_sp._ctvdG25" 0 0
0 0 0 0 0 0 0 0 0 0 1 1 1
0 0 1 0 0 0 0 0 0 0 0 0 0
0 0 0 0 0 0 0 0 0 0 0 0 0
0 0 0 0 0 0 0 0 0 0 0 0 0
0 0 0 0 0 0 0 0 0 0 0 0 0
0 0 0 0 0
"Viruses;Viruses__na;Viruses__na;Viruses__na;Viruses__na;Viruses__na;virus_sp._ctviY17" 0 0
0 0 0 0 0 0 0 0 0 0 0 0 0
0 0 0 0 0 0 0 0 0 0 0 0 0
0 0 0 0 0 0 0 0 2 1 2 0 0
27 0 0 0 0 0 0 0 0 0 0 3 0
0 0 0 0 0 0 7 0 0 0 0 0 0
1 0 0 0 0
"Viruses;Viruses__na;Viruses__na;Viruses__na;Viruses__na;Viruses__na;virus_sp._ctWpE22" 0 0
0 0 0 0 0 0 0 0 0 0 0 1 0
0 0 0 0 0 0 0 0 0 0 0 0 0
0 0 0 0 0 0 0 0 0 0 0 0 0
0 0 0 0 0 0 0 0 0 0 0 0 0
0 0 0 0 0 0 0 0 0 0 0 0 0
0 0 0 0 0
"Viruses;Viruses__na;Viruses__na;Viruses__na;Viruses__na;Viruses__na;Virus_sp._ctWxR2" 0 0
0 0 0 0 0 0 0 0 0 0 0 0 0
0 0 0 0 0 0 0 0 0 0 0 0 0
0 0 0 1 0 0 4 0 0 0 0 0 0
0 0 0 0 0 0 0 0 0 0 0 0 0

0 0 0 0 0 0 7 0 0 1 0 0 0
0 0 0 0 0
"Viruses;Viruses__na;Viruses__na;Viruses__na;Viruses__na;Viruses__na;virus_sp._ctX0L1" 0 0
0 0 0 0 0 0 0 0 0 0 0 1 0
0 0 2 0 0 0 1 0 0 0 0 0 0
0 0 0 0 0 0 0 0 0 0 0 0 0
0 0 0 0 0 0 0 0 0 0 0 0 0
0 0 0 0 0 0 0 0 0 0 0 0 0
0 0 0 0 0
"Viruses;Viruses__na;Viruses__na;Viruses__na;Viruses__na;Viruses__na;virus_sp._ctyg714" 0 0
0 0 0 0 0 0 0 0 0 0 0 0 0
0 0 0 0 0 0 0 0 0 1 2 0 0
0 0 0 0 0 0 0 0 0 0 0 0 0
0 0 0 0 0 0 0 1 2 0 0 0 0
0 0 0 0 0 0 1 0 0 0 0 0 4
1 0 0 0 0
"Viruses;Viruses__na;Viruses__na;Viruses__na;Viruses__na;Viruses__na;virus_sp._ctzmw3" 0 0
0 0 0 0 0 0 0 0 0 0 0 0 0
0 0 0 0 0 0 0 1 0 0 0 0 0
0 0 0 0 0 0 0 0 0 0 0 0 0
0 0 0 0 0 0 0 0 0 0 0 0 0
0 0 0 0 0 0 0 0 0 0 0 0 0
0 0 0 0 0
"Viruses;Viruses__na;Viruses__na;Viruses__na;Viruses__na;Viruses__na;Yasminevirus_sp._GU-2018" 0
0 0 0 0 0 0 0 0 0 0 0 0 0
0 0 0 0 0 0 0 0 0 0 0 0 0
0 0 0 0 0 0 0 0 0 0 0 0 0
0 0 0 0 0 0 0 1 0 0 0 0 0
0 0 0 0 0 0 0 0 0 0 0 0 0
0 0 0 0 0 0
"Viruses;Viruses__uc;Viruses__uc;Viruses__uc;Viruses__uc;Viruses__uc;Viruses__uc" 24 37 209
130 1116 888 0 0 18 3 39 0 6139 5578 19457 17367
7188 40528 2482 4324 2038 1629 1204 385 5151 5036 7473 985 5139
174 895 1072 58 17 12277 262 3943 3765 521 190 1226 33525
1860 1871 16109 3128 837 285 6582 2424 4912 335 1007 117413 5044
8245 153 74 1708 1194 11255 3233 157 343 44 8 7096 9058
236 2003 48136 0
